# Supplementary material for: A Plasma Extracellular Vesicle-Derived microRNA Signature as a Potential Biomarker for Subclinical Coronary Atherosclerosis
Source: Int J Mol Sci. 2025 Sep 7;26(17):8727. doi: 10.3390/ijms26178727 (PMC12428963; doi:10.3390/ijms26178727)
Supplement: Supplementary file 1 [file ijms-26-08727-s001.zip › Table_S1.pdf]

Supplementary Table S1. Differentially expressed miRNAs. Affymetrix miRNA 4.0 GeneChip

miRNA_ID	Code_ID	logFC	AveExpr	t	P.Value	adj.P.Val	B
hsa-miR-6849-5p	MIMAT0027598	2.016083833	4.230455497	-5.663877873	5.03E-05	0.896940866	-1.11052398
hsa-miR-3148	MIMAT0015021	1.621518047	3.451815332	-3.538583189	0.003100147	0.896940866	-2.62034435
hsa-miR-595	MIMAT0003263	1.470053644	3.623172034	-3.709247266	0.002195582	0.896940866	-2.47607344
hsa-miR-1180-3p	MIMAT0005825	1.466629107	3.436153635	-2.383920908	0.031217272	0.896940866	-3.653993275
hsa-miR-4793-3p	MIMAT0019966	1.433217479	4.286232768	-2.600201913	0.020456596	0.896940866	-3.457744953
hsa-miR-4701-3p	MIMAT0019799	1.342691076	4.859638404	-3.114384938	0.007315087	0.896940866	-2.99201629
hsa-miR-4440	MIMAT0018958	1.322450225	4.396161215	-1.974877923	0.067550974	0.896940866	-4.015651294
hsa-miR-7844-5p	MIMAT0030419	1.290799839	3.059498216	-2.745038007	0.015352674	0.896940866	-3.325861093
hsa-miR-6790-5p	MIMAT0027480	1.056530719	4.092879494	-2.013746668	0.062893618	0.896940866	-3.982164917
hsa-miR-3064-5p	MIMAT0019864	1.040463565	3.054334679	-1.889595324	0.078884089	0.896940866	-4.088242955
hsa-miR-610	MIMAT0003278	0.98947441	2.185627574	-1.969761373	0.06818688	0.896940866	-4.020041436
hsa-miR-297	MIMAT0004450	0.935491363	3.713037435	-1.336446042	0.201907525	0.905221726	-4.517962617
hsa-miR-4516	MIMAT0019053	0.833352555	8.922339612	-2.109530571	0.052642703	0.896940866	-3.898701812
hsa-miR-6749-5p	MIMAT0027398	0.807460801	6.210110738	-2.565010255	0.021924536	0.896940866	-3.489771567
hsa-miR-4462	MIMAT0018986	0.798795764	2.792045483	-2.106738751	0.052918317	0.896940866	-3.901151955
hsa-miR-574-5p	MIMAT0004795	0.762303407	2.868387566	-1.891313246	0.078639839	0.896940866	-4.086793456
hsa-miR-6126	MIMAT0024599	0.751850879	7.489570933	-1.851967416	0.084407535	0.896940866	-4.119851103
hsa-miR-1268b	MIMAT0018925	0.736411567	5.688618897	-2.106945979	0.052897814	0.896940866	-3.900970122
hsa-miR-4689	MIMAT0019778	0.723639282	4.725920011	-1.544833402	0.143833321	0.896940866	-4.365995035
hsa-miR-6780b-5p	MIMAT0027572	0.720489771	4.699538685	-1.397576468	0.183165687	0.903156384	-4.474853628
hsa-miR-6850-5p	MIMAT0027600	0.714426351	6.945585004	-1.879711049	0.080302698	0.896940866	-4.096572102
hsa-miR-4487	MIMAT0019021	0.708603691	7.255107938	-1.938124255	0.072240958	0.896940866	-4.047090431
hsa-miR-6821-5p	MIMAT0027542	0.708245422	7.45950371	-2.109193917	0.052675868	0.896940866	-3.898997317
hsa-miR-671-5p	MIMAT0003880	0.703958916	2.776839219	-1.592664755	0.132700699	0.896940866	-4.329235238
hsa-miR-206	MIMAT0000462	0.689737242	2.064213939	-1.74194909	0.102586693	0.896940866	-4.210609376
hsa-miR-3663-3p	MIMAT0018085	0.685553786	5.082598353	-1.532124065	0.146921317	0.900006277	-4.375652374
hsa-miR-1281	MIMAT0005939	0.673741675	5.019211407	-1.626751102	0.12522429	0.896940866	-4.302652708
hsa-miR-3178	MIMAT0015055	0.667648795	6.937755367	-1.612063007	0.128400344	0.896940866	-4.314145767
hsa-miR-4706	MIMAT0019806	0.667138019	3.302862593	-1.473130094	0.161996791	0.903074508	-4.419847927
hsa-miR-4449	MIMAT0018968	0.666746058	2.398994733	-1.813022906	0.090485807	0.896940866	-4.152270996
hsa-miR-4463	MIMAT0018987	0.658409894	6.951986317	-1.549824365	0.142635787	0.896940866	-4.36218975
hsa-miR-4484	MIMAT0019018	0.656898173	6.463536763	-1.019252584	0.324715732	0.930208704	-4.718809313
hsa-miR-6871-5p	MIMAT0027642	0.644918685	1.584824681	-4.189149564	0.000838708	0.896940866	-2.090300814
hsa-miR-1825	MIMAT0006765	0.640171543	2.456910274	-2.133518036	0.050328668	0.896940866	-3.877610316
hsa-miR-6756-5p	MIMAT0027412	0.616522847	6.510868953	-1.949404183	0.070771124	0.896940866	-4.037465693
hsa-miR-937-5p	MIMAT0022938	0.613040622	6.165549434	-1.649078063	0.12052615	0.896940866	-4.285073373
hsa-miR-6088	MIMAT0023713	0.6118369	10.21021028	-1.933148164	0.072898103	0.896940866	-4.051329392
hsa-miR-4758-5p	MIMAT0019903	0.605796541	5.583594199	-1.420255023	0.176584927	0.903156384	-4.458538002
hsa-miR-4322	MIMAT0016873	0.604485845	2.355601711	-1.303927913	0.212490566	0.91645315	-4.540358855
hsa-miR-6752-5p	MIMAT0027404	0.6022878	7.426450407	-2.068024845	0.056879868	0.896940866	-3.935023816
hsa-miR-4739	MIMAT0019868	0.601802249	7.0459866	-1.909189264	0.076138335	0.896940866	-4.071678265
hsa-miR-4270	MIMAT0016900	0.595323876	7.059730345	-1.662706232	0.117733983	0.896940866	-4.274279715
hsa-miR-3620-5p	MIMAT0022967	0.586007249	7.182504984	-1.576659463	0.136341107	0.896940866	-4.341607533
hsa-miR-140-3p	MIMAT0004597	0.570185459	7.645905959	-2.231500948	0.041821976	0.896940866	-3.790781693
hsa-miR-4695-5p	MIMAT0019788	0.567183276	6.047897795	-1.396093436	0.183602939	0.903156384	-4.475914591
hsa-miR-6812-5p	MIMAT0027524	0.566339193	3.743915115	-1.126952261	0.277987485	0.927057986	-4.655215402
hsa-miR-4651	MIMAT0019715	0.559611584	6.681706168	-1.598974763	0.131288506	0.896940866	-4.324338076
hsa-miR-6741-5p	MIMAT0027383	0.557361283	2.293051963	-1.374354715	0.190110821	0.903156384	-4.491381123
hsa-miR-4443	MIMAT0018961	0.555048889	6.944242409	-1.084714774	0.295676856	0.927057986	-4.680753824
hsa-miR-4763-3p	MIMAT0019913	0.552481521	7.838431194	-1.513720286	0.151492041	0.902932883	-4.389552393
hsa-miR-6775-5p	MIMAT0027450	0.551068543	5.882441524	-1.977687295	0.06720411	0.896940866	-4.013238967
hsa-miR-4734	MIMAT0019859	0.537275829	7.268003597	-1.577133256	0.13623213	0.896940866	-4.341242309
hsa-mir-6800	MI0022645	0.522563375	7.661627108	-1.713671241	0.107783988	0.896940866	-4.233503495
hsa-mir-6722	MI0022557	0.52088174	4.855869662	-2.051452717	0.058657574	0.896940866	-3.949461542
hsa-miR-6789-5p	MIMAT0027478	0.51960953	7.229297869	-1.487427484	0.158229185	0.903074508	-4.409234314
hsa-miR-6084	MIMAT0023709	0.5105328	1.393937607	-2.264880913	0.039244602	0.896940866	-3.760982807
hsa-miR-150-3p	MIMAT0004610	0.507007719	2.347432231	-1.185992729	0.254612828	0.921665152	-4.618272102
hsa-miR-670-5p	MIMAT0010357	0.503198329	1.443497371	-2.030609049	0.060965796	0.896940866	-3.967565489
hsa-miR-3157-3p	MIMAT0019210	0.502795111	2.714161663	-1.280440354	0.220406645	0.917147059	-4.556296244
hsa-miR-6800-5p	MIMAT0027500	0.494222531	8.118834244	-1.50727707	0.153120309	0.902932883	-4.394394946
hsa-miR-762	MIMAT0010313	0.48941972	8.244942959	-1.257952904	0.228203003	0.919664114	-4.571362843
hsa-mir-6876	MI0022723	0.488900042	1.517074027	-2.215406674	0.043120439	0.896940866	-3.805112313

hsa-miR-4745-5p	MIMAT0019878	0.484870458	7.323191475	-1.614546255	0.12785857	0.896940866	-4.312206741
hsa-miR-6724-5p	MIMAT0025856	0.484012145	9.566209736	-1.36457296	0.193099844	0.903764656	-4.498287917
hsa-miR-4497	MIMAT0019032	0.480214457	9.2472342	-1.336538698	0.201877987	0.905221726	-4.517898261
hsa-miR-6089	MIMAT0023714	0.476947573	10.87628876	-1.454230466	0.167091694	0.903074508	-4.433779902
hsa-miR-6722-3p	MIMAT0025854	0.474904811	6.289593575	-1.21810939	0.242547579	0.919664114	-4.597585531
hsa-miR-5001-5p	MIMAT0021021	0.465230857	8.562130201	-1.445617926	0.169457209	0.903074508	-4.440091131
hsa-miR-3185	MIMAT0015065	0.460627352	5.904239834	-1.503672427	0.154037641	0.903074508	-4.397098646
hsa-miR-4507	MIMAT0019044	0.460097701	6.339809787	-1.117553456	0.281853408	0.927057986	-4.660963595
hsa-miR-6856-5p	MIMAT0027612	0.457357793	2.309870629	-1.152735463	0.26758748	0.927057986	-4.639257797
hsa-mir-4758	MI0017399	0.453821635	1.734979813	-2.131445462	0.05052483	0.896940866	-3.879435433
hsa-miR-6819-5p	MIMAT0027538	0.45179839	2.783035428	-1.037963094	0.316212909	0.930208704	-4.70812525
hsa-miR-1207-5p	MIMAT0005871	0.450638973	6.241168712	-1.993203557	0.065317374	0.896940866	-3.999892831
hsa-miR-1268a	MIMAT0005922	0.442522367	5.982888461	-1.47907751	0.160420545	0.903074508	-4.415440586
hsa-miR-1469	MIMAT0007347	0.432193632	7.953285156	-1.178346546	0.257552253	0.922824015	-4.623136546
hsa-miR-4632-5p	MIMAT0022977	0.427678124	6.42814395	-1.127445444	0.277785735	0.927057986	-4.654912756
hsa-miR-6803-5p	MIMAT0027506	0.426279084	8.727089393	-1.103788578	0.287587727	0.927057986	-4.669314852
hsa-miR-8069	MIMAT0030996	0.42031407	9.79303846	-1.277928454	0.221266912	0.917147059	-4.557988611
hsa-mir-885	MI0005560	0.419520524	6.073241237	-1.817385936	0.089785992	0.896940866	-4.148654349
hsa-miR-6791-5p	MIMAT0027482	0.419321963	8.537346192	-1.166523683	0.262148442	0.924045176	-4.630611751
hsa-miR-4274	MIMAT0016906	0.414095731	1.60136074	-1.692988496	0.111729849	0.896940866	-4.250128274
hsa-miR-5787	MIMAT0023252	0.413274425	8.674710131	-1.194446409	0.251393076	0.920370901	-4.612866683
hsa-miR-4530	MIMAT0019069	0.412568034	7.867412799	-0.931592153	0.36673305	0.930573075	-4.766740386
hsa-miR-4281	MIMAT0016907	0.411971736	7.925087412	-1.247049494	0.232060602	0.919664114	-4.578599403
hsa-miR-6858-5p	MIMAT0027616	0.410721647	7.528169649	-1.104646382	0.287227848	0.927057986	-4.66879676
hsa-miR-4674	MIMAT0019756	0.4092696	5.090414403	-1.158811415	0.265180213	0.926762219	-4.63545738
hsa-miR-3621	MIMAT0018002	0.408351372	4.365214166	-0.893843204	0.385937687	0.930573075	-4.786270729
hsa-miR-6836-3p	MIMAT0027575	0.40652897	1.47588547	-2.599562136	0.020482419	0.896940866	-3.458327356
hsa-miR-1915-3p	MIMAT0007892	0.402389071	9.974889038	-1.22570524	0.239760004	0.919664114	-4.592633666
hsa-mir-4281	MI0015885	0.401981266	6.256567838	-1.003981281	0.331776644	0.930208704	-4.727413025
hsa-mir-638	MI0003653	0.398866599	1.035830793	-3.066193398	0.008062315	0.896940866	-3.035187062
hsa-miR-4687-3p	MIMAT0019775	0.394050734	8.982036534	-1.124123883	0.279146637	0.927057986	-4.656949099
hsa-mir-6776	MI0022621	0.390741413	2.93389994	-1.245339877	0.232670075	0.919664114	-4.579729961
hsa-miR-3940-5p	MIMAT0019229	0.386788204	7.573338772	-1.196051363	0.250785357	0.919981056	-4.611837231
hsa-miR-486-5p	MIMAT0002177	0.386629379	9.314947207	-0.840821615	0.414039088	0.93368138	-4.812535894
hsa-miR-6786-5p	MIMAT0027472	0.385510804	8.655520137	-1.112786	0.283829711	0.927057986	-4.663865113
hsa-miR-455-3p	MIMAT0004784	0.385454496	3.888676135	-0.982035936	0.342114376	0.930208704	-4.739591339
hsa-miR-663a	MIMAT0003326	0.381344614	6.078754758	-1.026612734	0.321351523	0.930208704	-4.71462519
hsa-miR-1306-3p	MIMAT0005950	0.370951814	2.133112281	-1.187867608	0.253896019	0.921246697	-4.617075738
hsa-miR-93-5p	MIMAT0000093	0.368857339	9.960604009	-1.945146741	0.071322681	0.896940866	-4.041100961
hsa-miR-2861	MIMAT0013802	0.367364429	9.779528902	-1.066387823	0.303606728	0.930201434	-4.691597368
hsa-miR-6090	MIMAT0023715	0.365735482	10.79967639	-1.123761769	0.279295305	0.927057986	-4.657170821
hsa-miR-665	MIMAT0004952	0.365117689	4.676069968	-0.742638749	0.469509297	0.951768888	-4.857453813
hsa-miR-6797-3p	MIMAT0027495	0.363859798	1.595080606	-1.498791322	0.155287166	0.903074508	-4.400753497
hsa-mir-4730	MI0017367	0.362933001	1.410248209	-2.473537055	0.026227826	0.896940866	-3.572882243
hsa-mir-1825	MI0008193	0.359314847	1.167518627	-3.954732939	0.001339566	0.896940866	-2.27487763
hsa-miR-3147	MIMAT0015019	0.357929656	1.5475358	-2.441473447	0.027918826	0.896940866	-3.601946502
hsa-miR-23a-5p	MIMAT0004496	0.355686691	4.264151484	-0.851128544	0.408473703	0.931990513	-4.807538455
hsa-miR-4707-5p	MIMAT0019807	0.355328576	7.303982819	-1.112207273	0.284070321	0.927057986	-4.66421668
hsa-miR-378h	MIMAT0018984	0.350578683	3.541329971	-0.753227907	0.463315316	0.951465791	-4.852847051
hsa-miR-6776-5p	MIMAT0027452	0.349734482	3.135539714	-0.915543627	0.374815946	0.930573075	-4.775126777
hsa-miR-4741	MIMAT0019871	0.345886479	6.148307039	-0.929340577	0.367859769	0.930573075	-4.767924361
hsa-mir-297	MI0005775	0.34580363	1.297974514	-1.974863377	0.067552775	0.896940866	-4.015663781
hsa-miR-4466	MIMAT0018993	0.345186797	9.771718657	-0.991741972	0.337514371	0.930208704	-4.73423222
hsa-miR-6883-5p	MIMAT0027666	0.344319895	1.632893622	-1.829163524	0.087920888	0.896940866	-4.138871914
hsa-miR-7110-5p	MIMAT0028117	0.342338135	9.755591843	-0.941617008	0.361745374	0.930573075	-4.761439796
hsa-miR-149-3p	MIMAT0004609	0.339242131	8.479206736	-0.924449878	0.370315346	0.930573075	-4.770487813
hsa-miR-4481	MIMAT0019015	0.332401066	2.133672182	-1.431176048	0.173486042	0.903074508	-4.450620574
hsa-miR-8063	MIMAT0030990	0.330887772	3.011117645	-0.624626082	0.54187838	0.963932703	-4.904778587
hsa-miR-4433b-3p	MIMAT0030414	0.330844056	7.278004284	-0.898701139	0.383428745	0.930573075	-4.783795692
hsa-miR-602	MIMAT0003270	0.330434698	2.58831999	-1.003706868	0.33190452	0.930208704	-4.727566662
hsa-miR-6794-5p	MIMAT0027488	0.326501642	6.085779059	-0.926112891	0.369479101	0.930573075	-4.769617421
hsa-miR-3175	MIMAT0015052	0.322615138	2.516060096	-0.479586621	0.638643098	0.982649571	-4.952480006
hsa-mir-4259	MI0015858	0.321381836	1.252642887	-1.71537729	0.107464013	0.896940866	-4.232127587
hsa-miR-6503-3p	MIMAT0025463	0.319878876	1.262828069	-2.152978203	0.048520914	0.896940866	-3.86044894
hsa-miR-3177-3p	MIMAT0015054	0.317401007	1.195991229	-2.393529138	0.030642035	0.896940866	-3.645316479
hsa-miR-4721	MIMAT0019835	0.317165885	2.01204905	-1.17540502	0.258689973	0.923131598	-4.625001657

hsa-miR-6798-5p	MIMAT0027496	0.308846603	6.711289813	-0.673583183	0.51112463	0.960979619	-4.886050682
hsa-miR-4701-5p	MIMAT0019798	0.303140742	1.171030456	-2.849981963	0.01245087	0.896940866	-3.230454852
hsa-mir-7108	MI0022959	0.302940167	1.465153426	-1.396709305	0.183421255	0.903156384	-4.475474088
hsa-miR-6069	MIMAT0023694	0.297711272	1.404499122	-1.629383506	0.124662281	0.896940866	-4.300586856
hsa-miR-7704	MIMAT0030019	0.297158856	9.956180621	-1.067978112	0.302912491	0.929637747	-4.690662213
hsa-miR-4488	MIMAT0019022	0.296908522	8.619417986	-0.865420801	0.400838278	0.930573075	-4.800521786
hsa-mir-6850	MI0022696	0.295179502	1.127324777	-1.577776987	0.136084186	0.896940866	-4.340745988
hsa-mir-663a	MI0003672	0.2940562	1.051252544	-3.305822002	0.004966708	0.896940866	-2.822240749
hsa-miR-1237-5p	MIMAT0022946	0.291355768	8.485501799	-0.852314644	0.407836423	0.931695939	-4.806959984
hsa-miR-6068	MIMAT0023693	0.289968973	7.154375809	-0.932835758	0.366111753	0.930573075	-4.766085417
hsa-miR-3682-3p	MIMAT0018110	0.287486859	1.113426992	-2.985860028	0.009478768	0.896940866	-3.107469193
hsa-miR-7108-5p	MIMAT0028113	0.287007165	9.050967814	-0.881522194	0.39235062	0.930573075	-4.792496832
hsa-miR-1238-5p	MIMAT0022947	0.285889991	1.106554226	-1.924312275	0.0740783	0.896940866	-4.058845749
hsa-miR-6752-3p	MIMAT0027405	0.285283309	1.123500899	-2.31353719	0.035753318	0.896940866	-3.717376833
hsa-mir-4767	MI0017408	0.282427537	1.264743691	-1.896009449	0.077975609	0.896940866	-4.082828232
hsa-miR-5093	MIMAT0021085	0.282044174	2.099518947	-0.722247486	0.481578506	0.957574873	-4.866160079
hsa-mir-711	MI0012488	0.281651316	2.925097903	-1.216125342	0.243279833	0.919664114	-4.598875257
hsa-miR-4647	MIMAT0019709	0.281214351	1.362549502	-2.48216063	0.025789967	0.896940866	-3.565058294
hsa-miR-1910-5p	MIMAT0007884	0.279482957	1.350671208	-1.455803463	0.16666263	0.903074508	-4.432624664
hsa-miR-4634	MIMAT0019691	0.276652003	2.955109909	-0.575468456	0.573758414	0.967644566	-4.922264287
hsa-miR-3187-5p	MIMAT0019216	0.275489396	1.358477231	-1.461222178	0.165191577	0.903074508	-4.428639049
hsa-miR-6792-5p	MIMAT0027484	0.273526082	1.372494831	-1.167953937	0.261589115	0.924045176	-4.629710459
hsa-miR-8075	MIMAT0031002	0.272583991	6.747412438	-0.779352904	0.448250799	0.950247229	-4.841233334
hsa-miR-8060	MIMAT0030987	0.272299766	1.369133311	-2.447384773	0.027599459	0.896940866	-3.596591444
hsa-miR-6125	MIMAT0024598	0.271959883	10.42184313	-0.811116391	0.430354659	0.94301493	-4.826642336
hsa-miR-8084	MIMAT0031011	0.271472969	1.123753203	-3.03972705	0.008504249	0.896940866	-3.058959292
hsa-miR-1227-5p	MIMAT0022941	0.27078374	6.827638695	-0.78681575	0.444004524	0.948115267	-4.837851353
hsa-miR-6869-5p	MIMAT0027638	0.268596045	9.923036181	-0.922054051	0.371522368	0.930573075	-4.771739434
hsa-miR-3065-3p	MIMAT0015378	0.267494798	1.038842284	-2.707473032	0.0165434	0.896940866	-3.360060225
hsa-let-7a-1	MI0000060	0.2674922	1.113923239	-3.244476703	0.005623373	0.896940866	-2.876318545
hsa-miR-1343-5p	MIMAT0027038	0.267311223	5.718951515	-0.659193377	0.520058391	0.960979619	-4.891689909
hsa-miR-3665	MIMAT0018087	0.267271989	11.25428565	-0.86634792	0.40034627	0.930573075	-4.800063152
hsa-miR-6743-5p	MIMAT0027387	0.266099463	6.604066511	-0.917557985	0.37379477	0.930573075	-4.774080876
hsa-miR-1909-3p	MIMAT0007883	0.264693154	3.411949818	-0.408516489	0.688837417	0.98554565	-4.97148269
hsa-miR-6732-5p	MIMAT0027365	0.264205873	8.414077848	-0.900608368	0.382446757	0.930573075	-4.782820877
hsa-miR-1255b-5p	MIMAT0005945	0.263670172	1.045349721	-3.06230851	0.008125736	0.896940866	-3.038673782
hsa-miR-328-5p	MIMAT0026486	0.263304217	6.637605108	-0.683480172	0.505031959	0.960979619	-4.882107611
hsa-miR-3187-3p	MIMAT0015069	0.262023791	2.09716802	-0.780838073	0.447403727	0.950247229	-4.840562563
hsa-miR-629-3p	MIMAT0003298	0.261334104	1.163245813	-2.517955109	0.024046016	0.896940866	-3.532555235
hsa-miR-6755-5p	MIMAT0027410	0.259816569	0.924220098	-3.152773827	0.006769234	0.896940866	-2.957741231
hsa-miR-8088	MIMAT0031015	0.259740903	0.928606271	-2.071416855	0.056522161	0.896940866	-3.932064013
hsa-miR-4513	MIMAT0019050	0.259471521	1.347406397	-1.518208761	0.150366388	0.902932883	-4.386171617
hsa-mir-1225	MI0006311	0.258886313	1.075011661	-2.209089358	0.043640318	0.896940866	-3.81073045
hsa-miR-4298	MIMAT0016852	0.256945374	3.207706082	-0.781140755	0.447231215	0.950247229	-4.840425719
hsa-miR-195-3p	MIMAT0004615	0.256064638	1.227965133	-1.717194017	0.107124194	0.896940866	-4.230661658
hsa-mir-3161	MI0014191	0.255902942	0.897783488	-4.571830336	0.000394529	0.896940866	-1.805803419
hsa-miR-451a	MIMAT0001631	0.255669521	7.721030447	-0.506256258	0.620253127	0.97746608	-4.944598456
hsa-miR-939-5p	MIMAT0004982	0.254726482	3.324476366	-0.55298892	0.588658633	0.968786597	-4.929813601
hsa-miR-92a-3p	MIMAT0000092	0.253899184	10.47279646	-1.250907853	0.230689712	0.919664114	-4.576043794
hsa-miR-34b-3p	MIMAT0004676	0.252610855	0.984736567	-2.235395012	0.041513344	0.896940866	-3.787310637
hsa-miR-6847-5p	MIMAT0027594	0.251957442	1.413557442	-1.328441106	0.204472577	0.906966856	-4.523511065
hsa-miR-4508	MIMAT0019045	0.249964862	8.447570965	-0.631805219	0.537305394	0.96159396	-4.902113744
hsa-miR-6715b-3p	MIMAT0025843	0.247957117	1.35718081	-2.015073519	0.062739928	0.896940866	-3.981017674
hsa-miR-3162-5p	MIMAT0015036	0.247552918	3.613317519	-0.668908833	0.514016903	0.960979619	-4.887894733
hsa-miR-6736-5p	MIMAT0027373	0.246679996	1.137051311	-1.500359677	0.154884755	0.903074508	-4.39957994
hsa-miR-6892-3p	MIMAT0027685	0.245334101	1.112816245	-2.390755261	0.030807065	0.896940866	-3.647821994
hsa-miR-518b	MIMAT0002844	0.243981303	0.995355758	-2.358580439	0.032783824	0.896940866	-3.676850881
hsa-mir-370	MI0000778	0.242664061	0.908403899	-2.278208364	0.038257741	0.896940866	-3.74905781
hsa-miR-4460	MIMAT0018982	0.242160691	0.966687364	-2.452231201	0.027340221	0.896940866	-3.592199922
hsa-let-7b-5p	MIMAT0000063	0.241218587	12.79121484	-1.629309215	0.124678112	0.896940866	-4.300645183
hsa-mir-4433a	MI0016773	0.240983677	1.16698315	-2.469279029	0.026446632	0.896940866	-3.576744391
hsa-mir-4322	MI0015851	0.240070678	0.963933357	-2.789753937	0.01404355	0.896940866	-3.285178566
hsa-miR-5196-5p	MIMAT0021128	0.240030642	2.826615532	-0.665472407	0.516149198	0.960979619	-4.889242924
hsa-miR-6769b-5p	MIMAT0027620	0.239041453	4.037603347	-0.797789192	0.437807062	0.946184304	-4.832826829
hsa-miR-373-5p	MIMAT0000725	0.238616548	1.018172149	-2.163561956	0.047563192	0.896940866	-3.851097137
hsa-miR-122b-5p	MIMAT0019876	0.238573006	0.932042886	-2.64650719	0.018668635	0.896940866	-3.415581784

hsa-miR-4476	MIMAT0019003	0.237118509	1.180725646	-2.144382042	0.049311907	0.896940866	-3.868035102
hsa-miR-661	MIMAT0003324	0.236986073	0.973983115	-2.427416329	0.028692462	0.896940866	-3.614674351
hsa-miR-6894-5p	MIMAT0027688	0.236765829	1.722076373	-1.233252417	0.23701508	0.919664114	-4.587691328
hsa-miR-6765-5p	MIMAT0027430	0.236397029	6.569035395	-0.654798954	0.522804281	0.960979619	-4.893389793
hsa-miR-6086	MIMAT0023711	0.235987028	2.074584285	-0.779597478	0.448111236	0.950247229	-4.84112295
hsa-miR-6884-5p	MIMAT0027668	0.235471092	0.96011022	-3.747275803	0.002033399	0.896940866	-2.444401823
hsa-miR-6507-3p	MIMAT0025471	0.235002065	1.043196858	-2.37712854	0.031630081	0.896940866	-3.660123949
hsa-miR-5572	MIMAT0022260	0.232267407	1.717825195	-0.968452629	0.348626092	0.930573075	-4.747018492
hsa-miR-6757-5p	MIMAT0027414	0.232254764	1.60533941	-1.0343782	0.317829411	0.930208704	-4.710184389
hsa-miR-6721-5p	MIMAT0025852	0.232166429	1.198701141	-2.278186028	0.038259375	0.896940866	-3.749077809
hsa-miR-4434	MIMAT0018950	0.231743526	0.950080924	-1.741622371	0.102645463	0.896940866	-4.210874954
hsa-miR-3188	MIMAT0015070	0.231197643	2.31967431	-0.534789498	0.600865017	0.973319934	-4.93571801
hsa-miR-4776-5p	MIMAT0019932	0.231150795	1.266990311	-1.837700839	0.08659057	0.896940866	-4.131763136
hsa-miR-550a-5p	MIMAT0004800	0.231022768	1.680307323	-1.139475361	0.272898667	0.927057986	-4.647499075
hsa-mir-4683	MI0017315	0.230731389	0.988192122	-1.977280303	0.06725426	0.896940866	-4.013588519
hsa-miR-509-3p	MIMAT0002881	0.229334833	0.871529481	-2.1697888	0.047007955	0.896940866	-3.84558924
hsa-miR-8089	MIMAT0031016	0.228969223	4.477930535	-0.751055158	0.464582117	0.951465791	-4.853797046
hsa-miR-1249-3p	MIMAT0005901	0.228944465	1.456255573	-1.227775235	0.239004688	0.919664114	-4.591280308
hsa-miR-320a-3p	MIMAT0000510	0.228831111	11.49455152	-0.753712392	0.463033132	0.951465791	-4.852634884
hsa-miR-6727-5p	MIMAT0027355	0.22881045	9.51290841	-0.71407081	0.486470146	0.957942093	-4.869589888
hsa-miR-6799-5p	MIMAT0027498	0.226713452	4.205640862	-0.547822131	0.592111124	0.970056246	-4.931508786
hsa-mir-6777	MI0022622	0.224212813	1.029359727	-2.08778954	0.054824477	0.896940866	-3.91775575
hsa-miR-122-5p	MIMAT0000421	0.223565687	6.249856036	-0.418839207	0.681444705	0.985064407	-4.968904603
hsa-mir-4539	MI0016910	0.222495426	1.629494506	-0.836382189	0.416451445	0.934671969	-4.814672143
hsa-miR-3622b-5p	MIMAT0018005	0.222478763	1.1318315	-2.176654354	0.046402747	0.896940866	-3.83951144
hsa-mir-6739	MI0022584	0.221495866	0.932647915	-2.704653783	0.01663629	0.896940866	-3.362627414
hsa-mir-1224	MI0003764	0.221142069	0.934819879	-1.702488291	0.109902089	0.896940866	-4.24250517
hsa-mir-770	MI0005118	0.21923805	1.083925808	-1.849129452	0.084837834	0.896940866	-4.122223917
hsa-miR-7154-5p	MIMAT0028218	0.218646317	0.99351025	-1.856608808	0.083707998	0.896940866	-4.11596703
hsa-miR-6085	MIMAT0023710	0.218588108	6.329342221	-0.715836283	0.485411456	0.957574873	-4.868852321
hsa-miR-423-5p	MIMAT0004748	0.217901107	7.273993727	-0.66030906	0.519362559	0.960979619	-4.891256672
hsa-mir-6084	MI0020361	0.217683191	1.246484644	-1.900580121	0.077333984	0.896940866	-4.078965094
hsa-mir-1200	MI0006332	0.217598988	0.907178737	-2.568302712	0.021783017	0.896940866	-3.486776119
hsa-miR-7113-3p	MIMAT0028124	0.217574718	1.072720047	-1.560331707	0.14014228	0.896940866	-4.354155147
hsa-mir-876	MI0005542	0.216493802	1.108512331	-2.078448911	0.055787168	0.896940866	-3.925923013
hsa-miR-3149	MIMAT0015022	0.212846117	1.068707644	-1.521048571	0.149657846	0.902932883	-4.384029532
hsa-miR-7848-3p	MIMAT0030423	0.211786153	0.889745113	-2.859157256	0.012224254	0.896940866	-3.222127326
hsa-mir-3663	MI0016064	0.210622853	0.930590675	-2.323039746	0.035106502	0.896940866	-3.708839138
hsa-miR-151a-3p	MIMAT0000757	0.210479165	9.301044661	-1.36222053	0.193824343	0.903764656	-4.499944018
hsa-miR-548g-3p	MIMAT0005912	0.210269119	0.938481835	-1.242484558	0.233690786	0.919664114	-4.581615671
hsa-mir-3158-1	MI0014186	0.210202273	0.922124381	-2.015629531	0.062675627	0.896940866	-3.980536847
hsa-miR-4433a-5p	MIMAT0020956	0.209196936	1.518870241	-1.363422294	0.19345395	0.903764656	-4.499098221
hsa-miR-425-5p	MIMAT0003393	0.20888056	9.027007545	-1.300585802	0.213602936	0.91645315	-4.542639
hsa-miR-3960	MIMAT0019337	0.208698089	11.27220424	-0.598373571	0.558781895	0.96620986	-4.91428274
hsa-miR-7847-3p	MIMAT0030422	0.208589558	4.180810064	-0.836878322	0.416181395	0.934608491	-4.814433891
hsa-let-7f-1-3p	MIMAT0004486	0.207983836	1.282815039	-1.257318826	0.228425947	0.919664114	-4.571784916
hsa-miR-769-5p	MIMAT0003886	0.207815692	1.031551837	-2.020828855	0.062077249	0.896940866	-3.976038335
hsa-miR-338-5p	MIMAT0004701	0.207526569	1.714960335	-0.731520332	0.476067051	0.954010043	-4.86222793
hsa-miR-4253	MIMAT0016882	0.207419106	2.331703949	-0.491472353	0.630415959	0.980033631	-4.949017684
hsa-mir-6827	MI0022672	0.20740995	0.969796656	-2.200384894	0.044366225	0.896940866	-3.818464984
hsa-miR-3910	MIMAT0018184	0.207080211	1.081422032	-1.919237476	0.074763907	0.896940866	-4.063156477
hsa-miR-4787-5p	MIMAT0019956	0.206635059	10.56836319	-0.719208059	0.483393359	0.957574873	-4.867439117
hsa-mir-5703	MI0019310	0.206345947	4.527541323	-0.910344917	0.377460225	0.930573075	-4.777817101
hsa-mir-1913	MI0008334	0.205485554	1.033054046	-1.666893166	0.11688749	0.896940866	-4.270954119
hsa-miR-7975	MIMAT0031178	0.205322182	1.431863938	-0.952487334	0.356390392	0.930573075	-4.755638705
hsa-mir-3677	MI0016078	0.204422812	0.947560631	-1.95513987	0.070034168	0.896940866	-4.032563362
hsa-miR-4750-3p	MIMAT0022979	0.204401817	1.911837616	-0.716990295	0.484720182	0.957574873	-4.868369317
hsa-miR-6729-5p	MIMAT0027359	0.204302149	10.21156349	-0.640511296	0.531788664	0.960979619	-4.8988444
hsa-miR-6890-5p	MIMAT0027680	0.203002957	1.236656056	-1.401947688	0.181881854	0.903156384	-4.471722162
hsa-miR-638	MIMAT0003308	0.202846419	9.698307672	-0.688153892	0.502169542	0.960979619	-4.880227335
hsa-mir-194-1	MI0000488	0.202612265	0.980805103	-1.462539152	0.164835685	0.903074508	-4.427668975
hsa-miR-1233-5p	MIMAT0022943	0.202572103	4.157033376	-0.535453132	0.6004177	0.97307982	-4.935505984
hsa-mir-3918	MI0016424	0.201407832	1.052585823	-1.926156217	0.073830592	0.896940866	-4.057278308
hsa-miR-3691-3p	MIMAT0019224	0.201305839	0.817582013	-2.03367221	0.06062145	0.896940866	-3.964908891
hsa-mir-4737	MI0017374	0.19996379	1.109262708	-2.805461181	0.013610007	0.896940866	-3.270897715
hsa-mir-8083	MI0025919	0.199877275	1.03417463	-1.914379143	0.075425628	0.896940866	-4.06727904

hsa-miR-4523	MIMAT0019061	0.199426701	0.970331493	-1.35298251	0.196690819	0.905071197	-4.506428947
hsa-miR-659-5p	MIMAT0022710	0.198709718	0.948148627	-2.134478632	0.050237989	0.896940866	-3.876764238
hsa-miR-4431	MIMAT0018947	0.198201836	0.919015302	-2.288758337	0.03749311	0.896940866	-3.739607535
hsa-miR-1306-5p	MIMAT0022726	0.198096333	1.318142592	-2.038924517	0.060035151	0.896940866	-3.960350524
hsa-mir-3714	MI0016135	0.197439311	1.065983674	-1.61004825	0.128841355	0.896940866	-4.315717754
hsa-miR-24-3p	MIMAT0000080	0.196917989	11.83376101	-1.389057988	0.185688895	0.903156384	-4.480937668
hsa-mir-1257	MI0006391	0.196895849	1.030768518	-2.378647811	0.0315373	0.896940866	-3.658752919
hsa-miR-4778-5p	MIMAT0019936	0.196474577	1.189955702	-1.919772904	0.074691302	0.896940866	-4.06270188
hsa-miR-541-3p	MIMAT0004920	0.195213031	0.856920786	-2.441187339	0.027934373	0.896940866	-3.602205646
hsa-mir-4455	MI0016801	0.195029404	1.065856755	-1.811469213	0.090736182	0.896940866	-4.153557942
hsa-miR-6818-3p	MIMAT0027537	0.194714155	0.77077974	-1.929571964	0.073373709	0.896940866	-4.054373177
hsa-miR-548as-3p	MIMAT0022268	0.193653103	1.043593189	-2.11606874	0.05200242	0.896940866	-3.892959997
hsa-mir-4490	MI0016852	0.19297128	1.109596208	-1.54773001	0.143137274	0.896940866	-4.363787437
hsa-miR-301a-5p	MIMAT0022696	0.192377225	0.960173578	-2.281092509	0.038047265	0.896940866	-3.746475206
hsa-miR-4713-5p	MIMAT0019820	0.192022885	0.950676377	-2.742758291	0.015422501	0.896940866	-3.327936068
hsa-miR-6770-5p	MIMAT0027440	0.191997587	1.05761528	-2.52368234	0.023777663	0.896940866	-3.527350924
hsa-mir-6880	MI0022727	0.19172863	1.09329956	-2.557885316	0.022233812	0.896940866	-3.496253037
hsa-mir-3960	MI0016964	0.191206038	1.363431942	-0.889384302	0.38825028	0.930573075	-4.788532422
hsa-miR-7107-5p	MIMAT0028111	0.191103672	5.872639135	-0.577396389	0.572489759	0.967644566	-4.921603701
hsa-miR-3917	MIMAT0018191	0.191055509	1.031559906	-1.48746204	0.158220169	0.903074508	-4.409208585
hsa-mir-3150b	MI0016426	0.189969877	0.992210931	-1.552324686	0.142039045	0.896940866	-4.360280714
hsa-mir-5188	MI0018167	0.189401818	0.957648769	-2.432707707	0.028398888	0.896940866	-3.609884429
hsa-miR-6799-3p	MIMAT0027499	0.189209296	0.896500562	-2.955150798	0.010082674	0.896940866	-3.135193502
hsa-miR-6825-5p	MIMAT0027550	0.188990046	1.771483152	-0.922646038	0.371223873	0.930573075	-4.771430424
hsa-mir-1909	MI0008330	0.188809907	1.204472907	-1.375000209	0.189914908	0.903156384	-4.490924189
hsa-miR-127-5p	MIMAT0004604	0.188296383	0.85806221	-1.99368283	0.065259872	0.896940866	-3.999479983
hsa-mir-936	MI0005758	0.187790993	0.940049342	-2.00834414	0.063522948	0.896940866	-3.986833379
hsa-miR-584-3p	MIMAT0022708	0.187554535	1.343947954	-1.08758577	0.294448612	0.927057986	-4.67904199
hsa-miR-106b-5p	MIMAT0000680	0.186921381	8.58540628	-0.957544932	0.353917814	0.930573075	-4.752920786
hsa-miR-136-5p	MIMAT0000448	0.186532689	0.795014988	-2.940086489	0.01039258	0.896940866	-3.148810298
hsa-mir-6745	MI0022590	0.186460892	0.897638538	-2.218091926	0.042901213	0.896940866	-3.802723061
hsa-miR-4276	MIMAT0016904	0.186452444	0.758303884	-2.852997207	0.012375949	0.896940866	-3.227717906
hsa-mir-600	MI0003613	0.186389748	0.975743049	-2.023527475	0.061768733	0.896940866	-3.973701864
hsa-miR-103b	MIMAT0007402	0.18617824	0.936142701	-2.288306021	0.037525596	0.896940866	-3.740012888
hsa-mir-6838	MI0022684	0.185767802	0.985910975	-2.283345467	0.037883608	0.896940866	-3.74445732
hsa-miR-4662a-3p	MIMAT0019732	0.185624235	1.023692277	-2.226405476	0.042229067	0.896940866	-3.795321484
hsa-miR-4520-2-3p	MIMAT0020300	0.185612182	0.910071665	-2.590430168	0.02085444	0.896940866	-3.466639786
hsa-miR-5187-5p	MIMAT0021117	0.185605611	1.376533499	-1.055494417	0.308393642	0.930208704	-4.69797344
hsa-mir-548j	MI0006345	0.184652358	1.030876031	-2.451762143	0.02736521	0.896940866	-3.592624997
hsa-mir-665	MI0005563	0.184222843	1.075953043	-1.399978038	0.182459427	0.903156384	-4.473133978
hsa-miR-3190-5p	MIMAT0015073	0.184174255	1.011696802	-1.944634034	0.071389364	0.896940866	-4.041538533
hsa-miR-1237-3p	MIMAT0005592	0.183872424	0.910121037	-1.836847521	0.086722725	0.896940866	-4.132474332
hsa-miR-5699-5p	MIMAT0027103	0.183622626	1.082906131	-1.396689464	0.183427106	0.903156384	-4.475488281
hsa-mir-4446	MI0016789	0.183450427	1.286062357	-1.144998458	0.270676836	0.927057986	-4.644075234
hsa-miR-4539	MIMAT0019082	0.183384521	2.50325414	-0.520550492	0.610502545	0.975777389	-4.940207309
hsa-miR-6832-5p	MIMAT0027564	0.183338187	1.167271151	-1.827709454	0.088149273	0.896940866	-4.1400812
hsa-mir-8072	MI0025908	0.18330978	1.070192979	-1.713875215	0.107745689	0.896940866	-4.233339029
hsa-miR-6868-3p	MIMAT0027637	0.182692301	0.874568015	-2.855848722	0.012305501	0.896940866	-3.225129847
hsa-miR-3942-5p	MIMAT0018358	0.182629403	1.023006232	-1.486332505	0.158515116	0.903074508	-4.410049404
hsa-miR-548am-3p	MIMAT0019076	0.182285405	0.889797959	-2.248484465	0.040491454	0.896940866	-3.775632777
hsa-miR-4483	MIMAT0019017	0.182071897	0.912669717	-1.47361453	0.161867919	0.903074508	-4.419489347
hsa-miR-202-5p	MIMAT0002810	0.180814191	0.856030921	-1.640065413	0.122403986	0.896940866	-4.292185228
hsa-miR-1228-5p	MIMAT0005582	0.179677599	7.568800621	-0.426197777	0.676195315	0.985064407	-4.967028875
hsa-miR-6864-5p	MIMAT0027628	0.179344476	0.979072757	-2.051242024	0.0586805	0.896940866	-3.949644852
hsa-miR-6762-5p	MIMAT0027424	0.17911991	1.041270043	-1.192001854	0.252320891	0.920483828	-4.614432702
hsa-miR-30c-1-3p	MIMAT0004674	0.17860349	1.03255855	-1.943969538	0.071475874	0.896940866	-4.042105585
hsa-mir-466	MI0014157	0.178485609	1.25200995	-1.232137155	0.237419151	0.919664114	-4.588423056
hsa-miR-6836-5p	MIMAT0027574	0.178476605	1.237632722	-0.842217471	0.413282482	0.93368138	-4.811862184
hsa-mir-4666a	MI0017296	0.178144037	1.029681403	-2.413070045	0.029502995	0.896940866	-3.627654128
hsa-miR-6165	MIMAT0024782	0.177695659	1.818503448	-0.771049219	0.453005366	0.951465791	-4.84496281
hsa-mir-6836	MI0022682	0.177678926	1.329679941	-1.030156339	0.3197408	0.930208704	-4.712602062
hsa-miR-3196	MIMAT0015080	0.177304777	8.807163181	-0.533144108	0.601974793	0.973599219	-4.936242626
hsa-miR-4458	MIMAT0018980	0.176416708	1.019806146	-1.199167062	0.24960883	0.919664114	-4.609835832
hsa-miR-6722-5p	MIMAT0025853	0.175508337	1.127498537	-1.202835392	0.248229093	0.919664114	-4.607474512
hsa-mir-8060	MI0025896	0.17469398	1.068219266	-2.409136694	0.029728991	0.896940866	-3.631210989
hsa-mir-8075	MI0025911	0.174403003	0.986316177	-1.29407524	0.215783206	0.917147059	-4.547069069

hsa-miR-6768-5p	MIMAT0027436	0.1741279	1.115581006	-1.67793168	0.114681053	0.896940866	-4.262165301
hsa-mir-655	MI0003677	0.17404284	0.920100696	-1.969665581	0.068198837	0.896940866	-4.020123587
hsa-mir-5697	MI0019304	0.174014469	0.997548864	-2.378022941	0.031575429	0.896940866	-3.659316834
hsa-miR-6513-5p	MIMAT0025482	0.173990177	1.05231686	-1.401216762	0.182096013	0.903156384	-4.47224623
hsa-miR-6746-3p	MIMAT0027393	0.17389966	0.917665378	-2.418319163	0.029203941	0.896940866	-3.622906197
hsa-miR-342-3p	MIMAT0000753	0.173881826	6.844137436	-0.633403069	0.536290515	0.961424653	-4.901516806
hsa-miR-6751-3p	MIMAT0027403	0.173506891	0.833091842	-2.713514075	0.016346046	0.896940866	-3.354559499
hsa-miR-4482-3p	MIMAT0020958	0.173098605	0.947502849	-1.535654275	0.146058007	0.897988521	-4.372974637
hsa-mir-6884	MI0022731	0.172605003	0.958399948	-1.789012892	0.094424291	0.896940866	-4.172101913
hsa-miR-370-5p	MIMAT0026483	0.17222347	0.791446879	-2.178844918	0.046211175	0.896940866	-3.837571145
hsa-mir-7854	MI0025524	0.172045754	1.083806291	-1.159353601	0.264966205	0.926762219	-4.635117516
hsa-miR-6776-3p	MIMAT0027453	0.171901145	1.011658017	-1.975416739	0.067484323	0.896940866	-4.015188728
hsa-miR-1182	MIMAT0005827	0.171867424	1.431247391	-1.147224846	0.26978509	0.927057986	-4.642691502
hsa-miR-6880-3p	MIMAT0027661	0.171467794	1.07089056	-1.327524764	0.204767868	0.907676232	-4.52414475
hsa-mir-3926-2	MI0016437	0.171048313	0.835250668	-2.193544964	0.044944516	0.896940866	-3.824537323
hsa-mir-3141	MI0014165	0.170951976	1.374696207	-1.291319577	0.216711349	0.917147059	-4.548939454
hsa-miR-4505	MIMAT0019041	0.170719564	6.475320791	-0.431194296	0.672640818	0.984005175	-4.965737279
hsa-miR-548ai	MIMAT0018989	0.170706676	1.231742483	-1.707612741	0.108927023	0.896940866	-4.238383988
hsa-miR-570-5p	MIMAT0022707	0.170706676	1.231742483	-1.707612741	0.108927023	0.896940866	-4.238383988
hsa-miR-1180-5p	MIMAT0026735	0.170464732	1.573399238	-0.832595472	0.418516355	0.935967201	-4.816486546
hsa-miR-4700-3p	MIMAT0019797	0.170050061	1.052199165	-2.214232464	0.043216631	0.896940866	-3.806156864
hsa-let-7b	MI0000063	0.169459094	0.9592045	-2.165976414	0.04734718	0.896940866	-3.84896196
hsa-miR-135b-3p	MIMAT0004698	0.16944995	1.004907553	-1.725650068	0.105554838	0.896940866	-4.223828142
hsa-mir-523	MI0003153	0.169380418	0.894416799	-2.424289843	0.028867278	0.896940866	-3.617503908
hsa-mir-7-2	MI0000264	0.168916023	0.923542155	-2.003565808	0.06408435	0.896940866	-3.990958741
hsa-miR-6772-5p	MIMAT0027444	0.168414927	1.110459436	-1.538250391	0.145425869	0.896940866	-4.3710031
hsa-miR-4650-3p	MIMAT0019714	0.168399052	1.063395654	-1.725061828	0.105663354	0.896940866	-4.224304057
hsa-miR-941	MIMAT0004984	0.167296571	1.216355355	-1.319692586	0.207305789	0.911337372	-4.529548737
hsa-miR-6892-5p	MIMAT0027684	0.167240942	1.072070981	-1.818049274	0.089680017	0.896940866	-4.148104142
hsa-miR-210-5p	MIMAT0026475	0.167235405	1.487301311	-1.569265418	0.13805145	0.896940866	-4.347299068
hsa-mir-3191	MI0014236	0.166977778	1.122664594	-1.277185588	0.221521836	0.917147059	-4.55848866
hsa-mir-486-2	MI0023622	0.166934264	1.821951188	-0.642229383	0.530703733	0.960979619	-4.898194344
hsa-mir-6509	MI0022221	0.166853126	0.905859196	-2.444811467	0.027738055	0.896940866	-3.598922789
hsa-miR-4735-3p	MIMAT0019861	0.166822551	1.037162688	-1.401974337	0.18187405	0.903156384	-4.471703052
hsa-mir-4503	MI0016866	0.166233398	1.127126182	-1.851692326	0.084449159	0.896940866	-4.120081175
hsa-mir-8066	MI0025902	0.166223	0.939366543	-2.868623093	0.011994688	0.896940866	-3.213539032
hsa-mir-218-1	MI0000294	0.166139043	0.981011945	-2.18242051	0.045900057	0.896940866	-3.83440295
hsa-mir-4286	MI0015894	0.16574715	1.0138314	-1.832503791	0.08739824	0.896940866	-4.136092328
hsa-mir-3916	MI0016422	0.165017814	1.052041471	-2.104504337	0.053139862	0.896940866	-3.903112201
hsa-mir-4720	MI0017355	0.164510104	0.92662478	-2.133253866	0.050353631	0.896940866	-3.877842973
hsa-miR-21-3p	MIMAT0004494	0.16416463	1.091386832	-1.504390861	0.153854442	0.903074508	-4.396560091
hsa-miR-145-3p	MIMAT0004601	0.163968841	0.831715777	-2.452284882	0.027337363	0.896940866	-3.592151274
hsa-mir-3158-2	MI0014187	0.163926606	1.001683041	-1.865761699	0.08234368	0.896940866	-4.108295244
hsa-mir-6825	MI0022670	0.163367423	1.729963758	-0.885768425	0.39013248	0.930573075	-4.790359431
hsa-mir-2116	MI0010635	0.162894718	1.061605861	-1.278918645	0.220927478	0.917147059	-4.557321761
hsa-mir-628	MI0003642	0.162860129	1.001077106	-1.271104832	0.223617271	0.918865246	-4.562574075
hsa-miR-216a-5p	MIMAT0000273	0.162778741	1.087891521	-2.389712139	0.030869343	0.896940866	-3.648764084
hsa-miR-877-3p	MIMAT0004950	0.162126533	1.023383682	-1.827024393	0.088257056	0.896940866	-4.140650786
hsa-mir-378c	MI0015825	0.16209971	1.058967776	-1.221198251	0.241410983	0.919664114	-4.595574563
hsa-mir-6090	MI0020367	0.161821493	2.935706659	-0.344171988	0.735635551	0.989764242	-4.986142851
hsa-miR-4795-5p	MIMAT0019968	0.161729549	0.93916325	-2.015360797	0.062706698	0.896940866	-3.980769249
hsa-miR-6851-5p	MIMAT0027602	0.161357455	1.200836429	-1.902042071	0.077129763	0.896940866	-4.077728642
hsa-miR-4683	MIMAT0019768	0.161335327	0.912871268	-1.869668927	0.081767363	0.896940866	-4.105015336
hsa-miR-1323	MIMAT0005795	0.16091517	0.982017097	-2.125895943	0.051053562	0.896940866	-3.884319818
hsa-mir-320c-1	MI0003778	0.160617387	1.024777874	-2.524540673	0.023737693	0.896940866	-3.52657088
hsa-mir-5688	MI0019292	0.160340283	1.026106253	-2.209358068	0.043618087	0.896940866	-3.810491561
hsa-mir-4693	MI0017326	0.160292073	0.989269368	-1.929596066	0.073370495	0.896940866	-4.054352671
hsa-mir-6846	MI0022692	0.159937147	1.10202796	-1.507054458	0.153176828	0.902932883	-4.394562033
hsa-miR-5694	MIMAT0022487	0.159751061	0.788674206	-2.6505694	0.018519183	0.896940866	-3.411882294
hsa-miR-576-3p	MIMAT0004796	0.158641081	1.042564958	-1.675691614	0.115125859	0.896940866	-4.263951299
hsa-miR-1273c	MIMAT0015017	0.158603408	0.996996836	-2.379163647	0.031505857	0.896940866	-3.658287383
hsa-mir-29a	MI0000087	0.158425623	0.873885463	-1.716609998	0.107233332	0.896940866	-4.231132992
hsa-miR-1228-3p	MIMAT0005583	0.158357614	2.084929918	-0.420224655	0.680455052	0.985064407	-4.968553857
hsa-mir-4466	MI0016817	0.15787263	1.39885677	-0.794371305	0.439731459	0.946841421	-4.834398387
hsa-miR-5006-5p	MIMAT0021033	0.157601862	1.69545622	-0.728273172	0.477992689	0.955328398	-4.863610021
hsa-miR-3120-5p	MIMAT0019198	0.157568019	1.008743482	-1.811056496	0.090802794	0.896940866	-4.153899716

hsa-miR-1292-3p	MIMAT0022948	0.157338429	1.191446558	-1.564367507	0.139194437	0.896940866	-4.351060721
hsa-mir-3167	MI0014198	0.157153575	0.886909474	-1.799833793	0.09263085	0.896940866	-4.163179606
hsa-miR-3193	MIMAT0015077	0.157136095	0.996940902	-1.847577051	0.085074042	0.896940866	-4.123521201
hsa-mir-1302-8	MI0006369	0.1569169	1.011517402	-1.831049314	0.087625479	0.896940866	-4.137302942
hsa-miR-3938	MIMAT0018353	0.156808792	1.054386921	-1.217249749	0.242864637	0.919664114	-4.598144529
hsa-mir-1324	MI0006657	0.156574449	0.919641679	-1.847157912	0.085137917	0.896940866	-4.123871377
hsa-mir-6751	MI0022596	0.156352462	0.886474546	-1.782964988	0.095440001	0.896940866	-4.177077695
hsa-mir-4727	MI0017364	0.156347867	0.813907243	-2.291876829	0.037269855	0.896940866	-3.736812378
hsa-miR-3181	MIMAT0015061	0.156137961	0.951885606	-1.604249543	0.130117897	0.896940866	-4.320235998
hsa-miR-3667-3p	MIMAT0018090	0.156056199	0.913384004	-1.547421436	0.143211287	0.896940866	-4.364022726
hsa-mir-6867	MI0022714	0.155237819	1.162622239	-1.513500819	0.151547262	0.902932883	-4.389717544
hsa-mir-212	MI0000288	0.154980233	0.854537988	-1.70372999	0.109665125	0.896940866	-4.241507152
hsa-miR-4775	MIMAT0019931	0.154755501	0.922352903	-1.221839648	0.24117549	0.919664114	-4.595156522
hsa-miR-5003-3p	MIMAT0021026	0.154566976	0.850724419	-1.535399072	0.146120272	0.897988521	-4.373168337
hsa-miR-1236-5p	MIMAT0022945	0.154378013	1.393439679	-1.163589121	0.263298916	0.924981202	-4.632458397
hsa-mir-6890	MI0022737	0.154004668	1.127648887	-1.446828956	0.16912292	0.903074508	-4.439205127
hsa-mir-4505	MI0016868	0.153743972	0.920282961	-2.508270885	0.024506383	0.896940866	-3.541353051
hsa-miR-3689f	MIMAT0019010	0.15295904	0.888071665	-1.980068881	0.066911332	0.896940866	-4.011192971
hsa-miR-130a-5p	MIMAT0004593	0.152785014	0.836935255	-2.21135045	0.043453575	0.896940866	-3.808720063
hsa-mir-4659a	MI0017287	0.152583078	0.960179045	-1.804126694	0.091927761	0.896940866	-4.159633006
hsa-mir-596	MI0003608	0.152359788	0.87271176	-1.76278364	0.098899587	0.896940866	-4.193623531
hsa-mir-4308	MI0015839	0.152108574	0.983458867	-1.936764176	0.072420037	0.896940866	-4.048249462
hsa-mir-6849	MI0022695	0.152104756	1.155600756	-1.325293758	0.205488242	0.908883572	-4.525686322
hsa-miR-3529-5p	MIMAT0019828	0.151977873	0.914653687	-1.794380602	0.093530845	0.896940866	-4.167679134
hsa-miR-4708-5p	MIMAT0019809	0.15193071	0.94324878	-2.018272912	0.062370752	0.896940866	-3.978250272
hsa-mir-1272	MI0006408	0.151809068	1.013873204	-1.990701387	0.065618334	0.896940866	-4.002047628
hsa-mir-3940	MI0016597	0.1513042	0.991450586	-2.311058619	0.035923865	0.896940866	-3.719602638
hsa-miR-4756-5p	MIMAT0019899	0.15106648	0.973659885	-1.417681412	0.177321801	0.903156384	-4.460398104
hsa-mir-548n	MI0006399	0.150987658	0.970372181	-2.483465196	0.025724338	0.896940866	-3.563874453
hsa-miR-6833-3p	MIMAT0027567	0.150733719	0.899655077	-1.543186142	0.14423043	0.896940866	-4.36724938
hsa-mir-5580	MI0019135	0.15072635	0.965980792	-2.161193849	0.047775946	0.896940866	-3.853190692
hsa-miR-16-5p	MIMAT0000069	0.150686898	11.60834154	-0.868183045	0.399373582	0.930573075	-4.799154096
hsa-miR-6858-3p	MIMAT0027617	0.150577447	1.125537436	-1.265187717	0.225671327	0.919664114	-4.566536246
hsa-mir-216b	MI0005569	0.150541371	0.867309296	-2.357229269	0.032869407	0.896940866	-3.678068541
hsa-mir-3658	MI0016058	0.150489219	1.17685009	-1.585965964	0.134214105	0.896940866	-4.334422139
hsa-miR-320b	MIMAT0005792	0.150449905	11.32161439	-0.511202208	0.616870837	0.976745744	-4.943092207
hsa-mir-1253	MI0006387	0.149984196	0.971861454	-2.097615417	0.053828302	0.896940866	-3.909151832
hsa-miR-4710	MIMAT0019815	0.149806156	1.954927018	-0.615104624	0.547976363	0.965790957	-4.908269382
hsa-miR-4798-3p	MIMAT0019975	0.149691167	0.983959766	-1.991195665	0.065558782	0.896940866	-4.001622048
hsa-miR-548aj-3p	MIMAT0018990	0.149547781	1.025661973	-1.866843605	0.082183736	0.896940866	-4.107387336
hsa-miR-506-5p	MIMAT0022701	0.149163776	0.942783129	-1.73825912	0.103252163	0.896940866	-4.213607392
hsa-miR-4696	MIMAT0019790	0.148918229	0.81892787	-1.859386828	0.083291787	0.896940866	-4.113640267
hsa-mir-548at	MI0019137	0.147969267	0.956117305	-1.852680501	0.084299722	0.896940866	-4.119254645
hsa-miR-5590-3p	MIMAT0022300	0.14747743	1.050731614	-2.164833507	0.047449318	0.896940866	-3.849972748
hsa-mir-4476	MI0016828	0.147431381	0.865547891	-2.461727936	0.026838946	0.896940866	-3.583591677
hsa-miR-548aw	MIMAT0022471	0.147111595	0.972441793	-1.690125306	0.1122859	0.896940866	-4.252421524
hsa-miR-4707-3p	MIMAT0019808	0.146899393	1.059231832	-1.824964953	0.088581781	0.896940866	-4.142362505
hsa-mir-3146	MI0014172	0.146723456	0.946996131	-1.921819914	0.074414307	0.896940866	-4.060963426
hsa-miR-6818-5p	MIMAT0027536	0.146473498	0.877769118	-2.096299966	0.053960692	0.896940866	-3.910304418
hsa-miR-106a-3p	MIMAT0004517	0.145972894	0.795548243	-2.560813698	0.022106194	0.896940866	-3.493589248
hsa-miR-324-3p	MIMAT0000762	0.145663074	3.09827816	-0.395846811	0.697955978	0.985651988	-4.974561788
hsa-mir-552	MI0003557	0.145493167	0.853030267	-1.995447572	0.065048536	0.896940866	-3.997959514
hsa-miR-382-3p	MIMAT0022697	0.145277221	1.018827098	-1.642691759	0.12185418	0.896940866	-4.290114962
hsa-mir-4774	MI0017417	0.145162095	1.033161487	-1.254519941	0.229412097	0.919664114	-4.573646166
hsa-mir-3197	MI0014245	0.144597981	1.09596685	-1.910939257	0.075897339	0.896940866	-4.070195417
hsa-miR-3924	MIMAT0018199	0.144591183	0.969922653	-1.268021637	0.224685714	0.919664114	-4.56464026
hsa-miR-4717-3p	MIMAT0019830	0.144574713	1.911015537	-0.521040276	0.610169783	0.97572307	-4.940054795
hsa-miR-7706	MIMAT0030021	0.144235885	0.956138731	-1.909112246	0.076148957	0.896940866	-4.071743513
hsa-mir-6796	MI0022641	0.143738536	1.225024841	-0.919711601	0.372705106	0.930573075	-4.772960528
hsa-miR-6763-5p	MIMAT0027426	0.143642183	3.950044977	-0.465287191	0.648606462	0.982649571	-4.956538025
hsa-mir-6801	MI0022646	0.143399708	0.970629077	-3.135502081	0.007009642	0.896940866	-2.973149033
hsa-mir-4460	MI0016806	0.143183014	1.008904846	-1.660318813	0.118219035	0.896940866	-4.276174003
hsa-mir-1267	MI0006404	0.143147265	0.967939657	-1.915978722	0.075207179	0.896940866	-4.065922175
hsa-miR-7-2-3p	MIMAT0004554	0.143048625	1.013455255	-1.650569675	0.120217778	0.896940866	-4.28389432
hsa-mir-4478	MI0016831	0.143042256	0.87690947	-1.733944296	0.104035128	0.896940866	-4.217109099
hsa-miR-4724-3p	MIMAT0019842	0.142885764	0.769572539	-1.947471812	0.071020985	0.896940866	-4.039116053

hsa-miR-4669	MIMAT0019749	0.142742957	1.697570831	-1.253249223	0.229860921	0.919664114	-4.574490208
hsa-miR-568	MIMAT0003232	0.142619467	0.976039658	-1.755648915	0.100148866	0.896940866	-4.199451339
hsa-mir-604	MI0003617	0.142083734	0.930069192	-1.567877827	0.138374447	0.896940866	-4.348365444
hsa-mir-612	MI0003625	0.141790065	0.963542864	-1.548475858	0.142958512	0.896940866	-4.363218608
hsa-miR-520h	MIMAT0002867	0.141672469	0.936671629	-1.576045599	0.136482411	0.896940866	-4.342080637
hsa-mir-4305	MI0015835	0.14132606	1.038376021	-1.695608524	0.111223128	0.896940866	-4.248028027
hsa-miR-6732-3p	MIMAT0027366	0.140525217	1.226260306	-0.76571093	0.456078558	0.951465791	-4.8473417
hsa-mir-499a	MI0003183	0.140495049	0.996850088	-1.729408167	0.104863865	0.896940866	-4.220785746
hsa-mir-140	MI0000456	0.140487994	0.959047434	-1.161012052	0.264312409	0.926587765	-4.634077189
hsa-miR-1245b-3p	MIMAT0019951	0.140066079	0.927007373	-1.655578598	0.119187238	0.896940866	-4.279930805
hsa-miR-4789-3p	MIMAT0019960	0.139883501	0.871584625	-1.881952312	0.079979035	0.896940866	-4.094685075
hsa-miR-4698	MIMAT0019793	0.1398032	1.110043425	-1.599842368	0.131095345	0.896940866	-4.32366388
hsa-miR-4692	MIMAT0019783	0.139669899	1.143996175	-1.030138431	0.319748925	0.930208704	-4.7126123
hsa-miR-519a-3p	MIMAT0002869	0.139626513	0.895477289	-1.871609809	0.081482428	0.896940866	-4.103384978
hsa-mir-6731	MI0022576	0.139574034	0.878985752	-2.346238054	0.033573476	0.896940866	-3.687969293
hsa-mir-211	MI0000287	0.139356886	0.937066754	-2.352138755	0.03319374	0.896940866	-3.682654998
hsa-miR-6757-3p	MIMAT0027415	0.139205989	0.919850572	-1.980584027	0.066848156	0.896940866	-4.010750294
hsa-miR-1911-5p	MIMAT0007885	0.139205423	1.014906432	-1.36327219	0.193500182	0.903764656	-4.499203891
hsa-miR-4769-3p	MIMAT0019923	0.139038503	1.200697599	-0.720034876	0.482899258	0.957574873	-4.867091661
hsa-miR-3124-5p	MIMAT0014986	0.13887561	1.201531228	-0.890743001	0.387544608	0.930573075	-4.787844268
hsa-miR-711	MIMAT0012734	0.138597027	1.037483187	-1.0315834	0.319093789	0.930208704	-4.711785736
hsa-miR-1256	MIMAT0005907	0.138430771	0.916305967	-1.730781573	0.104612339	0.896940866	-4.219673071
hsa-miR-7107-3p	MIMAT0028112	0.138408946	0.809590285	-1.63498063	0.123474535	0.896940866	-4.296188289
hsa-miR-1910-3p	MIMAT0026917	0.137973944	1.055868255	-1.751196203	0.100935554	0.896940866	-4.203082602
hsa-miR-2467-5p	MIMAT0019952	0.137908085	0.871640569	-1.933276604	0.072881074	0.896940866	-4.051220032
hsa-mir-1306	MI0006443	0.137903369	1.086889857	-1.191576704	0.252482523	0.92077882	-4.614704817
hsa-mir-374b	MI0005566	0.137851549	0.993951789	-1.361803384	0.193953045	0.903764656	-4.500237487
hsa-mir-6863	MI0022710	0.137845821	0.958506863	-1.523441165	0.149063071	0.902931596	-4.382222922
hsa-miR-4665-3p	MIMAT0019740	0.137704804	1.189286343	-1.277691691	0.221348135	0.917147059	-4.558148007
hsa-mir-4420	MI0016757	0.137548959	0.8995881	-1.365081043	0.192943654	0.903764656	-4.497929978
hsa-mir-5692c-1	MI0019288	0.137481857	0.883141883	-1.463812753	0.164492122	0.903074508	-4.426730329
hsa-mir-606	MI0003619	0.137341537	1.071141512	-1.208395355	0.246149127	0.919664114	-4.603885399
hsa-mir-4674	MI0017305	0.137088993	1.772724453	-0.553887134	0.58805949	0.968608702	-4.929517376
hsa-miR-548aa	MIMAT0018447	0.136988991	1.114470162	-1.634973955	0.123475946	0.896940866	-4.296193539
hsa-miR-548t-3p	MIMAT0022730	0.136988991	1.114470162	-1.634973955	0.123475946	0.896940866	-4.296193539
hsa-miR-6790-3p	MIMAT0027481	0.136774558	1.067030659	-1.577309066	0.136191712	0.896940866	-4.34110677
hsa-miR-6738-3p	MIMAT0027378	0.136563682	0.901976688	-1.469918026	0.162853445	0.903074508	-4.422223646
hsa-miR-6795-3p	MIMAT0027491	0.136448463	1.004299582	-1.30497596	0.212142694	0.91645315	-4.53964299
hsa-mir-6071	MI0020348	0.136158635	1.007438979	-1.35276357	0.196759168	0.905071197	-4.506582278
hsa-miR-3934-3p	MIMAT0022975	0.136075722	0.910241876	-1.126687957	0.278095651	0.927057986	-4.655377553
hsa-mir-6789	MI0022634	0.136065296	1.226466775	-1.519189639	0.150121337	0.902932883	-4.385432006
hsa-mir-4632	MI0017259	0.13585729	0.92800942	-1.771339029	0.097419687	0.896940866	-4.186620302
hsa-mir-4423	MI0016760	0.135596362	0.88621501	-1.891311889	0.078640031	0.896940866	-4.086794601
hsa-miR-4720-3p	MIMAT0019834	0.135560115	0.872335906	-1.081585019	0.297020116	0.927057986	-4.682615901
hsa-mir-6080	MI0020357	0.135557797	1.043028005	-1.721922133	0.106244205	0.896940866	-4.226842845
hsa-mir-548s	MI0014141	0.1355185	1.036202086	-1.745418777	0.101964392	0.896940866	-4.207787482
hsa-mir-1271	MI0003814	0.135193664	0.983946383	-1.349038284	0.197925086	0.905221726	-4.509188652
hsa-mir-3133	MI0014153	0.135068658	1.064320619	-1.813648697	0.090385135	0.896940866	-4.151752502
hsa-miR-934	MIMAT0004977	0.134960022	0.845504375	-1.851550197	0.084470672	0.896940866	-4.120200039
hsa-mir-1295a	MI0006357	0.134487448	0.96199365	-1.909166467	0.076141479	0.896940866	-4.071697579
hsa-mir-26b	MI0000084	0.134158242	1.118670655	-1.159468371	0.264920921	0.926762219	-4.635045559
hsa-miR-4773	MIMAT0019928	0.134154346	0.994710184	-1.760603395	0.099279878	0.896940866	-4.195405618
hsa-mir-6779	MI0022624	0.134078914	0.863870773	-2.207467797	0.043774703	0.896940866	-3.812171901
hsa-mir-567	MI0003573	0.134001256	0.927581506	-1.38409367	0.1871724	0.903156384	-4.484471956
hsa-miR-365a-3p	MIMAT0000710	0.133437732	1.077993054	-1.002843232	0.332307201	0.930208704	-4.728049968
hsa-miR-365b-3p	MIMAT0022834	0.133437732	1.077993054	-1.002843232	0.332307201	0.930208704	-4.728049968
hsa-mir-7977	MI0025753	0.133433072	0.931548981	-1.084821728	0.295631032	0.927057986	-4.680690116
hsa-mir-369	MI0000777	0.133249367	0.908913941	-1.60250525	0.130504007	0.896940866	-4.321593334
hsa-miR-6745	MIMAT0027391	0.133143527	1.135088014	-1.474154027	0.1617245	0.903074508	-4.419089925
hsa-miR-548j-5p	MIMAT0005875	0.13313257	0.967912112	-1.664186822	0.117434039	0.896940866	-4.27310422
hsa-mir-1292	MI0006433	0.132973604	0.93940486	-1.600731305	0.130897686	0.896940866	-4.322972895
hsa-mir-4271	MI0015879	0.132758847	0.972503734	-1.356708562	0.195530553	0.904897643	-4.503816899
hsa-miR-6866-5p	MIMAT0027632	0.132749931	0.856883342	-2.024792689	0.061624573	0.896940866	-3.972606067
hsa-mir-19b-1	MI0000074	0.132485034	0.955530198	-1.509069792	0.152665799	0.902932883	-4.393048837
hsa-mir-3672	MI0016073	0.13243488	0.970014597	-2.168779953	0.047097502	0.896940866	-3.846481898
hsa-miR-20b-3p	MIMAT0004752	0.132335869	0.906819694	-1.543208298	0.144225083	0.896940866	-4.367232514

hsa-miR-4715-3p	MIMAT0019825	0.132225915	0.887875466	-1.278746135	0.220986584	0.917147059	-4.557437965
hsa-miR-4715-5p	MIMAT0019824	0.132070063	1.084922685	-0.650774318	0.525326314	0.960979619	-4.894937474
hsa-miR-4297	MIMAT0016846	0.132026274	0.845421893	-1.245203232	0.232718843	0.919664114	-4.579820276
hsa-mir-3137	MI0014160	0.131812344	1.001961429	-1.572685641	0.137258047	0.896940866	-4.344668277
hsa-mir-93	MI0000095	0.131448311	1.280320665	-1.477065595	0.160952324	0.903074508	-4.41693275
hsa-mir-6822	MI0022667	0.131445195	0.929027285	-1.602664526	0.130468709	0.896940866	-4.321469427
hsa-mir-610	MI0003623	0.131245665	0.948751385	-1.600368092	0.130978417	0.896940866	-4.323255252
hsa-mir-7974	MI0025750	0.13124509	0.880150576	-1.46984801	0.16287216	0.903074508	-4.422275395
hsa-miR-4796-3p	MIMAT0019971	0.131199034	0.969803283	-1.17112955	0.260350493	0.923131598	-4.627706341
hsa-mir-3170	MI0014201	0.130855463	0.91467739	-1.756378435	0.100020493	0.896940866	-4.198855976
hsa-mir-6860	MI0022707	0.13085401	1.371967625	-0.898874642	0.383339342	0.930573075	-4.783707084
hsa-mir-4497	MI0016859	0.130681206	0.907308743	-1.614096925	0.127956455	0.896940866	-4.31255772
hsa-miR-7973	MIMAT0031176	0.130517971	1.106404973	-1.534746694	0.146279544	0.898411941	-4.373663405
hsa-mir-331	MI0000812	0.130433088	0.939716158	-1.78315906	0.095407258	0.896940866	-4.17691815
hsa-miR-1303	MIMAT0005891	0.130393892	0.925673373	-1.074228178	0.300195349	0.927544599	-4.686976263
hsa-miR-890	MIMAT0004912	0.130169123	1.000219062	-1.622051051	0.126233161	0.896940866	-4.306336637
hsa-mir-3615	MI0016005	0.129933834	0.967910935	-1.474106726	0.16173707	0.903074508	-4.419124948
hsa-mir-4645	MI0017272	0.129868031	0.863143759	-1.634736081	0.123526225	0.896940866	-4.296380643
hsa-mir-1226	MI0006313	0.129733054	1.081794228	-1.513826944	0.151465211	0.902932883	-4.389472127
hsa-mir-425	MI0001448	0.129264724	1.334462593	-0.974316285	0.345804493	0.930573075	-4.74382278
hsa-mir-554	MI0003559	0.129089857	0.840576292	-1.740032027	0.102931955	0.896940866	-4.212167337
hsa-miR-4725-5p	MIMAT0019843	0.128942195	1.263963173	-0.858893088	0.404313791	0.930588488	-4.803739003
hsa-mir-2053	MI0010487	0.128570603	0.948173023	-1.986623263	0.066111554	0.896940866	-4.005557473
hsa-mir-3148	MI0014175	0.12854546	1.10737407	-0.996791456	0.335138704	0.930208704	-4.73142714
hsa-mir-6750	MI0022595	0.128473309	1.077836028	-1.933305616	0.072877227	0.896940866	-4.05119533
hsa-mir-6797	MI0022642	0.128461917	0.996859755	-1.710636168	0.108355287	0.896940866	-4.23594953
hsa-miR-875-5p	MIMAT0004922	0.127960328	0.822235079	-1.740031626	0.102932027	0.896940866	-4.212167663
hsa-mir-4662a	MI0017290	0.127924401	0.909907434	-1.554282392	0.141573287	0.896940866	-4.358784718
hsa-miR-3176	MIMAT0015053	0.127503348	0.897958749	-1.440944416	0.170752408	0.903074508	-4.443505912
hsa-miR-6791-3p	MIMAT0027483	0.127498818	1.002171382	-1.742244194	0.102533636	0.896940866	-4.210369476
hsa-miR-1247-5p	MIMAT0005899	0.127167786	1.047441699	-1.469675543	0.162918268	0.903074508	-4.42240286
hsa-miR-4655-3p	MIMAT0019722	0.126794974	1.027843561	-1.529192318	0.147641547	0.901741266	-4.377873381
hsa-mir-6793	MI0022638	0.126758111	0.98848305	-1.941786266	0.071760778	0.896940866	-4.043968161
hsa-mir-933	MI0005755	0.126728392	1.055143046	-1.286667577	0.218285402	0.917147059	-4.552090608
hsa-miR-5583-5p	MIMAT0022281	0.126648702	0.944525332	-1.662799948	0.117714978	0.896940866	-4.274205327
hsa-miR-4329	MIMAT0016923	0.126484334	1.17615309	-1.018883701	0.324885007	0.930208704	-4.719018378
hsa-miR-4282	MIMAT0016912	0.125781269	0.910570599	-1.295092163	0.215441494	0.917147059	-4.546378136
hsa-miR-569	MIMAT0003234	0.125440045	0.926693373	-1.305341693	0.212021405	0.91645315	-4.539393083
hsa-mir-4442	MI0016785	0.125398606	1.161839391	-0.827777208	0.421153387	0.937468841	-4.81878486
hsa-miR-8074	MIMAT0031001	0.125208653	0.937172291	-1.40101447	0.182155321	0.903156384	-4.472391241
hsa-miR-4518	MIMAT0019055	0.12484028	0.880086847	-1.45200427	0.167700497	0.903074508	-4.435413517
hsa-miR-18a-5p	MIMAT0000072	0.1247597	7.759897584	-0.567295401	0.579152908	0.967979651	-4.925041705
hsa-miR-590-5p	MIMAT0003258	0.124422234	0.986045046	-1.713034565	0.107903612	0.896940866	-4.234016789
hsa-miR-592	MIMAT0003260	0.124392516	0.824961431	-1.431398771	0.173423314	0.903074508	-4.450458701
hsa-miR-548q	MIMAT0011163	0.124271426	1.058151881	-1.265320396	0.225625107	0.919664114	-4.566447547
hsa-miR-938	MIMAT0004981	0.124258107	0.914676029	-1.830564266	0.087701377	0.896940866	-4.13770657
hsa-miR-376b-3p	MIMAT0002172	0.124190918	1.052758087	-1.037991521	0.316200115	0.930208704	-4.708108898
hsa-miR-4726-3p	MIMAT0019846	0.124077537	1.026168438	-2.15761125	0.04809949	0.896940866	-3.856356737
hsa-miR-1471	MIMAT0007349	0.124034461	1.077734832	-0.854104902	0.406875776	0.931395621	-4.806085542
hsa-mir-483	MI0002467	0.123889334	0.986633001	-1.625658128	0.125458276	0.896940866	-4.303509911
hsa-miR-7114-3p	MIMAT0028126	0.123822596	1.510409968	-1.008607551	0.329626118	0.930208704	-4.72481775
hsa-miR-577	MIMAT0003242	0.123781811	1.000171264	-1.33322697	0.202935892	0.905221726	-4.520196587
hsa-mir-6877	MI0022724	0.123617152	0.94503271	-1.103091498	0.287880423	0.927057986	-4.66973564
hsa-mir-6125	MI0021259	0.123596937	1.050140601	-1.019769785	0.324478502	0.930208704	-4.718516087
hsa-miR-4691-5p	MIMAT0019781	0.123356731	1.019317744	-1.648523189	0.120641037	0.896940866	-4.28551183
hsa-miR-7151-5p	MIMAT0028212	0.123237671	0.857273048	-1.63547909	0.123369233	0.896940866	-4.295796169
hsa-mir-124-1	MI0000443	0.1228786	0.883730961	-1.00966903	0.329134099	0.930208704	-4.724220909
hsa-miR-4506	MIMAT0019042	0.122794543	1.170916659	-1.166102287	0.262313411	0.924045176	-4.63087714
hsa-mir-150	MI0000479	0.122655603	0.955017249	-1.555027319	0.141396402	0.896940866	-4.358215188
hsa-miR-4753-5p	MIMAT0019890	0.122650896	0.929556484	-1.344840328	0.199245626	0.905221726	-4.512119895
hsa-miR-4455	MIMAT0018977	0.122626095	1.278621516	-0.663845501	0.517160456	0.960979619	-4.889878981
hsa-miR-3922-5p	MIMAT0019227	0.122516231	0.856858531	-1.135773217	0.274395668	0.927057986	-4.649787009
hsa-miR-6817-5p	MIMAT0027534	0.122376051	0.974750969	-1.302019546	0.213125169	0.91645315	-4.541661332
hsa-miR-3166	MIMAT0015040	0.122355432	0.876389467	-1.536753499	0.14579007	0.897988521	-4.372140108
hsa-mir-4652	MI0017280	0.122299834	0.926529786	-1.619653181	0.126750555	0.896940866	-4.30821384
hsa-miR-4489	MIMAT0019023	0.12186159	0.996973836	-1.029019382	0.320256959	0.930208704	-4.713251789

hsa-mir-6873	MI0022720	0.121840939	1.013920034	-1.87416974	0.081107974	0.896940866	-4.101233511
hsa-mir-548y	MI0016595	0.121724747	1.009883208	-1.197887753	0.250091397	0.919664114	-4.610658075
hsa-miR-3118	MIMAT0014980	0.121546125	0.81878331	-1.141516956	0.272075773	0.927057986	-4.646234933
hsa-mir-216a	MI0000292	0.121229331	0.986626638	-1.27040855	0.223858207	0.918945188	-4.563040996
hsa-miR-1282	MIMAT0005940	0.120849415	0.822182725	-2.114441566	0.052161093	0.896940866	-3.894389478
hsa-miR-1914-5p	MIMAT0007889	0.120599799	1.104293978	-1.138741231	0.273195029	0.927057986	-4.647953221
hsa-mir-6883	MI0022730	0.120075649	0.885859605	-1.129393002	0.276990112	0.927057986	-4.653716624
hsa-miR-3666	MIMAT0018088	0.120009322	0.823694687	-1.478117065	0.160674222	0.903074508	-4.41615307
hsa-mir-320c-2	MI0008191	0.119636856	0.926247401	-1.139873603	0.272738003	0.927057986	-4.647252622
hsa-miR-7974	MIMAT0031177	0.119636435	1.00162117	-1.4289264	0.174120692	0.903114784	-4.45225469
hsa-mir-555	MI0003561	0.119416222	0.848037114	-1.762728954	0.09890911	0.896940866	-4.193668244
hsa-miR-330-5p	MIMAT0004693	0.11920704	0.859049549	-0.991910271	0.337434998	0.930208704	-4.734138914
hsa-mir-4316	MI0015845	0.119155267	0.976181297	-1.331124326	0.203609878	0.905422616	-4.52165379
hsa-miR-4675	MIMAT0019757	0.119103525	0.795123517	-1.062670147	0.305234226	0.930208704	-4.693779211
hsa-miR-1252-5p	MIMAT0005944	0.119042423	1.041426055	-1.00091367	0.333208143	0.930208704	-4.729128562
hsa-mir-4783	MI0017428	0.119009222	0.814291466	-1.425594877	0.175064069	0.903156384	-4.454671634
hsa-miR-6784-3p	MIMAT0027469	0.11891737	1.211605858	-0.944323541	0.360406883	0.930573075	-4.760000607
hsa-miR-548ag	MIMAT0018969	0.118901021	1.059562076	-1.523917905	0.148944797	0.902931596	-4.381862741
hsa-miR-6846-5p	MIMAT0027592	0.118727323	2.415780645	-0.627425609	0.540092578	0.963391053	-4.903742772
hsa-mir-629	MI0003643	0.118580855	0.880199305	-1.172854499	0.259679573	0.923131598	-4.626616019
hsa-miR-5091	MIMAT0021083	0.118504435	0.915383007	-1.465973368	0.163910642	0.903074508	-4.425136783
hsa-miR-7851-3p	MIMAT0030426	0.118271657	1.565100788	-0.357896355	0.725554499	0.989104742	-4.983220708
hsa-miR-744-3p	MIMAT0004946	0.118267427	0.969704316	-1.673716833	0.115519231	0.896940866	-4.265524748
hsa-miR-3688-5p	MIMAT0019223	0.117859355	0.869504118	-1.122509836	0.279809748	0.927057986	-4.657936952
hsa-mir-937	MI0005759	0.117836568	1.006965648	-1.67068703	0.116125031	0.896940866	-4.267936909
hsa-mir-4454	MI0016800	0.117763887	1.044960845	-1.22786694	0.238971269	0.919664114	-4.591220313
hsa-mir-6769a	MI0022614	0.117682341	0.959969349	-1.725029967	0.105669234	0.896940866	-4.224329831
hsa-miR-6823-3p	MIMAT0027547	0.117572964	0.838351426	-1.304989462	0.212138215	0.91645315	-4.539633765
hsa-mir-1255b-1	MI0006435	0.117425427	1.136609702	-1.409926475	0.179557503	0.903156384	-4.465989844
hsa-miR-95-3p	MIMAT0000094	0.117407693	0.757344124	-2.061094413	0.057617196	0.896940866	-3.94106628
hsa-mir-1294	MI0006356	0.117294525	0.92770289	-1.542379925	0.144425126	0.896940866	-4.367863006
hsa-miR-4454	MIMAT0018976	0.117258566	6.988716117	-0.494604512	0.628256272	0.978652564	-4.948091827
hsa-miR-2116-5p	MIMAT0011160	0.117199236	0.761192396	-1.784795641	0.095131538	0.896940866	-4.175572402
hsa-miR-3920	MIMAT0018195	0.117170099	0.766280826	-1.793847946	0.093619169	0.896940866	-4.168118297
hsa-miR-4788	MIMAT0019958	0.117036361	0.985650095	-1.069052618	0.30244408	0.929580142	-4.690029736
hsa-miR-3614-5p	MIMAT0017992	0.116909054	0.902747297	-1.262252604	0.226695727	0.919664114	-4.568496745
hsa-miR-6766-5p	MIMAT0027432	0.11678664	0.867538694	-1.383299909	0.187410498	0.903156384	-4.485036289
hsa-mir-221	MI0000298	0.11673773	0.936366372	-2.062993456	0.057414289	0.896940866	-3.939411211
hsa-mir-181c	MI0000271	0.116722995	0.93201832	-1.026339981	0.321475744	0.930208704	-4.714780678
hsa-miR-4325	MIMAT0016887	0.116658446	1.001927205	-1.317429815	0.208043691	0.9124223	-4.531105884
hsa-miR-556-3p	MIMAT0004793	0.11665595	0.895353391	-1.650794929	0.120171269	0.896940866	-4.283716217
hsa-mir-200b	MI0000342	0.116606404	0.927901573	-1.498835691	0.15527577	0.903074508	-4.400720307
hsa-miR-6772-3p	MIMAT0027445	0.116604599	0.907276868	-1.168915671	0.261213524	0.924045176	-4.629103944
hsa-mir-1284	MI0006431	0.116503521	0.953932663	-1.868269648	0.08197334	0.896940866	-4.106190293
hsa-miR-320c	MIMAT0005793	0.116424184	11.20084867	-0.431373142	0.672513737	0.984005175	-4.965690778
hsa-miR-4469	MIMAT0018996	0.11634286	0.877345949	-0.84393689	0.412351736	0.93366101	-4.811030973
hsa-mir-4662b	MI0017293	0.116280202	0.977560408	-1.27422892	0.222538757	0.91750564	-4.560476852
hsa-mir-202	MI0003130	0.11626115	0.955701974	-1.722956077	0.106052614	0.896940866	-4.226007044
hsa-mir-3127	MI0014144	0.116239726	0.965646829	-1.151961094	0.267895471	0.927057986	-4.639741066
hsa-mir-3139	MI0014162	0.11620852	0.884104282	-1.931010193	0.0731821	0.896940866	-4.053149335
hsa-miR-1908-3p	MIMAT0026916	0.116161691	0.89442482	-1.140627295	0.272434132	0.927057986	-4.646786017
hsa-mir-8062	MI0025898	0.116159368	0.944611421	-1.43224217	0.173185946	0.903074508	-4.449845582
hsa-miR-9-3p	MIMAT0000442	0.115974791	1.074613226	-0.985892551	0.340281316	0.930208704	-4.737467107
hsa-mir-19a	MI0000073	0.11590833	0.944482889	-1.356943476	0.195457589	0.904897643	-4.503652056
hsa-mir-526a-2	MI0003168	0.115775489	1.023339398	-1.165948769	0.26237353	0.924045176	-4.630973806
hsa-miR-8065	MIMAT0030992	0.115694204	0.957239745	-1.498694928	0.155311928	0.903074508	-4.400825601
hsa-mir-551b	MI0003575	0.115666482	1.135895118	-1.332392039	0.203203307	0.905221726	-4.520775411
hsa-miR-6506-3p	MIMAT0025469	0.11557948	0.911767964	-1.081236237	0.297170089	0.927057986	-4.68282315
hsa-mir-373	MI0000781	0.11544355	0.920601966	-1.777860165	0.096304836	0.896940866	-4.18127139
hsa-mir-525	MI0003152	0.115432229	0.904320242	-1.575086123	0.136703521	0.896940866	-4.342819892
hsa-mir-1286	MI0006348	0.115317792	0.906283549	-1.554898876	0.141426888	0.896940866	-4.358313399
hsa-mir-4475	MI0016827	0.115226958	0.975185397	-1.011983286	0.328063216	0.930208704	-4.722917899
hsa-miR-4802-5p	MIMAT0019981	0.115160551	0.740536004	-1.452076973	0.167680586	0.903074508	-4.435360192
hsa-mir-4514	MI0016880	0.115004075	0.923002258	-1.764889622	0.098533468	0.896940866	-4.191901137
hsa-mir-1250	MI0006385	0.114884341	0.968827082	-1.022081177	0.323419838	0.930208704	-4.717204178
hsa-miR-6785-5p	MIMAT0027470	0.114701004	1.976936625	-0.348196691	0.732673937	0.989764242	-4.985297484

hsa-let-7f-2	MI0000068	0.114689014	0.864262717	-1.227651247	0.239049877	0.919664114	-4.591361418
hsa-miR-548a-5p	MIMAT0004803	0.114608142	1.047720407	-1.221627338	0.241253421	0.919664114	-4.595294916
hsa-miR-4258	MIMAT0016879	0.114288362	1.441585742	-0.685531604	0.503774391	0.960979619	-4.881283739
hsa-mir-4784	MI0017429	0.114226197	1.015900283	-1.360700771	0.194293569	0.903794512	-4.501012901
hsa-mir-4719	MI0017354	0.114202294	0.911728158	-1.484747799	0.158929695	0.903074508	-4.411228386
hsa-miR-627-5p	MIMAT0003296	0.114181802	0.90904438	-1.037770571	0.31629957	0.930208704	-4.708235979
hsa-mir-4707	MI0017340	0.114130779	1.013204626	-0.762384217	0.458000251	0.951465791	-4.848816744
hsa-miR-3179	MIMAT0015056	0.114109471	1.044033244	-1.181713824	0.256254576	0.92177216	-4.620997204
hsa-miR-3165	MIMAT0015039	0.11392404	0.873254303	-1.392484167	0.184670652	0.903156384	-4.478493575
hsa-miR-6510-3p	MIMAT0025477	0.113884783	0.840686872	-1.506541755	0.153307063	0.902932883	-4.394946796
hsa-mir-6853	MI0022699	0.113678596	0.957377788	-1.561112312	0.139958521	0.896940866	-4.353556985
hsa-mir-4482	MI0016843	0.113593541	1.147196175	-1.046091157	0.312569922	0.930208704	-4.703435431
hsa-mir-151b	MI0003772	0.113392022	0.948154588	-1.670170572	0.11622857	0.896940866	-4.268347854
hsa-mir-6076	MI0020353	0.113092876	0.8796912	-1.744985598	0.102041903	0.896940866	-4.208139937
hsa-mir-601	MI0003614	0.113000866	1.032645787	-0.850186671	0.408980229	0.932204908	-4.80799732
hsa-miR-3124-3p	MIMAT0019200	0.112858627	0.90133481	-1.634134254	0.123653513	0.896940866	-4.296853952
hsa-miR-5010-3p	MIMAT0021044	0.112843367	1.037003606	-1.189573501	0.253245167	0.921246697	-4.615985983
hsa-miR-636	MIMAT0003306	0.112806259	0.936788197	-1.583986468	0.13466413	0.896940866	-4.335952494
hsa-mir-639	MI0003654	0.112771402	0.906814401	-1.689814056	0.112346491	0.896940866	-4.252670696
hsa-miR-1265	MIMAT0005918	0.112666166	0.956129728	-1.302781992	0.212871446	0.91645315	-4.541141116
hsa-mir-330	MI0000803	0.11252093	1.032509506	-1.070471414	0.301826397	0.929265009	-4.689193834
hsa-miR-4471	MIMAT0018998	0.112294541	0.928144519	-1.184928906	0.255020243	0.921665152	-4.618950304
hsa-miR-6847-3p	MIMAT0027595	0.112041712	0.904100452	-2.049139023	0.058909781	0.896940866	-3.95147419
hsa-miR-2114-5p	MIMAT0011156	0.112028989	0.893979599	-1.507234305	0.153131166	0.902932883	-4.394427046
hsa-miR-3143	MIMAT0015012	0.112022984	0.978157403	-1.722659028	0.106107627	0.896940866	-4.226247193
hsa-mir-3977	MI0016995	0.112011029	0.84140853	-1.309641791	0.210599507	0.91621875	-4.536451154
hsa-mir-378e	MI0016750	0.111740873	1.060856906	-1.148027573	0.269464117	0.927057986	-4.642192095
hsa-mir-4256	MI0015855	0.11173404	0.847356342	-1.364501409	0.193121847	0.903764656	-4.498338317
hsa-mir-6761	MI0022606	0.111707532	1.024971417	-1.719879972	0.106623509	0.896940866	-4.22849291
hsa-miR-548ab	MIMAT0018928	0.111591421	1.007303624	-1.013903066	0.327176767	0.930208704	-4.721835158
hsa-mir-4494	MI0016856	0.111543314	1.032427299	-0.849601685	0.409295034	0.932273639	-4.808282094
hsa-miR-4646-3p	MIMAT0019708	0.111379058	1.054443239	-1.482692656	0.159468696	0.903074508	-4.412756208
hsa-mir-7702	MI0025238	0.111376162	1.054849358	-1.405244326	0.180918515	0.903156384	-4.469356281
hsa-mir-6870	MI0022717	0.111165524	0.863886587	-1.385282761	0.186816182	0.903156384	-4.483626158
hsa-mir-6869	MI0022716	0.11114695	1.073921998	-1.185093149	0.254957309	0.921665152	-4.618845627
hsa-mir-3650	MI0016050	0.11081448	0.868335395	-1.872420876	0.081363622	0.896940866	-4.102703461
hsa-miR-4664-5p	MIMAT0019737	0.110783689	1.019839578	-1.454923606	0.166902514	0.903074508	-4.433270944
hsa-miR-208a-5p	MIMAT0026474	0.110668761	0.753423177	-1.197548108	0.250219635	0.919664114	-4.610876263
hsa-mir-4732	MI0017369	0.110546314	1.124998011	-1.384739502	0.186978858	0.903156384	-4.484012637
hsa-miR-384	MIMAT0001075	0.110472246	0.869912339	-1.748813011	0.101358839	0.896940866	-4.20502429
hsa-miR-1224-5p	MIMAT0005458	0.110364208	1.917659502	-0.3314097	0.745055364	0.990306303	-4.988759959
hsa-mir-3686	MI0016087	0.110310265	0.993126514	-1.711276597	0.108234517	0.896940866	-4.235433577
hsa-mir-6875	MI0022722	0.110291362	0.968366571	-1.110017613	0.28498207	0.927057986	-4.665545577
hsa-mir-633	MI0003648	0.110277981	0.975031776	-1.806006309	0.091621413	0.896940866	-4.158078927
hsa-mir-3920	MI0016427	0.110192998	0.960092688	-1.241530331	0.234032682	0.919664114	-4.582245165
hsa-mir-298	MI0005523	0.110170419	0.898614894	-1.182881823	0.255805629	0.921727531	-4.62025407
hsa-mir-6786	MI0022631	0.110147148	1.035232769	-2.110282887	0.05256866	0.896940866	-3.898041401
hsa-mir-339	MI0000815	0.110050704	1.243894691	-0.912737416	0.376241726	0.930573075	-4.776580593
hsa-mir-99a	MI0000101	0.109818295	0.939521435	-1.410348484	0.179435247	0.903156384	-4.465686064
hsa-miR-4423-5p	MIMAT0019232	0.109818118	0.740913367	-1.85834197	0.083448113	0.896940866	-4.114515577
hsa-miR-5188	MIMAT0021119	0.109744803	0.993168665	-1.0796573	0.297849716	0.927057986	-4.683760712
hsa-miR-4781-3p	MIMAT0019943	0.10960191	0.950571513	-1.615541786	0.127641926	0.896940866	-4.311428924
hsa-miR-668-5p	MIMAT0026636	0.109590516	1.282719159	-0.973241915	0.346320274	0.930573075	-4.744409505
hsa-miR-196b-3p	MIMAT0009201	0.109255616	1.035168408	-1.562915836	0.139534745	0.896940866	-4.352174316
hsa-miR-3157-5p	MIMAT0015031	0.109168139	0.891670988	-1.855115659	0.083932473	0.896940866	-4.117217009
hsa-mir-4694	MI0017327	0.109053214	0.816621075	-1.480686994	0.159996192	0.903074508	-4.414245985
hsa-miR-491-3p	MIMAT0004765	0.108566867	0.915928421	-0.954292231	0.355506629	0.930573075	-4.754670137
hsa-mir-8068	MI0025904	0.108526847	0.95141751	-1.183843482	0.255436447	0.921727531	-4.619641809
hsa-miR-1277-3p	MIMAT0005933	0.108355616	0.832780201	-1.217672707	0.242708598	0.919664114	-4.597869528
hsa-miR-4500	MIMAT0019036	0.107902333	0.930802762	-1.418304236	0.177143243	0.903156384	-4.459948152
hsa-miR-4475	MIMAT0019002	0.107718469	0.876234363	-0.944290482	0.360423211	0.930573075	-4.760018207
hsa-miR-29a-5p	MIMAT0004503	0.107576715	0.946872503	-0.864552936	0.401299204	0.930573075	-4.800950725
hsa-miR-6888-3p	MIMAT0027677	0.107494222	0.887802927	-0.916212037	0.374476886	0.930573075	-4.774779938
hsa-mir-4438	MI0016781	0.107427485	0.993698444	-1.493696415	0.156600482	0.903074508	-4.404560705
hsa-mir-4467	MI0016818	0.107425246	0.890469683	-1.138988353	0.273095241	0.927057986	-4.647800372
hsa-miR-383-5p	MIMAT0000738	0.107282515	0.997863004	-0.886798342	0.389595746	0.930573075	-4.789839687

hsa-miR-634	MIMAT0003304	0.107085623	1.066721916	-1.283648888	0.219311656	0.917147059	-4.554131115
hsa-miR-16-1-3p	MIMAT0004489	0.106997337	0.942856854	-0.737090931	0.472774535	0.952660318	-4.859844053
hsa-miR-1269a	MIMAT0005923	0.106991972	0.883575286	-1.440913568	0.170760984	0.903074508	-4.443528428
hsa-miR-559	MIMAT0003223	0.10657791	1.20361506	-1.009142689	0.329378004	0.930208704	-4.72451692
hsa-mir-5100	MI0019116	0.106521113	0.973939657	-1.174641268	0.258986005	0.923131598	-4.625485353
hsa-mir-643	MI0003658	0.106504886	0.906918547	-1.518065462	0.150402217	0.902932883	-4.386279644
hsa-mir-3130-1	MI0014147	0.106417892	0.833738126	-1.300197796	0.213732378	0.916792111	-4.54290345
hsa-mir-433	MI0001723	0.106374485	1.090452576	-1.398484823	0.182898293	0.903156384	-4.474203426
hsa-mir-6848	MI0022694	0.106324589	1.146722658	-1.623440015	0.125934293	0.896940866	-4.305248569
hsa-miR-7114-5p	MIMAT0028125	0.106232693	2.284282535	-0.262631819	0.796512832	0.994214304	-5.001191553
hsa-mir-5708	MI0019316	0.106082175	0.962964656	-1.485984532	0.158606073	0.903074508	-4.410308354
hsa-mir-498	MI0003142	0.10608079	1.018906577	-1.529541337	0.147555649	0.901517345	-4.377609108
hsa-miR-6827-5p	MIMAT0027554	0.106001001	1.371745426	-0.533721035	0.601585553	0.973554267	-4.936058852
hsa-mir-4734	MI0017371	0.105661729	1.529229706	-0.575074501	0.574017833	0.967644566	-4.922399017
hsa-miR-6852-5p	MIMAT0027604	0.105514525	1.534055035	-0.292339264	0.774150323	0.994214304	-4.996169145
hsa-mir-5093	MI0017982	0.105426868	0.934547192	-1.097243906	0.290344515	0.927057986	-4.673257359
hsa-miR-302a-3p	MIMAT0000684	0.105416247	0.839291673	-1.244401579	0.233005109	0.919664114	-4.580349977
hsa-mir-645	MI0003660	0.105397269	0.822680119	-1.268986055	0.224351075	0.919588975	-4.563994344
hsa-mir-6730	MI0022575	0.105250108	0.91980419	-1.040330201	0.315148808	0.930208704	-4.706762471
hsa-miR-6863	MIMAT0027627	0.105225349	0.956159704	-1.142046969	0.271862451	0.927057986	-4.645906471
hsa-mir-548f-5	MI0006378	0.104994989	1.009875258	-1.259291803	0.227732803	0.919664114	-4.570471109
hsa-miR-4802-3p	MIMAT0019982	0.104912877	1.069141535	-1.105952821	0.286680398	0.927057986	-4.668007104
hsa-miR-5589-5p	MIMAT0022297	0.104912466	0.841913084	-1.227243617	0.239198491	0.919664114	-4.591628037
hsa-miR-708-3p	MIMAT0004927	0.104831197	0.845415336	-1.204227506	0.247707036	0.919664114	-4.606577009
hsa-miR-7158-3p	MIMAT0028227	0.104605512	0.938268495	-1.014867294	0.32673219	0.930208704	-4.721290714
hsa-mir-5007	MI0017874	0.104473871	0.984452522	-0.984047145	0.341157573	0.930208704	-4.738484413
hsa-miR-3605-3p	MIMAT0017982	0.104412765	0.925596227	-1.231167083	0.237771056	0.919664114	-4.589059133
hsa-miR-6877-3p	MIMAT0027655	0.104348407	1.436377388	-0.561183037	0.583204422	0.968332399	-4.927094482
hsa-mir-4276	MI0015882	0.104298383	1.048521905	-1.005125089	0.331244013	0.930208704	-4.726772267
hsa-miR-1263	MIMAT0005915	0.104293548	1.014574315	-1.075803987	0.299513128	0.927057986	-4.68604426
hsa-mir-5197	MI0018176	0.104237936	0.942463193	-1.682422746	0.113793767	0.896940866	-4.258580824
hsa-miR-579-5p	MIMAT0026616	0.104217145	0.796471586	-1.465195624	0.164119757	0.903074508	-4.425710571
hsa-mir-7845	MI0025515	0.104023899	1.196186613	-0.846448583	0.410994594	0.93308407	-4.809814121
hsa-mir-1908	MI0008329	0.103987158	1.013241706	-0.808609776	0.431750082	0.94301493	-4.827812394
hsa-miR-185-5p	MIMAT0000455	0.103946818	10.56954686	-0.340650611	0.738230359	0.989764242	-4.986874622
hsa-mir-3610	MI0016000	0.103927681	0.871814532	-1.280547716	0.220369935	0.917147059	-4.556223857
hsa-mir-153-2	MI0000464	0.103906403	0.916536916	-1.358070005	0.195107996	0.904897643	-4.502861288
hsa-mir-6078	MI0020355	0.103860524	0.981680731	-1.175037163	0.258832522	0.923131598	-4.625234656
hsa-miR-3663-5p	MIMAT0018084	0.103848106	1.037953933	-1.150404571	0.268515365	0.927057986	-4.640711717
hsa-miR-744-5p	MIMAT0004945	0.103695991	7.322405883	-0.755203827	0.462165125	0.951465791	-4.851980989
hsa-miR-6884-3p	MIMAT0027669	0.103685315	1.057835821	-1.402587509	0.181694558	0.903156384	-4.471263269
hsa-mir-5698	MI0019305	0.103669296	0.979337895	-1.259424118	0.227686378	0.919664114	-4.570382947
hsa-mir-3618	MI0016008	0.103386863	0.951562552	-1.27270541	0.223064196	0.918415347	-4.561500051
hsa-mir-6864	MI0022711	0.103183846	0.889368011	-1.326181925	0.205201217	0.908380751	-4.525072834
hsa-miR-4446-3p	MIMAT0018965	0.103129047	2.175750104	-0.393246258	0.699833675	0.98572659	-4.975182167
hsa-mir-6854	MI0022700	0.103116622	0.937858417	-1.207498407	0.246483756	0.919664114	-4.604465229
hsa-miR-7845-5p	MIMAT0030420	0.102938901	2.230734603	-0.31592933	0.756538094	0.99324537	-4.991804503
hsa-mir-132	MI0000449	0.102818002	1.019682722	-0.784947045	0.445065407	0.949053423	-4.838700878
hsa-miR-3121-5p	MIMAT0019199	0.102692229	0.863899479	-1.429227422	0.17403566	0.903114784	-4.452036127
hsa-mir-4739	MI0017377	0.102671373	0.83797464	-0.950143867	0.357540148	0.930573075	-4.756894013
hsa-mir-6072	MI0020349	0.10263603	0.913923263	-1.538781532	0.145296826	0.896940866	-4.3705995
hsa-miR-4257	MIMAT0016878	0.102589076	0.972484746	-1.354417821	0.196243215	0.905071197	-4.505423334
hsa-miR-4725-3p	MIMAT0019844	0.102562154	1.493978328	-0.677634718	0.508625347	0.960979619	-4.884442849
hsa-miR-5739	MIMAT0023116	0.102301772	3.029648214	-0.181181071	0.858722591	0.996937676	-5.01223199
hsa-mir-6808	MI0022653	0.10224052	0.920413695	-0.562878253	0.582079303	0.968332399	-4.926527253
hsa-mir-3606	MI0015996	0.102087748	0.854269798	-1.405938572	0.180716177	0.903156384	-4.468857583
hsa-miR-5191	MIMAT0021122	0.102030219	0.99976972	-0.905649524	0.379859398	0.930573075	-4.780235829
hsa-miR-4638-5p	MIMAT0019695	0.101968317	1.15862774	-0.554710596	0.587510482	0.968332399	-4.929245405
hsa-mir-520e	MI0003143	0.101745037	0.940742612	-1.544918823	0.143812753	0.896940866	-4.365929967
hsa-miR-4261	MIMAT0016890	0.101700806	1.030020369	-1.191799507	0.252397809	0.920672039	-4.614562222
hsa-mir-532	MI0003205	0.101554559	0.95144876	-1.759951151	0.099393897	0.896940866	-4.195938541
hsa-mir-6755	MI0022600	0.101532948	0.922642959	-1.389169765	0.185655603	0.903156384	-4.480857993
hsa-miR-369-5p	MIMAT0001621	0.101298164	0.827197796	-1.207577417	0.246454265	0.919664114	-4.604414166
hsa-miR-6828-5p	MIMAT0027556	0.101166926	0.94116889	-1.313067455	0.20947222	0.914707942	-4.53410267
hsa-miR-1253	MIMAT0005904	0.101145697	0.896097071	-1.11719491	0.282001681	0.927057986	-4.661182143
hsa-miR-6835-5p	MIMAT0027570	0.101135256	1.022340116	-0.689691584	0.501229862	0.960979619	-4.87960616

hsa-miR-4677-3p	MIMAT0019761	0.100919022	0.819426965	-1.370908874	0.191159447	0.903697113	-4.493817953
hsa-miR-4292	MIMAT0016919	0.100869515	0.963432292	-1.362801803	0.193645118	0.903764656	-4.499534982
hsa-miR-3144-3p	MIMAT0015015	0.100723454	0.899523569	-1.270950756	0.223670569	0.918871758	-4.562677413
hsa-mir-4798	MI0017445	0.100599899	1.09872429	-1.824295569	0.088687556	0.896940866	-4.14291868
hsa-miR-4777-3p	MIMAT0019935	0.100560536	0.886895232	-1.405635874	0.180804376	0.903156384	-4.46907504
hsa-miR-363-5p	MIMAT0003385	0.10001899	0.834371283	-1.450340018	0.168156826	0.903074508	-4.436633738
hsa-miR-3678-5p	MIMAT0018102	0.099993064	0.831096225	-1.988968555	0.065827497	0.896940866	-4.003539314
hsa-miR-4664-3p	MIMAT0019738	0.099848437	0.936484442	-1.179460067	0.257122569	0.922824015	-4.622429595
hsa-mir-615	MI0003628	0.099595555	0.868358919	-1.356265153	0.195668336	0.904897643	-4.504127992
hsa-mir-130b	MI0000748	0.09940972	0.938390646	-0.965495429	0.35005522	0.930573075	-4.748624134
hsa-mir-6802	MI0022647	0.099391662	0.997560426	-1.0241249	0.322485848	0.930208704	-4.716042198
hsa-mir-6752	MI0022597	0.099210235	0.846148281	-1.107644044	0.285972865	0.927057986	-4.666983796
hsa-mir-659	MI0003683	0.099138056	0.972627068	-1.323249579	0.206150078	0.909829393	-4.527097237
hsa-miR-590-3p	MIMAT0004801	0.099011705	0.99891921	-0.965501525	0.35005227	0.930573075	-4.748620828
hsa-miR-4534	MIMAT0019073	0.098973592	2.218655051	-0.435190611	0.669803668	0.984005175	-4.964693791
hsa-mir-3671	MI0016072	0.098933966	0.995212002	-0.931511246	0.366773495	0.930573075	-4.766782972
hsa-mir-4761	MI0017402	0.098832928	0.95005671	-1.113187896	0.28366271	0.927057986	-4.663620886
hsa-mir-3117	MI0014130	0.098720546	0.975268578	-0.890643708	0.387596149	0.930573075	-4.787894588
hsa-mir-942	MI0005767	0.098531815	0.956991669	-1.249119897	0.231324187	0.919664114	-4.577228764
hsa-miR-4782-5p	MIMAT0019944	0.098488105	0.906189172	-1.221501048	0.241299787	0.919664114	-4.59537723
hsa-mir-7848	MI0025518	0.098312762	0.972705997	-1.03616838	0.317021435	0.930208704	-4.709156835
hsa-miR-8083	MIMAT0031010	0.098305927	0.871793035	-1.58272571	0.134951427	0.896940866	-4.336926622
hsa-miR-6842-5p	MIMAT0027586	0.09821647	0.900270861	-1.201599705	0.248693202	0.919664114	-4.608270525
hsa-miR-891a-3p	MIMAT0026717	0.09818408	0.809894664	-1.390074896	0.185386197	0.903156384	-4.480212662
hsa-miR-4328	MIMAT0016926	0.097880183	0.826962109	-1.269126605	0.224302339	0.919588975	-4.563900183
hsa-mir-548ap	MI0017875	0.097870753	0.915693959	-1.414508618	0.178233715	0.903156384	-4.462688268
hsa-mir-582	MI0003589	0.097854391	0.847112932	-1.318569182	0.207671874	0.911884967	-4.530322049
hsa-mir-382	MI0000790	0.097583773	0.994230115	-0.632229913	0.537035545	0.96159396	-4.901955219
hsa-miR-3914	MIMAT0018188	0.097569969	1.132751844	-1.628555825	0.124838753	0.896940866	-4.301236599
hsa-miR-1250-3p	MIMAT0026740	0.097410752	0.865671518	-1.109594546	0.285158482	0.927057986	-4.665802101
hsa-mir-3621	MI0016012	0.097018665	0.90993201	-0.999388008	0.333921731	0.930208704	-4.729980184
hsa-mir-4287	MI0015895	0.09690512	0.962952957	-1.103477631	0.287718263	0.927057986	-4.669502578
hsa-miR-6751-5p	MIMAT0027402	0.096598305	1.37669334	-0.578790858	0.571573064	0.967644566	-4.921124612
hsa-miR-1200	MIMAT0005863	0.096578868	0.816024505	-1.26059347	0.22727641	0.919664114	-4.56960352
hsa-mir-496	MI0003136	0.096465904	0.971728879	-0.732762494	0.475331666	0.953466088	-4.861697767
hsa-miR-4704-5p	MIMAT0019803	0.096264178	0.849266429	-1.064010915	0.304646538	0.930208704	-4.692993032
hsa-mir-4695	MI0017328	0.096262612	0.992891198	-1.279910573	0.220587862	0.917147059	-4.556653374
hsa-mir-518e	MI0003169	0.096124971	0.83955014	-1.612489174	0.128307227	0.896940866	-4.313813115
hsa-miR-190b-5p	MIMAT0004929	0.096107649	0.885675495	-1.098974516	0.289613631	0.927057986	-4.672216615
hsa-miR-1225-5p	MIMAT0005572	0.096093722	4.307541184	-0.185312763	0.855539598	0.996937676	-5.011768735
hsa-mir-510	MI0003197	0.096068592	0.942405128	-1.145083329	0.270642801	0.927057986	-4.644022522
hsa-miR-4671-3p	MIMAT0019753	0.096034101	0.848603689	-1.46312369	0.164677928	0.903074508	-4.427238232
hsa-miR-4699-5p	MIMAT0019794	0.09584916	0.892262037	-0.736939402	0.472863913	0.952660318	-4.859909113
hsa-miR-5089-5p	MIMAT0021081	0.095760611	0.906867027	-0.936944688	0.364064124	0.930573075	-4.763916165
hsa-mir-199b	MI0000282	0.095758933	0.949472767	-1.102681981	0.288052479	0.927057986	-4.669982746
hsa-mir-4285	MI0015891	0.095721481	1.219838603	-0.923670126	0.37070789	0.930573075	-4.770895468
hsa-miR-769-3p	MIMAT0003887	0.095717064	0.865824942	-1.013671252	0.327283715	0.930208704	-4.721965987
hsa-mir-4469	MI0016820	0.095534425	1.368016693	-0.622525114	0.543220713	0.964741076	-4.905553122
hsa-miR-193a-3p	MIMAT0000459	0.095533467	0.922948123	-1.010788482	0.328615779	0.930208704	-4.723590919
hsa-mir-6512	MI0022224	0.09552842	1.048384344	-0.791677276	0.441252074	0.947408294	-4.835632923
hsa-miR-614	MIMAT0003282	0.095482834	0.929087263	-0.880763362	0.392747907	0.930573075	-4.792877877
hsa-miR-3171	MIMAT0015046	0.095351659	1.006360452	-1.370966145	0.19114198	0.903697113	-4.493777485
hsa-mir-147a	MI0000262	0.095274681	0.978489116	-1.062607262	0.305261811	0.930208704	-4.693816066
hsa-miR-5090	MIMAT0021082	0.095117478	1.005560089	-0.99437032	0.336276303	0.930208704	-4.732773577
hsa-miR-4273	MIMAT0016903	0.095052424	0.778297906	-1.378640075	0.188813252	0.903156384	-4.488344923
hsa-miR-3677-5p	MIMAT0019221	0.095032588	0.872277682	-1.374801211	0.189975288	0.903156384	-4.491065072
hsa-miR-1976	MIMAT0009451	0.094941058	1.00162974	-1.176711609	0.258184137	0.923131598	-4.624173629
hsa-mir-559	MI0003565	0.09489958	0.964890541	-0.972345943	0.346750825	0.930573075	-4.744898398
hsa-mir-6798	MI0022643	0.094893474	0.881244766	-1.338170169	0.201358463	0.905221726	-4.516764597
hsa-mir-611	MI0003624	0.094860046	1.121438545	-0.689689528	0.501231118	0.960979619	-4.879606992
hsa-miR-4666a-5p	MIMAT0019741	0.094784617	0.98428312	-0.878958002	0.393694186	0.930573075	-4.793783306
hsa-mir-4804	MI0017452	0.094777801	0.96313568	-1.323374468	0.206109594	0.909829393	-4.52701108
hsa-mir-3907	MI0016410	0.094741394	1.10768231	-1.070792482	0.301686747	0.929265009	-4.689004551
hsa-miR-7843-5p	MIMAT0030411	0.094737106	0.956407981	-0.836218664	0.416540478	0.934671969	-4.814750643
hsa-miR-3690	MIMAT0018119	0.09470629	1.093229784	-0.909410273	0.377936969	0.930573075	-4.778299405
hsa-miR-3140-5p	MIMAT0019204	0.094450705	0.877065025	-1.140353724	0.2725444	0.927057986	-4.64695541

hsa-mir-4429	MI0016768	0.094366645	0.919177005	-1.061637258	0.305687532	0.930208704	-4.694384326
hsa-miR-222-5p	MIMAT0004569	0.094363078	0.878726645	-1.082984385	0.296418965	0.927057986	-4.681783857
hsa-miR-4658	MIMAT0019725	0.094330224	0.89087446	-1.402886037	0.181607224	0.903156384	-4.471049112
hsa-miR-1205	MIMAT0005869	0.094106421	0.931346562	-1.084797075	0.295641594	0.927057986	-4.680704802
hsa-miR-6859-5p	MIMAT0027618	0.094082055	1.130016869	-0.862765722	0.402249507	0.930573075	-4.801832883
hsa-miR-433-5p	MIMAT0026554	0.093961694	0.92666498	-0.906059799	0.379649351	0.930573075	-4.780024907
hsa-miR-6894-3p	MIMAT0027689	0.093848471	0.963798893	-0.813485048	0.4290387	0.94301493	-4.825533761
hsa-miR-1290	MIMAT0005880	0.093499961	1.38814184	-0.37346327	0.714183444	0.9869374	-4.979771497
hsa-mir-4773-1	MI0017415	0.093417091	0.869928691	-1.194630035	0.251323488	0.920354866	-4.612748953
hsa-mir-4260	MI0015859	0.093406955	1.160879264	-0.998474473	0.334349534	0.930208704	-4.730489611
hsa-mir-4531	MI0016898	0.093278275	1.156884462	-0.928741742	0.368159836	0.930573075	-4.768238849
hsa-miR-380-3p	MIMAT0000735	0.093258998	0.946852893	-0.814596888	0.428421885	0.94301493	-4.825012423
hsa-miR-6761-3p	MIMAT0027423	0.092977565	0.976115463	-0.919461795	0.372831388	0.930573075	-4.773090594
hsa-miR-512-3p	MIMAT0002823	0.092916199	1.117826951	-1.000083173	0.333596451	0.930208704	-4.729592275
hsa-mir-7156	MI0023616	0.092830894	0.939149443	-1.2454835	0.232618826	0.919664114	-4.579635027
hsa-miR-6814-5p	MIMAT0027528	0.092830715	0.947401475	-0.852059379	0.407973519	0.93183346	-4.807084538
hsa-mir-3654	MI0016054	0.092754898	0.912581251	-1.22405972	0.240361761	0.919664114	-4.593708316
hsa-mir-6787	MI0022632	0.092735978	1.334436436	-1.018158359	0.325218041	0.930208704	-4.719429287
hsa-miR-3186-3p	MIMAT0015068	0.09272251	0.880740112	-1.039391004	0.3155707	0.930208704	-4.707303478
hsa-mir-4747	MI0017386	0.092712949	0.85937689	-1.139424215	0.272919307	0.927057986	-4.647530722
hsa-mir-4517	MI0016883	0.092684366	0.983353227	-1.20185149	0.24859858	0.919664114	-4.608108377
hsa-mir-1285-2	MI0006347	0.09262245	0.952257951	-0.833118074	0.418230982	0.935967201	-4.816236567
hsa-mir-4540	MI0016911	0.092605666	0.882175752	-1.037311513	0.316506276	0.930208704	-4.708499938
hsa-miR-4448	MIMAT0018967	0.092604493	0.69092336	-1.242940289	0.233527638	0.919664114	-4.581314906
hsa-mir-5011	MI0017879	0.092505879	0.998417157	-0.884438611	0.390826239	0.930573075	-4.791029755
hsa-mir-1231	MI0006321	0.092497273	1.002342312	-1.47340216	0.161924404	0.903074508	-4.419646552
hsa-mir-4292	MI0015897	0.092471347	1.028496503	-0.813487103	0.429037559	0.94301493	-4.825532798
hsa-mir-4533	MI0016900	0.092416138	1.070172448	-1.054957586	0.308630962	0.930208704	-4.698286312
hsa-miR-3915	MIMAT0018189	0.092376863	0.907765075	-0.804870561	0.433837069	0.943879286	-4.829551913
hsa-miR-4303	MIMAT0016856	0.092352534	0.852277674	-0.844502751	0.412045729	0.933586591	-4.810757101
hsa-mir-122	MI0000442	0.092347127	0.922213141	-0.847421808	0.41046952	0.932670697	-4.809341777
hsa-miR-5192	MIMAT0021123	0.09232506	0.981974725	-0.745256448	0.467973415	0.951465791	-4.856320422
hsa-mir-127	MI0000472	0.092205068	1.004485717	-0.783280854	0.446012667	0.949212502	-4.839456833
hsa-mir-4307	MI0015838	0.092156632	0.833604095	-1.143477601	0.271287275	0.927057986	-4.645019291
hsa-mir-6803	MI0022648	0.092045401	0.941788596	-1.334411704	0.202556927	0.905221726	-4.519374834
hsa-mir-6833	MI0022678	0.092040081	0.938731384	-1.730511264	0.104661801	0.896940866	-4.219892099
hsa-mir-6881	MI0022728	0.092013912	0.887739546	-1.395458452	0.183790416	0.903156384	-4.476368633
hsa-mir-3612	MI0016002	0.091763795	0.965407799	-0.866885774	0.400061023	0.930573075	-4.799796889
hsa-mir-181d	MI0003139	0.091739593	1.088721662	-0.99122839	0.337756668	0.930208704	-4.734516871
hsa-miR-6823-5p	MIMAT0027546	0.091715479	0.978462322	-1.158779615	0.265192769	0.926762219	-4.63547731
hsa-miR-6867-5p	MIMAT0027634	0.091697621	1.121901962	-1.100865392	0.288816629	0.927057986	-4.671078031
hsa-miR-6512-3p	MIMAT0025481	0.091672291	0.901265691	-1.363024985	0.19357634	0.903764656	-4.499377901
hsa-mir-657	MI0003681	0.09145014	0.876387189	-1.241826806	0.233926414	0.919664114	-4.58204962
hsa-mir-6882	MI0022729	0.091334695	1.049516638	-0.646655669	0.527914371	0.960979619	-4.896512226
hsa-mir-5683	MI0019284	0.09120037	0.873834325	-1.087946178	0.294294692	0.927057986	-4.678826846
hsa-miR-374c-3p	MIMAT0022735	0.090964171	0.815543239	-1.6755135	0.115161291	0.896940866	-4.264093256
hsa-mir-190b	MI0005545	0.09092337	0.983885262	-1.160741625	0.264418933	0.926587765	-4.634246901
hsa-miR-2681-5p	MIMAT0013515	0.090725088	0.927714639	-1.289690509	0.217261531	0.917147059	-4.550043854
hsa-mir-548z	MI0016688	0.090489735	1.010804901	-0.811065553	0.430382931	0.94301493	-4.826666098
hsa-let-7e	MI0000066	0.090371004	1.03833044	-1.074524095	0.300067149	0.927498149	-4.686801326
hsa-mir-4483	MI0016844	0.090291568	1.125276056	-1.102337016	0.288197473	0.927057986	-4.670190846
hsa-mir-548ao	MI0017871	0.090274095	0.963832708	-0.92364242	0.370721844	0.930573075	-4.770909948
hsa-mir-423	MI0001445	0.090270576	2.585598109	-0.396584238	0.6974239	0.985651988	-4.974385148
hsa-mir-7973-1	MI0025748	0.090225017	0.970415992	-1.051387756	0.310212496	0.930208704	-4.700363629
hsa-mir-33b	MI0003646	0.090208087	0.975910173	-0.777031561	0.449576801	0.950247229	-4.842279494
hsa-miR-4436a	MIMAT0018952	0.090187786	1.008202715	-0.783115819	0.446106562	0.949212502	-4.839531633
hsa-mir-3160-2	MI0014190	0.090176818	0.887843209	-1.73323577	0.104164194	0.896940866	-4.217683693
hsa-miR-4796-5p	MIMAT0019970	0.090151663	0.900397599	-0.773778733	0.451439022	0.950325019	-4.843740797
hsa-miR-4794	MIMAT0019967	0.090114111	0.840168696	-1.169758726	0.26088462	0.923951887	-4.628571965
hsa-mir-4288	MI0015896	0.090110668	0.876556795	-1.597833948	0.131542866	0.896940866	-4.325224264
hsa-mir-6760	MI0022605	0.090053083	1.053144495	-0.902850496	0.38129452	0.930573075	-4.781672649
hsa-mir-9-1	MI0000466	0.089915721	0.943535408	-1.289406942	0.217357413	0.917147059	-4.550235994
hsa-miR-616-3p	MIMAT0004805	0.089853963	0.987029397	-1.197661932	0.250176653	0.919664114	-4.610803148
hsa-miR-4653-5p	MIMAT0019718	0.089698856	0.833334595	-1.377695589	0.189098613	0.903156384	-4.489014636
hsa-miR-6872-3p	MIMAT0027645	0.089540736	0.905113265	-1.070171536	0.301956874	0.929265009	-4.689370584
hsa-miR-6890-3p	MIMAT0027681	0.089538	1.031699861	-0.893291799	0.386223166	0.930573075	-4.78655094

hsa-mir-1-1	MI0000651	0.089423744	0.955845047	-1.252091197	0.230270542	0.919664114	-4.575258862
hsa-miR-651-3p	MIMAT0026624	0.089330839	1.129128119	-1.008047348	0.329885997	0.930208704	-4.725132532
hsa-mir-621	MI0003635	0.089303749	0.867984504	-1.107647213	0.285971541	0.927057986	-4.666981878
hsa-miR-4251	MIMAT0016883	0.089234237	0.847214982	-1.153128865	0.267431115	0.927057986	-4.639012188
hsa-miR-548l	MIMAT0005889	0.089175513	1.028821159	-0.84938277	0.409412883	0.932273639	-4.808388619
hsa-mir-921	MI0005713	0.089166006	1.040253216	-0.758221551	0.460411899	0.951465791	-4.850654392
hsa-miR-200a-5p	MIMAT0001620	0.08911373	0.86420633	-1.24271234	0.233609231	0.919664114	-4.581465353
hsa-mir-5694	MI0019301	0.08898441	0.883208972	-0.781776087	0.446869246	0.950068641	-4.84013833
hsa-mir-199a-1	MI0000242	0.088748023	1.026815776	-0.686709208	0.503053323	0.960979619	-4.880809789
hsa-mir-4265	MI0015869	0.088669896	0.945414688	-1.00796952	0.329922113	0.930208704	-4.725176253
hsa-miR-6761-5p	MIMAT0027422	0.088586746	0.868616058	-0.950897554	0.357170091	0.930573075	-4.756490572
hsa-mir-6764	MI0022609	0.088568512	0.972313533	-1.347216016	0.198497441	0.905221726	-4.51046182
hsa-miR-6805-3p	MIMAT0027511	0.088342194	1.660793362	-0.41977875	0.680773504	0.985064407	-4.968666866
hsa-miR-450b-5p	MIMAT0004909	0.088338912	0.781126441	-1.027745395	0.320836043	0.930208704	-4.713979137
hsa-mir-1914	MI0008335	0.0882824	1.187366191	-0.619520366	0.545143658	0.965790957	-4.906656642
hsa-miR-4432	MIMAT0018948	0.088024717	0.921007608	-1.049024325	0.311262812	0.930208704	-4.701735846
hsa-mir-3682	MI0016083	0.088012941	1.081911384	-0.741678472	0.470073492	0.952023826	-4.857868691
hsa-miR-191-5p	MIMAT0000440	0.087575873	11.05392837	-0.716311889	0.485126488	0.957574873	-4.868653344
hsa-miR-7856-5p	MIMAT0030431	0.087571036	0.865348825	-1.079377413	0.297970308	0.927057986	-4.683926795
hsa-mir-4653	MI0017281	0.087565983	0.906343179	-0.89309809	0.38632349	0.930573075	-4.786649344
hsa-mir-4479	MI0016838	0.087544188	1.925903767	-0.622581423	0.543184713	0.964741076	-4.905532395
hsa-miR-22-3p	MIMAT0000077	0.087513656	8.039590878	-0.535287373	0.600529413	0.97307982	-4.935558966
hsa-miR-4478	MIMAT0019006	0.08726049	1.000962567	-0.578884824	0.57151132	0.967644566	-4.92109229
hsa-mir-602	MI0003615	0.0872023	0.852321602	-1.034627062	0.317717001	0.930208704	-4.710041628
hsa-mir-3122	MI0014138	0.087178782	0.904807079	-1.403850917	0.181325183	0.903156384	-4.470356724
hsa-mir-940	MI0005762	0.086712048	1.131886539	-0.565142816	0.580578058	0.968332399	-4.925767011
hsa-miR-873-3p	MIMAT0022717	0.086697817	0.931932978	-1.290057472	0.2171375	0.917147059	-4.549795163
hsa-mir-147b	MI0005544	0.08662742	1.005738122	-1.071252559	0.301486717	0.929265009	-4.688733238
hsa-miR-323b-3p	MIMAT0015050	0.086600408	1.186383064	-0.387032965	0.704328108	0.986573137	-4.976648326
hsa-mir-5693	MI0019300	0.086396919	0.804626305	-1.337772758	0.201484915	0.905221726	-4.517040833
hsa-miR-6753-5p	MIMAT0027406	0.086338786	1.455817899	-0.433275398	0.67116271	0.984005175	-4.965195035
hsa-miR-219a-2-3p	MIMAT0004675	0.086264517	1.018010665	-1.306321431	0.211696766	0.91645315	-4.538723382
hsa-miR-6801-5p	MIMAT0027502	0.08607217	1.017436336	-0.730741838	0.476528287	0.954185228	-4.862559784
hsa-miR-3688-3p	MIMAT0018116	0.085867654	0.98009258	-0.861734133	0.402798705	0.930573075	-4.802341355
hsa-mir-30d	MI0000255	0.085857505	0.972270156	-0.905437519	0.379967969	0.930573075	-4.780344789
hsa-mir-7161	MI0023619	0.085786093	0.963474428	-0.997463649	0.334823352	0.930208704	-4.731052848
hsa-mir-518c	MI0003159	0.085736649	0.88218058	-1.478090928	0.16068113	0.903074508	-4.416172455
hsa-mir-4496	MI0016858	0.085718462	0.953061067	-0.976456256	0.344778753	0.930573075	-4.742652531
hsa-mir-769	MI0003834	0.085634657	0.868451122	-0.92945135	0.367804281	0.930573075	-4.767866168
hsa-miR-3935	MIMAT0018350	0.085491854	1.149752217	-0.909182025	0.378053457	0.930573075	-4.778417125
hsa-mir-6504	MI0022216	0.085470216	0.838728517	-1.004576015	0.331499621	0.930208704	-4.72707993
hsa-miR-5008-3p	MIMAT0021040	0.08538842	0.944046305	-0.73276607	0.47532955	0.953466088	-4.86169624
hsa-miR-6803-3p	MIMAT0027507	0.085168286	1.018162807	-0.917424732	0.373862264	0.930573075	-4.774150124
hsa-miR-6797-5p	MIMAT0027494	0.085161661	2.102167868	-0.222510332	0.827006722	0.996080495	-5.007131513
hsa-miR-4254	MIMAT0016884	0.08507284	0.877262462	-0.763797044	0.457183511	0.951465791	-4.848191003
hsa-miR-4521	MIMAT0019058	0.085072052	0.823276729	-1.40471803	0.181072027	0.903156384	-4.469734229
hsa-miR-432-3p	MIMAT0002815	0.085054061	0.868462874	-0.985114454	0.340650587	0.930208704	-4.737896235
hsa-mir-4538	MI0016909	0.085029146	1.176084732	-0.631635587	0.537413199	0.96159396	-4.902177035
hsa-mir-488	MI0003123	0.085005997	0.955377868	-1.316474704	0.208355789	0.913123326	-4.531762599
hsa-miR-181d-3p	MIMAT0026608	0.084937634	1.010463935	-0.890844945	0.387491697	0.930573075	-4.787792599
hsa-miR-216b-5p	MIMAT0004959	0.084873243	0.940889888	-0.789377168	0.442552977	0.947408294	-4.83668402
hsa-miR-4718	MIMAT0019831	0.084867607	0.959061874	-0.645387402	0.528712764	0.960979619	-4.896995291
hsa-miR-4673	MIMAT0019755	0.084839722	1.127400141	-0.570821778	0.576822151	0.967979651	-4.923847917
hsa-miR-2278	MIMAT0011778	0.084743366	1.383941975	-0.466798018	0.647550431	0.982649571	-4.956114825
hsa-miR-4524b-5p	MIMAT0022255	0.084477345	0.849857246	-0.843504697	0.412585559	0.93368138	-4.811240044
hsa-miR-4666a-3p	MIMAT0019742	0.084444055	0.8987695	-1.061815086	0.305609453	0.930208704	-4.694280179
hsa-mir-4458	MI0016804	0.084426262	1.029454509	-0.846924319	0.41073787	0.932955426	-4.809583286
hsa-miR-18b-3p	MIMAT0004751	0.084181934	0.964361691	-1.157414631	0.265732149	0.927057986	-4.636332385
hsa-mir-8054	MI0025890	0.084158064	0.856463888	-1.402082873	0.181842268	0.903156384	-4.471625216
hsa-miR-4784	MIMAT0019948	0.084154948	1.044650036	-0.775900019	0.450224054	0.950247229	-4.842788445
hsa-miR-4275	MIMAT0016905	0.084046944	0.785508965	-1.025889662	0.32168091	0.930208704	-4.715037319
hsa-miR-3119	MIMAT0014981	0.083859232	0.788088968	-1.210938009	0.245202438	0.919664114	-4.602239984
hsa-mir-4323	MI0015853	0.083835371	1.177217148	-1.22003412	0.241838856	0.919664114	-4.596332895
hsa-miR-146b-3p	MIMAT0004766	0.083648303	1.044323707	-0.75847867	0.460262709	0.951465791	-4.850541144
hsa-miR-187-5p	MIMAT0004561	0.083638436	1.066251643	-0.446841192	0.661562248	0.984005175	-4.961598797
hsa-miR-3156-5p	MIMAT0015030	0.083615201	0.885054255	-1.063843138	0.304720033	0.930208704	-4.693091453

hsa-mir-3181	MI0014223	0.08355415	1.109269578	-1.121222893	0.280339319	0.927057986	-4.658723824
hsa-mir-4670	MI0017301	0.083334462	0.986006918	-0.99965326	0.333797588	0.930208704	-4.729832196
hsa-miR-489-3p	MIMAT0002805	0.083235665	0.909933806	-0.926088368	0.369491423	0.930573075	-4.769630266
hsa-miR-6516-5p	MIMAT0030417	0.0832067	0.945068775	-0.644024607	0.52957142	0.960979619	-4.897513388
hsa-miR-601	MIMAT0003269	0.083199165	1.076789134	-0.675155387	0.510153938	0.960979619	-4.885427805
hsa-miR-6768-3p	MIMAT0027437	0.083124575	0.879252803	-1.434173056	0.172643524	0.903074508	-4.448441029
hsa-mir-8089	MI0025925	0.083051996	1.28636567	-0.745962119	0.467559904	0.951465791	-4.856014276
hsa-miR-4473	MIMAT0019000	0.083002427	0.820950945	-1.050503618	0.310605106	0.930208704	-4.700877251
hsa-miR-4755-3p	MIMAT0019896	0.082994178	0.937134831	-0.946496624	0.359334694	0.930573075	-4.758842586
hsa-mir-4468	MI0016819	0.082974222	0.835866181	-1.092486543	0.292360749	0.927057986	-4.67611172
hsa-miR-429	MIMAT0001536	0.082612925	0.994239815	-0.893987155	0.385863183	0.930573075	-4.786197553
hsa-mir-3684	MI0016085	0.082206937	0.928313003	-1.158731123	0.265211917	0.926762219	-4.6355077
hsa-mir-492	MI0003131	0.08219301	1.057882516	-0.75828583	0.4603746	0.951465791	-4.850626084
hsa-miR-519e-5p	MIMAT0002828	0.082170163	0.965524838	-0.884296297	0.390900532	0.930573075	-4.79110144
hsa-miR-5702	MIMAT0022495	0.081972033	0.899171015	-1.358837944	0.194869973	0.904897643	-4.50232198
hsa-mir-10b	MI0000267	0.081959903	0.945157707	-0.845796264	0.411346779	0.93320603	-4.810130454
hsa-miR-8067	MIMAT0030994	0.081892655	0.942604753	-0.683677751	0.50491076	0.960979619	-4.882028359
hsa-mir-598	MI0003610	0.081888698	1.020658439	-0.867371778	0.399803391	0.930573075	-4.799556173
hsa-miR-337-5p	MIMAT0004695	0.081860782	1.324403615	-0.447173425	0.66132789	0.984005175	-4.961509385
hsa-miR-4433a-3p	MIMAT0018949	0.081637026	6.558613366	-0.236139303	0.816613041	0.994603723	-5.005222709
hsa-miR-4778-3p	MIMAT0019937	0.081524605	0.760127167	-0.609697163	0.551456185	0.96620986	-4.910229744
hsa-mir-653	MI0003674	0.081367156	1.087995228	-0.546136381	0.593239776	0.970338677	-4.932058624
hsa-miR-4641	MIMAT0019701	0.081218147	0.892311971	-1.388618975	0.185819699	0.903156384	-4.481250555
hsa-mir-3178	MI0014212	0.081210693	1.022560001	-0.940405195	0.362345781	0.930573075	-4.762083055
hsa-mir-7-1	MI0000263	0.081193139	0.979695534	-1.070030719	0.302018157	0.929265009	-4.689453569
hsa-miR-4639-5p	MIMAT0019697	0.081118549	0.872918294	-0.946713986	0.359227571	0.930573075	-4.758726633
hsa-miR-4787-3p	MIMAT0019957	0.081101457	1.159158933	-0.804755633	0.433901317	0.943962572	-4.829605267
hsa-miR-496	MIMAT0002818	0.081099483	0.780848412	-1.471132385	0.162529134	0.903074508	-4.421325857
hsa-miR-3150a-5p	MIMAT0019206	0.081086604	0.96950936	-0.688291566	0.502085368	0.960979619	-4.880171771
hsa-mir-6133	MI0021278	0.081006983	0.843296695	-1.230901465	0.237867483	0.919664114	-4.589233236
hsa-mir-101-1	MI0000103	0.080771966	0.937451487	-0.989291048	0.338671778	0.930208704	-4.735589553
hsa-mir-5002	MI0017868	0.080472055	1.101949043	-0.839417286	0.414801202	0.934032076	-4.813212717
hsa-miR-124-3p	MIMAT0000422	0.080397639	0.971431506	-0.767077453	0.455290633	0.951465791	-4.846734137
hsa-miR-133a-5p	MIMAT0026478	0.080154244	0.883160461	-0.954290229	0.355507608	0.930573075	-4.754671212
hsa-miR-664b-5p	MIMAT0022271	0.080135641	1.116799082	-0.632202083	0.537053225	0.96159396	-4.90196561
hsa-miR-567	MIMAT0003231	0.08009057	0.878274916	-0.879449463	0.393436436	0.930573075	-4.793536985
hsa-mir-7973-2	MI0025749	0.080063888	0.95791725	-1.542751294	0.144335415	0.896940866	-4.367580373
hsa-mir-4790	MI0017437	0.079892144	0.936115963	-1.08802073	0.294262861	0.927057986	-4.678782336
hsa-miR-6515-3p	MIMAT0025487	0.079848187	1.047446477	-0.492852343	0.629463996	0.979368249	-4.948610454
hsa-miR-5579-3p	MIMAT0022270	0.079784374	0.754041121	-0.655584895	0.522312575	0.960979619	-4.893086536
hsa-let-7c	MI0000064	0.079532587	0.813964628	-1.058903995	0.306889462	0.930208704	-4.695983347
hsa-mir-367	MI0000775	0.079442179	0.921314758	-1.229850858	0.238249182	0.919664114	-4.589921601
hsa-mir-548ar	MI0019131	0.079403243	0.972416962	-1.136266291	0.274195932	0.927057986	-4.649482617
hsa-miR-204-5p	MIMAT0000265	0.079334448	1.02216669	-0.899134752	0.383205338	0.930573075	-4.783574219
hsa-mir-873	MI0005564	0.079320083	0.951658797	-0.928378515	0.368341924	0.930573075	-4.768429521
hsa-miR-6719-3p	MIMAT0025850	0.07929644	0.823048967	-1.092489626	0.292359438	0.927057986	-4.676109873
hsa-miR-892b	MIMAT0004918	0.079221984	0.883790493	-1.075723184	0.299548082	0.927057986	-4.686092076
hsa-mir-1208	MI0006341	0.079059078	0.943558796	-0.933584261	0.365738156	0.930573075	-4.76569085
hsa-miR-299-3p	MIMAT0000687	0.078889213	0.954733055	-1.04288607	0.314002774	0.930208704	-4.70528823
hsa-miR-3134	MIMAT0015000	0.078850456	0.936314643	-0.762721071	0.457805437	0.951465791	-4.848667645
hsa-miR-6875-3p	MIMAT0027651	0.078827175	1.000236248	-0.755244161	0.462141665	0.951465791	-4.851963289
hsa-mir-4640	MI0017267	0.078798755	0.987487363	-1.055574514	0.308358244	0.930208704	-4.697926748
hsa-miR-4528	MIMAT0019067	0.078714418	0.827181252	-1.030165663	0.319736569	0.930208704	-4.712596731
hsa-miR-6502-3p	MIMAT0025461	0.07866274	0.819125992	-1.069216093	0.302372862	0.929573508	-4.689933467
hsa-mir-3917	MI0016423	0.078336595	1.047841593	-0.647899406	0.527132081	0.960979619	-4.896037656
hsa-mir-658	MI0003682	0.078311692	0.934123604	-1.166152044	0.262293928	0.924045176	-4.630845807
hsa-mir-4744	MI0017382	0.078285465	0.948813094	-1.306028391	0.211793824	0.91645315	-4.538923727
hsa-miR-503-3p	MIMAT0022925	0.078266167	0.781246822	-1.006879561	0.330428206	0.930208704	-4.725788263
hsa-miR-1229-3p	MIMAT0005584	0.078229237	0.9714298	-1.498361332	0.155397647	0.903074508	-4.401075116
hsa-miR-6771-5p	MIMAT0027442	0.078190598	5.948578538	-0.191975382	0.850412311	0.996161815	-5.010999856
hsa-mir-4723	MI0017359	0.078145479	0.944801891	-1.306225682	0.211728475	0.91645315	-4.538788847
hsa-miR-4691-3p	MIMAT0019782	0.078135748	0.977724364	-0.712568929	0.487371855	0.958579725	-4.870216039
hsa-miR-298	MIMAT0004901	0.078090305	1.031447669	-1.114445056	0.283140792	0.927057986	-4.662856484
hsa-mir-1289-2	MI0006351	0.078060815	1.004868455	-0.786360515	0.444262818	0.948333516	-4.83805847
hsa-let-7a-2-3p	MIMAT0010195	0.077760786	0.803365558	-1.144350488	0.27093679	0.927057986	-4.644477572
hsa-miR-4519	MIMAT0019056	0.077693853	0.974878371	-0.83491747	0.417249372	0.935561983	-4.81537481

hsa-mir-378a	MI0000786	0.077579996	0.839913857	-1.449252411	0.168455599	0.903074508	-4.437430688
hsa-miR-6077	MIMAT0023702	0.077445021	1.014541815	-1.075850542	0.29949299	0.927057986	-4.686016709
hsa-miR-5000-3p	MIMAT0021020	0.077433155	0.896126288	-0.886688479	0.389652976	0.930573075	-4.789895153
hsa-miR-3130-3p	MIMAT0014994	0.077368214	1.006016734	-0.838280726	0.415418671	0.93437823	-4.813759769
hsa-miR-597-5p	MIMAT0003265	0.077314254	0.761504836	-0.884512616	0.390787608	0.930573075	-4.790992473
hsa-mir-548c	MI0003630	0.077202875	0.942266809	-0.839926299	0.41452486	0.933957172	-4.812967509
hsa-miR-323b-5p	MIMAT0001630	0.077193364	0.938097123	-0.803050753	0.434855098	0.944553236	-4.830395946
hsa-mir-3162	MI0014192	0.077110489	0.904579097	-0.935148884	0.364958063	0.930573075	-4.764865216
hsa-miR-6825-3p	MIMAT0027551	0.07678882	0.880901717	-0.938309938	0.363385526	0.930573075	-4.763193635
hsa-miR-6071	MIMAT0023696	0.076759492	0.755731819	-0.780176093	0.447781165	0.950247229	-4.840861683
hsa-mir-548h-1	MI0006411	0.076752606	0.97268819	-0.857919172	0.404834032	0.930588488	-4.804217204
hsa-mir-7843	MI0025510	0.076574872	0.974706657	-1.398682924	0.18284002	0.903156384	-4.474061587
hsa-miR-3198	MIMAT0015083	0.076521422	0.896475613	-1.048485134	0.311502793	0.930208704	-4.702048558
hsa-miR-7850-5p	MIMAT0030425	0.07651047	1.026903167	-1.356388162	0.195630105	0.904897643	-4.504041697
hsa-mir-4465	MI0016816	0.076389108	0.956443964	-0.835808017	0.416764115	0.934881655	-4.814947717
hsa-mir-4643	MI0017270	0.076343513	0.884594925	-1.185012802	0.254988094	0.921665152	-4.618896836
hsa-miR-548v	MIMAT0015020	0.076307427	0.832178403	-0.890357871	0.387744547	0.930573075	-4.788039419
hsa-miR-4760-5p	MIMAT0019906	0.07630057	0.917129294	-1.325538497	0.205409119	0.908865348	-4.5255173
hsa-miR-4649-5p	MIMAT0019711	0.076281141	5.207385497	-0.136656108	0.893173636	0.999057213	-5.016564763
hsa-miR-516b-5p	MIMAT0002859	0.076095134	0.934469592	-1.02974754	0.319926318	0.930208704	-4.712835741
hsa-mir-3619	MI0016009	0.076092607	0.989131582	-0.898189933	0.383692243	0.930573075	-4.784056679
hsa-miR-4748	MIMAT0019884	0.076084122	1.009342643	-0.705459574	0.491653734	0.959967659	-4.873163833
hsa-miR-598-3p	MIMAT0003266	0.07592467	0.855631233	-0.871534045	0.397601481	0.930573075	-4.797489869
hsa-mir-6732	MI0022577	0.075880866	0.961890853	-1.094469803	0.291518956	0.927057986	-4.674922964
hsa-miR-3199	MIMAT0015084	0.075804352	0.91472921	-0.810586179	0.430649584	0.94301493	-4.826890097
hsa-miR-4714-3p	MIMAT0019823	0.075628145	1.063659847	-0.737967918	0.472257455	0.952334436	-4.859467278
hsa-miR-5579-5p	MIMAT0022269	0.075611121	1.00511369	-1.099333474	0.289462205	0.927057986	-4.672000587
hsa-miR-6775-3p	MIMAT0027451	0.07557653	0.868752481	-0.713895698	0.48657523	0.957997689	-4.869662956
hsa-miR-4670-5p	MIMAT0019750	0.075389185	0.867404162	-0.837558983	0.415811091	0.934467968	-4.814106826
hsa-miR-147b-3p	MIMAT0004928	0.075335177	0.783620659	-0.886140766	0.38993838	0.930573075	-4.79017159
hsa-miR-609	MIMAT0003277	0.075245561	0.813809036	-1.093565653	0.291902498	0.927057986	-4.675465116
hsa-miR-656-5p	MIMAT0026627	0.074855543	1.023082628	-0.931263414	0.366897407	0.930573075	-4.766913401
hsa-mir-300	MI0005525	0.074844739	0.863551709	-0.829806547	0.42004142	0.936613328	-4.817818279
hsa-miR-1307-3p	MIMAT0005951	0.074837731	4.026272927	-0.132340727	0.896525721	0.999057213	-5.016920434
hsa-miR-3651	MIMAT0018071	0.074741202	0.867663801	-0.897436393	0.384080874	0.930573075	-4.784441156
hsa-miR-6839-3p	MIMAT0027581	0.074608765	0.7425958	-1.286672244	0.218283818	0.917147059	-4.552087451
hsa-mir-3912	MI0016416	0.074498565	0.874390035	-1.147768477	0.269567685	0.927057986	-4.642353317
hsa-mir-4641	MI0017268	0.074409698	0.848632871	-0.923904426	0.370589909	0.930573075	-4.770773007
hsa-mir-634	MI0003649	0.074400953	0.856431287	-0.883475349	0.391329285	0.930573075	-4.791514773
hsa-miR-4765	MIMAT0019916	0.074278244	0.933771856	-0.800141715	0.436485621	0.944812484	-4.83174168
hsa-miR-4779	MIMAT0019938	0.074238306	1.116307738	-0.627986346	0.539735279	0.962946437	-4.903534786
hsa-miR-4801	MIMAT0019980	0.074086052	0.944218494	-1.099780289	0.289273799	0.927057986	-4.671731609
hsa-miR-6718-5p	MIMAT0025849	0.074054861	0.934578292	-0.875012925	0.395767306	0.930573075	-4.795756319
hsa-miR-4648	MIMAT0019710	0.073871389	1.037802109	-0.802630009	0.435090685	0.944553236	-4.830590849
hsa-mir-2278	MI0011285	0.073768383	0.945122777	-0.807343938	0.43245587	0.94301493	-4.828402066
hsa-miR-510-5p	MIMAT0002882	0.073704381	1.0035004	-0.88410381	0.391001033	0.930573075	-4.791198384
hsa-mir-670	MI0003933	0.073680517	0.873110475	-0.855486379	0.4061355	0.930793333	-4.805409684
hsa-mir-1539	MI0007260	0.073626391	1.06539449	-0.736327793	0.473224769	0.952660318	-4.860171589
hsa-miR-100-3p	MIMAT0004512	0.073624365	0.781047345	-1.060035395	0.306391519	0.930208704	-4.695321848
hsa-miR-135a-5p	MIMAT0000428	0.073477247	0.877690073	-0.673468481	0.51119549	0.960979619	-4.886096074
hsa-mir-6892	MI0022739	0.073365513	0.813769062	-1.332294724	0.203234494	0.905221726	-4.520842859
hsa-mir-182	MI0000272	0.07335851	1.435183912	-0.875685952	0.395413119	0.930573075	-4.795420262
hsa-miR-556-5p	MIMAT0003220	0.073344847	0.891009421	-1.084525945	0.295757772	0.927057986	-4.680866289
hsa-miR-1197	MIMAT0005955	0.073282518	0.959314234	-0.710082003	0.488867165	0.959021141	-4.871250247
hsa-miR-5187-3p	MIMAT0021118	0.072902405	0.779359297	-0.885554799	0.390243872	0.930573075	-4.790467172
hsa-miR-8053	MIMAT0030980	0.072856758	0.767842795	-0.816183956	0.427542418	0.942766561	-4.824267174
hsa-miR-548au-5p	MIMAT0022291	0.072799419	1.078102376	-0.611243647	0.550459762	0.966076637	-4.909670739
hsa-mir-548g	MI0006395	0.072752296	0.900643119	-0.852796611	0.407577654	0.931695939	-4.806724725
hsa-miR-621	MIMAT0003290	0.072718952	0.839315133	-0.900924972	0.38228391	0.930573075	-4.782658886
hsa-mir-7855	MI0025525	0.07270579	0.923717667	-0.783249292	0.446030624	0.949212502	-4.83947114
hsa-miR-4680-3p	MIMAT0019765	0.072677546	0.839318663	-1.276145542	0.22187913	0.917147059	-4.559188404
hsa-miR-6886-5p	MIMAT0027672	0.0725715	1.059480252	-0.880146436	0.393071097	0.930573075	-4.793187458
hsa-miR-186-3p	MIMAT0004612	0.072541126	1.098620714	-0.934855597	0.365104203	0.930573075	-4.765020068
hsa-miR-340-5p	MIMAT0004692	0.072517699	1.135562565	-0.554360426	0.587743911	0.968332399	-4.929361105
hsa-mir-192	MI0000234	0.07230153	0.862747003	-0.828915723	0.420529308	0.936848998	-4.818242836
hsa-mir-451b	MI0017360	0.07224284	0.982306533	-0.938331564	0.363374784	0.930573075	-4.763182183

hsa-miR-767-5p	MIMAT0003882	0.072064915	0.805804365	-1.079495208	0.29791955	0.927057986	-4.6838569
hsa-miR-548au-3p	MIMAT0022292	0.071921311	0.844895871	-1.014993137	0.326674199	0.930208704	-4.721219627
hsa-miR-579-3p	MIMAT0003244	0.071899326	0.933675188	-1.141690962	0.272005724	0.927057986	-4.64612711
hsa-mir-429	MI0001641	0.071856639	0.904077797	-0.876248022	0.395117485	0.930573075	-4.795139438
hsa-mir-935	MI0005757	0.071783399	1.027186844	-0.978683567	0.343713429	0.930208704	-4.741432276
hsa-mir-9-3	MI0000468	0.071755375	0.829936898	-1.326575947	0.205073985	0.908342323	-4.524800578
hsa-mir-3159	MI0014188	0.071668427	0.943897734	-0.451095585	0.65856396	0.984005175	-4.960449016
hsa-mir-299	MI0000744	0.071668373	0.951094387	-0.946340302	0.359411749	0.930573075	-4.758925963
hsa-miR-98-3p	MIMAT0022842	0.071556869	0.993322032	-0.599951692	0.557757813	0.96620986	-4.913722131
hsa-mir-374a	MI0000782	0.071544296	0.854293412	-0.947444535	0.358867694	0.930573075	-4.758336756
hsa-miR-5002-3p	MIMAT0021024	0.071402077	0.997305083	-0.988147963	0.339212542	0.930208704	-4.736221659
hsa-mir-6795	MI0022640	0.071398455	0.92114567	-0.918254401	0.373442167	0.930573075	-4.77371883
hsa-miR-648	MIMAT0003318	0.07139185	1.31595114	-0.777594993	0.449254731	0.950247229	-4.842025826
hsa-mir-4306	MI0015836	0.071341837	0.963970911	-0.650933028	0.525226728	0.960979619	-4.894876608
hsa-miR-128-1-5p	MIMAT0026477	0.071260156	0.793407325	-1.033329771	0.318303298	0.930208704	-4.710785518
hsa-mir-3675	MI0016076	0.071208712	0.934826368	-1.034290339	0.317869104	0.930208704	-4.710234783
hsa-mir-6874	MI0022721	0.071185486	0.902337391	-0.890633403	0.387601499	0.930573075	-4.78789981
hsa-miR-4327	MIMAT0016889	0.071170318	1.230522575	-0.375475893	0.712718338	0.9869374	-4.97931512
hsa-miR-657	MIMAT0003335	0.071047892	0.909503109	-1.125683279	0.278507102	0.927057986	-4.655993658
hsa-mir-1297	MI0006358	0.071042844	0.937462627	-1.053246412	0.309388323	0.930208704	-4.699282759
hsa-mir-6780b	MI0022681	0.070964366	0.859186261	-1.2212354	0.241397338	0.919664114	-4.595550355
hsa-mir-3605	MI0015995	0.070859603	1.099551657	-0.577418728	0.572475068	0.967644566	-4.921596035
hsa-mir-6840	MI0022686	0.070793979	0.837240981	-0.994655238	0.336142289	0.930208704	-4.732615268
hsa-mir-4506	MI0016869	0.070731047	0.913000789	-0.778664471	0.448643791	0.950247229	-4.841543878
hsa-miR-5009-5p	MIMAT0021041	0.07072312	0.862811603	-0.995654967	0.335672355	0.930208704	-4.732059495
hsa-mir-614	MI0003627	0.070718423	1.022585118	-1.040639786	0.315009831	0.930208704	-4.706584054
hsa-miR-4428	MIMAT0018943	0.07071354	1.04259696	-0.665548543	0.516101901	0.960979619	-4.889213123
hsa-mir-3668	MI0016069	0.07069589	0.913824478	-0.654588301	0.522936115	0.960979619	-4.893471017
hsa-mir-3978	MI0016996	0.070505833	0.942152884	-0.836165152	0.416569616	0.934671969	-4.814776329
hsa-mir-4294	MI0015827	0.070433784	0.87120768	-0.844290083	0.412160718	0.933595576	-4.810860049
hsa-miR-4637	MIMAT0019694	0.070322583	0.776130511	-1.003121185	0.332177564	0.930208704	-4.727894458
hsa-mir-4741	MI0017379	0.070278273	1.170714627	-0.826836083	0.421669721	0.937848766	-4.819232422
hsa-mir-1199	MI0020340	0.070162444	0.882149456	-0.824177811	0.423130355	0.939362308	-4.820494193
hsa-mir-7850	MI0025520	0.07008579	1.161021488	-0.843986334	0.412324992	0.93366101	-4.811007049
hsa-mir-4492	MI0016854	0.069915971	0.972659744	-0.835602179	0.416876243	0.934898338	-4.815046469
hsa-miR-6793-5p	MIMAT0027486	0.069870562	1.404353695	-0.341826906	0.737363211	0.989764242	-4.986630996
hsa-miR-7111-3p	MIMAT0028120	0.069806618	1.063541204	-0.932651991	0.366203516	0.930573075	-4.766182247
hsa-miR-544b	MIMAT0015004	0.069732373	0.811762952	-0.775987235	0.450174145	0.950247229	-4.84274924
hsa-mir-4748	MI0017387	0.069532991	1.07213258	-0.934700881	0.365181312	0.930573075	-4.76510174
hsa-mir-4644	MI0017271	0.069499407	0.982274908	-0.612523226	0.549636053	0.965790957	-4.909207218
hsa-mir-4638	MI0017265	0.069378054	0.991384154	-0.895197092	0.385237342	0.930573075	-4.785582091
hsa-miR-6828-3p	MIMAT0027557	0.069299217	0.904570343	-0.972575106	0.346640667	0.930573075	-4.744773389
hsa-mir-3942	MI0016599	0.069284667	0.957203175	-1.104750929	0.28718401	0.927057986	-4.668733595
hsa-miR-6812-3p	MIMAT0027525	0.069245489	0.954457162	-0.661630629	0.518539006	0.960979619	-4.890742618
hsa-mir-3614	MI0016004	0.069217888	0.860367016	-0.950533974	0.357348574	0.930573075	-4.756685226
hsa-mir-548ag-2	MI0016794	0.069204902	0.975436978	-0.637254621	0.533848582	0.960979619	-4.900072193
hsa-mir-6805	MI0022650	0.069174	1.022498379	-0.50801232	0.61905122	0.97740883	-4.944065254
hsa-miR-5591-5p	MIMAT0022301	0.06912425	0.924655459	-0.746380131	0.467315062	0.951465791	-4.855832805
hsa-miR-6878-3p	MIMAT0027657	0.068877932	0.798892561	-1.137515796	0.273690267	0.927057986	-4.648710799
hsa-mir-4725	MI0017362	0.068770423	1.098193302	-0.807022308	0.432635317	0.94301493	-4.828551764
hsa-mir-432	MI0003133	0.068755912	0.877512454	-0.812978586	0.429319858	0.94301493	-4.825771032
hsa-mir-6718	MI0022553	0.068727957	0.932294552	-0.785998101	0.444468515	0.94833899	-4.838223281
hsa-mir-15a	MI0000069	0.068701429	1.02414441	-0.486464192	0.633876449	0.98055505	-4.950486426
hsa-miR-4517	MIMAT0019054	0.068528687	0.947321133	-0.867179982	0.39990505	0.930573075	-4.799651182
hsa-miR-1185-5p	MIMAT0005798	0.068454639	0.931048612	-1.084558407	0.29574386	0.927057986	-4.680846956
hsa-miR-4785	MIMAT0019949	0.068376585	1.269440865	-0.564275039	0.581153096	0.968332399	-4.926058673
hsa-mir-490	MI0003125	0.068363782	0.954744863	-1.134404412	0.274950727	0.927057986	-4.650631493
hsa-miR-31-5p	MIMAT0000089	0.068300413	0.951839578	-0.468042257	0.646681327	0.982649571	-4.955765312
hsa-mir-106b	MI0000734	0.068281418	1.036661291	-0.732798843	0.475310157	0.953466088	-4.861682241
hsa-mir-5190	MI0018169	0.068224666	0.840108441	-1.012939633	0.327621412	0.930208704	-4.722378734
hsa-miR-450a-2-3p	MIMAT0031074	0.067955901	0.924150002	-0.61087746	0.550695614	0.966076637	-4.909803223
hsa-miR-6734-5p	MIMAT0027369	0.067743333	1.15281156	-0.593133812	0.562189334	0.96620986	-4.916134236
hsa-mir-155	MI0000681	0.067707191	1.339692139	-0.717345027	0.48450781	0.957574873	-4.868220705
hsa-mir-411	MI0003675	0.067668994	1.190363191	-0.616882482	0.546834898	0.965790957	-4.90762135
hsa-miR-376a-5p	MIMAT0003386	0.067626123	0.857132706	-1.139718533	0.272800555	0.927057986	-4.647348595
hsa-mir-4692	MI0017325	0.067583178	1.026806845	-0.638085855	0.533322385	0.960979619	-4.899759359

hsa-miR-3612	MIMAT0017989	0.067498449	0.980423838	-0.833218212	0.418176315	0.935967201	-4.816188652
hsa-mir-2117	MI0010636	0.067408606	0.899778013	-1.163871723	0.263187956	0.924680755	-4.632280715
hsa-miR-6833-5p	MIMAT0027566	0.067401341	1.131338333	-0.65224115	0.524406329	0.960979619	-4.894374416
hsa-mir-548au	MI0019145	0.067332076	0.918567288	-0.74416852	0.468611361	0.951465791	-4.856791898
hsa-miR-3619-3p	MIMAT0019219	0.067279329	0.932918649	-0.590517874	0.563894635	0.96620986	-4.917052908
hsa-mir-6807	MI0022652	0.067104736	1.097825788	-0.765235147	0.456353089	0.951465791	-4.847553009
hsa-miR-4720-5p	MIMAT0019833	0.06708866	0.855515384	-1.001047211	0.333145735	0.930208704	-4.729053969
hsa-miR-6862-3p	MIMAT0027626	0.067073096	1.024358891	-0.589532507	0.5645377	0.96620986	-4.917397969
hsa-mir-130a	MI0000448	0.066935159	0.997851852	-1.055934242	0.308199305	0.930208704	-4.697717011
hsa-mir-183	MI0000273	0.066897494	1.00492236	-0.694707041	0.498172097	0.960979619	-4.87757133
hsa-mir-3163	MI0014193	0.066712915	1.002062453	-0.577811342	0.572216897	0.967644566	-4.921461251
hsa-miR-33b-3p	MIMAT0004811	0.066507234	0.970644674	-0.931244119	0.366907055	0.930573075	-4.766923554
hsa-mir-181b-2	MI0000683	0.066407431	1.076972271	-0.579763628	0.570934042	0.967644566	-4.920789764
hsa-mir-548v	MI0014174	0.066285654	0.878174416	-0.894554383	0.385569699	0.930573075	-4.785909108
hsa-miR-6824-5p	MIMAT0027548	0.06617896	4.17097739	-0.089984192	0.929525529	0.999227185	-5.019807229
hsa-miR-193b-3p	MIMAT0002819	0.065971577	0.994842626	-1.082227718	0.296743908	0.927057986	-4.682233866
hsa-mir-6771	MI0022616	0.065954361	1.00937303	-0.852800542	0.407575544	0.931695939	-4.806722806
hsa-mir-3116-1	MI0014128	0.065941199	0.892290348	-0.914176924	0.375509879	0.930573075	-4.775835296
hsa-mir-1276	MI0006416	0.065824195	0.897070711	-1.055296662	0.308481049	0.930208704	-4.698088709
hsa-miR-6762-3p	MIMAT0027425	0.065675815	0.862277042	-0.796139607	0.43873517	0.946548346	-4.833586056
hsa-mir-6766	MI0022611	0.065652717	0.881478958	-0.858953603	0.40428148	0.930588488	-4.803709274
hsa-mir-576	MI0003583	0.06557053	0.898000859	-0.9545259	0.355392325	0.930573075	-4.754544631
hsa-mir-7155	MI0023615	0.065491218	0.845215686	-0.980738824	0.342732463	0.930208704	-4.740304255
hsa-miR-1199-5p	MIMAT0031119	0.065382613	0.871133219	-0.897760191	0.383913846	0.930573075	-4.784275979
hsa-miR-5004-5p	MIMAT0021027	0.065280792	1.051648782	-0.662996327	0.517688739	0.960979619	-4.890210409
hsa-mir-584	MI0003591	0.065266905	1.072929804	-0.788415963	0.443097336	0.947408294	-4.837122469
hsa-miR-4474-5p	MIMAT0019234	0.065130831	0.804653395	-1.117286534	0.281963785	0.927057986	-4.6611263
hsa-mir-328	MI0000804	0.065092012	0.994776377	-0.605217234	0.554348193	0.96620986	-4.911841669
hsa-mir-3155b	MI0016839	0.065008522	1.101940478	-0.521016541	0.610185907	0.97572307	-4.940062189
hsa-mir-6726	MI0022571	0.064562196	0.957541735	-0.889383841	0.388250519	0.930573075	-4.788532655
hsa-miR-500b-5p	MIMAT0016925	0.06439592	0.990308583	-0.527897421	0.605520368	0.975047002	-4.937905272
hsa-miR-585-3p	MIMAT0003250	0.064307551	0.806559175	-0.950387789	0.357420354	0.930573075	-4.756763473
hsa-miR-7161-5p	MIMAT0028232	0.064091947	1.068709386	-0.568185295	0.578564272	0.967979651	-4.924741103
hsa-miR-302f	MIMAT0005932	0.064089698	0.889954712	-1.313987514	0.209170279	0.914362911	-4.533471194
hsa-miR-3127-5p	MIMAT0014990	0.064020744	1.113329436	-0.585129437	0.567415963	0.966465648	-4.918933287
hsa-miR-4636	MIMAT0019693	0.063900046	0.900801183	-0.820757724	0.425014393	0.940652535	-4.822112353
hsa-miR-208a-3p	MIMAT0000241	0.06370833	1.161652841	-0.475743603	0.641313811	0.982649571	-4.953582157
hsa-mir-128-2	MI0000727	0.063648575	0.960113253	-0.665913148	0.515875436	0.960979619	-4.889070366
hsa-mir-5680	MI0019280	0.063620837	0.919833324	-0.665286576	0.51626465	0.960979619	-4.889315649
hsa-miR-6765-3p	MIMAT0027431	0.063584703	1.053303209	-0.579570643	0.571060785	0.967644566	-4.920856235
hsa-miR-5683	MIMAT0022472	0.063543632	0.872690367	-0.937059478	0.364007035	0.930573075	-4.763855449
hsa-mir-410	MI0002465	0.063287449	1.049330313	-0.715048246	0.485883842	0.957574873	-4.869181745
hsa-miR-4632-3p	MIMAT0019688	0.063266242	0.915408459	-0.710194555	0.488799432	0.959021141	-4.871203512
hsa-miR-582-5p	MIMAT0003247	0.063243306	0.845846642	-0.999813869	0.333722436	0.930208704	-4.729742575
hsa-miR-211-3p	MIMAT0022694	0.063124254	1.03862636	-0.706106413	0.49126323	0.959742702	-4.872896736
hsa-mir-6894	MI0022741	0.062772893	0.877117006	-0.764015089	0.457057542	0.951465791	-4.848094339
hsa-miR-3170	MIMAT0015045	0.062745148	0.852764965	-1.007150787	0.330302218	0.930208704	-4.725636021
hsa-miR-6783-3p	MIMAT0027467	0.062718388	0.876185072	-0.633054143	0.536512045	0.96159396	-4.90164728
hsa-miR-3156-3p	MIMAT0019209	0.062666284	0.968190022	-0.923137409	0.370976237	0.930573075	-4.771173807
hsa-mir-5702	MI0019309	0.062647593	0.884712996	-0.953060257	0.356109696	0.930573075	-4.755331421
hsa-mir-4251	MI0015861	0.062647248	0.859376185	-1.002291514	0.332564629	0.930208704	-4.728358542
hsa-mir-92a-1	MI0000093	0.062499393	0.878566105	-0.845603535	0.411450871	0.93320603	-4.810223875
hsa-mir-8073	MI0025909	0.062430818	0.885641287	-0.867947839	0.399498163	0.930573075	-4.799270701
hsa-miR-3622b-3p	MIMAT0018006	0.062362442	0.934330649	-0.766003039	0.455910059	0.951465791	-4.847211908
hsa-miR-892c-3p	MIMAT0025858	0.062318484	0.823186135	-0.704337394	0.492331646	0.960113358	-4.873626685
hsa-mir-6893	MI0022740	0.062254989	0.939456164	-0.805973549	0.433220784	0.943297642	-4.829039528
hsa-miR-7106-3p	MIMAT0028110	0.062248732	0.978511214	-0.431211919	0.672628296	0.984005175	-4.965732698
hsa-miR-548ar-5p	MIMAT0022265	0.062241635	1.173138786	-0.539030712	0.59800914	0.972358931	-4.934358699
hsa-miR-6793-3p	MIMAT0027487	0.062177585	1.410281129	-0.643964555	0.529609275	0.960979619	-4.897536195
hsa-mir-4786	MI0017433	0.06213644	0.900149063	-0.686146691	0.503397687	0.960979619	-4.881036277
hsa-miR-4288	MIMAT0016918	0.062123601	0.887504228	-0.64795542	0.527096864	0.960979619	-4.896016263
hsa-miR-548z	MIMAT0018446	0.062108374	0.965492288	-0.819411804	0.42575731	0.941399883	-4.822747543
hsa-miR-548h-3p	MIMAT0022723	0.062108374	0.965492288	-0.819411804	0.42575731	0.941399883	-4.822747543
hsa-miR-548d-3p	MIMAT0003323	0.062096865	0.880084245	-0.720902719	0.482380965	0.957574873	-4.866726577
hsa-miR-4300	MIMAT0016853	0.062028682	0.906545051	-0.697192886	0.496660631	0.960979619	-4.876557831
hsa-miR-139-5p	MIMAT0000250	0.061955061	6.694737339	-0.252242989	0.804378068	0.99449721	-5.002822751

hsa-miR-519c-3p	MIMAT0002832	0.061839731	1.003172584	-0.647661147	0.527281891	0.960979619	-4.896128633
hsa-miR-662	MIMAT0003325	0.061790203	0.910444617	-0.568279491	0.578501982	0.967979651	-4.924709258
hsa-mir-551a	MI0003556	0.061742447	0.899167925	-0.809831667	0.431069498	0.94301493	-4.827242426
hsa-miR-517c-3p	MIMAT0002866	0.061686386	0.890661074	-0.892192552	0.386792706	0.930573075	-4.787109113
hsa-miR-208b-5p	MIMAT0026722	0.061581437	0.855963896	-0.847566778	0.410391344	0.932667949	-4.809271377
hsa-miR-520g-3p	MIMAT0002858	0.06146624	0.837284939	-1.010295204	0.328844101	0.930208704	-4.723868589
hsa-miR-542-3p	MIMAT0003389	0.061413503	0.905765215	-0.811813402	0.42996715	0.94301493	-4.826316416
hsa-miR-541-5p	MIMAT0004919	0.061315581	1.029331803	-0.636859819	0.534098606	0.960979619	-4.900220644
hsa-miR-4649-3p	MIMAT0019712	0.061177932	0.99726086	-0.653543774	0.523590099	0.960979619	-4.893873415
hsa-mir-1269b	MI0016888	0.061166695	1.016213523	-0.598359436	0.558791072	0.96620986	-4.914287755
hsa-miR-5571-5p	MIMAT0022257	0.061012761	1.620257876	-0.310840367	0.760326064	0.99324537	-4.992774176
hsa-mir-3928	MI0016438	0.060904267	1.005799524	-0.705303353	0.491748075	0.959967659	-4.873228307
hsa-mir-3972	MI0016990	0.060889301	0.97945821	-0.757849812	0.460627648	0.951465791	-4.850818064
hsa-miR-3184-3p	MIMAT0022731	0.060804781	1.044548367	-0.532001708	0.602745915	0.974069804	-4.93660597
hsa-miR-6780a-3p	MIMAT0027461	0.060667721	0.871518709	-0.952950622	0.356163398	0.930573075	-4.755390235
hsa-miR-6514-3p	MIMAT0025485	0.060647893	0.801655974	-0.841300549	0.413779386	0.93368138	-4.812304846
hsa-mir-3657	MI0016057	0.060627402	0.949684582	-0.486289804	0.633997106	0.98055505	-4.95053731
hsa-mir-553	MI0003558	0.06059867	0.964918235	-0.753461353	0.463179335	0.951465791	-4.852744835
hsa-miR-4713-3p	MIMAT0019821	0.060495955	1.046168375	-0.57103954	0.576678381	0.96794959	-4.923773971
hsa-miR-31-3p	MIMAT0004504	0.060491051	1.02412459	-0.590869604	0.563665186	0.96620986	-4.916929607
hsa-miR-122b-3p	MIMAT0019877	0.060450295	1.086630696	-0.467254547	0.647231483	0.982649571	-4.955986687
hsa-mir-1207	MI0006340	0.0602806	0.917125784	-0.643642308	0.529812436	0.960979619	-4.897658547
hsa-miR-4680-5p	MIMAT0019764	0.060247923	0.929594391	-0.512516635	0.615973471	0.976745744	-4.94268957
hsa-mir-4495	MI0016857	0.060126424	0.969843185	-0.653123521	0.523853353	0.960979619	-4.894035149
hsa-mir-450b	MI0005531	0.059808737	0.964846118	-0.720039721	0.482896363	0.957574873	-4.867089623
hsa-miR-4799-3p	MIMAT0019977	0.059807927	0.793205777	-0.739836423	0.471156916	0.952023826	-4.858663184
hsa-miR-4678	MIMAT0019762	0.059784237	0.917661798	-0.645853169	0.528419477	0.960979619	-4.896817988
hsa-mir-8053	MI0025889	0.059673263	0.856882159	-0.648620404	0.526678884	0.960979619	-4.895762162
hsa-miR-6736-3p	MIMAT0027374	0.059625839	0.984746605	-0.806931804	0.43268582	0.94301493	-4.828593877
hsa-mir-1243	MI0006373	0.059467019	1.016573725	-0.577057925	0.572712375	0.967644566	-4.921719822
hsa-miR-181b-3p	MIMAT0022692	0.059330701	0.900682466	-0.65076	0.525335298	0.960979619	-4.894942964
hsa-mir-3659	MI0016060	0.0592943	1.041665432	-0.641679203	0.531051023	0.960979619	-4.898402685
hsa-miR-593-5p	MIMAT0003261	0.05923935	1.18762692	-0.530366002	0.603850876	0.974777836	-4.937124929
hsa-miR-5194	MIMAT0021125	0.059056121	1.16920508	-0.429071759	0.674149798	0.984627063	-4.966287726
hsa-mir-378d-2	MI0003840	0.058924557	0.859786345	-0.869344981	0.398758523	0.930573075	-4.798577659
hsa-mir-19b-2	MI0000075	0.058891973	0.923996037	-0.846713186	0.410851792	0.933014442	-4.809685745
hsa-mir-301a	MI0000745	0.058599452	0.906859251	-0.756148717	0.461615723	0.951465791	-4.851566121
hsa-mir-4326	MI0015866	0.058414789	0.863669686	-0.651489885	0.524877402	0.960979619	-4.894662942
hsa-miR-4727-5p	MIMAT0019847	0.058384759	0.879589817	-0.675995771	0.509635515	0.960979619	-4.885094318
hsa-miR-371a-3p	MIMAT0000723	0.058336922	1.008020673	-0.75690974	0.461173524	0.951465791	-4.851231646
hsa-mir-4252	MI0015864	0.058311463	1.002586939	-0.563867479	0.581423269	0.968332399	-4.92619551
hsa-mir-5192	MI0018171	0.058269377	0.965076929	-0.653435927	0.523657649	0.960979619	-4.893914929
hsa-miR-4804-3p	MIMAT0019985	0.058179082	0.922201041	-0.66039972	0.519306039	0.960979619	-4.891221438
hsa-miR-1296-5p	MIMAT0005794	0.058106233	1.028522194	-0.464305702	0.649292919	0.982649571	-4.956812248
hsa-miR-4770	MIMAT0019924	0.058046508	0.913982607	-0.518996765	0.611558748	0.976286456	-4.940690228
hsa-mir-6081	MI0020358	0.05804527	0.900505044	-0.659591159	0.51981024	0.960979619	-4.891535521
hsa-miR-632	MIMAT0003302	0.057948664	0.801733902	-0.645617236	0.528568029	0.960979619	-4.896907815
hsa-miR-17-5p	MIMAT0000070	0.057928094	10.23253494	-0.513747105	0.615133998	0.976745744	-4.942311761
hsa-mir-548x-2	MI0016833	0.057883189	0.928430599	-0.656432876	0.521782351	0.960979619	-4.892758965
hsa-mir-4675	MI0017306	0.057829748	0.9037848	-0.811115631	0.430355081	0.94301493	-4.826642691
hsa-miR-19b-1-5p	MIMAT0004491	0.057798567	0.868317339	-0.776174277	0.450067122	0.950247229	-4.842665148
hsa-mir-485	MI0002469	0.057684504	0.890922776	-0.77077421	0.453163369	0.951465791	-4.845085721
hsa-miR-4430	MIMAT0018945	0.057649048	2.106952819	-0.215726286	0.832193003	0.996080495	-5.008039774
hsa-mir-1323	MI0003786	0.057600587	1.09562695	-0.844797353	0.411886472	0.933439868	-4.810614453
hsa-miR-449c-3p	MIMAT0013771	0.057545383	0.787080314	-0.666438024	0.515549523	0.960979619	-4.888864732
hsa-mir-3128	MI0014145	0.057481893	0.866558261	-0.871732684	0.3974966	0.930573075	-4.797391045
hsa-miR-7153-5p	MIMAT0028216	0.057355775	0.903897006	-0.859111438	0.404197215	0.930588488	-4.803631727
hsa-mir-3136	MI0014158	0.057323414	0.876268954	-0.62898944	0.539096439	0.96223264	-4.903162296
hsa-mir-2113	MI0003939	0.057269672	0.962610763	-0.647769729	0.527213615	0.960979619	-4.896087176
hsa-miR-1183	MIMAT0005828	0.057195764	1.261271599	-0.325870279	0.749157261	0.990891279	-4.989865791
hsa-miR-6780a-5p	MIMAT0027460	0.057097641	1.133227286	-0.656844109	0.521525326	0.960979619	-4.892599969
hsa-mir-508	MI0003195	0.057062889	0.91055928	-0.800281335	0.436407275	0.944812484	-4.83167719
hsa-miR-3664-5p	MIMAT0018086	0.056776802	0.876167422	-0.814826931	0.428294336	0.94301493	-4.824904479
hsa-mir-4511	MI0016877	0.05660566	0.984358393	-1.119039807	0.28123937	0.927057986	-4.66005703
hsa-miR-6731-5p	MIMAT0027363	0.056579563	0.994495933	-0.559478451	0.58433689	0.968332399	-4.927663225
hsa-mir-98	MI0000100	0.056290041	1.09132814	-0.449566384	0.659640971	0.984005175	-4.960863499

hsa-miR-802	MIMAT0004185	0.05620177	0.951686108	-0.446601086	0.661731643	0.984005175	-4.961663375
hsa-miR-1225-3p	MIMAT0005573	0.056191385	1.036162863	-0.572910274	0.575444069	0.967644566	-4.923137627
hsa-miR-3615	MIMAT0017994	0.056046227	1.379348235	-0.362742348	0.722007349	0.988653693	-4.9821623
hsa-miR-374a-5p	MIMAT0000727	0.05597742	1.056693102	-0.61466753	0.548257196	0.965790957	-4.908428438
hsa-miR-548ay-3p	MIMAT0025453	0.055946692	0.864790125	-0.606512861	0.553510961	0.96620986	-4.911376625
hsa-mir-6828	MI0022673	0.055845191	1.004834721	-0.540361192	0.59711465	0.972358931	-4.933930191
hsa-mir-326	MI0000808	0.05583102	1.100358555	-0.542086685	0.595955594	0.972081199	-4.933372976
hsa-mir-548w	MI0014222	0.055788408	0.987704167	-0.771943995	0.452491521	0.950829786	-4.844562638
hsa-miR-6735-5p	MIMAT0027371	0.055738403	1.382368422	-0.346875186	0.733645897	0.989764242	-4.985576118
hsa-miR-3913-3p	MIMAT0019225	0.055707166	0.954743777	-0.783808034	0.445712819	0.949212502	-4.839217804
hsa-miR-520b-3p	MIMAT0002843	0.055681191	0.950578796	-0.608972735	0.551923282	0.96620986	-4.910491149
hsa-miR-4279	MIMAT0016909	0.055675982	0.915102026	-0.622161281	0.543453356	0.964741076	-4.905687006
hsa-mir-6769b	MI0022706	0.055675382	1.074617507	-0.568679024	0.57823782	0.967979651	-4.924574133
hsa-mir-6767	MI0022612	0.055672823	1.050560705	-0.598842933	0.558477209	0.96620986	-4.914116149
hsa-mir-7154	MI0023614	0.055646349	1.003288139	-0.790839786	0.441725464	0.947408294	-4.836015949
hsa-mir-124-2	MI0000444	0.055606427	0.868767779	-0.777997798	0.449024567	0.950247229	-4.841844376
hsa-miR-573	MIMAT0003238	0.05557861	0.904812212	-0.640376427	0.531873883	0.960979619	-4.898895361
hsa-mir-6747	MI0022592	0.055558296	0.927999578	-0.637815805	0.533493303	0.960979619	-4.899861033
hsa-mir-4664	MI0017294	0.055555007	0.904484758	-0.6246247	0.541879263	0.963932703	-4.904779097
hsa-miR-544a	MIMAT0003164	0.055528491	0.854692659	-0.759702381	0.459553079	0.951465791	-4.850001693
hsa-mir-6872	MI0022719	0.055511735	1.098527597	-0.640929423	0.531524512	0.960979619	-4.898686345
hsa-mir-6762	MI0022607	0.055449963	0.912840853	-0.653930179	0.523348115	0.960979619	-4.893724624
hsa-miR-4465	MIMAT0018992	0.055283538	0.988443162	-0.435691359	0.669448531	0.984005175	-4.964562385
hsa-miR-142-5p	MIMAT0000433	0.055218134	1.006634563	-0.462764308	0.650371638	0.982649571	-4.957241784
hsa-miR-548az-3p	MIMAT0025457	0.055216716	1.003228467	-0.839049513	0.41500094	0.934266111	-4.813389805
hsa-miR-875-3p	MIMAT0004923	0.055203133	0.913579024	-0.815646531	0.4278401	0.94301493	-4.824519678
hsa-mir-1229	MI0006319	0.054960181	0.93738818	-0.764993251	0.456492704	0.951465791	-4.847660397
hsa-miR-6715b-5p	MIMAT0025842	0.054905669	0.851425619	-0.643145397	0.5301258	0.960979619	-4.897847105
hsa-miR-218-5p	MIMAT0000275	0.054798646	0.816009037	-0.575067379	0.574022524	0.967644566	-4.922401452
hsa-miR-6731-3p	MIMAT0027364	0.054771817	1.147264817	-0.443017839	0.664261893	0.984005175	-4.962623148
hsa-miR-33a-5p	MIMAT0000091	0.054641962	0.854785683	-0.691186104	0.500317552	0.960979619	-4.879001217
hsa-miR-450b-3p	MIMAT0004910	0.054571415	0.763467852	-0.666127345	0.515742419	0.960979619	-4.888986467
hsa-miR-8086	MIMAT0031013	0.054559746	0.913840361	-0.626241149	0.540847741	0.963646638	-4.904181541
hsa-miR-5196-3p	MIMAT0021129	0.054433123	0.964658546	-0.524876026	0.607566831	0.975135504	-4.938855674
hsa-miR-606	MIMAT0003274	0.054415668	1.040900271	-0.603773544	0.555281909	0.96620986	-4.91235877
hsa-mir-6806	MI0022651	0.054407862	0.862793468	-0.637900289	0.533439829	0.960979619	-4.899829229
hsa-miR-550b-3p	MIMAT0018445	0.054237079	0.893983194	-0.613863228	0.548774167	0.965790957	-4.908720845
hsa-miR-552-5p	MIMAT0026615	0.054187714	0.833727617	-0.702097581	0.493686377	0.960979619	-4.874548518
hsa-mir-556	MI0003562	0.054068089	1.075754857	-0.609068324	0.551861636	0.96620986	-4.910456673
hsa-mir-5196	MI0018175	0.053924978	0.860052737	-0.791417137	0.441399082	0.947408294	-4.835751936
hsa-mir-6079	MI0020356	0.053808028	0.959935939	-0.559312872	0.584446954	0.968332399	-4.927718385
hsa-miR-2909	MIMAT0013863	0.05372862	0.837798755	-0.516071244	0.613549902	0.976745744	-4.941595802
hsa-mir-3937	MI0016593	0.053663559	1.054479922	-0.589817086	0.564351939	0.96620986	-4.917298369
hsa-miR-4477a	MIMAT0019004	0.053612018	0.912909852	-0.632689369	0.536743692	0.96159396	-4.901783608
hsa-mir-5195	MI0018174	0.053568469	1.14535481	-0.583681274	0.568364315	0.966625582	-4.919435902
hsa-mir-4709	MI0017342	0.053545813	0.85907564	-0.504763127	0.621275958	0.977648994	-4.945050442
hsa-miR-6786-3p	MIMAT0027473	0.053508809	0.986697554	-0.705931639	0.491368725	0.959845635	-4.872968927
hsa-mir-4780	MI0017424	0.053450225	0.988972357	-0.761875386	0.458294622	0.951465791	-4.849041853
hsa-miR-611	MIMAT0003279	0.053411681	0.998051939	-0.624612147	0.541887278	0.963932703	-4.904783732
hsa-miR-604	MIMAT0003272	0.053378941	0.815122813	-0.695752507	0.497536094	0.960979619	-4.877145486
hsa-miR-513a-5p	MIMAT0002877	0.053331007	0.904870952	-0.559557249	0.584284514	0.968332399	-4.92763697
hsa-miR-1284	MIMAT0005941	0.053174091	0.904477013	-0.678352126	0.508183537	0.960979619	-4.884157231
hsa-mir-1275	MI0006415	0.053145699	0.926387583	-0.606497095	0.553521145	0.96620986	-4.911382289
hsa-miR-4719	MIMAT0019832	0.053107586	0.961904598	-0.872370486	0.397159966	0.930573075	-4.797073605
hsa-mir-3927	MI0016435	0.052979513	0.936803693	-0.529912263	0.604157566	0.975026084	-4.937268619
hsa-mir-4480	MI0016841	0.052816971	1.011540708	-0.556752571	0.586150218	0.968332399	-4.928569349
hsa-mir-4765	MI0017406	0.052771279	0.890923143	-0.693046723	0.499183124	0.960979619	-4.878246424
hsa-miR-510-3p	MIMAT0026613	0.052663052	0.897834391	-0.50647193	0.620105453	0.97746608	-4.944533065
hsa-miR-4653-3p	MIMAT0019719	0.052643509	0.912579072	-0.810379506	0.430764579	0.94301493	-4.826986634
hsa-miR-513b-3p	MIMAT0026749	0.052579996	0.862361423	-0.707275558	0.49055787	0.95960537	-4.872413403
hsa-miR-4520-3p	MIMAT0019057	0.052575592	0.898934196	-0.6628094	0.517805071	0.960979619	-4.890283313
hsa-mir-4298	MI0015830	0.052573128	1.066396018	-0.731598754	0.476020604	0.954010043	-4.862194483
hsa-mir-3140	MI0014163	0.052458889	0.912262039	-0.667386254	0.514961035	0.960979619	-4.888492861
hsa-mir-4672	MI0017303	0.052452768	1.005125989	-0.557646539	0.585555208	0.968332399	-4.928272639
hsa-miR-653-3p	MIMAT0026625	0.05238003	0.816982764	-0.690060892	0.501004332	0.960979619	-4.879456785
hsa-mir-6716	MI0022550	0.052222057	0.885137896	-0.695220396	0.497859741	0.960979619	-4.8773623

hsa-mir-558	MI0003564	0.052090552	0.845263907	-0.775440505	0.450487067	0.950247229	-4.84299494
hsa-mir-3188	MI0014232	0.052081342	0.863813465	-0.760349556	0.459178055	0.951465791	-4.849716084
hsa-miR-1262	MIMAT0005914	0.052066034	0.909317112	-0.746991652	0.466957017	0.951465791	-4.855567161
hsa-mir-4534	MI0016901	0.051998729	1.020941913	-0.469502178	0.645662249	0.982649571	-4.955354078
hsa-mir-1258	MI0006392	0.051971298	1.004471426	-0.999444044	0.333895503	0.930208704	-4.729948923
hsa-miR-4727-3p	MIMAT0019848	0.051947284	1.127516866	-0.513310753	0.61543163	0.976745744	-4.942445839
hsa-mir-548ba	MI0025747	0.051908688	0.870368707	-0.601065249	0.557035807	0.96620986	-4.913325726
hsa-miR-5587-5p	MIMAT0022289	0.051853434	0.991540918	-0.539926178	0.597407038	0.972358931	-4.934070405
hsa-mir-371a	MI0000779	0.051767212	0.897297375	-0.698844421	0.495657946	0.960979619	-4.875882671
hsa-miR-4684-5p	MIMAT0019769	0.051753597	0.841683137	-0.610960969	0.550641823	0.966076637	-4.909773016
hsa-miR-362-5p	MIMAT0000705	0.051749902	1.963514226	-0.213142506	0.834170398	0.996080495	-5.008378368
hsa-mir-7978	MI0025754	0.051629852	0.869646557	-0.545539663	0.593639554	0.970617318	-4.932252871
hsa-miR-198	MIMAT0000228	0.05162343	0.949774961	-0.518396369	0.611967129	0.976338147	-4.940876472
hsa-mir-4279	MI0015887	0.051607744	0.834601654	-0.65646405	0.521762864	0.960979619	-4.892746916
hsa-mir-4277	MI0015886	0.051605957	1.016413455	-0.698106558	0.496105772	0.960979619	-4.876184494
hsa-mir-3115	MI0014127	0.051565447	0.925933751	-0.556124579	0.586568383	0.968332399	-4.928777513
hsa-miR-1233-3p	MIMAT0005588	0.051524952	0.897762062	-0.466984831	0.647419909	0.982649571	-4.956062405
hsa-miR-1279	MIMAT0005937	0.051498471	0.879268612	-0.639538894	0.532403263	0.960979619	-4.899211608
hsa-mir-29c	MI0000735	0.051428681	0.911010162	-0.622128047	0.54347461	0.964741076	-4.905699232
hsa-mir-758	MI0003757	0.051418521	0.83897644	-0.807222377	0.432523687	0.94301493	-4.828458651
hsa-mir-4657	MI0017285	0.051412624	0.938095305	-0.598177876	0.558908956	0.96620986	-4.914352163
hsa-miR-6811-3p	MIMAT0027523	0.051398032	0.853609904	-0.52631898	0.606589058	0.975063724	-4.938402426
hsa-miR-367-5p	MIMAT0004686	0.051377697	0.842309749	-0.67310146	0.511422263	0.960979619	-4.886241267
hsa-miR-300	MIMAT0004903	0.051377001	0.941769088	-0.612343995	0.54975139	0.965790957	-4.909272198
hsa-mir-376b	MI0002466	0.051287834	0.827936218	-0.764119774	0.456997071	0.951465791	-4.848047921
hsa-miR-4493	MIMAT0019028	0.051225902	0.857907637	-0.588892542	0.564955558	0.96620986	-4.917621788
hsa-miR-4768-5p	MIMAT0019920	0.051156457	0.89239643	-0.792352656	0.44087055	0.947337233	-4.835323778
hsa-mir-6885	MI0022732	0.051153686	0.905255704	-0.577250777	0.572585526	0.967644566	-4.921653666
hsa-mir-6727	MI0022572	0.051143439	1.018818967	-0.489111551	0.632046093	0.98055505	-4.949711823
hsa-miR-663b	MIMAT0005867	0.051043318	0.919894142	-0.605284734	0.554304557	0.96620986	-4.911817464
hsa-mir-4422	MI0016759	0.050995137	1.015858809	-0.743517019	0.468993647	0.95151948	-4.857073945
hsa-mir-6782	MI0022627	0.050817704	0.851448743	-0.564993399	0.580677049	0.968332399	-4.92581726
hsa-miR-539-5p	MIMAT0003163	0.050726466	1.070589404	-0.738536384	0.471922466	0.952023826	-4.859222837
hsa-miR-3155a	MIMAT0015029	0.050639585	0.913948837	-0.590183796	0.564112615	0.96620986	-4.917169957
hsa-mir-619	MI0003633	0.050545745	0.861109017	-0.787899253	0.44339014	0.947408294	-4.837357969
hsa-miR-2355-3p	MIMAT0017950	0.050544214	1.141796805	-0.180152093	0.859515698	0.996966918	-5.012345747
hsa-miR-8087	MIMAT0031014	0.050489105	0.835645196	-0.582499166	0.569139058	0.967066304	-4.919845313
hsa-mir-5191	MI0018170	0.050379281	0.985292685	-0.663906562	0.517122482	0.960979619	-4.889855135
hsa-miR-6720-5p	MIMAT0027345	0.050293586	0.925653433	-0.563419836	0.581720089	0.968332399	-4.926345697
hsa-miR-513a-3p	MIMAT0004777	0.050243417	0.94144891	-0.619946086	0.544870984	0.965736099	-4.906500593
hsa-miR-3944-5p	MIMAT0019231	0.050190564	0.973327923	-0.382497251	0.707616289	0.986573137	-4.977704306
hsa-mir-5689	MI0019294	0.050113248	0.893599966	-0.684433868	0.504447097	0.960979619	-4.881724879
hsa-miR-4766-5p	MIMAT0019917	0.049946508	0.912306783	-0.602688036	0.555984528	0.96620986	-4.91274682
hsa-miR-588	MIMAT0003255	0.049901128	0.83670995	-0.417820113	0.682173051	0.985064407	-4.969161887
hsa-mir-101-2	MI0000739	0.049895275	0.908754728	-0.729517344	0.477254312	0.954692455	-4.863081116
hsa-mir-4667	MI0017297	0.04987558	0.942103337	-0.690743523	0.500587618	0.960979619	-4.879180486
hsa-mir-296	MI0000747	0.04984331	0.983163135	-0.494133592	0.628580757	0.978704654	-4.948231387
hsa-mir-6756	MI0022601	0.04972814	1.029291991	-0.575737486	0.573581293	0.967644566	-4.922172231
hsa-mir-6724-1	MI0022559	0.049697207	1.037704302	-0.512389322	0.61606036	0.976745744	-4.942728612
hsa-mir-194-2	MI0000732	0.049503542	1.010126696	-0.636183186	0.534527262	0.960979619	-4.90047487
hsa-miR-105-3p	MIMAT0004516	0.049419884	0.836075706	-0.631602309	0.53743435	0.96159396	-4.902189449
hsa-mir-548h-3	MI0006413	0.049405917	0.984046779	-0.571000287	0.576704295	0.96794959	-4.923787302
hsa-miR-4799-5p	MIMAT0019976	0.049331598	0.968534483	-0.669427034	0.513695801	0.960979619	-4.887690879
hsa-miR-124-5p	MIMAT0004591	0.049305926	0.7217954	-0.656013843	0.522044325	0.960979619	-4.892920884
hsa-miR-302c-5p	MIMAT0000716	0.049268787	0.876982779	-0.647588243	0.527327736	0.960979619	-4.896156464
hsa-mir-4451	MI0016797	0.049074528	0.89886802	-0.603358241	0.555550666	0.96620986	-4.91250731
hsa-mir-3666	MI0016067	0.049049792	0.942730281	-0.539997109	0.597359358	0.972358931	-4.93404755
hsa-miR-6862-5p	MIMAT0027625	0.048941062	1.023765655	-0.527861488	0.605544686	0.975047002	-4.937916605
hsa-mir-4782	MI0017427	0.048876739	0.823436245	-0.762035269	0.458202113	0.951465791	-4.848971134
hsa-miR-587	MIMAT0003253	0.04882432	0.826727123	-0.616899842	0.546823758	0.965790957	-4.907615014
hsa-mir-8086	MI0025922	0.048619945	0.903040541	-0.668322538	0.514380339	0.960979619	-4.8881252
hsa-mir-188	MI0000484	0.048534999	1.100873258	-0.558328237	0.58510169	0.968332399	-4.928046081
hsa-mir-609	MI0003622	0.048418688	0.990467603	-0.51072017	0.617200085	0.97695399	-4.94323962
hsa-mir-6500	MI0022211	0.048413545	0.884667191	-0.543693912	0.594877001	0.971192525	-4.932852445
hsa-miR-4747-3p	MIMAT0019883	0.048408518	0.87293006	-0.428666888	0.674437798	0.984680459	-4.966392426
hsa-mir-5585	MI0019142	0.048384911	0.977271928	-0.470580746	0.64490984	0.982649571	-4.955049476

hsa-miR-4326	MIMAT0016888	0.048373977	0.791374325	-0.756233463	0.461566468	0.951465791	-4.851528889
hsa-miR-5696	MIMAT0022489	0.048356892	0.863810737	-0.613684407	0.548889141	0.965790957	-4.908785808
hsa-mir-3183	MI0014225	0.048336394	0.911004628	-0.531879486	0.602828445	0.974069804	-4.9366448
hsa-miR-643	MIMAT0003313	0.04827475	0.902332166	-0.516157795	0.613490949	0.976745744	-4.941569081
hsa-mir-642a	MI0003657	0.048238588	1.115118088	-0.483594189	0.635863541	0.981968874	-4.95132164
hsa-miR-377-3p	MIMAT0000730	0.048158592	1.042756222	-0.43835373	0.667561722	0.984005175	-4.963861287
hsa-mir-3169	MI0014200	0.048108224	1.000368628	-0.547130126	0.592574306	0.970056246	-4.931734689
hsa-mir-3145	MI0014170	0.048087441	0.870639178	-0.65512107	0.522602725	0.960979619	-4.893265544
hsa-miR-1257	MIMAT0005908	0.048080726	0.977477126	-0.539622229	0.597611376	0.972358931	-4.934168312
hsa-miR-508-3p	MIMAT0002880	0.047990185	0.931126754	-0.574600948	0.574329748	0.967644566	-4.922560855
hsa-mir-6728	MI0022573	0.047946869	0.980764524	-0.857198523	0.405219269	0.930588488	-4.804570748
hsa-miR-6830-5p	MIMAT0027560	0.047928715	1.197463544	-0.432302782	0.671853339	0.984005175	-4.965448769
hsa-miR-675-3p	MIMAT0006790	0.047857822	1.042027284	-0.573820604	0.574843932	0.967644566	-4.922827267
hsa-miR-4757-5p	MIMAT0019901	0.04784838	0.85016653	-0.431679274	0.672296236	0.984005175	-4.965611139
hsa-mir-33a	MI0000091	0.047792728	0.875639877	-0.700657888	0.494558326	0.960979619	-4.875139643
hsa-mir-1269a	MI0006406	0.047772369	0.972586033	-0.659388345	0.519936754	0.960979619	-4.891614248
hsa-miR-6735-3p	MIMAT0027372	0.047758211	0.933175905	-0.80159968	0.435667941	0.944812484	-4.831067755
hsa-miR-372-5p	MIMAT0026484	0.047610659	0.867180701	-0.556750873	0.586151348	0.968332399	-4.928569912
hsa-miR-4264	MIMAT0016899	0.047586217	0.902731446	-0.426451326	0.676014748	0.985055481	-4.966963682
hsa-miR-6070	MIMAT0023695	0.047564615	0.754252588	-0.6267469	0.540525224	0.963646638	-4.903994285
hsa-mir-4729	MI0017366	0.047293174	0.889588547	-0.504563467	0.621412792	0.977648994	-4.945110784
hsa-miR-4999-5p	MIMAT0021017	0.047262187	0.781667744	-0.575444702	0.573774054	0.967644566	-4.922272413
hsa-miR-3191-3p	MIMAT0015075	0.047207267	1.036723164	-0.560225678	0.583840319	0.968332399	-4.927414109
hsa-mir-374c	MI0016684	0.047144506	0.936398957	-0.601918658	0.556482815	0.96620986	-4.913021466
hsa-mir-6811	MI0022656	0.047060489	0.936199132	-0.492098078	0.629984229	0.97971495	-4.948833169
hsa-miR-500b-3p	MIMAT0027032	0.047038457	1.078255243	-0.654635029	0.52290687	0.960979619	-4.893453002
hsa-miR-5195-3p	MIMAT0021127	0.04701829	1.55411907	-0.24910994	0.806754404	0.994603723	-5.003301928
hsa-mir-4528	MI0016895	0.046954994	0.949938162	-0.562884021	0.582075477	0.968332399	-4.92652532
hsa-mir-6889	MI0022736	0.046940312	0.90193477	-0.650450257	0.525529685	0.960979619	-4.895061711
hsa-miR-619-3p	MIMAT0003288	0.04690487	1.011501363	-0.516244028	0.613432215	0.976745744	-4.941542453
hsa-mir-454	MI0003820	0.046904869	0.893225301	-0.709600771	0.48915683	0.959021141	-4.871449994
hsa-mir-6792	MI0022637	0.046884706	1.17612251	-0.318801301	0.754403187	0.992853492	-4.991250449
hsa-mir-586	MI0003594	0.046821664	0.908184874	-0.627324354	0.540157112	0.963391053	-4.903780311
hsa-mir-3199-1	MI0014247	0.046818128	0.907031145	-0.713722884	0.486678947	0.958122977	-4.869735048
hsa-miR-4477b	MIMAT0019005	0.04681154	0.973882759	-0.596904229	0.559736294	0.96620986	-4.914803471
hsa-miR-6788-3p	MIMAT0027477	0.046685757	1.083909167	-0.39250981	0.700365787	0.98572659	-4.975357132
hsa-mir-6132	MI0021277	0.046635477	0.847915399	-0.652741166	0.524092931	0.960979619	-4.894182214
hsa-miR-3150a-3p	MIMAT0015023	0.046561824	0.900143626	-0.687477097	0.502583457	0.960979619	-4.880500337
hsa-mir-5787	MI0019797	0.046545228	0.955036681	-0.487781341	0.632965475	0.98055505	-4.950101536
hsa-mir-4651	MI0017279	0.046490636	0.841083939	-0.568805879	0.578153959	0.967979651	-4.924531211
hsa-mir-5583-1	MI0019139	0.046454087	0.891077763	-0.597213859	0.559535104	0.96620986	-4.914693838
hsa-mir-618	MI0003632	0.046436925	0.894967977	-0.548439637	0.591697961	0.970056246	-4.931306975
hsa-miR-653-5p	MIMAT0003328	0.046421542	0.79058954	-0.676293081	0.509452182	0.960979619	-4.884976246
hsa-mir-1202	MI0006334	0.046373391	0.890189719	-0.533756032	0.601561945	0.973554267	-4.936047698
hsa-mir-6508	MI0022220	0.046281723	0.977764864	-0.836419611	0.416431071	0.934671969	-4.814654176
hsa-mir-4703	MI0017336	0.046173244	0.933277088	-0.5479692	0.592012709	0.970056246	-4.931460741
hsa-miR-6822-5p	MIMAT0027544	0.046129157	0.85726241	-0.438017359	0.667799979	0.984005175	-4.963950092
hsa-miR-6080	MIMAT0023705	0.046024489	0.845360847	-0.575797653	0.573541685	0.967644566	-4.922151638
hsa-mir-4321	MI0015852	0.045927899	1.073485403	-0.354478022	0.728060563	0.989374359	-4.983958953
hsa-mir-23b	MI0000439	0.045748089	0.904556267	-0.581976349	0.569481886	0.96749112	-4.920026138
hsa-mir-138-1	MI0000476	0.045712651	0.893774401	-0.719111099	0.483451322	0.957574873	-4.867479839
hsa-mir-4504	MI0016867	0.04569929	0.933583166	-0.486225485	0.634041611	0.98055505	-4.950556073
hsa-miR-526b-3p	MIMAT0002836	0.045645078	0.894209869	-0.622859516	0.543006938	0.964741076	-4.905430004
hsa-mir-323b	MI0014206	0.045620938	0.924083345	-0.611164973	0.55051043	0.966076637	-4.909699209
hsa-mir-422a	MI0001444	0.045572424	0.908690168	-0.579058849	0.57139698	0.967644566	-4.921032416
hsa-mir-4699	MI0017332	0.045543172	0.953375424	-0.559273686	0.584473004	0.968332399	-4.927731437
hsa-mir-6746	MI0022591	0.045474255	1.132666311	-0.400949384	0.694277677	0.98554565	-4.973333016
hsa-miR-203b-5p	MIMAT0019813	0.045459092	0.812710357	-0.4810181	0.637649609	0.982649571	-4.952067302
hsa-miR-548c-3p	MIMAT0003285	0.045314625	0.955438402	-0.596182732	0.560205257	0.96620986	-4.91505873
hsa-mir-2277	MI0011284	0.045201783	1.092221045	-0.494572608	0.628278253	0.978652564	-4.948101286
hsa-mir-4512	MI0016878	0.045186137	0.828863874	-0.745936667	0.467574814	0.951465791	-4.856025322
hsa-mir-1322	MI0006653	0.045058582	1.120213469	-0.473134527	0.643129927	0.982649571	-4.954325588
hsa-mir-6075	MI0020352	0.044864884	1.054986458	-0.382992976	0.707256617	0.986573137	-4.977589483
hsa-mir-4498	MI0016860	0.04481987	0.874875595	-0.577782724	0.572235714	0.967644566	-4.921471079
hsa-mir-4472-1	MI0016823	0.044815896	1.072918962	-0.636422865	0.5343754	0.960979619	-4.900384846
hsa-miR-3132	MIMAT0014997	0.044753564	1.034026714	-0.425396752	0.676765905	0.985064407	-4.967234588

hsa-mir-3138	MI0014161	0.044693811	1.030424398	-0.51518724	0.614152191	0.976745744	-4.941868484
hsa-mir-8063	MI0025899	0.044447509	0.960138242	-0.455072399	0.655766773	0.984005175	-4.95936479
hsa-miR-6891-5p	MIMAT0027682	0.044323859	5.149999044	-0.126418269	0.901129459	0.999227185	-5.017390044
hsa-mir-5687	MI0019291	0.044299645	0.879382784	-0.562880263	0.582077969	0.968332399	-4.926526579
hsa-mir-4654	MI0017282	0.044237149	0.934356902	-0.386004407	0.705073232	0.986573137	-4.976888849
hsa-mir-6510	MI0022222	0.044037907	0.917718672	-0.465634592	0.648363568	0.982649571	-4.956440831
hsa-mir-4535	MI0016903	0.043916592	0.962113922	-0.51214348	0.616228161	0.976745744	-4.942803975
hsa-miR-204-3p	MIMAT0022693	0.04384782	1.056590447	-0.444234259	0.663402462	0.984005175	-4.962298164
hsa-mir-1471	MI0007076	0.043750934	0.943679305	-0.675675074	0.509833314	0.960979619	-4.885221624
hsa-mir-1976	MI0009986	0.043746963	1.032069386	-0.391441862	0.70113771	0.98572659	-4.975610287
hsa-miR-5582-5p	MIMAT0022279	0.04367171	0.913767984	-0.402272368	0.693325269	0.98554565	-4.973011934
hsa-mir-4684	MI0017316	0.043541397	1.035149415	-0.56297567	0.582014681	0.968332399	-4.926494608
hsa-miR-337-3p	MIMAT0000754	0.043508745	1.143572702	-0.400089492	0.694896994	0.98554565	-4.973541159
hsa-mir-6073	MI0020350	0.043502925	0.952606546	-0.594402781	0.5613631	0.96620986	-4.915687232
hsa-miR-650	MIMAT0003320	0.043473746	1.08485054	-0.365084293	0.720295462	0.988545996	-4.981645827
hsa-mir-7849	MI0025519	0.043268348	0.85314909	-0.480893392	0.637736132	0.982649571	-4.952103302
hsa-miR-2052	MIMAT0009977	0.043198437	0.813723287	-0.723371076	0.480908645	0.957366263	-4.865686025
hsa-mir-513b	MI0006648	0.043047811	0.86017778	-0.570126363	0.577281397	0.967979651	-4.924083886
hsa-miR-4451	MIMAT0018973	0.043038956	1.055622489	-0.413982948	0.684918406	0.98554565	-4.970125195
hsa-mir-4763	MI0017404	0.043029646	0.922312281	-0.567088378	0.579289891	0.967979651	-4.925111573
hsa-mir-6085	MI0020362	0.04297693	0.977777763	-0.680215599	0.507036972	0.960979619	-4.883414051
hsa-mir-4697	MI0017330	0.042971843	0.960358723	-0.40203326	0.693497362	0.98554565	-4.97307004
hsa-miR-8082	MIMAT0031009	0.042870043	0.793196549	-0.518078549	0.612183359	0.976534918	-4.940974978
hsa-miR-8068	MIMAT0030995	0.042835272	0.883201047	-0.465696562	0.648320245	0.982649571	-4.956423485
hsa-miR-34b-5p	MIMAT0000685	0.042835246	0.880979024	-0.591988196	0.562935809	0.96620986	-4.916537022
hsa-miR-4531	MIMAT0019070	0.04283507	0.917043434	-0.541823632	0.59613222	0.972194814	-4.933458032
hsa-miR-1260a	MIMAT0005911	0.042699048	1.1366907	-0.747210725	0.466828791	0.951465791	-4.855471949
hsa-mir-3165	MI0014195	0.042526006	0.844854319	-0.630008527	0.538447842	0.961766436	-4.902783303
hsa-mir-6830	MI0022675	0.042491449	0.885652399	-0.622871546	0.542999248	0.964741076	-4.905425574
hsa-mir-8085	MI0025921	0.042483567	0.928050266	-0.622264252	0.543387509	0.964741076	-4.905649122
hsa-miR-6729-3p	MIMAT0027360	0.042397911	1.17578218	-0.445684984	0.662378129	0.984005175	-4.961909461
hsa-mir-4731	MI0017368	0.042313978	0.941306809	-0.448623861	0.660305175	0.984005175	-4.961118292
hsa-mir-4733	MI0017370	0.042199423	1.052398198	-0.415015002	0.684179553	0.98554565	-4.969866946
hsa-mir-1236	MI0006326	0.04215575	0.875150267	-0.444290408	0.663362803	0.984005175	-4.962283142
hsa-mir-4686	MI0017318	0.042048432	0.998035128	-0.433017769	0.671345616	0.984005175	-4.965262298
hsa-mir-8079	MI0025915	0.041981326	1.04112404	-0.535034663	0.600699746	0.973257028	-4.93563971
hsa-mir-539	MI0003514	0.041900118	0.932290409	-0.589907211	0.564293116	0.96620986	-4.917266816
hsa-mir-3194	MI0014239	0.041891436	0.911424911	-0.441660828	0.665221232	0.984005175	-4.962984682
hsa-miR-6871-3p	MIMAT0027643	0.041863921	0.920634995	-0.682428983	0.505677066	0.960979619	-4.882528905
hsa-miR-6738-5p	MIMAT0027377	0.041752352	1.085386242	-0.349756677	0.731527188	0.989764242	-4.984967236
hsa-miR-4515	MIMAT0019052	0.041672754	0.948830947	-0.417621213	0.682315243	0.985064407	-4.969212031
hsa-miR-942-3p	MIMAT0026734	0.04162352	0.910933389	-0.472773102	0.643381693	0.982649571	-4.954428265
hsa-mir-4722	MI0017357	0.041587332	1.608257222	-0.166210619	0.870276482	0.997482768	-5.013823546
hsa-mir-6749	MI0022594	0.041493318	0.847294403	-0.568890159	0.578098248	0.967979651	-4.924502689
hsa-mir-4510	MI0016876	0.041374334	0.979984753	-0.586435361	0.566561477	0.96620986	-4.918479042
hsa-miR-518e-3p	MIMAT0002861	0.041170224	0.925193767	-0.450306683	0.659119483	0.984005175	-4.960663013
hsa-mir-4320	MI0015849	0.041096488	0.85624824	-0.570200009	0.577232753	0.967979651	-4.924058909
hsa-miR-3670	MIMAT0018093	0.041076204	0.84179195	-0.867064013	0.399966525	0.930573075	-4.799708621
hsa-miR-4676-3p	MIMAT0019759	0.041068744	0.744052773	-0.570030566	0.577344676	0.967979651	-4.924116371
hsa-mir-646	MI0003661	0.041036199	0.981558262	-0.545273055	0.593818216	0.970641798	-4.932339594
hsa-mir-489	MI0003124	0.04099162	0.862271518	-0.595376775	0.560729366	0.96620986	-4.915343531
hsa-miR-4740-5p	MIMAT0019869	0.040926353	0.940898658	-0.470881369	0.644700197	0.982649571	-4.954964457
hsa-miR-1272	MIMAT0005925	0.040898696	0.903356941	-0.469295397	0.645806544	0.982649571	-4.955412399
hsa-miR-152-5p	MIMAT0026479	0.04089708	0.850686639	-0.597298573	0.559480065	0.96620986	-4.914663833
hsa-miR-1914-3p	MIMAT0007890	0.040855539	1.109154556	-0.366028515	0.719605704	0.98842922	-4.98143668
hsa-miR-4433b-5p	MIMAT0030413	0.040745687	1.490534592	-0.357309279	0.725984669	0.989104742	-4.983347988
hsa-miR-4643	MIMAT0019703	0.040738206	0.88179098	-0.526777693	0.606278388	0.975047002	-4.938258093
hsa-mir-4647	MI0017274	0.040710751	1.197279601	-0.429407474	0.673911031	0.984515661	-4.966200838
hsa-mir-1266	MI0006403	0.040642935	0.93600423	-0.547682642	0.592204474	0.970056246	-4.931554343
hsa-mir-1538	MI0007259	0.040569542	0.826633695	-0.587916337	0.565593278	0.96620986	-4.917962764
hsa-mir-502	MI0003186	0.040538175	0.797498131	-0.485358192	0.634641861	0.980972044	-4.950808847
hsa-mir-1203	MI0006335	0.040415778	0.85941787	-0.352578482	0.729454557	0.989764242	-4.984366202
hsa-miR-625-3p	MIMAT0004808	0.040323141	0.911983644	-0.33983343	0.738832991	0.989829051	-4.987043387
hsa-mir-139	MI0000261	0.040296137	3.359444644	-0.201773786	0.842884606	0.996080495	-5.009820134
hsa-mir-3193	MI0014238	0.040229134	0.981786432	-0.551949638	0.589352263	0.969049836	-4.930155785
hsa-mir-620	MI0003634	0.040209108	1.014064823	-0.455390443	0.655543299	0.984005175	-4.959277685

hsa-mir-6834	MI0022679	0.040032946	0.9012942	-0.595266824	0.560800888	0.96620986	-4.915382357
hsa-miR-3936	MIMAT0018351	0.039970437	1.170727546	-0.292958573	0.773686254	0.994214304	-4.996058812
hsa-miR-6500-3p	MIMAT0025455	0.039894181	0.977696357	-0.521082636	0.610141007	0.97572307	-4.940041598
hsa-mir-1911	MI0008332	0.039859222	0.960231248	-0.413944792	0.684945728	0.98554565	-4.970134731
hsa-mir-378j	MI0021273	0.039827443	0.801842246	-0.450370756	0.659074357	0.984005175	-4.960645646
hsa-mir-626	MI0003640	0.039807973	0.811847594	-0.514097979	0.614894721	0.976745744	-4.94220387
hsa-mir-3149	MI0014176	0.039579934	0.95715905	-0.544215025	0.594527499	0.970952097	-4.932683361
hsa-mir-30c-2	MI0000254	0.039501361	0.96929122	-0.670536877	0.513008477	0.960979619	-4.887253795
hsa-mir-3119-2	MI0014135	0.03949456	0.959669883	-0.509716175	0.617886122	0.97718859	-4.943546228
hsa-mir-943	MI0005768	0.03944366	0.845398967	-0.640136444	0.532025539	0.960979619	-4.898986016
hsa-mir-154	MI0000480	0.039362036	1.003235154	-0.464176886	0.649383038	0.982649571	-4.956848197
hsa-mir-6126	MI0021260	0.039347937	1.103388985	-0.42913921	0.674101822	0.984627063	-4.966270274
hsa-miR-2682-3p	MIMAT0013518	0.039272519	0.737649489	-0.517507466	0.612571992	0.976708039	-4.941151836
hsa-mir-191	MI0000465	0.039165596	0.937636357	-0.448776148	0.660197838	0.984005175	-4.961077159
hsa-mir-3185	MI0014227	0.039088073	0.990769664	-0.458849113	0.653115264	0.983035193	-4.958326663
hsa-let-7a-3p	MIMAT0004481	0.039060621	0.89257339	-0.398008597	0.696396643	0.985651988	-4.974043062
hsa-mir-548m	MI0006400	0.039049746	1.086874527	-0.366333634	0.719382867	0.98842922	-4.981368983
hsa-mir-548aw	MI0019283	0.038999762	0.970816478	-0.420175191	0.680490374	0.985064407	-4.968566399
hsa-mir-1206	MI0006339	0.038958375	0.910889252	-0.486244391	0.634028529	0.98055505	-4.950550558
hsa-miR-4685-3p	MIMAT0019772	0.03888417	0.990661283	-0.301525669	0.767275886	0.994214304	-4.994509002
hsa-miR-3117-5p	MIMAT0019197	0.038874305	0.96817014	-0.371362118	0.715714238	0.987260208	-4.980245401
hsa-mir-4801	MI0017449	0.038838606	0.887173531	-0.599272154	0.558198658	0.96620986	-4.913963698
hsa-mir-4678	MI0017309	0.038810538	1.015756743	-0.400805761	0.694381103	0.98554565	-4.973367812
hsa-miR-548x-3p	MIMAT0015081	0.038571586	0.933784613	-0.391919777	0.700792226	0.98572659	-4.975497081
hsa-miR-4697-5p	MIMAT0019791	0.038479932	0.90141847	-0.387444999	0.704029704	0.986573137	-4.976551799
hsa-let-7c-3p	MIMAT0026472	0.03838767	0.884333528	-0.468182497	0.646583403	0.982649571	-4.955725863
hsa-let-7g-3p	MIMAT0004584	0.038336346	0.915975997	-0.512459788	0.616012268	0.976745744	-4.942707004
hsa-mir-4680	MI0017312	0.038051284	0.861681325	-0.413704776	0.685117608	0.98554565	-4.970194696
hsa-miR-874-5p	MIMAT0026718	0.038045649	1.003941717	-0.495218019	0.627833657	0.978652564	-4.94790982
hsa-miR-4763-5p	MIMAT0019912	0.037991849	1.057772284	-0.397483846	0.696775025	0.985651988	-4.974169229
hsa-mir-3151	MI0014178	0.037924584	0.922995905	-0.471879315	0.644004492	0.982649571	-4.954681856
hsa-mir-583	MI0003590	0.037913493	0.935509585	-0.39252444	0.700355215	0.98572659	-4.975353659
hsa-mir-4658	MI0017286	0.037870872	0.986273219	-0.430133028	0.673395126	0.984315762	-4.96601283
hsa-mir-6855	MI0022701	0.037845378	1.011128299	-0.384115363	0.706442537	0.986573137	-4.977328973
hsa-miR-4731-3p	MIMAT0019854	0.037791647	0.838314736	-0.453701655	0.656730319	0.984005175	-4.959739537
hsa-miR-4447	MIMAT0018966	0.037726144	0.878383913	-0.378914513	0.71021788	0.9869374	-4.97852987
hsa-miR-3684	MIMAT0018112	0.037703684	0.841233578	-0.502736118	0.622665804	0.978294147	-4.945662006
hsa-miR-1913	MIMAT0007888	0.037685246	0.970523503	-0.372220001	0.715089074	0.987260208	-4.980052224
hsa-miR-5707	MIMAT0022501	0.037651106	0.878650929	-0.466723715	0.64760235	0.982649571	-4.956135669
hsa-miR-452-3p	MIMAT0001636	0.037603239	0.851165409	-0.450286306	0.659133835	0.984005175	-4.960668536
hsa-miR-6508-5p	MIMAT0025472	0.037535204	1.046185101	-0.278828635	0.784296224	0.994214304	-4.998518941
hsa-miR-200a-3p	MIMAT0000682	0.037478367	0.892571064	-0.462373832	0.650645035	0.982649571	-4.957350379
hsa-mir-1205	MI0006338	0.037344812	0.872810495	-0.549337029	0.591097792	0.970056246	-4.931013312
hsa-miR-6809-3p	MIMAT0027519	0.03734472	0.814100187	-0.495807314	0.627427848	0.978652564	-4.947734793
hsa-miR-4716-3p	MIMAT0019827	0.037326576	1.07146079	-0.418194807	0.681905219	0.985064407	-4.96906736
hsa-mir-103b-1	MI0007261	0.037322901	0.761998341	-0.482176811	0.636845956	0.982359276	-4.951732378
hsa-mir-759	MI0004065	0.037311366	0.93069178	-0.51705083	0.612882829	0.976745744	-4.941293119
hsa-miR-335-3p	MIMAT0004703	0.037180326	0.946824731	-0.334539609	0.742741212	0.990306303	-4.988127072
hsa-miR-4527	MIMAT0019066	0.037173444	0.895452748	-0.485601299	0.634473581	0.980803444	-4.950738037
hsa-miR-4426	MIMAT0018941	0.03714477	0.90815652	-0.464224259	0.649349895	0.982649571	-4.956834977
hsa-mir-6738	MI0022583	0.037143835	0.923173353	-0.578816681	0.571556096	0.967644566	-4.92111573
hsa-mir-3974	MI0016992	0.037074587	0.891930454	-0.577747629	0.572258789	0.967644566	-4.92148313
hsa-mir-7151	MI0023611	0.037059529	0.898654635	-0.439832652	0.66651462	0.984005175	-4.963470056
hsa-mir-203a	MI0000283	0.036955828	1.193672866	-0.296067222	0.771358191	0.994214304	-4.995501525
hsa-miR-4745-3p	MIMAT0019879	0.036750615	0.894473375	-0.487212756	0.633358648	0.98055505	-4.950267807
hsa-miR-4743-3p	MIMAT0022978	0.036743443	0.809044838	-0.777232013	0.449462202	0.950247229	-4.842189266
hsa-mir-3613	MI0016003	0.036742156	0.774196278	-0.360336579	0.723767494	0.989002954	-4.982689476
hsa-mir-24-2	MI0000081	0.036734795	0.865332705	-0.397752288	0.69658145	0.985651988	-4.974104707
hsa-miR-4307	MIMAT0016860	0.036722598	0.910612791	-0.363665141	0.721332632	0.988653693	-4.981959181
hsa-mir-7847	MI0025517	0.036651464	0.916100313	-0.43077986	0.67293534	0.984005175	-4.965844962
hsa-miR-320d	MIMAT0006764	0.036572148	9.910069861	-0.149053694	0.883555447	0.998806144	-5.015479724
hsa-miR-3916	MIMAT0018190	0.036533803	1.114037373	-0.268735231	0.7919026	0.994214304	-5.000202946
hsa-mir-149	MI0000478	0.036375129	0.928346657	-0.358022591	0.725462014	0.989104742	-4.983193313
hsa-miR-518f-3p	MIMAT0002842	0.036325995	0.874820032	-0.418418536	0.681745318	0.985064407	-4.96901088
hsa-mir-3154	MI0014182	0.036311814	1.271088729	-0.485813173	0.634326937	0.980720757	-4.950676296
hsa-miR-151a-5p	MIMAT0004697	0.036220197	10.96245512	-0.277620286	0.785205651	0.994214304	-4.998723767

hsa-miR-3191-5p	MIMAT0022732	0.036190164	0.800479041	-0.57325919	0.575214006	0.967644566	-4.923018725
hsa-mir-208a	MI0000251	0.036143254	0.88272608	-0.701602791	0.493985943	0.960979619	-4.874751798
hsa-miR-153-5p	MIMAT0026480	0.036135425	0.903622205	-0.399754024	0.695138669	0.98554565	-4.973622244
hsa-miR-3972	MIMAT0019357	0.035868487	0.868531115	-0.395266456	0.69837484	0.985665229	-4.974700579
hsa-miR-6853-3p	MIMAT0027607	0.035736446	0.854629339	-0.348943209	0.732125088	0.989764242	-4.985139626
hsa-mir-4752	MI0017391	0.035733303	0.884371289	-0.495438253	0.627681982	0.978652564	-4.947844431
hsa-miR-509-5p	MIMAT0004779	0.035713473	0.784943182	-0.474941804	0.641871672	0.982649571	-4.953811038
hsa-miR-5695	MIMAT0022488	0.035649827	0.970054719	-0.475012206	0.64182268	0.982649571	-4.953790956
hsa-miR-551a	MIMAT0003214	0.035552074	0.987093438	-0.506391841	0.620160289	0.97746608	-4.944557351
hsa-miR-4661-5p	MIMAT0019729	0.03548329	0.97320909	-0.492547259	0.629674395	0.979484522	-4.948700576
hsa-miR-4772-3p	MIMAT0019927	0.035479651	0.718066343	-0.400487914	0.694610012	0.98554565	-4.973444772
hsa-miR-5692b	MIMAT0022497	0.035181	1.000773995	-0.3807642	0.708874268	0.986821593	-4.978104592
hsa-miR-19b-3p	MIMAT0000074	0.035152841	9.097439728	-0.328903056	0.746910532	0.990306303	-4.98926262
hsa-miR-95-5p	MIMAT0026473	0.035131244	0.928028686	-0.449489635	0.659695045	0.984005175	-4.960884266
hsa-miR-7162-5p	MIMAT0028234	0.035091785	0.780638507	-0.35268501	0.729376354	0.989764242	-4.98434342
hsa-miR-4633-3p	MIMAT0019690	0.034745392	0.869917871	-0.33039832	0.745803691	0.990306303	-4.988963222
hsa-miR-3201	MIMAT0015086	0.034711336	1.061662445	-0.336455328	0.741326058	0.990306303	-4.987736831
hsa-miR-524-3p	MIMAT0002850	0.03460957	0.867217038	-0.386187217	0.704940775	0.986573137	-4.976846145
hsa-miR-372-3p	MIMAT0000724	0.034543779	0.862363797	-0.451287284	0.658429002	0.984005175	-4.960396961
hsa-miR-3682-5p	MIMAT0019222	0.034542314	0.773668985	-0.346026642	0.734270244	0.989764242	-4.985754484
hsa-mir-31	MI0000089	0.034527009	0.949615251	-0.374540606	0.713399043	0.9869374	-4.9795275
hsa-mir-6891	MI0022738	0.034464309	1.195353978	-0.252229174	0.804388542	0.99449721	-5.002824877
hsa-miR-146a-3p	MIMAT0004608	0.034440837	0.939085076	-0.323056323	0.751243992	0.991956125	-4.990420548
hsa-mir-4802	MI0017450	0.0344369	0.841169417	-0.429983805	0.673501217	0.984331059	-4.966051522
hsa-mir-133b	MI0000822	0.034400889	0.845516805	-0.398618123	0.695957237	0.985651988	-4.973896309
hsa-miR-132-5p	MIMAT0004594	0.034359577	0.787851099	-0.56008334	0.583934893	0.968332399	-4.927461587
hsa-miR-5197-3p	MIMAT0021131	0.034326249	1.110421913	-0.354990794	0.727684432	0.989333548	-4.983848652
hsa-miR-4296	MIMAT0016845	0.03419727	1.02901909	-0.323581448	0.750854424	0.991817554	-4.99031738
hsa-mir-1302-4	MI0006365	0.034188842	0.930010239	-0.450020201	0.659321268	0.984005175	-4.960740635
hsa-mir-6809	MI0022654	0.03416542	1.018357093	-0.393382566	0.699735205	0.98572659	-4.975149749
hsa-miR-613	MIMAT0003281	0.034126148	0.835165778	-0.472154573	0.64381266	0.982649571	-4.954603807
hsa-mir-181a-2	MI0000269	0.033794024	1.047244209	-0.380021018	0.709413995	0.986886843	-4.978275706
hsa-mir-648	MI0003663	0.033780186	0.915723242	-0.438748921	0.66728185	0.984005175	-4.963756868
hsa-miR-3928-5p	MIMAT0027037	0.033599035	0.961976662	-0.332482314	0.74426202	0.990306303	-4.988543726
hsa-mir-3175	MI0014209	0.03354326	1.028840631	-0.322067862	0.751977479	0.992075387	-4.990614299
hsa-miR-7849-3p	MIMAT0030424	0.033523714	0.863348501	-0.433746352	0.670828409	0.984005175	-4.965071975
hsa-mir-4689	MI0017322	0.033467321	0.869943757	-0.428504892	0.674553047	0.984680459	-4.966434292
hsa-mir-3202-2	MI0014253	0.033457856	0.898103566	-0.361636359	0.722816328	0.988677831	-4.98240508
hsa-mir-2909	MI0013083	0.033349512	0.960441719	-0.389216789	0.70274711	0.986486204	-4.976135589
hsa-mir-1238	MI0006328	0.033220591	0.949389654	-0.547918803	0.592046432	0.970056246	-4.931477206
hsa-miR-219a-5p	MIMAT0000276	0.033211664	0.853678942	-0.374520491	0.713413686	0.9869374	-4.979532062
hsa-miR-924	MIMAT0004974	0.033187894	1.13196975	-0.223049693	0.826594744	0.996080495	-5.007058108
hsa-miR-548a-3p	MIMAT0003251	0.033184142	0.985409966	-0.29861488	0.76945195	0.994214304	-4.995040495
hsa-mir-16-1	MI0000070	0.033170967	1.06492425	-0.515246622	0.614111724	0.976745744	-4.941850181
hsa-miR-596	MIMAT0003264	0.032958394	0.957466963	-0.303861699	0.76553097	0.993908457	-4.994078794
hsa-miR-6856-3p	MIMAT0027613	0.032935986	0.862458675	-0.354420031	0.728103106	0.989374359	-4.983971418
hsa-miR-6834-3p	MIMAT0027569	0.032922613	1.082948378	-0.39659994	0.697412573	0.985651988	-4.974381384
hsa-miR-374b-3p	MIMAT0004956	0.032850222	0.807600057	-0.356153786	0.726831616	0.989139314	-4.983597908
hsa-mir-4317	MI0015850	0.032709657	0.838549718	-0.457193409	0.654277087	0.983702178	-4.958782789
hsa-mir-573	MI0003580	0.032544407	0.952505515	-0.4013114	0.694017011	0.98554565	-4.973245258
hsa-miR-1258	MIMAT0005909	0.032522332	0.907611225	-0.395732324	0.6980386	0.985651988	-4.974589183
hsa-mir-6772	MI0022617	0.03250957	0.725013917	-0.437410409	0.668229984	0.984005175	-4.964110168
hsa-mir-661	MI0003669	0.032447814	0.962431795	-0.400720618	0.694442418	0.98554565	-4.973388433
hsa-mir-301b	MI0005568	0.032365285	1.006764459	-0.309157402	0.761580185	0.99324537	-4.993091456
hsa-miR-520d-3p	MIMAT0002856	0.032346535	0.882288045	-0.4743168	0.642306682	0.982649571	-4.953989195
hsa-mir-1255b-2	MI0006436	0.032307418	0.92098746	-0.374030587	0.713770343	0.9869374	-4.979643095
hsa-mir-2861	MI0013006	0.032080592	1.107659874	-0.25742038	0.800455542	0.994214304	-5.002017947
hsa-miR-15a-3p	MIMAT0004488	0.032027782	0.940040825	-0.356070422	0.726892733	0.989139314	-4.983615908
hsa-miR-548j-3p	MIMAT0026737	0.03194832	0.957203983	-0.366770286	0.719064014	0.988322209	-4.981272008
hsa-mir-219b	MI0017299	0.031940078	0.869659403	-0.404147159	0.691976536	0.98554565	-4.972555176
hsa-mir-365b	MI0000769	0.031882745	0.960154953	-0.399145624	0.695577053	0.985651988	-4.97376913
hsa-mir-6835	MI0022680	0.031741227	0.841818224	-0.461687559	0.651125665	0.982649571	-4.957541026
hsa-mir-3664	MI0016065	0.031644391	0.91728553	-0.299286551	0.768949639	0.994214304	-4.994918302
hsa-miR-7705	MIMAT0030020	0.03155577	0.993333645	-0.328338442	0.747328628	0.990471312	-4.989375327
hsa-miR-361-5p	MIMAT0000703	0.031506791	8.28830138	-0.199518334	0.844615999	0.996080495	-5.010096851
hsa-mir-6134	MI0021279	0.031446028	0.909774619	-0.381671123	0.708215845	0.986617	-4.977895339

hsa-mir-4803	MI0017451	0.031416735	0.926787974	-0.335529534	0.742009827	0.990306303	-4.987925692
hsa-mir-1204	MI0006337	0.031401348	0.889846579	-0.364090328	0.72102183	0.988653693	-4.981865423
hsa-miR-138-5p	MIMAT0000430	0.031263145	0.883155565	-0.361999388	0.722550752	0.988653693	-4.98232547
hsa-miR-5698	MIMAT0022491	0.031221703	1.021333981	-0.326702464	0.74854053	0.990697422	-4.989700827
hsa-miR-576-5p	MIMAT0003241	0.031217598	0.961784526	-0.350087058	0.731284409	0.989764242	-4.98489711
hsa-mir-4295	MI0015822	0.031129707	0.936419241	-0.461731597	0.651094818	0.982649571	-4.9575288
hsa-mir-4524b	MI0019114	0.031043141	1.00939347	-0.381802957	0.708120155	0.986573137	-4.977864881
hsa-mir-4487	MI0016848	0.031019628	0.983013715	-0.474390716	0.642255228	0.982649571	-4.953968137
hsa-mir-4290	MI0015899	0.030963418	1.040534302	-0.338068841	0.740134896	0.990306303	-4.98740646
hsa-miR-134-3p	MIMAT0026481	0.030936899	0.891348813	-0.423300334	0.678260209	0.985064407	-4.967771208
hsa-mir-4477b	MI0016830	0.030856366	0.95057037	-0.383221136	0.7070911	0.986573137	-4.977536586
hsa-miR-561-5p	MIMAT0022706	0.030775996	1.031081905	-0.361058611	0.72323906	0.988677831	-4.982531616
hsa-mir-541	MI0005539	0.030759041	0.980412017	-0.326896289	0.748396912	0.990697422	-4.989662346
hsa-miR-935	MIMAT0004978	0.03070894	0.965729672	-0.361109987	0.723201464	0.988677831	-4.982520372
hsa-mir-134	MI0000474	0.030646192	1.136718557	-0.373464656	0.714182434	0.9869374	-4.979771183
hsa-miR-4644	MIMAT0019704	0.030512011	1.776979096	-0.127495916	0.900291487	0.999227185	-5.017306189
hsa-mir-181b-1	MI0000270	0.030490664	0.966530782	-0.273779918	0.788098137	0.994214304	-4.999368926
hsa-mir-6857	MI0022703	0.030480278	0.918301724	-0.378402425	0.710590037	0.9869374	-4.978647252
hsa-mir-892a	MI0005528	0.030439924	0.888289788	-0.353812446	0.728548889	0.989647177	-4.984101891
hsa-miR-6785-3p	MIMAT0027471	0.030332513	1.311374037	-0.243049215	0.811356888	0.994603723	-5.004212075
hsa-miR-3649	MIMAT0018069	0.030268456	0.780653786	-0.574395718	0.574464955	0.967644566	-4.922630954
hsa-miR-218-2-3p	MIMAT0004566	0.030259091	0.829922118	-0.225012681	0.825095812	0.995938202	-5.006789464
hsa-mir-544a	MI0003515	0.030209681	0.922725647	-0.535005961	0.600719093	0.973257028	-4.935648879
hsa-mir-1285-1	MI0006346	0.0301634	1.160942513	-0.333365026	0.74360936	0.990306303	-4.988365263
hsa-mir-1197	MI0006656	0.030161314	0.780980246	-0.409589733	0.688067254	0.98554565	-4.971217547
hsa-mir-4778	MI0017422	0.030100769	0.901164856	-0.418540866	0.681657895	0.985064407	-4.968979986
hsa-mir-3935	MI0016591	0.030047727	0.990567933	-0.381405342	0.708408776	0.986717613	-4.977956712
hsa-mir-654	MI0003676	0.030002738	0.909955231	-0.472455101	0.643603247	0.982649571	-4.954518543
hsa-mir-1304	MI0006371	0.029993684	0.925392452	-0.395890933	0.697924139	0.985651988	-4.974551228
hsa-mir-4705	MI0017338	0.029952471	0.885302243	-0.43537723	0.669671306	0.984005175	-4.964644836
hsa-mir-4769	MI0017410	0.029860641	1.059669205	-0.382268232	0.707782478	0.986573137	-4.977757305
hsa-miR-5007-3p	MIMAT0021036	0.029770387	0.883563421	-0.36614271	0.719522301	0.98842922	-4.98141135
hsa-mir-744	MI0005559	0.029726434	0.956297895	-0.379556354	0.709751534	0.9869374	-4.978382527
hsa-mir-4432	MI0016772	0.02970932	0.899613024	-0.418977341	0.681346005	0.985064407	-4.968869682
hsa-mir-2682	MI0012063	0.029669144	0.880459855	-0.398756876	0.695857226	0.985651988	-4.973862872
hsa-mir-548ad	MI0016770	0.029313922	0.947524355	-0.382881243	0.707337678	0.986573137	-4.977615376
hsa-miR-212-5p	MIMAT0022695	0.029261857	0.90923974	-0.453041424	0.657194644	0.984005175	-4.95991965
hsa-miR-4771	MIMAT0019925	0.029240682	0.962734556	-0.269876414	0.79104149	0.994214304	-5.000015616
hsa-mir-607	MI0003620	0.029092895	0.925268468	-0.382356477	0.70771844	0.986573137	-4.977736887
hsa-miR-6796-3p	MIMAT0027493	0.029074994	1.535692727	-0.146738321	0.885350347	0.998806144	-5.015689488
hsa-mir-3660	MI0016061	0.028957299	0.886444919	-0.491092935	0.630677813	0.980034222	-4.949129458
hsa-mir-424	MI0001446	0.0289318	1.017294345	-0.200285276	0.844027164	0.996080495	-5.010003103
hsa-mir-542	MI0003686	0.028781018	0.991866573	-0.377269461	0.711413683	0.9869374	-4.978906405
hsa-mir-1180	MI0006273	0.028779436	0.909899516	-0.349113124	0.732000185	0.989764242	-4.98510365
hsa-miR-6822-3p	MIMAT0027545	0.028769702	0.854273703	-0.425274201	0.67685322	0.985064407	-4.967266028
hsa-miR-6886-3p	MIMAT0027673	0.028762824	0.982743563	-0.303775384	0.76559542	0.993908457	-4.994094748
hsa-miR-3940-3p	MIMAT0018356	0.028743002	0.948302612	-0.39183479	0.700853659	0.98572659	-4.975517222
hsa-mir-4515	MI0016881	0.028734577	0.926034894	-0.41820482	0.681898062	0.985064407	-4.969064833
hsa-miR-3927-3p	MIMAT0018202	0.028650306	0.865875885	-0.350532456	0.730957157	0.989764242	-4.984802468
hsa-mir-6768	MI0022613	0.028604032	0.933876601	-0.294029347	0.772884097	0.994214304	-4.995867507
hsa-miR-6853-5p	MIMAT0027606	0.028572799	0.801371736	-0.402268751	0.693327872	0.98554565	-4.973012813
hsa-miR-4268	MIMAT0016896	0.028533196	0.770709333	-0.345441596	0.734700827	0.989764242	-4.985877213
hsa-mir-3074	MI0014181	0.028403866	1.049118099	-0.316337669	0.756234425	0.99324537	-4.991726027
hsa-mir-4299	MI0015829	0.028383092	0.938760732	-0.569357626	0.577789287	0.967979651	-4.92434442
hsa-miR-504-5p	MIMAT0002875	0.02799014	0.955263316	-0.361088511	0.72321718	0.988677831	-4.982525072
hsa-miR-6083	MIMAT0023708	0.027962324	1.009006611	-0.309423229	0.761382048	0.99324537	-4.993041454
hsa-mir-30b	MI0000441	0.027876905	0.873387008	-0.300060222	0.768371181	0.994214304	-4.994777217
hsa-mir-544b	MI0014159	0.027764382	1.008405473	-0.382177283	0.70784848	0.986573137	-4.977778343
hsa-mir-3609	MI0015999	0.027740624	0.927980513	-0.489477534	0.631793252	0.98055505	-4.949604423
hsa-miR-101-5p	MIMAT0004513	0.027667811	0.963324216	-0.273165651	0.788561091	0.994214304	-4.999471298
hsa-mir-4309	MI0015837	0.027659146	1.022843392	-0.273262456	0.788488126	0.994214304	-4.99945518
hsa-miR-4662a-5p	MIMAT0019731	0.02759594	0.862847503	-0.342357824	0.736971945	0.989764242	-4.986520767
hsa-miR-4716-5p	MIMAT0019826	0.027580129	0.922762747	-0.280360867	0.783143503	0.994214304	-4.998257953
hsa-mir-2052	MI0010486	0.027547655	0.914648416	-0.343512514	0.736121247	0.989764242	-4.986280455
hsa-mir-302e	MI0006417	0.027504584	0.921558884	-0.331572009	0.744935296	0.990306303	-4.988727283
hsa-mir-8067	MI0025903	0.027473049	0.916374585	-0.368660818	0.717684129	0.987694154	-4.980850843

hsa-miR-1271-3p	MIMAT0022712	0.027362712	0.989015673	-0.307593642	0.762746097	0.993609336	-4.993384748
hsa-mir-1305	MI0006372	0.027319519	0.836892015	-0.414771465	0.684353873	0.98554565	-4.969927942
hsa-miR-885-5p	MIMAT0004947	0.027315496	1.136295778	-0.19734463	0.846285424	0.996080495	-5.010360618
hsa-miR-4423-3p	MIMAT0018936	0.027199785	1.102693129	-0.281961617	0.781939798	0.994214304	-4.997983791
hsa-miR-548o-3p	MIMAT0005919	0.027199248	0.872281516	-0.442424453	0.664681313	0.984005175	-4.962781369
hsa-miR-6806-3p	MIMAT0027513	0.02719422	0.866280648	-0.44096673	0.66571216	0.984005175	-4.963169191
hsa-miR-6840-3p	MIMAT0027583	0.027086123	2.238382769	-0.059368655	0.953465385	0.999227185	-5.021209913
hsa-mir-4269	MI0015875	0.02707253	1.036211778	-0.311123325	0.760115276	0.99324537	-4.992720665
hsa-mir-5579	MI0019133	0.02703694	0.991516623	-0.421008956	0.679895079	0.985064407	-4.968354804
hsa-mir-222	MI0000299	0.027029241	1.025584673	-0.271962672	0.789467979	0.994214304	-4.999671127
hsa-miR-6769a-5p	MIMAT0027438	0.027003649	1.193134792	-0.411103378	0.686981665	0.98554565	-4.970842459
hsa-mir-142	MI0000458	0.026885993	1.027529329	-0.304853739	0.764790354	0.993908457	-4.993895112
hsa-mir-7111	MI0022962	0.0268851	0.899055596	-0.348954663	0.732116668	0.989764242	-4.985137202
hsa-miR-4290	MIMAT0016921	0.026850119	0.97732082	-0.247360496	0.808082172	0.994603723	-5.003566919
hsa-miR-514a-3p	MIMAT0002883	0.026849107	0.836560931	-0.430774664	0.672939033	0.984005175	-4.965846312
hsa-mir-324	MI0000813	0.026817	1.26280032	-0.181085996	0.858795866	0.996937676	-5.012242528
hsa-mir-24-1	MI0000080	0.026799808	0.91816774	-0.287644024	0.777671519	0.994214304	-4.996998152
hsa-mir-7846	MI0025516	0.026772935	1.08426445	-0.321495315	0.75240245	0.992432131	-4.99072626
hsa-miR-141-5p	MIMAT0004598	0.026738895	0.828037586	-0.337623647	0.740463487	0.990306303	-4.987497769
hsa-miR-5586-5p	MIMAT0022287	0.026703004	0.883646687	-0.282991766	0.781165469	0.994214304	-4.997806544
hsa-miR-660-3p	MIMAT0022711	0.026695895	0.991921749	-0.258600136	0.799562501	0.994214304	-5.001832301
hsa-miR-3152-5p	MIMAT0019207	0.02662022	0.89976683	-0.43032407	0.673259314	0.984315135	-4.965963275
hsa-miR-4803	MIMAT0019983	0.026606282	0.81643095	-0.373868855	0.713888101	0.9869374	-4.979679719
hsa-miR-32-3p	MIMAT0004505	0.026602469	0.982105058	-0.430869062	0.672871943	0.984005175	-4.965821793
hsa-mir-6124	MI0021258	0.02658267	1.031051351	-0.256031902	0.801506947	0.994214304	-5.002235364
hsa-miR-3131	MIMAT0014996	0.026374268	1.126561153	-0.375699796	0.712555419	0.9869374	-4.979264201
hsa-mir-1249	MI0006384	0.026337786	0.852371371	-0.462143879	0.650806064	0.982649571	-4.95741429
hsa-miR-6813-3p	MIMAT0027527	0.026173261	1.031891243	-0.25625764	0.801335983	0.994214304	-5.002200096
hsa-let-7i-3p	MIMAT0004585	0.026149399	0.928057874	-0.30347423	0.765820302	0.993908457	-4.994150377
hsa-miR-187-3p	MIMAT0000262	0.026108753	0.878879331	-0.204896217	0.840489067	0.996080495	-5.009431956
hsa-miR-197-5p	MIMAT0022691	0.026073997	2.928160233	-0.071679142	0.943832251	0.999227185	-5.02071495
hsa-miR-5581-3p	MIMAT0022276	0.025960007	0.964982662	-0.358877442	0.724835838	0.989068072	-4.98300755
hsa-miR-4260	MIMAT0016881	0.02583191	0.908524927	-0.437102932	0.668447868	0.984005175	-4.964191179
hsa-miR-1537-5p	MIMAT0026765	0.025828143	0.890480985	-0.341614844	0.737519512	0.989764242	-4.986674977
hsa-mir-7975	MI0025751	0.025744772	0.957951645	-0.323247718	0.751101997	0.991856256	-4.990382965
hsa-mir-3934	MI0016590	0.025662204	0.952014428	-0.313652219	0.758232269	0.99324537	-4.992240303
hsa-miR-3943	MIMAT0018359	0.025564528	0.901333017	-0.284274171	0.780201863	0.994214304	-4.997585004
hsa-mir-4491	MI0016853	0.025434099	0.959432719	-0.34250711	0.736861942	0.989764242	-4.986489743
hsa-mir-378f	MI0016756	0.025400516	0.908731457	-0.339106214	0.739369427	0.990306303	-4.987193239
hsa-miR-25-3p	MIMAT0000081	0.025380613	8.733835325	-0.123595429	0.903325059	0.999227185	-5.017606335
hsa-mir-5684	MI0019285	0.025371478	0.845378874	-0.37448577	0.71343896	0.9869374	-4.979539935
hsa-mir-2110	MI0010629	0.025288949	0.894022135	-0.347631293	0.733089727	0.989764242	-4.985416822
hsa-miR-6879-3p	MIMAT0027659	0.025260805	1.013191549	-0.22224626	0.827208446	0.996080495	-5.007167389
hsa-miR-561-3p	MIMAT0003225	0.025258458	0.80050916	-0.29808166	0.769850795	0.994214304	-4.995137309
hsa-mir-4704	MI0017337	0.025205139	0.832257216	-0.276664516	0.785925213	0.994214304	-4.998885158
hsa-mir-4330	MI0015902	0.025163293	0.854893246	-0.296736096	0.770857569	0.994214304	-4.99538086
hsa-miR-4453	MIMAT0018975	0.025151608	0.915615741	-0.269722992	0.791157242	0.994214304	-5.000040846
hsa-miR-373-3p	MIMAT0000726	0.025136988	0.95374627	-0.313243251	0.758536678	0.99324537	-4.992318245
hsa-miR-6891-3p	MIMAT0027683	0.025086933	0.977523293	-0.363836804	0.721207145	0.988653693	-4.981921341
hsa-miR-5197-5p	MIMAT0021130	0.02500997	0.890454819	-0.208749505	0.837535065	0.996080495	-5.00894477
hsa-miR-3977	MIMAT0019362	0.024733234	0.924464277	-0.258678501	0.799503192	0.994214304	-5.00181994
hsa-miR-4320	MIMAT0016871	0.02468316	0.826537159	-0.402826822	0.69292628	0.98554565	-4.972877065
hsa-miR-1199-3p	MIMAT0031120	0.024653525	0.906063333	-0.28043019	0.783091363	0.994214304	-4.998246112
hsa-miR-212-3p	MIMAT0000269	0.024653308	0.976516946	-0.351903428	0.72995019	0.989764242	-4.984510415
hsa-mir-4463	MI0016811	0.024647635	0.946534366	-0.342973884	0.736518029	0.989764242	-4.986392652
hsa-miR-4537	MIMAT0019080	0.024643899	0.871048096	-0.354849421	0.727788125	0.989333548	-4.983879078
hsa-miR-4302	MIMAT0016855	0.024578775	0.798700533	-0.299186957	0.769024114	0.994214304	-4.994936437
hsa-mir-26a-2	MI0000750	0.024551099	0.815280393	-0.288002281	0.777402665	0.994214304	-4.996935362
hsa-miR-4293	MIMAT0016848	0.024528515	0.866604414	-0.34025478	0.738522243	0.989829051	-4.986956419
hsa-miR-6760-5p	MIMAT0027420	0.024525474	1.167334897	-0.295559034	0.771738618	0.994214304	-4.995593023
hsa-mir-6780a	MI0022625	0.024514288	0.930600434	-0.343062249	0.73645293	0.989764242	-4.986374258
hsa-miR-581	MIMAT0003246	0.024422153	0.863155513	-0.249846129	0.806195845	0.994603723	-5.003189865
hsa-miR-301b-3p	MIMAT0004958	0.02434339	0.864660399	-0.329932999	0.746148074	0.990306303	-4.989056536
hsa-miR-4265	MIMAT0016891	0.024327956	0.761470324	-0.42127583	0.679704582	0.985064407	-4.968286991
hsa-miR-6747-3p	MIMAT0027395	0.024301332	0.997292252	-0.228547125	0.8223987	0.995221754	-5.006299883
hsa-miR-676-5p	MIMAT0018203	0.024285851	0.830946253	-0.243956923	0.810667115	0.994603723	-5.004077174

hsa-miR-219a-1-3p	MIMAT0004567	0.024163103	0.88469677	-0.326434256	0.748739279	0.990697422	-4.989754039
hsa-miR-892a	MIMAT0004907	0.02405791	0.798826426	-0.264097929	0.795404677	0.994214304	-5.000956122
hsa-miR-1304-5p	MIMAT0005892	0.02402553	0.998188033	-0.277309625	0.785439513	0.994214304	-4.998776285
hsa-miR-6777-5p	MIMAT0027454	0.024002169	1.664524116	-0.095702581	0.925061112	0.999227185	-5.019481588
hsa-miR-6133	MIMAT0024617	0.023965361	1.518951173	-0.132964718	0.896040892	0.999057213	-5.016869709
hsa-mir-6088	MI0020365	0.023942181	0.847176581	-0.462591883	0.650492357	0.982649571	-4.957289748
hsa-mir-6852	MI0022698	0.023767349	1.047942768	-0.187230791	0.854062857	0.996937676	-5.011550155
hsa-mir-200c	MI0000650	0.023762893	0.944672175	-0.239492041	0.814061562	0.994603723	-5.004735942
hsa-miR-3136-5p	MIMAT0015003	0.023620744	0.923003657	-0.320965984	0.752795419	0.992468979	-4.990829596
hsa-miR-525-3p	MIMAT0002839	0.023473681	0.938647175	-0.294916248	0.77221989	0.994214304	-4.995708534
hsa-miR-3921	MIMAT0018196	0.023418911	1.938541451	-0.127265405	0.90047072	0.999227185	-5.017324185
hsa-miR-4704-3p	MIMAT0019804	0.02335968	0.897532987	-0.261431218	0.797420641	0.994214304	-5.001383385
hsa-mir-6895	MI0022742	0.023345204	1.022435984	-0.287378955	0.777870459	0.994214304	-4.99704456
hsa-miR-4645-3p	MIMAT0019706	0.023310286	0.93250932	-0.268083005	0.79239488	0.994214304	-5.000309659
hsa-mir-6851	MI0022697	0.023213529	0.984624387	-0.229525813	0.821652278	0.994979764	-5.006162983
hsa-mir-4263	MI0015876	0.023191688	0.960959765	-0.309701999	0.761174283	0.99324537	-4.992988971
hsa-miR-2276-5p	MIMAT0026921	0.022993358	0.773014124	-0.369075297	0.71738174	0.987670493	-4.980758225
hsa-miR-6770-3p	MIMAT0027441	0.022954276	0.79113233	-0.324512272	0.750164059	0.991311193	-4.990134103
hsa-miR-302b-5p	MIMAT0000714	0.02292394	0.966226226	-0.247586736	0.807910429	0.994603723	-5.003532754
hsa-mir-4439	MI0016782	0.022923369	0.963778	-0.222464714	0.827041569	0.996080495	-5.007137714
hsa-miR-1293	MIMAT0005883	0.022911167	0.88403589	-0.204311112	0.840937838	0.996080495	-5.009505146
hsa-miR-5681b	MIMAT0022480	0.02284965	0.907141832	-0.345662023	0.734538586	0.989764242	-4.985830997
hsa-mir-3130-2	MI0014148	0.02282011	0.849009329	-0.274222832	0.787764378	0.994214304	-4.999294971
hsa-mir-4708	MI0017341	0.022804199	1.090243558	-0.257422785	0.80045372	0.994214304	-5.002017569
hsa-mir-5189	MI0018168	0.022791638	0.977833807	-0.282420319	0.781594977	0.994214304	-4.997904945
hsa-miR-3679-3p	MIMAT0018105	0.022770099	0.89248434	-0.302311185	0.766688992	0.994214304	-4.994364704
hsa-miR-6874-3p	MIMAT0027649	0.022737279	0.819452971	-0.357404516	0.725914879	0.989104742	-4.983327354
hsa-miR-6895-3p	MIMAT0027691	0.022621505	0.826311381	-0.27703581	0.785645656	0.994214304	-4.998822527
hsa-miR-190a-3p	MIMAT0026482	0.022595298	1.012062846	-0.297746625	0.770101434	0.994214304	-4.995198052
hsa-mir-4449	MI0016792	0.022526536	2.394596406	-0.067631585	0.946998597	0.999227185	-5.020887941
hsa-mir-4253	MI0015860	0.022438073	0.964922967	-0.271412981	0.789882478	0.994214304	-4.999762147
hsa-mir-511	MI0003127	0.022394472	1.071916646	-0.229749225	0.821481912	0.994979764	-5.006131651
hsa-miR-4485-3p	MIMAT0019019	0.022381593	4.798590881	-0.037005887	0.970982742	0.999227185	-5.021871269
hsa-miR-449b-5p	MIMAT0003327	0.022272235	0.902674721	-0.326415969	0.748752831	0.990697422	-4.989757666
hsa-miR-7155-3p	MIMAT0028221	0.022230835	0.88862281	-0.210800471	0.835963785	0.996080495	-5.008681788
hsa-miR-3660	MIMAT0018081	0.022129037	0.785658359	-0.346858462	0.7336582	0.989764242	-4.985579637
hsa-miR-1297	MIMAT0005886	0.022001799	0.905469845	-0.295552754	0.771743319	0.994214304	-4.995594153
hsa-mir-6748	MI0022593	0.021973765	1.068488894	-0.234000727	0.818241652	0.994603723	-5.005529652
hsa-mir-1183	MI0006276	0.021876449	1.043861044	-0.18791234	0.85353825	0.996937676	-5.011471947
hsa-miR-3164	MIMAT0015038	0.021827317	0.986274325	-0.240726681	0.813122532	0.994603723	-5.004554981
hsa-miR-659-3p	MIMAT0003337	0.021797834	1.06224774	-0.227509307	0.823190413	0.995589896	-5.006444422
hsa-miR-3126-3p	MIMAT0015377	0.021787348	0.972544088	-0.280020225	0.783399727	0.994214304	-4.998316097
hsa-miR-4445-5p	MIMAT0018963	0.021596391	0.875861414	-0.269421752	0.791384534	0.994214304	-5.000090344
hsa-miR-615-5p	MIMAT0004804	0.021503409	0.954870257	-0.241666992	0.812407559	0.994603723	-5.004416543
hsa-mir-4325	MI0015865	0.02138936	0.881326858	-0.257343954	0.800513404	0.994214304	-5.002029945
hsa-miR-644a	MIMAT0003314	0.021382637	0.861203257	-0.240692918	0.813148207	0.994603723	-5.004559942
hsa-miR-499a-3p	MIMAT0004772	0.021357491	0.879611037	-0.180508142	0.859241248	0.996937676	-5.012306457
hsa-mir-4999	MI0017865	0.021339098	0.89068301	-0.302099588	0.766847071	0.994214304	-4.99440361
hsa-mir-9-2	MI0000467	0.021184816	0.991165038	-0.402312686	0.693296253	0.98554565	-4.973002133
hsa-mir-5006	MI0017873	0.021162581	0.946606393	-0.311838779	0.759582391	0.99324537	-4.992585152
hsa-miR-618	MIMAT0003287	0.021155428	1.018042427	-0.235208999	0.817321398	0.994603723	-5.005356572
hsa-mir-378h	MI0016808	0.021107642	1.03395497	-0.214017927	0.833500298	0.996080495	-5.0082641
hsa-mir-3119-1	MI0014134	0.021072115	0.922855618	-0.19395905	0.848887098	0.996080495	-5.010765728
hsa-mir-128-1	MI0000447	0.020932864	0.892113644	-0.308548248	0.762034289	0.99324537	-4.99320588
hsa-miR-6837-5p	MIMAT0027576	0.020795115	1.143210059	-0.248770007	0.807012353	0.994603723	-5.003353563
hsa-mir-548f-2	MI0006375	0.020770486	0.847385026	-0.293734799	0.773104727	0.994214304	-4.9959202
hsa-miR-1827	MIMAT0006767	0.020677628	0.783421835	-0.262748666	0.796424497	0.994214304	-5.001172837
hsa-mir-6847	MI0022693	0.02064685	0.856214989	-0.338931498	0.739498328	0.990306303	-4.987229195
hsa-mir-3120	MI0014136	0.020616041	0.969045738	-0.270941511	0.790238048	0.994214304	-4.999840071
hsa-miR-889-5p	MIMAT0026719	0.020533728	0.994655127	-0.187500869	0.853854962	0.996937676	-5.011519197
hsa-miR-3714	MIMAT0018165	0.020511734	1.060654302	-0.234535441	0.817834365	0.994603723	-5.005453165
hsa-miR-1911-3p	MIMAT0007886	0.020434785	0.800491786	-0.223937592	0.82591666	0.996080495	-5.006936883
hsa-mir-27b	MI0000440	0.020431195	0.938570392	-0.22687804	0.823672081	0.995590185	-5.006532021
hsa-miR-4418	MIMAT0018930	0.020421991	0.846999221	-0.233253601	0.818810824	0.994603723	-5.005636234
hsa-miR-4480	MIMAT0019014	0.020363881	0.819732746	-0.222758224	0.826817369	0.996080495	-5.007097798
hsa-mir-505	MI0003190	0.020358782	0.953631419	-0.325092279	0.749733996	0.99116432	-4.990019641

hsa-mir-1264	MI0003758	0.020276225	0.99595338	-0.226862927	0.823683613	0.995590185	-5.006534115
hsa-mir-7158	MI0023618	0.020206973	0.839817013	-0.253950796	0.803083581	0.994214304	-5.002559064
hsa-mir-10a	MI0000266	0.020109487	0.962039756	-0.232935111	0.819053486	0.994603723	-5.005681565
hsa-mir-6816	MI0022661	0.020104977	0.894530617	-0.206230891	0.839465597	0.996080495	-5.009264227
hsa-mir-3652	MI0016052	0.019773335	0.971620214	-0.157452322	0.877050366	0.998806144	-5.014691408
hsa-miR-6082	MIMAT0023707	0.019729415	0.801418124	-0.222596566	0.826940851	0.996080495	-5.007119789
hsa-miR-548az-5p	MIMAT0025456	0.019696998	0.908919141	-0.22669507	0.823811704	0.995678798	-5.006557366
hsa-mir-6887	MI0022734	0.019619804	1.126336318	-0.137418894	0.89258134	0.999057213	-5.016500712
hsa-mir-96	MI0000098	0.019589662	0.835971304	-0.300552511	0.76800318	0.994214304	-4.994687259
hsa-mir-135a-1	MI0000452	0.019571958	0.882357957	-0.269730675	0.791151446	0.994214304	-5.000039583
hsa-miR-3689b-3p	MIMAT0018181	0.019441125	0.888186956	-0.280129227	0.783317736	0.994214304	-4.998297499
hsa-miR-3689c	MIMAT0019007	0.019441125	0.888186956	-0.280129227	0.783317736	0.994214304	-4.998297499
hsa-miR-504-3p	MIMAT0026612	0.019425094	1.901154019	-0.062500224	0.951014112	0.999227185	-5.021092815
hsa-miR-620	MIMAT0003289	0.019383292	1.021008538	-0.201815617	0.842852502	0.996080495	-5.009814973
hsa-miR-3941	MIMAT0018357	0.019238801	0.908926317	-0.292350917	0.774141591	0.994214304	-4.996167071
hsa-mir-100	MI0000102	0.019230616	0.901252153	-0.206053435	0.839601658	0.996080495	-5.00928659
hsa-miR-3064-3p	MIMAT0019865	0.019206724	0.892736983	-0.232196724	0.819616148	0.994603723	-5.005786427
hsa-mir-3134	MI0014155	0.019151892	0.979828325	-0.340215447	0.738551249	0.989829051	-4.986964541
hsa-mir-8069-1	MI0025905	0.019068196	1.01408753	-0.244427305	0.810309733	0.994603723	-5.004007072
hsa-miR-3135a	MIMAT0015001	0.019051212	0.870227646	-0.179270422	0.86019539	0.997215479	-5.012442707
hsa-miR-6887-5p	MIMAT0027674	0.019023922	1.309808268	-0.155613611	0.878473755	0.998806144	-5.014867671
hsa-miR-887-3p	MIMAT0004951	0.018976145	0.957944188	-0.197419947	0.846227567	0.996080495	-5.010351527
hsa-miR-4759	MIMAT0019905	0.018931633	0.939629266	-0.218601264	0.829994132	0.996080495	-5.007658268
hsa-mir-3926-1	MI0016434	0.018922349	1.001510914	-0.265827191	0.794098205	0.994214304	-5.000676772
hsa-miR-3973	MIMAT0019358	0.018858322	0.992858095	-0.280693918	0.782893013	0.994214304	-4.998201038
hsa-miR-3129-5p	MIMAT0014992	0.018853547	0.82962786	-0.199328324	0.844761898	0.996080495	-5.010120022
hsa-miR-211-5p	MIMAT0000268	0.018849388	0.93077782	-0.167597962	0.869204427	0.997250847	-5.013681787
hsa-miR-6835-3p	MIMAT0027571	0.018841252	0.879733105	-0.223656637	0.826131209	0.996080495	-5.006975293
hsa-miR-490-3p	MIMAT0002806	0.018667447	0.86158967	-0.233035981	0.81897663	0.994603723	-5.005667215
hsa-mir-4714	MI0017348	0.018632924	0.964917097	-0.241261759	0.812715659	0.994603723	-5.004476269
hsa-miR-4687-5p	MIMAT0019774	0.018627532	1.258983627	-0.174870133	0.86358933	0.997250847	-5.012919551
hsa-mir-888	MI0005537	0.018594754	0.880480969	-0.218958581	0.829720948	0.996080495	-5.007610503
hsa-mir-506	MI0003193	0.018594509	0.883976567	-0.267457515	0.792867068	0.994214304	-5.000411759
hsa-miR-6860	MIMAT0027622	0.018492556	2.624645406	-0.050411311	0.960479455	0.999227185	-5.021511647
hsa-miR-6805-5p	MIMAT0027510	0.018395139	7.30641712	-0.050783006	0.960188327	0.999227185	-5.021500105
hsa-mir-5681b	MI0019293	0.018390952	0.858511064	-0.210375388	0.836289388	0.996080495	-5.008736503
hsa-miR-516b-3p	MIMAT0002860	0.018343634	0.831853057	-0.190634515	0.851443635	0.996641394	-5.011156762
hsa-miR-516a-3p	MIMAT0006778	0.018343634	0.831853057	-0.190634515	0.851443635	0.996641394	-5.011156762
hsa-miR-4436b-5p	MIMAT0019940	0.018262706	1.120180991	-0.21959032	0.829238011	0.996080495	-5.007525864
hsa-mir-1261	MI0006396	0.01824421	0.900830747	-0.293569064	0.773228879	0.994214304	-4.995949826
hsa-mir-5685	MI0019287	0.018106063	0.911340926	-0.267171085	0.793083324	0.994214304	-5.000458434
hsa-miR-1538	MIMAT0007400	0.018085124	1.047223651	-0.144312973	0.887231199	0.998806144	-5.015905708
hsa-mir-493	MI0003132	0.017943329	0.828888364	-0.249722955	0.806289292	0.994603723	-5.003208638
hsa-miR-449b-3p	MIMAT0009203	0.017850074	1.078729085	-0.136020075	0.893667561	0.999057213	-5.016617899
hsa-miR-200b-5p	MIMAT0004571	0.017847257	0.756070759	-0.26028573	0.79828706	0.994214304	-5.001565602
hsa-miR-892c-5p	MIMAT0025857	0.017796439	0.932698022	-0.261484628	0.797380249	0.994214304	-5.00137487
hsa-mir-616	MI0003629	0.017793219	0.98877918	-0.232214894	0.819602301	0.994603723	-5.00578385
hsa-miR-542-5p	MIMAT0003340	0.017720681	0.789010052	-0.215546051	0.832330901	0.996080495	-5.008063524
hsa-mir-4312	MI0015842	0.017580648	0.960440546	-0.169028008	0.868099653	0.997250847	-5.013534439
hsa-mir-28	MI0000086	0.017485187	1.826281592	-0.115510248	0.909618163	0.999227185	-5.018198882
hsa-miR-3672	MIMAT0018095	0.017475486	0.965371293	-0.228054784	0.822774264	0.9953669	-5.006368533
hsa-miR-1248	MIMAT0005900	0.017399215	0.962701559	-0.193360626	0.849347152	0.996080495	-5.010836611
hsa-miR-6499-5p	MIMAT0025450	0.017334345	0.824880733	-0.202774065	0.842117013	0.996080495	-5.009696424
hsa-mir-6842	MI0022688	0.017325756	1.054679191	-0.168015127	0.86888212	0.997250847	-5.013638932
hsa-miR-580-5p	MIMAT0026617	0.017322673	0.892949112	-0.176038544	0.862687861	0.997250847	-5.012794083
hsa-mir-126	MI0000471	0.017272836	1.029088993	-0.214755241	0.832936017	0.996080495	-5.008167499
hsa-mir-6715b	MI0022549	0.017259675	1.06872748	-0.23838751	0.814901886	0.994603723	-5.004897053
hsa-mir-4775	MI0017418	0.017220182	0.912635457	-0.27440916	0.787623982	0.994214304	-4.999263824
hsa-mir-1252	MI0006434	0.017181126	1.14682784	-0.26331841	0.795993817	0.994214304	-5.00108146
hsa-mir-8055	MI0025891	0.017169784	1.062246404	-0.147541008	0.884728022	0.998806144	-5.015617138
hsa-mir-6774	MI0022619	0.017070575	0.916965568	-0.173463096	0.864675168	0.997250847	-5.013069541
hsa-miR-6741-3p	MIMAT0027384	0.017067827	0.842334693	-0.177237578	0.861762971	0.997250847	-5.012664463
hsa-miR-4312	MIMAT0016864	0.016994134	0.918272373	-0.180609523	0.859163105	0.996937676	-5.012295256
hsa-miR-6864-3p	MIMAT0027629	0.016992633	0.855959105	-0.276542346	0.786017205	0.994214304	-4.998905748
hsa-mir-3939	MI0016596	0.016933792	0.927801984	-0.190628747	0.851448072	0.996641394	-5.011157435
hsa-miR-153-3p	MIMAT0000439	0.016820451	0.851100596	-0.281606845	0.782206524	0.994214304	-4.998044686

hsa-miR-8079	MIMAT0031006	0.01674331	0.826549218	-0.248306034	0.807364464	0.994603723	-5.003423926
hsa-miR-3155b	MIMAT0019012	0.016658169	0.816871088	-0.206977148	0.838893475	0.996080495	-5.009169974
hsa-miR-624-5p	MIMAT0003293	0.016593505	0.840628773	-0.236581208	0.816276622	0.994603723	-5.005158939
hsa-miR-6081	MIMAT0023706	0.016457906	1.019762336	-0.228450858	0.82247213	0.995221754	-5.006313318
hsa-mir-3922	MI0016429	0.016446102	1.011322082	-0.193376257	0.849335134	0.996080495	-5.010834762
hsa-miR-186-5p	MIMAT0000456	0.016318943	0.847106596	-0.188261262	0.853269703	0.996911892	-5.011431799
hsa-mir-4788	MI0017435	0.015996046	1.07741328	-0.206387888	0.839345227	0.996080495	-5.009244426
hsa-miR-500a-5p	MIMAT0004773	0.015914256	0.996672081	-0.13138915	0.897265161	0.999057213	-5.016997332
hsa-mir-4668	MI0017298	0.015867718	0.892586549	-0.149185792	0.883453063	0.998806144	-5.015467658
hsa-miR-142-3p	MIMAT0000434	0.015785114	0.894503954	-0.23485958	0.817587497	0.994603723	-5.005406715
hsa-mir-5584	MI0019141	0.015577545	0.98461525	-0.185711485	0.855232565	0.996937676	-5.01172348
hsa-miR-767-3p	MIMAT0003883	0.015568353	0.879833703	-0.233155589	0.818885498	0.994603723	-5.00565019
hsa-miR-6866-3p	MIMAT0027633	0.015549986	0.893749437	-0.161022678	0.87428773	0.99830329	-5.014343263
hsa-mir-4526	MI0016893	0.015447246	1.00493891	-0.278592966	0.784473567	0.994214304	-4.998558957
hsa-miR-6501-3p	MIMAT0025459	0.015440426	0.974989119	-0.142355338	0.888749853	0.998806144	-5.016077613
hsa-mir-4696	MI0017329	0.015408912	0.843737829	-0.202184886	0.842569116	0.996080495	-5.009769364
hsa-miR-3151-5p	MIMAT0015024	0.015293098	1.241869523	-0.098014466	0.923256926	0.999227185	-5.019344251
hsa-mir-136	MI0000475	0.01509774	0.989079874	-0.21537858	0.832459038	0.996080495	-5.008085574
hsa-miR-4435	MIMAT0018951	0.014802938	0.877052638	-0.15342691	0.880167097	0.998806144	-5.01507461
hsa-mir-4671	MI0017302	0.014780818	0.849448191	-0.178397742	0.860868263	0.997250847	-5.012538212
hsa-miR-4318	MIMAT0016869	0.014764483	0.87197863	-0.182539543	0.857675762	0.996937676	-5.01208082
hsa-mir-595	MI0003607	0.014758145	0.924258984	-0.195979832	0.847333992	0.996080495	-5.010524764
hsa-mir-4450	MI0016795	0.014664584	0.948380665	-0.224743044	0.825301664	0.996055622	-5.006826503
hsa-miR-4793-5p	MIMAT0019965	0.014444349	0.97107102	-0.188978906	0.85271743	0.99684332	-5.011348992
hsa-miR-5690	MIMAT0022482	0.01443133	0.805019545	-0.184608011	0.856082346	0.996937676	-5.011848487
hsa-miR-375-3p	MIMAT0000728	0.014389666	0.688186998	-0.217940105	0.830499677	0.996080495	-5.007746446
hsa-mir-1251	MI0006386	0.014318385	0.89098498	-0.331411778	0.745053827	0.990306303	-4.988759541
hsa-mir-8056	MI0025892	0.014253568	0.811937008	-0.237513328	0.815567128	0.994603723	-5.005024042
hsa-miR-8078	MIMAT0031005	0.01424366	0.841218657	-0.18948595	0.852327274	0.996778901	-5.011290297
hsa-mir-548t	MI0014164	0.014225593	0.967317632	-0.18401803	0.856536764	0.996937676	-5.01191502
hsa-miR-4786-3p	MIMAT0019955	0.014219994	1.215048301	-0.104919957	0.917870549	0.999227185	-5.018914553
hsa-mir-144	MI0000460	0.014187783	0.957472284	-0.173484977	0.86465828	0.997250847	-5.013067218
hsa-miR-4524b-3p	MIMAT0022256	0.014156013	0.837701283	-0.155483456	0.878574528	0.998806144	-5.01488007
hsa-miR-3141	MIMAT0015010	0.014126072	5.808300267	-0.041555869	0.967417059	0.999227185	-5.021761569
hsa-miR-8061	MIMAT0030988	0.013982228	0.9743388	-0.19103945	0.85113215	0.996592491	-5.011109492
hsa-mir-650	MI0003665	0.013971735	0.877687129	-0.160894616	0.874386792	0.998366068	-5.014355884
hsa-miR-106a-5p	MIMAT0000103	0.013969436	10.10875443	-0.125155704	0.902111378	0.999227185	-5.017487386
hsa-miR-578	MIMAT0003243	0.013958684	0.843723795	-0.159127075	0.875754283	0.998800418	-5.01452907
hsa-mir-767	MI0003763	0.013904246	0.912432118	-0.185914249	0.855076438	0.996937676	-5.01170043
hsa-miR-4280	MIMAT0016911	0.013886992	0.885021405	-0.134161351	0.895111251	0.999057213	-5.016771766
hsa-mir-4499	MI0016862	0.013779459	1.031204954	-0.120961706	0.905374303	0.999227185	-5.017803744
hsa-miR-1181	MIMAT0005826	0.013735042	1.004680357	-0.171805094	0.865955044	0.997250847	-5.013244739
hsa-miR-4743-5p	MIMAT0019874	0.013721717	1.86873615	-0.075169176	0.941102832	0.999227185	-5.020557726
hsa-miR-5011-5p	MIMAT0021045	0.013698802	0.948032248	-0.179331624	0.860148204	0.997192578	-5.012435991
hsa-mir-3611	MI0016001	0.013690453	1.010629883	-0.158562686	0.87619102	0.998806144	-5.014583969
hsa-miR-4441	MIMAT0018959	0.013490788	0.995234974	-0.114551365	0.910364934	0.999227185	-5.018266506
hsa-miR-3152-3p	MIMAT0015025	0.013427573	1.029154168	-0.158209049	0.876464693	0.998806144	-5.014618269
hsa-miR-4301	MIMAT0016850	0.013408891	1.079084868	-0.128410621	0.899580314	0.999227185	-5.017234456
hsa-mir-4639	MI0017266	0.013387711	0.902578498	-0.253422624	0.803483863	0.994214304	-5.002640802
hsa-mir-133a-1	MI0000450	0.013385609	1.002733954	-0.229498318	0.821673246	0.994979764	-5.006166837
hsa-miR-7152-5p	MIMAT0028214	0.013185814	0.929538527	-0.160239529	0.874893564	0.99850194	-5.014420293
hsa-mir-877	MI0005561	0.01318397	0.905961863	-0.164569788	0.871544757	0.997852043	-5.013989693
hsa-mir-1912	MI0008333	0.01305162	0.87609124	-0.164184602	0.871842539	0.997852043	-5.014028459
hsa-mir-30a	MI0000088	0.012945142	0.898586095	-0.138297268	0.891899372	0.999057213	-5.016426515
hsa-mir-4418	MI0016754	0.012943996	1.027202132	-0.142709043	0.88847543	0.998806144	-5.016046726
hsa-miR-3923	MIMAT0018198	0.012839071	1.01339962	-0.11111918	0.91303861	0.999227185	-5.018503947
hsa-mir-5695	MI0019302	0.012782165	0.887739306	-0.182007374	0.858085815	0.996937676	-5.012140173
hsa-mir-197	MI0000239	0.012673409	1.093658909	-0.114750313	0.910209988	0.999227185	-5.018252522
hsa-miR-6777-3p	MIMAT0027455	0.012650971	0.976025343	-0.134940049	0.894506381	0.999057213	-5.016707561
hsa-mir-451a	MI0001729	0.012606707	1.017218249	-0.155828799	0.87830715	0.998806144	-5.014847149
hsa-mir-4267	MI0015871	0.012606179	1.068075429	-0.169916482	0.867413409	0.997250847	-5.013442266
hsa-mir-455	MI0003513	0.012537478	0.951199193	-0.174152145	0.86414338	0.997250847	-5.012996239
hsa-mir-3116-2	MI0014129	0.01242448	0.905110713	-0.242150193	0.81204022	0.994603723	-5.004345196
hsa-mir-6824	MI0022669	0.012378845	0.969280391	-0.184501694	0.85616423	0.996937676	-5.011860492
hsa-mir-5681a	MI0019281	0.012315473	0.93284733	-0.145157293	0.88657635	0.998806144	-5.015830844
hsa-mir-5003	MI0017869	0.012206444	0.892893264	-0.147341712	0.884882529	0.998806144	-5.015635138

hsa-mir-3616	MI0016006	0.012072984	0.831766532	-0.236006539	0.816714121	0.994603723	-5.005241844
hsa-miR-103a-2-5p	MIMAT0009196	0.012020186	1.01500821	-0.103634977	0.918872543	0.999227185	-5.018996721
hsa-miR-4774-5p	MIMAT0019929	0.012020103	0.938385823	-0.151944208	0.88131562	0.998806144	-5.015213267
hsa-mir-1265	MI0006401	0.011991885	0.982275563	-0.14151605	0.889401078	0.998869208	-5.016150597
hsa-miR-4259	MIMAT0016880	0.011989285	1.115192643	-0.096866538	0.924152711	0.999227185	-5.019412852
hsa-miR-4780	MIMAT0019939	0.01191364	0.884540365	-0.178260002	0.860974476	0.997250847	-5.012553244
hsa-miR-3692-3p	MIMAT0018122	0.011813072	0.903261928	-0.182508088	0.857699998	0.996937676	-5.012084333
hsa-miR-338-3p	MIMAT0000763	0.011812054	0.845376731	-0.169637668	0.867628749	0.997250847	-5.013471243
hsa-mir-4753	MI0017392	0.011720049	0.892540714	-0.143961546	0.887503788	0.998806144	-5.01593674
hsa-mir-7159	MI0023620	0.011716528	0.934010284	-0.154397991	0.879415034	0.998806144	-5.014983071
hsa-miR-6883-3p	MIMAT0027667	0.011646491	1.010096196	-0.110777249	0.913305034	0.999227185	-5.018527207
hsa-miR-548ae-3p	MIMAT0018954	0.01140473	0.905278261	-0.141059766	0.889755155	0.998939026	-5.016190095
hsa-miR-548at-5p	MIMAT0022277	0.011356669	0.983172604	-0.116206166	0.909076242	0.999227185	-5.018149452
hsa-mir-1296	MI0003780	0.011286317	0.89857224	-0.189226773	0.852526698	0.996837241	-5.011320318
hsa-mir-365a	MI0000767	0.011205866	0.85463626	-0.124679549	0.902481735	0.999227185	-5.017523844
hsa-miR-8055	MIMAT0030982	0.011175546	0.904162471	-0.121570372	0.90490065	0.999227185	-5.017758498
hsa-miR-580-3p	MIMAT0003245	0.011139042	0.956259113	-0.142007121	0.889020034	0.998806144	-5.016107946
hsa-mir-122b	MI0017383	0.011091132	0.904288729	-0.117391992	0.90815293	0.999227185	-5.018064541
hsa-mir-3186	MI0014229	0.010886303	0.882957391	-0.15797409	0.876646533	0.998806144	-5.014641015
hsa-mir-302a	MI0000738	0.010859496	0.925509301	-0.183597723	0.856860527	0.996937676	-5.011962289
hsa-miR-6811-5p	MIMAT0027522	0.010847357	0.813630787	-0.132488332	0.896411031	0.999057213	-5.016908457
hsa-mir-3184	MI0014226	0.010783351	0.856946238	-0.220619378	0.828451495	0.996080495	-5.007387478
hsa-mir-762	MI0003892	0.0107552	0.813385661	-0.122127996	0.90446675	0.999227185	-5.017716848
hsa-mir-1273h	MI0025512	0.010732296	0.78786521	-0.161270597	0.87409596	0.998241091	-5.0143188
hsa-miR-518d-3p	MIMAT0002864	0.01065395	0.862875681	-0.192273133	0.850183335	0.996080495	-5.010964866
hsa-mir-3622b	MI0016014	0.010573995	0.980497785	-0.11378296	0.910963427	0.999227185	-5.018320291
hsa-miR-6849-3p	MIMAT0027599	0.010516575	0.974675584	-0.086459934	0.932278194	0.999227185	-5.019997949
hsa-mir-7976	MI0025752	0.010467329	1.007887536	-0.180117729	0.859542188	0.996966918	-5.012349535
hsa-miR-4777-5p	MIMAT0019934	0.010392134	0.856558473	-0.130537345	0.897927155	0.999057213	-5.017065699
hsa-miR-3194-5p	MIMAT0015078	0.010378743	0.876746321	-0.155228552	0.878771894	0.998806144	-5.014904322
hsa-mir-8064	MI0025900	0.010303364	0.922967644	-0.137832342	0.892260329	0.999057213	-5.016465846
hsa-miR-6780b-3p	MIMAT0027573	0.010284003	0.853979794	-0.165753481	0.870629789	0.99760832	-5.01387
hsa-mir-4690	MI0017323	0.010096849	1.234154516	-0.066036333	0.948246797	0.999227185	-5.020953362
hsa-mir-2276	MI0011282	0.010005167	0.960356447	-0.070631705	0.944651554	0.999227185	-5.020760681
hsa-miR-5697	MIMAT0022490	0.009992141	1.031230841	-0.092054446	0.927908965	0.999227185	-5.019691648
hsa-mir-3177	MI0014211	0.009961766	0.82065707	-0.144199173	0.887319468	0.998806144	-5.015915765
hsa-miR-6819-3p	MIMAT0027539	0.009953918	1.226869646	-0.108639612	0.914970876	0.999227185	-5.018671002
hsa-miR-4740-3p	MIMAT0019870	0.009879147	1.02167905	-0.120489147	0.905742065	0.999227185	-5.017838715
hsa-miR-6781-3p	MIMAT0027463	0.009865467	0.794951824	-0.128660023	0.899386422	0.999227185	-5.017214809
hsa-miR-3929	MIMAT0018206	0.009816135	0.906955947	-0.132535427	0.896374438	0.999057213	-5.016904632
hsa-mir-4470	MI0016821	0.009766448	0.904990102	-0.152861871	0.880604752	0.998806144	-5.015127608
hsa-miR-548w	MIMAT0015060	0.009760776	1.095790226	-0.109812136	0.914057087	0.999227185	-5.018592475
hsa-mir-6505	MI0022217	0.009547919	0.946976608	-0.113371387	0.911284014	0.999227185	-5.01834895
hsa-mir-7153	MI0023613	0.009528543	0.997225462	-0.136469375	0.893318643	0.999057213	-5.016580389
hsa-miR-203a-3p	MIMAT0000264	0.009414171	0.943226331	-0.145398947	0.886388941	0.998806144	-5.015809337
hsa-miR-3620-3p	MIMAT0018001	0.009409477	1.028336327	-0.121993662	0.904571276	0.999227185	-5.017726899
hsa-miR-3195	MIMAT0015079	0.009321394	1.210703187	-0.06628011	0.948056045	0.999227185	-5.020943466
hsa-miR-4699-3p	MIMAT0019795	0.009276312	0.943245247	-0.129412314	0.89880161	0.999149876	-5.017155315
hsa-mir-4749	MI0017388	0.009205662	0.806356949	-0.14994559	0.882864216	0.998806144	-5.01539805
hsa-mir-6506	MI0022218	0.009193832	0.887686267	-0.132981363	0.89602796	0.999057213	-5.016868352
hsa-mir-4779	MI0017423	0.009126628	0.931355122	-0.114902337	0.910091589	0.999227185	-5.018241819
hsa-mir-637	MI0003652	0.008994573	0.849632762	-0.124635401	0.902516075	0.999227185	-5.017527217
hsa-mir-4434	MI0016774	0.00885253	0.915139158	-0.104963295	0.917836757	0.999227185	-5.018911764
hsa-miR-3611	MIMAT0017988	0.008844205	0.880161388	-0.123824356	0.903146969	0.999227185	-5.017588975
hsa-miR-1255a	MIMAT0005906	0.008833217	1.12804077	-0.097445472	0.923700927	0.999227185	-5.019378355
hsa-miR-513c-5p	MIMAT0005789	0.00881895	0.881963519	-0.116558137	0.908802175	0.999227185	-5.018124339
hsa-miR-6820-3p	MIMAT0027541	0.008808828	0.975090935	-0.091115784	0.928641882	0.999227185	-5.019744378
hsa-mir-7114	MI0022965	0.008794696	1.416225523	-0.060477773	0.952597152	0.999227185	-5.021169128
hsa-miR-5009-3p	MIMAT0021042	0.008769211	0.845518493	-0.163757869	0.872172463	0.997852043	-5.0140713
hsa-miR-6861-3p	MIMAT0027624	0.008686202	1.104993556	-0.045689712	0.964178115	0.999227185	-5.021650889
hsa-mir-4706	MI0017339	0.008518216	0.961029024	-0.075903779	0.940528424	0.999227185	-5.020523681
hsa-mir-3171	MI0014202	0.008511307	0.963510463	-0.119571378	0.906456369	0.999227185	-5.017906245
hsa-mir-892c	MI0022560	0.008475563	0.956116315	-0.069943483	0.945189915	0.999227185	-5.020790362
hsa-mir-647	MI0003662	0.008416905	0.856927417	-0.07180756	0.943731807	0.999227185	-5.020709298
hsa-miR-6842-3p	MIMAT0027587	0.008405043	0.874702169	-0.125725981	0.901667843	0.999227185	-5.017443539
hsa-miR-4470	MIMAT0018997	0.008358384	0.840821615	-0.111494296	0.912746341	0.999227185	-5.018478347

hsa-mir-4266	MI0015870	0.008291572	0.994780335	-0.087649752	0.93134877	0.999227185	-5.019934411
hsa-miR-612	MIMAT0003280	0.008288023	0.936769096	-0.107840191	0.915593963	0.999227185	-5.01872406
hsa-miR-23a-3p	MIMAT0000078	0.008273399	12.12188949	-0.051386295	0.959715816	0.999227185	-5.021481191
hsa-mir-3919	MI0016425	0.008258689	0.902552852	-0.151627398	0.881561062	0.998806144	-5.01524272
hsa-miR-642b-5p	MIMAT0022736	0.008139034	0.884425114	-0.10290909	0.919438634	0.999227185	-5.019042692
hsa-mir-4508	MI0016872	0.00811289	0.975582684	-0.102021449	0.920130933	0.999227185	-5.019098468
hsa-mir-20b	MI0001519	0.008089678	0.91071939	-0.113672592	0.911049394	0.999227185	-5.018327986
hsa-miR-6868-5p	MIMAT0027636	0.008084432	0.795664142	-0.111494324	0.912746319	0.999227185	-5.018478345
hsa-miR-518f-5p	MIMAT0002841	0.008079178	0.839349689	-0.115571891	0.909570158	0.999227185	-5.018194516
hsa-miR-5693	MIMAT0022486	0.007981805	0.805314256	-0.1139665	0.910820467	0.999227185	-5.018307476
hsa-mir-924	MI0005716	0.007924633	1.004406242	-0.091649143	0.928225422	0.999227185	-5.019714482
hsa-miR-4522	MIMAT0019060	0.00789636	0.868500456	-0.095845818	0.924949318	0.999227185	-5.019473174
hsa-mir-1262	MI0006397	0.007832045	1.119483867	-0.064079101	0.94977842	0.999227185	-5.021031497
hsa-mir-4773-2	MI0017416	0.00774507	0.820504224	-0.101766014	0.920330167	0.999227185	-5.019114429
hsa-miR-3133	MIMAT0014998	0.007720946	0.94073251	-0.138065712	0.892079143	0.999057213	-5.01644612
hsa-mir-6823	MI0022668	0.007670534	0.860214952	-0.118708675	0.907127891	0.999227185	-5.017969253
hsa-miR-184	MIMAT0000454	0.007633293	0.905939894	-0.150471112	0.882456976	0.998806144	-5.015349699
hsa-miR-4452	MIMAT0018974	0.007597883	0.913457857	-0.109373485	0.914398928	0.999227185	-5.018621951
hsa-miR-3681-5p	MIMAT0018108	0.007537848	0.926271373	-0.069888856	0.945232648	0.999227185	-5.020792705
hsa-miR-5000-5p	MIMAT0021019	0.007489547	0.96218489	-0.063734373	0.950048206	0.999227185	-5.021045015
hsa-miR-4495	MIMAT0019030	0.007396548	0.902679447	-0.106631296	0.916536314	0.999227185	-5.018803551
hsa-miR-4308	MIMAT0016861	0.007372008	0.853484864	-0.076546843	0.940025622	0.999227185	-5.020493607
hsa-miR-555	MIMAT0003219	0.007347049	0.949225112	-0.104045908	0.918552094	0.999227185	-5.018970554
hsa-mir-4473	MI0016825	0.007281844	0.911725565	-0.094126662	0.926291197	0.999227185	-5.019573329
hsa-miR-3117-3p	MIMAT0014979	0.007250861	0.836153412	-0.080583125	0.936870322	0.999227185	-5.020299058
hsa-mir-376a-2	MI0003529	0.007241554	0.879846512	-0.096822086	0.924187401	0.999227185	-5.019415492
hsa-miR-29b-1-5p	MIMAT0004514	0.007239699	0.985391909	-0.06326287	0.950417218	0.999227185	-5.021063387
hsa-miR-3180-5p	MIMAT0015057	0.00707504	0.905824989	-0.098976016	0.922506664	0.999227185	-5.019286167
hsa-mir-6742	MI0022587	0.007012539	1.128800166	-0.067104567	0.947410945	0.999227185	-5.020909727
hsa-mir-378b	MI0014154	0.006995296	0.913245734	-0.113481415	0.911198308	0.999227185	-5.018341299
hsa-mir-377	MI0000785	0.006893843	0.861638726	-0.075627089	0.940744772	0.999227185	-5.020536543
hsa-mir-106a	MI0000113	0.006886718	0.887082887	-0.095937934	0.924877424	0.999227185	-5.019467756
hsa-miR-6848-3p	MIMAT0027597	0.006821842	0.870783286	-0.066974387	0.947512802	0.999227185	-5.020915082
hsa-miR-449c-5p	MIMAT0010251	0.006776127	0.885276052	-0.107841979	0.91559257	0.999227185	-5.018723942
hsa-miR-7157-5p	MIMAT0028224	0.006697966	0.850241656	-0.10927694	0.914474168	0.999227185	-5.018628423
hsa-miR-3153	MIMAT0015026	0.006650212	1.130454463	-0.071722508	0.943798331	0.999227185	-5.020713043
hsa-miR-4798-5p	MIMAT0019974	0.006636001	0.867789347	-0.056070136	0.956047867	0.999227185	-5.02132675
hsa-mir-3173	MI0014204	0.006535338	0.946402541	-0.063586937	0.950163592	0.999227185	-5.021050775
hsa-miR-4654	MIMAT0019720	0.006412305	1.11580104	-0.072910635	0.942869063	0.999227185	-5.020660325
hsa-mir-8076	MI0025912	0.006246328	0.893992977	-0.086188974	0.932489869	0.999227185	-5.020012297
hsa-miR-4782-3p	MIMAT0019945	0.006192699	0.830349446	-0.087897563	0.931155206	0.999227185	-5.019921068
hsa-mir-218-2	MI0000295	0.00615399	0.931142111	-0.075814215	0.940598455	0.999227185	-5.02052785
hsa-mir-4519	MI0016885	0.006068714	0.896900367	-0.092457444	0.927594321	0.999227185	-5.019668844
hsa-mir-214	MI0000290	0.006004966	0.990074339	-0.060087276	0.952902832	0.999227185	-5.021183573
hsa-mir-5001	MI0017867	0.006004814	1.009518962	-0.073496075	0.942411206	0.999227185	-5.02063403
hsa-miR-217-5p	MIMAT0000274	0.005975986	0.925003828	-0.088784758	0.930462259	0.999227185	-5.019872992
hsa-miR-649	MIMAT0003319	0.005971787	0.818981574	-0.114147134	0.910679773	0.999227185	-5.018294845
hsa-miR-937-3p	MIMAT0004980	0.00594594	0.960405058	-0.067741972	0.94691223	0.999227185	-5.020883356
hsa-miR-1287-5p	MIMAT0005878	0.005756244	0.824795117	-0.064630472	0.949346925	0.999227185	-5.021009723
hsa-mir-6514	MI0022226	0.005755991	0.984557741	-0.090861778	0.928840224	0.999227185	-5.019758554
hsa-miR-7152-3p	MIMAT0028215	0.005725464	0.905977652	-0.057755775	0.954728078	0.999227185	-5.021267877
hsa-miR-148b-5p	MIMAT0004699	0.005587702	0.951295518	-0.069104365	0.945846352	0.999227185	-5.020826157
hsa-miR-4482-5p	MIMAT0019016	0.005541069	1.036903764	-0.049697104	0.96103887	0.999227185	-5.021533588
hsa-miR-4529-3p	MIMAT0019068	0.005463137	1.390144432	-0.03467358	0.972810754	0.999227185	-5.021922576
hsa-mir-145	MI0000461	0.005433582	0.933253678	-0.081559661	0.936107093	0.999227185	-5.020250489
hsa-mir-4701	MI0017334	0.005342705	1.423072768	-0.033972149	0.973360552	0.999227185	-5.021937353
hsa-miR-1207-3p	MIMAT0005872	0.005319526	0.840807047	-0.06710194	0.947413	0.999227185	-5.020909835
hsa-miR-876-3p	MIMAT0004925	0.005108521	0.853753802	-0.049692439	0.961042524	0.999227185	-5.02153373
hsa-miR-516a-5p	MIMAT0004770	0.005040671	0.90438489	-0.061343425	0.951919551	0.999227185	-5.021136771
hsa-mir-564	MI0003570	0.005034061	0.947591763	-0.05684325	0.955442532	0.999227185	-5.021299964
hsa-mir-6069	MI0020346	0.005003168	0.944919095	-0.053102834	0.958371468	0.999227185	-5.021426153
hsa-mir-548q	MI0010637	0.004985993	0.94683499	-0.063550159	0.950192376	0.999227185	-5.021052209
hsa-mir-6832	MI0022677	0.004912262	0.930464267	-0.051537144	0.95959767	0.999227185	-5.021476427
hsa-mir-3691	MI0016092	0.004876667	0.977274039	-0.03613417	0.971665956	0.999227185	-5.021890836
hsa-mir-3152	MI0014179	0.004806073	0.917570359	-0.061975724	0.951424635	0.999227185	-5.021112847
hsa-miR-3922-3p	MIMAT0018197	0.004753489	0.761451229	-0.071162133	0.944236646	0.999227185	-5.020737607

hsa-miR-155-3p	MIMAT0004658	0.004697603	0.863773274	-0.063625875	0.950133118	0.999227185	-5.021049255
hsa-miR-874-3p	MIMAT0004911	0.00469547	1.612066994	-0.023702385	0.981411684	0.999227185	-5.022119139
hsa-miR-889-3p	MIMAT0004921	0.004607658	0.876289273	-0.085570724	0.932972865	0.999227185	-5.020044867
hsa-mir-448	MI0001637	0.004541852	0.95409184	-0.060109127	0.952885726	0.999227185	-5.021182768
hsa-mir-4293	MI0015826	0.004486649	0.926771654	-0.050039489	0.960770688	0.999227185	-5.021523109
hsa-miR-512-5p	MIMAT0002822	0.004477749	0.883790132	-0.061150682	0.95207042	0.999227185	-5.021144016
hsa-mir-593	MI0003605	0.004253918	0.949627127	-0.053641574	0.957949568	0.999227185	-5.021408507
hsa-miR-6783-5p	MIMAT0027466	0.004250256	1.074938614	-0.047717533	0.962589514	0.999227185	-5.021592764
hsa-miR-2276-3p	MIMAT0011775	0.004232631	0.972813918	-0.03256393	0.974464389	0.999227185	-5.02196611
hsa-mir-204	MI0000284	0.004195845	0.981936181	-0.078737539	0.938312947	0.999227185	-5.020389254
hsa-miR-3127-3p	MIMAT0019201	0.004117441	0.910733668	-0.039774718	0.968812812	0.999227185	-5.021806026
hsa-miR-219b-3p	MIMAT0019748	0.004114908	0.940257149	-0.048514018	0.961965589	0.999227185	-5.021569243
hsa-mir-6744	MI0022589	0.004055409	0.749049011	-0.057443344	0.954972689	0.999227185	-5.02127892
hsa-miR-1208	MIMAT0005873	0.004045838	0.839427416	-0.06757074	0.947046202	0.999227185	-5.020890465
hsa-mir-3914-2	MI0016421	0.003987623	0.938194122	-0.044651986	0.964991133	0.999227185	-5.021679659
hsa-miR-6743-3p	MIMAT0027388	0.003789303	0.980483046	-0.044730718	0.964929449	0.999227185	-5.021677499
hsa-mir-8077	MI0025913	0.003788643	1.103239639	-0.035324598	0.972300484	0.999227185	-5.02190859
hsa-mir-4484	MI0016845	0.003738795	0.850336407	-0.06275381	0.950815636	0.999227185	-5.02108307
hsa-miR-4525	MIMAT0019064	0.003723704	0.972794655	-0.050023507	0.960783207	0.999227185	-5.021523599
hsa-miR-3909	MIMAT0018183	0.003637114	0.750267113	-0.047125842	0.963053029	0.999227185	-5.021609985
hsa-mir-4772	MI0017414	0.003588587	0.938542525	-0.051349952	0.959744281	0.999227185	-5.021482337
hsa-miR-6516-3p	MIMAT0030418	0.00353069	0.944762981	-0.045254143	0.964519362	0.999227185	-5.021663045
hsa-mir-557	MI0003563	0.00340091	0.98740757	-0.045005912	0.964713841	0.999227185	-5.021669921
hsa-mir-4687	MI0017319	0.003360329	0.957592147	-0.049911156	0.960871208	0.999227185	-5.021527045
hsa-miR-6720-3p	MIMAT0025851	0.003284326	0.748780149	-0.041595462	0.967386035	0.999227185	-5.021760559
hsa-mir-4646	MI0017273	0.003272848	0.958412582	-0.058524944	0.954125895	0.999227185	-5.021240433
hsa-miR-4762-3p	MIMAT0019911	0.003213454	0.812296709	-0.040424604	0.968303533	0.999227185	-5.02179003
hsa-miR-563	MIMAT0003227	0.003030547	0.950369178	-0.020139332	0.984205526	0.999227185	-5.022167084
hsa-mir-4443	MI0016786	0.002987441	1.100065682	-0.031297106	0.975457439	0.999227185	-5.021990939
hsa-miR-1468-3p	MIMAT0026638	0.002896722	1.066704538	-0.028199962	0.977885431	0.999227185	-5.022047494
hsa-mir-1181	MI0006274	0.002688147	0.903736011	-0.034947238	0.972596259	0.999227185	-5.021916729
hsa-miR-548at-3p	MIMAT0022278	0.002541363	0.923242557	-0.034828254	0.972689519	0.999227185	-5.021919277
hsa-mir-520f	MI0003146	0.002508672	0.874492481	-0.031056	0.975646444	0.999227185	-5.021995553
hsa-miR-302b-3p	MIMAT0000715	0.002483928	0.866265279	-0.022682205	0.982211597	0.999227185	-5.022133663
hsa-miR-2115-5p	MIMAT0011158	0.002454093	0.858489878	-0.027858251	0.978153328	0.999227185	-5.022053373
hsa-mir-4436a	MI0016776	0.002321257	1.064426174	-0.025300264	0.980158843	0.999227185	-5.022095107
hsa-miR-10a-3p	MIMAT0004555	0.002151445	0.800141225	-0.035645126	0.972049258	0.999227185	-5.021901609
hsa-miR-5704	MIMAT0022498	0.002102591	0.930115716	-0.021917193	0.98281145	0.999227185	-5.022144135
hsa-miR-1178-3p	MIMAT0005823	0.002055829	0.711108777	-0.02928451	0.977035178	0.999227185	-5.022028359
hsa-miR-3194-3p	MIMAT0019218	0.002037603	0.863660272	-0.024160298	0.981052645	0.999227185	-5.022112412
hsa-miR-921	MIMAT0004971	0.001995834	0.887235752	-0.026636585	0.979111121	0.999227185	-5.022073806
hsa-mir-3688-1	MI0016089	0.001954321	0.85356492	-0.017817397	0.986026308	0.999227185	-5.022194134
hsa-miR-422a	MIMAT0001339	0.001906897	1.245078917	-0.014314166	0.98877357	0.999227185	-5.022228684
hsa-let-7f-2-3p	MIMAT0004487	0.001750774	0.9194196	-0.018120051	0.985788972	0.999227185	-5.022190796
hsa-mir-4757	MI0017398	0.00166903	0.823527312	-0.025908948	0.979681608	0.999227185	-5.02208554
hsa-mir-922	MI0005714	0.00166709	1.004819892	-0.019510537	0.984698597	0.999227185	-5.022174736
hsa-mir-346	MI0000826	0.001638541	0.979969409	-0.025713041	0.979835207	0.999227185	-5.022088644
hsa-miR-2682-5p	MIMAT0013517	0.001529951	0.910055883	-0.015436528	0.987893387	0.999227185	-5.022218435
hsa-mir-608	MI0003621	0.001518427	1.035610476	-0.01292437	0.989863502	0.999227185	-5.022240303
hsa-mir-129-1	MI0000252	0.001412212	0.879163688	-0.015620015	0.987749493	0.999227185	-5.022216686
hsa-miR-664b-3p	MIMAT0022272	0.001386935	0.977173903	-0.017260469	0.986463044	0.999227185	-5.02220013
hsa-miR-6840-5p	MIMAT0027582	0.001233433	1.15527371	-0.012053277	0.990546658	0.999227185	-5.022246981
hsa-miR-764	MIMAT0010367	0.001206633	0.846400225	-0.013699451	0.989255651	0.999227185	-5.022233969
hsa-miR-487b-5p	MIMAT0026614	0.001145511	0.7740022	-0.014155049	0.988898354	0.999227185	-5.022230074
hsa-miR-451b	MIMAT0019840	0.001118564	0.862314925	-0.01729502	0.98643595	0.999227185	-5.022199764
hsa-miR-548ad-3p	MIMAT0018946	0.001117441	0.973049615	-0.015467014	0.98786948	0.999227185	-5.022218146
hsa-miR-147a	MIMAT0000251	0.00109712	0.889680692	-0.014651045	0.98850938	0.999227185	-5.022225689
hsa-mir-3189	MI0014233	0.001061856	0.831698996	-0.014228131	0.988841041	0.999227185	-5.022229437
hsa-miR-6744-5p	MIMAT0027389	0.000992791	0.924593513	-0.011919403	0.990651649	0.999227185	-5.022247966
hsa-mir-5692c-2	MI0019289	0.000909139	0.898154412	-0.013547006	0.989375205	0.999227185	-5.022235244
hsa-miR-4289	MIMAT0016920	0.00089279	0.853628371	-0.009700524	0.992391844	0.999227185	-5.022262691
hsa-miR-548al	MIMAT0019024	0.000799656	0.850688115	-0.01239478	0.990278833	0.999227185	-5.022244419
hsa-mir-32	MI0000090	0.000773631	1.063153859	-0.010469138	0.99178904	0.999227185	-5.022257932
hsa-miR-33a-3p	MIMAT0004506	0.00073239	0.904164882	-0.010041776	0.992124209	0.999227185	-5.022260623
hsa-mir-3065	MI0014228	0.000561377	0.92367564	-0.006269287	0.995082928	0.999801366	-5.022279509
hsa-miR-4760-3p	MIMAT0019907	0.000492128	0.989067204	-0.004938572	0.996126613	0.999973228	-5.022284087

hsa-mir-4676	MI0017307	0.000388518	0.972172288	-0.003924374	0.996922056	0.999973228	-5.022286846
hsa-mir-3201	MI0014250	0.000235789	1.087383913	-0.002308255	0.998189598	0.999973228	-5.022289938
hsa-miR-655-5p	MIMAT0026626	0.000216098	0.909262913	-0.00200161	0.998430105	0.999973228	-5.022290344
hsa-miR-378a-3p	MIMAT0000732	6.83E-05	4.118938234	-0.000179391	0.999859301	0.999973228	-5.022291563
hsa-mir-1587	MI0016905	6.04E-05	0.867170748	-0.000561005	0.999559995	0.999973228	-5.022291477
hsa-miR-877-5p	MIMAT0004949	3.19E-05	3.766983805	-6.92E-05	0.999945721	0.999973228	-5.022291572
hsa-miR-6755-3p	MIMAT0027411	2.74E-05	0.768790847	0.000365829	0.999713074	0.999973228	-5.022291532
hsa-miR-1276	MIMAT0005930	7.86E-05	0.941233892	0.000870306	0.999317405	0.999973228	-5.022291341
hsa-miR-139-3p	MIMAT0004552	9.74E-05	2.002924895	0.000407891	0.999680085	0.999973228	-5.022291522
hsa-mir-5187	MI0018166	0.000108282	0.872052163	0.001273328	0.999001308	0.999973228	-5.022291076
hsa-mir-548ab	MI0016752	0.000216462	0.887653501	0.002643655	0.997926539	0.999973228	-5.022289428
hsa-mir-137	MI0000454	0.000548477	0.898467795	0.006977464	0.994527506	0.999801366	-5.02227663
hsa-miR-3618	MIMAT0017998	0.000570617	0.868418217	0.007201398	0.994351876	0.999742062	-5.022275656
hsa-miR-5692a	MIMAT0022484	0.000665345	0.94142136	0.009225023	0.99276477	0.999260685	-5.022265453
hsa-miR-365b-5p	MIMAT0022833	0.000762243	0.936689592	0.01195363	0.990624807	0.999227185	-5.022247715
hsa-miR-597-3p	MIMAT0026619	0.000789469	0.710159901	0.015925287	0.987510096	0.999227185	-5.02221373
hsa-miR-617	MIMAT0003286	0.00085423	1.042651059	0.007375359	0.994215439	0.999715483	-5.022274877
hsa-miR-6834-5p	MIMAT0027568	0.000888826	1.018022487	0.010786178	0.991540395	0.999227185	-5.022255864
hsa-miR-4652-3p	MIMAT0019717	0.000896821	0.960087554	0.011329039	0.991114649	0.999227185	-5.022252179
hsa-miR-6874-5p	MIMAT0027648	0.000897429	0.990834673	0.009249765	0.992745365	0.999260685	-5.022265312
hsa-miR-6841-5p	MIMAT0027584	0.000991086	0.878663152	0.013512073	0.9894026	0.999227185	-5.022235534
hsa-miR-5092	MIMAT0021084	0.000994568	0.938582005	0.010032709	0.99213132	0.999227185	-5.022260679
hsa-miR-3678-3p	MIMAT0018103	0.0011327	0.769654775	0.015090573	0.988164692	0.999227185	-5.022221676
hsa-miR-6816-3p	MIMAT0027533	0.001252025	0.844246343	0.010965426	0.991399817	0.999227185	-5.022254667
hsa-mir-631	MI0003645	0.001325903	1.019103911	0.014805015	0.988388632	0.999227185	-5.022224297
hsa-let-7b-3p	MIMAT0004482	0.001343488	1.019536435	0.01456536	0.988576576	0.999227185	-5.022226457
hsa-miR-1587	MIMAT0019077	0.001363341	4.213544788	0.002620699	0.997944544	0.999973228	-5.022289465
hsa-mir-302d	MI0000774	0.001431268	0.856947834	0.016945643	0.986709929	0.999227185	-5.022203436
hsa-miR-6134	MIMAT0024618	0.001526234	0.999330683	0.010872774	0.991472481	0.999227185	-5.022255288
hsa-mir-7106	MI0022957	0.001541969	0.86610767	0.022378995	0.982449346	0.999227185	-5.022137856
hsa-miR-128-2-5p	MIMAT0031095	0.001587446	0.803799702	0.017478614	0.986291977	0.999227185	-5.022197804
hsa-miR-6788-5p	MIMAT0027476	0.001608138	0.883264904	0.022771442	0.982141627	0.999227185	-5.022132418
hsa-miR-1322	MIMAT0005953	0.001609887	0.814395737	0.012253527	0.990389611	0.999227185	-5.022245487
hsa-mir-5699	MI0019306	0.001624934	1.163334207	0.014236708	0.988834315	0.999227185	-5.022229363
hsa-miR-4772-5p	MIMAT0019926	0.00164752	0.858198947	0.015219439	0.988063633	0.999227185	-5.022220478
hsa-mir-21	MI0000077	0.001674412	1.068779321	0.016443011	0.987104093	0.999227185	-5.022208587
hsa-mir-153-1	MI0000463	0.001733438	0.853827969	0.028146171	0.977927602	0.999227185	-5.022048424
hsa-mir-7515	MI0024354	0.00178808	0.986258513	0.024740696	0.980597574	0.999227185	-5.022103701
hsa-mir-548f-3	MI0006376	0.001798462	0.927636211	0.018137993	0.985774902	0.999227185	-5.022190596
hsa-mir-6781	MI0022626	0.00186766	0.816956968	0.024141138	0.981067667	0.999227185	-5.022112696
hsa-miR-371a-5p	MIMAT0004687	0.00187786	1.175197837	0.011566553	0.990928376	0.999227185	-5.02225051
hsa-miR-4503	MIMAT0019039	0.001882485	0.949455482	0.022782108	0.982133264	0.999227185	-5.022132269
hsa-miR-6792-3p	MIMAT0027485	0.001962609	1.093982349	0.029682438	0.976723221	0.999227185	-5.022021158
hsa-mir-3646	MI0016046	0.002010329	1.014731781	0.02614342	0.979497775	0.999227185	-5.022081795
hsa-mir-1281	MI0006428	0.002019426	0.990333319	0.027929714	0.978097302	0.999227185	-5.022052149
hsa-miR-4446-5p	MIMAT0019233	0.002146225	0.885441691	0.032781351	0.974293959	0.999227185	-5.021961749
hsa-miR-216b-3p	MIMAT0026721	0.002296916	0.845675138	0.025589642	0.979931957	0.999227185	-5.022090587
hsa-mir-3200	MI0014249	0.002344239	0.903207682	0.031877558	0.975002423	0.999227185	-5.021979684
hsa-miR-6734-3p	MIMAT0027370	0.002417232	0.997855955	0.02780978	0.978191329	0.999227185	-5.022054201
hsa-miR-181a-3p	MIMAT0000270	0.002424627	0.907410104	0.029978391	0.97649121	0.999227185	-5.022015739
hsa-miR-3129-3p	MIMAT0019202	0.002484123	0.883482833	0.026916601	0.978891584	0.999227185	-5.022069203
hsa-mir-1260a	MI0006394	0.002484536	1.129910841	0.02111876	0.983437519	0.999227185	-5.022154681
hsa-miR-3182	MIMAT0015062	0.002504462	0.752021877	0.044376711	0.965206808	0.999227185	-5.02168718
hsa-mir-3174	MI0014208	0.002541667	0.899646216	0.03130054	0.975454747	0.999227185	-5.021990873
hsa-miR-6499-3p	MIMAT0025451	0.00256607	0.797754255	0.042457938	0.966710219	0.999227185	-5.021738311
hsa-mir-4289	MI0015898	0.00258882	0.874167908	0.036632904	0.971275067	0.999227185	-5.021879698
hsa-miR-944	MIMAT0004987	0.002627997	1.016954267	0.023893021	0.981262209	0.999227185	-5.022116354
hsa-miR-4754	MIMAT0019894	0.002746593	0.941744561	0.032147188	0.974791064	0.999227185	-5.021974386
hsa-miR-664a-3p	MIMAT0005949	0.002789308	1.074407583	0.025370976	0.980103401	0.999227185	-5.022094007
hsa-mir-1827	MI0008195	0.002814046	0.981773706	0.035096711	0.972479101	0.999227185	-5.021913516
hsa-miR-214-3p	MIMAT0000271	0.002979916	1.36492856	0.017544373	0.986240409	0.999227185	-5.022197098
hsa-miR-6513-3p	MIMAT0025483	0.003026402	0.956124176	0.030538275	0.976052299	0.999227185	-5.02200534
hsa-miR-7-5p	MIMAT0000252	0.003034782	1.09816903	0.037318413	0.970737804	0.999227185	-5.02186414
hsa-miR-4294	MIMAT0016849	0.003063814	0.895450631	0.036081574	0.971707179	0.999227185	-5.021892002
hsa-miR-1539	MIMAT0007401	0.003084095	0.832573469	0.037761336	0.970390673	0.999227185	-5.021853935
hsa-mir-193a	MI0000487	0.003127509	0.89332837	0.039660534	0.968902293	0.999227185	-5.021808809

hsa-miR-6855-5p	MIMAT0027610	0.003173928	1.147504886	0.027176487	0.97868783	0.999227185	-5.022064889
hsa-mir-5088	MI0017977	0.003195041	0.964419782	0.045186598	0.964572281	0.999227185	-5.02166492
hsa-miR-5684	MIMAT0022473	0.003330954	0.905016344	0.036804765	0.971140371	0.999227185	-5.021875825
hsa-miR-520f-3p	MIMAT0002830	0.003379268	0.934878635	0.061294692	0.951957697	0.999227185	-5.021138605
hsa-miR-4746-5p	MIMAT0019880	0.003469014	0.930019177	0.033738014	0.973544075	0.999227185	-5.021942219
hsa-mir-23c	MI0016010	0.003492135	1.004057095	0.036277301	0.971553774	0.999227185	-5.021887655
hsa-miR-4774-3p	MIMAT0019930	0.003505389	0.956319011	0.043837781	0.965629061	0.999227185	-5.021701769
hsa-miR-4315	MIMAT0016866	0.003555302	0.827271082	0.042673977	0.966540939	0.999227185	-5.021732667
hsa-miR-6501-5p	MIMAT0025458	0.003572192	0.778552639	0.043778669	0.965675376	0.999227185	-5.021703359
hsa-miR-4659a-3p	MIMAT0019727	0.003575363	0.840721133	0.046819441	0.963293061	0.999227185	-5.021618818
hsa-mir-548p	MI0006420	0.003709266	0.924871712	0.048009406	0.962360873	0.999227185	-5.02158419
hsa-miR-6767-5p	MIMAT0027434	0.003713712	0.968337671	0.031353928	0.975412895	0.999227185	-5.021989846
hsa-miR-2277-3p	MIMAT0011777	0.003774722	0.976627062	0.049902812	0.960877744	0.999227185	-5.0215273
hsa-mir-107	MI0000114	0.00380052	0.852444723	0.068426464	0.946376699	0.999227185	-5.020854761
hsa-mir-575	MI0003582	0.003801545	1.056203825	0.052214737	0.959066986	0.999227185	-5.021454854
hsa-mir-3199-2	MI0014248	0.003955092	0.889180146	0.057364498	0.95503442	0.999227185	-5.021281698
hsa-miR-6758-3p	MIMAT0027417	-0.004031458	0.893159656	0.057686582	0.954782251	0.999227185	-5.021270327
hsa-miR-4742-3p	MIMAT0019873	-0.004244945	0.836601948	0.047184378	0.963007173	0.999227185	-5.02160829
hsa-mir-504	MI0003189	-0.00434197	0.861917195	0.062479419	0.951030395	0.999227185	-5.021093613
hsa-miR-922	MIMAT0004972	-0.004408566	0.90670715	0.044994818	0.964722533	0.999227185	-5.021670228
hsa-mir-3689b	MI0016411	-0.004408726	1.182436434	0.033621339	0.97363553	0.999227185	-5.021944631
hsa-miR-6893-5p	MIMAT0027686	-0.004448368	1.497795246	0.020228613	0.984135518	0.999227185	-5.022165978
hsa-miR-761	MIMAT0010364	-0.004622937	0.87769323	0.046232733	0.963752695	0.999227185	-5.021635571
hsa-mir-1302-5	MI0006366	-0.004655405	0.962651359	0.050805395	0.960170791	0.999227185	-5.021499407
hsa-mir-3135a	MI0014156	-0.004699408	0.883344262	0.052752159	0.958646097	0.999227185	-5.021437544
hsa-miR-3183	MIMAT0015063	-0.004757243	0.834508186	0.054061297	0.957620882	0.999227185	-5.021394636
hsa-miR-593-3p	MIMAT0004802	-0.004795967	0.868237746	0.05142974	0.95968179	0.999227185	-5.02147982
hsa-mir-5092	MI0017981	-0.004859522	1.00867742	0.042452081	0.966714808	0.999227185	-5.021738464
hsa-mir-545	MI0003516	-0.004908007	0.931451038	0.045841253	0.964059392	0.999227185	-5.021646633
hsa-mir-580	MI0003587	-0.004998285	0.888318346	0.081781224	0.935933936	0.999227185	-5.020239388
hsa-miR-652-3p	MIMAT0003322	-0.00506227	8.198748477	0.021983576	0.982759398	0.999227185	-5.02214324
hsa-miR-548ap-5p	MIMAT0021037	-0.005090302	1.084061749	0.057630532	0.954826134	0.999227185	-5.021272311
hsa-mir-4673	MI0017304	-0.005103851	1.156973193	0.0565001	0.955711209	0.999227185	-5.021311898
hsa-mir-6794	MI0022639	-0.005138422	0.887397514	0.055596972	0.956418359	0.999227185	-5.021342963
hsa-miR-6507-5p	MIMAT0025470	-0.005187538	1.09140226	0.046645007	0.963429713	0.999227185	-5.021623821
hsa-miR-6500-5p	MIMAT0025454	-0.005230208	0.807940577	0.076350205	0.940179367	0.999227185	-5.02050283
hsa-mir-320b-1	MI0003776	-0.005267801	0.981736999	0.054187623	0.957521957	0.999227185	-5.02139044
hsa-miR-670-3p	MIMAT0026640	-0.005297827	0.844499615	0.08060114	0.936856241	0.999227185	-5.020298167
hsa-mir-548i-2	MI0006422	-0.005408462	0.784862655	0.079400536	0.937794681	0.999227185	-5.020357093
hsa-miR-6778-3p	MIMAT0027457	-0.005486072	0.883497288	0.058342247	0.954268926	0.999227185	-5.021246985
hsa-mir-590	MI0003602	-0.005507167	0.926852851	0.060790897	0.952352046	0.999227185	-5.021157477
hsa-mir-4649	MI0017276	-0.005507641	0.965040097	0.073416918	0.942473111	0.999227185	-5.020637597
hsa-miR-302c-3p	MIMAT0000717	-0.005634029	0.904869033	0.064481885	0.949463206	0.999227185	-5.021015609
hsa-mir-4756	MI0017397	-0.005754092	0.895412117	0.054226925	0.957491181	0.999227185	-5.021389132
hsa-mir-383	MI0000791	-0.005942524	0.854044238	0.074518353	0.941611758	0.999227185	-5.020587611
hsa-miR-367-3p	MIMAT0000719	-0.005971183	0.983142728	0.095474721	0.925238956	0.999227185	-5.019494946
hsa-miR-6739-3p	MIMAT0027380	-0.006012844	0.852216566	0.0538211	0.95780898	0.999227185	-5.021402587
hsa-miR-6129	MIMAT0024613	-0.006013436	1.00286245	0.05607102	0.956047174	0.999227185	-5.02132672
hsa-mir-6758	MI0022603	-0.006066976	0.934568088	0.09370075	0.926623678	0.999227185	-5.019597862
hsa-mir-5009	MI0017877	-0.0060727	0.922692707	0.062077464	0.951345003	0.999227185	-5.021108974
hsa-miR-6074	MIMAT0023699	-0.006118328	1.036333763	0.053400153	0.958138629	0.999227185	-5.021416437
hsa-miR-6510-5p	MIMAT0025476	-0.006157552	2.19790761	0.01821804	0.985712132	0.999227185	-5.022189703
hsa-miR-6865-5p	MIMAT0027630	-0.006209788	1.717536396	0.035120114	0.972460758	0.999227185	-5.021913011
hsa-miR-564	MIMAT0003228	-0.006212052	1.015136878	0.064682833	0.94930595	0.999227185	-5.021007646
hsa-miR-4722-5p	MIMAT0019836	-0.006249479	1.431770127	0.053403995	0.95813562	0.999227185	-5.021416311
hsa-miR-616-5p	MIMAT0003284	-0.006250472	0.942582413	0.075634483	0.940738991	0.999227185	-5.0205362
hsa-mir-651	MI0003666	-0.006316148	1.018933314	0.077323385	0.93941849	0.999227185	-5.020456954
hsa-mir-4264	MI0015877	-0.006330306	0.97982736	0.08596694	0.932663325	0.999227185	-5.020024021
hsa-mir-7113	MI0022964	-0.006398388	0.963723178	0.087338677	0.931591756	0.999227185	-5.019951106
hsa-miR-6782-3p	MIMAT0027465	-0.006441067	0.949366443	0.072787675	0.94296523	0.999227185	-5.02066582
hsa-miR-6839-5p	MIMAT0027580	-0.00647567	0.8854882	0.053089241	0.958382114	0.999227185	-5.021426596
hsa-miR-7162-3p	MIMAT0028235	-0.006518066	0.880504567	0.105362191	0.917525739	0.999227185	-5.01888604
hsa-miR-490-5p	MIMAT0004764	-0.006533728	0.933672464	0.099721243	0.921925244	0.999227185	-5.019240761
hsa-miR-183-3p	MIMAT0004560	-0.006702071	1.117173203	0.053661361	0.957934072	0.999227185	-5.021407856
hsa-mir-7110	MI0022961	-0.006706719	0.948705397	0.099759154	0.921895667	0.999227185	-5.019238442
hsa-miR-1206	MIMAT0005870	-0.006903517	0.809560285	0.08435817	0.933920231	0.999227185	-5.020108067

hsa-miR-3654	MIMAT0018074	-0.006978513	1.00534972	0.069694426	0.945384746	0.999227185	-5.020801031
hsa-mir-5700	MI0019307	-0.007077914	1.056733774	0.10562749	0.917318894	0.999227185	-5.018868878
hsa-mir-205	MI0000285	-0.007394324	0.935001282	0.104400456	0.918275625	0.999227185	-5.018947894
hsa-mir-29b-2	MI0000107	-0.007584876	0.90167864	0.111464279	0.912769728	0.999227185	-5.018480399
hsa-miR-296-5p	MIMAT0000690	-0.007783546	1.038952742	0.108394017	0.915162292	0.999227185	-5.018687344
hsa-miR-548as-5p	MIMAT0022267	-0.007817807	0.990090506	0.083317871	0.934733097	0.999227185	-5.02016157
hsa-miR-26a-2-3p	MIMAT0004681	-0.007836664	0.895256557	0.111079697	0.913069374	0.999227185	-5.018506636
hsa-mir-4716	MI0017350	-0.007863255	1.214342826	0.075321816	0.940983476	0.999227185	-5.020550679
hsa-miR-4783-3p	MIMAT0019947	-0.007905166	1.401326447	0.045581031	0.96426326	0.999227185	-5.021653933
hsa-mir-3921	MI0016428	-0.008302487	1.005990147	0.103354462	0.919091301	0.999227185	-5.019014524
hsa-miR-4309	MIMAT0016859	-0.008336871	0.813241	0.148756256	0.883785987	0.998806144	-5.015506854
hsa-miR-554	MIMAT0003217	-0.008348726	0.778401788	0.088322102	0.930823611	0.999227185	-5.019898123
hsa-miR-7160-3p	MIMAT0028231	-0.008352546	0.88015015	0.093436221	0.926830184	0.999227185	-5.019613044
hsa-miR-150-5p	MIMAT0000451	-0.008375219	7.388166508	0.02429459	0.98094735	0.999227185	-5.022110415
hsa-mir-6735	MI0022580	-0.008408718	0.918339495	0.108542662	0.915046438	0.999227185	-5.018677458
hsa-mir-4795	MI0017442	-0.008484061	0.988371983	0.093728929	0.92660168	0.999227185	-5.019596243
hsa-miR-155-5p	MIMAT0000646	-0.00850042	6.620520406	0.042295867	0.966837211	0.999227185	-5.021742527
hsa-miR-608	MIMAT0003276	-0.008507355	1.091059704	0.071626636	0.943873319	0.999227185	-5.020717259
hsa-miR-4728-3p	MIMAT0019850	-0.008533617	0.96252785	0.090830495	0.928864652	0.999227185	-5.019760297
hsa-miR-3908	MIMAT0018182	-0.008561115	0.954340437	0.179114236	0.860315807	0.997250847	-5.012459834
hsa-miR-8052	MIMAT0030979	-0.00862921	0.898572288	0.080611481	0.936848159	0.999227185	-5.020297656
hsa-miR-215-3p	MIMAT0026476	-0.008674697	0.735126027	0.093414057	0.926847487	0.999227185	-5.019614314
hsa-mir-636	MI0003651	-0.008679857	0.830180301	0.101142479	0.920816535	0.999227185	-5.019153224
hsa-miR-6880-5p	MIMAT0027660	-0.00871702	1.361657127	0.062766632	0.9508056	0.999227185	-5.021082576
hsa-mir-375	MI0000783	-0.008757415	0.895081552	0.134268162	0.895028279	0.999057213	-5.016762981
hsa-miR-5692c	MIMAT0022476	-0.008760659	1.003168588	0.108925408	0.914748133	0.999227185	-5.018651939
hsa-miR-718	MIMAT0012735	-0.008797059	1.095703315	0.08978562	0.929680602	0.999227185	-5.019818177
hsa-miR-4737	MIMAT0019863	-0.008864591	0.878024274	0.113218616	0.911403016	0.999227185	-5.018359562
hsa-miR-5001-3p	MIMAT0021022	-0.008938514	0.813904503	0.13373897	0.895439372	0.999057213	-5.016806437
hsa-miR-523-3p	MIMAT0002840	-0.008953962	0.866657069	0.117214466	0.908291147	0.999227185	-5.018077307
hsa-mir-4736	MI0017373	-0.008958847	0.922086391	0.119733398	0.906330262	0.999227185	-5.017894361
hsa-mir-4452	MI0016798	-0.008995148	0.927878732	0.147393573	0.884842323	0.998806144	-5.015630456
hsa-mir-5589	MI0019148	-0.009024559	0.842797328	0.14858392	0.883919567	0.998806144	-5.015522548
hsa-mir-361	MI0000760	-0.009027233	5.632385396	0.070929204	0.944418844	0.999227185	-5.02074776
hsa-mir-4794	MI0017441	-0.009049545	0.974351322	0.120757674	0.905533085	0.999227185	-5.01781886
hsa-mir-6501	MI0022213	-0.009072527	0.913068582	0.082391851	0.935456731	0.999227185	-5.020208638
hsa-miR-3151-3p	MIMAT0027026	-0.009074918	1.164611944	0.081553628	0.936111808	0.999227185	-5.020250791
hsa-miR-4755-5p	MIMAT0019895	-0.00907882	0.840157101	0.17135298	0.866304116	0.997250847	-5.013292222
hsa-mir-7706	MI0025242	-0.009147312	0.988457879	0.119308758	0.906660783	0.999227185	-5.017925474
hsa-mir-4262	MI0015872	-0.009149823	0.945540144	0.083442221	0.934635929	0.999227185	-5.02015521
hsa-miR-4700-5p	MIMAT0019796	-0.009182308	0.952884645	0.10640713	0.916711069	0.999227185	-5.018818193
hsa-miR-4313	MIMAT0016865	-0.009234443	1.266774725	0.074010475	0.942008923	0.999227185	-5.020610753
hsa-mir-652	MI0003667	-0.009243981	1.140720679	0.098614385	0.922788823	0.999227185	-5.019308078
hsa-miR-7977	MIMAT0031180	-0.009376681	1.425162821	0.056684498	0.95556683	0.999227185	-5.021305494
hsa-miR-3136-3p	MIMAT0019203	-0.009417491	0.929547403	0.089018103	0.930280014	0.999227185	-5.019860267
hsa-mir-22	MI0000078	-0.009454879	0.982711844	0.083595599	0.93451608	0.999227185	-5.020147351
hsa-mir-548h-4	MI0006414	-0.009467899	0.98479642	0.202095561	0.842637665	0.996080495	-5.009780405
hsa-mir-4301	MI0015828	-0.0097259	0.943847107	0.099249274	0.922293465	0.999227185	-5.019269557
hsa-miR-4472	MIMAT0018999	-0.00980011	1.142928978	0.066022871	0.948257331	0.999227185	-5.020953908
hsa-miR-374c-5p	MIMAT0018443	-0.009836603	1.103589706	0.107877822	0.915564632	0.999227185	-5.018721571
hsa-miR-5580-5p	MIMAT0022273	-0.009961834	0.82993642	0.091421164	0.928403431	0.999227185	-5.019727282
hsa-mir-6502	MI0022214	-0.010003228	0.83266485	0.108798054	0.914847389	0.999227185	-5.01866044
hsa-miR-3192-5p	MIMAT0015076	-0.010051525	1.004208445	0.09962794	0.921998036	0.999227185	-5.019246464
hsa-miR-6804-3p	MIMAT0027509	-0.010189628	1.070288704	0.103177025	0.919229677	0.999227185	-5.019025761
hsa-miR-6739-5p	MIMAT0027379	-0.010205429	0.977729856	0.140133792	0.890473786	0.998939026	-5.016269861
hsa-mir-1537	MI0007258	-0.010238888	1.03889339	0.108923975	0.91474925	0.999227185	-5.018652035
hsa-miR-3913-5p	MIMAT0018187	-0.010268562	0.773149423	0.130852939	0.897681876	0.999057213	-5.017040421
hsa-miR-4437	MIMAT0018953	-0.010289661	0.933320329	0.081038647	0.936514293	0.999227185	-5.020276475
hsa-miR-4747-5p	MIMAT0019882	-0.010297505	0.99557099	0.129222307	0.898949311	0.999157065	-5.017170374
hsa-mir-3143	MI0014167	-0.010338425	0.99785147	0.167796223	0.869051245	0.997250847	-5.013661433
hsa-mir-5193	MI0018172	-0.010369534	1.031520046	0.115481111	0.909640853	0.999227185	-5.018200945
hsa-mir-138-2	MI0000455	-0.010449298	0.867823335	0.13955191	0.890925425	0.998939026	-5.016319718
hsa-miR-548ah-5p	MIMAT0018972	-0.010472385	0.960915735	0.165749323	0.870633003	0.99760832	-5.013870422
hsa-miR-888-3p	MIMAT0004917	-0.010557135	0.817256397	0.113228118	0.911395614	0.999227185	-5.018358902
hsa-mir-380	MI0000788	-0.010573091	0.941663091	0.090846282	0.928852325	0.999227185	-5.019759418
hsa-mir-8057	MI0025893	-0.010702086	0.857685946	0.131095412	0.897493435	0.999057213	-5.017020958

hsa-miR-545-3p	MIMAT0003165	-0.010915642	0.883280957	0.154769853	0.879127074	0.998806144	-5.014947865
hsa-mir-549a	MI0003679	-0.010929683	0.939053318	0.16133064	0.874049517	0.998241091	-5.014312869
hsa-miR-219b-5p	MIMAT0019747	-0.010954913	0.910311287	0.131228016	0.897390382	0.999057213	-5.017010299
hsa-miR-383-3p	MIMAT0026485	-0.010996038	0.873673293	0.125050737	0.90219302	0.999227185	-5.017495435
hsa-miR-1178-5p	MIMAT0022940	-0.010999288	0.757753483	0.139775671	0.890751744	0.998939026	-5.01630057
hsa-miR-591	MIMAT0003259	-0.01102211	0.895163974	0.166428548	0.870108062	0.997398876	-5.013801356
hsa-mir-6086	MI0020363	-0.011039107	0.977798981	0.129410039	0.898803379	0.999149876	-5.017155496
hsa-miR-548ba	MIMAT0031175	-0.011060728	0.920353213	0.151030605	0.882023449	0.998806144	-5.015298037
hsa-miR-548ah-3p	MIMAT0020957	-0.011135376	0.813696257	0.130378358	0.898050723	0.999057213	-5.01707841
hsa-mir-4500	MI0016863	-0.011147742	0.953947904	0.120550146	0.905694592	0.999227185	-5.017834209
hsa-miR-125b-1-3p	MIMAT0004592	-0.011220336	1.068954812	0.082198449	0.935607872	0.999227185	-5.020218403
hsa-miR-6826-3p	MIMAT0027553	-0.011225543	0.980527589	0.135846536	0.893802334	0.999057213	-5.016632354
hsa-mir-6866	MI0022713	-0.011290625	0.979692958	0.137421723	0.892579143	0.999057213	-5.016500474
hsa-miR-376c-5p	MIMAT0022861	-0.011675712	0.740171542	0.210598676	0.836118351	0.996080495	-5.008707776
hsa-mir-660	MI0003684	-0.011754683	1.209560067	0.097924118	0.923327425	0.999227185	-5.019349679
hsa-miR-218-1-3p	MIMAT0004565	-0.011787228	0.925185344	0.118475935	0.907309067	0.999227185	-5.017986173
hsa-miR-1247-3p	MIMAT0022721	-0.011863581	1.466862062	0.067901234	0.946787624	0.999227185	-5.020876729
hsa-miR-630	MIMAT0003299	-0.011956149	0.900409877	0.116941461	0.908503708	0.999227185	-5.018096902
hsa-mir-4304	MI0015832	-0.012095239	0.850494179	0.213009214	0.834272439	0.996080495	-5.008395725
hsa-mir-1237	MI0006327	-0.012114556	0.961088788	0.145649901	0.886194327	0.998806144	-5.015786964
hsa-miR-3662	MIMAT0018083	-0.012282639	1.043517174	0.104893445	0.91789122	0.999227185	-5.018916258
hsa-miR-1295b-3p	MIMAT0022294	-0.012400909	1.003908612	0.083495829	0.934594039	0.999227185	-5.020152465
hsa-miR-4277	MIMAT0016908	-0.012476074	0.85154671	0.139644041	0.890853913	0.998939026	-5.016311837
hsa-miR-1255b-2-3p	MIMAT0022725	-0.012479877	0.909517807	0.123082678	0.903723965	0.999227185	-5.0176451
hsa-mir-578	MI0003585	-0.012504313	0.971948518	0.194852058	0.848200681	0.996080495	-5.010659549
hsa-miR-3126-5p	MIMAT0014989	-0.012532285	0.929998155	0.121022114	0.905327293	0.999227185	-5.017799263
hsa-mir-4424	MI0016763	-0.012537294	1.070862508	0.141894972	0.889107052	0.998806144	-5.0161177
hsa-mir-5008	MI0017876	-0.012549002	0.957851728	0.168402996	0.868582467	0.997250847	-5.013598992
hsa-miR-615-3p	MIMAT0003283	-0.01259992	0.772371388	0.129820174	0.898484576	0.999057213	-5.017122916
hsa-miR-5585-5p	MIMAT0022285	-0.012650014	0.859594346	0.175179524	0.863350605	0.997250847	-5.012886408
hsa-mir-4710	MI0017344	-0.012707894	0.851212544	0.245739207	0.80931322	0.994603723	-5.00381085
hsa-miR-942-5p	MIMAT0004985	-0.012734993	0.765273353	0.139534033	0.890939301	0.998939026	-5.016321246
hsa-miR-3667-5p	MIMAT0018089	-0.012806151	1.178851103	0.104656318	0.918076116	0.999227185	-5.018931493
hsa-mir-4453	MI0016799	-0.012867794	0.86929672	0.112095182	0.912278191	0.999227185	-5.018437159
hsa-mir-125b-1	MI0000446	-0.01293797	1.002657188	0.122964873	0.903815618	0.999227185	-5.017653984
hsa-mir-3662	MI0016063	-0.012938004	0.978134165	0.199487948	0.84463933	0.996080495	-5.010100558
hsa-miR-143-5p	MIMAT0004599	-0.012940349	0.815878298	0.1360664	0.893631584	0.999057213	-5.016614037
hsa-miR-4660	MIMAT0019728	-0.013008099	0.801042607	0.217187036	0.831075594	0.996080495	-5.007846559
hsa-mir-3941	MI0016598	-0.013144318	0.996376248	0.106092336	0.916956483	0.999227185	-5.018838703
hsa-mir-761	MI0003941	-0.013163632	0.89258837	0.145498808	0.886311498	0.998806144	-5.015800439
hsa-miR-5190	MIMAT0021121	-0.013199758	0.956799292	0.176219637	0.862548159	0.997250847	-5.012774562
hsa-miR-96-5p	MIMAT0000095	-0.013227942	0.905289968	0.183570158	0.856881761	0.996937676	-5.011965385
hsa-mir-6856	MI0022702	-0.013234576	0.85912939	0.180657789	0.859125903	0.996937676	-5.012289921
hsa-miR-5087	MIMAT0021079	-0.013235013	0.89280847	0.194569604	0.848417777	0.996080495	-5.010693185
hsa-miR-5591-3p	MIMAT0022302	-0.013237326	0.946070395	0.1260859	0.901387933	0.999227185	-5.017415764
hsa-miR-3681-3p	MIMAT0018109	-0.013246208	0.956385	0.148418166	0.884048049	0.998806144	-5.015537626
hsa-mir-1182	MI0006275	-0.01325302	0.879091397	0.20328788	0.841722786	0.996080495	-5.009632642
hsa-miR-7157-3p	MIMAT0028225	-0.013268498	0.75342105	0.266626864	0.793494262	0.994214304	-5.000546983
hsa-miR-4524a-3p	MIMAT0019063	-0.013278602	0.869553097	0.193407124	0.849311403	0.996080495	-5.010831111
hsa-mir-6820	MI0022665	-0.013282921	0.95527291	0.206453724	0.839294751	0.996080495	-5.009236118
hsa-miR-6885-3p	MIMAT0027671	-0.013477258	0.982206105	0.17010582	0.867267182	0.997250847	-5.013422561
hsa-mir-4746	MI0017385	-0.013512589	0.996044291	0.13021423	0.898178289	0.999057213	-5.017091516
hsa-mir-627	MI0003641	-0.013587534	0.866499437	0.22664302	0.823851425	0.995693701	-5.006564573
hsa-mir-450a-2	MI0003187	-0.013600794	1.017156358	0.143622232	0.887766996	0.998806144	-5.01596663
hsa-miR-6889-3p	MIMAT0027679	-0.013712005	1.044195604	0.072417419	0.943254811	0.999227185	-5.020682314
hsa-mir-4713	MI0017347	-0.013795794	0.957836969	0.125499331	0.901844117	0.999227185	-5.017460989
hsa-mir-95	MI0000097	-0.013907129	0.839936296	0.206236407	0.839461368	0.996080495	-5.009263531
hsa-mir-4726	MI0017363	-0.013946405	1.091237002	0.120280901	0.905904137	0.999227185	-5.017854083
hsa-mir-8087	MI0025923	-0.013954615	0.928069034	0.273964473	0.787959059	0.994214304	-4.999338125
hsa-mir-874	MI0005532	-0.013981852	0.999624192	0.153807144	0.879872607	0.998806144	-5.015038836
hsa-miR-3978	MIMAT0019363	-0.013989398	1.348910915	0.141865676	0.889129784	0.998806144	-5.016120246
hsa-miR-2054	MIMAT0009979	-0.014041324	0.907232066	0.200417242	0.843925854	0.996080495	-5.009986936
hsa-miR-4638-3p	MIMAT0019696	-0.014071852	0.963482693	0.203181536	0.841804375	0.996080495	-5.009645856
hsa-miR-450a-5p	MIMAT0001545	-0.014131022	0.807467015	0.21791691	0.830517414	0.996080495	-5.007749534
hsa-miR-135a-3p	MIMAT0004595	-0.014187584	1.481463094	0.070027011	0.945124573	0.999227185	-5.020786775
hsa-miR-6132	MIMAT0024616	-0.014261385	4.224316054	0.03406348	0.973288964	0.999227185	-5.021935446

hsa-mir-372	MI0000780	-0.014285647	0.856582097	0.186273593	0.854799759	0.996937676	-5.011659517
hsa-miR-5680	MIMAT0022468	-0.014448468	0.934797465	0.152372119	0.880984126	0.998806144	-5.015173388
hsa-miR-92b-3p	MIMAT0003218	-0.014454081	1.962005692	0.05468636	0.957131408	0.999227185	-5.021373778
hsa-mir-5000	MI0017866	-0.014555747	0.945348972	0.2271904	0.823433736	0.995590185	-5.006488706
hsa-miR-3680-3p	MIMAT0018107	-0.014690821	0.884034847	0.176036088	0.862689756	0.997250847	-5.012794347
hsa-miR-675-5p	MIMAT0004284	-0.01472368	1.025311272	0.1840352	0.856523539	0.996937676	-5.011913086
hsa-miR-6810-5p	MIMAT0027520	-0.014737842	1.353758592	0.082332372	0.935503213	0.999227185	-5.020211644
hsa-miR-495-5p	MIMAT0022924	-0.014810418	0.880158787	0.27622534	0.786255919	0.994214304	-4.998959134
hsa-let-7a-3	MI0000062	-0.014829681	0.881287657	0.212323928	0.834797106	0.996080495	-5.008484796
hsa-miR-6869-3p	MIMAT0027639	-0.014863894	0.835153653	0.125991721	0.901461175	0.999227185	-5.017423039
hsa-miR-5088-3p	MIMAT0027041	-0.01489303	0.843359717	0.196586912	0.846867538	0.996080495	-5.010451889
hsa-miR-582-3p	MIMAT0004797	-0.014906593	0.941571973	0.199101066	0.844936405	0.996080495	-5.010147707
hsa-miR-4324	MIMAT0016876	-0.014983895	0.850132493	0.166765957	0.869847319	0.997357345	-5.013766942
hsa-miR-7156-5p	MIMAT0028222	-0.014996415	0.797843534	0.219021035	0.829673201	0.996080495	-5.007602146
hsa-miR-8062	MIMAT0030989	-0.015023049	0.952068491	0.192964734	0.849651536	0.996080495	-5.010883384
hsa-miR-4661-3p	MIMAT0019730	-0.015127789	0.892819404	0.168884352	0.86821062	0.997250847	-5.013549297
hsa-miR-6726-3p	MIMAT0027354	-0.015193373	0.915242165	0.192257644	0.850195246	0.996080495	-5.010966687
hsa-miR-6881-3p	MIMAT0027663	-0.015227468	0.869929088	0.118550882	0.907250725	0.999227185	-5.017980728
hsa-miR-1286	MIMAT0005877	-0.015267395	0.771790391	0.17490831	0.863559872	0.997250847	-5.012915464
hsa-miR-3121-3p	MIMAT0014983	-0.0153038	1.033481583	0.172973401	0.865053142	0.997250847	-5.01312146
hsa-mir-2392	MI0016870	-0.015405526	1.026466011	0.177481815	0.861574601	0.997250847	-5.012637953
hsa-mir-5094	MI0017983	-0.015444986	1.020459846	0.19847604	0.845416395	0.996080495	-5.010223686
hsa-mir-764	MI0003944	-0.015606613	0.905692708	0.153956179	0.879757185	0.998806144	-5.01502479
hsa-miR-6804-5p	MIMAT0027508	-0.015613448	1.139590879	0.194740835	0.848286167	0.996080495	-5.0106728
hsa-miR-8066	MIMAT0030993	-0.015620817	0.814805494	0.34468656	0.735256656	0.989764242	-4.986035304
hsa-miR-558	MIMAT0003222	-0.015645947	0.758876725	0.211119125	0.835719723	0.996080495	-5.0086407
hsa-miR-1301-3p	MIMAT0005797	-0.015656683	3.958289153	0.032808097	0.974272993	0.999227185	-5.021961211
hsa-mir-571	MI0003578	-0.015709311	0.955768405	0.171263062	0.866373543	0.997250847	-5.013301651
hsa-mir-4518	MI0016884	-0.015811439	0.967068784	0.163901622	0.87206132	0.997852043	-5.014056881
hsa-mir-4274	MI0015884	-0.015816046	0.887631078	0.201763524	0.842892482	0.996080495	-5.0098214
hsa-miR-1269b	MIMAT0019059	-0.015839426	0.846972444	0.143136652	0.888143687	0.998806144	-5.016009284
hsa-miR-329-5p	MIMAT0026555	-0.015886282	0.931254696	0.120071422	0.906067172	0.999227185	-5.017869515
hsa-mir-875	MI0005541	-0.016004829	0.945873766	0.20508158	0.840346907	0.996080495	-5.009408726
hsa-mir-6841	MI0022687	-0.016018968	0.870759908	0.208604496	0.837646185	0.996080495	-5.008963267
hsa-mir-4665	MI0017295	-0.0160498	0.970982392	0.241935168	0.81220368	0.994603723	-5.004376963
hsa-mir-4762	MI0017403	-0.016094851	0.908995581	0.228390055	0.82251851	0.995221754	-5.0063218
hsa-mir-30c-1	MI0000736	-0.016286407	0.938102974	0.202665185	0.842200556	0.996080495	-5.009709919
hsa-miR-4733-5p	MIMAT0019857	-0.016389202	1.004689045	0.226514108	0.823949801	0.995779492	-5.006582413
hsa-miR-3144-5p	MIMAT0015014	-0.016403509	0.930112577	0.184498145	0.856166964	0.996937676	-5.011860893
hsa-miR-6763-3p	MIMAT0027427	-0.016483672	1.064951298	0.163392537	0.872454935	0.997852043	-5.014107889
hsa-mir-8080	MI0025916	-0.01651451	0.913473634	0.282938108	0.781205796	0.994214304	-4.997815792
hsa-miR-887-5p	MIMAT0026720	-0.01659729	0.746661044	0.200817726	0.843618421	0.996080495	-5.009937808
hsa-miR-1251-3p	MIMAT0026741	-0.016677798	0.745009195	0.25621835	0.801365739	0.994214304	-5.002206237
hsa-miR-1270	MIMAT0005924	-0.016683125	1.060054708	0.183837611	0.856675737	0.996937676	-5.011935323
hsa-miR-5700	MIMAT0022493	-0.016700902	0.988321636	0.294425226	0.772587598	0.994214304	-4.995796606
hsa-miR-7109-3p	MIMAT0028116	-0.016717476	1.01491718	0.161414301	0.873984806	0.998241091	-5.014304603
hsa-miR-3975	MIMAT0019360	-0.01677074	0.866688015	0.219570506	0.829253157	0.996080495	-5.007528523
hsa-miR-6726-5p	MIMAT0027353	-0.016770811	1.365319212	0.083792528	0.934362201	0.999227185	-5.02013724
hsa-mir-570	MI0003577	-0.016828064	1.060428454	0.170551182	0.866923245	0.997250847	-5.013376126
hsa-mir-3908	MI0016412	-0.016834943	0.888480791	0.258571024	0.799584535	0.994214304	-5.001836892
hsa-mir-4502	MI0016865	-0.016853306	1.152770318	0.158596955	0.876164501	0.998806144	-5.014580641
hsa-mir-412	MI0002464	-0.016880687	0.968631095	0.221005396	0.828156508	0.996080495	-5.007335401
hsa-miR-553	MIMAT0003216	-0.017070737	0.999170062	0.200974716	0.843497915	0.996080495	-5.009918523
hsa-miR-6838-5p	MIMAT0027578	-0.017075728	0.950141952	0.229144692	0.821942929	0.994995229	-5.006216364
hsa-mir-3665	MI0016066	-0.017097822	0.803127328	0.233465644	0.818649275	0.994603723	-5.005606019
hsa-miR-7159-3p	MIMAT0028229	-0.017142864	0.891913103	0.245026383	0.809854635	0.994603723	-5.003917596
hsa-miR-6767-3p	MIMAT0027435	-0.017155084	0.830304456	0.146514676	0.885523753	0.998806144	-5.015709576
hsa-miR-920	MIMAT0004970	-0.01721326	0.95983765	0.212336845	0.834787216	0.996080495	-5.008483119
hsa-miR-499a-5p	MIMAT0002870	-0.017236431	1.00963677	0.193647765	0.849126399	0.996080495	-5.010802627
hsa-miR-627-3p	MIMAT0026623	-0.017257225	0.768258224	0.241275307	0.812705359	0.994603723	-5.004474274
hsa-miR-6737-3p	MIMAT0027376	-0.017257896	0.958291921	0.17152456	0.866171637	0.997250847	-5.013274216
hsa-mir-4456	MI0016802	-0.017275527	0.864163984	0.209992857	0.836582425	0.996080495	-5.008785647
hsa-mir-151a	MI0000809	-0.017283549	1.069737811	0.167960343	0.868924445	0.997250847	-5.013644566
hsa-miR-4502	MIMAT0019038	-0.017345787	1.267920759	0.086027257	0.932616204	0.999227185	-5.020020839
hsa-miR-508-5p	MIMAT0004778	-0.01737968	0.925344002	0.256995569	0.800777181	0.994214304	-5.00208459
hsa-mir-5087	MI0017976	-0.017388126	0.841808773	0.221013436	0.828150364	0.996080495	-5.007334315

hsa-miR-4764-5p	MIMAT0019914	-0.017416979	0.937533685	0.214345986	0.833249216	0.996080495	-5.008221159
hsa-miR-369-3p	MIMAT0000721	-0.017438703	0.958553528	0.163651961	0.872254349	0.997852043	-5.014081916
hsa-mir-6082	MI0020359	-0.017593088	0.840789809	0.205188745	0.840264722	0.996080495	-5.009395286
hsa-miR-141-3p	MIMAT0000432	-0.017645488	0.982157809	0.219960978	0.828954693	0.996080495	-5.007476092
hsa-miR-1264	MIMAT0005791	-0.017855902	0.838678095	0.313502452	0.758343741	0.99324537	-4.992268857
hsa-miR-130b-5p	MIMAT0004680	-0.018090388	0.909621006	0.210314201	0.836336259	0.996080495	-5.00874437
hsa-mir-6861	MI0022708	-0.018185606	0.903135963	0.262318838	0.796749456	0.994214304	-5.001241645
hsa-mir-892b	MI0005538	-0.018261813	0.980995222	0.214149915	0.833399278	0.996080495	-5.008246832
hsa-miR-4311	MIMAT0016863	-0.018329191	0.94884918	0.157433595	0.877064861	0.998806144	-5.014693214
hsa-miR-759	MIMAT0010497	-0.018403282	0.828461202	0.247818386	0.807734589	0.994603723	-5.00349774
hsa-mir-6513	MI0022225	-0.018495674	0.90622392	0.2572741	0.800566291	0.994214304	-5.002040907
hsa-mir-630	MI0003644	-0.018618824	0.996382783	0.234094969	0.818169865	0.994603723	-5.005516184
hsa-miR-8077	MIMAT0031004	-0.018686751	0.998786215	0.23301179	0.818995062	0.994603723	-5.005670657
hsa-mir-3681	MI0016082	-0.018822767	0.984568522	0.192924091	0.849682785	0.996080495	-5.01088818
hsa-miR-943	MIMAT0004986	-0.018824706	0.805447049	0.208909427	0.837412519	0.996080495	-5.008924355
hsa-miR-548an	MIMAT0019079	-0.01883589	0.878524925	0.238311197	0.814959953	0.994603723	-5.004908157
hsa-miR-1246	MIMAT0005898	-0.018929404	4.912145784	0.035176548	0.972416525	0.999227185	-5.021911794
hsa-mir-18a	MI0000072	-0.018959272	0.946512909	0.192249277	0.85020168	0.996080495	-5.010967671
hsa-miR-1298-3p	MIMAT0026641	-0.018992044	0.90062688	0.163690462	0.872224581	0.997852043	-5.014078057
hsa-miR-518a-3p	MIMAT0002863	-0.019009217	0.894631823	0.190008193	0.851925465	0.996778901	-5.011229679
hsa-mir-499b	MI0017396	-0.019068448	0.86653456	0.232720817	0.819216771	0.994603723	-5.005712032
hsa-miR-4510	MIMAT0019047	-0.01908301	1.06329703	0.146676553	0.885398239	0.998806144	-5.015695039
hsa-mir-4529	MI0016896	-0.019124242	1.056130387	0.238651537	0.814700994	0.994603723	-5.004858608
hsa-miR-1261	MIMAT0005913	-0.019378388	1.008924187	0.177377951	0.861654706	0.997250847	-5.012649231
hsa-miR-4712-3p	MIMAT0019819	-0.019470246	0.937723817	0.231976515	0.81978397	0.994603723	-5.005817636
hsa-miR-3177-5p	MIMAT0019215	-0.019496896	0.832385427	0.216235204	0.83180366	0.996080495	-5.007972605
hsa-miR-4797-5p	MIMAT0019972	-0.019562041	0.936407978	0.194558193	0.848426549	0.996080495	-5.010694543
hsa-miR-3614-3p	MIMAT0017993	-0.019569281	0.94362956	0.278925172	0.784223582	0.994214304	-4.998502539
hsa-mir-4313	MI0015843	-0.019696477	1.245501295	0.136073066	0.893626408	0.999057213	-5.016613481
hsa-mir-34a	MI0000268	-0.019725447	0.96255032	0.20027687	0.844033617	0.996080495	-5.010004132
hsa-miR-149-5p	MIMAT0000450	-0.01974891	0.895255681	0.200712121	0.843699487	0.996080495	-5.009950772
hsa-mir-4437	MI0016778	-0.0198853	0.904679976	0.275765316	0.786602369	0.994214304	-4.999036496
hsa-miR-200c-5p	MIMAT0004657	-0.01992286	0.83558932	0.251377103	0.805034621	0.994603723	-5.002955774
hsa-miR-1193	MIMAT0015049	-0.019996824	0.976428411	0.206397342	0.839337978	0.996080495	-5.009243233
hsa-mir-1279	MI0006426	-0.020143238	0.956142835	0.263659112	0.795736307	0.994214304	-5.001026724
hsa-mir-4759	MI0017400	-0.020215921	0.976220415	0.246901113	0.808430929	0.994603723	-5.003636196
hsa-mir-2115	MI0010634	-0.020255969	0.972987993	0.27261157	0.788978756	0.994214304	-4.999563445
hsa-miR-548t-5p	MIMAT0015009	-0.020303859	0.91670056	0.233477934	0.818639913	0.994603723	-5.005604267
hsa-miR-16-2-3p	MIMAT0004518	-0.020352924	0.894070523	0.208514389	0.837715236	0.996080495	-5.008974754
hsa-miR-3168	MIMAT0015043	-0.020358291	0.886856491	0.172672846	0.865285144	0.997250847	-5.013153254
hsa-mir-6837	MI0022683	-0.020401944	0.960913684	0.195827088	0.847451363	0.996080495	-5.010543064
hsa-mir-3661	MI0016062	-0.020418653	0.890727618	0.222147423	0.827283951	0.996080495	-5.007180805
hsa-mir-1178	MI0006271	-0.020492619	0.914769921	0.312463483	0.759117201	0.99324537	-4.992466578
hsa-miR-6817-3p	MIMAT0027535	-0.020534432	0.877829365	0.23677204	0.816131355	0.994603723	-5.005131364
hsa-miR-3150b-3p	MIMAT0018194	-0.020594849	0.924400616	0.219459708	0.829337853	0.996080495	-5.007543383
hsa-mir-3975	MI0016993	-0.020597166	1.028563295	0.352110971	0.729797796	0.989764242	-4.984466106
hsa-mir-129-2	MI0000473	-0.020746386	0.897421082	0.320431748	0.7531921	0.992561169	-4.99093372
hsa-miR-570-3p	MIMAT0003235	-0.020746676	0.845955701	0.297601816	0.770209773	0.994214304	-4.995224286
hsa-mir-4754	MI0017394	-0.020791466	1.084146016	0.174538351	0.863845348	0.997250847	-5.012955027
hsa-mir-3914-1	MI0016419	-0.020801224	1.05029989	0.242371082	0.811872311	0.994603723	-5.004312533
hsa-mir-6817	MI0022662	-0.020848261	0.968880869	0.269441278	0.7913698	0.994214304	-5.000087138
hsa-mir-1288	MI0006432	-0.02086972	0.881186766	0.245509627	0.809487583	0.994603723	-5.003845263
hsa-mir-198	MI0000240	-0.020938846	0.922527842	0.211868182	0.835146079	0.996080495	-5.008543874
hsa-miR-103a-3p	MIMAT0000101	-0.020959243	11.12722423	0.171716265	0.866023625	0.997250847	-5.013254078
hsa-miR-548ao-3p	MIMAT0021030	-0.020984521	0.921505298	0.191759687	0.850578193	0.99626475	-5.01102517
hsa-miR-3139	MIMAT0015007	-0.021103391	0.859181705	0.190991368	0.851169135	0.996603697	-5.01111511
hsa-mir-3622a	MI0016013	-0.021167263	0.926849317	0.278302467	0.784692188	0.994214304	-4.998608239
hsa-miR-656-3p	MIMAT0003332	-0.021228909	1.051888067	0.221888716	0.827481593	0.996080495	-5.007215896
hsa-miR-1292-5p	MIMAT0005943	-0.021250628	1.201432592	0.16361725	0.872281187	0.997852043	-5.014085393
hsa-mir-6821	MI0022666	-0.02130864	1.009267022	0.215946293	0.832024683	0.996080495	-5.008010756
hsa-mir-4431	MI0016771	-0.021394532	0.998759095	0.243808627	0.810779795	0.994603723	-5.004099247
hsa-miR-3976	MIMAT0019361	-0.021408542	1.017986045	0.272820753	0.788821066	0.994214304	-4.999528678
hsa-mir-18b	MI0001518	-0.021441116	0.9042259	0.220205566	0.828767752	0.996080495	-5.007443204
hsa-mir-4743	MI0017381	-0.021481244	0.96606077	0.342287115	0.737024051	0.989764242	-4.986535458
hsa-miR-4753-3p	MIMAT0019891	-0.021515774	0.876254338	0.311365729	0.759934714	0.99324537	-4.992674786
hsa-miR-552-3p	MIMAT0003215	-0.021543827	0.924073502	0.256355226	0.801262079	0.994214304	-5.00218484

hsa-mir-5707	MI0019315	-0.021549444	0.899804973	0.391476216	0.701112874	0.98572659	-4.975602154
hsa-mir-4474	MI0016826	-0.021596586	0.954242531	0.239064448	0.814386846	0.994603723	-5.004798399
hsa-miR-6857-5p	MIMAT0027614	-0.021732138	1.196874369	0.179359346	0.860126831	0.997192578	-5.012432949
hsa-mir-4700	MI0017333	-0.021751208	0.921006367	0.218364189	0.830175399	0.996080495	-5.007689916
hsa-miR-6733-3p	MIMAT0027368	-0.021812817	0.836535386	0.336528089	0.741272328	0.990306303	-4.987721967
hsa-mir-219a-2	MI0000740	-0.0218781	0.916489295	0.222846321	0.826750079	0.996080495	-5.007085807
hsa-miR-1912-3p	MIMAT0007887	-0.021886145	0.965838087	0.229998427	0.82129189	0.994979764	-5.006096666
hsa-miR-6504-3p	MIMAT0025465	-0.021892576	0.821221318	0.261843707	0.797108711	0.994214304	-5.001317575
hsa-mir-3166	MI0014196	-0.02212377	0.90379289	0.245838446	0.809237853	0.994603723	-5.003795964
hsa-miR-4499	MIMAT0019035	-0.022174262	1.075625578	0.162954718	0.872793478	0.997882552	-5.014151631
hsa-miR-4450	MIMAT0018971	-0.022217126	0.895897398	0.207808945	0.838255883	0.996080495	-5.009064519
hsa-mir-4718	MI0017353	-0.022278167	0.971483623	0.189711975	0.852153367	0.996778901	-5.011264082
hsa-miR-7156-3p	MIMAT0028223	-0.022292455	0.909718655	0.243252227	0.811202604	0.994603723	-5.004181947
hsa-mir-4513	MI0016879	-0.022305904	0.910044211	0.328147867	0.747469767	0.990537905	-4.989413326
hsa-mir-196b	MI0001150	-0.022322009	0.839909503	0.359428484	0.724432308	0.989068072	-4.982887577
hsa-miR-4486	MIMAT0019020	-0.022352248	2.851729348	0.052660137	0.958718165	0.999227185	-5.021440521
hsa-miR-5586-3p	MIMAT0022288	-0.022474923	0.818096841	0.340161696	0.738590889	0.989829051	-4.986975641
hsa-miR-1289	MIMAT0005879	-0.022479757	0.91521415	0.243618444	0.810924308	0.994603723	-5.004127536
hsa-miR-1294	MIMAT0005884	-0.022568718	0.975355455	0.17181762	0.865945373	0.997250847	-5.013243421
hsa-miR-6857-3p	MIMAT0027615	-0.0225885	0.828607788	0.1822414	0.857905485	0.996937676	-5.012114093
hsa-miR-101-3p	MIMAT0000099	-0.022836013	0.968641815	0.257382543	0.800484188	0.994214304	-5.002023887
hsa-miR-3616-3p	MIMAT0017996	-0.022837871	0.85826474	0.24473729	0.810074239	0.994603723	-5.0039608
hsa-mir-585	MI0003592	-0.022855433	0.948919657	0.383626966	0.706796731	0.986573137	-4.977442423
hsa-mir-891a	MI0005524	-0.022862749	1.041840888	0.210592666	0.836122955	0.996080495	-5.00870855
hsa-mir-3131	MI0014151	-0.022885624	0.912056954	0.26160941	0.797285885	0.994214304	-5.001354968
hsa-mir-4635	MI0017262	-0.023030331	0.85613792	0.347896144	0.732894947	0.989764242	-4.985360944
hsa-miR-4271	MIMAT0016901	-0.023033946	1.415452471	0.118228055	0.907502035	0.999227185	-5.018004158
hsa-miR-6073	MIMAT0023698	-0.023038071	0.792485944	0.292741195	0.773849132	0.994214304	-4.996097565
hsa-mir-6804	MI0022649	-0.023055004	1.027815471	0.335838516	0.741781595	0.990306303	-4.987862717
hsa-mir-6844	MI0022690	-0.023069458	0.883552904	0.312611136	0.759007265	0.99324537	-4.992438518
hsa-mir-8082	MI0025918	-0.023423709	0.928077466	0.295917008	0.771470635	0.994214304	-4.995528587
hsa-mir-4679-1	MI0017310	-0.023504514	0.885114059	0.345485104	0.734668802	0.989764242	-4.985868093
hsa-miR-6861-5p	MIMAT0027623	-0.023545894	2.097758014	0.053303171	0.958214578	0.999227185	-5.021419612
hsa-mir-6753	MI0022598	-0.023595756	1.003961402	0.238227379	0.815023732	0.994603723	-5.004920349
hsa-miR-6854-3p	MIMAT0027609	-0.023631472	0.771373774	0.285756973	0.779088142	0.994214304	-4.997327616
hsa-mir-1468	MI0003782	-0.023679531	0.921717645	0.350556975	0.730939144	0.989764242	-4.984797255
hsa-miR-4536-3p	MIMAT0020959	-0.023696329	0.861861686	0.210679481	0.836056457	0.996080495	-5.008697372
hsa-mir-184	MI0000481	-0.02369968	0.916910273	0.30613917	0.76383106	0.993908457	-4.993656232
hsa-mir-6843	MI0022689	-0.02376092	0.906888002	0.341200198	0.737825165	0.989764242	-4.986760897
hsa-mir-4310	MI0015840	-0.023867146	0.932407089	0.266315445	0.793729441	0.994214304	-5.000597573
hsa-mir-4735	MI0017372	-0.023874513	0.868251936	0.377505291	0.711242208	0.9869374	-4.978852524
hsa-miR-1288-5p	MIMAT0026743	-0.02390995	0.822927524	0.272857359	0.788793472	0.994214304	-4.999522592
hsa-mir-4319	MI0015848	-0.023986113	0.862354907	0.310667758	0.760454658	0.99324537	-4.992806795
hsa-mir-5194	MI0018173	-0.024027376	0.988484802	0.274353799	0.787665695	0.994214304	-4.99927308
hsa-miR-6873-3p	MIMAT0027647	-0.024063286	0.862282572	0.37128229	0.715772422	0.987260208	-4.980263355
hsa-miR-517-5p	MIMAT0002851	-0.024245932	0.998345416	0.258974072	0.799279503	0.994214304	-5.001773283
hsa-miR-4731-5p	MIMAT0019853	-0.024452007	0.821279188	0.32644241	0.748733237	0.990697422	-4.989752422
hsa-miR-4645-5p	MIMAT0019705	-0.024625394	0.851435103	0.300572503	0.767988236	0.994214304	-4.994683603
hsa-miR-24-1-5p	MIMAT0000079	-0.024649906	0.915984464	0.265903907	0.794040261	0.994214304	-5.000664338
hsa-mir-4440	MI0016783	-0.024654468	1.014473058	0.253886964	0.803131954	0.994214304	-5.002568951
hsa-miR-4695-3p	MIMAT0019789	-0.024724813	0.91081719	0.359889159	0.724095021	0.989068072	-4.982787142
hsa-mir-181a-1	MI0000289	-0.024785708	0.845851954	0.238968972	0.814459482	0.994603723	-5.00481233
hsa-miR-515-5p	MIMAT0002826	-0.024961235	1.00265619	0.387905787	0.703696051	0.986573137	-4.976443733
hsa-mir-938	MI0005760	-0.025000695	1.021212083	0.252219767	0.804395674	0.99449721	-5.002826325
hsa-miR-3691-5p	MIMAT0018120	-0.025066669	0.981106331	0.292208925	0.774248002	0.994214304	-4.996192336
hsa-mir-6720	MI0022555	-0.025115838	1.001870073	0.242165105	0.812028884	0.994603723	-5.004342991
hsa-miR-378e	MIMAT0018927	-0.025123118	0.878638527	0.277332395	0.785422371	0.994214304	-4.998772438
hsa-miR-4804-5p	MIMAT0019984	-0.025152308	1.02594812	0.346577606	0.73386483	0.989764242	-4.985638718
hsa-miR-4712-5p	MIMAT0019818	-0.025388038	0.778079446	0.235792762	0.816876887	0.994603723	-5.005272634
hsa-miR-520a-3p	MIMAT0002834	-0.025412018	0.894646581	0.39549698	0.698208451	0.985651988	-4.974645473
hsa-mir-8078	MI0025914	-0.025530294	0.914548035	0.25683546	0.800898415	0.994214304	-5.002109679
hsa-miR-1236-3p	MIMAT0005591	-0.025657949	0.941633407	0.232734639	0.819206238	0.994603723	-5.005710068
hsa-mir-4493	MI0016855	-0.025713632	0.931902899	0.264081196	0.795417322	0.994214304	-5.000958817
hsa-miR-4708-3p	MIMAT0019810	-0.02577669	1.043557266	0.200538369	0.843832868	0.996080495	-5.009972087
hsa-miR-548d-5p	MIMAT0004812	-0.025802344	1.019076314	0.294949042	0.772195334	0.994214304	-4.995702647
hsa-mir-7152	MI0023612	-0.025890605	0.984951595	0.316802126	0.755889074	0.993091273	-4.991636645

hsa-miR-182-3p	MIMAT0000260	-0.02597783	0.891812178	0.41200289	0.686336872	0.98554565	-4.970618922
hsa-mir-6888	MI0022735	-0.026137597	0.876670883	0.252967502	0.803828828	0.994214304	-5.0027111
hsa-miR-3907	MIMAT0018179	-0.026181563	1.311762432	0.164824515	0.871347843	0.997852043	-5.013964008
hsa-miR-548u	MIMAT0015013	-0.026280327	0.915166958	0.212310457	0.834807421	0.996080495	-5.008486544
hsa-miR-6852-3p	MIMAT0027605	-0.026305857	0.783585472	0.243909085	0.810703464	0.994603723	-5.004084296
hsa-miR-3186-5p	MIMAT0015067	-0.026352141	0.794379977	0.272291736	0.789219876	0.994214304	-4.999616552
hsa-miR-3202	MIMAT0015089	-0.026411003	0.998374633	0.194830291	0.848217411	0.996080495	-5.010662143
hsa-mir-548b	MI0003596	-0.026458764	0.901442695	0.28210833	0.781829504	0.994214304	-4.997958586
hsa-miR-539-3p	MIMAT0022705	-0.026462439	0.980009069	0.373033086	0.714496751	0.986960752	-4.979868734
hsa-miR-4304	MIMAT0016854	-0.026484536	0.826876916	0.337244609	0.740743292	0.990306303	-4.987575417
hsa-mir-1193	MI0014205	-0.026511155	0.888781063	0.388674797	0.703139357	0.986573137	-4.976263103
hsa-miR-940	MIMAT0004983	-0.026554028	2.683682264	0.086511651	0.932237793	0.999227185	-5.019995205
hsa-mir-146a	MI0000477	-0.026556661	0.847760921	0.277673193	0.785165826	0.994214304	-4.998714817
hsa-mir-568	MI0003574	-0.026563495	0.945237317	0.368229643	0.717998751	0.987694154	-4.980947084
hsa-miR-6716-5p	MIMAT0025844	-0.026567182	3.897418089	0.038004417	0.970200167	0.999227185	-5.021848282
hsa-miR-27a-5p	MIMAT0004501	-0.026648076	0.982923953	0.369616906	0.716986675	0.987445697	-4.980637047
hsa-miR-424-5p	MIMAT0001341	-0.026693145	0.795494452	0.375321555	0.712830648	0.9869374	-4.979350202
hsa-mir-4682	MI0017314	-0.026914971	0.954835344	0.380393286	0.70914362	0.986865843	-4.978190034
hsa-miR-548m	MIMAT0005917	-0.027208171	0.79843708	0.406344933	0.690396822	0.98554565	-4.972017111
hsa-mir-133a-2	MI0000451	-0.027350609	0.913988895	0.332466009	0.744274078	0.990306303	-4.988547018
hsa-miR-122-3p	MIMAT0004590	-0.027396346	0.926592465	0.302799328	0.766324354	0.994181122	-4.994274847
hsa-miR-3609	MIMAT0017986	-0.027469421	1.064952721	0.517511621	0.612569165	0.976708039	-4.941150551
hsa-miR-6872-5p	MIMAT0027644	-0.02753357	0.892407328	0.317512699	0.755360826	0.993032785	-4.991499651
hsa-miR-365a-5p	MIMAT0009199	-0.0277287	1.269473313	0.235469137	0.817123306	0.994603723	-5.005319193
hsa-miR-1301-5p	MIMAT0026639	-0.027762417	0.823127425	0.342461829	0.736895307	0.989764242	-4.986499154
hsa-miR-3162-3p	MIMAT0019213	-0.02786255	1.766385947	0.173590753	0.864576642	0.997250847	-5.013055983
hsa-miR-548ak	MIMAT0019013	-0.027890862	0.899598259	0.541951392	0.596046433	0.972142131	-4.933416727
hsa-miR-520d-5p	MIMAT0002855	-0.027892695	0.93588135	0.282626633	0.7814399	0.994214304	-4.997869441
hsa-miR-655-3p	MIMAT0003331	-0.027999007	0.977189458	0.486466958	0.633874536	0.98055505	-4.950485618
hsa-miR-1273h-3p	MIMAT0030416	-0.028380242	1.12152429	0.284605089	0.77995327	0.994214304	-4.997527677
hsa-miR-5682	MIMAT0022470	-0.028442464	0.798533866	0.358641388	0.725008726	0.989104742	-4.983058889
hsa-miR-4464	MIMAT0018988	-0.028545056	0.931095883	0.409279459	0.68828987	0.98554565	-4.971294269
hsa-miR-4420	MIMAT0018933	-0.028590283	0.871805151	0.500535888	0.624176107	0.978652564	-4.946323183
hsa-miR-499b-5p	MIMAT0019897	-0.028614497	0.86864199	0.332187977	0.744479693	0.990306303	-4.988603131
hsa-miR-646	MIMAT0003316	-0.02863548	0.921877263	0.433566353	0.67095617	0.984005175	-4.965119024
hsa-miR-1304-3p	MIMAT0022720	-0.028785213	1.197921132	0.209817101	0.836717071	0.996080495	-5.008808197
hsa-mir-4489	MI0016850	-0.028925857	1.011482961	0.342954835	0.736532064	0.989764242	-4.986396617
hsa-mir-4688	MI0017321	-0.029103634	0.906016224	0.276224785	0.786256337	0.994214304	-4.998959227
hsa-miR-583	MIMAT0003248	-0.029175989	0.976362192	0.422612853	0.678750543	0.985064407	-4.967946625
hsa-miR-2467-3p	MIMAT0019953	-0.029199959	0.971793909	0.299222605	0.768997457	0.994214304	-4.994929947
hsa-miR-548av-3p	MIMAT0022304	-0.029201445	0.895013822	0.328216091	0.74741924	0.990537905	-4.989399725
hsa-miR-502-5p	MIMAT0002873	-0.029228231	0.910909747	0.296149719	0.771296441	0.994214304	-4.995486657
hsa-mir-4742	MI0017380	-0.029264935	0.855640187	0.38005062	0.709392494	0.986886843	-4.978268896
hsa-miR-2117	MIMAT0011162	-0.029484663	0.701565412	0.594521527	0.561285816	0.96620986	-4.915645357
hsa-miR-5581-5p	MIMAT0022275	-0.029611593	0.892474263	0.358942255	0.724788371	0.989068072	-4.982993449
hsa-mir-5591	MI0019151	-0.029651514	0.926487646	0.385031295	0.705778477	0.986573137	-4.977115834
hsa-mir-624	MI0003638	-0.029683543	0.903343975	0.416548615	0.68308225	0.98554565	-4.969482044
hsa-mir-675	MI0005416	-0.029683891	0.846048051	0.509388777	0.618109915	0.977302963	-4.943646088
hsa-miR-7852-3p	MIMAT0030427	-0.029704876	0.980019708	0.240365697	0.813397055	0.994603723	-5.004607985
hsa-mir-6818	MI0022663	-0.030008148	0.932692334	0.531186976	0.603296162	0.974392455	-4.936864649
hsa-miR-6503-5p	MIMAT0025462	-0.030021264	1.113400543	0.229667344	0.82154435	0.994979764	-5.006143138
hsa-miR-126-5p	MIMAT0000444	-0.030074389	0.908247914	0.3704775	0.716359106	0.987260208	-4.980444144
hsa-mir-5587	MI0019144	-0.03011268	0.992760902	0.388801891	0.70304737	0.986573137	-4.976233218
hsa-mir-148a	MI0000253	-0.030142881	1.007517977	0.351035245	0.730587804	0.989764242	-4.98469549
hsa-miR-4672	MIMAT0019754	-0.030199224	0.899834087	0.404385734	0.691804981	0.98554565	-4.972496904
hsa-mir-4768	MI0017409	-0.030315687	0.94164962	0.291954062	0.774439014	0.994214304	-4.996237655
hsa-miR-3167	MIMAT0015042	-0.030378122	0.926094216	0.475068212	0.641783707	0.982649571	-4.953774978
hsa-miR-192-3p	MIMAT0004543	-0.030382712	0.830658052	0.465317749	0.648585096	0.982649571	-4.956529479
hsa-mir-548ag-1	MI0016793	-0.030469626	0.947112152	0.361135309	0.723182935	0.988677831	-4.982514829
hsa-mir-3155a	MI0014183	-0.030507744	1.007824568	0.273002186	0.788684303	0.994214304	-4.999498503
hsa-miR-3163	MIMAT0015037	-0.030520225	1.053599888	0.286651767	0.778416313	0.994214304	-4.997171658
hsa-miR-668-3p	MIMAT0003881	-0.030614237	0.958861693	0.263272496	0.796028522	0.994214304	-5.001088831
hsa-miR-1283	MIMAT0005799	-0.0307219	0.828525612	0.339882192	0.738797026	0.989829051	-4.987033328
hsa-miR-1234-3p	MIMAT0005589	-0.030723989	1.211018766	0.212224784	0.834873019	0.996080495	-5.008497658
hsa-miR-6882-3p	MIMAT0027665	-0.030915412	0.878055845	0.3991593	0.695567198	0.985651988	-4.973765831
hsa-mir-223	MI0000300	-0.030994995	0.994010167	0.467129548	0.647318805	0.982649571	-4.956021784

hsa-mir-152	MI0000462	-0.031054	0.984155894	0.352019025	0.729865308	0.989764242	-4.984485739
hsa-miR-488-3p	MIMAT0004763	-0.031070673	0.865918396	0.402468458	0.69318415	0.98554565	-4.972964256
hsa-mir-219a-1	MI0000296	-0.031137835	0.868319761	0.328443814	0.747250594	0.990470453	-4.989354307
hsa-miR-6130	MIMAT0024614	-0.031275437	0.963197522	0.266126381	0.79387223	0.994214304	-5.000628258
hsa-mir-561	MI0003567	-0.031402365	0.890214875	0.450422702	0.659037773	0.984005175	-4.960631564
hsa-mir-3157	MI0014185	-0.031447831	1.003519827	0.29750733	0.770280467	0.994214304	-4.995241397
hsa-miR-1287-3p	MIMAT0026738	-0.031470453	0.886511982	0.422136159	0.679090625	0.985064407	-4.968068097
hsa-miR-34c-5p	MIMAT0000686	-0.03148164	0.862494893	0.274642178	0.787448417	0.994214304	-4.999224842
hsa-miR-4514	MIMAT0019051	-0.031510932	1.075972175	0.299279045	0.768955252	0.994214304	-4.994919669
hsa-mir-4521	MI0016887	-0.031546692	0.847696311	0.230256169	0.821095369	0.994909671	-5.006060443
hsa-miR-1204	MIMAT0005868	-0.031556032	0.769025295	0.453401145	0.656941642	0.984005175	-4.959821549
hsa-miR-6878-5p	MIMAT0027656	-0.031589359	0.961098408	0.395570963	0.698155055	0.985651988	-4.974627781
hsa-mir-378g	MI0016761	-0.031608321	0.936659677	0.505426788	0.620821234	0.97746608	-4.944849701
hsa-miR-2053	MIMAT0009978	-0.031618326	0.918210915	0.278548192	0.784507262	0.994214304	-4.998566556
hsa-mir-487a	MI0002471	-0.031619925	1.1117056	0.365809145	0.719765932	0.98846477	-4.981485318
hsa-mir-4717	MI0017352	-0.031675414	1.017963728	0.318772684	0.754424449	0.992853492	-4.991255994
hsa-mir-6819	MI0022664	-0.031719191	1.127175839	0.248910847	0.806905477	0.994603723	-5.003332178
hsa-mir-4770	MI0017411	-0.031751711	0.861225251	0.510718764	0.617201046	0.97695399	-4.94324005
hsa-miR-93-3p	MIMAT0004509	-0.031760486	2.482249549	0.109357745	0.914411195	0.999227185	-5.018623007
hsa-miR-514a-5p	MIMAT0022702	-0.031831654	0.844767479	0.343764039	0.735935988	0.989764242	-4.986228003
hsa-mir-592	MI0003604	-0.031902647	0.897588764	0.358119236	0.725391212	0.989104742	-4.983172333
hsa-miR-4749-3p	MIMAT0019886	-0.032015588	1.515997867	0.174236628	0.864078184	0.997250847	-5.012987232
hsa-mir-644a	MI0003659	-0.032270049	0.906740631	0.320977532	0.752786844	0.992468979	-4.990827343
hsa-miR-571	MIMAT0003236	-0.032280083	0.811726264	0.475408329	0.641547055	0.982649571	-4.953677909
hsa-miR-7853-5p	MIMAT0030428	-0.03233886	0.75527747	0.562827388	0.582113046	0.968332399	-4.926544296
hsa-miR-8080	MIMAT0031007	-0.032528218	1.052081865	0.333829065	0.74326634	0.990306303	-4.988271259
hsa-mir-548o	MI0006402	-0.03261828	0.903028601	0.400870771	0.694334287	0.98554565	-4.973352063
hsa-mir-4488	MI0016849	-0.032749873	1.128162017	0.308943379	0.761739721	0.99324537	-4.993131683
hsa-mir-4776-2	MI0017420	-0.03279996	0.924991059	0.343585119	0.736067768	0.989764242	-4.986265318
hsa-mir-4428	MI0016767	-0.032925827	0.868145796	0.370518224	0.716329414	0.987260208	-4.980435005
hsa-miR-8085	MIMAT0031012	-0.033033086	1.418479014	0.161367306	0.874021156	0.998241091	-5.014309247
hsa-mir-4666b	MI0019299	-0.033120596	0.833122857	0.577516655	0.572410669	0.967644566	-4.921562425
hsa-mir-302b	MI0000772	-0.033125771	0.8750614	0.505759458	0.620593357	0.97746608	-4.944748983
hsa-mir-196a-2	MI0000279	-0.033143112	0.840287244	0.547117864	0.592582515	0.970056246	-4.931738689
hsa-miR-6078	MIMAT0023703	-0.033292649	0.77926665	0.408442568	0.688890476	0.98554565	-4.971500927
hsa-miR-5590-5p	MIMAT0022299	-0.033313419	0.869368991	0.459470238	0.652679655	0.983005937	-4.958155143
hsa-miR-4676-5p	MIMAT0019758	-0.033399857	0.914404647	0.358903489	0.724816761	0.989068072	-4.983001883
hsa-mir-199a-2	MI0000281	-0.033445744	0.908589747	0.478238595	0.639579324	0.982649571	-4.952867575
hsa-mir-3678	MI0016079	-0.033503474	0.811693366	0.447819667	0.660872133	0.984005175	-4.961335284
hsa-miR-6502-5p	MIMAT0025460	-0.033564849	0.855275244	0.520619451	0.610455689	0.975777389	-4.940185845
hsa-mir-562	MI0003568	-0.033588131	0.897959199	0.529477705	0.604451364	0.975026084	-4.937406125
hsa-miR-4421	MIMAT0018934	-0.03362975	0.827029082	0.437372521	0.668256831	0.984005175	-4.964120153
hsa-miR-5691	MIMAT0022483	-0.033633069	0.8242084	0.450836559	0.658746336	0.984005175	-4.960519319
hsa-miR-3529-3p	MIMAT0022741	-0.033648548	0.976127863	0.453372586	0.656961727	0.984005175	-4.95982934
hsa-miR-412-3p	MIMAT0002170	-0.033730572	0.921424457	0.478082736	0.639687611	0.982649571	-4.952912319
hsa-miR-3911	MIMAT0018185	-0.033782474	1.198182775	0.215703292	0.832210596	0.996080495	-5.008042805
hsa-miR-513c-3p	MIMAT0022728	-0.033819103	0.937772824	0.468177745	0.646586721	0.982649571	-4.9557272
hsa-miR-509-3-5p	MIMAT0004975	-0.033840827	0.878516551	0.384990755	0.705807863	0.986573137	-4.977125278
hsa-miR-3659	MIMAT0018080	-0.033845749	0.890562949	0.408605419	0.688773587	0.98554565	-4.971460746
hsa-miR-4457	MIMAT0018979	-0.03385739	0.860892021	0.357304598	0.725988099	0.989104742	-4.983349002
hsa-miR-4316	MIMAT0016867	-0.03403751	1.051907732	0.28626918	0.778703544	0.994214304	-4.9972384
hsa-mir-599	MI0003611	-0.03406187	1.04750531	0.282026331	0.781891148	0.994214304	-4.997972675
hsa-mir-3164	MI0014194	-0.034080447	0.932792548	0.587291678	0.566001547	0.96620986	-4.918180673
hsa-miR-3122	MIMAT0014984	-0.034173632	0.93410887	0.373806699	0.713933359	0.9869374	-4.97969379
hsa-mir-200a	MI0000737	-0.034261259	0.932632705	0.530668276	0.603646606	0.974742703	-4.937029141
hsa-miR-6882-5p	MIMAT0027664	-0.034584525	0.946224134	0.301469974	0.767317504	0.994214304	-4.99451922
hsa-miR-494-5p	MIMAT0026607	-0.034591165	0.890975911	0.472941184	0.643264602	0.982649571	-4.954380524
hsa-miR-6826-5p	MIMAT0027552	-0.034624049	1.438470897	0.132242083	0.89660237	0.999057213	-5.016928432
hsa-mir-3667	MI0016068	-0.034652977	1.001450238	0.388291107	0.703417092	0.986573137	-4.97635327
hsa-mir-4433b	MI0025511	-0.034663191	0.956965303	0.37053788	0.716315083	0.987260208	-4.980430594
hsa-miR-511-5p	MIMAT0002808	-0.03480408	0.768399787	0.35344947	0.728815252	0.989702354	-4.984179733
hsa-miR-3925-3p	MIMAT0019228	-0.034813139	0.888150155	0.42363119	0.678024285	0.985064407	-4.967686689
hsa-miR-1277-5p	MIMAT0022724	-0.034888016	0.969140822	0.581732049	0.569642118	0.967582466	-4.920110581
hsa-miR-4729	MIMAT0019851	-0.03493114	0.832694573	0.46214112	0.650807997	0.982649571	-4.957415057
hsa-miR-371b-3p	MIMAT0019893	-0.034999667	1.046207431	0.450093543	0.659269606	0.984005175	-4.960720767
hsa-miR-6749-3p	MIMAT0027399	-0.035093383	0.972511574	0.369306495	0.717213087	0.987607855	-4.980706519

hsa-mir-27a	MI0000085	-0.035136333	0.882458121	0.431825413	0.672192418	0.984005175	-4.965573103
hsa-miR-6744-3p	MIMAT0027390	-0.035257235	0.865167245	0.486239048	0.634032226	0.98055505	-4.950552116
hsa-miR-4732-3p	MIMAT0019856	-0.035289887	0.885691516	0.383430176	0.706939467	0.986573137	-4.977488095
hsa-mir-6810	MI0022655	-0.035298384	0.880541391	0.472506483	0.643567447	0.982649571	-4.95450396
hsa-miR-3918	MIMAT0018192	-0.035342957	0.980240398	0.390960048	0.701486082	0.985875184	-4.975724282
hsa-mir-8052	MI0025888	-0.035504488	0.887202096	0.487407901	0.633223694	0.98055505	-4.950210762
hsa-mir-34b	MI0000742	-0.035561245	0.967206363	0.421685824	0.679411968	0.985064407	-4.96818273
hsa-miR-548av-5p	MIMAT0022303	-0.035680605	0.850057612	0.336856344	0.741029947	0.990306303	-4.987654866
hsa-miR-4671-5p	MIMAT0019752	-0.035800139	0.793997635	0.593693309	0.561824962	0.96620986	-4.915937258
hsa-miR-4287	MIMAT0016917	-0.035812784	0.859160055	0.555295182	0.587120895	0.968332399	-4.929052099
hsa-mir-486-1	MI0002470	-0.035854391	0.842725817	0.415081352	0.684132064	0.98554565	-4.969850322
hsa-mir-622	MI0003636	-0.035923146	0.948412798	0.340654137	0.738227759	0.989764242	-4.986873893
hsa-miR-6802-3p	MIMAT0027505	-0.035961379	1.065252194	0.32688434	0.748405766	0.990697422	-4.989664719
hsa-miR-4659a-5p	MIMAT0019726	-0.035983693	0.815223495	0.363168591	0.721695664	0.988653693	-4.982068541
hsa-miR-7703	MIMAT0030018	-0.035986672	0.853870243	0.370392611	0.716421	0.987260208	-4.980463192
hsa-miR-4738-3p	MIMAT0019867	-0.036036005	1.092166832	0.307753968	0.762626533	0.993576403	-4.993354745
hsa-mir-217	MI0000293	-0.036169961	0.847260833	0.513550011	0.615268425	0.976745744	-4.942372336
hsa-mir-3142	MI0014166	-0.036176742	0.935845559	0.479984347	0.638366992	0.982649571	-4.952365457
hsa-miR-524-5p	MIMAT0002849	-0.036281283	0.813228522	0.552741229	0.588823908	0.968797375	-4.929895209
hsa-mir-5571	MI0019115	-0.036336358	1.026164518	0.497633137	0.626171314	0.978652564	-4.947191246
hsa-mir-449c	MI0003823	-0.036424884	0.840341782	0.600051988	0.557692763	0.96620986	-4.913686456
hsa-mir-1307	MI0006444	-0.036489652	0.955533287	0.570163185	0.577257076	0.967979651	-4.924071398
hsa-miR-4732-5p	MIMAT0019855	-0.036491139	1.690205217	0.140837514	0.889927631	0.998939026	-5.016209288
hsa-miR-1202	MIMAT0005865	-0.036494417	1.559072405	0.254439641	0.802713155	0.994214304	-5.002483263
hsa-miR-513b-5p	MIMAT0005788	-0.036624529	0.8819547	0.347311419	0.733324997	0.989764242	-4.985484254
hsa-miR-1227-3p	MIMAT0005580	-0.036696017	0.940057311	0.346000134	0.734289752	0.989764242	-4.985760049
hsa-mir-4296	MI0015823	-0.036868803	0.869254472	0.42114332	0.679799166	0.985064407	-4.968320667
hsa-mir-193b	MI0003137	-0.036953044	0.897486697	0.315459291	0.756887698	0.99324537	-4.991894713
hsa-miR-640	MIMAT0003310	-0.037077795	0.851347827	0.471809751	0.644052977	0.982649571	-4.954701574
hsa-miR-6504-5p	MIMAT0025464	-0.037083711	0.817309227	0.534268907	0.601216032	0.973453769	-4.93588416
hsa-miR-361-3p	MIMAT0004682	-0.037119801	0.912422555	0.400026899	0.694942084	0.98554565	-4.973556293
hsa-miR-4717-5p	MIMAT0019829	-0.03713544	0.916378624	0.474778593	0.641985256	0.982649571	-4.953857583
hsa-mir-2681	MI0012062	-0.037212074	0.909782907	0.422046921	0.679154297	0.985064407	-4.968090822
hsa-miR-6756-3p	MIMAT0027413	-0.037247585	1.027139704	0.523829044	0.608276772	0.975135504	-4.939183805
hsa-mir-4421	MI0016758	-0.037280962	0.957568714	0.455558373	0.655425317	0.984005175	-4.959231669
hsa-mir-26a-1	MI0000083	-0.037340404	0.815653963	0.453306558	0.657008164	0.984005175	-4.959847351
hsa-mir-3144	MI0014169	-0.037438821	0.874321203	0.654159208	0.523204717	0.960979619	-4.893636395
hsa-mir-6763	MI0022608	-0.037443094	1.060567393	0.292711261	0.773871563	0.994214304	-4.9961029
hsa-miR-4639-3p	MIMAT0019698	-0.037560439	0.876763303	0.476918292	0.640496911	0.982649571	-4.953246164
hsa-mir-563	MI0003569	-0.037623465	1.048144628	0.362022773	0.722533645	0.988653693	-4.982320339
hsa-miR-548y	MIMAT0018354	-0.037650615	1.019920062	0.374617871	0.7133428	0.9869374	-4.979509974
hsa-miR-507	MIMAT0002879	-0.037734666	0.785823706	0.42709298	0.675557882	0.984705521	-4.966798533
hsa-miR-6845-3p	MIMAT0027591	-0.03775796	0.980396711	0.428134212	0.674816792	0.984705521	-4.966530031
hsa-miR-5006-3p	MIMAT0021034	-0.037805561	0.745333465	0.42834675	0.674665563	0.984705521	-4.966475146
hsa-miR-452-5p	MIMAT0001635	-0.03787893	1.072063362	0.225543333	0.824690728	0.995938202	-5.006716441
hsa-miR-1245a	MIMAT0005897	-0.037934272	0.88600578	0.500742177	0.62403443	0.978652564	-4.946261309
hsa-miR-3169	MIMAT0015044	-0.037944551	0.928565681	0.740870261	0.470548664	0.952023826	-4.858217498
hsa-miR-4509	MIMAT0019046	-0.037998552	1.06520993	0.507332569	0.619516331	0.97746608	-4.944271859
hsa-mir-579	MI0003586	-0.038000872	0.903260068	0.468315441	0.646490579	0.982649571	-4.955688455
hsa-miR-191-3p	MIMAT0001618	-0.038086871	1.264507196	0.341699082	0.737457423	0.989764242	-4.98665751
hsa-mir-514b	MI0014251	-0.03810015	0.969703549	0.567449715	0.579050812	0.967979651	-4.92498961
hsa-miR-4711-3p	MIMAT0019817	-0.038103304	0.957192562	0.331847169	0.74473176	0.990306303	-4.98867185
hsa-miR-5585-3p	MIMAT0022286	-0.038132827	0.981539549	0.265735146	0.79416773	0.994214304	-5.000691687
hsa-mir-4481	MI0016842	-0.038170125	0.920948805	0.557234885	0.585829159	0.968332399	-4.928409323
hsa-miR-4738-5p	MIMAT0019866	-0.038183074	0.873167752	0.362752101	0.722000217	0.988653693	-4.982160156
hsa-miR-5004-3p	MIMAT0021028	-0.038203213	0.74441231	0.515375602	0.614023832	0.976745744	-4.941810419
hsa-miR-758-5p	MIMAT0022929	-0.038229992	1.00356138	0.633288135	0.53636348	0.961508042	-4.901559791
hsa-miR-5588-5p	MIMAT0022295	-0.038243983	0.885317878	0.625734255	0.541171094	0.963766957	-4.904369079
hsa-miR-5699-3p	MIMAT0022492	-0.038351126	0.814335123	0.486944196	0.633544395	0.98055505	-4.950346277
hsa-miR-6814-3p	MIMAT0027529	-0.038582862	0.821441622	0.469003996	0.646009915	0.982649571	-4.955494545
hsa-mir-3125	MI0014142	-0.038639208	1.030478285	0.396176802	0.697717859	0.985651988	-4.974482783
hsa-mir-15b	MI0000438	-0.038663288	0.853685284	0.448306274	0.660529049	0.984005175	-4.96120403
hsa-mir-4501	MI0016864	-0.038670326	0.88897214	0.533197101	0.601939035	0.973599219	-4.936225754
hsa-mir-7-3	MI0000265	-0.038699698	1.007009595	0.423029885	0.678453084	0.985064407	-4.967840249
hsa-mir-4781	MI0017426	-0.038739322	0.812109066	0.545932395	0.593376423	0.970443275	-4.932125049
hsa-mir-891b	MI0005534	-0.038843248	1.041232162	0.55374874	0.588151783	0.968623156	-4.929563047

hsa-miR-4319	MIMAT0016870	-0.038846316	0.989819487	0.504916287	0.621171002	0.977635702	-4.945004137
hsa-mir-5705	MI0019313	-0.039134719	0.975218606	0.591954976	0.562957463	0.96620986	-4.916548691
hsa-miR-6505-3p	MIMAT0025467	-0.039159067	0.969712075	0.560575397	0.583607987	0.968332399	-4.92729741
hsa-miR-6779-3p	MIMAT0027459	-0.039164676	1.01830313	0.410274063	0.687576363	0.98554565	-4.971048133
hsa-mir-548f-1	MI0006374	-0.039179511	0.974498231	0.36458151	0.72066285	0.988602576	-4.981756979
hsa-mir-6785	MI0022630	-0.039291992	1.123967029	0.450873171	0.658720557	0.984005175	-4.960509384
hsa-mir-613	MI0003626	-0.039443217	1.070188013	0.386168846	0.704954085	0.986573137	-4.976850437
hsa-mir-4272	MI0015880	-0.039466259	0.940506798	0.645946456	0.528360747	0.960979619	-4.896782463
hsa-mir-4318	MI0015847	-0.039471424	0.986353343	0.504474666	0.621473654	0.977686912	-4.945137615
hsa-miR-1245b-5p	MIMAT0019950	-0.039523885	0.788296596	0.518004984	0.612233415	0.976534918	-4.940997771
hsa-miR-1278	MIMAT0005936	-0.039662158	0.921183149	0.654982326	0.522689535	0.960979619	-4.893319068
hsa-miR-548n	MIMAT0005916	-0.04000816	1.039021932	0.404277301	0.691882951	0.98554565	-4.972523393
hsa-mir-3160-1	MI0014189	-0.040102406	0.842373878	0.461671309	0.651137048	0.982649571	-4.957545537
hsa-mir-4268	MI0015874	-0.040131689	0.905930901	0.394510229	0.698920786	0.98572659	-4.974881134
hsa-miR-380-5p	MIMAT0000734	-0.040242326	0.798630416	0.527384209	0.605867737	0.975047002	-4.93806707
hsa-miR-3160-3p	MIMAT0015034	-0.040284212	0.97101843	0.479617018	0.638621994	0.982649571	-4.952471254
hsa-mir-6503	MI0022215	-0.040286574	1.139112862	0.393101926	0.699937947	0.98572659	-4.975216483
hsa-miR-1291	MIMAT0005881	-0.040298916	0.833062758	0.421698818	0.679402696	0.985064407	-4.968179424
hsa-miR-5705	MIMAT0022499	-0.040323012	0.994108356	0.301756233	0.767103606	0.994214304	-4.994466686
hsa-mir-4258	MI0015857	-0.040331484	0.828966362	0.462907801	0.650271183	0.982649571	-4.957201855
hsa-miR-548aq-5p	MIMAT0022263	-0.040342456	0.962936376	0.524592375	0.60775913	0.975135504	-4.938944633
hsa-mir-3692	MI0016093	-0.04039892	1.014986101	0.40814494	0.689104124	0.98554565	-4.971574324
hsa-miR-199b-5p	MIMAT0000263	-0.040465812	0.951241628	0.442663235	0.664512522	0.984005175	-4.962717725
hsa-miR-196b-5p	MIMAT0001080	-0.040624553	1.02018104	0.297424653	0.770342325	0.994214304	-4.995256364
hsa-miR-3145-5p	MIMAT0019205	-0.040802881	0.927938639	0.726908702	0.478803254	0.955762806	-4.864189131
hsa-mir-6719	MI0022554	-0.040855499	1.002616092	0.400942268	0.694282801	0.98554565	-4.973334741
hsa-miR-3652	MIMAT0018072	-0.041046939	1.484087777	0.221266916	0.827956674	0.996080495	-5.007300069
hsa-mir-887	MI0005562	-0.04123011	0.840373819	0.718882833	0.483587796	0.957574873	-4.867575689
hsa-miR-514b-5p	MIMAT0015087	-0.041241152	0.922375987	0.424643336	0.677302771	0.985064407	-4.967427735
hsa-mir-6070	MI0020347	-0.041258879	1.003926097	0.441640292	0.665235755	0.984005175	-4.962990145
hsa-miR-4511	MIMAT0019048	-0.041272091	1.045433574	0.663945059	0.51709854	0.960979619	-4.889840099
hsa-miR-6750-5p	MIMAT0027400	-0.041398274	2.299289719	0.157448033	0.877053686	0.998806144	-5.014691822
hsa-miR-631	MIMAT0003300	-0.041461722	0.93899747	0.566190538	0.579884171	0.968089289	-4.925414308
hsa-miR-4427	MIMAT0018942	-0.041525084	0.858811599	0.417928961	0.682095242	0.985064407	-4.969134435
hsa-mir-588	MI0003597	-0.041541723	0.943689781	0.941516304	0.361795243	0.930573075	-4.761493278
hsa-miR-506-3p	MIMAT0002878	-0.041802774	0.893877953	0.400193193	0.694822294	0.98554565	-4.97351608
hsa-mir-3944	MI0016601	-0.04180453	0.965180284	0.531978151	0.602761822	0.974069804	-4.936613455
hsa-miR-520c-3p	MIMAT0002846	-0.041863758	0.923282004	0.420114787	0.68053351	0.985064407	-4.968581713
hsa-miR-346	MIMAT0000773	-0.041927759	1.03707547	0.270502535	0.790569154	0.994214304	-4.999912504
hsa-mir-3168	MI0014199	-0.041956286	0.829993951	0.588571029	0.565165549	0.96620986	-4.917734147
hsa-miR-377-5p	MIMAT0004689	-0.041992327	1.051657572	0.390374799	0.701909334	0.986021798	-4.975862565
hsa-mir-3153	MI0014180	-0.042052665	1.846963435	0.242080799	0.812092971	0.994603723	-5.004355451
hsa-mir-3620	MI0016011	-0.042060206	0.941602005	0.497344655	0.626369768	0.978652564	-4.947277254
hsa-mir-208b	MI0005570	-0.042094831	0.894909602	0.694236489	0.49845851	0.960979619	-4.877762808
hsa-miR-4783-5p	MIMAT0019946	-0.042145098	0.963874207	0.463678168	0.64973199	0.982649571	-4.956987286
hsa-mir-6831	MI0022676	-0.042213945	0.932466205	0.514362738	0.614714199	0.976745744	-4.942122412
hsa-mir-7150	MI0023610	-0.042323495	0.967582628	0.541388118	0.596424702	0.972347475	-4.933598766
hsa-miR-6506-5p	MIMAT0025468	-0.042410361	0.881320601	0.550609166	0.590247527	0.969688229	-4.930596242
hsa-mir-939	MI0005761	-0.042423649	1.068863657	0.319640031	0.753780099	0.992667719	-4.991087716
hsa-mir-934	MI0005756	-0.042573899	0.948310768	0.465785647	0.648257968	0.982649571	-4.956398547
hsa-mir-3182	MI0014224	-0.042669006	0.903895451	0.455518269	0.655453492	0.984005175	-4.95924266
hsa-miR-4524a-5p	MIMAT0019062	-0.042816623	0.968206833	0.427947079	0.674949957	0.984705521	-4.966578334
hsa-miR-6832-3p	MIMAT0027565	-0.042901754	0.823845381	0.464196872	0.649369055	0.982649571	-4.95684262
hsa-mir-4329	MI0015901	-0.043093301	1.424039819	0.276629538	0.78595155	0.994214304	-4.998891054
hsa-miR-5047	MIMAT0020541	-0.043146302	0.957845115	0.332635172	0.744148986	0.990306303	-4.988512855
hsa-mir-3688-2	MI0017447	-0.043177345	0.948935364	0.520755811	0.610363039	0.975777389	-4.940143391
hsa-miR-4999-3p	MIMAT0021018	-0.043279568	0.805290142	0.826136856	0.422053606	0.938060329	-4.819564659
hsa-miR-138-2-3p	MIMAT0004596	-0.043347057	0.834627755	0.486450093	0.633886204	0.98055505	-4.95049054
hsa-mir-449b	MI0003673	-0.043400029	1.008090515	0.489747119	0.631607038	0.98055505	-4.949525262
hsa-mir-4751	MI0017390	-0.043494703	1.14191004	0.368770557	0.717604063	0.987694154	-4.980826331
hsa-miR-4535	MIMAT0019075	-0.043678609	1.100455871	0.425329824	0.67681359	0.985064407	-4.96725176
hsa-mir-548e	MI0006344	-0.043822828	0.97847993	0.435833855	0.669347486	0.984005175	-4.964524965
hsa-mir-1915	MI0008336	-0.043920363	1.010251217	0.429259624	0.67401618	0.984590139	-4.966239112
hsa-miR-4272	MIMAT0016902	-0.044184031	0.805436492	0.529223945	0.60462296	0.975026084	-4.937486373
hsa-mir-6733	MI0022578	-0.044360351	0.912773612	0.761090546	0.458748901	0.951465791	-4.849388806
hsa-mir-641	MI0003656	-0.044391649	1.061581392	0.529732323	0.604279212	0.975026084	-4.93732557

hsa-miR-492	MIMAT0002812	-0.044525432	0.911027223	0.599596654	0.557988118	0.96620986	-4.913848374
hsa-miR-6801-3p	MIMAT0027503	-0.044540519	0.991129092	0.460931458	0.651655384	0.982649571	-4.957750757
hsa-mir-3923	MI0016430	-0.044559493	1.079962427	0.49894523	0.625269071	0.978652564	-4.946799462
hsa-miR-5688	MIMAT0022479	-0.044607077	1.007946086	0.405587848	0.690940831	0.98554565	-4.972202781
hsa-miR-29c-5p	MIMAT0004673	-0.044608932	1.138102899	0.330982277	0.745371585	0.990306303	-4.988845935
hsa-mir-2355	MI0015873	-0.0446091	0.895330295	0.699841856	0.495052959	0.960979619	-4.87547421
hsa-mir-4793	MI0017440	-0.044662195	1.0276626	0.550543158	0.59029163	0.969688229	-4.930617905
hsa-mir-589	MI0003599	-0.044740141	1.03838985	0.690623147	0.500661087	0.960979619	-4.879229227
hsa-mir-548a-3	MI0003612	-0.044866143	0.932326499	0.481244924	0.637492253	0.982647382	-4.952001799
hsa-mir-4715	MI0017349	-0.04491419	0.91793731	0.640022186	0.532097752	0.960979619	-4.899029167
hsa-miR-3680-5p	MIMAT0018106	-0.045091342	0.814715983	0.541587872	0.596290542	0.972234933	-4.93353423
hsa-mir-4711	MI0017345	-0.045107355	0.944215595	0.413210949	0.685471302	0.98554565	-4.970317965
hsa-mir-3135b	MI0016809	-0.045213361	0.881215281	0.632179459	0.537067599	0.96159396	-4.901974057
hsa-miR-7151-3p	MIMAT0028213	-0.045265322	0.860825283	0.509816602	0.617817483	0.97718859	-4.943515585
hsa-miR-325	MIMAT0000771	-0.04562408	0.895728665	0.594272837	0.561447677	0.96620986	-4.915733046
hsa-miR-105-5p	MIMAT0000102	-0.045647271	0.860147443	0.783515997	0.445878907	0.949212502	-4.839350234
hsa-mir-4445	MI0016788	-0.045719747	0.945139451	0.736929666	0.472869656	0.952660318	-4.859913293
hsa-miR-3200-3p	MIMAT0015085	-0.045806898	0.770962227	0.659723825	0.519727494	0.960979619	-4.891484012
hsa-mir-569	MI0003576	-0.045837067	0.860935324	0.599165715	0.558267727	0.96620986	-4.914001512
hsa-mir-4261	MI0015868	-0.045838762	0.997205959	0.419886604	0.680696472	0.985064407	-4.968639543
hsa-mir-577	MI0003584	-0.045910449	0.931672072	0.784648152	0.44523524	0.949212502	-4.838836591
hsa-mir-4311	MI0015841	-0.045979432	0.878981333	0.527089999	0.60606692	0.975047002	-4.938159758
hsa-mir-1-2	MI0000437	-0.045992695	0.955640932	0.670167748	0.513237019	0.960979619	-4.88739924
hsa-mir-4527	MI0016894	-0.046063716	0.906412047	0.693139531	0.499126578	0.960979619	-4.878208726
hsa-miR-3939	MIMAT0018355	-0.046166204	0.926512194	0.580303703	0.570579424	0.967644566	-4.920603631
hsa-miR-20a-5p	MIMAT0000075	-0.04621688	9.74643088	0.245862065	0.809219916	0.994603723	-5.003792421
hsa-mir-3064	MI0017375	-0.046245244	0.925365208	0.420598442	0.680188152	0.985064407	-4.968459036
hsa-mir-6783	MI0022628	-0.046362708	0.951554654	0.446779529	0.661605749	0.984005175	-4.961615384
hsa-miR-3926	MIMAT0018201	-0.046440697	0.865584097	0.634234112	0.535763098	0.961410641	-4.901205788
hsa-miR-1908-5p	MIMAT0007881	-0.046525131	8.249264024	0.147260854	0.884945217	0.998806144	-5.015642434
hsa-miR-1468-5p	MIMAT0006789	-0.046525916	0.794961579	0.616201365	0.547272052	0.965790957	-4.907869823
hsa-miR-4693-5p	MIMAT0019784	-0.046573376	0.845706226	0.679361308	0.507562417	0.960979619	-4.883754985
hsa-mir-676	MI0016436	-0.046604494	0.873920741	0.905758614	0.37980354	0.930573075	-4.780179754
hsa-miR-378a-5p	MIMAT0000731	-0.046604603	0.879092914	0.471149605	0.644513166	0.982649571	-4.954888554
hsa-miR-6509-5p	MIMAT0025474	-0.04685998	1.187590681	0.400574296	0.694547798	0.98554565	-4.973423862
hsa-miR-30c-2-3p	MIMAT0004550	-0.046865871	0.944359499	0.432648361	0.671607917	0.984005175	-4.965358678
hsa-mir-323a	MI0000807	-0.047109109	0.91841077	0.759092173	0.459906854	0.951465791	-4.850270789
hsa-mir-3925	MI0016433	-0.04720271	0.888908292	0.612556346	0.549614741	0.965790957	-4.909195209
hsa-miR-520f-5p	MIMAT0026609	-0.047283547	0.924557866	0.589965962	0.564254773	0.96620986	-4.917246246
hsa-miR-6855-3p	MIMAT0027611	-0.047303863	1.056571081	0.53546947	0.60040669	0.97307982	-4.935500761
hsa-mir-320b-2	MI0003839	-0.047341271	0.863523537	0.893023289	0.386362234	0.930573075	-4.786687338
hsa-mir-6068	MI0020345	-0.047467266	0.903946396	0.447776255	0.660902744	0.984005175	-4.961346987
hsa-miR-6766-3p	MIMAT0027433	-0.047474208	0.980764026	0.549984668	0.590664848	0.970023457	-4.930801097
hsa-miR-4283	MIMAT0016914	-0.047575402	0.926755433	0.42187654	0.679275872	0.985064407	-4.968134197
hsa-miR-4263	MIMAT0016898	-0.047734504	0.868702715	0.548652866	0.591555327	0.970056246	-4.931237239
hsa-mir-574	MI0003581	-0.047995483	1.029291727	0.500060377	0.624502741	0.978652564	-4.946465713
hsa-mir-196a-1	MI0000238	-0.048086201	0.941477113	0.61951455	0.545147384	0.965790957	-4.906658773
hsa-mir-7852	MI0025522	-0.048150336	0.938847169	0.505562016	0.620728599	0.97746608	-4.944808768
hsa-mir-708	MI0005543	-0.048346738	1.081969122	0.573441254	0.575093979	0.967644566	-4.922956655
hsa-miR-4723-5p	MIMAT0019838	-0.048351692	0.886427716	0.55870071	0.584853969	0.968332399	-4.927922182
hsa-miR-4491	MIMAT0019026	-0.048391559	1.035787239	0.804294662	0.434159072	0.944343675	-4.829819199
hsa-miR-4703-5p	MIMAT0019801	-0.048590341	0.994845513	0.681223306	0.506417572	0.960979619	-4.883011387
hsa-miR-6829-3p	MIMAT0027559	-0.048604225	1.041935164	0.543249655	0.595175038	0.97134249	-4.932996471
hsa-miR-562	MIMAT0003226	-0.048689155	0.990669107	0.609576387	0.551534044	0.96620986	-4.910273345
hsa-mir-92a-2	MI0000094	-0.048694956	0.976171837	0.801255113	0.435861099	0.944812484	-4.831227124
hsa-miR-376a-2-5p	MIMAT0022928	-0.048775111	0.815799797	0.729269729	0.477401208	0.954875771	-4.863186443
hsa-miR-1238-3p	MIMAT0005593	-0.048836904	1.205693069	0.346881655	0.733641137	0.989764242	-4.985574756
hsa-mir-4789	MI0017436	-0.04902485	0.879149975	0.616946882	0.546793575	0.965790957	-4.907597843
hsa-miR-34c-3p	MIMAT0004677	-0.049040444	1.013398685	0.721098912	0.482263842	0.957574873	-4.866643988
hsa-mir-4471	MI0016822	-0.049076388	0.956033521	0.548070222	0.591945112	0.970056246	-4.931427731
hsa-mir-6788	MI0022633	-0.049283052	0.941726118	0.609085556	0.551850523	0.96620986	-4.910450457
hsa-miR-1252-3p	MIMAT0026744	-0.04930754	0.876452744	0.620467914	0.544536854	0.965636501	-4.906309181
hsa-miR-7106-5p	MIMAT0028109	-0.049440395	1.738858872	0.170383363	0.867052843	0.997250847	-5.013393637
hsa-miR-5685	MIMAT0022475	-0.049547302	0.883628795	0.456010355	0.655107815	0.984005175	-4.959107737
hsa-mir-920	MI0005712	-0.049585277	0.996407974	0.480015691	0.638345235	0.982649571	-4.952356426
hsa-mir-6871	MI0022718	-0.049598179	0.93850053	0.578323692	0.571880083	0.967644566	-4.921285234

hsa-miR-527	MIMAT0002862	-0.049651148	0.82676045	0.779599067	0.448110329	0.950247229	-4.841122233
hsa-miR-518a-5p	MIMAT0005457	-0.049651148	0.82676045	0.779599067	0.448110329	0.950247229	-4.841122233
hsa-mir-8084	MI0025920	-0.049736472	0.944254123	0.661192674	0.518811841	0.960979619	-4.890913075
hsa-miR-4761-3p	MIMAT0019909	-0.049898574	0.839428399	0.646568111	0.527969468	0.960979619	-4.896545603
hsa-miR-4496	MIMAT0019031	-0.04993837	1.133132402	0.366305787	0.719403204	0.98842922	-4.981375164
hsa-miR-1298-5p	MIMAT0005800	-0.050125869	0.824159144	0.612544138	0.549622597	0.965790957	-4.909199635
hsa-miR-3192-3p	MIMAT0027027	-0.050187995	0.780911044	0.958014775	0.353688725	0.930573075	-4.752667689
hsa-miR-4479	MIMAT0019011	-0.050347488	0.9189084	0.459244387	0.652838034	0.983005937	-4.958217536
hsa-miR-6789-3p	MIMAT0027479	-0.050445981	0.691136764	0.618888716	0.54554837	0.965790957	-4.906887992
hsa-miR-4422	MIMAT0018935	-0.050467234	1.15270848	0.449323955	0.659811784	0.984005175	-4.960929084
hsa-miR-599	MIMAT0003267	-0.050527425	0.790533785	0.451923726	0.657981029	0.984005175	-4.960223987
hsa-mir-1234	MI0006324	-0.050568798	0.905900673	0.620164677	0.544731005	0.965736099	-4.90642043
hsa-miR-3912-3p	MIMAT0018186	-0.050581173	0.900364485	0.489838232	0.631544109	0.98055505	-4.949498499
hsa-miR-4723-3p	MIMAT0019839	-0.050582871	0.981936031	0.55041859	0.590374864	0.969734917	-4.93065878
hsa-miR-6876-5p	MIMAT0027652	-0.050623661	0.878415903	0.508570089	0.618669701	0.977382438	-4.94389553
hsa-mir-4787	MI0017434	-0.050664036	1.168327926	0.466752555	0.647582198	0.982649571	-4.956127579
hsa-miR-939-3p	MIMAT0022939	-0.050669721	0.841821087	0.711012024	0.488307653	0.959021141	-4.870863873
hsa-miR-6850-3p	MIMAT0027601	-0.050703481	0.801219884	0.593690681	0.561826672	0.96620986	-4.915938184
hsa-miR-6774-5p	MIMAT0027448	-0.050762461	1.207923747	0.308314567	0.762208513	0.99324537	-4.993249716
hsa-miR-1343-3p	MIMAT0019776	-0.050766312	0.98181073	0.584679578	0.56771047	0.966465648	-4.919089545
hsa-miR-586	MIMAT0003252	-0.050806211	0.857638912	0.654266187	0.523137743	0.960979619	-4.893595173
hsa-miR-3689a-3p	MIMAT0018118	-0.050853164	0.930452436	0.400181121	0.69483099	0.98554565	-4.973519
hsa-miR-936	MIMAT0004979	-0.050932961	1.136258066	0.427390863	0.67534583	0.984705521	-4.966721783
hsa-miR-6844	MIMAT0027589	-0.051014576	0.84345914	0.710904844	0.488372115	0.959021141	-4.870908424
hsa-mir-597	MI0003609	-0.051053087	0.828980517	0.979221436	0.343456515	0.930208704	-4.741137256
hsa-miR-6769a-3p	MIMAT0027439	-0.051224198	0.994406994	0.430949568	0.672814729	0.984005175	-4.965800879
hsa-mir-617	MI0003631	-0.051265861	0.972743317	0.748563484	0.466037484	0.951465791	-4.854883469
hsa-miR-3190-3p	MIMAT0022839	-0.051376196	0.99151983	0.884277974	0.390910098	0.930573075	-4.791110669
hsa-miR-4657	MIMAT0019724	-0.051434955	0.980656376	0.644370243	0.529353571	0.960979619	-4.897382082
hsa-mir-6813	MI0022658	-0.051550149	0.984238511	0.599833647	0.55783438	0.96620986	-4.913764113
hsa-let-7a-2	MI0000061	-0.051622829	0.962185123	0.51093538	0.617053079	0.976821573	-4.943173823
hsa-miR-3919	MIMAT0018193	-0.051710122	0.996682154	0.475236476	0.641666625	0.982649571	-4.953726964
hsa-miR-8064	MIMAT0030991	-0.051714484	1.467175071	0.228569119	0.822381924	0.995221754	-5.006296813
hsa-mir-605	MI0003618	-0.051724619	1.016549867	0.568715341	0.578213811	0.967979651	-4.924561846
hsa-miR-1288-3p	MIMAT0005942	-0.051733405	0.80372283	0.662607578	0.517930689	0.960979619	-4.890362006
hsa-mir-3976	MI0016994	-0.051755556	0.943961868	0.838201033	0.41546199	0.93437823	-4.813798103
hsa-mir-146b	MI0003129	-0.051776703	1.01062452	0.319396731	0.753960827	0.992667719	-4.991134965
hsa-mir-7856	MI0025526	-0.051789644	0.913345037	0.701463617	0.494070224	0.960979619	-4.874808953
hsa-mir-4642	MI0017269	-0.051799916	0.917855332	0.762661507	0.457839881	0.951465791	-4.848694013
hsa-miR-136-3p	MIMAT0004606	-0.051813782	0.980829857	0.528053793	0.605414546	0.975047002	-4.937855943
hsa-miR-6764-5p	MIMAT0027428	-0.051946854	0.890642725	0.678985089	0.507793916	0.960979619	-4.883905005
hsa-mir-1293	MI0006355	-0.051948996	0.812257798	0.851389032	0.408333689	0.931990513	-4.807411473
hsa-mir-4777	MI0017421	-0.051970604	0.904135555	0.753853608	0.462950902	0.951465791	-4.85257302
hsa-miR-129-1-3p	MIMAT0004548	-0.051974382	1.127041327	0.487488113	0.633168226	0.98055505	-4.950187308
hsa-mir-4441	MI0016784	-0.052086361	0.906987538	0.769823323	0.453709955	0.951465791	-4.845510402
hsa-mir-103a-1	MI0000109	-0.052175895	0.891277238	0.69245728	0.499542347	0.960979619	-4.878485742
hsa-miR-548ao-5p	MIMAT0021029	-0.052213519	0.989648026	0.385405626	0.705507154	0.986573137	-4.977028585
hsa-miR-4540	MIMAT0019083	-0.052237264	0.893465434	0.483682344	0.635802462	0.981916263	-4.951296056
hsa-mir-7160	MI0023621	-0.052301444	0.818207145	0.813893349	0.428812121	0.94301493	-4.825342382
hsa-miR-329-3p	MIMAT0001629	-0.052324976	1.151773006	0.342620691	0.736778251	0.989764242	-4.986466129
hsa-miR-106b-3p	MIMAT0004672	-0.052335687	5.435136708	0.121895248	0.904647853	0.999227185	-5.017734256
hsa-miR-6810-3p	MIMAT0027521	-0.052366037	1.086244567	0.724226846	0.480398829	0.956832921	-4.865324521
hsa-miR-3161	MIMAT0015035	-0.052419296	1.008642184	0.319687004	0.753745209	0.992667719	-4.99107859
hsa-mir-6868	MI0022715	-0.052452941	0.952430573	0.604467469	0.554833002	0.96620986	-4.912110363
hsa-miR-6845-5p	MIMAT0027590	-0.052544289	1.632090599	0.187368802	0.853956621	0.996937676	-5.011534341
hsa-mir-1227	MI0006316	-0.052623266	1.154267904	0.546846267	0.592764356	0.970056246	-4.931827276
hsa-mir-4691	MI0017324	-0.052726135	0.89755592	0.694153613	0.498508965	0.960979619	-4.87779652
hsa-mir-190a	MI0000486	-0.052759999	0.888742539	0.696573079	0.497037239	0.960979619	-4.876810839
hsa-miR-3622a-5p	MIMAT0018003	-0.052857338	1.139915594	0.451826985	0.658049114	0.984005175	-4.960250295
hsa-mir-4764	MI0017405	-0.053240771	0.966116746	0.74853238	0.466055669	0.951465791	-4.854897011
hsa-miR-651-5p	MIMAT0003321	-0.053398514	0.811351871	0.829767459	0.42006282	0.936613328	-4.817836917
hsa-miR-4790-5p	MIMAT0019961	-0.053411929	0.834595441	0.604092097	0.55507581	0.96620986	-4.912244769
hsa-miR-3928-3p	MIMAT0018205	-0.053657386	0.96722078	0.619840865	0.544938371	0.965736099	-4.906539171
hsa-miR-4756-3p	MIMAT0019900	-0.053843099	0.843252973	0.635632087	0.534876532	0.960979619	-4.900681747
hsa-miR-765	MIMAT0003945	-0.053878717	1.090870703	0.702271271	0.493581243	0.960979619	-4.874477128
hsa-mir-4712	MI0017346	-0.053946231	0.888878723	0.833719298	0.417902834	0.935967201	-4.815948812

hsa-miR-585-5p	MIMAT0026618	-0.053964769	0.901699557	0.567639636	0.57892517	0.967979651	-4.924925476
hsa-miR-4694-3p	MIMAT0019787	-0.05429248	0.887502567	0.501869082	0.623260754	0.978652564	-4.945922883
hsa-miR-23b-5p	MIMAT0004587	-0.054463569	1.195257455	0.378098758	0.710810761	0.9869374	-4.978716787
hsa-miR-2681-3p	MIMAT0013516	-0.054507604	0.937084408	0.650109953	0.525743298	0.960979619	-4.895192113
hsa-mir-3924	MI0016432	-0.054765664	1.093972578	0.608975936	0.551921217	0.96620986	-4.910489995
hsa-miR-6843-3p	MIMAT0027588	-0.054797426	0.829083901	0.646708047	0.527881413	0.960979619	-4.896492257
hsa-mir-6845	MI0022691	-0.05485205	1.13137469	0.567757373	0.57884729	0.967979651	-4.924885708
hsa-mir-4328	MI0015904	-0.054884452	0.950721644	0.815786002	0.427762833	0.94301493	-4.824454163
hsa-mir-384	MI0001145	-0.05488696	0.928591501	0.621820193	0.543671505	0.964811939	-4.905812454
hsa-mir-632	MI0003647	-0.054999374	1.035062261	0.653044768	0.523902693	0.960979619	-4.894065446
hsa-miR-7161-3p	MIMAT0028233	-0.055113861	0.939787001	0.637933003	0.533419123	0.960979619	-4.899816913
hsa-mir-5590	MI0019150	-0.055366127	0.920357325	0.84166224	0.413583329	0.93368138	-4.812130282
hsa-mir-340	MI0000802	-0.055418663	0.875680889	0.88578863	0.390121945	0.930573075	-4.790349239
hsa-miR-362-3p	MIMAT0004683	-0.055596904	0.973436162	0.790278229	0.442043063	0.947408294	-4.836272577
hsa-miR-4752	MIMAT0019889	-0.055640386	0.850184135	0.879953735	0.393172084	0.930573075	-4.79328412
hsa-mir-3973	MI0016991	-0.055686091	0.956816515	0.706639458	0.490941562	0.959629992	-4.872676461
hsa-mir-4297	MI0015824	-0.05575146	0.882177314	0.550008964	0.59064861	0.970023457	-4.930793131
hsa-miR-521	MIMAT0002854	-0.055763003	0.901825918	0.539152114	0.597927492	0.972358931	-4.93431964
hsa-miR-519d-3p	MIMAT0002853	-0.055840329	0.945657238	0.63553555	0.534937727	0.960979619	-4.900717968
hsa-mir-6743	MI0022588	-0.055899857	1.019350532	0.577999373	0.572093275	0.967644566	-4.92139667
hsa-miR-4663	MIMAT0019735	-0.056017619	0.920739085	0.678988909	0.507791565	0.960979619	-4.883903482
hsa-miR-3925-5p	MIMAT0018200	-0.056045663	1.089120133	0.805122905	0.433696025	0.943798359	-4.829434743
hsa-mir-4522	MI0016889	-0.056073397	0.859683091	0.836220051	0.416539723	0.934671969	-4.814749977
hsa-mir-5586	MI0019143	-0.056172835	0.937104382	0.659262979	0.520014966	0.960979619	-4.891662901
hsa-miR-1285-5p	MIMAT0022719	-0.056230788	0.787586087	0.865716415	0.400681356	0.930573075	-4.800375595
hsa-mir-548i-4	MI0006424	-0.056246458	0.939823725	0.726502576	0.479044673	0.955959324	-4.864361311
hsa-miR-129-5p	MIMAT0000242	-0.056252634	1.062448628	0.385230037	0.705634419	0.986573137	-4.977069521
hsa-mir-4776-1	MI0017419	-0.056259957	0.888252603	0.787944452	0.443364522	0.947408294	-4.837337374
hsa-miR-548f-3p	MIMAT0005895	-0.056531422	0.986868187	0.571955062	0.576074146	0.967644566	-4.923462793
hsa-miR-223-5p	MIMAT0004570	-0.056601684	1.036094075	0.685361715	0.503878467	0.960979619	-4.881352053
hsa-miR-5582-3p	MIMAT0022280	-0.056638861	1.00508749	0.510043771	0.617662233	0.97718859	-4.943446247
hsa-miR-5011-3p	MIMAT0021046	-0.056641748	0.778447554	0.971730561	0.347046758	0.930573075	-4.745233969
hsa-miR-4501	MIMAT0019037	-0.056644127	0.85888707	0.600110958	0.557654517	0.96620986	-4.913665478
hsa-mir-6759	MI0022604	-0.056818586	0.880515232	0.723167434	0.48103001	0.957398519	-4.865771992
hsa-miR-107	MIMAT0000104	-0.05697116	10.69486824	0.460606145	0.651883355	0.982649571	-4.957840892
hsa-mir-671	MI0003760	-0.056973498	0.928261735	0.632626702	0.536783493	0.96159396	-4.901807021
hsa-miR-633	MIMAT0003303	-0.056973963	0.968754373	0.508411467	0.618778188	0.977382438	-4.943943815
hsa-mir-4464	MI0016812	-0.057078277	0.954910233	0.674971395	0.510267481	0.960979619	-4.885500768
hsa-miR-4456	MIMAT0018978	-0.057141762	0.728752717	0.711471977	0.488031081	0.959021141	-4.870672618
hsa-mir-3187	MI0014231	-0.057286493	0.851464168	0.919669156	0.37272656	0.930573075	-4.772982629
hsa-mir-7107	MI0022958	-0.057314409	1.010381812	0.933481838	0.365789263	0.930573075	-4.765744857
hsa-miR-1305	MIMAT0005893	-0.057344295	0.977242161	0.67691213	0.509070573	0.960979619	-4.884730249
hsa-mir-6826	MI0022671	-0.057503969	1.03750637	0.45835737	0.653460228	0.983228103	-4.958462297
hsa-mir-4255	MI0015863	-0.057758905	0.908209131	1.136408351	0.274138405	0.927057986	-4.649394899
hsa-miR-3605-5p	MIMAT0017981	-0.057763958	1.182381535	0.42358232	0.678059131	0.985064407	-4.967699177
hsa-miR-3173-5p	MIMAT0019214	-0.057811841	0.92014963	0.632312282	0.536983216	0.96159396	-4.901924462
hsa-mir-4275	MI0015883	-0.057829078	0.854287615	0.706779057	0.490857341	0.959629992	-4.872618749
hsa-mir-4457	MI0016803	-0.057987285	0.865013038	0.638271907	0.533204648	0.960979619	-4.899689287
hsa-miR-4791	MIMAT0019963	-0.058037244	0.81671616	0.694215446	0.498471321	0.960979619	-4.877771368
hsa-mir-3911	MI0016415	-0.058089671	0.949813819	0.801218435	0.435881663	0.944812484	-4.831244085
hsa-mir-6773	MI0022618	-0.058096936	0.955931858	0.893817469	0.385951008	0.930573075	-4.78628381
hsa-miR-548ac	MIMAT0018938	-0.058221489	1.005293546	1.022060265	0.323429406	0.930208704	-4.717216059
hsa-miR-4742-5p	MIMAT0019872	-0.058254581	0.974404936	0.731383083	0.476148348	0.954010043	-4.862286459
hsa-mir-141	MI0000457	-0.058304896	0.885489362	0.721246897	0.482175508	0.957574873	-4.866581679
hsa-mir-148b	MI0000811	-0.058431609	0.939021019	0.570245332	0.577202818	0.967979651	-4.924043536
hsa-mir-103a-2	MI0000108	-0.058504597	0.765845865	0.906875398	0.379232024	0.930573075	-4.779605368
hsa-miR-8081	MIMAT0031008	-0.058523699	0.802939667	0.858537971	0.404503434	0.930588488	-4.803913422
hsa-miR-4705	MIMAT0019805	-0.058557879	0.88460481	0.861098404	0.403137402	0.930573075	-4.802654446
hsa-miR-7112-3p	MIMAT0028122	-0.058603414	0.87325468	0.739639796	0.471272655	0.952023826	-4.858747886
hsa-mir-1245a	MI0006380	-0.058786617	0.856912271	0.656073005	0.522007333	0.960979619	-4.892898029
hsa-miR-6742-3p	MIMAT0027386	-0.058891929	0.868803744	0.874891033	0.395831475	0.930573075	-4.795817158
hsa-miR-200c-3p	MIMAT0000617	-0.058990686	5.832217918	0.144468825	0.887110315	0.998806144	-5.015891921
hsa-mir-449a	MI0001648	-0.059004273	0.795404602	0.863620995	0.401794552	0.930573075	-4.801410921
hsa-mir-125b-2	MI0000470	-0.059026001	1.11111891	0.365494628	0.719995679	0.98850182	-4.981555002
hsa-mir-6879	MI0022726	-0.059148705	0.976588744	0.940793354	0.362153388	0.930573075	-4.761877086
hsa-miR-6808-3p	MIMAT0027517	-0.059362166	0.874849247	0.95084696	0.357194924	0.930573075	-4.756517662

hsa-mir-8061	MI0025897	-0.05940841	0.968427393	1.113099009	0.283699639	0.927057986	-4.663674908
hsa-miR-331-5p	MIMAT0004700	-0.059440118	0.97339342	0.579924506	0.570828396	0.967644566	-4.920734335
hsa-miR-4722-3p	MIMAT0019837	-0.059789111	1.103415641	0.624392283	0.542027667	0.964055569	-4.904864898
hsa-miR-4642	MIMAT0019702	-0.059796046	0.905541181	0.642965313	0.530239391	0.960979619	-4.897915406
hsa-miR-5587-3p	MIMAT0022290	-0.059912883	0.950983711	0.648718649	0.526617148	0.960979619	-4.895724601
hsa-miR-4526	MIMAT0019065	-0.059991236	1.150130793	0.361924028	0.722605879	0.988667351	-4.982342002
hsa-mir-7844	MI0025514	-0.060030905	0.927371414	1.041499176	0.314624271	0.930208704	-4.706088558
hsa-mir-4681	MI0017313	-0.060039017	0.879481861	0.923028248	0.371031241	0.930573075	-4.771230826
hsa-miR-3664-3p	MIMAT0019220	-0.060309628	0.946355607	0.75947425	0.45968532	0.951465791	-4.850102319
hsa-miR-888-5p	MIMAT0004916	-0.0603356	0.926289383	0.656512839	0.521732368	0.960979619	-4.892728056
hsa-mir-302c	MI0000773	-0.060622952	0.896559678	0.756415078	0.461460922	0.951465791	-4.851449087
hsa-miR-548p	MIMAT0005934	-0.060634623	1.017970021	0.657460963	0.521139921	0.960979619	-4.8923613
hsa-mir-4300	MI0015831	-0.060713885	0.9236297	0.851995486	0.408007839	0.9318533	-4.807115708
hsa-miR-526a-5p	MIMAT0002845	-0.060714014	0.811268488	0.774224222	0.451183698	0.950247229	-4.843540986
hsa-miR-520c-5p	MIMAT0005455	-0.060714014	0.811268488	0.774224222	0.451183698	0.950247229	-4.843540986
hsa-miR-518d-5p	MIMAT0005456	-0.060714014	0.811268488	0.774224222	0.451183698	0.950247229	-4.843540986
hsa-miR-4786-5p	MIMAT0019954	-0.060829114	0.906318337	0.883849164	0.391134015	0.930573075	-4.791326604
hsa-mir-379	MI0000787	-0.061169444	0.842530919	0.87723158	0.394600517	0.930573075	-4.794647659
hsa-miR-466	MIMAT0015002	-0.061250464	0.993312559	0.720806602	0.482438352	0.957574873	-4.866767031
hsa-mir-4766	MI0017407	-0.061514175	0.985930447	0.646030899	0.528307588	0.960979619	-4.896750301
hsa-miR-6807-3p	MIMAT0027515	-0.061621279	0.819590513	0.639830146	0.532219138	0.960979619	-4.899101676
hsa-miR-6885-5p	MIMAT0027670	-0.061653804	1.134326534	0.412621635	0.685893487	0.98554565	-4.970464884
hsa-mir-548am	MI0016904	-0.061743048	0.878657434	0.889731025	0.388070119	0.930573075	-4.788356899
hsa-miR-3150b-5p	MIMAT0019226	-0.061779154	0.959719165	0.69182134	0.499930076	0.960979619	-4.87874373
hsa-mir-6074	MI0020351	-0.061831388	0.897983429	0.766109822	0.455848472	0.951465791	-4.84716445
hsa-miR-8070	MIMAT0030997	-0.061849796	0.930086719	0.894095675	0.385807023	0.930573075	-4.78614238
hsa-miR-449a	MIMAT0001541	-0.061850736	0.894987814	0.712849544	0.487203302	0.958507503	-4.870099138
hsa-miR-431-3p	MIMAT0004757	-0.061868668	0.899942116	0.640307658	0.531917339	0.960979619	-4.898921343
hsa-miR-181a-2-3p	MIMAT0004558	-0.0619222	1.391822493	0.349584468	0.731653747	0.989764242	-4.985003764
hsa-miR-6824-3p	MIMAT0027549	-0.061962773	0.867154689	0.965993098	0.349814424	0.930573075	-4.748354203
hsa-miR-526b-5p	MIMAT0002835	-0.062166582	0.800094382	0.678309506	0.508209778	0.960979619	-4.884174207
hsa-mir-4324	MI0015854	-0.062212312	0.94681223	1.234091512	0.236711422	0.919664114	-4.587140477
hsa-miR-326	MIMAT0000756	-0.062273748	0.932431234	0.652770806	0.524074357	0.960979619	-4.894170816
hsa-miR-1224-3p	MIMAT0005459	-0.062307244	1.295025757	0.599424657	0.558099707	0.96620986	-4.913909507
hsa-miR-671-3p	MIMAT0004819	-0.062334627	1.204950049	0.443113272	0.66419445	0.984005175	-4.962597683
hsa-miR-4733-3p	MIMAT0019858	-0.062444989	0.898760813	0.864017189	0.401583916	0.930573075	-4.801215331
hsa-miR-545-5p	MIMAT0004785	-0.062492629	0.908379921	0.738807226	0.471762914	0.952023826	-4.859106315
hsa-miR-412-5p	MIMAT0026557	-0.062669553	1.130840244	0.463772663	0.649665865	0.982649571	-4.956960943
hsa-miR-626	MIMAT0003295	-0.062893067	0.899054709	0.912525025	0.376349788	0.930573075	-4.776690473
hsa-miR-3689a-5p	MIMAT0018117	-0.062910409	0.88580076	0.840773843	0.414064999	0.93368138	-4.812558934
hsa-miR-3689b-5p	MIMAT0018180	-0.062910409	0.88580076	0.840773843	0.414064999	0.93368138	-4.812558934
hsa-miR-3689e	MIMAT0019009	-0.062910409	0.88580076	0.840773843	0.414064999	0.93368138	-4.812558934
hsa-miR-25-5p	MIMAT0004498	-0.062911168	1.213527687	0.321339656	0.752518002	0.992441805	-4.990756665
hsa-mir-497	MI0003138	-0.062925296	0.949750385	0.725241926	0.479794529	0.956472927	-4.864895221
hsa-mir-4425	MI0016764	-0.062978026	0.861358262	0.843867689	0.412389169	0.93366101	-4.811064455
hsa-miR-876-5p	MIMAT0004924	-0.063063891	0.814783059	0.809062776	0.431497684	0.94301493	-4.827601174
hsa-mir-376a-1	MI0000784	-0.063240177	0.908635934	0.723571086	0.480789462	0.957324568	-4.865601569
hsa-miR-5186	MIMAT0021116	-0.063241094	0.963711954	0.528069407	0.60540398	0.975047002	-4.937851017
hsa-mir-3176	MI0014210	-0.063277227	1.043296837	0.612732725	0.549501255	0.965790957	-4.909131243
hsa-miR-1250-5p	MIMAT0005902	-0.063594014	0.971415486	0.710819205	0.488423624	0.959021141	-4.870944017
hsa-miR-647	MIMAT0003317	-0.063600338	0.823425706	0.68998099	0.501053122	0.960979619	-4.879489109
hsa-miR-676-3p	MIMAT0018204	-0.06371027	0.794923666	0.742721556	0.469460665	0.951768888	-4.857418015
hsa-miR-519e-3p	MIMAT0002829	-0.063767484	0.999505913	0.591068568	0.563535415	0.96620986	-4.916859828
hsa-mir-8059	MI0025895	-0.063997879	1.111941289	0.49822647	0.62576324	0.978652564	-4.947014202
hsa-mir-3674	MI0016075	-0.064107694	0.874945805	0.999701982	0.333774789	0.930208704	-4.729805011
hsa-miR-6876-3p	MIMAT0027653	-0.064242438	0.796029573	0.671271111	0.512554058	0.960979619	-4.886964271
hsa-mir-1287	MI0006349	-0.064338477	0.969292673	0.900552667	0.382475413	0.930573075	-4.782849372
hsa-miR-525-5p	MIMAT0002838	-0.064384066	0.852079907	0.515309844	0.614068641	0.976745744	-4.941830692
hsa-miR-203b-3p	MIMAT0019814	-0.064392212	0.853065796	0.728574997	0.477813502	0.955217368	-4.863481788
hsa-miR-129-2-3p	MIMAT0004605	-0.064421602	1.126045111	0.614074191	0.548638543	0.965790957	-4.908644183
hsa-miR-4635	MIMAT0019692	-0.064437094	0.842570072	0.917745303	0.373699907	0.930573075	-4.773983518
hsa-miR-221-5p	MIMAT0004568	-0.064484003	0.912006951	0.645548485	0.528611322	0.960979619	-4.896933985
hsa-miR-6740-5p	MIMAT0027381	-0.064544424	1.203228772	0.536557536	0.599673657	0.97307982	-4.935152586
hsa-miR-3074-3p	MIMAT0015027	-0.064622868	0.978399513	0.764431212	0.456817199	0.951465791	-4.847909795
hsa-miR-766-3p	MIMAT0003888	-0.064874116	2.479153981	0.270847064	0.790309282	0.994214304	-4.999855665
hsa-mir-662	MI0003670	-0.064882574	0.845169953	0.838342923	0.415384866	0.93437823	-4.813729849

hsa-mir-760	MI0005567	-0.065032416	0.896748398	0.794821523	0.439477663	0.946592983	-4.834191715
hsa-miR-4764-3p	MIMAT0019915	-0.065122816	1.022305612	0.671326887	0.512519548	0.960979619	-4.886942266
hsa-mir-3150a	MI0014177	-0.065128934	1.117155364	0.56106734	0.583281251	0.968332399	-4.927133136
hsa-mir-766	MI0003836	-0.065347437	1.042322648	0.66028312	0.519378731	0.960979619	-4.891266753
hsa-miR-758-3p	MIMAT0003879	-0.065366999	0.978051401	0.56691002	0.579407921	0.967979651	-4.925171748
hsa-miR-3927-5p	MIMAT0022970	-0.065388687	0.854115759	1.113076992	0.283708787	0.927057986	-4.663688288
hsa-mir-8088	MI0025924	-0.065579879	1.035048128	0.567769764	0.578839093	0.967979651	-4.924881523
hsa-mir-4661	MI0017289	-0.065600953	0.842951385	1.069170046	0.302392921	0.929573508	-4.689960585
hsa-miR-4659b-5p	MIMAT0019733	-0.065642558	0.824768042	0.656741047	0.521589734	0.960979619	-4.892639824
hsa-mir-3943	MI0016600	-0.065776879	0.996095628	0.855572299	0.406089489	0.930793333	-4.805367618
hsa-miR-376b-5p	MIMAT0022923	-0.066055748	0.73877198	0.795182373	0.439274312	0.946592983	-4.834025992
hsa-miR-7153-3p	MIMAT0028217	-0.066141228	0.825331599	1.264446521	0.225929671	0.919664114	-4.567031632
hsa-miR-8056	MIMAT0030983	-0.066225409	0.730630562	0.897848249	0.38386843	0.930573075	-4.78423105
hsa-let-7i	MI0000434	-0.066472597	0.894377442	0.978400309	0.343848783	0.930208704	-4.741587589
hsa-mir-5691	MI0019296	-0.066561065	0.842223592	0.652753384	0.524085275	0.960979619	-4.894177516
hsa-mir-99b	MI0000746	-0.066766531	1.050672254	0.764071458	0.45702498	0.951465791	-4.848069346
hsa-mir-4797	MI0017444	-0.066808749	0.924026355	0.827021487	0.42156797	0.937848766	-4.819144286
hsa-miR-3125	MIMAT0014988	-0.067403104	0.982810743	0.683398602	0.505082001	0.960979619	-4.882140324
hsa-miR-605-5p	MIMAT0003273	-0.067484149	1.063639464	1.020086716	0.324333194	0.930208704	-4.718336344
hsa-mir-2467	MI0017432	-0.06753584	0.892416958	1.000406539	0.33344522	0.930208704	-4.729411759
hsa-mir-548aj-1	MI0016814	-0.067603429	0.955937436	0.690013161	0.501033477	0.960979619	-4.879476094
hsa-miR-4757-3p	MIMAT0019902	-0.067678637	0.838403695	0.833029875	0.418279136	0.935967201	-4.816278765
hsa-miR-549a-3p	MIMAT0003333	-0.06779774	0.877598923	0.724964209	0.479959814	0.956472927	-4.865012728
hsa-mir-548ay	MI0022210	-0.067934124	0.886115907	0.644952268	0.528986843	0.960979619	-4.897160827
hsa-miR-323a-5p	MIMAT0004696	-0.068029152	1.138966145	0.484385372	0.635315462	0.981414312	-4.951091865
hsa-mir-3129	MI0014146	-0.068104081	0.982479012	0.985786602	0.340331581	0.930208704	-4.737525556
hsa-miR-30d-3p	MIMAT0004551	-0.068116744	0.847718293	0.757654484	0.460741037	0.951465791	-4.850904036
hsa-mir-6127	MI0021271	-0.068197418	0.944143415	0.856662138	0.405506161	0.930588488	-4.804833728
hsa-miR-6859-3p	MIMAT0027619	-0.068297536	0.950581867	0.676215096	0.509500267	0.960979619	-4.885007221
hsa-mir-1289-1	MI0006350	-0.068330159	1.169847059	0.424255633	0.677579108	0.985064407	-4.967526998
hsa-miR-518c-5p	MIMAT0002847	-0.068407312	0.844632262	1.023539194	0.322753319	0.930208704	-4.716375399
hsa-mir-29b-1	MI0000105	-0.068445474	0.975519429	1.181562409	0.25631282	0.92177216	-4.621093501
hsa-miR-6867-3p	MIMAT0027635	-0.068489378	0.982195868	0.921605872	0.371748461	0.930573075	-4.771973267
hsa-miR-7108-3p	MIMAT0028114	-0.068768673	1.223724739	0.427574981	0.675214778	0.984705521	-4.966674318
hsa-mir-4523	MI0016890	-0.069477937	1.283654771	0.421588984	0.679481079	0.985064407	-4.968207365
hsa-miR-5100	MIMAT0022259	-0.069609769	2.136390483	0.222730548	0.826838509	0.996080495	-5.007101564
hsa-miR-548c-5p	MIMAT0004806	-0.069650852	1.021943469	0.924121567	0.37048059	0.930573075	-4.770659489
hsa-miR-548o-5p	MIMAT0022738	-0.069650852	1.021943469	0.924121567	0.37048059	0.930573075	-4.770659489
hsa-miR-548am-5p	MIMAT0022740	-0.069650852	1.021943469	0.924121567	0.37048059	0.930573075	-4.770659489
hsa-miR-5689	MIMAT0022481	-0.069756065	0.927638015	0.837184004	0.416015066	0.934545952	-4.814287036
hsa-miR-379-3p	MIMAT0004690	-0.069787065	0.931438495	1.06276518	0.305192544	0.930208704	-4.693723513
hsa-mir-5706	MI0019314	-0.069954526	0.934134434	0.847320815	0.410523987	0.932736156	-4.809390814
hsa-mir-7705	MI0025241	-0.070200503	0.9983737	0.584369202	0.56791371	0.966465648	-4.919197287
hsa-miR-4709-5p	MIMAT0019811	-0.070382036	1.00606368	0.802197275	0.435333071	0.944702909	-4.830791214
hsa-miR-193b-5p	MIMAT0004767	-0.070679729	0.965382601	0.572031704	0.576023579	0.967644566	-4.923436722
hsa-miR-3686	MIMAT0018114	-0.070764441	0.852954032	0.723451497	0.480860721	0.957324568	-4.865652068
hsa-miR-5094	MIMAT0021086	-0.070882341	1.016814108	0.802322456	0.435262945	0.944629713	-4.830733262
hsa-miR-6881-5p	MIMAT0027662	-0.071046811	1.144938657	0.901055444	0.382216815	0.930573075	-4.782592116
hsa-miR-7702	MIMAT0030017	-0.071226815	0.921917671	0.925187982	0.369944031	0.930573075	-4.770101665
hsa-miR-3945	MIMAT0018361	-0.071266848	1.166892948	0.854197798	0.406825969	0.931395621	-4.806040124
hsa-miR-4538	MIMAT0019081	-0.071347202	1.137725651	0.46492073	0.648862728	0.982649571	-4.956640477
hsa-miR-658	MIMAT0003336	-0.071372276	0.943142826	0.605873217	0.553924212	0.96620986	-4.91160633
hsa-miR-6807-5p	MIMAT0027514	-0.071441775	1.004679254	0.543486239	0.595016312	0.971259148	-4.932919785
hsa-miR-3173-3p	MIMAT0015048	-0.071879106	1.225411778	0.754049599	0.462836792	0.951465791	-4.852487142
hsa-mir-8074	MI0025910	-0.071898151	0.937184331	1.036890459	0.316695956	0.930208704	-4.708741963
hsa-miR-3160-5p	MIMAT0019212	-0.072020852	0.909514608	0.652094561	0.524498227	0.960979619	-4.894430738
hsa-mir-3685	MI0016086	-0.072064746	1.045210286	0.703585762	0.492786018	0.960403858	-4.873936328
hsa-mir-587	MI0003595	-0.072065722	1.00801234	0.884935602	0.390566863	0.930573075	-4.790779335
hsa-miR-3658	MIMAT0018078	-0.07221412	0.998040345	0.901048357	0.38222046	0.930573075	-4.782595743
hsa-mir-6715a	MI0022548	-0.072295178	0.993761148	0.87089013	0.397941593	0.930573075	-4.797810087
hsa-miR-1295a	MIMAT0005885	-0.072317572	0.87408692	0.696995723	0.496780413	0.960979619	-4.876638336
hsa-miR-4267	MIMAT0016893	-0.072321586	0.826244088	1.020526609	0.324131587	0.930208704	-4.71808679
hsa-miR-6888-5p	MIMAT0027676	-0.072332155	0.897495879	1.100149009	0.289118391	0.927057986	-4.671509578
hsa-mir-7853	MI0025523	-0.072472922	0.992721012	1.092663952	0.292285374	0.927057986	-4.676005451
hsa-miR-4323	MIMAT0016875	-0.072536698	1.070950572	0.740426775	0.470809527	0.952023826	-4.858408752
hsa-miR-378j	MIMAT0024612	-0.072695516	0.822529839	0.863004697	0.402122351	0.930573075	-4.801715017

hsa-miR-548ar-3p	MIMAT0022266	-0.072696543	1.009113246	0.957985484	0.353703003	0.930573075	-4.752683471
hsa-miR-3648	MIMAT0018068	-0.072710875	2.744661794	0.136112861	0.893595503	0.999057213	-5.016610163
hsa-miR-4314	MIMAT0016868	-0.072750492	0.951693569	0.86467277	0.401235538	0.930573075	-4.800891519
hsa-miR-3074-5p	MIMAT0019208	-0.072906001	0.859492796	1.05140167	0.31020632	0.930208704	-4.700355543
hsa-miR-6750-3p	MIMAT0027401	-0.073099842	1.001494024	0.946407472	0.359378638	0.930573075	-4.758890138
hsa-miR-4445-3p	MIMAT0018964	-0.073244048	0.830861603	0.910363062	0.377450974	0.930573075	-4.777807734
hsa-miR-6128	MIMAT0024611	-0.073408892	0.866700514	0.919909795	0.372604935	0.930573075	-4.772857312
hsa-miR-1266-3p	MIMAT0026742	-0.073495857	0.87307869	0.767912504	0.454809568	0.951465791	-4.846362396
hsa-mir-635	MI0003650	-0.07362289	0.940147803	0.818402649	0.426314889	0.9417163	-4.823223204
hsa-miR-302a-5p	MIMAT0000683	-0.073628719	0.893983703	0.810574271	0.430656209	0.94301493	-4.82689566
hsa-mir-5704	MI0019312	-0.073938035	0.93021139	0.793946176	0.439971196	0.946910953	-4.834593447
hsa-mir-7703	MI0025239	-0.074002522	0.964558056	0.816990109	0.427096137	0.942182528	-4.823888138
hsa-mir-335	MI0000816	-0.074058716	1.000054593	0.50789769	0.619129643	0.977427364	-4.944100114
hsa-miR-642a-5p	MIMAT0003312	-0.074066874	0.929216115	0.984737831	0.340829427	0.930208704	-4.738103846
hsa-miR-19b-2-5p	MIMAT0004492	-0.074091546	0.793703187	0.785824009	0.444567347	0.948380773	-4.838302427
hsa-mir-4745	MI0017384	-0.074246671	0.981057387	0.923511257	0.370787904	0.930573075	-4.77097849
hsa-mir-944	MI0005769	-0.074286957	0.92991183	0.786778608	0.444025594	0.948115267	-4.837868256
hsa-mir-1302-6	MI0006367	-0.074298207	0.924386036	1.108616474	0.285566634	0.927057986	-4.666394859
hsa-miR-4662b	MIMAT0019736	-0.074461688	0.896289472	0.933933333	0.365564016	0.930573075	-4.765506749
hsa-miR-4744	MIMAT0019875	-0.074595586	0.946297969	0.823398409	0.423559231	0.939568509	-4.820863471
hsa-miR-8059	MIMAT0030986	-0.074640421	0.821712954	0.919078576	0.373025171	0.930573075	-4.773290067
hsa-miR-600	MIMAT0003268	-0.074969496	1.00067697	1.178137638	0.257632927	0.922824015	-4.623269122
hsa-miR-4728-5p	MIMAT0019849	-0.074993361	2.448937597	0.254935121	0.802337752	0.994214304	-5.002406285
hsa-miR-6769b-3p	MIMAT0027621	-0.07501265	1.005290726	0.68427166	0.504546545	0.960979619	-4.881790009
hsa-miR-7159-5p	MIMAT0028228	-0.075100712	0.932464908	0.682436446	0.505672484	0.960979619	-4.882525916
hsa-miR-7976	MIMAT0031179	-0.07527005	0.784874946	0.956190264	0.354578912	0.930573075	-4.753649947
hsa-mir-1263	MI0006398	-0.075285942	1.044924519	0.878020398	0.394186233	0.930573075	-4.794252909
hsa-miR-3189-5p	MIMAT0019217	-0.075339152	1.008519539	1.093187439	0.292063048	0.927057986	-4.675691799
hsa-miR-7978	MIMAT0031181	-0.07545401	0.804020795	0.962916941	0.351304677	0.930573075	-4.750020838
hsa-mir-4485	MI0016846	-0.075558346	2.422622927	0.400774619	0.694403529	0.98554565	-4.973375354
hsa-miR-2114-3p	MIMAT0011157	-0.075669496	0.865522578	1.03944241	0.315547597	0.930208704	-4.707273876
hsa-miR-4474-3p	MIMAT0019001	-0.075686222	0.780233026	0.859550418	0.403962912	0.930573075	-4.803415984
hsa-miR-6728-3p	MIMAT0027358	-0.075798489	0.900075781	0.73854013	0.471920259	0.952023826	-4.859221226
hsa-miR-4667-3p	MIMAT0019744	-0.075895031	0.932963976	0.71078957	0.488441449	0.959021141	-4.870956332
hsa-miR-873-5p	MIMAT0004953	-0.075940247	0.76657353	0.694947561	0.498025736	0.960979619	-4.877473412
hsa-miR-4436b-3p	MIMAT0019941	-0.07596389	0.867757267	0.554931136	0.587363492	0.968332399	-4.929172502
hsa-miR-3675-5p	MIMAT0018098	-0.076164934	0.959077818	0.797204322	0.438135985	0.946344991	-4.833096175
hsa-miR-3650	MIMAT0018070	-0.076229483	0.908838619	1.282274315	0.219780233	0.917147059	-4.555059149
hsa-mir-663b	MI0006336	-0.076494087	1.011197775	0.825000232	0.422678112	0.938816973	-4.820104202
hsa-miR-6837-3p	MIMAT0027577	-0.076598103	0.855131065	0.810591624	0.430646555	0.94301493	-4.826887553
hsa-miR-548ay-5p	MIMAT0025452	-0.076739442	1.001251811	1.03184158	0.318976836	0.930208704	-4.711637951
hsa-miR-4424	MIMAT0018939	-0.077101231	0.935799551	1.379160002	0.188656315	0.903156384	-4.487976125
hsa-miR-135b-5p	MIMAT0000758	-0.077126663	0.830864863	0.940963522	0.362069066	0.930573075	-4.761786768
hsa-mir-1246	MI0006381	-0.077306997	1.004111799	0.777480085	0.449320403	0.950247229	-4.842077573
hsa-miR-6854-5p	MIMAT0027608	-0.077473971	1.029129164	1.111361193	0.28442236	0.927057986	-4.664730403
hsa-mir-7109	MI0022960	-0.077477734	0.927696017	1.25216239	0.230245343	0.919664114	-4.575211622
hsa-mir-4738	MI0017376	-0.077541724	0.891702835	1.093987539	0.291723487	0.927057986	-4.675212186
hsa-miR-6076	MIMAT0023701	-0.077593191	1.050325327	0.835434318	0.416967698	0.935045754	-4.815126985
hsa-miR-9-5p	MIMAT0000441	-0.077593803	0.815375268	1.214242213	0.243976427	0.919664114	-4.600097956
hsa-mir-3202-1	MI0014252	-0.077606018	0.807493375	0.91109476	0.377078041	0.930573075	-4.777429857
hsa-miR-3200-5p	MIMAT0017392	-0.077706613	1.057819922	0.861680926	0.402827045	0.930573075	-4.802367567
hsa-mir-4314	MI0015846	-0.077963309	1.148243959	0.740880309	0.470542755	0.952023826	-4.858213163
hsa-mir-103b-2	MI0007262	-0.078022007	0.855775981	1.385218392	0.186835451	0.903156384	-4.483671956
hsa-miR-1296-3p	MIMAT0026637	-0.078144309	0.838288665	1.092589046	0.292317197	0.927057986	-4.676050321
hsa-mir-6815	MI0022660	-0.078175917	0.826189527	1.090547038	0.293185713	0.927057986	-4.67727262
hsa-miR-6774-3p	MIMAT0027449	-0.078204726	0.866547018	0.973271792	0.346305924	0.930573075	-4.744393197
hsa-miR-532-3p	MIMAT0004780	-0.078276389	1.66965877	0.508297184	0.618856356	0.977382438	-4.943978595
hsa-miR-450a-1-3p	MIMAT0022700	-0.078800731	0.829066502	0.963202587	0.351166108	0.930573075	-4.749866262
hsa-miR-2110	MIMAT0010133	-0.078981138	4.113802876	0.202297113	0.842482995	0.996080495	-5.009755487
hsa-let-7d	MI0000065	-0.079184705	0.979335116	1.045838651	0.312682634	0.930208704	-4.703581564
hsa-miR-6779-5p	MIMAT0027458	-0.079523201	6.872699602	0.473629205	0.642785412	0.982649571	-4.954184935
hsa-miR-148a-5p	MIMAT0004549	-0.07966722	0.973885666	0.899493322	0.383020661	0.930573075	-4.783391007
hsa-mir-1343	MI0017320	-0.079674438	0.940203187	0.808821167	0.43163229	0.94301493	-4.827713842
hsa-miR-6716-3p	MIMAT0025845	-0.079951084	0.888076912	1.392850418	0.184562074	0.903156384	-4.478232072
hsa-mir-4291	MI0015900	-0.079988993	1.06879574	0.7445192	0.468405669	0.951465791	-4.856639991
hsa-miR-8054	MIMAT0030981	-0.080089884	0.929736581	1.232370525	0.237334555	0.919664114	-4.588269981

hsa-miR-374a-3p	MIMAT0004688	-0.080176423	0.867681796	0.977024336	0.344506819	0.930522809	-4.74234152
hsa-mir-548f-4	MI0006377	-0.080424025	0.9805845	0.766101434	0.45585331	0.951465791	-4.847168178
hsa-mir-6130	MI0021275	-0.080492521	1.060577039	1.179124327	0.257252066	0.922824015	-4.622642802
hsa-miR-4425	MIMAT0018940	-0.080594576	0.917497542	1.164072879	0.263108997	0.924582056	-4.632154221
hsa-miR-4429	MIMAT0018944	-0.080601192	8.281805021	0.281257386	0.782469283	0.994214304	-4.998104595
hsa-miR-551b-3p	MIMAT0003233	-0.080730346	1.04321185	0.963921954	0.350817307	0.930573075	-4.749476814
hsa-miR-3674	MIMAT0018097	-0.080927846	1.005430732	0.878875797	0.39373731	0.930573075	-4.793824496
hsa-miR-548f-5p	MIMAT0026739	-0.081077463	0.869814449	1.078083042	0.298528471	0.927057986	-4.684694424
hsa-miR-196a-5p	MIMAT0000226	-0.081101175	0.959428408	1.58251779	0.134998857	0.896940866	-4.33708723
hsa-mir-4427	MI0016766	-0.081382429	0.861786896	0.98334718	0.341490356	0.930208704	-4.738869869
hsa-miR-4330	MIMAT0016924	-0.081495472	0.876100657	1.117248665	0.281979447	0.927057986	-4.66114938
hsa-miR-557	MIMAT0003221	-0.081576756	1.018230651	0.859448816	0.404017133	0.930573075	-4.803465926
hsa-mir-6129	MI0021274	-0.081649035	0.961615743	1.039327575	0.315599207	0.930208704	-4.707340001
hsa-mir-4462	MI0016810	-0.081878193	1.05272844	0.977047981	0.344495504	0.930522809	-4.742328571
hsa-miR-92a-2-5p	MIMAT0004508	-0.081897668	0.840244046	0.858265339	0.404649067	0.930588488	-4.804047286
hsa-miR-598-5p	MIMAT0026620	-0.081935804	1.03656407	0.617210318	0.546624555	0.965790957	-4.907501664
hsa-miR-4781-5p	MIMAT0019942	-0.082087632	0.879673999	1.070337735	0.301884556	0.929265009	-4.68927263
hsa-miR-1295b-5p	MIMAT0022293	-0.08215565	0.921435947	0.996661213	0.33519983	0.930208704	-4.731499639
hsa-mir-3915	MI0016420	-0.082308887	0.977486072	1.033966573	0.318015404	0.930208704	-4.710420458
hsa-mir-5682	MI0019282	-0.082491297	0.845246389	1.098894724	0.2896473	0.927057986	-4.672264628
hsa-miR-486-3p	MIMAT0004762	-0.082519432	4.189455298	0.236448369	0.816377748	0.994603723	-5.005178121
hsa-mir-1295b	MI0019146	-0.082674807	0.865866951	1.054052404	0.309031424	0.930208704	-4.698813576
hsa-miR-7155-5p	MIMAT0028220	-0.082818639	0.753604888	1.152982679	0.267489211	0.927057986	-4.639103462
hsa-miR-6733-5p	MIMAT0027367	-0.082871938	1.022105165	1.145034921	0.270662213	0.927057986	-4.644052588
hsa-mir-6740	MI0022585	-0.08338395	0.997733538	1.322362766	0.206437729	0.910046702	-4.527708859
hsa-mir-1260b	MI0014197	-0.083456586	1.123221134	0.710364094	0.488697415	0.959021141	-4.871133102
hsa-mir-591	MI0003603	-0.08349423	0.883571562	0.875426937	0.395549402	0.930573075	-4.79554962
hsa-miR-6798-3p	MIMAT0027497	-0.083619774	1.427566572	0.739384387	0.471423019	0.952023826	-4.858857881
hsa-miR-6727-3p	MIMAT0027356	-0.083761962	1.186101924	0.960200875	0.352624175	0.930573075	-4.75148872
hsa-mir-6729	MI0022574	-0.083773474	1.196964577	0.706664149	0.490926665	0.959629992	-4.872666254
hsa-mir-5089	MI0017978	-0.083801823	0.902169278	0.902517395	0.381465552	0.930573075	-4.781843388
hsa-miR-1537-3p	MIMAT0007399	-0.083878957	1.088901831	0.848240229	0.410028308	0.932647366	-4.808944201
hsa-miR-7160-5p	MIMAT0028230	-0.083938417	1.188697689	0.76919946	0.454068785	0.951465791	-4.845788776
hsa-mir-135a-2	MI0000453	-0.084548455	1.036106462	1.08032742	0.297561134	0.927057986	-4.683362931
hsa-mir-4663	MI0017292	-0.084581465	0.867468014	0.946039507	0.35956005	0.930573075	-4.759086365
hsa-miR-345-3p	MIMAT0022698	-0.084780208	1.113028467	1.029204715	0.32017278	0.930208704	-4.713145917
hsa-miR-6771-3p	MIMAT0027443	-0.085218153	0.977561125	1.131990967	0.275931456	0.927057986	-4.652118567
hsa-miR-519d-5p	MIMAT0026610	-0.085232755	0.97257052	1.008197841	0.329816169	0.930208704	-4.725047983
hsa-mir-572	MI0003579	-0.085271224	1.315709491	0.604351803	0.554907814	0.96620986	-4.912151787
hsa-miR-4795-3p	MIMAT0019969	-0.085338624	0.909921697	0.974962112	0.345494706	0.930573075	-4.743469831
hsa-miR-6505-5p	MIMAT0025466	-0.085380408	0.98985976	1.228340945	0.23879859	0.919664114	-4.590910159
hsa-miR-302d-5p	MIMAT0004685	-0.085482513	1.038902081	1.108591721	0.285576969	0.927057986	-4.666409855
hsa-mir-4273	MI0015881	-0.085493004	1.053732418	1.082405227	0.296667655	0.927057986	-4.682128319
hsa-miR-3689d	MIMAT0019008	-0.085612281	0.922562091	0.963518434	0.351012933	0.930573075	-4.7496953
hsa-mir-4282	MI0015890	-0.085627628	1.004622869	0.887316703	0.389325793	0.930573075	-4.789577903
hsa-miR-8072	MIMAT0030999	-0.085633055	8.584918985	0.214482475	0.833144759	0.996080495	-5.008203275
hsa-mir-5696	MI0019303	-0.085675285	0.852294463	1.781170648	0.095743204	0.896940866	-4.178552423
hsa-miR-520a-5p	MIMAT0002833	-0.085817425	0.856634198	1.335741192	0.202132337	0.905221726	-4.518452083
hsa-miR-3944-3p	MIMAT0018360	-0.085837087	1.12050745	0.678774228	0.507923693	0.960979619	-4.883989054
hsa-mir-501	MI0003185	-0.085838674	1.017475335	1.252455363	0.230141666	0.919664114	-4.575017197
hsa-mir-4655	MI0017283	-0.085872309	1.021602033	0.809600431	0.431198242	0.94301493	-4.827350347
hsa-miR-5189-3p	MIMAT0027088	-0.085927906	0.976828984	0.674089841	0.510811699	0.960979619	-4.885850099
hsa-miR-7843-3p	MIMAT0030412	-0.085977195	1.023579641	0.811596704	0.430087601	0.94301493	-4.826417769
hsa-miR-3713	MIMAT0018164	-0.086127903	1.036324632	0.820540659	0.425134151	0.940652535	-4.822214856
hsa-mir-6839	MI0022685	-0.086207543	0.882643618	1.301699485	0.213231749	0.91645315	-4.541879646
hsa-miR-6893-3p	MIMAT0027687	-0.086239965	0.965420883	1.004952247	0.33132446	0.930208704	-4.72686913
hsa-mir-210	MI0000286	-0.086318929	1.070944622	0.983869682	0.341241922	0.930208704	-4.738582159
hsa-miR-5010-5p	MIMAT0021043	-0.086345461	1.430331638	0.651476355	0.524885888	0.960979619	-4.894668135
hsa-miR-4646-5p	MIMAT0019707	-0.086427123	0.971208159	0.707893797	0.490185122	0.95960537	-4.872157526
hsa-mir-4755	MI0017395	-0.086465275	0.96190897	0.934589463	0.365236848	0.930573075	-4.765160549
hsa-miR-1267	MIMAT0005921	-0.086632039	0.866483871	1.123642434	0.279344311	0.927057986	-4.657243877
hsa-miR-7158-5p	MIMAT0028226	-0.086670573	0.864953043	0.924453514	0.370313516	0.930573075	-4.770485911
hsa-miR-6773-5p	MIMAT0027446	-0.086679645	1.068731988	0.912988607	0.37611395	0.930573075	-4.776450612
hsa-miR-1321	MIMAT0005952	-0.08676092	0.858887559	1.203526076	0.247969973	0.919664114	-4.60702932
hsa-miR-205-3p	MIMAT0009197	-0.086840332	0.862363508	1.000175437	0.333553296	0.930208704	-4.729540774
hsa-mir-3649	MI0016049	-0.087026475	0.85516015	1.256208488	0.22881676	0.919664114	-4.572523643

hsa-miR-3159	MIMAT0015033	-0.087056051	0.929511525	0.985764186	0.340342216	0.930208704	-4.737537921
hsa-mir-6717	MI0022551	-0.087378702	0.930954396	0.776865446	0.449671784	0.950247229	-4.842354252
hsa-mir-1299	MI0006359	-0.087434046	1.127854156	0.509269577	0.618191404	0.977329171	-4.94368243
hsa-miR-4533	MIMAT0019072	-0.087489723	1.156499368	0.794357469	0.43973926	0.946841421	-4.834404737
hsa-mir-224	MI0000301	-0.087500748	0.940289407	1.053856934	0.309117952	0.930208704	-4.698927388
hsa-miR-4790-3p	MIMAT0019962	-0.087512592	0.981672628	0.93445895	0.36530191	0.930573075	-4.765229429
hsa-mir-187	MI0000274	-0.08797608	0.974761218	0.99804563	0.334550494	0.930208704	-4.730728622
hsa-miR-4659b-3p	MIMAT0019734	-0.088111092	0.777466778	1.255352588	0.229118375	0.919664114	-4.573092769
hsa-miR-3617-3p	MIMAT0022966	-0.088116304	1.01113335	1.091119428	0.292942069	0.927057986	-4.676930181
hsa-mir-3529	MI0017351	-0.08813025	0.83608699	1.051279367	0.310260608	0.930208704	-4.700426614
hsa-miR-4504	MIMAT0019040	-0.088210175	0.897365976	0.916180167	0.374493047	0.930573075	-4.77479648
hsa-mir-4477a	MI0016829	-0.088403955	0.918014519	1.096000211	0.290870609	0.927057986	-4.674004495
hsa-miR-5195-5p	MIMAT0021126	-0.088503033	1.190889402	0.865054051	0.401033017	0.930573075	-4.800703096
hsa-mir-378i	MI0016902	-0.088515559	0.873226279	0.882610583	0.391781264	0.930573075	-4.791949812
hsa-miR-4520-5p	MIMAT0019235	-0.088592223	0.737285826	1.112180325	0.284081528	0.927057986	-4.664233046
hsa-miR-4682	MIMAT0019767	-0.088734093	1.012827502	0.894712487	0.385487922	0.930573075	-4.785828681
hsa-miR-4668-3p	MIMAT0019746	-0.088820183	0.956767283	1.096800052	0.290532188	0.927057986	-4.673524075
hsa-mir-1245b	MI0017431	-0.088827006	0.942649493	1.053873045	0.30911082	0.930208704	-4.698918009
hsa-miR-4690-3p	MIMAT0019780	-0.088875617	0.864492149	1.50610835	0.153417228	0.903040236	-4.395271987
hsa-mir-5690	MI0019295	-0.088921861	0.864293475	0.963017122	0.351256074	0.930573075	-4.749966629
hsa-miR-708-5p	MIMAT0004926	-0.089085072	0.988902498	1.018097611	0.325245944	0.930208704	-4.71946369
hsa-mir-5692b	MI0019311	-0.0890857	0.842357184	1.126557982	0.278148854	0.927057986	-4.655457282
hsa-miR-5088-5p	MIMAT0021080	-0.089117228	1.087042331	0.886879713	0.389553361	0.930573075	-4.789798601
hsa-miR-548b-3p	MIMAT0003254	-0.089254688	0.859319729	1.04466331	0.31320766	0.930208704	-4.704261403
hsa-mir-4633	MI0017260	-0.089278213	0.847938855	1.497359413	0.155655333	0.903074508	-4.401824302
hsa-miR-4650-5p	MIMAT0019713	-0.089485219	0.832969084	1.019338173	0.324676465	0.930208704	-4.718760797
hsa-mir-8058	MI0025894	-0.089511291	0.762966711	1.279797045	0.220626711	0.917147059	-4.556729891
hsa-miR-4529-5p	MIMAT0019236	-0.089533149	0.786145008	1.12814641	0.277499176	0.927057986	-4.654482426
hsa-let-7f-1	MI0000067	-0.089606509	0.970401503	1.091078594	0.292959445	0.927057986	-4.676954615
hsa-mir-4791	MI0017438	-0.08981126	0.8547535	1.342023082	0.200135822	0.905221726	-4.514083573
hsa-miR-3158-5p	MIMAT0019211	-0.089981885	0.841721588	1.124047806	0.279177866	0.927057986	-4.656995686
hsa-miR-572	MIMAT0003237	-0.090044198	4.398530386	0.208374001	0.837822822	0.996080495	-5.008992642
hsa-miR-3115	MIMAT0014977	-0.090143069	0.891841928	1.272452383	0.223151557	0.918415347	-4.561669902
hsa-miR-3912-5p	MIMAT0027036	-0.090278501	0.8185264	1.050479662	0.310615749	0.930208704	-4.700891163
hsa-miR-5701	MIMAT0022494	-0.090302387	0.772618463	1.0642383	0.304546952	0.930208704	-4.692859623
hsa-miR-4291	MIMAT0016922	-0.090332986	0.884311825	1.210239035	0.2454624	0.919664114	-4.602692561
hsa-miR-4697-3p	MIMAT0019792	-0.090403497	1.001206341	1.07298291	0.300735277	0.928625668	-4.687712008
hsa-miR-8057	MIMAT0030984	-0.090415149	0.796888107	1.218084371	0.242556802	0.919664114	-4.597601805
hsa-mir-6829	MI0022674	-0.090452134	1.019399028	1.268995941	0.224347647	0.919588975	-4.563987722
hsa-miR-455-5p	MIMAT0003150	-0.090663543	0.87487253	1.379922537	0.188426341	0.903156384	-4.487435072
hsa-mir-4636	MI0017263	-0.090768683	0.929866974	1.372605803	0.190642457	0.903156384	-4.492618428
hsa-mir-6131	MI0021276	-0.090839468	1.083816648	0.657347581	0.521210749	0.960979619	-4.892405185
hsa-miR-6740-3p	MIMAT0027382	-0.090896755	0.948877028	0.805176727	0.433665946	0.943789399	-4.829409748
hsa-mir-4799	MI0017446	-0.090902046	0.867299267	0.884154814	0.390974401	0.930573075	-4.791172698
hsa-miR-4766-3p	MIMAT0019918	-0.091035329	0.798395802	1.481522257	0.159776338	0.903074508	-4.413625715
hsa-miR-6816-5p	MIMAT0027532	-0.09115813	6.74061636	0.283373574	0.780878537	0.994214304	-4.997740688
hsa-miR-99b-5p	MIMAT0000689	-0.091283396	5.080712269	0.136585912	0.893228146	0.999057213	-5.016570639
hsa-mir-6778	MI0022623	-0.091305433	0.99085599	0.936533844	0.364268506	0.930573075	-4.764133423
hsa-miR-6515-5p	MIMAT0025486	-0.091353538	1.299628257	0.632937154	0.536586332	0.96159396	-4.90169101
hsa-mir-4728	MI0017365	-0.091524919	1.059499415	1.26661675	0.225173896	0.919664114	-4.565580553
hsa-mir-5583-2	MI0019140	-0.09153044	0.9581587	1.398300873	0.182952417	0.903156384	-4.47433512
hsa-miR-423-3p	MIMAT0001340	-0.091602499	5.272177684	0.258408393	0.799707625	0.994214304	-5.00186253
hsa-miR-6815-3p	MIMAT0027531	-0.09172248	0.836760475	1.306053331	0.211785562	0.91645315	-4.538906677
hsa-miR-4640-3p	MIMAT0019700	-0.091849822	0.908304802	1.263691652	0.22619302	0.919664114	-4.567535944
hsa-miR-3606-3p	MIMAT0022965	-0.092137875	1.054558772	1.733300415	0.104152412	0.896940866	-4.217631272
hsa-miR-4686	MIMAT0019773	-0.092244906	0.994275058	0.798625827	0.437336822	0.945720391	-4.832441236
hsa-miR-6796-5p	MIMAT0027492	-0.092275325	2.2692789	0.214411615	0.833198989	0.996080495	-5.008212561
hsa-miR-4492	MIMAT0019027	-0.092367418	5.902296052	0.24914208	0.806730016	0.994603723	-5.003297043
hsa-mir-2114	MI0010633	-0.092389529	1.02144769	0.841575967	0.413630088	0.93368138	-4.812171926
hsa-miR-1179	MIMAT0005824	-0.092418249	0.861669457	1.332335074	0.203221563	0.905221726	-4.520814894
hsa-miR-4688	MIMAT0019777	-0.092669911	1.470400349	0.427039979	0.675595615	0.984721037	-4.966812184
hsa-miR-6851-3p	MIMAT0027603	-0.092690089	0.88820329	1.032927792	0.318485127	0.930208704	-4.711015868
hsa-miR-5589-3p	MIMAT0022298	-0.092721381	0.937067203	1.26242271	0.226636258	0.919664114	-4.568383213
hsa-miR-3116	MIMAT0014978	-0.092914196	0.825412236	1.086130869	0.295070561	0.927057986	-4.679909919
hsa-miR-4677-5p	MIMAT0019760	-0.092927786	0.853591289	1.288138656	0.217786668	0.917147059	-4.551094998
hsa-mir-6757	MI0022602	-0.092984608	1.047538667	1.14893874	0.269100134	0.927057986	-4.641624901

hsa-miR-6809-5p	MIMAT0027518	-0.09309592	0.806573561	1.295296159	0.215372998	0.917147059	-4.546239487
hsa-miR-4730	MIMAT0019852	-0.093352842	0.848416855	0.997994026	0.334574682	0.930208704	-4.730757377
hsa-miR-8076	MIMAT0031003	-0.093736992	0.88841244	1.027165365	0.321099943	0.930208704	-4.714310049
hsa-mir-4785	MI0017430	-0.093792035	1.673331532	0.762575771	0.457889462	0.951465791	-4.848731965
hsa-miR-4797-3p	MIMAT0019973	-0.093902308	0.907065637	0.860607014	0.403399329	0.930573075	-4.802896315
hsa-miR-4703-3p	MIMAT0019802	-0.093902713	0.869019276	1.065413044	0.304032842	0.930208704	-4.692170033
hsa-miR-5681a	MIMAT0022469	-0.094032036	0.99311427	0.822892394	0.423837824	0.939877364	-4.821103056
hsa-miR-205-5p	MIMAT0000266	-0.094165271	0.816738356	1.201301991	0.248805119	0.919664114	-4.608462217
hsa-miR-3189-3p	MIMAT0015071	-0.094218204	1.037044871	1.002561711	0.332438539	0.930208704	-4.728207439
hsa-miR-4758-3p	MIMAT0019904	-0.094221489	1.250983405	0.788861745	0.442844824	0.947408294	-4.836919186
hsa-mir-4303	MI0015834	-0.094320731	0.849086447	1.395410962	0.183804443	0.903156384	-4.476402585
hsa-miR-3616-5p	MIMAT0017995	-0.09460219	0.847396861	1.263496657	0.226261086	0.919664114	-4.56766618
hsa-mir-3192	MI0014237	-0.094614884	1.020806439	0.947125816	0.359024668	0.930573075	-4.75850688
hsa-mir-3683	MI0016084	-0.094852946	0.903322202	1.370854362	0.191176074	0.903697113	-4.49385647
hsa-miR-4295	MIMAT0016844	-0.094868759	0.834834842	1.257620005	0.22832003	0.919664114	-4.571584456
hsa-miR-3619-5p	MIMAT0017999	-0.09492097	2.232483	0.669259476	0.513799615	0.960979619	-4.88775681
hsa-miR-4670-3p	MIMAT0019751	-0.095054491	0.907520661	0.978319655	0.34388733	0.930208704	-4.741631805
hsa-miR-454-5p	MIMAT0003884	-0.095284126	1.016544965	1.200007868	0.249292063	0.919664114	-4.609295073
hsa-miR-4709-3p	MIMAT0019812	-0.09543573	1.04190611	1.238683535	0.235055004	0.919664114	-4.584121089
hsa-miR-4269	MIMAT0016897	-0.095436182	0.904396281	1.33021059	0.20390333	0.905730418	-4.52228655
hsa-miR-514b-3p	MIMAT0015088	-0.095615094	0.77369331	1.237710542	0.23540522	0.919664114	-4.584761539
hsa-miR-4690-5p	MIMAT0019779	-0.095632215	3.92206299	0.159968799	0.875103015	0.99850194	-5.014446834
hsa-mir-4750	MI0017389	-0.095879096	1.206835998	1.030970485	0.319371561	0.930208704	-4.712136455
hsa-miR-550a-3p	MIMAT0003257	-0.095967191	0.911876473	1.431843317	0.173298167	0.903074508	-4.450135562
hsa-miR-6773-3p	MIMAT0027447	-0.096138496	0.880234118	1.621510169	0.126349709	0.896940866	-4.306760208
hsa-mir-1228	MI0006318	-0.096230186	1.013892866	1.517863756	0.15045266	0.902932883	-4.386431693
hsa-mir-5047	MI0017932	-0.096297259	0.901213742	1.221136463	0.241433678	0.919664114	-4.595614826
hsa-miR-1307-5p	MIMAT0022727	-0.096400235	1.05617395	0.797252891	0.438108664	0.946344991	-4.833073815
hsa-miR-1470	MIMAT0007348	-0.096467713	1.164281018	0.806603431	0.432869093	0.94301493	-4.828746645
hsa-miR-3154	MIMAT0015028	-0.096498546	1.181644212	0.557790702	0.585459285	0.968332399	-4.928224749
hsa-mir-5582	MI0019138	-0.096626667	0.971185778	1.447612871	0.168906822	0.903074508	-4.438631355
hsa-miR-548ax	MIMAT0022474	-0.09667341	1.010647943	1.188447275	0.253674713	0.921246697	-4.616705567
hsa-miR-5588-3p	MIMAT0022296	-0.096689573	1.081412499	0.949056085	0.358074713	0.930573075	-4.757475823
hsa-mir-3936	MI0016592	-0.096886473	0.933039169	0.946345957	0.359408962	0.930573075	-4.758922947
hsa-miR-15b-3p	MIMAT0004586	-0.097337013	0.904418237	1.586636782	0.13406189	0.896940866	-4.333903281
hsa-miR-489-5p	MIMAT0026605	-0.097667632	1.016208184	1.254172741	0.229534661	0.919664114	-4.573876845
hsa-miR-519b-3p	MIMAT0002837	-0.097733509	0.947670492	0.979573005	0.343288661	0.930208704	-4.740944349
hsa-miR-5003-5p	MIMAT0021025	-0.097843136	0.860244908	1.464249029	0.16437457	0.903074508	-4.426408675
hsa-miR-8058	MIMAT0030985	-0.097876	0.859635725	1.89747943	0.077768735	0.896940866	-4.081586221
hsa-mir-4669	MI0017300	-0.097983064	1.159099458	1.209439381	0.24576007	0.919664114	-4.603210092
hsa-mir-5004	MI0017870	-0.098014265	1.03830428	0.92971148	0.367674001	0.930573075	-4.767729489
hsa-miR-4768-3p	MIMAT0019921	-0.098165815	0.93995853	1.358792905	0.194883927	0.904897643	-4.502353615
hsa-miR-144-3p	MIMAT0000436	-0.09826691	0.867031674	1.126622425	0.278122474	0.927057986	-4.655417752
hsa-miR-302e	MIMAT0005931	-0.098388027	0.930817129	1.336376798	0.201929601	0.905221726	-4.51801071
hsa-miR-652-5p	MIMAT0022709	-0.09848807	1.221091061	0.707035383	0.490702721	0.95960537	-4.872512752
hsa-miR-551b-5p	MIMAT0004794	-0.098550294	1.313509247	0.93005824	0.367500382	0.930573075	-4.767547244
hsa-miR-3974	MIMAT0019359	-0.098574504	1.006427448	1.389194396	0.185648268	0.903156384	-4.480840436
hsa-mir-6878	MI0022725	-0.09870789	0.883745214	1.719777486	0.106642575	0.896940866	-4.228575692
hsa-mir-889	MI0005540	-0.098788553	0.845939746	1.439997469	0.171015837	0.903074508	-4.444196959
hsa-miR-4262	MIMAT0016894	-0.098879412	0.81792756	1.649950809	0.120345638	0.896940866	-4.284383576
hsa-mir-431	MI0001721	-0.098938222	1.113208186	1.577923726	0.136050482	0.896940866	-4.340632835
hsa-miR-323a-3p	MIMAT0000755	-0.098993465	0.891063992	0.988097174	0.339236584	0.930208704	-4.736249731
hsa-miR-515-3p	MIMAT0002827	-0.099453998	0.719445502	1.234470501	0.23657437	0.919664114	-4.586891589
hsa-miR-216a-3p	MIMAT0022844	-0.099800879	0.95951424	0.845409323	0.411555781	0.93320603	-4.810317996
hsa-miR-4321	MIMAT0016874	-0.099899279	1.034097546	0.937269673	0.363902512	0.930573075	-4.763744253
hsa-miR-522-3p	MIMAT0002868	-0.099985206	0.942725661	1.096721699	0.290565327	0.927057986	-4.673571149
hsa-miR-3622a-3p	MIMAT0018004	-0.10024843	0.960876427	1.237902529	0.235336084	0.919664114	-4.584635197
hsa-miR-3130-5p	MIMAT0014995	-0.100251333	0.970922427	0.985208997	0.340605703	0.930208704	-4.737844108
hsa-miR-3668	MIMAT0018091	-0.100332029	0.8674548	1.445546498	0.169476943	0.903074508	-4.440143375
hsa-mir-5010	MI0017878	-0.100560335	1.004187636	1.507333226	0.153106055	0.902932883	-4.394352795
hsa-miR-6841-3p	MIMAT0027585	-0.100648352	0.903616566	1.155127661	0.266637726	0.927057986	-4.637763318
hsa-miR-3646	MIMAT0018065	-0.100900407	1.148844403	1.538630769	0.145333445	0.896940866	-4.370714069
hsa-mir-6083	MI0020360	-0.100923721	0.967190509	1.257007856	0.228535348	0.919664114	-4.571991857
hsa-miR-520e-3p	MIMAT0002825	-0.101137554	0.971780292	1.068575086	0.302652185	0.929637747	-4.690310884
hsa-miR-6794-3p	MIMAT0027489	-0.101527723	1.092434716	0.969798259	0.347977147	0.930573075	-4.746286526
hsa-mir-3713	MI0016134	-0.101534598	1.054785021	0.881640724	0.392288588	0.930573075	-4.792437287

hsa-miR-3655	MIMAT0018075	-0.101963574	0.971590234	0.702609396	0.493376616	0.960979619	-4.874338106
hsa-mir-302f	MI0006418	-0.101990555	0.940923119	1.146342976	0.270138043	0.927057986	-4.643239841
hsa-miR-6760-3p	MIMAT0027421	-0.102532373	0.918480403	1.245089501	0.232759439	0.919664114	-4.57989544
hsa-mir-6515	MI0022227	-0.102619498	0.880026516	1.325353631	0.205468883	0.908883572	-4.525644975
hsa-mir-3910-1	MI0016414	-0.102637414	0.993855133	1.390336047	0.185308527	0.903156384	-4.480026418
hsa-mir-4660	MI0017288	-0.102641584	1.062674637	1.235668737	0.236141466	0.919664114	-4.586104323
hsa-miR-4681	MIMAT0019766	-0.102852146	0.939900582	1.293690951	0.215912449	0.917147059	-4.54733007
hsa-miR-4789-5p	MIMAT0019959	-0.103149812	0.789649898	1.375972868	0.189620008	0.903156384	-4.490235392
hsa-mir-5572	MI0019117	-0.103328599	0.976258894	1.430234417	0.173751454	0.903114784	-4.451304764
hsa-mir-6784	MI0022629	-0.103359621	1.099397502	1.810524397	0.090888738	0.896940866	-4.154340299
hsa-mir-6754	MI0022599	-0.103475187	1.107119117	0.865674777	0.400703456	0.930573075	-4.800396189
hsa-miR-5002-5p	MIMAT0021023	-0.103483104	1.001769052	1.043682645	0.313646215	0.930208704	-4.704828169
hsa-mir-5091	MI0017980	-0.103582104	1.080747504	0.884135948	0.390984252	0.930573075	-4.791182199
hsa-miR-2115-3p	MIMAT0011159	-0.103711867	1.000120595	1.982789282	0.066578321	0.896940866	-4.008854787
hsa-miR-891a-5p	MIMAT0004902	-0.103721852	0.924723283	1.082621373	0.296574824	0.927057986	-4.68199978
hsa-mir-6507	MI0022219	-0.103859556	1.041786022	1.159504364	0.26490672	0.926762219	-4.635022991
hsa-miR-194-3p	MIMAT0004671	-0.10393066	0.996291599	1.182339846	0.256013875	0.921727531	-4.620598969
hsa-miR-26b-3p	MIMAT0004500	-0.104020669	0.902728346	1.277648408	0.221362986	0.917147059	-4.558177144
hsa-mir-30e	MI0000749	-0.104205708	1.124547994	0.94802326	0.358582786	0.930573075	-4.758027726
hsa-mir-135b	MI0000810	-0.104961787	0.872262918	1.640448592	0.122323637	0.896940866	-4.291883292
hsa-miR-891b	MIMAT0004913	-0.105254059	0.828623942	1.140210416	0.272602176	0.927057986	-4.647044132
hsa-mir-4524a	MI0016891	-0.105302242	0.870432503	1.421272247	0.176294372	0.903156384	-4.457802193
hsa-mir-7157	MI0023617	-0.105377305	0.979108225	1.1461638	0.270209797	0.927057986	-4.643351212
hsa-mir-4648	MI0017275	-0.105880459	1.129152874	1.187193939	0.254153399	0.921246697	-4.61750577
hsa-mir-4536-2	MI0019149	-0.106074829	0.950282501	1.506587623	0.153295408	0.902932883	-4.394912377
hsa-miR-3174	MIMAT0015051	-0.106177325	1.079204557	1.11325595	0.283634438	0.927057986	-4.663579523
hsa-mir-5186	MI0018165	-0.106375369	1.007065931	1.158955177	0.265123456	0.926762219	-4.635367276
hsa-mir-6734	MI0022579	-0.106610056	0.967984537	1.26977045	0.224079191	0.919200047	-4.563468741
hsa-miR-3140-3p	MIMAT0015008	-0.10670783	0.785577723	1.171244436	0.260305767	0.923131598	-4.62763376
hsa-mir-6791	MI0022636	-0.10711001	1.009940003	1.302270043	0.213041783	0.91645315	-4.541490442
hsa-miR-4317	MIMAT0016872	-0.107119621	1.183160525	0.580999631	0.570122642	0.967644566	-4.920363546
hsa-miR-4746-3p	MIMAT0019881	-0.107337295	0.942055564	1.080701252	0.297400236	0.927057986	-4.683140941
hsa-mir-1282	MI0006429	-0.107631131	1.012514335	1.240102118	0.234545136	0.919664114	-4.583186689
hsa-miR-3675-3p	MIMAT0018099	-0.107648614	1.126225545	1.00536263	0.331133475	0.930208704	-4.726639122
hsa-miR-603	MIMAT0003271	-0.107769095	0.957266175	1.432914597	0.172996889	0.903074508	-4.449356587
hsa-miR-4285	MIMAT0016913	-0.10780964	0.760558222	1.547251083	0.143252161	0.896940866	-4.36415261
hsa-miR-6508-3p	MIMAT0025473	-0.107971423	0.823477684	1.15545297	0.26650877	0.927057986	-4.637559906
hsa-miR-6865-3p	MIMAT0027631	-0.107995466	1.291180109	0.697741442	0.496327457	0.960979619	-4.876333737
hsa-mir-1910	MI0008331	-0.108053809	0.900089472	0.951642721	0.35680448	0.930573075	-4.756091429
hsa-miR-1251-5p	MIMAT0005903	-0.108136285	0.875070839	1.755383764	0.10019556	0.896940866	-4.1996677
hsa-miR-511-3p	MIMAT0026606	-0.10824446	1.101579345	1.049914796	0.31086678	0.930208704	-4.701219124
hsa-miR-589-3p	MIMAT0003256	-0.108319277	1.080805094	0.873188164	0.396728672	0.930573075	-4.796666348
hsa-mir-4685	MI0017317	-0.108404417	1.035823895	1.113858534	0.2833842	0.927057986	-4.663213195
hsa-miR-96-3p	MIMAT0004510	-0.108730187	0.8635501	1.235041431	0.236368024	0.919664114	-4.586516545
hsa-mir-4486	MI0016847	-0.108752903	0.866822032	1.268686077	0.224455121	0.919659426	-4.564195291
hsa-mir-6775	MI0022620	-0.10899512	1.016273629	1.460315295	0.165437019	0.903074508	-4.429306734
hsa-miR-6748-3p	MIMAT0027397	-0.10915588	0.873017419	1.563057168	0.139501582	0.896940866	-4.352065924
hsa-miR-6512-5p	MIMAT0025480	-0.109164861	0.857394264	1.210631069	0.245316569	0.919664114	-4.602438748
hsa-miR-4490	MIMAT0019025	-0.109269589	0.835097867	1.248055248	0.231702639	0.919664114	-4.577933783
hsa-miR-196a-3p	MIMAT0004562	-0.109586152	0.930406028	1.581922526	0.135134726	0.896940866	-4.337546974
hsa-miR-1226-5p	MIMAT0005576	-0.109622383	1.780004185	0.445434972	0.662554608	0.984005175	-4.961976535
hsa-mir-4254	MI0015862	-0.109692604	0.950178251	1.604377049	0.130089712	0.896940866	-4.320136746
hsa-mir-3123	MI0014139	-0.109782415	1.115270519	1.110317778	0.284856955	0.927057986	-4.665363527
hsa-miR-6759-5p	MIMAT0027418	-0.109887751	0.975111634	1.608852584	0.129103689	0.896940866	-4.316650139
hsa-miR-885-3p	MIMAT0004948	-0.110436808	3.05171151	0.253577002	0.803366859	0.994214304	-5.002616929
hsa-miR-499b-3p	MIMAT0019898	-0.110738419	0.944781744	0.983842292	0.341254942	0.930208704	-4.738597245
hsa-mir-503	MI0003188	-0.110741038	1.012114954	1.019719607	0.324501512	0.930208704	-4.718544541
hsa-mir-1302-7	MI0006368	-0.110768885	0.971496056	1.34034277	0.200668295	0.905221726	-4.515253452
hsa-miR-4684-3p	MIMAT0019770	-0.110890499	1.022319493	1.211038367	0.24516513	0.919664114	-4.602174988
hsa-miR-190a-5p	MIMAT0000458	-0.111060562	0.842179436	1.70896651	0.108670697	0.896940866	-4.237294206
hsa-miR-424-3p	MIMAT0004749	-0.111299136	1.398730498	0.817488444	0.426820412	0.941917225	-4.823653668
hsa-miR-381-5p	MIMAT0022862	-0.111375187	0.858134894	1.076758641	0.299100382	0.927057986	-4.685479112
hsa-miR-6870-3p	MIMAT0027641	-0.111505029	0.972733118	0.990843975	0.337938108	0.930208704	-4.734729853
hsa-mir-5581	MI0019136	-0.111905809	0.995869119	1.36136841	0.194087322	0.903764656	-4.500543433
hsa-miR-3138	MIMAT0015006	-0.112343888	1.550019783	0.630777588	0.53795866	0.961617307	-4.902496919
hsa-mir-3938	MI0016594	-0.112357017	0.974664683	1.118688069	0.281384589	0.927057986	-4.660271648

hsa-miR-4512	MIMAT0019049	-0.112378913	0.850151214	1.051660773	0.310091333	0.930208704	-4.700204956
hsa-mir-3190	MI0014235	-0.112514347	0.961243317	1.327579116	0.204750343	0.907676232	-4.524107173
hsa-miR-345-5p	MIMAT0000772	-0.112588329	2.136392494	0.453034568	0.657199467	0.984005175	-4.959921519
hsa-miR-6895-5p	MIMAT0027690	-0.112705935	1.100589421	1.147245764	0.269776722	0.927057986	-4.642678491
hsa-miR-6742-5p	MIMAT0027385	-0.112823273	1.366842711	0.863602578	0.401804345	0.930573075	-4.801420012
hsa-miR-4278	MIMAT0016910	-0.11327527	0.939569777	1.476298478	0.161155472	0.903074508	-4.417501362
hsa-miR-378g	MIMAT0018937	-0.11330863	1.127401385	1.021613224	0.323633969	0.930208704	-4.717469975
hsa-miR-4668-5p	MIMAT0019745	-0.113404745	1.267720931	0.764165986	0.456970378	0.951465791	-4.848027429
hsa-miR-7855-5p	MIMAT0030430	-0.113436048	0.992914	1.271101477	0.223618431	0.918865246	-4.562576325
hsa-miR-6781-5p	MIMAT0027462	-0.113602775	1.556565809	0.482549248	0.636587744	0.982269833	-4.951624562
hsa-miR-6846-3p	MIMAT0027593	-0.113721595	0.949982078	1.434896357	0.172440697	0.903074508	-4.447914579
hsa-miR-3937	MIMAT0018352	-0.11438626	5.868071863	0.189615548	0.852227558	0.996778901	-5.011275269
hsa-miR-214-5p	MIMAT0004564	-0.114424822	0.885243367	1.38331306	0.187406551	0.903156384	-4.485026941
hsa-miR-138-1-3p	MIMAT0004607	-0.114435377	0.830913589	1.792943291	0.093769346	0.896940866	-4.168864027
hsa-miR-3184-5p	MIMAT0015064	-0.115025331	0.892168631	1.365901495	0.192691655	0.903764656	-4.497351789
hsa-miR-645	MIMAT0003315	-0.115160678	0.838743064	1.952053818	0.070429814	0.896940866	-4.035201721
hsa-miR-3142	MIMAT0015011	-0.115203334	0.823935072	1.255158262	0.229186899	0.919664114	-4.573221946
hsa-mir-4677	MI0017308	-0.11526137	0.917787793	1.382660138	0.187602584	0.903156384	-4.485490986
hsa-mir-6499	MI0022209	-0.115758732	0.852533151	1.407796982	0.18017546	0.903156384	-4.46752184
hsa-miR-6072	MIMAT0023697	-0.116080844	0.967897183	1.421503737	0.176228306	0.903156384	-4.457634697
hsa-mir-548k	MI0006354	-0.116108743	0.82226306	1.515483265	0.151049065	0.902932883	-4.388225213
hsa-miR-4751	MIMAT0019888	-0.116427067	0.934107054	1.065585573	0.303957391	0.930208704	-4.692068706
hsa-miR-5580-3p	MIMAT0022274	-0.116797996	0.986851344	1.28504947	0.21883503	0.917147059	-4.5531848
hsa-miR-4468	MIMAT0018995	-0.11731377	1.025283982	1.197695796	0.250163867	0.919664114	-4.610781394
hsa-miR-770-5p	MIMAT0003948	-0.117640573	0.899599968	1.194918074	0.251214361	0.92023334	-4.612564252
hsa-mir-6765	MI0022610	-0.117746628	1.780198324	0.396589346	0.697420215	0.985651988	-4.974383923
hsa-mir-4536-1	MI0016906	-0.117750332	0.94032752	1.139109231	0.27304644	0.927057986	-4.647725597
hsa-miR-144-5p	MIMAT0004600	-0.117757783	0.882848565	1.750164543	0.101118599	0.896940866	-4.203923298
hsa-miR-409-5p	MIMAT0001638	-0.11793352	1.353098276	0.645635646	0.528556437	0.960979619	-4.896900807
hsa-miR-4256	MIMAT0016877	-0.118300998	0.923646296	1.354874216	0.19610106	0.905071197	-4.505103422
hsa-mir-3910-2	MI0016431	-0.118379901	0.889145327	1.251066558	0.23063346	0.919664114	-4.575938554
hsa-mir-7704	MI0025240	-0.118398563	0.899957676	1.399317979	0.182653317	0.903156384	-4.473606807
hsa-miR-3145-3p	MIMAT0015016	-0.118622754	0.906609231	1.514284495	0.151350156	0.902932883	-4.389127755
hsa-mir-203b	MI0017343	-0.118681214	0.941326346	1.365737692	0.192741945	0.903764656	-4.497467243
hsa-miR-654-5p	MIMAT0003330	-0.119052684	1.149663491	0.681510689	0.506241009	0.960979619	-4.882896455
hsa-mir-4516	MI0016882	-0.119202311	0.984423826	1.294364224	0.215686056	0.917147059	-4.546872762
hsa-miR-23c	MIMAT0018000	-0.119453607	1.419840656	0.890310018	0.387769394	0.930573075	-4.788063662
hsa-mir-6799	MI0022644	-0.11948713	1.156356209	1.027593362	0.3209052	0.930208704	-4.714065888
hsa-miR-3146	MIMAT0015018	-0.11950403	0.862082706	1.601392937	0.130750736	0.896940866	-4.322458457
hsa-miR-550a-3-5p	MIMAT0020925	-0.119776684	1.906198703	0.779581777	0.448120194	0.950247229	-4.841130038
hsa-miR-6511b-3p	MIMAT0025848	-0.120354904	1.507704537	0.820277129	0.425279574	0.940744089	-4.822339268
hsa-miR-4749-5p	MIMAT0019885	-0.120659763	1.91270462	0.385269885	0.705605538	0.986573137	-4.977060233
hsa-mir-668	MI0003761	-0.120920405	0.887120626	1.422404529	0.175971416	0.903156384	-4.456982757
hsa-miR-3128	MIMAT0014991	-0.121165377	0.893197282	1.236266733	0.23592565	0.919664114	-4.58571122
hsa-miR-623	MIMAT0003292	-0.121425962	1.165296042	1.520897067	0.149695575	0.902932883	-4.384143873
hsa-mir-3648-1	MI0016048	-0.121515922	1.670829427	0.50128837	0.623659384	0.978652564	-4.946097369
hsa-miR-4726-5p	MIMAT0019845	-0.121516115	1.249650505	0.892729461	0.386514453	0.930573075	-4.786836556
hsa-miR-6838-3p	MIMAT0027579	-0.121544472	0.917097295	1.367690053	0.192143233	0.903764656	-4.49609055
hsa-miR-188-3p	MIMAT0004613	-0.121861767	1.013724195	1.36744359	0.19221873	0.903764656	-4.496264414
hsa-mir-452	MI0001733	-0.121866435	0.988087936	1.131079435	0.276302552	0.927057986	-4.652679588
hsa-miR-4439	MIMAT0018957	-0.122375558	0.905948861	1.293392447	0.216012884	0.917147059	-4.54753277
hsa-mir-1973	MI0009983	-0.122604587	1.183933964	0.932965475	0.366046989	0.930573075	-4.766017057
hsa-miR-501-5p	MIMAT0002872	-0.123029505	1.014917469	1.168857826	0.261236102	0.924045176	-4.629140434
hsa-miR-624-3p	MIMAT0004807	-0.1230458	0.779345408	1.458098509	0.166038255	0.903074508	-4.430937729
hsa-miR-642a-3p	MIMAT0020924	-0.123077069	1.784728101	0.330054594	0.746058076	0.990306303	-4.989032164
hsa-miR-4762-5p	MIMAT0019910	-0.123160499	0.917526224	1.689311519	0.112444382	0.896940866	-4.253072953
hsa-miR-4776-3p	MIMAT0019933	-0.123248178	0.889584649	1.747853007	0.101529788	0.896940866	-4.20580608
hsa-miR-6795-5p	MIMAT0027490	-0.12334441	1.310414394	0.708529204	0.489802199	0.95960537	-4.871894333
hsa-mir-1290	MI0006352	-0.123413414	0.843036369	1.853491381	0.084177273	0.896940866	-4.118576264
hsa-miR-619-5p	MIMAT0026622	-0.123739044	2.241865811	0.122477353	0.904194924	0.999227185	-5.017690657
hsa-mir-8081	MI0025917	-0.123990252	0.822147175	1.450233554	0.168186053	0.903074508	-4.436711767
hsa-mir-4525	MI0016892	-0.124001025	0.973990622	0.711823577	0.487819725	0.959005558	-4.870526343
hsa-miR-7110-3p	MIMAT0028118	-0.124260419	0.999606803	1.154490377	0.26689049	0.927057986	-4.638161678
hsa-miR-4769-5p	MIMAT0019922	-0.124363233	1.167521488	0.956939935	0.354212954	0.930573075	-4.753246536
hsa-miR-137-3p	MIMAT0000429	-0.124817255	0.851583317	1.594093556	0.132379797	0.896940866	-4.32812731
hsa-mir-3124	MI0014140	-0.125172148	0.897975821	1.956702731	0.069834572	0.896940866	-4.031226616

hsa-mir-548av	MI0019152	-0.125368068	0.878747952	1.951810495	0.070461095	0.896940866	-4.035409677
hsa-mir-491	MI0003126	-0.125858809	1.171173933	0.965219487	0.350188784	0.930573075	-4.748773753
hsa-miR-5584-5p	MIMAT0022283	-0.125914145	1.014777033	1.904815015	0.07674374	0.896940866	-4.07538234
hsa-miR-605-3p	MIMAT0026621	-0.125939571	0.898742423	1.644912619	0.121390926	0.896940866	-4.288362929
hsa-miR-3683	MIMAT0018111	-0.126052742	0.885910257	1.563538899	0.139388597	0.896940866	-4.35169643
hsa-miR-6873-5p	MIMAT0027646	-0.126451183	0.974885176	1.388556308	0.185838376	0.903156384	-4.481295212
hsa-miR-4666b	MIMAT0022485	-0.126564667	1.087861969	0.787114545	0.443835043	0.947942305	-4.837715354
hsa-mir-603	MI0003616	-0.126573482	0.94116041	1.146515799	0.270068846	0.927057986	-4.643132407
hsa-miR-5007-5p	MIMAT0021035	-0.126674184	0.855332568	1.482425274	0.159538934	0.903074508	-4.412954887
hsa-miR-99a-3p	MIMAT0004511	-0.126854996	0.777806063	1.285201724	0.218783266	0.917147059	-4.553081885
hsa-miR-5703	MIMAT0022496	-0.127272847	1.71086431	0.887825942	0.389060714	0.930573075	-4.789320599
hsa-miR-505-3p	MIMAT0002876	-0.127278351	0.95934601	0.963134723	0.351199026	0.930573075	-4.74990299
hsa-miR-26a-5p	MIMAT0000082	-0.127331449	12.84245466	0.947215022	0.358980728	0.930573075	-4.758459269
hsa-miR-1324	MIMAT0005956	-0.127341509	0.970744768	1.292552238	0.216295781	0.917147059	-4.54810314
hsa-mir-640	MI0003655	-0.127351956	0.877874397	1.514501396	0.15129564	0.902932883	-4.388964484
hsa-miR-20a-3p	MIMAT0004493	-0.127579829	0.919793041	1.28633996	0.218396596	0.917147059	-4.552312226
hsa-miR-5706	MIMAT0022500	-0.128283302	0.875573852	1.027420696	0.320983754	0.930208704	-4.714164399
hsa-miR-6759-3p	MIMAT0027419	-0.128463257	0.93660689	1.430917918	0.173558766	0.903090978	-4.45080816
hsa-miR-493-3p	MIMAT0003161	-0.128492705	1.354271393	0.522900944	0.608906444	0.975135504	-4.939474159
hsa-mir-1277	MI0006419	-0.128512125	0.887031221	1.898596156	0.077611905	0.896940866	-4.080642415
hsa-mir-3147	MI0014173	-0.12854559	1.02723069	1.213310833	0.244321529	0.919664114	-4.600702179
hsa-miR-181a-5p	MIMAT0000256	-0.128612031	8.000914668	0.495928247	0.627344585	0.978652564	-4.94769885
hsa-mir-6128	MI0021272	-0.12865566	0.872100684	1.871428029	0.081509077	0.896940866	-4.103537705
hsa-miR-518c-3p	MIMAT0002848	-0.1288262	0.80867344	1.826628748	0.088319357	0.896940866	-4.140979697
hsa-miR-154-3p	MIMAT0000453	-0.128859384	0.966933666	1.856804868	0.083678563	0.896940866	-4.115802866
hsa-miR-548k	MIMAT0005882	-0.129322406	1.048542945	1.195430063	0.251020479	0.920172177	-4.612235867
hsa-miR-5583-3p	MIMAT0022282	-0.1293306	0.890100992	1.752216766	0.100754766	0.896940866	-4.202250712
hsa-mir-4760	MI0017401	-0.129599513	0.872529628	2.239018158	0.041228098	0.896940866	-3.784079798
hsa-miR-6514-5p	MIMAT0025484	-0.129620583	1.020650474	1.170439845	0.260619124	0.923493139	-4.628141959
hsa-mir-6736	MI0022581	-0.130103523	1.017010764	1.123338725	0.279469062	0.927057986	-4.657429781
hsa-mir-3132	MI0014152	-0.130269196	0.815333845	2.543746257	0.022859996	0.896940866	-3.509111937
hsa-mir-3121	MI0014137	-0.130533544	0.967767502	1.505975519	0.153451005	0.903074508	-4.395371641
hsa-mir-4278	MI0015888	-0.130562327	0.970140888	1.819073432	0.089516617	0.896940866	-4.147254473
hsa-miR-17-3p	MIMAT0000071	-0.130598127	1.510493049	0.654302738	0.523114862	0.960979619	-4.893581087
hsa-mir-34c	MI0000743	-0.130642931	0.964345879	1.894494967	0.078189264	0.896940866	-4.084107425
hsa-miR-340-3p	MIMAT0000750	-0.130661076	0.816490406	1.735897853	0.103679993	0.896940866	-4.215524219
hsa-miR-6787-3p	MIMAT0027475	-0.130764761	0.889960485	1.89887317	0.077573046	0.896940866	-4.08040826
hsa-mir-4656	MI0017284	-0.131307856	0.981099198	1.595133794	0.132146582	0.896940866	-4.327320331
hsa-mir-8065	MI0025901	-0.131964363	0.937038984	1.847718222	0.085052538	0.896940866	-4.123403249
hsa-miR-4442	MIMAT0018960	-0.132281232	0.999498802	1.025729701	0.321753811	0.930208704	-4.71512846
hsa-mir-548ah	MI0016796	-0.132650523	0.925924004	1.320206626	0.20713845	0.911075497	-4.529194739
hsa-mir-4430	MI0016769	-0.132762375	1.015243986	1.228010261	0.238919047	0.919664114	-4.591126544
hsa-miR-6758-5p	MIMAT0027416	-0.132781364	1.162132644	1.510973022	0.152184513	0.902932883	-4.391618691
hsa-miR-4800-5p	MIMAT0019978	-0.132828974	1.063780678	0.811888554	0.429925383	0.94301493	-4.82628126
hsa-mir-4724	MI0017361	-0.133291937	0.947357256	1.418217833	0.177168005	0.903156384	-4.460010581
hsa-mir-345	MI0000825	-0.133486817	1.142192945	1.142210565	0.271796631	0.927057986	-4.645805062
hsa-miR-548e-3p	MIMAT0005874	-0.133636751	1.073011074	1.54668946	0.143386986	0.896940866	-4.364580753
hsa-mir-1298	MI0003938	-0.133645263	0.903198006	1.599069445	0.131267415	0.896940866	-4.324264511
hsa-miR-642b-3p	MIMAT0018444	-0.134078191	1.913507032	0.361389934	0.722996623	0.988677831	-4.982459075
hsa-miR-6827-3p	MIMAT0027555	-0.134113423	0.760779008	1.193466668	0.251764614	0.920370901	-4.613494606
hsa-mir-802	MI0003906	-0.134194176	0.909418475	1.782699647	0.095484784	0.896940866	-4.177295817
hsa-mir-7162	MI0023623	-0.134280879	0.949889757	1.195476413	0.251002933	0.920172177	-4.612206134
hsa-mir-125a	MI0000469	-0.134555928	0.993692313	1.223085947	0.240718417	0.919664114	-4.594343768
hsa-mir-371b	MI0017393	-0.134716357	1.077616372	1.481533108	0.159773483	0.903074508	-4.413617655
hsa-mir-765	MI0005116	-0.135413522	1.006037808	1.415554629	0.177932649	0.903156384	-4.46193361
hsa-mir-325	MI0000824	-0.135826058	0.852062211	1.393335057	0.18441848	0.903156384	-4.477885971
hsa-mir-6790	MI0022635	-0.135831569	1.51321163	1.253906333	0.22962874	0.919664114	-4.574053816
hsa-miR-548e-5p	MIMAT0026736	-0.135860088	0.876761697	1.936608106	0.072440612	0.896940866	-4.048382441
hsa-mir-186	MI0000483	-0.135919617	0.925459984	2.383590608	0.031237227	0.896940866	-3.654291461
hsa-mir-3617	MI0016007	-0.136089083	0.981697129	1.739929836	0.102950388	0.896940866	-4.212250362
hsa-miR-1973	MIMAT0009448	-0.136092757	4.444734897	0.201049576	0.843440453	0.996080495	-5.009909322
hsa-miR-19a-5p	MIMAT0004490	-0.136243714	0.835077091	1.880448352	0.080196094	0.896940866	-4.095951436
hsa-mir-3909	MI0016413	-0.136343923	0.874218841	1.595842834	0.131987822	0.896940866	-4.326770113
hsa-miR-6754-5p	MIMAT0027408	-0.136358266	1.504984065	0.747101305	0.466892833	0.951465791	-4.855519508
hsa-miR-3613-3p	MIMAT0017991	-0.136963813	1.136357827	0.918373464	0.373381906	0.930573075	-4.77365691
hsa-miR-2355-5p	MIMAT0016895	-0.137693674	0.927647323	1.561963301	0.139758427	0.896940866	-4.352904691

hsa-miR-34a-3p	MIMAT0004557	-0.137711367	1.06103168	1.005855243	0.330904326	0.930208704	-4.726362926
hsa-miR-483-3p	MIMAT0002173	-0.138296713	0.898842885	1.93594221	0.072528458	0.896940866	-4.048949768
hsa-miR-3610	MIMAT0017987	-0.138794195	0.982854933	0.910039894	0.377615767	0.930573075	-4.777974548
hsa-mir-623	MI0003637	-0.13896612	1.036945102	2.151404506	0.048664836	0.896940866	-3.861838373
hsa-mir-450a-1	MI0001652	-0.139018933	1.069864846	1.765290812	0.098463859	0.896940866	-4.191572909
hsa-miR-548ap-3p	MIMAT0021038	-0.13903881	0.965222265	1.210845588	0.245236799	0.919664114	-4.602299837
hsa-miR-6509-3p	MIMAT0025475	-0.139165054	1.048748816	1.562557122	0.139618945	0.896940866	-4.352449397
hsa-miR-33b-5p	MIMAT0003301	-0.139199939	0.890451005	1.480604984	0.160017792	0.903074508	-4.414306874
hsa-mir-1302-1	MI0006362	-0.139475538	1.098311771	1.536921895	0.14574906	0.897988521	-4.37201223
hsa-miR-378f	MIMAT0018932	-0.139679167	1.592875446	0.612507225	0.549646349	0.965790957	-4.90921302
hsa-mir-4800	MI0017448	-0.139875514	1.149727042	1.386630624	0.186413069	0.903156384	-4.482666845
hsa-miR-4735-5p	MIMAT0019860	-0.139917875	0.746964701	2.090950688	0.054502153	0.896940866	-3.914989098
hsa-mir-581	MI0003588	-0.140184224	1.140185881	1.75324307	0.100573248	0.896940866	-4.201413904
hsa-mir-378d-1	MI0016749	-0.140554333	0.906889808	1.922933379	0.074264026	0.896940866	-4.060017488
hsa-mir-1278	MI0006425	-0.14072363	0.992148633	1.782439226	0.095528755	0.896940866	-4.17750988
hsa-mir-507	MI0003194	-0.140774284	0.969964345	1.818006508	0.089686846	0.896940866	-4.148139617
hsa-mir-4447	MI0016790	-0.140804077	0.87705949	2.305608132	0.036301601	0.896940866	-3.724495654
hsa-mir-143	MI0000459	-0.141593205	0.977215392	1.610866907	0.128662002	0.896940866	-4.31507914
hsa-mir-23a	MI0000079	-0.141686579	1.057765078	1.450751149	0.168044001	0.903074508	-4.436332381
hsa-miR-1302	MIMAT0005890	-0.142224252	1.00009179	1.972067241	0.067899627	0.896940866	-4.018063465
hsa-miR-224-3p	MIMAT0009198	-0.142559501	1.299891878	0.706679323	0.490917511	0.959629992	-4.872659981
hsa-miR-5687	MIMAT0022478	-0.143457563	1.027628719	1.298257163	0.214380729	0.917147059	-4.544225291
hsa-mir-195	MI0000489	-0.143516764	1.045569432	1.310761948	0.210230363	0.91621875	-4.535683692
hsa-miR-519c-5p	MIMAT0002831	-0.143518573	0.786868685	2.08110267	0.055512089	0.896940866	-3.923603796
hsa-miR-523-5p	MIMAT0005449	-0.143518573	0.786868685	2.08110267	0.055512089	0.896940866	-3.923603796
hsa-miR-518e-5p	MIMAT0005450	-0.143518573	0.786868685	2.08110267	0.055512089	0.896940866	-3.923603796
hsa-miR-522-5p	MIMAT0005451	-0.143518573	0.786868685	2.08110267	0.055512089	0.896940866	-3.923603796
hsa-miR-519a-5p	MIMAT0005452	-0.143518573	0.786868685	2.08110267	0.055512089	0.896940866	-3.923603796
hsa-miR-519b-5p	MIMAT0005454	-0.143518573	0.786868685	2.08110267	0.055512089	0.896940866	-3.923603796
hsa-miR-188-5p	MIMAT0000457	-0.143689686	1.143962265	1.144884733	0.270722447	0.927057986	-4.64414586
hsa-miR-410-3p	MIMAT0002171	-0.143756838	1.027234038	1.919654732	0.07470732	0.896940866	-4.062802216
hsa-mir-4740	MI0017378	-0.144309965	1.323729112	1.234486963	0.236568418	0.919664114	-4.586880777
hsa-miR-2116-3p	MIMAT0011161	-0.144417317	1.04937014	1.979933183	0.066927983	0.896940866	-4.011309573
hsa-mir-4270	MI0015878	-0.144460535	1.070726663	1.268353129	0.224570647	0.919659426	-4.564418284
hsa-mir-5739	MI0019412	-0.144463887	0.940384993	1.620298893	0.126611049	0.896940866	-4.307708487
hsa-mir-4302	MI0015833	-0.144645887	0.900152539	1.91721518	0.075038712	0.896940866	-4.064873019
hsa-miR-381-3p	MIMAT0000736	-0.144671338	1.079108213	0.783123737	0.446102057	0.949212502	-4.839528045
hsa-miR-6829-5p	MIMAT0027558	-0.144818091	1.192020147	1.134927405	0.27473855	0.927057986	-4.650308924
hsa-miR-448	MIMAT0001532	-0.144878471	0.844745121	1.418732942	0.177020423	0.903156384	-4.459638364
hsa-miR-4305	MIMAT0016857	-0.1449129	0.893802246	1.490764234	0.157360523	0.903074508	-4.406748204
hsa-mir-20a	MI0000076	-0.144930521	0.918118233	2.013453707	0.062927598	0.896940866	-3.982418186
hsa-miR-6730-5p	MIMAT0027361	-0.145636723	1.06458892	0.938945423	0.363069957	0.930573075	-4.762857019
hsa-miR-6715a-3p	MIMAT0025841	-0.146449614	1.424304465	0.603478721	0.555472692	0.96620986	-4.912464228
hsa-mir-1256	MI0006390	-0.1465766	0.868474243	2.215369173	0.043123508	0.896940866	-3.805145675
hsa-miR-6806-5p	MIMAT0027512	-0.146938114	1.54374853	0.536968172	0.599397127	0.97307982	-4.93502101
hsa-mir-17	MI0000071	-0.147326525	0.981047955	1.463871732	0.164476226	0.903074508	-4.426686849
hsa-mir-25	MI0000082	-0.147585682	1.019296572	1.580468846	0.135467019	0.896940866	-4.338669291
hsa-mir-16-2	MI0000115	-0.147896456	1.040609381	1.496849903	0.155786513	0.903074508	-4.402205171
hsa-miR-1226-3p	MIMAT0005577	-0.148425286	1.027004878	1.421347071	0.176273016	0.903156384	-4.457748056
hsa-miR-4536-5p	MIMAT0019078	-0.148456618	0.934809272	1.448971351	0.168532879	0.903074508	-4.437636574
hsa-mir-4507	MI0016871	-0.149135812	1.308257537	0.970303568	0.347733675	0.930573075	-4.746011443
hsa-miR-4800-3p	MIMAT0019979	-0.149328411	1.096592802	1.337331228	0.20162548	0.905221726	-4.517347671
hsa-miR-6748-5p	MIMAT0027396	-0.14944706	1.226513822	1.602670358	0.130467417	0.896940866	-4.321464889
hsa-miR-5571-3p	MIMAT0022258	-0.150198843	0.741120264	1.62999964	0.124531053	0.896940866	-4.300103064
hsa-miR-1185-1-3p	MIMAT0022838	-0.150565463	1.260093753	1.442858872	0.170220855	0.903074508	-4.442107931
hsa-miR-4494	MIMAT0019029	-0.150580526	0.917361274	1.939005453	0.072125147	0.896940866	-4.046339323
hsa-mir-6865	MI0022712	-0.150607145	0.967539844	1.830567767	0.087700829	0.896940866	-4.137703657
hsa-miR-4652-5p	MIMAT0019716	-0.150734265	0.902546483	1.723388272	0.105972618	0.896940866	-4.225657599
hsa-miR-18a-3p	MIMAT0002891	-0.15083554	1.040563784	1.656797816	0.118937555	0.896940866	-4.278965076
hsa-miR-4761-5p	MIMAT0019908	-0.151103301	0.884602699	1.33860271	0.201220906	0.905221726	-4.516463878
hsa-mir-4327	MI0015867	-0.151126978	0.983548112	1.389927788	0.185429961	0.903156384	-4.480317565
hsa-miR-4665-5p	MIMAT0019739	-0.151239254	2.535438574	0.463026318	0.650188218	0.982649571	-4.957168867
hsa-miR-6753-3p	MIMAT0027407	-0.15129151	0.988342477	1.71877986	0.106828331	0.896940866	-4.229381391
hsa-miR-92a-1-5p	MIMAT0004507	-0.151386927	1.001133864	1.588380422	0.133666933	0.896940866	-4.332554043
hsa-mir-1255a	MI0006389	-0.151558909	0.929844713	1.78504744	0.095089179	0.896940866	-4.175365297
hsa-miR-4633-5p	MIMAT0019689	-0.151906805	0.870484389	2.223492146	0.042463481	0.896940866	-3.79791599

hsa-miR-1915-5p	MIMAT0007891	-0.151935847	0.919758326	1.402529665	0.181711485	0.903156384	-4.471304762
hsa-mir-4280	MI0015889	-0.152387306	0.842248323	2.11315595	0.052286776	0.896940866	-3.895518666
hsa-miR-6830-3p	MIMAT0027561	-0.152484076	0.80397466	1.606645139	0.129589215	0.896940866	-4.318370498
hsa-miR-29a-3p	MIMAT0000086	-0.152941644	6.481117857	0.605331913	0.554274059	0.96620986	-4.911800544
hsa-miR-3661	MIMAT0018082	-0.153263734	0.818879935	1.790543306	0.094168789	0.896940866	-4.170841543
hsa-miR-4693-3p	MIMAT0019785	-0.153457916	0.963357663	1.55902804	0.140449628	0.896940866	-4.355153735
hsa-mir-4257	MI0015856	-0.153788709	0.963427501	1.759052981	0.099551095	0.896940866	-4.196672246
hsa-miR-3657	MIMAT0018077	-0.153945045	0.81191602	1.851327633	0.08450437	0.896940866	-4.120386163
hsa-mir-381	MI0000789	-0.153954938	0.94137635	1.411003813	0.179245534	0.903156384	-4.465214214
hsa-miR-6079	MIMAT0023704	-0.154225023	0.8788076	1.356305095	0.195655922	0.904897643	-4.504099972
hsa-miR-1231	MIMAT0005586	-0.154367644	2.25576934	0.468666907	0.646245208	0.982649571	-4.955589509
hsa-miR-3123	MIMAT0014985	-0.154501182	0.865034734	1.951930973	0.070445605	0.896940866	-4.035306712
hsa-miR-6764-3p	MIMAT0027429	-0.154945392	0.834156062	1.833405623	0.087257607	0.896940866	-4.135341485
hsa-miR-6747-5p	MIMAT0027394	-0.155082684	1.065244991	1.376790026	0.189372543	0.903156384	-4.489656463
hsa-mir-4637	MI0017264	-0.155225334	0.926539115	2.657393485	0.018270717	0.896940866	-3.405667436
hsa-miR-7854-3p	MIMAT0030429	-0.155859167	1.199927012	1.196608711	0.250574582	0.919922365	-4.611479496
hsa-mir-337	MI0000806	-0.15598871	0.957908135	1.70111129	0.110165396	0.896940866	-4.243611501
hsa-miR-208b-3p	MIMAT0004960	-0.156507639	0.934898983	1.433162627	0.172927197	0.903074508	-4.44917618
hsa-mir-363	MI0000764	-0.156686302	1.081023847	1.75830266	0.099682584	0.896940866	-4.197285035
hsa-miR-6821-3p	MIMAT0027543	-0.15723701	0.962557068	2.625360268	0.019465641	0.896940866	-3.434839196
hsa-miR-6717-5p	MIMAT0025846	-0.157328009	1.168479595	1.542984591	0.144279082	0.896940866	-4.367402801
hsa-miR-4255	MIMAT0016885	-0.15740846	0.860601472	1.9117089	0.075791568	0.896940866	-4.069543089
hsa-miR-378b	MIMAT0014999	-0.157417381	1.059812647	1.804620089	0.091847257	0.896940866	-4.159225136
hsa-miR-4286	MIMAT0016916	-0.157649191	1.284506655	1.034631811	0.317714856	0.930208704	-4.710038904
hsa-miR-6887-3p	MIMAT0027675	-0.158455741	0.860659192	2.44593731	0.027677337	0.896940866	-3.597902842
hsa-mir-4698	MI0017331	-0.158499112	1.150381739	1.246924804	0.232105011	0.919664114	-4.578681898
hsa-miR-32-5p	MIMAT0000090	-0.158842173	0.82446367	1.681836282	0.113909293	0.896940866	-4.259049185
hsa-miR-3692-5p	MIMAT0018121	-0.159064927	0.9321993	1.793698754	0.093643921	0.896940866	-4.168241293
hsa-miR-4714-5p	MIMAT0019822	-0.159096047	1.017148002	1.884042229	0.079678284	0.896940866	-4.09292462
hsa-miR-607	MIMAT0003275	-0.159270587	0.81955842	1.439716502	0.171094064	0.903074508	-4.444401942
hsa-miR-3677-3p	MIMAT0018101	-0.15975259	0.834867884	1.192739501	0.252040644	0.920370901	-4.613960405
hsa-mir-649	MI0003664	-0.159973372	0.858647859	2.381172021	0.031383718	0.896940866	-3.656474701
hsa-mir-362	MI0000762	-0.160685902	1.174926856	1.236536668	0.235828282	0.919664114	-4.585533728
hsa-miR-4679	MIMAT0019763	-0.160851337	0.82987667	2.294683149	0.037070016	0.896940866	-3.734296362
hsa-miR-202-3p	MIMAT0002811	-0.160852862	1.072826517	1.597531139	0.131610452	0.896940866	-4.325459427
hsa-miR-5193	MIMAT0021124	-0.162174454	0.906723024	1.553534502	0.141751064	0.896940866	-4.359356354
hsa-mir-4284	MI0015893	-0.162424649	1.469846905	0.802751404	0.435022704	0.944553236	-4.830534624
hsa-miR-6831-3p	MIMAT0027563	-0.162850279	0.978285361	1.839193303	0.08635986	0.896940866	-4.130518892
hsa-miR-8073	MIMAT0031000	-0.162892716	1.369330138	0.933530909	0.365764777	0.930573075	-4.765718983
hsa-miR-146a-5p	MIMAT0000449	-0.163318113	9.825717647	1.047489546	0.311946259	0.930208704	-4.702625627
hsa-miR-4310	MIMAT0016862	-0.163528712	1.072267063	1.820917776	0.089223027	0.896940866	-4.145723808
hsa-miR-30d-5p	MIMAT0000245	-0.164068504	7.192365238	1.171400428	0.260245047	0.923131598	-4.627535201
hsa-miR-6754-3p	MIMAT0027409	-0.164093802	0.882308356	1.801664253	0.092330477	0.896940866	-4.16166784
hsa-miR-3942-3p	MIMAT0019230	-0.164244356	0.945010098	1.862215298	0.082869922	0.896940866	-4.111269705
hsa-miR-1185-2-3p	MIMAT0022713	-0.164291446	1.219616059	1.391219919	0.185045847	0.903156384	-4.479395898
hsa-mir-3945	MI0016602	-0.164391644	1.020942896	1.470182748	0.162782702	0.903074508	-4.422027973
hsa-miR-3671	MIMAT0018094	-0.164445906	0.96129525	2.040502464	0.059860026	0.896940866	-3.958980276
hsa-miR-27b-5p	MIMAT0004588	-0.165031193	1.605428003	0.801265093	0.435855504	0.944812484	-4.831222509
hsa-miR-3606-5p	MIMAT0017983	-0.165280347	0.907978026	2.07477057	0.056170526	0.896940866	-3.92913609
hsa-mir-92b	MI0003560	-0.165332093	1.180183763	1.046186341	0.312527443	0.930208704	-4.703380338
hsa-miR-1909-5p	MIMAT0007882	-0.165585562	1.101347078	0.562857282	0.582093214	0.968332399	-4.926534279
hsa-mir-656	MI0003678	-0.165721238	1.139099149	1.374619018	0.190030582	0.903156384	-4.491194045
hsa-miR-5008-5p	MIMAT0021039	-0.16593634	0.992794824	1.519017776	0.150164249	0.902932883	-4.385561617
hsa-miR-15a-5p	MIMAT0000068	-0.167387537	6.660986633	0.744603044	0.468356498	0.951465791	-4.856603662
hsa-mir-543	MI0005565	-0.16741674	1.002362109	1.388560379	0.185837163	0.903156384	-4.481292311
hsa-miR-3065-5p	MIMAT0015066	-0.167538537	1.025118026	2.07057912	0.056610312	0.896940866	-3.93279515
hsa-miR-181c-3p	MIMAT0004559	-0.168490862	1.117853045	2.154266614	0.048403377	0.896940866	-3.859311178
hsa-miR-4736	MIMAT0019862	-0.168683617	0.902117855	1.677238572	0.114818522	0.896940866	-4.262718047
hsa-mir-500b	MI0015903	-0.168745822	1.109019545	2.200491636	0.044357256	0.896940866	-3.818370183
hsa-mir-6165	MI0021472	-0.16874846	1.065491848	1.80145025	0.09236555	0.896940866	-4.161844621
hsa-miR-3158-3p	MIMAT0015032	-0.168757167	0.78063236	2.237708408	0.041331001	0.896940866	-3.785247872
hsa-miR-637	MIMAT0003307	-0.168943544	1.325436315	1.065948857	0.303798563	0.930208704	-4.691855304
hsa-miR-589-5p	MIMAT0004799	-0.16942114	1.040388887	1.76125381	0.099166294	0.896940866	-4.194874094
hsa-miR-550b-2-5p	MIMAT0022737	-0.169474736	1.544823508	0.676939269	0.509053847	0.960979619	-4.88471946
hsa-miR-487a-5p	MIMAT0026559	-0.169579173	1.009145008	1.307122021	0.211431783	0.91645315	-4.53817588
hsa-mir-6858	MI0022704	-0.16986964	1.17770073	1.000214078	0.333535224	0.930208704	-4.729519204

hsa-miR-26a-1-3p	MIMAT0004499	-0.170347196	0.857613477	1.540471955	0.144886773	0.896940866	-4.369314442
hsa-miR-7154-3p	MIMAT0028219	-0.170808356	0.928517141	1.910469858	0.075961914	0.896940866	-4.070593215
hsa-miR-5089-3p	MIMAT0022984	-0.170865446	1.015340654	1.551775733	0.14216988	0.896940866	-4.360700002
hsa-mir-1179	MI0006272	-0.171277369	0.898705205	2.750583323	0.015184101	0.896940866	-3.320814112
hsa-miR-3685	MIMAT0018113	-0.172027685	1.037790946	1.472720575	0.162105799	0.903074508	-4.420150995
hsa-miR-4299	MIMAT0016851	-0.173068286	1.171865219	1.246342481	0.232312498	0.919664114	-4.579067082
hsa-miR-7112-5p	MIMAT0028121	-0.17320142	1.039793113	1.247679147	0.231836448	0.919664114	-4.578182736
hsa-mir-4721	MI0017356	-0.173518021	1.065122647	1.83889022	0.086406667	0.896940866	-4.130771604
hsa-miR-664a-5p	MIMAT0005948	-0.173956101	4.164420298	0.388177304	0.703499477	0.986573137	-4.976379997
hsa-miR-548aq-3p	MIMAT0022264	-0.174749965	0.881269728	1.921137114	0.074506598	0.896940866	-4.061543387
hsa-miR-6730-3p	MIMAT0027362	-0.174928293	1.245022665	1.863059967	0.082744311	0.896940866	-4.11056148
hsa-miR-1243	MIMAT0005894	-0.174970656	0.856349494	1.978942209	0.067049694	0.896940866	-4.012160997
hsa-mir-3126	MI0014143	-0.175731332	0.920193493	1.850029351	0.084701178	0.896940866	-4.121471686
hsa-miR-30b-3p	MIMAT0004589	-0.176387868	1.32487318	1.011533802	0.328271012	0.930208704	-4.723171164
hsa-mir-4634	MI0017261	-0.176693514	2.166540522	0.775454629	0.450478981	0.950247229	-4.842988595
hsa-miR-1203	MIMAT0005866	-0.177339893	0.991098914	2.199309786	0.04445666	0.896940866	-3.81941976
hsa-miR-485-5p	MIMAT0002175	-0.178497639	1.260965623	1.077333602	0.298852	0.927057986	-4.68513855
hsa-miR-4438	MIMAT0018956	-0.178743768	0.966570373	2.49451498	0.025174819	0.896940866	-3.553844789
hsa-miR-411-5p	MIMAT0003329	-0.178761506	1.033484717	1.992852721	0.065359497	0.896940866	-4.000195021
hsa-miR-639	MIMAT0003309	-0.179200188	0.98250934	1.581645831	0.135197922	0.896940866	-4.337760643
hsa-mir-487b	MI0003530	-0.180034011	1.150263989	1.024031729	0.322528385	0.930208704	-4.716095212
hsa-miR-1260b	MIMAT0015041	-0.180133391	1.480763798	0.85601951	0.405850056	0.930619902	-4.805148609
hsa-mir-342	MI0000805	-0.181395734	1.154012926	2.476046476	0.026099685	0.896940866	-3.570605798
hsa-miR-635	MIMAT0003305	-0.181496955	0.891251558	1.735371993	0.103775484	0.896940866	-4.215950924
hsa-miR-181c-5p	MIMAT0000258	-0.181818173	1.341717371	0.877935418	0.394230851	0.930573075	-4.79429545
hsa-let-7g	MI0000433	-0.182220367	0.936222468	2.249454457	0.040416672	0.896940866	-3.774766768
hsa-miR-378d	MIMAT0018926	-0.183348846	1.136288209	1.879410568	0.08034618	0.896940866	-4.096825019
hsa-mir-6814	MI0022659	-0.18358231	0.996522828	1.777539813	0.096359339	0.896940866	-4.181534375
hsa-miR-133b	MIMAT0000770	-0.183695686	1.056450283	0.807540036	0.432346484	0.94301493	-4.82831077
hsa-let-7e-3p	MIMAT0004485	-0.183725694	0.792114787	2.347468532	0.033493953	0.896940866	-3.686861289
hsa-miR-548b-5p	MIMAT0004798	-0.184243134	1.190356863	1.86775844	0.082048707	0.896940866	-4.106619455
hsa-miR-760	MIMAT0004957	-0.185511173	1.799222409	0.892602619	0.386580176	0.930573075	-4.786900959
hsa-mir-6741	MI0022586	-0.185787359	0.891904021	2.736628315	0.015611794	0.896940866	-3.333515863
hsa-miR-548s	MIMAT0014987	-0.186556023	0.860344353	2.286921385	0.037625207	0.896940866	-3.74125366
hsa-miR-92b-5p	MIMAT0004792	-0.187017823	2.86654438	0.335042899	0.742369335	0.990306303	-4.988024761
hsa-mir-625	MI0003639	-0.187138335	1.07372916	2.034733242	0.06050259	0.896940866	-3.963988366
hsa-miR-2113	MIMAT0009206	-0.188145214	0.899390509	1.993290929	0.065306888	0.896940866	-3.999817571
hsa-miR-410-5p	MIMAT0026558	-0.189437024	0.950003277	2.336852511	0.034185939	0.896940866	-3.696417289
hsa-mir-495	MI0003135	-0.190114596	1.166510932	2.474633183	0.02617178	0.896940866	-3.57188791
hsa-miR-1972	MIMAT0009447	-0.190593299	1.20793458	0.371125707	0.715886555	0.987260208	-4.98029856
hsa-miR-23b-3p	MIMAT0000418	-0.190919642	10.52058944	1.239280103	0.23484048	0.919664114	-4.583728233
hsa-miR-3180	MIMAT0018178	-0.191008752	1.906172088	0.650912482	0.525239619	0.960979619	-4.894884488
hsa-miR-411-3p	MIMAT0004813	-0.191062853	1.188466928	1.479261927	0.160371874	0.903074508	-4.415303747
hsa-miR-5584-3p	MIMAT0022284	-0.191391931	0.851989388	2.226552059	0.042217305	0.896940866	-3.795190921
hsa-miR-215-5p	MIMAT0000272	-0.192178565	0.944623166	2.728192219	0.015875991	0.896940866	-3.34119557
hsa-miR-5708	MIMAT0022502	-0.193348319	0.897157205	2.320928821	0.035249228	0.896940866	-3.710736297
hsa-mir-4796	MI0017443	-0.193362483	1.018320526	2.725018423	0.015976505	0.896940866	-3.344085013
hsa-miR-7113-5p	MIMAT0028123	-0.193891255	0.989513239	2.149471753	0.048842136	0.896940866	-3.863544432
hsa-miR-6746-5p	MIMAT0027392	-0.194433034	1.451127447	1.110074557	0.284958331	0.927057986	-4.665511043
hsa-mir-6812	MI0022657	-0.194724995	1.084437614	2.025821888	0.061507531	0.896940866	-3.971714505
hsa-miR-622	MIMAT0003291	-0.195187228	0.888985428	2.008629167	0.063489603	0.896940866	-3.986587191
hsa-miR-200b-3p	MIMAT0000318	-0.195658581	1.065443408	1.579747159	0.135632247	0.896940866	-4.339226252
hsa-miR-7846-3p	MIMAT0030421	-0.195927965	1.036411278	2.444018187	0.027780915	0.896940866	-3.59964142
hsa-miR-2392	MIMAT0019043	-0.196320688	2.982705859	0.601261056	0.556908901	0.96620986	-4.913255952
hsa-miR-4444	MIMAT0018962	-0.196633197	0.923023407	2.514359195	0.024215984	0.896940866	-3.53582234
hsa-miR-548g-5p	MIMAT0022722	-0.196743957	0.927168893	2.830664473	0.012941477	0.896940866	-3.247996051
hsa-miR-548x-5p	MIMAT0022733	-0.196743957	0.927168893	2.830664473	0.012941477	0.896940866	-3.247996051
hsa-miR-548aj-5p	MIMAT0022739	-0.196743957	0.927168893	2.830664473	0.012941477	0.896940866	-3.247996051
hsa-mir-5090	MI0017979	-0.198930744	1.290145427	1.490427447	0.157448018	0.903074508	-4.406999289
hsa-let-7d-5p	MIMAT0000065	-0.200153927	11.8891997	0.937831493	0.363623238	0.930573075	-4.763446942
hsa-miR-4724-5p	MIMAT0019841	-0.200666409	0.871104073	2.17520568	0.046529844	0.896940866	-3.840794321
hsa-miR-20b-5p	MIMAT0001413	-0.201317753	7.372426497	0.982248261	0.342013276	0.930208704	-4.739474568
hsa-miR-548h-5p	MIMAT0005928	-0.201379293	0.962971161	1.97928854	0.067007135	0.896940866	-4.011863455
hsa-mir-215	MI0000291	-0.201697321	0.913182668	2.274423559	0.038535602	0.896940866	-3.752445862
hsa-mir-1321	MI0006652	-0.202814678	0.953303312	2.224308417	0.04239768	0.896940866	-3.797189131
hsa-miR-299-5p	MIMAT0002890	-0.203037624	1.084341577	1.393919524	0.18424543	0.903156384	-4.477468474

hsa-miR-497-3p	MIMAT0004768	-0.205385568	0.895767087	3.234344121	0.00573986	0.896940866	-2.885281933
hsa-miR-498-5p	MIMAT0002824	-0.205489636	1.570521956	1.37741033	0.189184868	0.903156384	-4.489216847
hsa-miR-7515	MIMAT0029310	-0.206208798	0.931105626	2.188128634	0.045407418	0.896940866	-3.829342377
hsa-miR-6889-5p	MIMAT0027678	-0.207167883	1.556281674	1.106664872	0.286382348	0.927057986	-4.667576411
hsa-miR-4266	MIMAT0016892	-0.207881454	0.776969423	2.67689844	0.017578168	0.896940866	-3.38790358
hsa-mir-4530	MI0016897	-0.209552092	2.032461613	1.207067941	0.246644478	0.919664114	-4.60474339
hsa-mir-3679	MI0016080	-0.20957461	2.420117403	0.965765725	0.349924424	0.930573075	-4.748477543
hsa-miR-4750-5p	MIMAT0019887	-0.209825669	3.415550364	0.404543535	0.691691519	0.98554565	-4.972458342
hsa-miR-4252	MIMAT0016886	-0.210142679	0.899503671	2.333026811	0.0344386	0.896940866	-3.699859096
hsa-miR-1244	MIMAT0005896	-0.210740365	1.11159827	1.663718812	0.117528779	0.896940866	-4.27347585
hsa-miR-1275	MIMAT0005929	-0.210916545	4.289373832	0.529609198	0.604362457	0.975026084	-4.937364529
hsa-mir-8070	MI0025906	-0.210928735	0.857078895	3.073486543	0.00794457	0.896940866	-3.028643978
hsa-mir-4537	MI0016908	-0.211047507	1.124568252	2.197952124	0.044571108	0.896940866	-3.820625295
hsa-miR-4640-5p	MIMAT0019699	-0.212805476	2.079284145	0.544626137	0.594251847	0.970952097	-4.93254986
hsa-miR-6127	MIMAT0024610	-0.213205188	3.32317035	0.63173299	0.537351296	0.96159396	-4.902140695
hsa-mir-421	MI0003685	-0.213637711	1.053304182	2.582296583	0.021191255	0.896940866	-3.474042416
hsa-miR-491-5p	MIMAT0002807	-0.214137594	1.231079863	1.470549644	0.162684696	0.903074508	-4.421756741
hsa-miR-302d-3p	MIMAT0000718	-0.21528312	1.01072721	2.497129975	0.025046423	0.896940866	-3.551470592
hsa-mir-4520-1	MI0016886	-0.216619694	1.095865738	2.21388491	0.043245141	0.896940866	-3.806466014
hsa-miR-126-3p	MIMAT0000445	-0.21906907	10.9690466	1.214037335	0.244052308	0.919664114	-4.600230898
hsa-miR-342-5p	MIMAT0004694	-0.219080489	3.898805033	0.468049386	0.646676349	0.982649571	-4.955763307
hsa-miR-3934-5p	MIMAT0018349	-0.220933713	1.065517243	1.597709965	0.131570535	0.896940866	-4.325320553
hsa-mir-6886	MI0022733	-0.222370555	0.96320494	1.882251145	0.079935969	0.896940866	-4.094433402
hsa-miR-29b-3p	MIMAT0000100	-0.222833143	1.02290765	2.783371621	0.014223528	0.896940866	-3.290982874
hsa-let-7c-5p	MIMAT0000064	-0.223130503	11.96395264	0.948284317	0.358454319	0.930573075	-4.757888275
hsa-mir-1301	MI0003815	-0.223223922	1.111796898	2.001283724	0.064354064	0.896940866	-3.992927732
hsa-miR-6737-5p	MIMAT0027375	-0.223720895	1.711304944	0.927547679	0.368758663	0.930573075	-4.768865424
hsa-miR-6848-5p	MIMAT0027596	-0.224045137	3.128154189	0.418777012	0.681489146	0.985064407	-4.968920322
hsa-mir-338	MI0000814	-0.224132493	0.897592467	2.591290479	0.020819117	0.896940866	-3.465856732
hsa-miR-548i	MIMAT0005935	-0.224506166	0.932078769	2.714012993	0.016329849	0.896940866	-3.354105218
hsa-miR-6831-5p	MIMAT0027562	-0.225288299	1.562987955	0.920867219	0.372121295	0.930573075	-4.772358443
hsa-miR-501-3p	MIMAT0004774	-0.227378694	2.221112621	0.839335537	0.414845595	0.934032076	-4.813252086
hsa-miR-3613-5p	MIMAT0017990	-0.227574858	3.508897381	0.415486795	0.683841906	0.98554565	-4.969748683
hsa-miR-484	MIMAT0002174	-0.229223377	2.051171875	0.547225725	0.592510308	0.970056246	-4.931703497
hsa-miR-4694-5p	MIMAT0019786	-0.229278448	0.844449205	3.257475483	0.005477372	0.896940866	-2.864832384
hsa-miR-29b-2-5p	MIMAT0004515	-0.230078553	1.360108519	1.437002536	0.171851207	0.903074508	-4.446380645
hsa-miR-766-5p	MIMAT0022714	-0.232586159	3.1219916	0.743767391	0.468846712	0.951465791	-4.85696558
hsa-miR-628-5p	MIMAT0004809	-0.233331113	1.191036151	2.046120956	0.059240264	0.896940866	-3.954098421
hsa-mir-1247	MI0006382	-0.233908707	0.831344122	3.533031142	0.003135178	0.896940866	-2.62509411
hsa-miR-296-3p	MIMAT0004679	-0.234836554	1.306427485	1.4809386	0.159929939	0.903074508	-4.414059164
hsa-miR-6800-3p	MIMAT0027501	-0.23739893	1.859001924	1.307100793	0.211438806	0.91645315	-4.5381904
hsa-mir-409	MI0001735	-0.237582527	1.012756764	2.778518234	0.014361892	0.896940866	-3.295397287
hsa-miR-4667-5p	MIMAT0019743	-0.237622622	1.163001445	1.764194866	0.098654117	0.896940866	-4.192469458
hsa-miR-488-5p	MIMAT0002804	-0.23809413	0.868820531	2.835453632	0.012818116	0.896940866	-3.243646227
hsa-mir-206	MI0000490	-0.238175603	1.043195431	2.177185227	0.046356253	0.896940866	-3.839041266
hsa-miR-517a-3p	MIMAT0002852	-0.23935021	0.842196189	3.479396224	0.003494747	0.896940866	-2.671152545
hsa-miR-517b-3p	MIMAT0002857	-0.23935021	0.842196189	3.479396224	0.003494747	0.896940866	-2.671152545
hsa-miR-1184	MIMAT0005829	-0.239740307	1.123546357	2.246050646	0.040679663	0.896940866	-3.777805314
hsa-miR-6511a-3p	MIMAT0025479	-0.240389977	1.217918597	1.517872612	0.150450445	0.902932883	-4.386425017
hsa-miR-500a-3p	MIMAT0002871	-0.241974162	3.019005459	0.649516062	0.526116208	0.960979619	-4.895419539
hsa-miR-497-5p	MIMAT0002820	-0.243037142	0.942809427	2.563592978	0.021985725	0.896940866	-3.491060928
hsa-miR-641	MIMAT0003311	-0.243119707	1.196292673	3.133734297	0.00703472	0.896940866	-2.974727259
hsa-miR-152-3p	MIMAT0000438	-0.244970424	5.262082524	0.875268389	0.39563284	0.930573075	-4.795628786
hsa-miR-363-3p	MIMAT0000707	-0.247002495	2.983859623	0.665497861	0.516133385	0.960979619	-4.889232962
hsa-miR-28-3p	MIMAT0004502	-0.250342065	4.404575748	0.468830507	0.646131008	0.982649571	-4.955543428
hsa-miR-6075	MIMAT0023700	-0.25267713	4.393670412	0.422487626	0.678839875	0.985064407	-4.967978549
hsa-miR-6728-5p	MIMAT0027357	-0.253654968	1.381854384	1.776464272	0.096542524	0.896940866	-4.182417147
hsa-miR-454-3p	MIMAT0003885	-0.255005084	1.098269568	1.511110657	0.152149758	0.902932883	-4.391515226
hsa-miR-151b	MIMAT0010214	-0.255685303	7.855428922	1.150444079	0.268499617	0.927057986	-4.640687092
hsa-miR-520g-5p	MIMAT0026611	-0.256950911	0.91433602	1.855426231	0.083885738	0.896940866	-4.116957051
hsa-miR-6870-5p	MIMAT0027640	-0.257657994	2.89418697	0.968710296	0.348501763	0.930573075	-4.746878397
hsa-miR-154-5p	MIMAT0000452	-0.258203223	1.152331473	2.038575975	0.060073896	0.896940866	-3.96065314
hsa-miR-3137	MIMAT0015005	-0.259003327	1.107652976	2.414305378	0.029432355	0.896940866	-3.626536871
hsa-miR-1271-5p	MIMAT0005796	-0.263017644	1.256419197	1.919098479	0.074782766	0.896940866	-4.063274482
hsa-miR-3679-5p	MIMAT0018104	-0.264328171	1.855252703	0.745799891	0.467654947	0.951465791	-4.85608468
hsa-miR-4711-5p	MIMAT0019816	-0.266308663	0.865863525	2.741551081	0.015459603	0.896940866	-3.329034888

hsa-miR-1266-5p	MIMAT0005920	-0.267946662	1.094924174	2.101386541	0.053450425	0.896940866	-3.905846376
hsa-miR-4498	MIMAT0019033	-0.269191008	2.250377645	0.918555053	0.373290013	0.930573075	-4.773562459
hsa-miR-6879-5p	MIMAT0027658	-0.26924396	3.50329796	0.535421318	0.60043914	0.97307982	-4.935516154
hsa-mir-6721	MI0022556	-0.270253134	1.016702172	3.449409428	0.003713521	0.896940866	-2.697037815
hsa-miR-6131	MIMAT0024615	-0.270344117	0.992280877	2.694378509	0.016979141	0.896940866	-3.371984451
hsa-mir-484	MI0002468	-0.273974568	2.634576175	1.013306984	0.327451821	0.930208704	-4.722171521
hsa-miR-432-5p	MIMAT0002814	-0.275183708	7.058855787	0.617812136	0.546238537	0.965790957	-4.907281799
hsa-miR-574-3p	MIMAT0003239	-0.275249569	5.150376113	0.436876152	0.668608589	0.984005175	-4.964250894
hsa-miR-194-5p	MIMAT0000460	-0.275618799	4.778975104	0.748404167	0.466130635	0.951465791	-4.854952825
hsa-mir-890	MI0005533	-0.280051244	0.939360052	3.351693583	0.004526148	0.896940866	-2.782025353
hsa-miR-10b-3p	MIMAT0004556	-0.280491902	1.118571591	2.833405544	0.01287073	0.896940866	-3.245506352
hsa-miR-7111-5p	MIMAT0028119	-0.280726235	1.260428435	2.051026205	0.058703992	0.896940866	-3.949832615
hsa-let-7e-5p	MIMAT0000066	-0.280823262	9.308308256	0.681398674	0.506309825	0.960979619	-4.882941258
hsa-miR-6511b-5p	MIMAT0025847	-0.286317509	3.668977179	0.888365886	0.388779784	0.930573075	-4.789047643
hsa-miR-221-3p	MIMAT0000278	-0.289531804	9.266730223	1.197659382	0.250177616	0.919664114	-4.610804786
hsa-miR-575	MIMAT0003240	-0.289925037	1.096387185	1.869964456	0.08172392	0.896940866	-4.104767135
hsa-miR-485-3p	MIMAT0002176	-0.291936404	1.047184845	3.280232539	0.005230794	0.896940866	-2.844758504
hsa-miR-192-5p	MIMAT0000222	-0.293164016	2.643499291	0.861931524	0.402693579	0.930573075	-4.802244101
hsa-miR-487a-3p	MIMAT0002178	-0.299327346	1.549506261	1.030480148	0.319593906	0.930208704	-4.712416912
hsa-miR-7-1-3p	MIMAT0004553	-0.301922736	1.425468179	1.600758625	0.130891616	0.896940866	-4.322951655
hsa-miR-433-3p	MIMAT0001627	-0.303894674	1.237851002	1.155722271	0.266402051	0.927057986	-4.637391483
hsa-miR-4655-5p	MIMAT0019721	-0.309507158	1.75442118	1.115322002	0.282777148	0.927057986	-4.662322872
hsa-mir-7851	MI0025521	-0.310448311	1.071447667	2.856788387	0.012282373	0.896940866	-3.224277057
hsa-miR-181d-5p	MIMAT0002821	-0.311857429	1.651143822	1.553107182	0.141852726	0.896940866	-4.359682896
hsa-miR-3120-3p	MIMAT0014982	-0.312769406	1.322566921	1.790381166	0.094195829	0.896940866	-4.170975097
hsa-miR-301a-3p	MIMAT0000688	-0.313766423	1.498419958	1.28872403	0.217588463	0.917147059	-4.550698601
hsa-miR-1299	MIMAT0005887	-0.314735876	1.514792971	0.614169317	0.548577395	0.965790957	-4.908609607
hsa-miR-4685-5p	MIMAT0019771	-0.315785168	1.709810931	1.00255363	0.332442309	0.930208704	-4.728211959
hsa-miR-4767	MIMAT0019919	-0.317307171	0.960374106	2.088345909	0.05476762	0.896940866	-3.917268908
hsa-miR-27a-3p	MIMAT0000084	-0.318123234	8.864376612	1.114086155	0.283289718	0.927057986	-4.663074777
hsa-miR-6877-5p	MIMAT0027654	-0.318317721	1.170915339	2.840276008	0.012695059	0.896940866	-3.239266913
hsa-miR-371b-5p	MIMAT0019892	-0.318864975	4.937851062	0.705316285	0.491740265	0.959967659	-4.87322297
hsa-miR-183-5p	MIMAT0000261	-0.320393467	1.597103815	0.773895774	0.451371933	0.950247229	-4.843688312
hsa-miR-1229-5p	MIMAT0022942	-0.324180385	5.010899855	0.655100983	0.522615293	0.960979619	-4.893273294
hsa-miR-197-3p	MIMAT0000227	-0.32656339	2.782368176	0.617004749	0.546756445	0.965790957	-4.90757672
hsa-miR-660-5p	MIMAT0003338	-0.328637421	2.540807482	0.718316567	0.483926451	0.957574873	-4.867813348
hsa-miR-34a-5p	MIMAT0000255	-0.32882177	0.977089441	3.199647659	0.006157206	0.896940866	-2.91603882
hsa-miR-330-3p	MIMAT0000751	-0.334555329	2.744223612	0.874959814	0.395795265	0.930573075	-4.795782829
hsa-miR-8071	MIMAT0030998	-0.335384302	1.695105465	1.21384055	0.244125208	0.919664114	-4.600358572
hsa-miR-532-5p	MIMAT0002888	-0.338173997	3.161663495	0.973988789	0.345961659	0.930573075	-4.744001686
hsa-miR-3135b	MIMAT0018985	-0.341300402	6.290395619	1.60865524	0.129147031	0.896940866	-4.316803992
hsa-miR-99b-3p	MIMAT0004678	-0.342284418	1.271123305	1.455085292	0.166858411	0.903074508	-4.4331522
hsa-miR-193a-5p	MIMAT0004614	-0.344203871	1.59101446	1.614801772	0.127802935	0.896940866	-4.312007129
hsa-miR-7150	MIMAT0028211	-0.347476213	4.361359853	0.703810296	0.492650257	0.960307746	-4.87384386
hsa-miR-493-5p	MIMAT0002813	-0.348413838	1.134805493	1.69748559	0.11086133	0.896940866	-4.246522319
hsa-miR-3617-5p	MIMAT0017997	-0.349521456	1.411681052	1.733589913	0.104099665	0.896940866	-4.217396508
hsa-miR-30a-3p	MIMAT0000088	-0.349802908	1.405530305	1.431465348	0.173404567	0.903074508	-4.450410311
hsa-miR-7109-5p	MIMAT0028115	-0.355799954	1.584609291	1.324666882	0.205691022	0.90933792	-4.526119158
hsa-miR-378i	MIMAT0019074	-0.356791239	1.694809558	1.402466739	0.181729899	0.903156384	-4.471349898
hsa-miR-6875-5p	MIMAT0027650	-0.362322156	1.647460837	1.024111223	0.322492092	0.930208704	-4.716049981
hsa-miR-483-5p	MIMAT0004761	-0.363605649	2.054342204	0.796851629	0.43833441	0.946344991	-4.833258515
hsa-miR-4306	MIMAT0016858	-0.364060186	4.049431635	0.682956248	0.505353427	0.960979619	-4.882317662
hsa-let-7a-5p	MIMAT0000062	-0.36948314	12.37005272	1.840482995	0.086160938	0.896940866	-4.129443335
hsa-miR-6782-5p	MIMAT0027464	-0.372670801	3.800957305	0.869380064	0.398739963	0.930573075	-4.798560245
hsa-let-7i-5p	MIMAT0000415	-0.373006295	10.20792938	2.400526782	0.030229431	0.896940866	-3.638993925
hsa-mir-494	MI0003134	-0.376862716	1.264151788	2.894613451	0.011385836	0.896940866	-3.189974523
hsa-miR-6808-5p	MIMAT0027516	-0.377067232	2.472871573	0.991689457	0.337539141	0.930208704	-4.734261332
hsa-miR-584-5p	MIMAT0003249	-0.377514563	7.998232177	1.394291018	0.184135506	0.903156384	-4.477203048
hsa-miR-3197	MIMAT0015082	-0.382202381	2.081533327	0.97403287	0.345940501	0.930573075	-4.743977608
hsa-miR-1273h-5p	MIMAT0030415	-0.38249476	1.813883896	1.535487385	0.146098723	0.897988521	-4.373101309
hsa-miR-6124	MIMAT0024597	-0.384792715	2.100620406	1.62737788	0.125090278	0.896940866	-4.302160993
hsa-miR-933	MIMAT0004976	-0.386224981	1.724061634	1.174247338	0.259138794	0.923131598	-4.625734742
hsa-miR-19a-3p	MIMAT0000073	-0.386455129	3.248132702	0.746942487	0.466985797	0.951465791	-4.855588526
hsa-miR-134-5p	MIMAT0000447	-0.386780898	5.577209254	0.996273857	0.335381673	0.930208704	-4.731715211
hsa-miR-376a-3p	MIMAT0000729	-0.388831251	1.38075018	1.813192447	0.090458523	0.896940866	-4.152130532
hsa-miR-132-3p	MIMAT0000426	-0.393724874	3.243343445	0.85637062	0.40566214	0.930588488	-4.804976594

hsa-miR-3180-3p	MIMAT0015058	-0.396574386	1.995505048	0.929447637	0.367806141	0.930573075	-4.767868118
hsa-miR-320e	MIMAT0015072	-0.400563569	6.893323109	1.204528292	0.247594349	0.919664114	-4.60638299
hsa-miR-27b-3p	MIMAT0000419	-0.401304735	7.688798589	1.621440548	0.126364718	0.896940866	-4.306814723
hsa-miR-125a-5p	MIMAT0000443	-0.40392056	6.404224123	0.952657592	0.35630696	0.930573075	-4.755547404
hsa-miR-30a-5p	MIMAT0000087	-0.413281697	5.025008573	1.396270726	0.183550622	0.903156384	-4.475787796
hsa-miR-6813-5p	MIMAT0027526	-0.41708548	2.781544259	0.760314911	0.459198126	0.951465791	-4.849731379
hsa-miR-6787-5p	MIMAT0027474	-0.422234093	3.921707044	0.9032579	0.381085406	0.930573075	-4.781463751
hsa-miR-28-5p	MIMAT0000085	-0.422261967	5.512015767	1.249161787	0.231309306	0.919664114	-4.577201016
hsa-miR-6784-5p	MIMAT0027468	-0.423210746	1.84438453	2.674683492	0.017655516	0.896940866	-3.3899208
hsa-miR-210-3p	MIMAT0000267	-0.424281388	3.00677845	0.859846495	0.403804934	0.930573075	-4.803270419
hsa-miR-5189-5p	MIMAT0021120	-0.427849662	2.668883607	1.183800641	0.255452884	0.921727531	-4.619669092
hsa-miR-543	MIMAT0004954	-0.435527722	1.377645018	2.217959385	0.04291201	0.896940866	-3.802841009
hsa-miR-185-3p	MIMAT0004611	-0.44268018	1.367141884	2.255003366	0.039991352	0.896940866	-3.769811086
hsa-mir-185	MI0000482	-0.442756134	1.46299793	3.617434571	0.002643072	0.896940866	-2.553263369
hsa-miR-6778-5p	MIMAT0027456	-0.455343787	4.088966027	1.090295571	0.2932928	0.927057986	-4.677423019
hsa-miR-224-5p	MIMAT0000281	-0.460619446	1.24697159	2.065791994	0.057116469	0.896940866	-3.936971301
hsa-miR-1285-3p	MIMAT0005876	-0.465198042	1.350137211	1.313614665	0.209292597	0.914362911	-4.533727134
hsa-miR-4284	MIMAT0016915	-0.465527055	1.947043579	1.617081777	0.127307419	0.896940866	-4.31022519
hsa-miR-125a-3p	MIMAT0004602	-0.467731947	1.475033982	1.739819529	0.102970289	0.896940866	-4.212339978
hsa-miR-378c	MIMAT0016847	-0.471413376	1.887116911	1.660287629	0.118225382	0.896940866	-4.276198736
hsa-miR-425-3p	MIMAT0001343	-0.477358927	3.801751204	0.960942052	0.352263759	0.930573075	-4.751088495
hsa-miR-6820-5p	MIMAT0027540	-0.478689826	2.487248784	1.088862175	0.293903767	0.927057986	-4.678279795
hsa-miR-629-5p	MIMAT0004810	-0.484330776	3.654708786	0.982072306	0.342097056	0.930208704	-4.739571338
hsa-miR-6815-5p	MIMAT0027530	-0.485890579	1.50893692	1.604973904	0.129957844	0.896940866	-4.319672087
hsa-miR-505-5p	MIMAT0004776	-0.490818043	3.746387723	0.780783327	0.447434934	0.950247229	-4.840587309
hsa-miR-328-3p	MIMAT0000752	-0.493827202	1.717976778	1.345423749	0.199061676	0.905221726	-4.51171289
hsa-miR-125b-2-3p	MIMAT0004603	-0.493878716	1.197261551	1.047999197	0.311719187	0.930208704	-4.702330275
hsa-miR-654-3p	MIMAT0004814	-0.499024042	1.451850013	2.062248592	0.057493797	0.896940866	-3.94006044
hsa-miR-324-5p	MIMAT0000761	-0.499063187	5.396599085	0.913147379	0.376033201	0.930573075	-4.776368438
hsa-miR-29c-3p	MIMAT0000681	-0.50062029	1.512299851	2.524923836	0.023719871	0.896940866	-3.526222659
hsa-miR-148a-3p	MIMAT0000243	-0.511992118	2.286344134	1.280323296	0.220446676	0.917147059	-4.556375163
hsa-miR-382-5p	MIMAT0000737	-0.513249736	5.985770488	1.538990839	0.145246	0.896940866	-4.37044043
hsa-miR-15b-5p	MIMAT0000417	-0.513937039	10.56735008	1.926405854	0.073797115	0.896940866	-4.057066059
hsa-miR-222-3p	MIMAT0000279	-0.516841579	7.356111651	1.793101627	0.093743046	0.896940866	-4.16873352
hsa-miR-431-5p	MIMAT0001625	-0.522976165	1.462006086	1.724148467	0.10583204	0.896940866	-4.225042848
hsa-miR-181b-5p	MIMAT0000257	-0.524511781	4.306620894	1.52560908	0.148525873	0.902434047	-4.380584498
hsa-miR-1-3p	MIMAT0000416	-0.528788742	1.170568766	1.443015118	0.170177534	0.903074508	-4.441993784
hsa-miR-199a-3p	MIMAT0000232	-0.535276969	8.178053795	2.002179824	0.064248033	0.896940866	-3.99215467
hsa-miR-199b-3p	MIMAT0004563	-0.535276969	8.178053795	2.002179824	0.064248033	0.896940866	-3.99215467
hsa-miR-6802-5p	MIMAT0027504	-0.538834524	3.658880553	0.986235613	0.340118596	0.930208704	-4.737277818
hsa-miR-2277-5p	MIMAT0017352	-0.539472251	2.140374258	2.517187887	0.024082185	0.896940866	-3.533252335
hsa-miR-130b-3p	MIMAT0000691	-0.541908456	5.597932751	0.927173955	0.368946225	0.930573075	-4.769061393
hsa-miR-133a-3p	MIMAT0000427	-0.549729064	1.164670561	1.288515104	0.217659188	0.917147059	-4.550840094
hsa-miR-4656	MIMAT0019723	-0.550789392	3.376334902	1.450767651	0.168039474	0.903074508	-4.436320284
hsa-miR-4467	MIMAT0018994	-0.575232741	6.775216627	1.079564112	0.297889863	0.927057986	-4.683816013
hsa-miR-30e-5p	MIMAT0000692	-0.58944486	4.096128765	1.192380991	0.252176819	0.920370901	-4.614189977
hsa-miR-495-3p	MIMAT0002817	-0.598550412	1.325885874	2.071977481	0.056463239	0.896940866	-3.93157467
hsa-miR-376c-3p	MIMAT0000720	-0.599755404	1.737922061	1.998169851	0.064723753	0.896940866	-3.995613094
hsa-miR-339-3p	MIMAT0004702	-0.601441786	3.831503797	1.598629098	0.131365532	0.896940866	-4.324606627
hsa-miR-24-2-5p	MIMAT0004497	-0.608312563	2.34046691	1.704270929	0.109562032	0.896940866	-4.241072256
hsa-miR-195-5p	MIMAT0000461	-0.609944718	3.961348687	1.026214963	0.321532693	0.930208704	-4.714851936
hsa-miR-503-5p	MIMAT0002874	-0.623201345	2.330211784	1.741394296	0.102686507	0.896940866	-4.211060334
hsa-miR-22-5p	MIMAT0004495	-0.649354622	1.381507646	3.271815972	0.005320674	0.896940866	-2.85217746
hsa-miR-370-3p	MIMAT0000722	-0.666356119	1.593105976	2.323416523	0.035081084	0.896940866	-3.708500481
hsa-let-7g-5p	MIMAT0000414	-0.668499157	8.631558809	2.00755611	0.063615223	0.896940866	-3.987513964
hsa-miR-26b-5p	MIMAT0000083	-0.675278283	2.053531621	1.667238674	0.116817874	0.896940866	-4.270679492
hsa-miR-6511a-5p	MIMAT0025478	-0.681686648	2.957139927	1.578840326	0.135840107	0.896940866	-4.339925892
hsa-miR-140-5p	MIMAT0000431	-0.701837301	1.512251286	2.123152219	0.051316857	0.896940866	-3.886733318
hsa-miR-143-3p	MIMAT0000435	-0.712683534	4.556424064	1.119510228	0.281045241	0.927057986	-4.659769914
hsa-miR-331-3p	MIMAT0000760	-0.729624845	1.743916745	1.562729629	0.139578448	0.896940866	-4.352317114
hsa-miR-10a-5p	MIMAT0000253	-0.731264622	2.132630736	2.109469212	0.052648747	0.896940866	-3.898755673
hsa-miR-21-5p	MIMAT0000076	-0.78601482	5.784960672	1.38375846	0.18727292	0.903156384	-4.484710303
hsa-miR-223-3p	MIMAT0000280	-0.786726525	7.621195452	2.625377568	0.019464976	0.896940866	-3.434823444
hsa-miR-18b-5p	MIMAT0001412	-0.794859492	4.287589672	1.636346512	0.123186172	0.896940866	-4.295113645
hsa-miR-409-3p	MIMAT0001639	-0.803249729	4.272692078	1.244610852	0.232930352	0.919664114	-4.580211721
hsa-miR-145-5p	MIMAT0000437	-0.833047718	5.280401893	1.589055436	0.133514301	0.896940866	-4.332031489

hsa-miR-628-3p	MIMAT0003297	-0.833122435	5.270203667	1.439323569	0.171203513	0.903074508	-4.44468857
hsa-miR-30c-5p	MIMAT0000244	-0.835703126	5.725441842	1.729411212	0.104863307	0.896940866	-4.220783279
hsa-miR-182-5p	MIMAT0000259	-0.84280676	5.504473615	1.966177058	0.068635586	0.896940866	-4.023114329
hsa-let-7d-3p	MIMAT0004484	-0.849339489	2.658674968	1.465941794	0.163919128	0.903074508	-4.425160081
hsa-miR-10b-5p	MIMAT0000254	-0.850434701	2.955147562	2.62993971	0.01929031	0.896940866	-3.430669166
hsa-miR-127-3p	MIMAT0000446	-0.853069582	3.246315654	1.710627835	0.108356859	0.896940866	-4.235956243
hsa-miR-128-3p	MIMAT0000424	-0.911072577	2.655653718	1.61001445	0.128848764	0.896940866	-4.315744117
hsa-miR-125b-5p	MIMAT0000423	-0.915041653	2.634709382	0.861691551	0.402821385	0.930573075	-4.802362332
hsa-miR-339-5p	MIMAT0000764	-0.92709939	4.521836548	1.909285402	0.076125077	0.896940866	-4.071596818
hsa-let-7f-5p	MIMAT0000067	-0.967507169	8.816266401	2.100620634	0.053526972	0.896940866	-3.906517854
hsa-miR-148b-3p	MIMAT0000759	-0.973894513	4.666863542	1.85872751	0.0833904	0.896940866	-4.114192623
hsa-miR-98-5p	MIMAT0000096	-0.974931836	2.812664133	1.623234063	0.125978569	0.896940866	-4.305409938
hsa-miR-502-3p	MIMAT0004775	-0.994634607	2.919657207	2.22997004	0.041943898	0.896940866	-3.792145909
hsa-miR-30e-3p	MIMAT0000693	-1.013450515	2.150775534	2.312625637	0.035815952	0.896940866	-3.718195478
hsa-miR-130a-3p	MIMAT0000425	-1.024869942	4.8114398	2.413859404	0.029457839	0.896940866	-3.626940227
hsa-miR-335-5p	MIMAT0000765	-1.043394334	2.90805938	1.593523774	0.132507688	0.896940866	-4.3285692
hsa-miR-30b-5p	MIMAT0000420	-1.050990292	5.098198252	1.853618958	0.084158023	0.896940866	-4.118469523
hsa-miR-199a-5p	MIMAT0000231	-1.076117381	5.302540594	1.869433937	0.081801922	0.896940866	-4.10521268
hsa-miR-100-5p	MIMAT0000098	-1.094469276	1.457965941	1.924094093	0.07410766	0.896940866	-4.059031175
hsa-miR-421	MIMAT0003339	-1.213435205	4.363291931	1.95232762	0.07039463	0.896940866	-4.034967704
hsa-miR-374b-5p	MIMAT0004955	-1.275294094	2.377410529	2.032694677	0.060731146	0.896940866	-3.965756828
hsa-miR-99a-5p	MIMAT0000097	-1.323648149	2.242763143	1.40748185	0.180267056	0.903156384	-4.467748424
hsa-miR-146b-5p	MIMAT0002809	-1.447767179	4.649862887	2.735064384	0.015660448	0.896940866	-3.334939505
hsa-miR-379-5p	MIMAT0000733	-1.582922343	4.146801404	3.221400208	0.005892162	0.896940866	-2.896744671
hsa-miR-625-5p	MIMAT0003294	-1.595565631	4.465929105	2.084374718	0.055174638	0.896940866	-3.920742944
hsa-miR-487b-3p	MIMAT0003180	-1.6919782	4.224034812	2.870721521	0.011944371	0.896940866	-3.211635558
hsa-miR-494-3p	MIMAT0002816	-1.716485816	3.979351685	4.029572516	0.00115306	0.896940866	-2.215124493
