# Supplementary material for: A Plasma Extracellular Vesicle-Derived microRNA Signature as a Potential Biomarker for Subclinical Coronary Atherosclerosis
Source: Int J Mol Sci. 2025 Sep 7;26(17):8727. doi: 10.3390/ijms26178727 (PMC12428963; doi:10.3390/ijms26178727)
Supplement: Supplementary file 1 [file ijms-26-08727-s001.zip › Table_S2.pdf]

Supplementary Table S2. Differentially expressed miRNAs.
Agilent-074809 SurePrint G3 Mouse GE v2 8x60K Microarray

ID	logFC	t	B	P.Value	adj.P.Val
mmu-miR-34	-3.0539845	-18.378432	5.020947	5.36E-06	0.0017
mmu-miR-14	-2.8920834	-9.357886	1.502202	1.73E-04	0.01216
mmu-miR-14	-2.6140038	-20.749505	5.579939	2.84E-06	0.00163
mmu-miR-15	-2.5410994	-16.037612	4.357681	1.09E-05	0.00296
mmu-miR-14	-2.4125915	-19.110355	5.204372	4.37E-06	0.00166
mmu-miR-34	-2.273749	-22.192171	5.874863	2.00E-06	0.00163
mmu-miR-34	-2.2239707	-11.250421	2.505296	6.79E-05	0.00845
mmu-miR-21	-2.0960873	-25.865278	6.503049	8.94E-07	0.00163
mmu-miR-14	-2.0273505	-9.7895019	1.748941	1.38E-04	0.01047
mmu-miR-34	-2.0048027	-1.5616272	-6.087781	1.76E-01	0.4908
mmu-miR-34	-1.7975826	-12.185662	2.934196	4.51E-05	0.00722
mmu-miR-22	-1.5386411	-15.102716	4.055061	1.49E-05	0.00315
mmu-miR-22	-1.4078686	-7.1024021	-0.005403	6.76E-04	0.02625
mmu-miR-70	-1.1853255	-1.3067197	-6.398298	2.45E-01	0.56747
mmu-miR-22	-1.1462879	-11.148678	2.456209	7.11E-05	0.00845
mmu-miR-15	-1.1111188	-8.7378822	1.126509	2.43E-04	0.01494
mmu-miR-30	-1.1046569	-1.5237036	-6.135432	1.85E-01	0.50957
mmu-miR-69	-0.9978733	-1.6173808	-6.016966	1.64E-01	0.46695
mmu-miR-69	-0.9708947	-2.8935028	-4.312531	3.19E-02	0.19938
mmu-miR-48	-0.9480903	-1.5251468	-6.133627	1.85E-01	0.5095
mmu-miR-53	-0.9380652	-6.3508989	-0.607722	1.16E-03	0.03864
mmu-miR-18	-0.8861451	-4.6807841	-2.184281	4.70E-03	0.07142
mmu-miR-42	-0.8554927	-10.466202	2.11354	9.81E-05	0.00945
mmu-miR-34	-0.8476548	-6.5392899	-0.451178	1.01E-03	0.03548
mmu-miR-18	-0.7852641	-10.610636	2.188069	9.15E-05	0.00945
mmu-miR-12	-0.7721594	-4.2289249	-2.677015	7.29E-03	0.08752
mmu-miR-19	-0.7647974	-4.4440446	-2.438611	5.89E-03	0.07784
mmu-miR-32	-0.7607661	-3.1136492	-4.025615	2.45E-02	0.17712
mmu-miR-36	-0.7557754	-5.8548524	-1.039116	1.70E-03	0.04311
mmu-miR-31	-0.6951722	-5.126456	-1.727576	3.13E-03	0.0593
mmu-miR-37	-0.6944855	-4.5849855	-2.286195	5.15E-03	0.07359
mmu-miR-70	-0.6846694	-2.0387194	-5.461383	9.39E-02	0.35044
mmu-miR-12	-0.6839818	-3.807949	-3.164054	1.13E-02	0.1175
mmu-miR-72	-0.6506399	-1.8100818	-5.766466	1.27E-01	0.40597
mmu-miR-51	-0.6153035	-1.0148044	-6.716762	3.54E-01	0.67126
mmu-miR-32	-0.6086921	-7.2223728	0.085509	6.23E-04	0.02625
mmu-miR-39	-0.5838424	-4.2450351	-2.658917	7.17E-03	0.08752
mmu-miR-18	-0.5799649	-0.9187784	-6.810025	3.98E-01	0.70309
mmu-miR-69	-0.5654762	-1.4180945	-6.265625	2.12E-01	0.53549
mmu-miR-18	-0.5591484	-1.3773884	-6.314712	2.24E-01	0.54371
mmu-miR-29	-0.5406504	-10.197251	1.97174	1.12E-04	0.00955
mmu-miR-45	-0.5305105	-1.6254872	-6.006599	1.62E-01	0.46472

mmu-miR-67	-0.5292892	-5.4966867	-1.369049	2.28E-03	0.0487
mmu-miR-69	-0.5285229	-5.0031055	-1.851164	3.50E-03	0.06127
mmu-miR-93	-0.5230845	-3.0757375	-4.074604	2.56E-02	0.18192
mmu-miR-48	-0.5147474	-2.3284084	-5.069617	6.45E-02	0.28617
mmu-miR-30	-0.499654	-4.9746359	-1.87999	3.59E-03	0.06202
mmu-miR-30	-0.494834	-0.7291526	-6.973588	4.97E-01	0.75973
mmu-miR-22	-0.4876445	-3.7759199	-3.202225	1.17E-02	0.1197
mmu-miR-13	-0.4726115	-5.143236	-1.710926	3.09E-03	0.0593
mmu-miR-81	-0.4688822	-1.7698478	-5.819395	1.34E-01	0.41909
mmu-miR-31	-0.4547827	-1.4452625	-6.23251	2.05E-01	0.52679
mmu-miR-36	-0.4513936	-3.0566737	-4.099306	2.62E-02	0.18333
mmu-miR-35	-0.4214565	-5.1207093	-1.733287	3.15E-03	0.0593
mmu-miR-20	-0.416333	-6.0058636	-0.904742	1.51E-03	0.0413
mmu-miR-51	-0.4089573	-0.8382442	-6.883026	4.38E-01	0.72895
mmu-miR-28	-0.399653	-1.11728	-6.610484	3.12E-01	0.6297
mmu-miR-51	-0.3992051	-0.884543	-6.841665	4.15E-01	0.7159
mmu-miR-70	-0.3926652	-2.3858838	-4.991804	5.99E-02	0.28074
mmu-miR-69	-0.3810456	-0.6122528	-7.059021	5.66E-01	0.80183
mmu-let-7i-5	-0.3777047	-4.4775272	-2.402133	5.71E-03	0.07784
mmu-miR-49	-0.3672588	-2.0710536	-5.417819	9.00E-02	0.34382
mmu-miR-32	-0.3397917	-1.5042939	-6.159646	1.90E-01	0.51381
dmr_6	-0.3212873	-2.7205882	-4.541642	3.94E-02	0.2228
mmu-miR-70	-0.3168514	-2.7322655	-4.526079	3.88E-02	0.2228
mmu-miR-69	-0.3157651	-3.3198308	-3.762523	1.93E-02	0.15234
mmu-miR-70	-0.3145134	-3.8730102	-3.087002	1.05E-02	0.11296
mmu-miR-18	-0.3120396	-2.4763437	-4.869527	5.34E-02	0.2632
mmu-miR-71	-0.306478	-3.0526666	-4.104504	2.63E-02	0.18353
mmu-miR-64	-0.3032095	-0.4451712	-7.158173	6.74E-01	0.86547
mmu-miR-70	-0.3003524	-0.8663786	-6.858091	4.24E-01	0.71995
mmu-miR-19	-0.2936935	-1.1943788	-6.526401	2.83E-01	0.60098
mmu-miR-12	-0.2928593	-4.670177	-2.195498	4.75E-03	0.07142
mmu-miR-21	-0.2910998	-8.0517217	0.678451	3.66E-04	0.02045
mmu-miR-48	-0.2866744	-2.5294488	-4.797908	5.00E-02	0.25817
mmu-miR-10	-0.2861143	-1.3536177	-6.343066	2.31E-01	0.55391
mmu-miR-70	-0.2854427	-1.0328991	-6.698481	3.47E-01	0.6651
mmu-miR-39	-0.2787775	-1.2041178	-6.515548	2.80E-01	0.59763
mmu-miR-18	-0.2771781	-2.643847	-4.644205	4.33E-02	0.2359
mmu-miR-21	-0.2757188	-1.9149205	-5.627351	1.10E-01	0.37932
mmu-miR-7a	-0.2735338	-9.7974519	1.753378	1.37E-04	0.01047
mmu-miR-19	-0.2665793	-1.0519815	-6.678971	3.38E-01	0.65737
mmu-miR-34	-0.2613542	-2.834349	-4.390571	3.42E-02	0.20792
mmu-miR-12	-0.259235	-1.9853669	-5.53309	1.01E-01	0.36559
mmu-miR-13	-0.2548967	-3.3297006	-3.750076	1.91E-02	0.15128
dmr_285	-0.2505237	-3.4549774	-3.593292	1.66E-02	0.14259
mr_1	-0.24247	-2.0772049	-5.409524	8.93E-02	0.34263
mmu-miR-45	-0.2410399	-1.8113981	-5.76473	1.27E-01	0.40597

mmu-miR-14	-0.2380881	-1.9311683	-5.605659	1.08E-01	0.37605
mmu-miR-19	-0.2366645	-0.674266	-7.015262	5.28E-01	0.7786
mmu-miR-96	-0.236304	-3.4913496	-3.548199	1.59E-02	0.14007
mmu-miR-71	-0.2342479	-3.1885404	-3.929389	2.25E-02	0.16818
mmu-miR-72	-0.2304379	-1.1857006	-6.53603	2.86E-01	0.60192
mmu-miR-34	-0.2280713	-2.9454414	-4.244323	2.99E-02	0.19073
mmu-miR-18	-0.2245152	-4.1237819	-2.796105	8.10E-03	0.09473
dmr_3	-0.2199706	-1.8459283	-5.719081	1.21E-01	0.39607
mmu-miR-65	-0.2198825	-2.3281342	-5.069989	6.45E-02	0.28617
mmu-miR-72	-0.2197288	-5.0648889	-1.788997	3.31E-03	0.06061
dmr_31a	-0.2171696	-2.7869509	-4.453363	3.63E-02	0.21444
mmu-miR-69	-0.21708	-2.6256731	-4.668563	4.43E-02	0.23924
mmu-miR-37	-0.2142313	-0.8461588	-6.876074	4.34E-01	0.72895
mmu-miR-42	-0.2038111	-1.5660419	-6.082206	1.75E-01	0.48927
mmu-miR-18	-0.2016627	-3.2005245	-3.91406	2.21E-02	0.16651
mmu-miR-30	-0.2009895	-7.3404308	0.173647	5.76E-04	0.02548
mmu-miR-63	-0.2006669	-0.8335276	-6.887144	4.41E-01	0.72895
mmu-miR-77	-0.1982796	-0.9209743	-6.807966	3.97E-01	0.70309
mmu-miR-24	-0.1981389	-1.6856462	-5.929152	1.49E-01	0.44414
mmu-miR-64	-0.1898443	-4.1508912	-2.765238	7.88E-03	0.09315
mmu-miR-70	-0.1879899	-0.7805867	-6.932134	4.69E-01	0.73829
mmu-miR-13	-0.1844182	-4.2273953	-2.678735	7.30E-03	0.08752
mmu-miR-29	-0.1833452	-1.0802216	-6.649673	3.27E-01	0.64748
mmu-miR-12	-0.1827695	-2.9754671	-4.205033	2.89E-02	0.18992
mmu-miR-15	-0.1825818	-4.0501408	-2.880526	8.74E-03	0.09902
mmu-miR-14	-0.1807263	-3.4243759	-3.63138	1.71E-02	0.14412
mmu-miR-20	-0.1788221	-1.4034993	-6.2833	2.16E-01	0.53932
mmu-miR-20	-0.1781456	-0.898848	-6.828552	4.08E-01	0.71025
mmu-miR-51	-0.1774159	-3.4451721	-3.605481	1.68E-02	0.14263
mmu-miR-22	-0.1772297	-4.496383	-2.381665	5.60E-03	0.07721
mmu-miR-81	-0.1733575	-0.3869874	-7.185906	7.14E-01	0.88205
mmu-miR-72	-0.1733263	-2.4183339	-4.947906	5.75E-02	0.27503
mmu-miR-69	-0.1693733	-3.0111284	-4.158505	2.77E-02	0.18689
mmu-miR-19	-0.168462	-2.174902	-5.277512	7.86E-02	0.31556
mmu-miR-70	-0.1655084	-1.0513218	-6.679649	3.39E-01	0.65737
mmu-miR-81	-0.1651643	-1.4888314	-6.178847	1.94E-01	0.51559
mmu-miR-18	-0.1615471	-2.3903089	-4.985816	5.96E-02	0.27989
mmu-miR-51	-0.1579811	-1.1151434	-6.612765	3.13E-01	0.6297
mmu-miR-76	-0.1577401	-2.9902224	-4.185763	2.84E-02	0.1885
mmu-miR-69	-0.1573865	-3.4426628	-3.608603	1.68E-02	0.14263
mmu-miR-63	-0.1550393	-1.3075149	-6.39737	2.45E-01	0.56747
mmu-miR-70	-0.1490155	-1.2137805	-6.50473	2.76E-01	0.59633
mmu-miR-67	-0.1488695	-1.6977904	-5.913414	1.47E-01	0.43977
mmu-let-7f-1	-0.1418727	-4.4538772	-2.427881	5.84E-03	0.07784
mmu-miR-72	-0.141868	-4.1610912	-2.753653	7.80E-03	0.09277
mmu-miR-76	-0.1418549	-3.8044794	-3.168182	1.13E-02	0.1175

mmu-miR-36	-0.1372373	-6.0763798	-0.842923	1.43E-03	0.0413
mmu-miR-69	-0.1372205	-3.56029	-3.463265	1.47E-02	0.13283
mmu-miR-31	-0.1356043	-0.8211114	-6.897901	4.47E-01	0.72914
mmu-miR-31	-0.1336791	-1.4155272	-6.26874	2.13E-01	0.53586
mmu-miR-29	-0.1325892	-0.8259416	-6.893732	4.44E-01	0.72895
mmu-miR-30	-0.1304683	-1.7607523	-5.831319	1.35E-01	0.42276
mmu-miR-34	-0.1301877	-2.8195824	-4.41011	3.49E-02	0.20915
mmu-miR-34	-0.129981	-4.9341615	-1.921169	3.72E-03	0.06375
mmu-miR-13	-0.1296448	-6.0494687	-0.866446	1.46E-03	0.0413
mmu-miR-64	-0.1287726	-0.3175253	-7.214189	7.63E-01	0.90649
mmu-miR-30	-0.1282915	-1.7050605	-5.903977	1.46E-01	0.43633
mmu-miR-72	-0.127576	-2.2098349	-5.230226	7.52E-02	0.30918
mmu-miR-34	-0.1256795	-4.2912714	-2.607197	6.85E-03	0.08626
mmu-miR-14	-0.1237499	-0.2706697	-7.230231	7.97E-01	0.92083
mmu-miR-14	-0.1209593	-2.603387	-4.698465	4.55E-02	0.24458
mmu-miR-16	-0.1188158	-1.9471705	-5.584266	1.06E-01	0.37363
mmu-miR-34	-0.1186966	-5.0011432	-1.853147	3.50E-03	0.06127
mmu-miR-19	-0.1178028	-0.5019422	-7.127662	6.36E-01	0.83933
mmu-miR-70	-0.1166127	-2.3382187	-5.056331	6.37E-02	0.28573
mmu-miR-70	-0.1154045	-4.2251119	-2.681304	7.32E-03	0.08752
mmu-miR-20	-0.1150764	-1.1241548	-6.603123	3.09E-01	0.62619
mmu-miR-12	-0.1149499	-1.7281837	-5.873887	1.41E-01	0.43161
mmu-miR-76	-0.111131	-3.667157	-3.333012	1.31E-02	0.12527
mmu-miR-69	-0.1108296	-4.5978375	-2.272444	5.08E-03	0.07325
mmu-miR-34	-0.110622	-5.8048192	-1.084245	1.77E-03	0.04371
mmu-miR-37	-0.1101231	-0.382552	-7.18787	7.17E-01	0.88229
mmu-miR-64	-0.1095275	-2.4680028	-4.880788	5.40E-02	0.26462
mmu-miR-25	-0.108473	-0.7918883	-6.922724	4.62E-01	0.73321
mmu-miR-62	-0.1080623	-1.8628186	-5.696685	1.18E-01	0.39357
mmu-miR-37	-0.1060975	-0.4715371	-7.144421	6.56E-01	0.85271
mmu-miR-30	-0.1059786	-1.3882368	-6.301694	2.21E-01	0.5409
mmu-miR-30	-0.1049224	-3.023632	-4.142228	2.73E-02	0.18559
mmu-miR-12	-0.1045597	-1.3560059	-6.340228	2.30E-01	0.55391
mmu-miR-68	-0.1034165	-5.8868773	-1.01039	1.66E-03	0.04311
mmu-miR-21	-0.1022717	-0.7116737	-6.987152	5.07E-01	0.7663
mmu-miR-70	-0.1021561	-3.2033811	-3.910409	2.21E-02	0.16651
mmu-miR-65	-0.1009666	-0.4926232	-7.1329	6.42E-01	0.84191
mmu-miR-24	-0.1004992	-3.5823143	-3.436281	1.44E-02	0.13129
mmu-miR-63	-0.0995758	-0.5041687	-7.126398	6.34E-01	0.83905
mmu-miR-37	-0.0988678	-0.9156872	-6.812918	4.00E-01	0.70381
mmu-miR-38	-0.0985549	-1.4156392	-6.268605	2.13E-01	0.53586
mmu-miR-53	-0.0974468	-3.372754	-3.69594	1.82E-02	0.14712
mmu-miR-31	-0.0970644	-0.5971951	-7.069097	5.75E-01	0.80719
mmu-miR-70	-0.0969604	-0.2383735	-7.23984	8.21E-01	0.92965
mmu-miR-67	-0.0907805	-2.6636235	-4.617729	4.22E-02	0.23352
mmu-miR-31	-0.0890895	-2.4853288	-4.857399	5.28E-02	0.2632

mmu-miR-19	-0.0888511	-2.3146553	-5.088245	6.57E-02	0.28795
mmu-miR-48	-0.0888041	-1.1364929	-6.589845	3.05E-01	0.61961
mmu-miR-29	-0.0884579	-2.2070872	-5.233947	7.54E-02	0.30918
mmu-miR-69	-0.0880277	-0.4353768	-7.163095	6.80E-01	0.86795
mmu-miR-76	-0.0871058	-2.3597876	-5.027126	6.20E-02	0.28131
mmu-miR-99	-0.0864443	-1.9029329	-5.643335	1.12E-01	0.38258
mmu-miR-70	-0.086076	-3.4712742	-3.573064	1.63E-02	0.14129
mmu-miR-69	-0.0858018	-2.3662244	-5.018412	6.15E-02	0.28131
mmu-miR-71	-0.0839063	-2.2120499	-5.227227	7.49E-02	0.30918
mmu-miR-30	-0.0817913	-2.6885866	-4.584352	4.09E-02	0.22884
mmu-miR-68	-0.0793033	-0.9180273	-6.810729	3.99E-01	0.70309
mmu-miR-76	-0.0778311	-0.7827422	-6.930347	4.67E-01	0.73768
mmu-miR-46	-0.0768151	-1.7877413	-5.795891	1.31E-01	0.41272
mmu-miR-50	-0.0766572	-2.4414045	-4.916719	5.58E-02	0.27153
mmu-miR-81	-0.0764086	-0.6028512	-7.065338	5.72E-01	0.80349
mmu-miR-35	-0.0745088	-4.8561034	-2.001241	3.99E-03	0.06629
mmu-miR-72	-0.0738657	-4.9983767	-1.855944	3.51E-03	0.06127
mmu-miR-42	-0.0735837	-1.3870844	-6.303079	2.21E-01	0.54102
mmu-miR-21	-0.0732551	-5.4394483	-1.423289	2.39E-03	0.04998
mmu-miR-70	-0.0730624	-3.4438328	-3.607147	1.68E-02	0.14263
mmu-miR-98	-0.0723004	-3.5854688	-3.432422	1.43E-02	0.13129
mmu-miR-76	-0.0716091	-2.0051762	-5.506494	9.81E-02	0.36043
mmu-miR-43	-0.071123	-2.7478256	-4.50536	3.81E-02	0.22038
mmu-miR-19	-0.0709114	-4.3899846	-2.497863	6.21E-03	0.08094
mmu-miR-18	-0.0701377	-4.0993412	-2.824031	8.31E-03	0.09626
mmu-miR-69	-0.0698672	-3.9273502	-3.023146	9.93E-03	0.10915
mmu-miR-70	-0.0696863	-3.6501582	-3.353616	1.34E-02	0.12634
mmu-miR-69	-0.0684271	-4.6367807	-2.230925	4.90E-03	0.07224
mmu-miR-20	-0.0677235	-2.6349991	-4.656061	4.38E-02	0.23783
mmu-miR-51	-0.0677119	-3.0063332	-4.164753	2.78E-02	0.18689
mmu-miR-19	-0.0675754	-1.3743445	-6.318356	2.25E-01	0.54414
mmu-miR-67	-0.0669424	-3.4797946	-3.562503	1.61E-02	0.14108
mmu-miR-30	-0.0666675	-2.5454251	-4.776392	4.90E-02	0.25511
mmu-miR-97	-0.0659833	-2.8341573	-4.390825	3.42E-02	0.20792
mmu-miR-70	-0.0658527	-4.5036184	-2.373825	5.56E-03	0.07721
mmu-miR-72	-0.0653367	-0.6797206	-7.011242	5.25E-01	0.77509
mmu-miR-37	-0.0643444	-1.9553692	-5.573294	1.05E-01	0.37315
mmu-miR-42	-0.0640385	-2.3125866	-5.091047	6.58E-02	0.28795
mmu-miR-56	-0.0640177	-1.9287264	-5.608921	1.08E-01	0.37657
mmu-miR-70	-0.0639478	-3.828528	-3.139612	1.10E-02	0.11643
mmu-miR-20	-0.063739	-3.0041446	-4.167605	2.79E-02	0.18689
mmu-miR-12	-0.0628091	-1.1696035	-6.553782	2.92E-01	0.60718
mmu-miR-11	-0.0627721	-4.5764984	-2.29529	5.19E-03	0.07364
mmu-miR-81	-0.0626163	-1.1828368	-6.539199	2.87E-01	0.60245
mmu-miR-19	-0.0625771	-1.7609283	-5.831089	1.35E-01	0.42276
mmu-miR-30	-0.0624618	-3.9151222	-3.037476	1.01E-02	0.10992

mmu-miR-20	-0.0619684	-2.060554	-5.431973	9.13E-02	0.34583
mmu-miR-56	-0.0615492	-2.1255768	-5.344216	8.39E-02	0.32919
mmu-miR-50	-0.0615053	-3.6598505	-3.341863	1.32E-02	0.12564
mmu-miR-81	-0.0610827	-3.6828377	-3.314045	1.29E-02	0.12417
mmu-miR-31	-0.0610507	-3.7718577	-3.207077	1.17E-02	0.1197
mmu-miR-33	-0.0604513	-2.5649074	-4.750175	4.78E-02	0.25219
mmu-miR-87	-0.0601781	-2.6011988	-4.701403	4.56E-02	0.24458
mmu-miR-56	-0.0597492	-3.4919924	-3.547404	1.59E-02	0.14007
mmu-miR-19	-0.0596525	-0.3804712	-7.188784	7.18E-01	0.88325
mmu-miR-19	-0.0596472	-2.7846247	-4.456451	3.64E-02	0.21444
mmu-miR-70	-0.0593244	-0.3144696	-7.21531	7.65E-01	0.90735
mmu-miR-69	-0.0590463	-1.6766099	-5.94084	1.51E-01	0.44731
mmu-miR-69	-0.0583231	-2.4030107	-4.968631	5.86E-02	0.27879
mmu-miR-63	-0.0580434	-0.9855372	-6.745868	3.67E-01	0.68107
mmu-miR-50	-0.0572618	-2.2801052	-5.135048	6.86E-02	0.29668
mmu-miR-48	-0.0569765	-2.8083443	-4.424994	3.53E-02	0.21138
mmu-miR-70	-0.0560642	-1.7205591	-5.883821	1.43E-01	0.43296
mmu-miR-92	-0.05606	-2.4511764	-4.903515	5.51E-02	0.26895
mmu-miR-69	-0.0557813	-2.1396287	-5.325222	8.23E-02	0.32538
mmu-miR-72	-0.0557364	-2.540159	-4.783483	4.93E-02	0.2561
mmu-miR-12	-0.0556834	-1.7256302	-5.877215	1.42E-01	0.43161
mmu-miR-56	-0.0556091	-0.8121383	-6.905598	4.52E-01	0.73138
mmu-miR-30	-0.0554353	-1.2222939	-6.495159	2.73E-01	0.59462
mmu-miR-19	-0.0546653	-1.9616799	-5.564843	1.04E-01	0.37133
mmu-miR-70	-0.0545966	-1.9336423	-5.602354	1.08E-01	0.37552
mmu-miR-18	-0.0535765	-2.8322661	-4.393326	3.43E-02	0.20792
mmu-miR-50	-0.053416	-2.1034567	-5.374096	8.63E-02	0.335
mmu-miR-31	-0.0531584	-3.6912251	-3.303915	1.28E-02	0.12417
mmu-miR-87	-0.0530832	-3.1424306	-3.988547	2.37E-02	0.17473
mmu-miR-19	-0.0527377	-2.4799545	-4.864652	5.32E-02	0.2632
mmu-miR-65	-0.0527019	-2.857381	-4.360142	3.33E-02	0.20694
mmu-miR-69	-0.0526514	-1.8476487	-5.716802	1.21E-01	0.39586
mmu-miR-71	-0.0524739	-2.6563027	-4.627526	4.26E-02	0.23429
NC2_000792	-0.0522748	-1.5123589	-6.149599	1.88E-01	0.51079
mmu-miR-76	-0.051973	-0.3371752	-7.206729	7.49E-01	0.89855
mmu-miR-69	-0.0519668	-2.37131	-5.011528	6.11E-02	0.28131
mmu-miR-56	-0.0517198	-1.3916816	-6.297551	2.20E-01	0.5409
mmu-miR-56	-0.0514473	-2.645953	-4.641384	4.32E-02	0.2359
mmu-miR-70	-0.0510818	-2.2093475	-5.230886	7.52E-02	0.30918
mmu-miR-18	-0.051067	-3.6834524	-3.313303	1.29E-02	0.12417
mmu-miR-70	-0.0508942	-2.9502624	-4.238008	2.98E-02	0.1906
mmu-miR-69	-0.0507659	-2.3554877	-5.032948	6.23E-02	0.28155
mmu-miR-50	-0.0507614	-1.8031253	-5.775638	1.28E-01	0.40782
mmu-miR-62	-0.0505953	-3.2805371	-3.812215	2.02E-02	0.15677
mmu-miR-12	-0.0504786	-2.5226172	-4.807113	5.04E-02	0.25829
mmu-miR-12	-0.0498157	-1.3494836	-6.347974	2.32E-01	0.55448

mmu-miR-48	-0.0497173	-1.6150193	-6.019983	1.64E-01	0.4677
mmu-miR-12	-0.0494728	-1.840349	-5.72647	1.22E-01	0.39831
mmu-miR-70	-0.0490283	-1.8931349	-5.656386	1.14E-01	0.38343
mmu-miR-87	-0.0489842	-2.4915965	-4.848942	5.24E-02	0.2632
mmu-miR-69	-0.0485143	-1.8121836	-5.763694	1.26E-01	0.40597
mmu-miR-51	-0.048445	-3.6825811	-3.314355	1.29E-02	0.12417
mmu-miR-70	-0.04842	-0.9460851	-6.784167	3.85E-01	0.69435
mmu-miR-72	-0.0483836	-2.9567841	-4.229468	2.95E-02	0.1906
mmu-miR-32	-0.0481125	-0.6286351	-7.047813	5.56E-01	0.79671
mmu-miR-76	-0.0480275	-2.5719356	-4.740723	4.74E-02	0.25219
mmu-miR-71	-0.047923	-3.4214185	-3.635069	1.72E-02	0.14412
mmu-miR-21	-0.0476969	-2.1247407	-5.345346	8.39E-02	0.32919
mmu-miR-35	-0.0469839	-1.906563	-5.638497	1.12E-01	0.38145
mmu-miR-69	-0.0469419	-1.9774998	-5.543642	1.02E-01	0.36798
mmu-miR-21	-0.0465354	-1.795013	-5.786323	1.29E-01	0.41015
mmu-miR-68	-0.04651	-1.8511136	-5.71221	1.20E-01	0.39508
mmu-miR-34	-0.0464808	-3.6799576	-3.317526	1.29E-02	0.12417
mmu-miR-70	-0.0464578	-1.8249384	-5.746852	1.24E-01	0.40233
mmu-miR-46	-0.0456304	-1.9605279	-5.566386	1.04E-01	0.37133
mmu-miR-30	-0.0456262	-2.1507347	-5.310206	8.11E-02	0.3229
mmu-miR-70	-0.0454984	-2.3606003	-5.026026	6.19E-02	0.28131
mmu-miR-63	-0.0454701	-1.9484494	-5.582554	1.06E-01	0.37363
mmu-miR-41	-0.0454333	-1.243972	-6.47062	2.66E-01	0.58563
mmu-miR-69	-0.0453305	-2.781283	-4.460887	3.65E-02	0.21444
mmu-miR-69	-0.0452569	-1.4394328	-6.239639	2.06E-01	0.52923
mmu-miR-67	-0.0447637	-1.7889687	-5.794277	1.30E-01	0.41272
mmu-miR-7b	-0.0446541	-1.053038	-6.677884	3.38E-01	0.65737
mmu-miR-40	-0.0446482	-1.1433217	-6.582458	3.02E-01	0.61562
mmu-miR-14	-0.0445701	-2.1250392	-5.344942	8.39E-02	0.32919
mmu-miR-46	-0.0444618	-3.1008357	-4.042152	2.49E-02	0.17795
mmu-miR-51	-0.0443685	-2.5143817	-4.818213	5.09E-02	0.25959
mmu-miR-30	-0.0443588	-1.4897428	-6.177718	1.93E-01	0.51559
mmu-miR-29	-0.0442266	-2.1799155	-5.270728	7.81E-02	0.31448
mmu-miR-15	-0.0440595	-1.8768672	-5.678026	1.16E-01	0.38965
mmu-miR-66	-0.0434234	-2.3349857	-5.06071	6.40E-02	0.28617
mmu-miR-30	-0.0433751	-2.3138183	-5.089379	6.57E-02	0.28795
mmu-miR-70	-0.0433272	-1.5348883	-6.121424	1.82E-01	0.50401
mmu-miR-76	-0.0433263	-2.841432	-4.381208	3.39E-02	0.20759
mmu-miR-69	-0.0433169	-1.6241546	-6.008305	1.62E-01	0.46472
mmu-miR-59	-0.0432684	-1.8368536	-5.731096	1.22E-01	0.39878
mmu-miR-65	-0.0427176	-0.8387474	-6.882585	4.38E-01	0.72895
mmu-miR-74	-0.0426016	-3.3431456	-3.733142	1.88E-02	0.15023
mmu-miR-69	-0.0424283	-1.3178542	-6.385273	2.42E-01	0.56552
mmu-miR-72	-0.0423809	-0.9402063	-6.78978	3.88E-01	0.69625
mmu-miR-70	-0.0422762	-2.0738615	-5.414033	8.97E-02	0.34329
mmu-miR-18	-0.0418798	-1.6545595	-5.969282	1.56E-01	0.45454

mmu-miR-70	-0.0416206	-2.2289958	-5.204279	7.33E-02	0.30842
mmu-miR-51	-0.0415713	-1.2769066	-6.432894	2.55E-01	0.57422
mmu-miR-71	-0.041534	-0.553047	-7.097366	6.03E-01	0.82224
mmu-miR-54	-0.041233	-2.0409699	-5.458354	9.36E-02	0.35044
mmu-miR-34	-0.0409393	-1.9421745	-5.590948	1.07E-01	0.37368
mmu-miR-21	-0.0409004	-1.389878	-6.299721	2.20E-01	0.5409
mmu-miR-51	-0.040851	-2.5674919	-4.746699	4.76E-02	0.25219
mmu-miR-69	-0.040709	-2.2928883	-5.117731	6.75E-02	0.29318
mmu-miR-62	-0.040625	-2.03472	-5.466767	9.44E-02	0.35128
mmu-miR-70	-0.040493	-2.0383311	-5.461906	9.40E-02	0.35044
mmu-miR-69	-0.0404631	-1.9461657	-5.58561	1.06E-01	0.37363
mmu-miR-65	-0.0404235	-1.1865293	-6.535112	2.86E-01	0.60192
mmu-miR-76	-0.0403285	-2.3901776	-4.985994	5.96E-02	0.27989
mmu-miR-49	-0.0402606	-1.8615901	-5.698316	1.19E-01	0.39357
mmu-miR-69	-0.0402049	-1.176962	-6.545685	2.89E-01	0.60628
mmu-miR-19	-0.0400551	-1.799362	-5.780596	1.29E-01	0.40917
mmu-miR-71	-0.0397608	-2.2767707	-5.139565	6.89E-02	0.29668
mmu-miR-69	-0.0397303	-2.2202897	-5.216069	7.41E-02	0.30918
mmu-miR-70	-0.0395241	-1.3289981	-6.372181	2.38E-01	0.5625
mmu-miR-75	-0.0391998	-2.7815804	-4.460492	3.65E-02	0.21444
mmu-miR-69	-0.0391446	-1.3028555	-6.402806	2.46E-01	0.56886
mmu-miR-18	-0.0391025	-1.8127825	-5.762904	1.26E-01	0.40597
mmu-miR-20	-0.0390439	-0.6255602	-7.049936	5.58E-01	0.79681
mmu-miR-69	-0.0390219	-1.9250364	-5.613849	1.09E-01	0.37714
mmu-miR-11	-0.0388646	-2.2383108	-5.191662	7.24E-02	0.30618
mmu-miR-69	-0.0388129	-0.2740034	-7.229172	7.94E-01	0.92026
mmu-miR-34	-0.0386724	-2.6734454	-4.60459	4.17E-02	0.23136
mmu-miR-54	-0.0384479	-2.1515376	-5.30912	8.11E-02	0.3229
mmu-miR-57	-0.0382812	-1.7253596	-5.877568	1.42E-01	0.43161
mmu-miR-51	-0.0382658	-0.7621593	-6.947246	4.79E-01	0.74668
mmu-miR-45	-0.0382192	-2.1791733	-5.271732	7.82E-02	0.31448
mmu-miR-18	-0.0381718	-1.0953774	-6.633746	3.21E-01	0.63933
mmu-miR-19	-0.0380762	-1.7510707	-5.843995	1.37E-01	0.42462
mmu-miR-63	-0.0379634	-1.4088558	-6.276823	2.15E-01	0.53839
mmu-miR-12	-0.0379249	-0.1515852	-7.259715	8.85E-01	0.95745
mmu-miR-18	-0.0378266	-1.3180446	-6.38505	2.42E-01	0.56552
mmu-miR-19	-0.0377102	-1.7463251	-5.850202	1.38E-01	0.42529
mmu-miR-46	-0.0376969	-1.5148972	-6.146433	1.87E-01	0.51079
mmu-miR-35	-0.0374964	-1.2423086	-6.472511	2.66E-01	0.58563
mmu-miR-70	-0.0374215	-1.3883461	-6.301563	2.21E-01	0.5409
NC1_000001	-0.0372671	-1.8290823	-5.741375	1.24E-01	0.40105
mmu-miR-74	-0.0372382	-1.3565821	-6.339543	2.30E-01	0.55391
mmu-miR-70	-0.0371326	-0.8475651	-6.874834	4.33E-01	0.72895
mmu-miR-14	-0.0371287	-1.1664168	-6.55728	2.93E-01	0.60784
mmu-miR-39	-0.0371285	-1.4283936	-6.253104	2.09E-01	0.5333
mmu-miR-76	-0.0370654	-1.5952232	-6.045213	1.68E-01	0.47718

mmu-miR-69	-0.0369997	-0.9933698	-6.738135	3.64E-01	0.67898
mmu-miR-70	-0.0369137	-0.113109	-7.265721	9.14E-01	0.96744
mmu-miR-46	-0.0368011	-2.9714842	-4.210238	2.90E-02	0.18992
mmu-miR-88	-0.0367899	-1.5196744	-6.140468	1.86E-01	0.51021
mmu-miR-69	-0.0366868	-1.9790301	-5.54159	1.02E-01	0.36794
mmu-miR-76	-0.0366695	-1.1594076	-6.564953	2.96E-01	0.61062
mmu-miR-30	-0.0366202	-1.0498519	-6.68116	3.39E-01	0.65737
mmu-miR-66	-0.036517	-1.6233063	-6.00939	1.62E-01	0.46472
mmu-miR-48	-0.0364359	-0.0952178	-7.267921	9.28E-01	0.96891
mmu-miR-76	-0.0364038	-0.9502913	-6.780136	3.83E-01	0.69435
NC2_000921	-0.0363944	-1.9401624	-5.593638	1.07E-01	0.37368
mmu-let-7b-:	-0.036335	-1.3421535	-6.356656	2.34E-01	0.55766
mmu-miR-87	-0.0363046	-2.2478103	-5.178795	7.16E-02	0.30379
mmu-miR-32	-0.0360141	-1.5062834	-6.157169	1.89E-01	0.51381
mmu-miR-45	-0.0357458	-0.1874994	-7.252549	8.58E-01	0.94358
mmu-miR-56	-0.0357282	-2.2165732	-5.221102	7.45E-02	0.30918
mmu-miR-69	-0.035676	-1.9010236	-5.645879	1.13E-01	0.38286
mmu-miR-19	-0.0355418	-1.9989496	-5.514858	9.89E-02	0.3619
mmu-miR-35	-0.0353575	-1.4045244	-6.282062	2.16E-01	0.53932
mmu-miR-49	-0.0353237	-1.4939565	-6.172492	1.92E-01	0.51559
mmu-miR-93	-0.0351448	-0.993743	-6.737766	3.64E-01	0.67898
mmu-miR-69	-0.035104	-1.0861832	-6.643425	3.24E-01	0.64337
mmu-miR-19	-0.0350272	-1.4650435	-6.208229	2.00E-01	0.51936
mmu-miR-55	-0.0348051	-0.8647995	-6.859507	4.25E-01	0.72035
mmu-miR-34	-0.0348051	-0.4213931	-7.169945	6.90E-01	0.87265
mmu-miR-11	-0.0347604	-1.1112236	-6.616945	3.14E-01	0.63146
mmu-miR-63	-0.03456	-1.9385783	-5.595756	1.07E-01	0.37378
mmu-miR-13	-0.0344635	-1.2547247	-6.458361	2.62E-01	0.58318
mmu-miR-19	-0.0343103	-0.9759114	-6.755313	3.72E-01	0.68552
mmu-miR-20	-0.0343075	-0.4389783	-7.161297	6.78E-01	0.86667
mmu-miR-69	-0.0342447	-2.2595672	-5.16287	7.05E-02	0.29988
mmu-miR-63	-0.0341524	-1.3199877	-6.382771	2.41E-01	0.56536
mmu-miR-81	-0.0339567	-1.8592458	-5.701426	1.19E-01	0.39357
mmu-miR-69	-0.0338247	-0.9907052	-6.740771	3.65E-01	0.67898
mmu-miR-69	-0.0337716	-1.9116203	-5.631753	1.11E-01	0.38028
mmu-miR-76	-0.0337703	-2.394191	-4.980563	5.93E-02	0.27989
mmu-miR-69	-0.0337466	-1.6841644	-5.93107	1.50E-01	0.44432
mmu-miR-70	-0.0337052	-1.6102294	-6.026097	1.65E-01	0.46995
mmu-miR-87	-0.0336061	-1.4188341	-6.264728	2.12E-01	0.53549
mmu-miR-19	-0.0334635	-2.4684751	-4.88015	5.40E-02	0.26462
mmu-miR-46	-0.0333981	-1.8739914	-5.681848	1.17E-01	0.39044
mmu-miR-69	-0.0333286	-1.4924098	-6.174411	1.93E-01	0.51559
mmu-miR-65	-0.0333265	-1.1872412	-6.534324	2.86E-01	0.60192
mmu-miR-76	-0.0331612	-1.1889256	-6.532456	2.85E-01	0.60192
mmu-miR-30	-0.0329552	-2.0892656	-5.393252	8.79E-02	0.33918
mmu-miR-51	-0.0328396	-0.2448956	-7.237995	8.16E-01	0.92676

mmu-miR-70	-0.0328137	-1.2282252	-6.488469	2.71E-01	0.59286
mmu-miR-11	-0.0328127	-2.5024965	-4.834238	5.17E-02	0.26072
mmu-miR-49	-0.0328053	-0.3881814	-7.185373	7.13E-01	0.88176
mmu-miR-50	-0.032733	-1.8504513	-5.713088	1.20E-01	0.39508
mmu-miR-70	-0.0327108	-2.5595511	-4.757381	4.81E-02	0.25219
mmu-miR-12	-0.0323489	-0.3095819	-7.217082	7.69E-01	0.90921
mmu-miR-68	-0.0323439	-1.9970074	-5.517466	9.92E-02	0.36213
mmu-miR-81	-0.0323352	-1.4673877	-6.205342	1.99E-01	0.51936
mmu-miR-11	-0.0323148	-0.6861562	-7.006464	5.22E-01	0.77235
mmu-miR-45	-0.0321886	-0.9979605	-6.733584	3.62E-01	0.67898
mmu-miR-71	-0.0321866	-1.6438608	-5.98304	1.58E-01	0.45923
mmu-miR-70	-0.0321761	-0.9688265	-6.762224	3.75E-01	0.68944
mmu-miR-12	-0.0321132	-1.9188086	-5.622163	1.10E-01	0.37875
mmu-miR-70	-0.0320091	-1.7542684	-5.83981	1.37E-01	0.4245
mmu-miR-35	-0.0319968	-1.3830878	-6.307879	2.22E-01	0.54313
mmu-miR-56	-0.0319492	-1.4871735	-6.180901	1.94E-01	0.51559
mmu-miR-63	-0.0319256	-1.7499722	-5.845432	1.37E-01	0.42462
mmu-miR-50	-0.0319011	-1.8573127	-5.70399	1.19E-01	0.39357
mmu-miR-19	-0.0318883	-1.3786589	-6.31319	2.23E-01	0.54371
mmu-miR-12	-0.0317117	-1.6955334	-5.916342	1.48E-01	0.43977
mmu-miR-49	-0.0316376	-1.5040369	-6.159966	1.90E-01	0.51381
mmu-miR-69	-0.0316199	-1.5746625	-6.071303	1.73E-01	0.48663
NegativeCon	-0.0315611	-2.517055	-4.81461	5.07E-02	0.25941
mmu-miR-69	-0.0315233	-1.2040727	-6.515598	2.80E-01	0.59763
mmu-miR-30	-0.0313377	-0.9083064	-6.819796	4.03E-01	0.70475
mmu-miR-70	-0.0312829	-1.2433633	-6.471312	2.66E-01	0.58563
mmu-miR-69	-0.0312311	-1.6043531	-6.03359	1.66E-01	0.47219
mmu-miR-31	-0.0310411	-1.6597491	-5.962599	1.55E-01	0.45421
mmu-miR-69	-0.0310278	-2.3110513	-5.093127	6.60E-02	0.28795
mmu-miR-29	-0.0308634	-1.6412251	-5.986424	1.58E-01	0.46012
mmu-miR-13	-0.0308253	-1.3945984	-6.294039	2.19E-01	0.5409
mmu-miR-70	-0.0307762	-0.9174479	-6.811271	3.99E-01	0.70309
mmu-miR-71	-0.0307242	-0.9586002	-6.772136	3.79E-01	0.69262
mmu-miR-70	-0.0307235	-1.8159452	-5.75873	1.26E-01	0.40575
mmu-miR-63	-0.0307051	-1.3890266	-6.300745	2.20E-01	0.5409
mmu-miR-76	-0.0305495	-2.4633042	-4.887133	5.43E-02	0.26552
mmu-miR-69	-0.0305335	-0.2349572	-7.240787	8.23E-01	0.93017
miRNABright	-0.0305131	-0.7925144	-6.922199	4.62E-01	0.73321
mmu-miR-34	-0.0305012	-1.2802849	-6.428995	2.54E-01	0.57311
mmu-miR-20	-0.0304407	-2.1864645	-5.261864	7.75E-02	0.3139
mmu-miR-57	-0.0303758	-1.7976665	-5.782829	1.29E-01	0.4094
mmu-miR-38	-0.0303539	-1.7469932	-5.849328	1.38E-01	0.42529
mmu-miR-16	-0.0303126	-0.5793287	-7.080767	5.86E-01	0.81497
mmu-miR-76	-0.0302421	-1.1988298	-6.521447	2.82E-01	0.59826
mmu-miR-70	-0.0298327	-1.5586842	-6.091495	1.77E-01	0.49113
mmu-miR-69	-0.029753	-0.7959846	-6.919287	4.60E-01	0.73321

mmu-miR-54	-0.0294765	-1.2449104	-6.469553	2.65E-01	0.58563
mmu-miR-30	-0.0294467	-0.9222258	-6.806791	3.97E-01	0.70309
mmu-miR-44	-0.0293701	-1.3278279	-6.373559	2.39E-01	0.5625
mmu-miR-26	-0.0293203	-1.4322602	-6.248393	2.08E-01	0.53133
mmu-miR-39	-0.0292755	-1.116768	-6.611031	3.12E-01	0.6297
mmu-miR-10	-0.0292401	-0.7096424	-6.98871	5.08E-01	0.7663
mmu-miR-80	-0.0289339	-0.9893587	-6.742101	3.66E-01	0.67898
mmu-miR-88	-0.0287355	-1.4569111	-6.218228	2.02E-01	0.52293
mmu-miR-30	-0.0286417	-1.9171118	-5.624428	1.10E-01	0.37891
mmu-miR-19	-0.0285744	-1.4184908	-6.265144	2.12E-01	0.53549
mmu-miR-37	-0.0285116	-1.125785	-6.601374	3.09E-01	0.62599
mmu-miR-70	-0.0284372	-1.5596953	-6.090219	1.76E-01	0.49113
mmu-miR-33	-0.0284326	-1.4198753	-6.263463	2.12E-01	0.53549
mmu-miR-70	-0.0283648	-1.0148242	-6.716742	3.54E-01	0.67126
mmu-miR-69	-0.028363	-0.9124886	-6.815904	4.01E-01	0.70456
mmu-miR-76	-0.0283572	-0.5064452	-7.125099	6.33E-01	0.83834
mmu-miR-76	-0.0282931	-2.2041715	-5.237894	7.57E-02	0.30968
mmu-miR-32	-0.028218	-1.2938638	-6.413267	2.49E-01	0.57063
mmu-miR-70	-0.0281745	-1.2992356	-6.407022	2.48E-01	0.56943
mmu-miR-67	-0.0280727	-1.6543814	-5.969511	1.56E-01	0.45454
mmu-miR-19	-0.0280549	-0.148844	-7.2602	8.87E-01	0.95745
mmu-miR-30	-0.028038	-0.9599749	-6.770808	3.79E-01	0.69262
mmu-miR-72	-0.0280286	-0.8304952	-6.889783	4.42E-01	0.72895
mmu-miR-35	-0.028026	-1.0022275	-6.729341	3.60E-01	0.67765
mmu-miR-70	-0.0280237	-1.2598026	-6.452552	2.60E-01	0.58144
mmu-miR-76	-0.0279085	-1.7729587	-5.815313	1.33E-01	0.41807
mmu-miR-12	-0.02779	-1.0009022	-6.73066	3.60E-01	0.67807
mmu-miR-69	-0.0277461	-0.7779482	-6.934315	4.70E-01	0.73931
mmu-miR-19	-0.0277206	-1.3010504	-6.404909	2.47E-01	0.56943
mmu-miR-14	-0.0276376	-0.7148804	-6.984683	5.05E-01	0.76532
mmu-miR-87	-0.0276129	-1.1154291	-6.61246	3.13E-01	0.6297
mmu-miR-65	-0.0275972	-2.0841368	-5.400173	8.85E-02	0.34077
mmu-miR-33	-0.0275782	-1.4919541	-6.174976	1.93E-01	0.51559
mmu-miR-63	-0.0274471	-1.3056972	-6.399492	2.46E-01	0.56747
mmu-let-7c-'	-0.0272254	-0.637759	-7.04146	5.50E-01	0.79401
mmu-miR-44	-0.0270526	-1.725589	-5.877269	1.42E-01	0.43161
mmu-miR-81	-0.0269953	-1.4546266	-6.221033	2.02E-01	0.52307
mmu-miR-35	-0.0269495	-1.347603	-6.350203	2.33E-01	0.55513
mmu-miR-70	-0.0267485	-1.2072894	-6.512003	2.79E-01	0.59763
mmu-miR-70	-0.0266365	-1.1509597	-6.574164	2.99E-01	0.61368
mmu-miR-11	-0.0265058	-1.0360438	-6.695282	3.45E-01	0.66453
mmu-miR-34	-0.0264643	-1.2199098	-6.497843	2.74E-01	0.59575
mmu-miR-34	-0.0264457	-1.1047366	-6.623841	3.17E-01	0.63526
mmu-miR-63	-0.026338	-0.8886703	-6.837898	4.13E-01	0.7151
mmu-miR-56	-0.0263135	-1.6755238	-5.942244	1.51E-01	0.44731
mmu-miR-44	-0.0260894	-0.6550131	-7.029232	5.40E-01	0.78734

mmu-miR-88	-0.026061	-0.9345704	-6.795137	3.91E-01	0.6985
mmu-miR-61	-0.0260266	-1.3737702	-6.319043	2.25E-01	0.54414
mmu-miR-29	-0.0260024	-1.5122301	-6.14976	1.88E-01	0.51079
mmu-miR-43	-0.0260017	-0.7639976	-6.945752	4.78E-01	0.74572
mmu-miR-11	-0.0259272	-1.5846763	-6.058611	1.71E-01	0.48169
mmu-miR-37	-0.0259232	-1.4186502	-6.264951	2.12E-01	0.53549
mmu-miR-54	-0.0257366	-1.3059962	-6.399143	2.45E-01	0.56747
mmu-miR-12	-0.0256927	-1.6643405	-5.95668	1.54E-01	0.45255
mmu-miR-68	-0.0255747	-0.7149576	-6.984624	5.05E-01	0.76532
mmu-miR-32	-0.0255427	-0.7845094	-6.92888	4.66E-01	0.73679
mmu-miR-70	-0.0255146	-1.9874941	-5.530236	1.00E-01	0.36527
mmu-miR-34	-0.0255064	-1.3314966	-6.369239	2.38E-01	0.5625
mmu-miR-72	-0.0254499	-1.6648522	-5.956021	1.54E-01	0.45255
mmu-miR-87	-0.0253486	-0.6723516	-7.016666	5.30E-01	0.77965
mmu-miR-72	-0.0253381	-1.9075525	-5.637177	1.12E-01	0.38145
mmu-miR-46	-0.0252185	-0.9612274	-6.769597	3.78E-01	0.69249
mmu-miR-12	-0.0251612	-0.6197465	-7.053926	5.61E-01	0.79891
mmu-miR-64	-0.0251108	-0.9847573	-6.746636	3.68E-01	0.68107
mmu-miR-69	-0.0250817	-1.0738176	-6.656361	3.29E-01	0.65063
mmu-miR-14	-0.0248707	-1.0581506	-6.672614	3.36E-01	0.65726
mmu-miR-21	-0.0248257	-0.2944712	-7.222391	7.80E-01	0.91369
mmu-miR-14	-0.0247896	-0.6641357	-7.022655	5.34E-01	0.78315
mmu-miR-63	-0.0246681	-1.4671841	-6.205593	1.99E-01	0.51936
mmu-miR-66	-0.0246103	-0.8083614	-6.908818	4.54E-01	0.73138
mmu-miR-70	-0.0245935	-1.3095728	-6.394966	2.44E-01	0.56747
mmu-miR-35	-0.0245607	-0.7016302	-6.994823	5.13E-01	0.76946
mmu-miR-63	-0.0245272	-1.2427231	-6.47204	2.66E-01	0.58563
mmu-miR-28	-0.0244221	-1.6202663	-6.013278	1.63E-01	0.46588
mmu-miR-29	-0.0244093	-1.3356685	-6.364319	2.36E-01	0.56045
mmu-miR-69	-0.0242584	-0.956554	-6.774111	3.80E-01	0.69262
mmu-miR-19	-0.024151	-1.4257996	-6.256262	2.10E-01	0.53439
mmu-miR-69	-0.0241061	-0.9613689	-6.76946	3.78E-01	0.69249
mmu-miR-69	-0.0240556	-1.513388	-6.148316	1.87E-01	0.51079
mmu-miR-65	-0.0240209	-1.1730497	-6.549994	2.91E-01	0.60718
mmu-miR-30	-0.0240122	-1.041474	-6.689743	3.43E-01	0.66301
mmu-miR-76	-0.0240071	-1.2351956	-6.480583	2.69E-01	0.58964
mmu-miR-11	-0.0239815	-0.1630083	-7.257598	8.77E-01	0.95487
mmu-miR-70	-0.0239319	-1.2800081	-6.429314	2.54E-01	0.57311
mmu-miR-56	-0.023816	-1.0867916	-6.642786	3.24E-01	0.64337
mmu-miR-30	-0.0237682	-1.3273018	-6.374178	2.39E-01	0.5625
mmu-miR-50	-0.0236788	-0.8250068	-6.89454	4.45E-01	0.72895
mmu-miR-69	-0.0235541	-0.9368589	-6.792964	3.90E-01	0.69775
mmu-miR-29	-0.0234372	-1.0599399	-6.670765	3.35E-01	0.65682
mmu-miR-76	-0.0232247	-1.1485298	-6.576806	3.00E-01	0.61381
mmu-miR-69	-0.0231914	-1.1989355	-6.521329	2.81E-01	0.59826
mmu-miR-18	-0.0230692	-0.7767699	-6.935287	4.71E-01	0.73971

mmu-miR-80	-0.0230419	-1.2994887	-6.406727	2.48E-01	0.56943
mmu-miR-44	-0.0230361	-1.166257	-6.557455	2.93E-01	0.60784
mmu-miR-72	-0.0230119	-1.4714839	-6.200293	1.98E-01	0.51936
mmu-miR-72	-0.0229447	-0.5119916	-7.121914	6.29E-01	0.83834
mmu-miR-34	-0.0229022	-0.8337925	-6.886914	4.40E-01	0.72895
mmu-miR-76	-0.0228566	-0.7868247	-6.926953	4.65E-01	0.73666
mmu-miR-48	-0.0227788	-1.489277	-6.178295	1.93E-01	0.51559
mmu-miR-69	-0.0226382	-0.4929231	-7.132733	6.42E-01	0.84191
mmu-miR-70	-0.022505	-0.8520899	-6.870833	4.31E-01	0.72744
mmu-miR-19	-0.0224616	-1.0288438	-6.702597	3.48E-01	0.66574
mmu-miR-10	-0.0224116	-1.3540384	-6.342567	2.31E-01	0.55391
mmu-miR-75	-0.0222598	-1.3258381	-6.375899	2.39E-01	0.5625
mmu-miR-66	-0.0222248	-0.4801774	-7.139756	6.50E-01	0.84775
mmu-miR-76	-0.0222241	-0.3517954	-7.2009	7.39E-01	0.89362
mmu-miR-21	-0.022158	-1.2575902	-6.455085	2.61E-01	0.5824
mmu-miR-19	-0.0221098	-1.2151563	-6.503186	2.76E-01	0.59614
mmu-miR-70	-0.0220912	-0.7663236	-6.943856	4.76E-01	0.74556
mmu-miR-43	-0.022022	-0.1227181	-7.264383	9.07E-01	0.96395
mmu-miR-63	-0.0219753	-0.5269581	-7.113162	6.20E-01	0.83395
mmu-let-7i-3	-0.0219709	-0.7582836	-6.950388	4.81E-01	0.748
mmu-miR-67	-0.0218924	-1.1450334	-6.580602	3.01E-01	0.61496
mmu-miR-76	-0.0218612	-1.0226273	-6.708885	3.51E-01	0.66817
mmu-miR-76	-0.021813	-0.7729077	-6.938466	4.73E-01	0.74201
mmu-miR-70	-0.0218062	-1.6959411	-5.915813	1.47E-01	0.43977
mmu-miR-69	-0.0217654	-1.206121	-6.513309	2.79E-01	0.59763
mmu-miR-63	-0.0217532	-1.4845924	-6.184097	1.95E-01	0.51559
mmu-miR-36	-0.0217061	-1.2642683	-6.447433	2.59E-01	0.58085
mmu-miR-72	-0.0216592	-0.9844435	-6.746944	3.68E-01	0.68107
mmu-miR-10	-0.0213163	-1.3782005	-6.313739	2.24E-01	0.54371
mmu-miR-48	-0.0212525	-1.4642491	-6.209207	2.00E-01	0.51936
mmu-miR-18	-0.0211508	-1.3773342	-6.314777	2.24E-01	0.54371
mmu-miR-30	-0.0210613	-1.2446351	-6.469866	2.66E-01	0.58563
mmu-miR-12	-0.0210114	-1.398604	-6.28921	2.18E-01	0.5409
mmu-miR-46	-0.0209131	-1.0272218	-6.70424	3.49E-01	0.66594
mmu-miR-68	-0.0208833	-0.8088325	-6.908417	4.53E-01	0.73138
mmu-miR-31	-0.0208471	-0.8743433	-6.85092	4.20E-01	0.71889
mmu-miR-59	-0.0208367	-0.6456271	-7.035918	5.45E-01	0.79078
mmu-miR-30	-0.0207851	-0.7943515	-6.920659	4.61E-01	0.73321
mmu-miR-70	-0.020774	-0.7367703	-6.967592	4.93E-01	0.75501
mmu-miR-63	-0.0206827	-1.1886044	-6.532813	2.85E-01	0.60192
hur_1	-0.0205127	-1.0293328	-6.702101	3.48E-01	0.66574
mmu-miR-76	-0.0204611	-0.8429769	-6.878875	4.36E-01	0.72895
mmu-miR-63	-0.0204016	-0.6385498	-7.040905	5.50E-01	0.79392
mmu-miR-15	-0.0203808	-0.9173145	-6.811396	3.99E-01	0.70309
mmu-miR-30	-0.0203255	-1.3100042	-6.394462	2.44E-01	0.56747
mmu-miR-13	-0.0202681	-1.0670251	-6.663426	3.32E-01	0.65453

mmu-miR-69	-0.0202011	-0.9241375	-6.804993	3.96E-01	0.70256
mmu-miR-36	-0.0201884	-0.9176267	-6.811104	3.99E-01	0.70309
mmu-miR-13	-0.0201275	-0.9898037	-6.741661	3.65E-01	0.67898
mmu-miR-31	-0.0200986	-0.7405396	-6.964607	4.91E-01	0.75421
mmu-miR-76	-0.0199666	-1.2835278	-6.425247	2.53E-01	0.57311
mmu-miR-18	-0.0198767	-0.8741017	-6.851138	4.20E-01	0.71889
mmu-miR-31	-0.0198683	-0.9834544	-6.747917	3.68E-01	0.68123
mmu-miR-45	-0.0198464	-0.8747466	-6.850556	4.20E-01	0.71889
mmu-miR-36	-0.0198409	-0.8148659	-6.903265	4.50E-01	0.73082
mmu-miR-22	-0.019802	-1.1852619	-6.536516	2.86E-01	0.60192
mmu-miR-30	-0.0197783	-1.3217056	-6.380755	2.41E-01	0.5648
mmu-miR-63	-0.0196902	-1.0497421	-6.681272	3.39E-01	0.65737
mmu-miR-70	-0.0196866	-0.6525166	-7.031019	5.41E-01	0.7878
mmu-miR-64	-0.0196639	-1.0640271	-6.666535	3.33E-01	0.65518
NC2_001060	-0.0196426	-1.4339331	-6.246353	2.08E-01	0.53133
mmu-miR-30	-0.019543	-0.8147349	-6.903377	4.50E-01	0.73082
mmu-miR-19	-0.0194711	-0.9261924	-6.803058	3.95E-01	0.70214
mmu-miR-76	-0.0194117	-0.7054409	-6.991923	5.10E-01	0.76738
mmu-miR-76	-0.0193992	-0.818867	-6.899833	4.48E-01	0.7298
mmu-miR-19	-0.0191866	-0.87154	-6.85345	4.21E-01	0.71889
mmu-miR-70	-0.0191396	-1.036146	-6.695178	3.45E-01	0.66453
mmu-miR-46	-0.0191324	-1.0509502	-6.680031	3.39E-01	0.65737
mmu-miR-19	-0.0190868	-1.3162009	-6.387211	2.42E-01	0.56603
mmu-miR-72	-0.0190823	-1.2871388	-6.421067	2.51E-01	0.57215
mmu-miR-63	-0.019077	-0.8546247	-6.868584	4.30E-01	0.72656
mmu-miR-70	-0.0190727	-0.9587286	-6.772013	3.79E-01	0.69262
mmu-miR-70	-0.0190226	-1.3819876	-6.309199	2.22E-01	0.54321
mmu-miR-51	-0.0189281	-0.8736117	-6.851581	4.20E-01	0.71889
mmu-miR-97	-0.0188841	-1.6400176	-5.987975	1.59E-01	0.46015
mmu-miR-76	-0.0187161	-0.9485171	-6.781838	3.84E-01	0.69435
mmu-miR-19	-0.0184624	-0.9109303	-6.817356	4.02E-01	0.70458
mmu-miR-76	-0.0183586	-1.1892594	-6.532086	2.85E-01	0.60192
mmu-miR-56	-0.0182836	-0.8861955	-6.840158	4.14E-01	0.7159
mmu-miR-70	-0.0180617	-1.1007741	-6.628041	3.19E-01	0.63707
mmu-miR-77	-0.0180556	-0.8885451	-6.838012	4.13E-01	0.7151
mmu-miR-30	-0.0180486	-1.1568706	-6.567723	2.97E-01	0.6111
mmu-miR-13	-0.0180108	-0.5093214	-7.123451	6.31E-01	0.83834
mmu-miR-70	-0.0179874	-1.0938854	-6.63532	3.21E-01	0.63933
mmu-miR-69	-0.0178987	-0.950462	-6.779972	3.83E-01	0.69435
mmu-miR-30	-0.0178651	-0.7255342	-6.976418	4.99E-01	0.76057
mmu-miR-10	-0.0178473	-0.7260399	-6.976023	4.99E-01	0.76057
mmu-miR-69	-0.0178361	-0.6065862	-7.062839	5.69E-01	0.80255
mmu-miR-69	-0.0177862	-0.5109384	-7.122521	6.30E-01	0.83834
mmu-miR-12	-0.0177202	-1.1736168	-6.549369	2.91E-01	0.60718
mmu-miR-21	-0.017705	-0.9555265	-6.775101	3.81E-01	0.69262
mmu-miR-45	-0.0176952	-0.6892308	-7.004168	5.20E-01	0.77178

mmu-miR-70	-0.0176928	-0.5572246	-7.094773	6.00E-01	0.82137
mmu-miR-63	-0.0176817	-0.9409687	-6.789053	3.88E-01	0.69625
mmu-miR-46	-0.017674	-1.0262747	-6.705199	3.49E-01	0.66594
mmu-miR-70	-0.0175914	-0.6337296	-7.044275	5.53E-01	0.79469
mmu-miR-70	-0.0174925	-0.8282181	-6.89176	4.43E-01	0.72895
mmu-miR-46	-0.0174572	-1.201473	-6.5185	2.81E-01	0.59826
mmu-miR-32	-0.0174493	-0.4149567	-7.173028	6.94E-01	0.8734
mmu-miR-70	-0.0174221	-0.4855585	-7.136811	6.47E-01	0.84599
mmu-miR-31	-0.017327	-0.3841668	-7.187157	7.16E-01	0.88229
mmu-miR-45	-0.0171626	-0.7141685	-6.985232	5.05E-01	0.76532
mmu-miR-30	-0.017141	-1.0518565	-6.679099	3.39E-01	0.65737
mmu-miR-69	-0.0170912	-0.5474342	-7.100822	6.06E-01	0.82464
mmu-miR-72	-0.0170375	-1.1688421	-6.554618	2.92E-01	0.60718
mmu-miR-46	-0.0170004	-0.1956901	-7.250704	8.52E-01	0.94232
mmu-miR-14	-0.0169377	-0.1789052	-7.2544	8.65E-01	0.94664
mmu-miR-56	-0.0168783	-1.0722904	-6.657952	3.30E-01	0.65119
mmu-miR-70	-0.0167883	-0.8080282	-6.909101	4.54E-01	0.73138
mmu-miR-19	-0.0167514	-1.2118122	-6.506938	2.77E-01	0.59647
hur_5	-0.0167215	-0.8201312	-6.898745	4.48E-01	0.72935
mmu-miR-31	-0.0166932	-0.8776381	-6.847939	4.18E-01	0.71889
mmu-miR-39	-0.0166648	-0.823828	-6.895558	4.46E-01	0.72907
mmu-miR-76	-0.0166174	-1.2296526	-6.486856	2.71E-01	0.59246
mmu-miR-37	-0.0165959	-0.7806436	-6.932087	4.68E-01	0.73829
mmu-miR-51	-0.0165729	-0.9956968	-6.73583	3.63E-01	0.67898
mmu-miR-13	-0.0165244	-0.6902533	-7.003403	5.19E-01	0.77178
mmu-miR-47	-0.0165234	-0.6402209	-7.039732	5.49E-01	0.7935
mmu-miR-18	-0.0163745	-0.8914907	-6.835316	4.11E-01	0.71389
mmu-miR-66	-0.0163612	-0.9932236	-6.73828	3.64E-01	0.67898
mmu-miR-21	-0.0162931	-0.663024	-7.023461	5.35E-01	0.78351
mmu-miR-14	-0.0162891	-0.8701888	-6.854667	4.22E-01	0.71889
mmu-miR-66	-0.0162602	-0.4198629	-7.170682	6.91E-01	0.8734
mmu-miR-70	-0.016224	-0.8075	-6.90955	4.54E-01	0.73138
mmu-miR-56	-0.0162166	-0.7390894	-6.965757	4.91E-01	0.75443
mmu-miR-69	-0.0161939	-0.6397246	-7.040081	5.49E-01	0.7935
mmu-miR-31	-0.0161918	-0.2269853	-7.242944	8.29E-01	0.93317
mmu-miR-69	-0.0161893	-0.8753689	-6.849993	4.19E-01	0.71889
mmu-miR-70	-0.0161433	-0.9515939	-6.778885	3.83E-01	0.69435
mmu-let-7g-3	-0.0161045	-1.0648975	-6.665633	3.33E-01	0.65515
mmu-miR-30	-0.0160783	-0.6694374	-7.018798	5.31E-01	0.78134
mmu-miR-35	-0.0159759	-0.631281	-7.045978	5.54E-01	0.79543
mmu-miR-48	-0.0159549	-0.6277351	-7.048435	5.56E-01	0.79671
mmu-miR-22	-0.015948	-0.4765758	-7.14171	6.53E-01	0.84914
mmu-miR-76	-0.0159287	-0.6258601	-7.049729	5.57E-01	0.79681
mmu-miR-69	-0.015908	-0.8426513	-6.879161	4.36E-01	0.72895
mmu-miR-21	-0.015819	-1.0172664	-6.714288	3.53E-01	0.67099
mmu-miR-31	-0.0157623	-0.9247222	-6.804443	3.95E-01	0.70256

mmu-miR-21	-0.0157235	-0.6973063	-6.998097	5.15E-01	0.77122
mmu-miR-64	-0.0156294	-1.1592062	-6.565173	2.96E-01	0.61062
mmu-miR-65	-0.0156042	-0.7915966	-6.922968	4.63E-01	0.73321
mmu-miR-11	-0.015592	-0.8661322	-6.858312	4.24E-01	0.71995
mmu-miR-29	-0.0155598	-1.0618633	-6.668776	3.34E-01	0.65627
mmu-miR-74	-0.0155339	-0.8275734	-6.892318	4.44E-01	0.72895
mmu-miR-69	-0.0155038	-0.7943913	-6.920625	4.61E-01	0.73321
mmu-miR-46	-0.0154587	-0.5804712	-7.08003	5.85E-01	0.81497
mmu-miR-70	-0.0154514	-1.268701	-6.442343	2.58E-01	0.57892
mmu-miR-76	-0.0154476	-1.3275107	-6.373932	2.39E-01	0.5625
mmu-miR-34	-0.0154393	-0.8104456	-6.907042	4.53E-01	0.73138
mmu-miR-21	-0.0154118	-1.0332653	-6.698109	3.46E-01	0.6651
mmu-miR-69	-0.0154042	-0.5785459	-7.081272	5.87E-01	0.81506
mmu-miR-88	-0.0153968	-0.6544349	-7.029647	5.40E-01	0.78734
mmu-miR-32	-0.015303	-0.4779599	-7.140961	6.52E-01	0.84852
mmu-miR-63	-0.0152381	-0.7412559	-6.964038	4.90E-01	0.7542
mmu-let-7a-:	-0.015205	-1.041192	-6.690031	3.43E-01	0.66301
mmu-miR-51	-0.0151965	-0.7032155	-6.993618	5.12E-01	0.7687
mmu-miR-39	-0.0151769	-0.8261795	-6.893526	4.44E-01	0.72895
mmu-miR-76	-0.0151737	-0.7593815	-6.949499	4.80E-01	0.74783
mmu-miR-75	-0.0151228	-0.8411499	-6.88048	4.37E-01	0.72895
mmu-miR-12	-0.0151075	-1.105751	-6.622764	3.17E-01	0.63513
mmu-miR-72	-0.0150806	-0.7927303	-6.922018	4.62E-01	0.73321
mmu-miR-32	-0.0149541	-0.9370704	-6.792764	3.89E-01	0.69775
mmu-miR-34	-0.0149533	-0.5113483	-7.122285	6.30E-01	0.83834
mmu-miR-27	-0.0149398	-0.6440155	-7.037058	5.46E-01	0.79097
mmu-miR-72	-0.0149038	-0.7975571	-6.917964	4.59E-01	0.73321
mmu-miR-70	-0.014881	-1.1482178	-6.577145	3.00E-01	0.61381
mmu-miR-30	-0.0147732	-0.9321257	-6.797454	3.92E-01	0.69911
mmu-miR-40	-0.0147583	-0.7688472	-6.941795	4.75E-01	0.74402
mmu-miR-69	-0.0147393	-0.538622	-7.106184	6.12E-01	0.82912
mmu-miR-76	-0.0147285	-0.138837	-7.261897	8.95E-01	0.95992
mmu-miR-76	-0.0147137	-0.528239	-7.112402	6.19E-01	0.83389
mmu-miR-46	-0.0146823	-0.6094061	-7.060943	5.67E-01	0.80201
mmu-miR-21	-0.0146497	-0.8487949	-6.873748	4.33E-01	0.72895
mmu-miR-58	-0.0146298	-0.8848688	-6.841368	4.15E-01	0.7159
mmu-miR-87	-0.0146202	-0.9126584	-6.815746	4.01E-01	0.70456
mmu-miR-87	-0.0145183	-0.3547354	-7.199699	7.36E-01	0.89167
mmu-miR-30	-0.0145177	-0.933154	-6.79648	3.91E-01	0.6989
mmu-miR-62	-0.0144251	-0.6883377	-7.004836	5.20E-01	0.77178
mmu-miR-30	-0.0144153	-0.6660599	-7.021258	5.33E-01	0.78253
mmu-miR-19	-0.0144002	-0.941388	-6.788654	3.87E-01	0.69625
mmu-miR-12	-0.0143155	-0.5434011	-7.103285	6.09E-01	0.82593
mmu-miR-66	-0.0142322	-0.6116309	-7.059442	5.66E-01	0.80183
mmu-miR-72	-0.0142267	-0.6205988	-7.053343	5.61E-01	0.79891
mmu-miR-69	-0.014194	-0.5367784	-7.107296	6.13E-01	0.82972

mmu-miR-74	-0.0141173	-0.5172379	-7.118872	6.26E-01	0.83829
mmu-miR-38	-0.0140773	-0.921192	-6.807761	3.97E-01	0.70309
mmu-miR-70	-0.0139745	-0.6808477	-7.010408	5.25E-01	0.77509
mmu-miR-19	-0.0138355	-0.5846121	-7.077349	5.83E-01	0.8138
mmu-miR-58	-0.0138286	-0.822933	-6.896331	4.46E-01	0.72907
mmu-miR-69	-0.0137976	-0.9724117	-6.758731	3.73E-01	0.68711
mmu-miR-12	-0.0137833	-0.6492508	-7.033347	5.43E-01	0.78823
mmu-miR-72	-0.013772	-0.9029434	-6.824769	4.06E-01	0.70743
mmu-miR-92	-0.0136934	-0.6964875	-6.998716	5.16E-01	0.77122
mmu-miR-47	-0.0136046	-0.4995019	-7.129042	6.38E-01	0.84029
mmu-miR-70	-0.0136042	-0.4430475	-7.159249	6.75E-01	0.86547
mmu-miR-70	-0.013479	-0.5510292	-7.098612	6.04E-01	0.82253
mmu-miR-1a	-0.0134487	-0.6328245	-7.044905	5.53E-01	0.79469
mmu-miR-65	-0.0134354	-0.9906076	-6.740867	3.65E-01	0.67898
mmu-miR-70	-0.0134304	-0.5739938	-7.084192	5.90E-01	0.81758
mmu-miR-39	-0.0134032	-0.7640557	-6.945704	4.78E-01	0.74572
mmu-miR-76	-0.0133521	-0.88128	-6.844635	4.16E-01	0.71803
mmu-miR-11	-0.0133416	-0.5240825	-7.114861	6.21E-01	0.83531
mmu-miR-70	-0.0133386	-0.503615	-7.126712	6.35E-01	0.83905
mmu-miR-69	-0.0132808	-0.6169296	-7.055848	5.63E-01	0.80012
mmu-miR-69	-0.0131916	-0.6100134	-7.060534	5.67E-01	0.80201
mmu-miR-63	-0.0131665	-0.8067573	-6.910181	4.55E-01	0.73138
mmu-miR-80	-0.0129391	-0.9465945	-6.78368	3.85E-01	0.69435
mmu-miR-19	-0.0128337	-0.7787574	-6.933647	4.70E-01	0.73924
mmu-miR-19	-0.0128333	-0.7173954	-6.982741	5.04E-01	0.76532
mmu-miR-47	-0.0127314	-0.6856743	-7.006823	5.22E-01	0.77235
mmu-miR-69	-0.0127262	-0.5713661	-7.085868	5.91E-01	0.81773
mmu-miR-39	-0.0126953	-0.7847304	-6.928696	4.66E-01	0.73679
mmu-miR-41	-0.012674	-0.3644849	-7.19565	7.30E-01	0.88942
mmu-miR-70	-0.012574	-0.3450793	-7.203607	7.43E-01	0.89766
mmu-miR-30	-0.0125287	-0.7478426	-6.958788	4.86E-01	0.74978
mmu-miR-38	-0.0124132	-0.3354663	-7.207395	7.50E-01	0.89855
mmu-miR-34	-0.0123871	-0.5894288	-7.074208	5.80E-01	0.81213
mmu-miR-19	-0.0121966	-0.5794078	-7.080716	5.86E-01	0.81497
mmu-miR-70	-0.0121801	-0.4048998	-7.177756	7.01E-01	0.87595
mmu-miR-56	-0.0121654	-0.7086052	-6.989505	5.09E-01	0.7663
mmu-miR-76	-0.0120651	-0.6335988	-7.044366	5.53E-01	0.79469
mmu-miR-44	-0.0120312	-0.7493612	-6.957572	4.86E-01	0.74969
mmu-miR-67	-0.0120302	-0.6801754	-7.010906	5.25E-01	0.77509
mmu-miR-30	-0.0120182	-0.5609308	-7.092458	5.98E-01	0.82137
mmu-miR-44	-0.0119892	-0.6925782	-7.001659	5.18E-01	0.77122
mmu-miR-69	-0.0119259	-0.5074456	-7.124527	6.32E-01	0.83834
mmu-miR-20	-0.0118705	-0.6978264	-6.997704	5.15E-01	0.77122
mmu-miR-30	-0.011837	-0.7645558	-6.945297	4.77E-01	0.74572
mmu-miR-39	-0.0117434	-0.431471	-7.165029	6.83E-01	0.86815
mmu-miR-69	-0.0117364	-0.5594678	-7.093374	5.99E-01	0.82137

mmu-miR-19	-0.0117246	-0.2644767	-7.232166	8.01E-01	0.92372
mmu-miR-36	-0.0116871	-0.5659888	-7.089277	5.95E-01	0.81931
mmu-miR-23	-0.0116712	-0.3081927	-7.217581	7.70E-01	0.90921
mmu-miR-69	-0.0116676	-0.9456037	-6.784628	3.86E-01	0.69435
mmu-miR-25	-0.0115073	-0.5526206	-7.097629	6.03E-01	0.82224
mmu-miR-21	-0.0114807	-0.8216013	-6.897479	4.47E-01	0.72914
mmu-miR-15	-0.01139	-0.8272752	-6.892577	4.44E-01	0.72895
mmu-miR-69	-0.0113405	-0.3647766	-7.195527	7.29E-01	0.88942
mmu-miR-69	-0.0112891	-0.3621274	-7.196639	7.31E-01	0.88942
mmu-miR-46	-0.0111913	-0.3167861	-7.214461	7.64E-01	0.90651
mmu-miR-19	-0.0111054	-0.5850763	-7.077047	5.83E-01	0.8138
mmu-miR-59	-0.0110629	-0.3822528	-7.188002	7.17E-01	0.88229
mmu-miR-1b	-0.0110531	-0.5120641	-7.121872	6.29E-01	0.83834
mmu-miR-69	-0.0109884	-0.3789799	-7.189437	7.19E-01	0.88339
mmu-miR-63	-0.0109749	-0.9133977	-6.815056	4.01E-01	0.70456
mmu-miR-33	-0.0109581	-0.1363378	-7.262302	8.97E-01	0.95992
mmu-miR-20	-0.0107731	-0.5723517	-7.08524	5.91E-01	0.81772
mmu-miR-19	-0.0107656	-0.4711198	-7.144644	6.56E-01	0.85271
mmu-miR-70	-0.0107538	-0.4833658	-7.138015	6.48E-01	0.84652
mmu-miR-69	-0.0107256	-0.5707035	-7.08629	5.92E-01	0.81773
mmu-miR-35	-0.0106733	-0.5971462	-7.069129	5.75E-01	0.80719
mmu-miR-70	-0.0106729	-0.4260736	-7.167675	6.87E-01	0.87033
mmu-miR-72	-0.0105805	-0.4131353	-7.173892	6.96E-01	0.8734
mmu-miR-30	-0.010543	-0.4252279	-7.168087	6.87E-01	0.87048
mmu-miR-34	-0.0105305	-0.5089028	-7.123692	6.31E-01	0.83834
mmu-miR-64	-0.0105183	-0.7084228	-6.989644	5.09E-01	0.7663
mmu-miR-81	-0.0104303	-0.7097858	-6.9886	5.08E-01	0.7663
mmu-miR-70	-0.0103547	-0.6039556	-7.0646	5.71E-01	0.80349
mmu-miR-14	-0.01034	-0.5226837	-7.115685	6.22E-01	0.83535
mmu-miR-36	-0.0103362	-0.6926995	-7.001568	5.18E-01	0.77122
mmu-miR-46	-0.0103332	-0.4161939	-7.172439	6.94E-01	0.8734
mmu-miR-29	-0.0102368	-0.3901548	-7.18449	7.12E-01	0.88121
mmu-miR-56	-0.0101849	-0.5278463	-7.112635	6.19E-01	0.83389
mmu-miR-72	-0.0101448	-0.4650662	-7.147864	6.60E-01	0.85623
mmu-miR-19	-0.010128	-0.4670938	-7.14679	6.59E-01	0.85504
mmu-miR-29	-0.0100963	-0.5292756	-7.111786	6.18E-01	0.83389
mmu-miR-70	-0.0100659	-0.5720799	-7.085413	5.91E-01	0.81772
mmu-miR-81	-0.0099851	-0.4303403	-7.165586	6.84E-01	0.86815
mmu-miR-21	-0.0099421	-0.5654889	-7.089593	5.95E-01	0.81931
mmu-miR-76	-0.0098668	-0.4121301	-7.174368	6.96E-01	0.8734
mmu-miR-12	-0.0098369	-0.5277356	-7.112701	6.19E-01	0.83389
mmu-miR-76	-0.0097228	-0.6031244	-7.065156	5.71E-01	0.80349
mmu-miR-76	-0.0096591	-0.6092695	-7.061035	5.68E-01	0.80201
mmu-miR-76	-0.0096089	-0.3404035	-7.205462	7.47E-01	0.89769
mmu-miR-19	-0.009517	-0.5796396	-7.080567	5.86E-01	0.81497
mmu-miR-27	-0.0095039	-0.3765767	-7.190483	7.21E-01	0.88427

mmu-miR-24	-0.0094654	-0.0694607	-7.270428	9.47E-01	0.97643
mmu-miR-34	-0.0094181	-0.4964751	-7.130746	6.39E-01	0.84175
mmu-miR-54	-0.0093294	-0.5830019	-7.078393	5.84E-01	0.81415
mmu-miR-29	-0.0093106	-0.3830039	-7.187671	7.17E-01	0.88229
mmu-miR-30	-0.0092145	-0.5064738	-7.125083	6.33E-01	0.83834
mmu-miR-76	-0.0091138	-0.5642021	-7.090404	5.96E-01	0.81984
mmu-miR-70	-0.0091112	-0.3100866	-7.216901	7.68E-01	0.90921
mmu-miR-30	-0.0090845	-0.4924769	-7.132982	6.42E-01	0.84191
mmu-miR-12	-0.0090332	-0.4064025	-7.177056	7.00E-01	0.87523
mmu-miR-36	-0.0090083	-0.5076063	-7.124435	6.32E-01	0.83834
mmu-miR-67	-0.0089997	-0.7197679	-6.980904	5.02E-01	0.76415
mmu-miR-87	-0.008914	-0.5736131	-7.084435	5.90E-01	0.81758
mmu-miR-96	-0.0088848	-0.4814837	-7.139044	6.49E-01	0.8472
mmu-miR-88	-0.0088805	-0.3581425	-7.198296	7.34E-01	0.89093
mmu-miR-12	-0.0088197	-0.607866	-7.061979	5.68E-01	0.80204
mmu-miR-66	-0.0086175	-0.3271577	-7.210586	7.56E-01	0.9033
mmu-miR-76	-0.0086133	-0.3856519	-7.186499	7.15E-01	0.88222
mmu-miR-70	-0.0085249	-0.4455317	-7.15799	6.74E-01	0.86547
mmu-miR-70	-0.0084661	-0.5436832	-7.103114	6.09E-01	0.82593
mmu-miR-69	-0.0084505	-0.3939901	-7.182761	7.09E-01	0.87848
mmu-miR-81	-0.0083099	-0.5263142	-7.113543	6.20E-01	0.83395
mmu-miR-46	-0.0082929	-0.3777713	-7.189963	7.20E-01	0.88386
mmu-miR-30	-0.0082897	-0.5336165	-7.109195	6.15E-01	0.83171
mmu-miR-22	-0.0082697	-0.4479992	-7.156733	6.72E-01	0.86466
mmu-miR-69	-0.0081854	-0.1892658	-7.252158	8.57E-01	0.94268
mmu-miR-63	-0.008123	-0.5132592	-7.121181	6.28E-01	0.83834
mmu-miR-46	-0.0081057	-0.2645465	-7.232144	8.01E-01	0.92372
mmu-miR-54	-0.0080775	-0.4160475	-7.172508	6.94E-01	0.8734
mmu-miR-33	-0.0080502	-0.4282954	-7.16659	6.85E-01	0.86898
NC2_001227	-0.0080394	-0.6690077	-7.019111	5.32E-01	0.78134
mmu-miR-11	-0.0080108	-0.3154869	-7.214938	7.64E-01	0.90705
mmu-miR-64	-0.0078418	-0.2074112	-7.24793	8.43E-01	0.9385
mmu-miR-30	-0.0078339	-0.2469239	-7.237412	8.14E-01	0.92676
mmu-miR-63	-0.0078051	-0.6115278	-7.059511	5.66E-01	0.80183
mmu-miR-69	-0.0077048	-0.4129641	-7.173973	6.96E-01	0.8734
mmu-miR-70	-0.0076731	-0.6027739	-7.065389	5.72E-01	0.80349
mmu-miR-56	-0.0076466	-0.4558717	-7.15268	6.67E-01	0.86131
mmu-miR-15	-0.007598	-0.1155705	-7.265388	9.12E-01	0.96717
mmu-miR-67	-0.0075967	-0.3295543	-7.209673	7.54E-01	0.90185
mmu-miR-49	-0.0075613	-0.3194956	-7.21346	7.62E-01	0.90649
mmu-miR-68	-0.0075319	-0.5468391	-7.101186	6.07E-01	0.82464
mmu-miR-70	-0.0074451	-0.3184506	-7.213847	7.62E-01	0.90649
mmu-miR-76	-0.0073867	-0.4534688	-7.153924	6.68E-01	0.86223
mmu-miR-80	-0.0073722	-0.4434054	-7.159068	6.75E-01	0.86547
mmu-miR-70	-0.0073661	-0.3342102	-7.207882	7.51E-01	0.89855
mmu-miR-72	-0.0073267	-0.2983039	-7.221069	7.77E-01	0.91219

mmu-miR-1a	-0.0069018	-0.2060681	-7.248256	8.44E-01	0.9385
mmu-miR-74	-0.0068621	-0.2207407	-7.244583	8.34E-01	0.93371
mmu-miR-69	-0.0068224	-0.4134666	-7.173735	6.95E-01	0.8734
mmu-miR-21	-0.0067301	-0.3624071	-7.196522	7.31E-01	0.88942
mmu-miR-21	-0.006492	-0.4395669	-7.161002	6.78E-01	0.86667
mmu-miR-29	-0.0064329	-0.2190863	-7.245009	8.35E-01	0.9345
mmu-miR-63	-0.006319	-0.3265259	-7.210825	7.57E-01	0.9033
mmu-miR-70	-0.0062534	-0.35758	-7.198529	7.34E-01	0.89093
mmu-miR-15	-0.0062249	-0.3180479	-7.213996	7.63E-01	0.90649
mmu-miR-72	-0.0062069	-0.4970238	-7.130438	6.39E-01	0.84175
mmu-miR-69	-0.0061741	-0.3593582	-7.197792	7.33E-01	0.89055
mmu-miR-12	-0.0061233	-0.401741	-7.179218	7.04E-01	0.8768
mmu-miR-70	-0.006072	-0.3047189	-7.218819	7.72E-01	0.91
mmu-miR-69	-0.005992	-0.3174775	-7.214206	7.63E-01	0.90649
mmu-miR-11	-0.0059458	-0.4098507	-7.175442	6.98E-01	0.8734
mmu-miR-18	-0.0059303	-0.2155061	-7.245922	8.37E-01	0.93584
mmu-miR-76	-0.0057215	-0.2103186	-7.247217	8.41E-01	0.93798
mmu-miR-37	-0.0057119	-0.1924743	-7.251438	8.55E-01	0.94268
mmu-miR-19	-0.0056657	-0.2516744	-7.236027	8.11E-01	0.92676
mmu-miR-70	-0.0056195	-0.365457	-7.19524	7.29E-01	0.88942
mmu-miR-69	-0.0056131	-0.2726728	-7.229596	7.95E-01	0.92026
mmu-miR-69	-0.0055599	-0.2307442	-7.241936	8.26E-01	0.93147
mmu-miR-70	-0.0054421	-0.2395347	-7.239515	8.20E-01	0.92965
mmu-miR-76	-0.0054346	-0.298151	-7.221122	7.77E-01	0.91219
mmu-miR-30	-0.0054266	-0.4103569	-7.175204	6.98E-01	0.8734
mmu-miR-13	-0.0053335	-0.3069705	-7.218018	7.71E-01	0.90921
mmu-miR-51	-0.0053154	-0.2855246	-7.225413	7.86E-01	0.91612
mmu-miR-88	-0.005303	-0.1744082	-7.255335	8.68E-01	0.94939
mmu-miR-76	-0.0052233	-0.2556443	-7.234849	8.08E-01	0.92676
mmu-miR-76	-0.0051056	-0.3425795	-7.204602	7.45E-01	0.89769
mmu-miR-76	-0.0050827	-0.2857144	-7.22535	7.86E-01	0.91612
mmu-miR-72	-0.005068	-0.1722636	-7.255772	8.70E-01	0.9495
mmu-miR-69	-0.0050647	-0.3351008	-7.207537	7.50E-01	0.89855
mmu-miR-70	-0.0050245	-0.2475582	-7.237228	8.14E-01	0.92676
mmu-miR-51	-0.0049332	-0.2716574	-7.229918	7.96E-01	0.92056
mmu-miR-51	-0.0049305	-0.2309561	-7.241878	8.26E-01	0.93147
mmu-miR-30	-0.0049091	-0.2510851	-7.2362	8.11E-01	0.92676
mmu-miR-15	-0.0048181	-0.0756063	-7.269901	9.43E-01	0.97485
mmu-miR-69	-0.0048074	-0.2649806	-7.23201	8.01E-01	0.92372
mmu-miR-69	-0.0047616	-0.3023185	-7.219666	7.74E-01	0.91062
mmu-miR-30	-0.0047583	-0.3952221	-7.182202	7.08E-01	0.87799
mmu-miR-30	-0.0046326	-0.2304859	-7.242006	8.26E-01	0.93147
mmu-miR-19	-0.0045	-0.3029251	-7.219453	7.73E-01	0.91062
mmu-miR-48	-0.0043734	-0.2734429	-7.229351	7.95E-01	0.92026
mmu-miR-31	-0.0043492	-0.215989	-7.245799	8.37E-01	0.93584
mmu-miR-69	-0.0043461	-0.2016098	-7.249323	8.48E-01	0.94018

mmu-miR-72	-0.0043113	-0.0929929	-7.268169	9.29E-01	0.96923
mmu-miR-72	-0.0042635	-0.2689076	-7.230786	7.98E-01	0.92175
mmu-miR-31	-0.0041174	-0.2363735	-7.240396	8.22E-01	0.92985
mmu-miR-72	-0.0040859	-0.2495371	-7.236653	8.12E-01	0.92676
mmu-miR-51	-0.0040763	-0.2216067	-7.244358	8.33E-01	0.93354
mmu-miR-64	-0.0040682	-0.2184551	-7.245171	8.35E-01	0.9345
mmu-miR-88	-0.004056	-0.189487	-7.252108	8.57E-01	0.94268
mmu-miR-63	-0.0040347	-0.1833598	-7.253451	8.61E-01	0.94479
mmu-miR-69	-0.0040108	-0.1524265	-7.259564	8.84E-01	0.95745
hur_4	-0.0040058	-0.101903	-7.267143	9.23E-01	0.96891
mmu-miR-69	-0.0039691	-0.2242695	-7.243662	8.31E-01	0.93317
mmu-miR-34	-0.0039577	-0.2923958	-7.2231	7.81E-01	0.91369
mmu-miR-21	-0.0039419	-0.2894492	-7.224099	7.83E-01	0.91506
mmu-miR-56	-0.0039015	-0.1304864	-7.263223	9.01E-01	0.96059
mmu-miR-72	-0.0038919	-0.251547	-7.236064	8.11E-01	0.92676
mmu-miR-29	-0.003794	-0.2300713	-7.242117	8.27E-01	0.93147
mmu-miR-29	-0.0037308	-0.1843901	-7.253228	8.61E-01	0.94479
mmu-miR-69	-0.0037222	-0.232189	-7.241544	8.25E-01	0.93147
mmu-miR-70	-0.0037151	-0.2101538	-7.247258	8.41E-01	0.93798
mmu-miR-29	-0.003653	-0.0978149	-7.267625	9.26E-01	0.96891
mmu-miR-29	-0.0036409	-0.1533918	-7.25939	8.84E-01	0.95745
mmu-miR-66	-0.0036187	-0.1560237	-7.258911	8.82E-01	0.95745
mmu-miR-74	-0.0035957	-0.1362245	-7.26232	8.97E-01	0.95992
mmu-miR-69	-0.0035844	-0.247967	-7.23711	8.13E-01	0.92676
mmu-miR-69	-0.0035759	-0.2382396	-7.239877	8.21E-01	0.92965
mmu-miR-63	-0.003536	-0.1669344	-7.256836	8.74E-01	0.95276
mmu-miR-68	-0.0033595	-0.1589651	-7.258365	8.80E-01	0.95653
mmu-miR-33	-0.0033091	-0.1307412	-7.263184	9.01E-01	0.96059
mmu-miR-63	-0.0033076	-0.1349664	-7.262522	8.98E-01	0.95992
mmu-miR-37	-0.0033038	-0.2096041	-7.247394	8.42E-01	0.93798
mmu-miR-72	-0.0032627	-0.2056463	-7.248358	8.45E-01	0.9385
mmu-let-7f-2	-0.0031938	-0.099495	-7.267429	9.24E-01	0.96891
NC1_000002	-0.0031579	-0.2727897	-7.229559	7.95E-01	0.92026
mmu-miR-31	-0.0031085	-0.1652979	-7.257156	8.75E-01	0.95355
mmu-miR-20	-0.0030851	-0.0197463	-7.273051	9.85E-01	0.99232
mmu-miR-19	-0.0030557	-0.1498671	-7.26002	8.86E-01	0.95745
mmu-miR-70	-0.0030517	-0.1727273	-7.255678	8.69E-01	0.9495
mmu-miR-70	-0.0030274	-0.0979146	-7.267614	9.26E-01	0.96891
mmu-miR-63	-0.0030079	-0.2227471	-7.244061	8.32E-01	0.93317
mmu-miR-34	-0.0029868	-0.14708	-7.260508	8.89E-01	0.95781
mmu-miR-46	-0.0029436	-0.146663	-7.26058	8.89E-01	0.95781
mmu-miR-63	-0.002938	-0.178433	-7.254499	8.65E-01	0.94664
mmu-miR-63	-0.0028748	-0.1728287	-7.255657	8.69E-01	0.9495
mmu-miR-30	-0.0028582	-0.1313472	-7.26309	9.00E-01	0.96059
mmu-miR-69	-0.0028472	-0.2243261	-7.243647	8.31E-01	0.93317
mmu-miR-70	-0.0027447	-0.1484488	-7.260269	8.87E-01	0.95745

mmu-miR-69	-0.0026765	-0.1411219	-7.26152	8.93E-01	0.95992
mmu-miR-59	-0.0026369	-0.1118708	-7.265885	9.15E-01	0.96744
mmu-miR-18	-0.0025357	-0.0776788	-7.269713	9.41E-01	0.97423
mmu-miR-69	-0.0025066	-0.1326646	-7.262885	8.99E-01	0.96046
mmu-miR-14	-0.0024827	-0.049547	-7.271829	9.62E-01	0.98562
mmu-miR-56	-0.0024819	-0.1969459	-7.250415	8.51E-01	0.94187
mmu-miR-70	-0.0024363	-0.1388446	-7.261895	8.95E-01	0.95992
mmu-miR-70	-0.0023465	-0.183744	-7.253368	8.61E-01	0.94479
mmu-miR-45	-0.0023427	-0.1359452	-7.262365	8.97E-01	0.95992
mmu-miR-11	-0.0023405	-0.1062883	-7.266604	9.19E-01	0.96891
mmu-miR-70	-0.0022888	-0.1235625	-7.264261	9.06E-01	0.96395
mmu-miR-30	-0.0022267	-0.101204	-7.267227	9.23E-01	0.96891
mmu-miR-49	-0.0022174	-0.0914433	-7.268338	9.31E-01	0.96923
mmu-miR-62	-0.0021646	-0.1222777	-7.264447	9.07E-01	0.96395
mmu-miR-19	-0.0021468	-0.0695146	-7.270423	9.47E-01	0.97643
mmu-miR-67	-0.0020932	-0.1505847	-7.259893	8.86E-01	0.95745
mmu-miR-39	-0.0019889	-0.0701126	-7.270374	9.47E-01	0.97643
mmu-miR-76	-0.0018781	-0.0884446	-7.268656	9.33E-01	0.9697
mmu-miR-72	-0.0018605	-0.1223641	-7.264434	9.07E-01	0.96395
mmu-miR-30	-0.0018343	-0.0821465	-7.269291	9.38E-01	0.97231
mmu-miR-29	-0.0018257	-0.0678546	-7.270558	9.48E-01	0.97663
mmu-miR-56	-0.0018083	-0.0741943	-7.270026	9.44E-01	0.97537
mmu-miR-45	-0.001795	-0.0939036	-7.268068	9.29E-01	0.96923
mmu-miR-19	-0.0017547	-0.1138952	-7.265615	9.14E-01	0.96744
mmu-miR-76	-0.0016618	-0.1136666	-7.265646	9.14E-01	0.96744
mmu-miR-69	-0.001626	-0.0981613	-7.267585	9.25E-01	0.96891
mmu-miR-31	-0.0015592	-0.1108563	-7.266018	9.16E-01	0.96771
mmu-miR-34	-0.0015583	-0.1033312	-7.26697	9.22E-01	0.96891
mmu-miR-70	-0.0014951	-0.0920568	-7.268271	9.30E-01	0.96923
mmu-miR-21	-0.0014515	-0.1094261	-7.266204	9.17E-01	0.968
mmu-miR-72	-0.0014381	-0.1036454	-7.266932	9.21E-01	0.96891
mmu-miR-57	-0.0014311	-0.0196796	-7.273052	9.85E-01	0.99232
mmu-miR-34	-0.0014281	-0.096698	-7.267754	9.27E-01	0.96891
mmu-miR-49	-0.0013272	-0.1012884	-7.267217	9.23E-01	0.96891
mmu-miR-42	-0.0012529	-0.0367001	-7.272484	9.72E-01	0.9885
mmu-miR-54	-0.001213	-0.0360809	-7.272511	9.73E-01	0.9885
mmu-miR-71	-0.0011882	-0.0646758	-7.270807	9.51E-01	0.97806
mmu-miR-81	-0.0010267	-0.0462967	-7.272013	9.65E-01	0.98562
mmu-miR-71	-0.0009985	-0.0432745	-7.272173	9.67E-01	0.98625
mmu-miR-76	-0.0009384	-0.0337511	-7.272607	9.74E-01	0.9885
mmu-miR-76	-0.0009375	-0.0365083	-7.272493	9.72E-01	0.9885
mmu-miR-30	-0.0008755	-0.0462043	-7.272018	9.65E-01	0.98562
mmu-miR-19	-0.0007724	-0.0594541	-7.27119	9.55E-01	0.98108
mmu-miR-48	-0.0006909	-0.0237178	-7.272948	9.82E-01	0.99232
mmu-miR-54	-0.0006781	-0.0203274	-7.273037	9.85E-01	0.99232
mmu-miR-21	-0.0006748	-0.0402729	-7.272322	9.69E-01	0.98752

mmu-miR-69	-0.0006655	-0.0325147	-7.272656	9.75E-01	0.98877
mmu-miR-70	-0.0006418	-0.0335529	-7.272615	9.74E-01	0.9885
mmu-miR-30	-0.0005969	-0.0384638	-7.272406	9.71E-01	0.9884
mmu-miR-69	-0.0005259	-0.0160618	-7.273129	9.88E-01	0.99457
mmu-miR-19	-0.0003319	-0.0199112	-7.273047	9.85E-01	0.99232
mmu-miR-74	-0.0002127	-0.0059197	-7.273261	9.95E-01	0.99995
mmu-miR-30	-7.407E-05	-0.0033912	-7.273275	9.97E-01	0.99995
mmu-miR-30	-5.271E-05	-0.0035595	-7.273274	9.97E-01	0.99995
mmu-miR-67	-2.865E-05	-0.0016354	-7.27328	9.99E-01	0.99995
Blank	-2.72E-06	-0.0002427	-7.273281	1	0.99995
mmu-miR-66	-1.31E-06	-0.0000695	-7.273281	1	0.99995
mmu-miR-30	0.00001373	0.0007551	-7.273281	9.99E-01	0.99995
mmu-miR-70	0.0000162	0.0007157	-7.273281	9.99E-01	0.99995
hur_2	0.00004008	0.0035394	-7.273274	9.97E-01	0.99995
mmu-miR-46	0.00005936	0.002059	-7.273279	9.98E-01	0.99995
mmu-miR-30	0.00008548	0.003676	-7.273273	9.97E-01	0.99995
mmu-miR-10	0.00014225	0.0014481	-7.27328	9.99E-01	0.99995
mmu-miR-87	0.0002483	0.0075035	-7.273248	9.94E-01	0.99995
mmu-miR-48	0.00038628	0.024629	-7.272922	9.81E-01	0.99232
mmu-miR-87	0.0005002	0.0231677	-7.272964	9.82E-01	0.99232
mmu-miR-19	0.00053596	0.0352854	-7.272545	9.73E-01	0.9885
mmu-miR-67	0.00055834	0.0288057	-7.27279	9.78E-01	0.9911
mmu-miR-30	0.00057949	0.0222022	-7.27299	9.83E-01	0.99232
mmu-miR-74	0.0006204	0.022132	-7.272992	9.83E-01	0.99232
mmu-miR-63	0.00065819	0.0441004	-7.272131	9.66E-01	0.98625
mmu-miR-72	0.0006858	0.0540536	-7.271553	9.59E-01	0.98317
mmu-miR-70	0.00083155	0.0472046	-7.271963	9.64E-01	0.98562
mmu-miR-20	0.00094327	0.0629967	-7.270934	9.52E-01	0.97884
mmu-miR-20	0.0009856	0.0252867	-7.272903	9.81E-01	0.99232
mmu-let-7b-!	0.00099902	0.0485043	-7.27189	9.63E-01	0.98562
mmu-miR-70	0.00101082	0.0241473	-7.272936	9.82E-01	0.99232
mmu-miR-70	0.00110968	0.0733756	-7.270097	9.44E-01	0.97549
mmu-miR-30	0.00113027	0.0551853	-7.27148	9.58E-01	0.98282
mmu-miR-49	0.00115687	0.0565901	-7.271387	9.57E-01	0.98225
mmu-miR-47	0.00119395	0.0584982	-7.271257	9.55E-01	0.98129
mmu-miR-30	0.00120299	0.0755384	-7.269907	9.43E-01	0.97485
mmu-miR-46	0.00120899	0.0435356	-7.27216	9.67E-01	0.98625
mmu-miR-30	0.00121539	0.0461386	-7.272022	9.65E-01	0.98562
mmu-miR-19	0.00127723	0.0709279	-7.270306	9.46E-01	0.97643
mmu-miR-76	0.00128422	0.0801725	-7.26948	9.39E-01	0.97319
mmu-miR-63	0.00131954	0.0966474	-7.267759	9.27E-01	0.96891
mmu-miR-30	0.00139755	0.0667657	-7.270645	9.49E-01	0.97696
mmu-miR-76	0.00143513	0.0871154	-7.268794	9.34E-01	0.96999
mmu-miR-76	0.00145957	0.0881525	-7.268687	9.33E-01	0.9697
mmu-miR-12	0.0015014	0.0498062	-7.271814	9.62E-01	0.98562
mmu-miR-61	0.00157947	0.0954645	-7.267894	9.27E-01	0.96891

mmu-miR-72	0.00172253	0.1250363	-7.264044	9.05E-01	0.96389
mmu-miR-72	0.00177591	0.0682447	-7.270527	9.48E-01	0.97663
mmu-miR-19	0.00187985	0.0887664	-7.268623	9.33E-01	0.9697
mmu-miR-19	0.00194527	0.1512585	-7.259773	8.85E-01	0.95745
mmu-miR-72	0.00199024	0.1121178	-7.265852	9.15E-01	0.96744
mmu-miR-80	0.00212006	0.1560046	-7.258914	8.82E-01	0.95745
mmu-miR-56	0.00225559	0.0958454	-7.267851	9.27E-01	0.96891
mmu-miR-70	0.00231696	0.0961848	-7.267812	9.27E-01	0.96891
mmu-miR-29	0.00242258	0.1545869	-7.259173	8.83E-01	0.95745
mmu-miR-59	0.00251014	0.109151	-7.26624	9.17E-01	0.968
mmu-miR-71	0.002522	0.1371152	-7.262177	8.96E-01	0.95992
mmu-miR-63	0.00254076	0.0796805	-7.269527	9.39E-01	0.97319
mmu-miR-70	0.00270449	0.1177202	-7.265092	9.11E-01	0.96653
mmu-miR-46	0.00274972	0.086406	-7.268867	9.34E-01	0.97002
mmu-miR-76	0.00276111	0.0839526	-7.269114	9.36E-01	0.97142
mmu-miR-64	0.00282124	0.1258419	-7.263925	9.05E-01	0.96379
mmu-miR-76	0.0029016	0.1377499	-7.262074	8.96E-01	0.95992
mmu-miR-21	0.00294661	0.1329782	-7.262836	8.99E-01	0.96046
mmu-miR-29	0.00296139	0.2074442	-7.247922	8.43E-01	0.9385
mmu-miR-76	0.00297756	0.1546001	-7.259171	8.83E-01	0.95745
mmu-miR-46	0.00299689	0.1374697	-7.262119	8.96E-01	0.95992
mmu-miR-30	0.00320646	0.2359981	-7.2405	8.22E-01	0.92985
mmu-miR-76	0.00323983	0.2227131	-7.24407	8.32E-01	0.93317
mmu-miR-76	0.00338672	0.1857863	-7.252925	8.60E-01	0.94444
mmu-miR-20	0.00342891	0.2731045	-7.229459	7.95E-01	0.92026
mmu-miR-10	0.00343422	0.2443246	-7.238159	8.16E-01	0.92676
mmu-miR-31	0.00344599	0.0920213	-7.268275	9.30E-01	0.96923
mmu-miR-69	0.00344679	0.1346769	-7.262568	8.98E-01	0.95992
mmu-miR-76	0.00355585	0.1968955	-7.250426	8.51E-01	0.94187
mmu-miR-19	0.00359365	0.236409	-7.240386	8.22E-01	0.92985
mmu-miR-7a	0.00361914	0.0967102	-7.267752	9.27E-01	0.96891
mmu-miR-19	0.00368559	0.1792598	-7.254325	8.64E-01	0.94664
mmu-miR-19	0.00371559	0.204152	-7.248718	8.46E-01	0.93864
mmu-miR-20	0.0037627	0.1346745	-7.262568	8.98E-01	0.95992
mmu-miR-18	0.00382371	0.1927743	-7.25137	8.54E-01	0.94268
mmu-miR-30	0.00387546	0.1422209	-7.261336	8.92E-01	0.95992
mmu-miR-30	0.00388538	0.1811964	-7.253915	8.63E-01	0.94601
mmu-miR-70	0.00389276	0.246904	-7.237418	8.14E-01	0.92676
mmu-miR-69	0.00391291	0.2817526	-7.22666	7.89E-01	0.91764
mmu-miR-69	0.00394817	0.1509662	-7.259825	8.86E-01	0.95745
mmu-miR-49	0.00397817	0.2639077	-7.232341	8.02E-01	0.92372
mmu-miR-50	0.00402216	0.2523559	-7.235826	8.10E-01	0.92676
mmu-miR-19	0.00410707	0.2566819	-7.234539	8.07E-01	0.92676
mmu-miR-19	0.0041161	0.2003547	-7.249619	8.49E-01	0.94067
mmu-miR-16	0.00420784	0.1496277	-7.260062	8.87E-01	0.95745
mmu-miR-72	0.00427386	0.254109	-7.235307	8.09E-01	0.92676

mmu-miR-68	0.00436016	0.2594038	-7.233718	8.05E-01	0.92588
mmu-miR-67	0.00437567	0.1714744	-7.255932	8.70E-01	0.9496
mmu-miR-13	0.00438573	0.2903953	-7.223779	7.83E-01	0.91482
mmu-miR-76	0.0043931	0.1619375	-7.257803	8.77E-01	0.9552
mmu-miR-69	0.00446602	0.1896077	-7.252081	8.57E-01	0.94268
mmu-miR-72	0.00461484	0.3554272	-7.199416	7.36E-01	0.89164
mmu-miR-49	0.00467122	0.1433781	-7.261141	8.91E-01	0.95992
mmu-miR-69	0.00467715	0.0888017	-7.268619	9.33E-01	0.9697
mmu-miR-72	0.00470674	0.2941279	-7.222509	7.80E-01	0.91369
mmu-miR-38	0.00474262	0.1933252	-7.251245	8.54E-01	0.94268
mmu-miR-70	0.00477885	0.1932004	-7.251273	8.54E-01	0.94268
mmu-miR-64	0.00481825	0.2858317	-7.225311	7.86E-01	0.91612
mmu-miR-70	0.00488388	0.2584277	-7.234013	8.06E-01	0.9261
mmu-miR-30	0.00500581	0.2436888	-7.23834	8.17E-01	0.92676
mmu-miR-23	0.00509579	0.2447276	-7.238044	8.16E-01	0.92676
mmu-miR-18	0.0051444	0.2536358	-7.235447	8.09E-01	0.92676
mmu-miR-12	0.00528866	0.3865136	-7.186117	7.14E-01	0.88205
mmu-miR-42	0.00537668	0.2529736	-7.235643	8.10E-01	0.92676
mmu-miR-29	0.00547053	0.2924956	-7.223066	7.81E-01	0.91369
mmu-miR-80	0.00550601	0.1011751	-7.26723	9.23E-01	0.96891
mmu-miR-70	0.00558081	0.2044859	-7.248637	8.46E-01	0.93864
mmu-miR-63	0.00566481	0.294443	-7.222401	7.80E-01	0.91369
mmu-miR-69	0.00569117	0.429922	-7.165792	6.84E-01	0.86815
mmu-miR-13	0.00575613	0.1157359	-7.265366	9.12E-01	0.96717
mmu-miR-63	0.00579998	0.3213297	-7.212778	7.60E-01	0.90649
mmu-miR-71	0.00588317	0.4339394	-7.163809	6.81E-01	0.86795
mmu-miR-70	0.00593148	0.3632922	-7.196151	7.30E-01	0.88942
mmu-miR-33	0.00596422	0.1985591	-7.25004	8.50E-01	0.9416
mmu-miR-49	0.0059773	0.1914635	-7.251666	8.55E-01	0.94268
mmu-miR-67	0.0060305	0.4334983	-7.164027	6.82E-01	0.86795
mmu-miR-81	0.00607763	0.2928254	-7.222954	7.81E-01	0.91369
mmu-miR-69	0.00609658	0.335359	-7.207436	7.50E-01	0.89855
mmu-miR-64	0.00612746	0.2848809	-7.225627	7.87E-01	0.91612
mmu-miR-68	0.00615443	0.334083	-7.207931	7.51E-01	0.89855
mmu-miR-10	0.00637619	0.3075113	-7.217825	7.70E-01	0.90921
mmu-miR-30	0.00651086	0.2793607	-7.227443	7.91E-01	0.91854
mmu-miR-30	0.00653891	0.4431512	-7.159196	6.75E-01	0.86547
mmu-miR-13	0.00657455	0.1591334	-7.258333	8.79E-01	0.95653
mmu-miR-69	0.00659831	0.2504569	-7.236384	8.12E-01	0.92676
mmu-miR-37	0.00662518	0.2240763	-7.243713	8.31E-01	0.93317
mmu-miR-14	0.00670021	0.2803337	-7.227125	7.90E-01	0.91828
mmu-miR-30	0.00675831	0.2626627	-7.232724	8.03E-01	0.92421
mmu-miR-12	0.00677723	0.3505354	-7.201412	7.39E-01	0.89413
mmu-miR-19	0.00682016	0.4941567	-7.132044	6.41E-01	0.84191
mmu-miR-30	0.00683437	0.4167418	-7.172177	6.93E-01	0.8734
mmu-miR-80	0.00683925	0.2755163	-7.228687	7.93E-01	0.92026

mmu-let-7e-!	0.0068685	0.2098177	-7.247341	8.42E-01	0.93798
mmu-miR-69	0.00695006	0.3412889	-7.205113	7.46E-01	0.89769
mmu-miR-72	0.0069909	0.343757	-7.204134	7.44E-01	0.89769
mmu-miR-21	0.00708512	0.0347288	-7.272568	9.74E-01	0.9885
mmu-miR-69	0.00733799	0.4222193	-7.169546	6.89E-01	0.87251
mmu-miR-55	0.00744176	0.276585	-7.228342	7.93E-01	0.92026
mmu-miR-70	0.00746453	0.3628503	-7.196336	7.31E-01	0.88942
mmu-miR-10	0.00758188	0.2113996	-7.24695	8.40E-01	0.93798
mmu-miR-76	0.00760538	0.349256	-7.201929	7.40E-01	0.89465
mmu-miR-72	0.00764689	0.3019975	-7.219779	7.74E-01	0.91062
mmu-miR-72	0.00765959	0.2998299	-7.220538	7.76E-01	0.91189
mmu-miR-18	0.00769108	0.3348785	-7.207623	7.51E-01	0.89855
mmu-miR-80	0.00773535	0.510027	-7.123046	6.31E-01	0.83834
mmu-miR-19	0.00789304	0.3846831	-7.186929	7.15E-01	0.88229
mmu-miR-49	0.00794775	0.3059944	-7.218366	7.71E-01	0.90948
mmu-miR-19	0.00807251	0.0407763	-7.272298	9.69E-01	0.98752
mmu-miR-10	0.00820654	0.3123609	-7.216078	7.67E-01	0.90802
mmu-miR-69	0.00822591	0.3655774	-7.19519	7.29E-01	0.88942
mmu-miR-21	0.00825458	0.3344868	-7.207775	7.51E-01	0.89855
mmu-miR-70	0.00826888	0.5000006	-7.128761	6.37E-01	0.84029
mmu-miR-70	0.00834344	0.4115374	-7.174647	6.97E-01	0.8734
mmu-miR-30	0.00849855	0.4443051	-7.158612	6.74E-01	0.86547
mmu-miR-26	0.00855935	0.5541785	-7.096665	6.02E-01	0.82206
mmu-miR-72	0.00857778	0.3404517	-7.205443	7.47E-01	0.89769
mmu-miR-69	0.00859166	0.5313445	-7.110554	6.17E-01	0.83311
mmu-miR-69	0.00863201	0.3070889	-7.217976	7.70E-01	0.90921
mmu-miR-56	0.00866018	0.4012573	-7.179441	7.04E-01	0.8768
mmu-miR-76	0.0086674	0.3989576	-7.180498	7.06E-01	0.87691
mmu-miR-70	0.00867017	0.2875788	-7.224727	7.85E-01	0.91608
mmu-miR-76	0.00868869	0.3757768	-7.190829	7.22E-01	0.88427
mmu-miR-51	0.00869496	0.4119947	-7.174431	6.97E-01	0.8734
mmu-miR-45	0.00870812	0.557877	-7.094366	6.00E-01	0.82137
mmu-miR-69	0.00871839	0.5566421	-7.095135	6.00E-01	0.82137
mmu-miR-38	0.00872573	0.4344569	-7.163552	6.81E-01	0.86795
mmu-miR-50	0.00887376	0.4342394	-7.16366	6.81E-01	0.86795
mmu-miR-69	0.00892144	0.5099586	-7.123085	6.31E-01	0.83834
mmu-miR-51	0.00896279	0.2593541	-7.233733	8.05E-01	0.92588
mmu-miR-63	0.00896781	0.6219722	-7.052402	5.60E-01	0.79873
mmu-miR-18	0.00900242	0.2260609	-7.243189	8.30E-01	0.93317
mmu-miR-66	0.00900394	0.3249205	-7.211432	7.58E-01	0.9041
mmu-miR-34	0.0090702	0.5591927	-7.093545	5.99E-01	0.82137
mmu-miR-64	0.00915472	0.3672403	-7.194487	7.28E-01	0.88942
mmu-miR-29	0.00920742	0.4829412	-7.138247	6.48E-01	0.84652
mmu-miR-31	0.00922759	0.5689595	-7.087397	5.93E-01	0.81866
mmu-miR-1b	0.00930722	0.4864069	-7.136344	6.46E-01	0.84583
mmu-miR-70	0.00931738	0.431121	-7.165202	6.83E-01	0.86815

mmu-miR-13	0.00933385	0.4173378	-7.171893	6.93E-01	0.8734
mmu-miR-34	0.00935204	0.5844916	-7.077427	5.83E-01	0.8138
mmu-miR-69	0.00942561	0.64987	-7.032906	5.43E-01	0.78823
mmu-miR-51	0.00943987	0.4411735	-7.160194	6.77E-01	0.86593
mmu-miR-72	0.00952667	0.6183449	-7.054883	5.62E-01	0.79949
mmu-miR-63	0.00956971	0.4309484	-7.165287	6.84E-01	0.86815
mmu-miR-69	0.00957029	0.5665893	-7.088898	5.94E-01	0.81931
mmu-miR-63	0.00960883	0.4942071	-7.132016	6.41E-01	0.84191
mmu-miR-69	0.00964771	0.6566535	-7.028055	5.39E-01	0.78723
mmu-miR-21	0.00966684	0.4020174	-7.17909	7.03E-01	0.8768
mmu-miR-21	0.00975585	0.3421821	-7.204759	7.45E-01	0.89769
mmu-miR-46	0.00987056	0.3981441	-7.18087	7.06E-01	0.87691
mmu-miR-56	0.00987951	0.6656431	-7.021561	5.34E-01	0.78253
mmu-miR-18	0.01017566	0.4561095	-7.152556	6.66E-01	0.86131
mmu-miR-71	0.01028313	0.4348497	-7.163357	6.81E-01	0.86795
mmu-miR-15	0.01028643	0.3990909	-7.180437	7.05E-01	0.87691
mmu-miR-72	0.01033214	0.5215324	-7.116361	6.23E-01	0.83576
mmu-miR-21	0.01033981	0.5464806	-7.101406	6.07E-01	0.82464
mmu-miR-69	0.01037391	0.8253192	-6.89427	4.45E-01	0.72895
mmu-miR-50	0.01041538	0.4107829	-7.175003	6.97E-01	0.8734
mmu-miR-54	0.01043855	0.6120259	-7.059175	5.66E-01	0.80183
mmu-miR-92	0.01045496	0.7080993	-6.989892	5.09E-01	0.7663
mmu-miR-69	0.01048562	0.4494871	-7.155972	6.71E-01	0.86453
mmu-miR-30	0.01048765	0.3130086	-7.215843	7.66E-01	0.90802
mmu-miR-66	0.01058882	0.5365778	-7.107417	6.13E-01	0.82972
mmu-miR-37	0.01066116	0.6947993	-6.999988	5.17E-01	0.77122
mmu-miR-66	0.01077728	0.4482992	-7.15658	6.72E-01	0.86466
mmu-miR-30	0.01079285	0.3971404	-7.181329	7.07E-01	0.87691
mmu-miR-72	0.01097435	0.8128389	-6.904999	4.51E-01	0.73138
mmu-miR-19	0.01113492	0.492127	-7.133177	6.42E-01	0.84191
mmu-miR-19	0.01113669	0.7546164	-6.953349	4.83E-01	0.74846
mmu-miR-63	0.01116092	0.5509335	-7.098671	6.04E-01	0.82253
mmu-miR-69	0.01124891	0.7523865	-6.955144	4.84E-01	0.74954
mmu-miR-69	0.01128975	0.7545138	-6.953432	4.83E-01	0.74846
mmu-miR-11	0.01137443	0.6277679	-7.048412	5.56E-01	0.79671
mmu-miR-20	0.01139723	0.8269077	-6.892895	4.44E-01	0.72895
mmu-miR-42	0.01163827	0.6265638	-7.049244	5.57E-01	0.79681
mmu-miR-70	0.01192177	0.4150183	-7.172998	6.94E-01	0.8734
mmu-miR-51	0.01201808	0.7055364	-6.99185	5.10E-01	0.76738
mmu-miR-30	0.01228454	0.3419605	-7.204847	7.46E-01	0.89769
mmu-miR-15	0.01238791	0.3609285	-7.197139	7.32E-01	0.88977
mmu-miR-32	0.0124158	0.4703277	-7.145068	6.57E-01	0.85282
mmu-miR-69	0.0124376	0.7388598	-6.965939	4.91E-01	0.75443
mmu-miR-46	0.01244194	0.8055603	-6.911198	4.55E-01	0.73138
mmu-let-7a-	0.01248951	0.5572364	-7.094765	6.00E-01	0.82137
mmu-miR-70	0.01258049	0.8019675	-6.914242	4.57E-01	0.73177

mmu-miR-7b	0.0126541	0.5602611	-7.092877	5.98E-01	0.82137
mmu-miR-70	0.01268789	0.6655492	-7.021629	5.34E-01	0.78253
mmu-miR-68	0.01283218	0.6493926	-7.033246	5.43E-01	0.78823
mmu-miR-70	0.012863	0.7494884	-6.95747	4.86E-01	0.74969
mmu-miR-69	0.01304458	0.8947085	-6.832362	4.10E-01	0.71311
mmu-miR-87	0.01305496	1.1014828	-6.62729	3.18E-01	0.63707
mmu-miR-19	0.01307457	0.7577511	-6.950819	4.81E-01	0.748
mmu-miR-76	0.01312825	0.7947413	-6.920331	4.61E-01	0.73321
mmu-miR-69	0.01317157	0.5853915	-7.076842	5.82E-01	0.8138
mmu-miR-38	0.01317583	0.7113866	-6.987372	5.07E-01	0.7663
mmu-miR-70	0.01318755	0.6578014	-7.02723	5.38E-01	0.78684
mmu-miR-19	0.01323775	0.441472	-7.160044	6.76E-01	0.86593
mmu-miR-62	0.01325029	0.7847757	-6.928658	4.66E-01	0.73679
mmu-miR-12	0.0134252	0.55571	-7.095715	6.01E-01	0.82137
mmu-miR-29	0.01352036	0.6517555	-7.031562	5.42E-01	0.78786
mmu-miR-18	0.01356273	0.9073426	-6.820692	4.04E-01	0.70475
mmu-miR-67	0.01376907	0.8536478	-6.869451	4.30E-01	0.72675
mmu-miR-63	0.01404293	1.1180629	-6.609647	3.12E-01	0.6297
mmu-miR-19	0.01410396	0.7697599	-6.941048	4.74E-01	0.74385
mmu-miR-87	0.01413027	0.7999138	-6.915977	4.58E-01	0.7329
mmu-miR-51	0.01418099	0.4583045	-7.151414	6.65E-01	0.86095
mmu-miR-33	0.01418196	0.2498074	-7.236574	8.12E-01	0.92676
mmu-miR-13	0.0141829	0.6877359	-7.005286	5.21E-01	0.77178
mmu-miR-74	0.01420984	0.9439271	-6.78623	3.86E-01	0.6951
mmu-miR-12	0.0142498	1.0504809	-6.680513	3.39E-01	0.65737
mmu-miR-70	0.01431753	1.1747596	-6.548111	2.90E-01	0.60718
mmu-miR-41	0.01437642	0.5153212	-7.119986	6.27E-01	0.83834
mmu-miR-46	0.01438301	0.8053272	-6.911396	4.55E-01	0.73138
mmu-miR-70	0.01442013	0.8567829	-6.866666	4.29E-01	0.72536
mmu-miR-70	0.01444684	0.7328559	-6.970679	4.95E-01	0.75715
mmu-miR-69	0.01452116	0.9304912	-6.799	3.93E-01	0.69983
mmu-miR-50	0.01453812	0.8034849	-6.912957	4.56E-01	0.73146
mmu-miR-72	0.0147842	0.9572553	-6.773435	3.80E-01	0.69262
mmu-miR-74	0.01479226	0.4571444	-7.152018	6.66E-01	0.86131
mmu-miR-34	0.01483311	0.4514407	-7.154969	6.70E-01	0.86342
mmu-miR-19	0.01484298	0.6621144	-7.024119	5.36E-01	0.7837
mmu-miR-19	0.01485939	0.6536214	-7.030229	5.41E-01	0.78745
mmu-miR-69	0.01489145	0.3977608	-7.181045	7.06E-01	0.87691
mmu-miR-76	0.01493792	0.7500155	-6.957047	4.85E-01	0.74969
mmu-miR-64	0.01501485	0.6948101	-6.99998	5.17E-01	0.77122
mmu-miR-70	0.01502315	0.8494651	-6.873156	4.32E-01	0.72895
mmu-miR-70	0.01507582	0.4789248	-7.140437	6.51E-01	0.84826
mmu-miR-30	0.01509247	0.6442639	-7.036883	5.46E-01	0.79097
mmu-miR-30	0.01511318	0.6161757	-7.056361	5.63E-01	0.80018
mmu-miR-51	0.01521077	0.3655662	-7.195194	7.29E-01	0.88942
mmu-miR-69	0.0152407	0.7726293	-6.938695	4.73E-01	0.74201

mmu-miR-31	0.0152627	0.5955608	-7.070177	5.76E-01	0.80797
mmu-miR-69	0.01537158	0.7159023	-6.983895	5.04E-01	0.76532
mmu-miR-12	0.01538317	0.5840934	-7.077685	5.83E-01	0.8138
mmu-miR-70	0.01539292	1.0772151	-6.652816	3.28E-01	0.64923
mmu-miR-68	0.0154584	0.8722309	-6.852827	4.21E-01	0.71889
mmu-miR-67	0.01548243	0.7353284	-6.968731	4.93E-01	0.75564
mmu-miR-69	0.01551085	0.8915007	-6.835306	4.11E-01	0.71389
mmu-miR-87	0.01567055	0.6945716	-7.00016	5.17E-01	0.77122
mmu-miR-70	0.01571342	0.8765061	-6.848964	4.19E-01	0.71889
mmu-miR-70	0.0157374	0.8226566	-6.89657	4.46E-01	0.72907
mmu-miR-76	0.01582821	0.8037659	-6.912719	4.56E-01	0.73146
mmu-miR-70	0.01585482	0.8349816	-6.885877	4.40E-01	0.72895
mmu-miR-66	0.01594911	0.8147374	-6.903375	4.50E-01	0.73082
mmu-miR-30	0.01605298	0.6329654	-7.044807	5.53E-01	0.79469
mmu-miR-69	0.01609652	0.7198004	-6.980879	5.02E-01	0.76415
mmu-miR-21	0.01622847	0.8148008	-6.903321	4.50E-01	0.73082
mmu-miR-76	0.01632522	0.5186389	-7.118055	6.25E-01	0.8377
mmu-miR-72	0.0163379	0.9633226	-6.767568	3.77E-01	0.69206
mmu-miR-29	0.0163785	0.3893718	-7.184841	7.12E-01	0.88131
mmu-miR-88	0.01638341	0.825743	-6.893903	4.45E-01	0.72895
mmu-miR-14	0.01655913	0.7454179	-6.960725	4.88E-01	0.75124
mmu-miR-69	0.01658021	0.8640855	-6.860147	4.25E-01	0.72035
mmu-miR-30	0.01669101	0.8255032	-6.894111	4.45E-01	0.72895
mmu-miR-71	0.0167115	0.6976186	-6.997861	5.15E-01	0.77122
mmu-miR-14	0.01711579	0.5429623	-7.103553	6.09E-01	0.82593
mmu-miR-13	0.01711747	1.1277132	-6.599303	3.08E-01	0.62514
mmu-miR-87	0.01726106	0.6084683	-7.061574	5.68E-01	0.80204
mmu-miR-72	0.01730473	0.6887644	-7.004517	5.20E-01	0.77178
mmu-miR-17	0.01734425	0.4611335	-7.149934	6.63E-01	0.85907
mmu-miR-19	0.01734608	0.8030591	-6.913318	4.56E-01	0.73146
dmr_308	0.01742748	1.2806325	-6.428593	2.54E-01	0.57311
mmu-miR-69	0.01747274	1.0381669	-6.693119	3.44E-01	0.66453
mmu-miR-36	0.01757317	1.2006293	-6.519441	2.81E-01	0.59826
mmu-miR-63	0.01765857	0.7604735	-6.948614	4.79E-01	0.74751
mmu-miR-70	0.01769084	0.909823	-6.818386	4.02E-01	0.70475
mmu-miR-30	0.01789782	0.9482339	-6.78211	3.84E-01	0.69435
mmu-miR-72	0.01834054	1.2672351	-6.444027	2.58E-01	0.57933
mmu-miR-97	0.01837093	1.0129507	-6.718623	3.55E-01	0.67145
mmu-miR-70	0.01837678	1.2186108	-6.499304	2.75E-01	0.59605
mmu-let-7g-!	0.0185225	0.3554303	-7.199414	7.36E-01	0.89164
mmu-miR-10	0.01856813	1.245165	-6.469263	2.65E-01	0.58563
mmu-miR-97	0.01867973	0.8316828	-6.888751	4.41E-01	0.72895
mmu-miR-54	0.01868545	0.6196968	-7.05396	5.61E-01	0.79891
mmu-miR-69	0.01878375	0.6221203	-7.052301	5.60E-01	0.79873
mmu-miR-19	0.018787	1.0115378	-6.720039	3.56E-01	0.67195
mmu-miR-49	0.01880833	1.1421394	-6.583739	3.02E-01	0.61588

mmu-miR-54	0.01896163	1.2153157	-6.503007	2.76E-01	0.59614
mmu-miR-70	0.01896698	0.398386	-7.18076	7.06E-01	0.87691
mmu-miR-30	0.01920983	0.8316348	-6.888792	4.42E-01	0.72895
mmu-miR-13	0.01929865	1.0199497	-6.711586	3.52E-01	0.6697
mmu-miR-99	0.0195934	0.5038227	-7.126594	6.35E-01	0.83905
mmu-miR-13	0.01974352	0.9728953	-6.758259	3.73E-01	0.68711
mmu-miR-63	0.01982467	0.5556502	-7.095752	6.01E-01	0.82137
mmu-miR-63	0.01985168	0.808025	-6.909104	4.54E-01	0.73138
mmu-miR-18	0.01989409	1.0505652	-6.680427	3.39E-01	0.65737
mmu-miR-30	0.01990366	1.3076337	-6.397231	2.45E-01	0.56747
mmu-miR-18	0.01998346	0.6891562	-7.004224	5.20E-01	0.77178
mmu-miR-69	0.02005723	1.1335959	-6.592971	3.06E-01	0.6212
mmu-miR-20	0.02025368	0.7156466	-6.984092	5.05E-01	0.76532
mmu-miR-46	0.02026533	0.9907501	-6.740726	3.65E-01	0.67898
mmu-miR-70	0.02034828	1.6514807	-5.973244	1.56E-01	0.45534
mmu-miR-56	0.02036857	0.9110939	-6.817203	4.02E-01	0.70458
mmu-miR-21	0.02062907	1.0319684	-6.699427	3.47E-01	0.66519
mmu-miR-76	0.02066359	0.6552615	-7.029054	5.40E-01	0.78734
mmu-miR-53	0.0208907	1.1916836	-6.529396	2.84E-01	0.60192
mmu-miR-29	0.02094771	0.5022255	-7.127502	6.36E-01	0.83933
mmu-miR-31	0.02096771	1.1498443	-6.575378	3.00E-01	0.61381
mmu-miR-69	0.02103462	1.1703193	-6.552996	2.92E-01	0.60718
mmu-miR-19	0.02118818	1.1884746	-6.532957	2.85E-01	0.60192
mmu-miR-70	0.02131888	1.0167575	-6.714799	3.53E-01	0.67099
mmu-miR-70	0.02143635	1.5890479	-6.053061	1.70E-01	0.47964
mmu-miR-38	0.02146317	1.2753185	-6.434725	2.55E-01	0.57471
mmu-miR-69	0.02146666	0.8101448	-6.907299	4.53E-01	0.73138
mmu-miR-64	0.02162882	0.8696493	-6.855153	4.22E-01	0.71889
mmu-miR-88	0.0216867	1.4489372	-6.22801	2.04E-01	0.52625
mmu-miR-63	0.02182889	1.6592049	-5.9633	1.55E-01	0.45421
mmu-miR-72	0.02193335	1.4094953	-6.276049	2.15E-01	0.53839
mmu-miR-66	0.0220352	0.9646191	-6.766311	3.77E-01	0.69164
mmu-miR-30	0.02224199	0.2066274	-7.248121	8.44E-01	0.9385
mmu-miR-64	0.0222702	1.2948484	-6.412124	2.49E-01	0.5706
mmu-miR-76	0.022359	0.4084698	-7.17609	6.99E-01	0.87402
mmu-miR-66	0.02240348	0.5909379	-7.07322	5.79E-01	0.81141
mmu-miR-34	0.02247548	1.1523736	-6.572625	2.99E-01	0.61324
mmu-miR-72	0.02249463	1.5132277	-6.148516	1.87E-01	0.51079
mmu-miR-31	0.02255667	0.0994798	-7.267431	9.24E-01	0.96891
mmu-miR-75	0.02260447	0.7484128	-6.958331	4.86E-01	0.74978
mmu-miR-81	0.02270994	0.8854237	-6.840862	4.14E-01	0.7159
mmu-miR-69	0.0228604	1.2159096	-6.50234	2.75E-01	0.59614
mmu-miR-69	0.02290471	1.1569022	-6.567689	2.97E-01	0.6111
mmu-miR-63	0.02291863	1.4756608	-6.195139	1.97E-01	0.51845
mmu-miR-20	0.02301569	1.3712871	-6.322012	2.26E-01	0.5452
mmu-miR-81	0.0230296	0.9344125	-6.795287	3.91E-01	0.6985

mmu-miR-71	0.023326	1.6539766	-5.970032	1.56E-01	0.45454
mmu-miR-20	0.0234824	1.2065446	-6.512836	2.79E-01	0.59763
mmu-miR-69	0.02348599	0.6358412	-7.042802	5.51E-01	0.79451
mmu-miR-63	0.0237339	1.5463156	-6.107073	1.80E-01	0.49844
mmu-miR-30	0.02377515	1.5228345	-6.136519	1.85E-01	0.50957
mmu-miR-67	0.02385921	1.0881095	-6.641401	3.24E-01	0.64316
mmu-miR-30	0.02410546	1.8578774	-5.703241	1.19E-01	0.39357
mmu-miR-18	0.02420861	1.4916559	-6.175346	1.93E-01	0.51559
mmu-miR-76	0.02449308	1.1238274	-6.603475	3.09E-01	0.62619
mmu-miR-69	0.02460326	0.880466	-6.845374	4.17E-01	0.71808
mmu-miR-69	0.02469482	1.2610589	-6.451113	2.60E-01	0.58119
mmu-miR-69	0.02490417	1.0562784	-6.674545	3.37E-01	0.65737
mmu-miR-66	0.02508275	1.592733	-6.048379	1.69E-01	0.47803
mmu-miR-66	0.02520907	1.4450475	-6.232773	2.05E-01	0.52679
mmu-miR-93	0.02525476	0.8707481	-6.854163	4.22E-01	0.71889
mmu-miR-30	0.0257477	0.9761655	-6.755064	3.71E-01	0.68552
mmu-miR-48	0.0257687	0.8436145	-6.878314	4.35E-01	0.72895
mmu-miR-12	0.02588741	0.7277393	-6.974694	4.98E-01	0.76033
mmu-miR-76	0.02596488	1.8943828	-5.654724	1.14E-01	0.38343
mmu-miR-69	0.02596999	1.2414386	-6.4735	2.67E-01	0.58563
mmu-miR-69	0.02600158	1.222781	-6.49461	2.73E-01	0.59462
mmu-miR-69	0.02600737	2.004892	-5.506876	9.82E-02	0.36043
mmu-miR-69	0.0260265	2.0335291	-5.468369	9.46E-02	0.35128
mmu-miR-69	0.02603018	1.654688	-5.969117	1.56E-01	0.45454
mmu-miR-70	0.02610908	1.6046671	-6.03319	1.66E-01	0.47219
mmu-miR-70	0.02628757	0.8634075	-6.860754	4.25E-01	0.72035
mmu-miR-18	0.02650221	0.9068798	-6.821121	4.04E-01	0.70475
mmu-miR-19	0.02654711	0.8391655	-6.882219	4.38E-01	0.72895
mmu-miR-70	0.02666436	1.2983214	-6.408086	2.48E-01	0.56943
mmu-miR-54	0.02677684	1.0304044	-6.701014	3.48E-01	0.66574
mmu-miR-35	0.02703983	0.893566	-6.833412	4.10E-01	0.71343
mmu-miR-65	0.02710864	2.3617419	-5.024481	6.18E-02	0.28131
mmu-miR-32	0.02714528	2.0695987	-5.419781	9.02E-02	0.34382
mmu-miR-38	0.02729243	1.1646001	-6.559271	2.94E-01	0.60823
mmu-miR-69	0.02773849	1.2793288	-6.430099	2.54E-01	0.57311
mmu-miR-69	0.02783194	1.0745254	-6.655623	3.29E-01	0.65063
mmu-miR-70	0.02786179	1.1640296	-6.559896	2.94E-01	0.60823
mmu-miR-68	0.0281822	0.5078124	-7.124317	6.32E-01	0.83834
mmu-miR-30	0.02826525	1.951835	-5.578024	1.05E-01	0.37363
mmu-miR-70	0.02830557	1.6917885	-5.921196	1.48E-01	0.44125
mmu-miR-20	0.0284452	1.2126934	-6.50595	2.77E-01	0.59647
mmu-miR-46	0.02868776	0.7508942	-6.956342	4.85E-01	0.74969
mmu-miR-63	0.02873576	1.2894671	-6.418369	2.51E-01	0.57136
mmu-miR-57	0.02873773	0.8363488	-6.884683	4.39E-01	0.72895
mmu-miR-51	0.02918456	1.389434	-6.300255	2.20E-01	0.5409
dmr_316	0.0292168	0.9275368	-6.801791	3.94E-01	0.70166

mmu-miR-69	0.02921955	0.7543563	-6.953558	4.83E-01	0.74846
mmu-miR-66	0.02927849	1.5702533	-6.076882	1.74E-01	0.48801
mmu-miR-20	0.02955738	0.9897728	-6.741692	3.65E-01	0.67898
mmu-miR-19	0.02961794	1.5614387	-6.088019	1.76E-01	0.4908
mmu-miR-39	0.029621	1.1530069	-6.571936	2.98E-01	0.61324
mmu-miR-66	0.02964078	1.352668	-6.344194	2.31E-01	0.55391
mmu-miR-76	0.02966246	1.5214091	-6.138301	1.85E-01	0.50979
mmu-miR-20	0.02976263	0.8293662	-6.890764	4.43E-01	0.72895
mmu-miR-42	0.02977998	0.9069945	-6.821015	4.04E-01	0.70475
mmu-miR-70	0.02980513	1.7128675	-5.893831	1.44E-01	0.43598
mmu-miR-69	0.02987761	1.7068053	-5.901711	1.45E-01	0.43602
mmu-miR-21	0.02994503	0.9981752	-6.733371	3.62E-01	0.67898
mmu-miR-56	0.02997774	1.2623761	-6.449604	2.60E-01	0.58089
mmu-miR-64	0.03007498	1.8852881	-5.666828	1.15E-01	0.38672
mmu-miR-63	0.0302383	0.5166135	-7.119235	6.26E-01	0.83829
mmu-miR-29	0.03035754	1.9444902	-5.587851	1.06E-01	0.37363
mmu-miR-70	0.03035756	1.6332353	-5.996675	1.60E-01	0.46319
mmu-miR-70	0.03038	1.4718768	-6.199809	1.98E-01	0.51936
mmu-miR-30	0.03041003	2.3907058	-4.985279	5.96E-02	0.27989
mmu-miR-51	0.03063849	1.2104041	-6.508516	2.77E-01	0.59686
mmu-miR-65	0.03103571	1.7111643	-5.896046	1.45E-01	0.43602
mmu-miR-50	0.03113373	1.6315658	-5.998814	1.60E-01	0.46319
mmu-miR-37	0.03114862	1.0664944	-6.663977	3.32E-01	0.65453
mmu-miR-19	0.03121726	2.227522	-5.206275	7.35E-02	0.30842
mmu-miR-87	0.03122494	1.4546287	-6.221031	2.02E-01	0.52307
mmu-miR-66	0.03124291	2.1102176	-5.364966	8.55E-02	0.33316
mmu-miR-34	0.031423	2.2665523	-5.153407	6.98E-02	0.29878
mmu-miR-37	0.03161716	0.4546298	-7.153324	6.67E-01	0.86181
mmu-miR-18	0.03164362	2.0035163	-5.508724	9.84E-02	0.36043
mmu-miR-63	0.03166284	2.0958537	-5.38436	8.72E-02	0.33696
mmu-miR-80	0.03174913	0.6357933	-7.042835	5.51E-01	0.79451
mmu-miR-44	0.03212202	1.7858415	-5.798389	1.31E-01	0.41307
mmu-miR-76	0.03223103	0.8269229	-6.892882	4.44E-01	0.72895
mmu-miR-63	0.03231094	1.3926418	-6.296395	2.19E-01	0.5409
mmu-miR-51	0.03246459	1.3113795	-6.392854	2.44E-01	0.56747
mmu-miR-70	0.03258057	2.1141165	-5.359699	8.51E-02	0.33241
mmu-miR-43	0.03310258	0.7554674	-6.952663	4.82E-01	0.74846
mmu-miR-13	0.03320162	2.0768604	-5.409988	8.94E-02	0.34263
mmu-miR-70	0.03375447	1.2809934	-6.428176	2.53E-01	0.57311
mmu-miR-29	0.03402304	1.2968534	-6.409793	2.48E-01	0.56981
mmu-miR-70	0.03427884	0.7935995	-6.92129	4.62E-01	0.73321
mmu-miR-19	0.03440581	1.2428329	-6.471916	2.66E-01	0.58563
mmu-miR-76	0.03440871	1.6304063	-6.0003	1.61E-01	0.46319
mmu-miR-69	0.03453983	1.7563762	-5.837051	1.36E-01	0.4245
mmu-miR-72	0.03471297	1.0943444	-6.634836	3.21E-01	0.63933
mmu-miR-63	0.03545725	1.7074175	-5.900915	1.45E-01	0.43602

mmu-miR-76	0.03559866	1.4397331	-6.239272	2.06E-01	0.52923
mmu-miR-30	0.03566183	1.3124225	-6.391634	2.43E-01	0.56747
mmu-miR-36	0.03569529	1.2817177	-6.427339	2.53E-01	0.57311
mmu-miR-36	0.03573383	2.9108401	-4.289729	3.12E-02	0.19655
mmu-miR-43	0.03584924	1.7320107	-5.868896	1.41E-01	0.4306
mmu-miR-30	0.03600684	1.6762239	-5.941339	1.51E-01	0.44731
mmu-miR-54	0.0361985	1.8286478	-5.741949	1.24E-01	0.40105
mmu-miR-11	0.03625004	1.7376929	-5.86148	1.40E-01	0.42876
mmu-miR-65	0.03628599	1.5667552	-6.081305	1.75E-01	0.48927
mmu-miR-66	0.03634391	1.0134981	-6.718074	3.55E-01	0.67145
mmu-miR-70	0.03643366	2.345639	-5.046283	6.31E-02	0.28436
mmu-miR-13	0.03651432	1.5361016	-6.119902	1.82E-01	0.50401
mmu-miR-49	0.03654662	0.9558439	-6.774796	3.81E-01	0.69262
mmu-miR-11	0.03655461	0.576144	-7.082815	5.88E-01	0.81656
mmu-miR-56	0.03658407	1.8965554	-5.651831	1.13E-01	0.38343
mmu-miR-69	0.03663492	1.3500308	-6.347324	2.32E-01	0.55448
mmu-miR-59	0.03700915	1.9684746	-5.555741	1.03E-01	0.36897
mmu-miR-41	0.0372147	0.3177306	-7.214113	7.63E-01	0.90649
mmu-miR-38	0.03738956	0.7975832	-6.917942	4.59E-01	0.73321
mmu-miR-72	0.03765771	3.3379109	-3.739732	1.89E-02	0.1505
mmu-miR-63	0.03766511	1.2253082	-6.491761	2.72E-01	0.5937
mmu-miR-49	0.03797996	2.4250223	-4.938863	5.70E-02	0.27452
mmu-miR-14	0.03817483	2.4376059	-4.921853	5.61E-02	0.27153
mmu-miR-31	0.03880741	2.0521452	-5.443304	9.23E-02	0.34757
mmu-miR-64	0.03890632	2.0673603	-5.422799	9.05E-02	0.34414
mmu-miR-70	0.03894839	0.3794257	-7.189242	7.19E-01	0.88339
mmu-miR-56	0.03897068	2.4323732	-4.928925	5.65E-02	0.27265
mmu-miR-18	0.0389718	0.8252383	-6.89434	4.45E-01	0.72895
mmu-miR-33	0.03916816	2.1517362	-5.308851	8.10E-02	0.3229
mmu-miR-69	0.03917545	2.2080194	-5.232684	7.53E-02	0.30918
mmu-miR-66	0.03933882	1.4766906	-6.193867	1.97E-01	0.51845
mmu-miR-70	0.03939961	2.1854928	-5.26318	7.76E-02	0.3139
mmu-miR-69	0.03941561	2.0049636	-5.50678	9.82E-02	0.36043
mmu-miR-87	0.03942546	1.8581634	-5.702862	1.19E-01	0.39357
mmu-miR-46	0.03942819	3.3898043	-3.674574	1.78E-02	0.14617
mmu-miR-70	0.03961083	2.3641168	-5.021265	6.16E-02	0.28131
mmu-miR-30	0.0396466	1.7539011	-5.840291	1.37E-01	0.4245
mmu-miR-27	0.03977344	0.6841712	-7.007942	5.23E-01	0.77305
mmu-miR-43	0.03987966	2.1384442	-5.326824	8.25E-02	0.32538
mmu-miR-69	0.04003274	2.019993	-5.486579	9.63E-02	0.35567
mmu-miR-63	0.04022815	1.7425278	-5.855165	1.39E-01	0.42673
mmu-miR-56	0.04046422	1.5345244	-6.121881	1.82E-01	0.50401
mmu-miR-63	0.0406602	2.2350274	-5.19611	7.27E-02	0.3068
mmu-miR-69	0.04067131	1.4850955	-6.183475	1.95E-01	0.51559
mmu-let-7d-:	0.04072827	1.0999338	-6.62893	3.19E-01	0.63707
mmu-miR-32	0.04084979	1.8293921	-5.740965	1.24E-01	0.40105

mmu-miR-70	0.04092591	3.2485297	-3.852851	2.10E-02	0.16004
mmu-miR-64	0.0409814	2.0642954	-5.42693	9.08E-02	0.34483
mmu-miR-70	0.04167385	1.3265032	-6.375117	2.39E-01	0.5625
mmu-miR-46	0.04222223	1.9705003	-5.553026	1.03E-01	0.36897
mmu-miR-17	0.04237112	0.8304771	-6.889799	4.42E-01	0.72895
mmu-miR-69	0.04249357	1.8707072	-5.686211	1.17E-01	0.39144
mmu-miR-46	0.04327633	1.4760952	-6.194603	1.97E-01	0.51845
mmu-miR-36	0.04382128	3.2221085	-3.8865	2.16E-02	0.16369
mmu-miR-70	0.0438965	2.2098748	-5.230172	7.52E-02	0.30918
mmu-miR-65	0.04397614	1.1999849	-6.52016	2.81E-01	0.59826
mmu-miR-72	0.04416717	1.8554009	-5.706526	1.19E-01	0.39388
mmu-miR-69	0.0442177	3.4313752	-3.622657	1.70E-02	0.14381
mmu-miR-63	0.04464283	1.2496922	-6.464106	2.64E-01	0.58557
mmu-miR-19	0.04470188	0.975999	-6.755227	3.72E-01	0.68552
mmu-miR-63	0.04502121	1.1716162	-6.55157	2.91E-01	0.60718
mmu-miR-69	0.04503188	2.6846227	-4.589649	4.11E-02	0.22884
mmu-miR-69	0.04523241	2.3159765	-5.086456	6.55E-02	0.28795
mmu-miR-69	0.04559688	2.5868758	-4.720641	4.65E-02	0.2483
mmu-miR-63	0.04600484	1.4906219	-6.176628	1.93E-01	0.51559
mmu-miR-37	0.04608306	0.7253415	-6.976568	4.99E-01	0.76057
mmu-miR-69	0.04627323	2.3329749	-5.063433	6.41E-02	0.28617
mmu-miR-35	0.04680624	0.7378668	-6.966725	4.92E-01	0.75467
mmu-miR-19	0.0470312	2.8497521	-4.370215	3.36E-02	0.20726
mmu-miR-20	0.04708754	1.4892445	-6.178335	1.93E-01	0.51559
mmu-miR-51	0.04713748	0.4035882	-7.178364	7.02E-01	0.87651
mmu-miR-63	0.04725899	2.746537	-4.507075	3.81E-02	0.22038
mmu-miR-51	0.04742286	2.4791209	-4.865778	5.32E-02	0.2632
mmu-miR-81	0.04769512	2.4222647	-4.942591	5.72E-02	0.27479
mmu-miR-62	0.04815407	3.0045651	-4.167057	2.79E-02	0.18689
mmu-miR-34	0.04831409	2.699198	-4.57018	4.04E-02	0.22696
mmu-miR-76	0.04847571	2.2766204	-5.139768	6.89E-02	0.29668
mmu-miR-19	0.04859067	2.3829464	-4.995779	6.02E-02	0.2811
hur_6	0.04870501	0.9657894	-6.765175	3.76E-01	0.69132
mmu-miR-76	0.04898112	2.6984189	-4.57122	4.05E-02	0.22696
mmu-miR-69	0.04923741	1.397234	-6.290862	2.18E-01	0.5409
mmu-miR-66	0.04925901	1.4451497	-6.232648	2.05E-01	0.52679
mmu-miR-34	0.04995211	1.6236977	-6.008889	1.62E-01	0.46472
mmu-miR-33	0.05026513	0.2831357	-7.226205	7.88E-01	0.91703
mmu-miR-37	0.0504371	0.3753011	-7.191035	7.22E-01	0.88427
mmu-miR-71	0.05103542	2.5059039	-4.829643	5.15E-02	0.26029
mmu-miR-71	0.05104733	2.9613298	-4.223519	2.94E-02	0.1906
mmu-miR-27	0.05128578	1.0261547	-6.70532	3.49E-01	0.66594
mmu-miR-67	0.05214919	2.9282657	-4.266845	3.06E-02	0.19375
mmu-miR-28	0.05229653	1.4046135	-6.281954	2.16E-01	0.53932
mmu-miR-92	0.05255857	1.9682442	-5.556049	1.03E-01	0.36897
mmu-miR-64	0.05302731	3.155502	-3.971748	2.33E-02	0.1741

mmu-miR-19	0.05340007	3.0301841	-4.133706	2.71E-02	0.1851
mmu-miR-69	0.05343145	2.0525019	-5.442823	9.22E-02	0.34757
mmu-miR-46	0.05351601	1.3951148	-6.293416	2.19E-01	0.5409
mmu-miR-30	0.05360495	3.5682149	-3.453547	1.46E-02	0.1323
mmu-miR-72	0.05433954	2.4381957	-4.921055	5.61E-02	0.27153
mmu-let-7e-:	0.05461366	1.7811126	-5.804605	1.32E-01	0.41428
mmu-miR-19	0.05461971	2.4781182	-4.867131	5.33E-02	0.2632
mmu-miR-32	0.05516266	2.5472793	-4.773896	4.88E-02	0.25511
mmu-miR-76	0.05555093	1.9214652	-5.618617	1.10E-01	0.37812
mmu-miR-18	0.05591638	1.4043313	-6.282295	2.16E-01	0.53932
mmu-miR-14	0.05619115	1.0091014	-6.722479	3.57E-01	0.67329
mmu-miR-12	0.05651819	0.8370002	-6.884114	4.39E-01	0.72895
mmu-miR-19	0.05656158	1.1843378	-6.537538	2.87E-01	0.60196
mmu-miR-69	0.05657492	2.3409888	-5.05258	6.35E-02	0.28539
mmu-miR-19	0.05658984	3.5896492	-3.42731	1.43E-02	0.13129
mmu-miR-70	0.05717212	2.0438568	-5.454467	9.33E-02	0.34998
mmu-miR-70	0.05789282	2.6568321	-4.626817	4.26E-02	0.23429
mmu-miR-70	0.05821869	1.4662286	-6.20677	1.99E-01	0.51936
mmu-miR-70	0.05888812	3.2494896	-3.85163	2.09E-02	0.16004
mmu-miR-19	0.06005359	3.7558016	-3.226281	1.19E-02	0.12041
mmu-miR-53	0.06060083	1.3522206	-6.344726	2.31E-01	0.55391
mmu-miR-69	0.06098484	0.2229693	-7.244003	8.32E-01	0.93317
mmu-miR-69	0.06217727	1.6311674	-5.999325	1.61E-01	0.46319
mmu-miR-33	0.06226801	1.9732228	-5.549377	1.02E-01	0.36897
mmu-miR-26	0.06227978	2.7207929	-4.541369	3.93E-02	0.2228
mmu-miR-46	0.06296958	1.9948238	-5.520398	9.95E-02	0.36247
mmu-miR-70	0.06298082	1.8947558	-5.654228	1.13E-01	0.38343
mmu-miR-13	0.06366248	2.210418	-5.229437	7.51E-02	0.30918
mmu-miR-32	0.06479462	1.479229	-6.190731	1.96E-01	0.51845
mmu-miR-70	0.06484319	3.7888285	-3.186822	1.15E-02	0.11883
mmu-miR-29	0.06487982	1.1466901	-6.578805	3.01E-01	0.61433
mmu-miR-81	0.06494838	2.5117643	-4.821742	5.11E-02	0.25975
mmu-miR-76	0.06496224	2.3596375	-5.027329	6.20E-02	0.28131
mmu-miR-10	0.06506287	2.217911	-5.21929	7.44E-02	0.30918
mmu-miR-70	0.06734595	3.3686089	-3.701141	1.83E-02	0.14718
mmu-miR-18	0.0675308	0.6755076	-7.014349	5.28E-01	0.77812
mmu-miR-64	0.06790476	2.7211206	-4.540932	3.93E-02	0.2228
mmu-miR-30	0.06796456	2.1092289	-5.366301	8.57E-02	0.33316
mmu-miR-98	0.06810048	0.5655555	-7.089551	5.95E-01	0.81931
mmu-miR-65	0.06849042	2.5589459	-4.758195	4.81E-02	0.25219
mmu-miR-69	0.06868199	1.5158996	-6.145182	1.87E-01	0.51079
mmu-miR-69	0.06929045	3.6060694	-3.407258	1.40E-02	0.13
mmu-miR-31	0.07087929	2.7937179	-4.444385	3.60E-02	0.21444
mmu-miR-30	0.07103648	2.3714011	-5.011405	6.10E-02	0.28131
mmu-miR-69	0.0712357	3.6321013	-3.37555	1.36E-02	0.12755
mmu-miR-69	0.07138851	3.0219041	-4.144476	2.73E-02	0.18559

mmu-miR-10	0.07203126	2.3681076	-5.015863	6.13E-02	0.28131
mmu-miR-13	0.07206018	1.2311551	-6.485157	2.70E-01	0.59201
mmu-miR-69	0.07271785	2.3278766	-5.070338	6.45E-02	0.28617
mmu-miR-66	0.07306067	2.3660482	-5.018651	6.15E-02	0.28131
mmu-miR-81	0.07368699	1.037091	-6.694215	3.45E-01	0.66453
mmu-miR-70	0.07371345	2.4900497	-4.851029	5.25E-02	0.2632
mmu-miR-29	0.07495233	3.1232187	-4.013278	2.42E-02	0.17665
mmu-miR-29	0.0755515	3.8432526	-3.122164	1.08E-02	0.11527
mmu-miR-33	0.07759692	2.3552681	-5.033245	6.23E-02	0.28155
mmu-miR-36	0.07814422	2.5247385	-4.804255	5.02E-02	0.25829
mmu-miR-69	0.07921047	3.8524355	-3.111299	1.07E-02	0.11479
mmu-miR-70	0.07962849	2.6259755	-4.668158	4.43E-02	0.23924
mmu-miR-70	0.07987024	0.9902754	-6.741195	3.65E-01	0.67898
mmu-miR-80	0.08011002	4.0051742	-2.932484	9.15E-03	0.10243
mmu-miR-70	0.08086761	3.3135449	-3.770458	1.94E-02	0.15234
mmu-miR-34	0.08090188	0.3620013	-7.196691	7.31E-01	0.88942
mmu-miR-22	0.08180684	2.7274244	-4.532529	3.90E-02	0.2228
mmu-miR-69	0.08273772	2.845974	-4.375206	3.38E-02	0.20726
mmu-miR-68	0.08305454	4.3431138	-2.549592	6.51E-03	0.08361
mmu-miR-41	0.0837655	1.4329147	-6.247595	2.08E-01	0.53133
mmu-miR-19	0.08537186	1.4678593	-6.204761	1.99E-01	0.51936
mmu-miR-69	0.08599534	5.7633054	-1.121922	1.83E-03	0.04448
mmu-miR-69	0.08611477	4.6607162	-2.205518	4.79E-03	0.07142
mmu-miR-66	0.08626223	1.8078545	-5.769404	1.27E-01	0.40597
mmu-miR-12	0.08838579	0.8700779	-6.854767	4.22E-01	0.71889
mmu-miR-34	0.09032242	0.190154	-7.251959	8.56E-01	0.94268
mmu-miR-67	0.09122478	2.616346	-4.681073	4.48E-02	0.24136
mmu-miR-69	0.09215587	3.6279189	-3.380637	1.37E-02	0.12755
mmu-miR-19	0.09256374	1.4859427	-6.182426	1.94E-01	0.51559
mmu-miR-32	0.09368373	5.2279748	-1.627435	2.87E-03	0.058
mmu-miR-37	0.0946311	2.5370539	-4.787664	4.95E-02	0.2564
mmu-miR-63	0.09516926	2.3069411	-5.098695	6.63E-02	0.28859
mmu-miR-99	0.0960514	1.412193	-6.272782	2.14E-01	0.53748
mmu-let-7c-!	0.09765773	2.4764929	-4.869325	5.34E-02	0.2632
mmu-miR-30	0.09766994	3.1307447	-4.003584	2.40E-02	0.17645
mmu-miR-63	0.09779759	3.7523932	-3.230363	1.20E-02	0.12041
mmu-miR-70	0.09786356	0.523352	-7.115291	6.22E-01	0.83535
mmu-miR-76	0.09808446	4.4540068	-2.42774	5.84E-03	0.07784
mmu-miR-21	0.0983834	1.2050726	-6.514481	2.79E-01	0.59763
mmu-miR-72	0.09876994	2.8999415	-4.304059	3.16E-02	0.19849
mmu-miR-69	0.09978241	4.4458151	-2.436678	5.88E-03	0.07784
mmu-miR-51	0.09983297	4.023017	-2.91183	8.99E-03	0.10115
mmu-miR-19	0.10264672	3.2446286	-3.857813	2.10E-02	0.16012
mmu-miR-39	0.10275518	1.0937463	-6.635467	3.21E-01	0.63933
mmu-miR-70	0.10277212	3.3782149	-3.689092	1.81E-02	0.14683
mmu-miR-46	0.1033946	1.5828364	-6.060945	1.71E-01	0.48214

mmu-miR-70	0.10376354	1.8974946	-5.65058	1.13E-01	0.38343
mmu-miR-69	0.10439828	2.2013334	-5.241737	7.60E-02	0.31016
mmu-miR-65	0.10450395	3.3977876	-3.664584	1.77E-02	0.14548
mmu-miR-11	0.10471049	6.1311701	-0.795291	1.37E-03	0.0413
mmu-miR-12	0.10518461	1.007904	-6.723677	3.57E-01	0.67362
mmu-miR-70	0.10595078	2.0506453	-5.445324	9.25E-02	0.34757
mmu-miR-21	0.10619561	1.2522726	-6.461162	2.63E-01	0.58433
mmu-miR-19	0.10671624	1.2630312	-6.448852	2.59E-01	0.58089
mmu-miR-69	0.10789038	1.0595265	-6.671192	3.35E-01	0.65682
mmu-miR-34	0.10876619	4.8189258	-2.039684	4.13E-03	0.06778
mmu-miR-70	0.10973299	3.1516639	-3.976679	2.34E-02	0.1742
mmu-miR-49	0.11293401	1.503369	-6.160797	1.90E-01	0.51381
mmu-miR-59	0.11516312	2.1388507	-5.326274	8.24E-02	0.32538
mmu-miR-37	0.11571875	1.2892744	-6.418593	2.51E-01	0.57136
mmu-miR-65	0.11634602	1.5715081	-6.075295	1.74E-01	0.48792
mmu-miR-69	0.11708881	3.476703	-3.566334	1.62E-02	0.14108
mmu-miR-70	0.11786758	5.1265643	-1.727468	3.13E-03	0.0593
mmu-miR-70	0.11831576	3.7427826	-3.241882	1.21E-02	0.1209
mmu-miR-70	0.11881232	3.8173888	-3.152834	1.12E-02	0.11717
mmu-miR-63	0.11968857	0.2441059	-7.238221	8.16E-01	0.92676
mmu-miR-10	0.12009589	2.9702789	-4.211814	2.91E-02	0.18992
mmu-miR-13	0.12244045	2.4131714	-4.954888	5.79E-02	0.27589
mmu-miR-49	0.12388077	1.8817946	-5.671475	1.15E-01	0.38782
mmu-miR-30	0.12607763	3.4099597	-3.649371	1.74E-02	0.14412
mmu-miR-76	0.12718706	0.6934119	-7.001032	5.17E-01	0.77122
mmu-miR-70	0.12812748	0.8378698	-6.883353	4.38E-01	0.72895
mmu-miR-29	0.12821054	2.2225589	-5.212996	7.39E-02	0.30918
mmu-let-7a-!	0.12867799	3.0328718	-4.130212	2.70E-02	0.1851
mmu-miR-99	0.1292473	1.3910702	-6.298286	2.20E-01	0.5409
mmu-miR-76	0.12979963	5.1245411	-1.729478	3.14E-03	0.0593
mmu-miR-20	0.13071185	2.2415733	-5.187244	7.21E-02	0.30557
mmu-miR-19	0.13076338	1.3679205	-6.326033	2.27E-01	0.54689
mmu-miR-69	0.13113353	1.7209505	-5.883312	1.43E-01	0.43296
mmu-let-7f-5	0.13730036	2.2658911	-5.154303	6.99E-02	0.29878
mmu-miR-69	0.13772158	6.3151301	-0.637885	1.19E-03	0.03901
mmu-miR-80	0.13840993	2.6851603	-4.58893	4.11E-02	0.22884
mmu-miR-13	0.13844187	5.4718474	-1.392535	2.33E-03	0.04917
mmu-miR-76	0.14030792	4.283313	-2.616076	6.90E-03	0.08637
mmu-miR-69	0.14093495	2.369155	-5.014445	6.12E-02	0.28131
mmu-miR-76	0.14454906	6.7577317	-0.274414	8.60E-04	0.03208
mmu-miR-41	0.14468641	1.4380993	-6.241268	2.07E-01	0.52943
mmu-miR-69	0.14862751	4.7678388	-2.092835	4.33E-03	0.06784
mmu-miR-19	0.14872951	4.744251	-2.117504	4.43E-03	0.0685
mmu-miR-70	0.14922065	9.4195306	1.538158	1.67E-04	0.01216
mmu-miR-76	0.14947683	6.1514785	-0.777724	1.35E-03	0.0413
mmu-miR-69	0.15036123	7.1653687	0.042483	6.48E-04	0.02625

mmu-miR-46	0.15169852	2.9951229	-4.179369	2.82E-02	0.18825
mmu-miR-69	0.15245038	1.1704381	-6.552865	2.92E-01	0.60718
mmu-miR-34	0.15431284	1.4204416	-6.262776	2.12E-01	0.53549
mmu-miR-46	0.15468152	1.3884561	-6.30143	2.21E-01	0.5409
mmu-miR-76	0.15475153	0.9466326	-6.783643	3.85E-01	0.69435
mmu-miR-69	0.15503672	4.0945009	-2.829572	8.35E-03	0.09626
mmu-miR-70	0.15949539	3.9722374	-2.97074	9.47E-03	0.10476
mmu-miR-18	0.16042049	1.0347188	-6.696631	3.46E-01	0.66495
mmu-miR-69	0.16230419	5.9962868	-0.913182	1.52E-03	0.0413
mmu-miR-81	0.16344285	2.0212995	-5.484822	9.61E-02	0.35567
mmu-miR-10	0.16440525	4.415321	-2.470038	6.06E-03	0.0795
mmu-miR-10	0.16462555	2.5656294	-4.749204	4.77E-02	0.25219
mmu-miR-50	0.16617021	1.335383	-6.364656	2.36E-01	0.56045
mmu-miR-70	0.16676795	0.9491657	-6.781216	3.84E-01	0.69435
mmu-miR-66	0.1672439	7.1098544	0.000284	6.73E-04	0.02625
mmu-miR-15	0.16779795	2.5261191	-4.802394	5.02E-02	0.25829
mmu-let-7j	0.16863238	1.1117064	-6.61643	3.14E-01	0.63146
mmu-miR-9-!	0.16882619	5.5443924	-1.324167	2.19E-03	0.0487
mmu-miR-30	0.16894232	2.7847917	-4.456229	3.64E-02	0.21444
mmu-miR-23	0.16929055	4.304857	-2.592061	6.76E-03	0.08567
mmu-miR-87	0.17288494	1.2891396	-6.418749	2.51E-01	0.57136
mmu-miR-18	0.17442681	1.3424997	-6.356247	2.34E-01	0.55766
mmu-miR-30	0.17717873	1.2259827	-6.491	2.72E-01	0.5937
mmu-miR-69	0.1776937	4.7853468	-2.074577	4.26E-03	0.06784
mmu-miR-21	0.17928534	1.4750623	-6.195878	1.97E-01	0.51845
mmu-miR-46	0.18238635	3.0626547	-4.091551	2.60E-02	0.18271
mmu-miR-7a	0.18264649	3.1183774	-4.019518	2.44E-02	0.17698
mmu-miR-37	0.18302336	4.7727743	-2.087684	4.31E-03	0.06784
mmu-miR-12	0.18441185	0.1188985	-7.264928	9.10E-01	0.96612
mmu-let-7d-!	0.18613239	7.4652134	0.26541	5.30E-04	0.02502
mmu-miR-71	0.18764267	2.9509467	-4.237111	2.97E-02	0.1906
mmu-miR-70	0.1878353	7.8989519	0.573669	4.02E-04	0.0208
mmu-miR-72	0.18818224	5.5366746	-1.331408	2.20E-03	0.0487
mmu-miR-80	0.18842946	3.3108935	-3.773806	1.95E-02	0.15234
mmu-miR-69	0.18925753	1.4644835	-6.208918	2.00E-01	0.51936
mmu-miR-19	0.18946831	2.1955722	-5.249536	7.66E-02	0.31181
mmu-miR-15	0.19025917	2.5608446	-4.755641	4.80E-02	0.25219
mmu-miR-30	0.19151431	7.275566	0.125382	6.02E-04	0.026
mmu-miR-30	0.19266407	3.4108621	-3.648244	1.74E-02	0.14412
mmu-miR-30	0.19350568	2.6484169	-4.638085	4.30E-02	0.2359
mmu-miR-19	0.19460905	7.4238965	0.235184	5.45E-04	0.02502
mmu-miR-18	0.19591963	1.255467	-6.457513	2.62E-01	0.58318
mmu-miR-70	0.19602339	4.6146831	-2.254457	5.00E-03	0.07275
mmu-miR-70	0.19631485	1.3751266	-6.31742	2.24E-01	0.54414
mmu-miR-70	0.19648504	6.0246796	-0.888189	1.49E-03	0.0413
mmu-miR-70	0.20204657	3.4917721	-3.547676	1.59E-02	0.14007

mmu-miR-35	0.20452821	3.5794852	-3.439743	1.44E-02	0.13129
mmu-miR-51	0.2055253	10.1348386	1.938259	1.15E-04	0.00955
mmu-miR-68	0.20602201	1.7192661	-5.885505	1.43E-01	0.43301
mmu-miR-28	0.20628612	2.3747918	-5.006815	6.08E-02	0.28131
mmu-miR-45	0.20774994	5.0398912	-1.814086	3.38E-03	0.06127
mmu-miR-72	0.21931813	4.3236977	-2.571118	6.63E-03	0.08465
mmu-miR-31	0.22312191	1.7517129	-5.843155	1.37E-01	0.42462
mmu-miR-66	0.22601415	4.552702	-2.320845	5.31E-03	0.07477
mmu-miR-23	0.22647059	6.0887882	-0.832105	1.41E-03	0.0413
mmu-miR-66	0.23047072	3.1442067	-3.986263	2.37E-02	0.17473
mmu-miR-19	0.23177191	2.1198808	-5.351912	8.45E-02	0.3306
mmu-miR-15	0.23218947	0.491596	-7.133472	6.43E-01	0.84191
mmu-miR-15	0.23385548	2.1423549	-5.321537	8.20E-02	0.32538
mmu-miR-15	0.23777387	2.2718033	-5.146294	6.94E-02	0.29785
mmu-miR-19	0.24582414	1.9248185	-5.61414	1.09E-01	0.37714
mmu-miR-26	0.2464225	7.4034847	0.220194	5.52E-04	0.02502
mmu-miR-29	0.24649517	2.846844	-4.374056	3.37E-02	0.20726
mmu-miR-30	0.25269905	2.2903605	-5.121155	6.77E-02	0.29347
mmu-miR-76	0.25527315	4.381312	-2.507409	6.27E-03	0.08107
mmu-miR-46	0.26040302	3.0725841	-4.078687	2.57E-02	0.18192
mmu-miR-66	0.26248987	1.4874142	-6.180603	1.94E-01	0.51559
mmu-miR-66	0.26732974	3.5406595	-3.487378	1.51E-02	0.1351
mmu-miR-51	0.26860782	1.5005521	-6.1643	1.91E-01	0.51498
mmu-miR-46	0.27090675	1.2149171	-6.503454	2.76E-01	0.59614
mmu-miR-10	0.27251835	1.4609497	-6.213266	2.01E-01	0.52089
mmu-miR-36	0.2742063	4.4453794	-2.437153	5.89E-03	0.07784
mmu-miR-36	0.28431894	4.0539405	-2.876149	8.71E-03	0.09902
mmu-let-7k	0.28543096	5.191473	-1.663279	2.96E-03	0.05923
mmu-miR-70	0.28908197	4.763392	-2.09748	4.35E-03	0.06784
mmu-miR-51	0.29114858	0.4839783	-7.137679	6.48E-01	0.84652
mmu-miR-30	0.29426981	3.385407	-3.68008	1.79E-02	0.14627
mmu-miR-32	0.29579557	2.479676	-4.865028	5.32E-02	0.2632
mmu-miR-80	0.3146693	0.8666561	-6.857842	4.24E-01	0.71995
mmu-miR-46	0.31523284	6.0812321	-0.83869	1.42E-03	0.0413
mmu-miR-70	0.31592164	1.940208	-5.593577	1.07E-01	0.37368
mmu-miR-67	0.31794995	1.8384822	-5.728941	1.22E-01	0.39861
mmu-miR-70	0.31951589	4.852424	-2.005037	4.01E-03	0.06629
mmu-miR-32	0.32438505	2.5068339	-4.828389	5.14E-02	0.26029
mmu-miR-43	0.32905567	5.5073051	-1.359033	2.26E-03	0.0487
mmu-miR-70	0.32984962	3.0395577	-4.121524	2.68E-02	0.18505
mmu-miR-33	0.33212506	5.576735	-1.293905	2.13E-03	0.04827
mmu-miR-69	0.33421865	2.8659153	-4.34888	3.29E-02	0.20548
mmu-miR-16	0.34038216	2.0571937	-5.436502	9.17E-02	0.34666
mmu-miR-13	0.34159909	2.9120724	-4.28811	3.12E-02	0.19655
mmu-miR-34	0.35453505	6.0277591	-0.885484	1.48E-03	0.0413
mmu-miR-63	0.35829848	3.0658255	-4.087442	2.59E-02	0.1827

mmu-miR-13	0.35861291	2.9440981	-4.246083	3.00E-02	0.19073
mmu-miR-70	0.35924582	1.7071158	-5.901308	1.45E-01	0.43602
mmu-miR-19	0.36029601	1.8080414	-5.769157	1.27E-01	0.40597
mmu-miR-28	0.36280825	4.0493467	-2.88144	8.75E-03	0.09902
mmu-miR-14	0.36736672	6.4221335	-0.548074	1.10E-03	0.0373
mmu-miR-14	0.38337864	2.8216846	-4.407327	3.48E-02	0.20915
mmu-miR-18	0.38357647	5.5122497	-1.354375	2.25E-03	0.0487
mmu-miR-70	0.38916313	5.5989969	-1.273153	2.09E-03	0.04827
mmu-miR-76	0.39142639	3.2611385	-3.836826	2.06E-02	0.15963
mmu-miR-30	0.39150526	2.3104577	-5.093931	6.60E-02	0.28795
mmu-miR-69	0.39739651	1.7351869	-5.864751	1.40E-01	0.42949
mmu-miR-18	0.40068861	7.8883535	0.566328	4.05E-04	0.0208
mmu-miR-72	0.40082163	2.096334	-5.383712	8.71E-02	0.33696
mmu-miR-14	0.4043055	3.8919965	-3.064639	1.03E-02	0.11135
mmu-miR-70	0.40453429	1.8688431	-5.688687	1.17E-01	0.39172
mmu-miR-50	0.40685422	1.9457565	-5.586157	1.06E-01	0.37363
mmu-miR-64	0.40873355	3.5136812	-3.520609	1.55E-02	0.13856
mmu-miR-36	0.409508	8.9034289	1.2294	2.22E-04	0.01454
mmu-miR-26	0.41073986	10.4396981	2.099742	9.93E-05	0.00945
mmu-miR-12	0.41078928	2.1812863	-5.268873	7.80E-02	0.31448
mmu-miR-69	0.42098076	5.062908	-1.790982	3.31E-03	0.06061
mmu-miR-34	0.42244211	3.1262007	-4.009436	2.42E-02	0.17665
mmu-miR-37	0.42854889	5.9157474	-0.9846	1.62E-03	0.04311
mmu-miR-20	0.43076631	2.2638256	-5.157101	7.01E-02	0.29891
mmu-miR-46	0.43166996	1.8189964	-5.754701	1.25E-01	0.40481
mmu-miR-72	0.43298941	2.3131073	-5.090342	6.58E-02	0.28795
mmu-miR-63	0.43504433	6.2640648	-0.681195	1.24E-03	0.03986
mmu-miR-69	0.43696858	1.9478732	-5.583325	1.06E-01	0.37363
mmu-miR-46	0.44173867	2.0195762	-5.48714	9.63E-02	0.35567
mmu-miR-31	0.44546033	5.3166968	-1.541061	2.65E-03	0.0543
mmu-miR-30	0.44606403	3.9741664	-2.968495	9.45E-03	0.10476
mmu-miR-18	0.44892676	3.1061234	-4.035325	2.47E-02	0.17751
mmu-miR-69	0.45194484	2.1522973	-5.308092	8.10E-02	0.3229
mmu-miR-29	0.46663136	5.7461927	-1.137515	1.86E-03	0.04448
mmu-miR-30	0.46715939	10.4981791	2.130136	9.65E-05	0.00945
mmu-miR-30	0.46919786	6.6797353	-0.336956	9.10E-04	0.03314
mmu-miR-46	0.47095262	2.724174	-4.536862	3.92E-02	0.2228
mmu-miR-67	0.47978926	5.1797665	-1.674813	2.99E-03	0.05923
mmu-miR-76	0.48075154	2.8244472	-4.403671	3.47E-02	0.20915
mmu-miR-69	0.48320799	2.8453865	-4.375982	3.38E-02	0.20726
mmu-miR-72	0.48343606	5.7204161	-1.161072	1.89E-03	0.04448
mmu-miR-18	0.48723954	3.3559908	-3.716987	1.85E-02	0.14868
mmu-miR-19	0.48786921	2.1937492	-5.252004	7.67E-02	0.31188
mmu-miR-69	0.48793412	2.7417875	-4.513398	3.83E-02	0.221
mmu-miR-29	0.49426827	4.890227	-1.96613	3.87E-03	0.06518
mmu-miR-70	0.50186627	3.6937738	-3.300839	1.27E-02	0.12417

mmu-miR-70	0.50348514	4.122125	-2.797995	8.12E-03	0.09473
mmu-miR-34	0.50393522	4.2303911	-2.675366	7.28E-03	0.08752
mmu-miR-13	0.50758382	9.1769818	1.395262	1.90E-04	0.01294
mmu-miR-70	0.51091373	4.7795191	-2.08065	4.29E-03	0.06784
mmu-miR-70	0.513648	2.3954788	-4.978821	5.92E-02	0.27989
mmu-miR-66	0.51419134	2.7725252	-4.472518	3.69E-02	0.21609
mmu-miR-70	0.52034741	4.2744309	-2.625997	6.96E-03	0.08657
mmu-miR-21	0.52460538	3.8946735	-3.061491	1.03E-02	0.11135
mmu-miR-30	0.53181362	5.4157575	-1.445864	2.44E-03	0.05044
mmu-miR-70	0.53192662	1.972326	-5.550579	1.02E-01	0.36897
mmu-miR-70	0.53238537	3.4111061	-3.647939	1.74E-02	0.14412
mmu-miR-69	0.53321703	5.0216598	-1.832439	3.44E-03	0.06127
mmu-miR-46	0.54158787	2.9717587	-4.20988	2.90E-02	0.18992
mmu-miR-30	0.54745185	15.1240713	4.062244	1.48E-05	0.00315
mmu-miR-81	0.54957469	2.707071	-4.559672	4.00E-02	0.22588
mmu-miR-30	0.54973238	3.0301751	-4.133718	2.71E-02	0.1851
mmu-miR-18	0.55924049	5.5829167	-1.288136	2.12E-03	0.04827
mmu-miR-11	0.56259211	4.6131268	-2.256117	5.01E-03	0.07275
mmu-miR-70	0.56986181	3.3091506	-3.776008	1.95E-02	0.15234
mmu-miR-76	0.57205294	4.6704617	-2.195197	4.75E-03	0.07142
mmu-miR-92	0.57208152	1.7101429	-5.897373	1.45E-01	0.43602
mmu-miR-69	0.57534198	7.0319907	-0.059403	7.10E-04	0.027
mmu-miR-64	0.57819561	3.0489839	-4.109283	2.65E-02	0.18366
mmu-miR-14	0.58005043	20.0288303	5.420492	3.42E-06	0.00163
mmu-miR-69	0.58040531	3.4640501	-3.582026	1.64E-02	0.14179
mmu-miR-72	0.58065848	2.9881331	-4.18849	2.84E-02	0.1885
mmu-miR-22	0.59144169	5.7211616	-1.160389	1.89E-03	0.04448
mmu-miR-30	0.60536741	5.8166512	-1.073545	1.75E-03	0.04371
mmu-miR-68	0.62177205	2.7507081	-4.501525	3.79E-02	0.22038
mmu-miR-68	0.62760759	10.195821	1.970975	1.12E-04	0.00955
mmu-miR-33	0.63592645	5.8616457	-1.033012	1.69E-03	0.04311
mmu-miR-30	0.64367482	5.0915893	-1.762296	3.23E-03	0.06025
mmu-miR-37	0.64593673	1.7828109	-5.802373	1.31E-01	0.41404
mmu-miR-72	0.65091247	4.5212768	-2.354723	5.47E-03	0.07649
mmu-miR-69	0.69058382	7.4839229	0.279047	5.24E-04	0.02502
mmu-miR-47	0.72278407	3.6305622	-3.377422	1.36E-02	0.12755
mmu-miR-51	0.73367697	3.7195931	-3.269733	1.24E-02	0.12267
mmu-miR-30	0.73372339	8.8086027	1.170701	2.34E-04	0.01483
mmu-miR-51	0.7354946	4.8988042	-1.957331	3.84E-03	0.06518
mmu-miR-13	0.75673584	8.1656947	0.755391	3.41E-04	0.01966
mmu-miR-69	0.75743433	3.7333187	-3.253238	1.22E-02	0.1215
mmu-miR-13	0.76586887	8.3420782	0.87244	3.07E-04	0.01823
mmu-miR-70	0.76726827	6.479628	-0.500337	1.05E-03	0.03639
mmu-miR-19	0.77312626	1.3405037	-6.358608	2.35E-01	0.55815
mmu-miR-51	0.77600551	6.6589087	-0.353762	9.23E-04	0.03314
mmu-miR-32	0.77774712	5.8644729	-1.030473	1.69E-03	0.04311

mmu-miR-30	0.78133035	11.7250249	2.727841	5.49E-05	0.00774
mmu-miR-69	0.78208278	1.2892172	-6.418659	2.51E-01	0.57136
mmu-miR-46	0.79345881	2.7473591	-4.505981	3.81E-02	0.22038
mmu-miR-70	0.82238849	4.6570545	-2.209399	4.81E-03	0.07142
mmu-miR-10	0.82817649	12.162157	2.923886	4.55E-05	0.00722
mmu-miR-30	0.82865528	2.4175962	-4.948904	5.76E-02	0.27503
mmu-miR-34	0.84492645	4.7853237	-2.074601	4.26E-03	0.06784
mmu-miR-51	0.85313017	3.1112408	-4.028721	2.46E-02	0.17712
mmu-miR-10	0.87014545	7.9125282	0.583058	3.99E-04	0.0208
mmu-miR-1a	0.89826118	13.7187336	3.55989	2.45E-05	0.00466
mmu-miR-57	0.9022521	3.0730027	-4.078145	2.57E-02	0.18192
mmu-miR-72	0.91250054	6.1076004	-0.815738	1.39E-03	0.0413
mmu-miR-69	0.94423066	7.5016188	0.291917	5.18E-04	0.02502
mmu-miR-34	0.96275373	3.2577915	-3.841077	2.07E-02	0.15963
mmu-miR-66	1.01027335	3.7514539	-3.231488	1.20E-02	0.12041
mmu-miR-11	1.03750651	2.9658045	-4.217665	2.92E-02	0.19029
mmu-miR-9-[illegible]	1.05847949	11.6434176	2.690311	5.69E-05	0.00774
mmu-miR-72	1.08985772	7.1366476	0.020688	6.61E-04	0.02625
mmu-miR-70	1.1118767	4.2368778	-2.668076	7.23E-03	0.08752
mmu-miR-30	1.20669469	2.952913	-4.234536	2.97E-02	0.1906
