# Supplementary material for: A Plasma Extracellular Vesicle-Derived microRNA Signature as a Potential Biomarker for Subclinical Coronary Atherosclerosis
Source: Int J Mol Sci. 2025 Sep 7;26(17):8727. doi: 10.3390/ijms26178727 (PMC12428963; doi:10.3390/ijms26178727)
Supplement: Supplementary file 1 [file ijms-26-08727-s001.zip › Table_S3.pdf]

Supplementary Table S3. Target genes by miRNA present in at least 3 databases

hsa-miR-487b-3p	hsa-miR-379-5p	hsa-miR-146b-5p	hsa-miR-4701-3p	hsa-miR-6849-5p	hsa-miR-1180-3p
FAM83B	AAK1	AAK1	MFAP5	CSDE1	PRIMA1
ALDH1A3	ABCC2	ABL2	CRNKL1	CDH6	COG5
AGA	ACKR4	BCORL1	PRPF38B	AXIN2	RPS19BP1
CRYBA1	ACOX3	BTG2	ETFRF1	KIAA1211L	TRAPPC3
ANP32E	ADAMTS15	CALHM5	ZNHIT1	CCDC68	TEF
ZNF280D	ADPRH	CCDC6	HSD17B4	CCR1	IGDCC3
GLCCI1	ANKRD40	CDKN2AIP	GEMIN5	NDP	SLC26A4
NRARP	ARL4A	CDS1	FGFR1	TIMM23B	FXYD5
NSMF	ARRB1	DCAF12	MPDU1	ZNF124	MKNK2
FAM241A	ATP1A2	DDHD1	NRG3	LRRN4CL	GGACT
PAK5	B4GALT3	ERLEC1	HDGF	CTXN2	NRN1
SSBP3	BCAS3	FAM210A	CD37	TTC29	UTP15
DIO2	BRCA1	FBXO28	OSBPL1A	PLPP3	OTUD7B
CXADR	C17orf58	FBXW2	OR2W5	HPSE	ST7
DLAT	C5orf51	GATD3	LIPA	FMO5	LRRC27
ZIC1	C7	GOSR1	ZCCHC17	ECT2	ETS1
CCNO	C8orf34	GRID1	SALL3	NOMO3	COL12A1
USP25	CCDC88A	IGSF1	RAB27B	NOMO2	AKR1C2
ZMYND8	CCPG1	IRAK1	SLC35E2A	KCNJ13	MLLT1
ZNF879	CD226	KDM2B	DUOX1	MLLT10	PNISR
GRM3	CD59	KIF24	NTS	NOMO1	PPP1R17
SRP9	CDKN2AIP	KLF7	PQLC2L	LAMA3	KCNA6
IDS	CLDN1	LCOR	OCM2	METTL4	CERS1
SRY	CREBBP	LFNG	EPM2A	CDH12	HECW1
DNAJC10	CTF1	LRP2	EXOC3L2	YPEL4	CMTM7
TMEM53	CTSL	LRRC15	RPE	ACVR1C	ZNF341
SPATA6	CXCL11	MARCHF6	IQSEC2	TMF1	ZSWIM6
DIABLO	CYB561D1	MMP16	DOCK7	KDM5A	MRPS11
IRS1	CYP2U1	MYBL1	TMEM100	EFEMP1	SAG
ANKRD45	DCUN1D4	MYT1	OCM	NKD1	KANSL1
PAK7	DNAJC30	NEMP1	BEST1	NEMP2	TMEM88
HERPUD1	DNAL1	NOVA1	USP14	TMPRSS11D	PINK1
PCF11	DNM3	NSD1	CAMK2D	EFNA5	
KAT6B	DNMT3B	PHOX2B	ATP2B2	NRG4	
CSNK2B	DYNC1LI2	PIP5K1B	GLYATL2	EIF2S1	
WWC2	EDEM3	PPP1R11	TYRO3	ETV1	
EFHD2	EDN1	PRCP	KSR2	PCSK5	
PLXNB2	EHD4	PRKAA2	PHLDB2	ABCA5	
ITGA6	EIF4G2	PRX	TNPO3	VEGFC	
LRP6	ELMOD2	PTGFRN	SPTBN1	PLXDC2	
	ELOA	RFX7	C6orf89	FUT10	
	ELP4	RIMS2	DHFR	KIN	
	ENAM	ROBO1	ADH6	FCHO2	
	ESRP1	RUNX1T1	ZNF607	GOLGA7	
	FAHD2B	SAMD8	TBX5	CCNJ	
	FAM114A2	SCN3B	CMTR1	FSBP	
	FAM222B	SEC23IP	USP1	MED13	
	FAM76A	SGIP1	FLI1	TTC30A	
	FBXO32	SIAH2	TCEANC2	TMEM45B	
	FIGN	SLC2A3	YIPF1	TMEM69	
	FOXP2	SORT1	DRAM1	MACF1	
	FZD4	SRSF12	FAM222A	USP3	
	GABRA3	STRBP	SUOX	GPR85	
	GALNT15	TDRKH	MSRB2	LHX6	
	GAREM2	TRAF6	TBC1D23	TIAM2	
	GAS7	USP3	PHF7	NAMPT	
	GDF6	USP47	CACUL1	RHOBTB1	
	GHRHR	WWC2	DPH6	KDM5B	
	GPATCH2L	XKR4	MBNL2	ABL2	
	GSDMD	ZDHHC13	ARHGAP20	SLC8A2	
	HACD4	ZFYVE1	RNF212	C1orf52	
	HBEGF	ZNF367	NCR3	UHRF2	
	HECW2	ZNF512B	DOK5	EPN2	
	HGF	ZNF532	ABCA6	ZNF780B	
	HLCS	ZNF652	CDY1B	JMJD6	
	HSPA5	ZNRF3	SYK	PCLO	
	HTR1D		GRM7	CELF2	
	HTR2C		CDY1	EBF3	
	ICOS		ZDHHC20	ANGPT2	
	IFNAR2		PLEKHG2	RNF219	
	IGF1R		HAX1	EXOSC3	
	IL18RAP		COBL	C1S	
	INSR		LURAP1	BCL6	
	KATNAL1		FAN1	SLC31A1	
	KCNC1		PPIL6	TSHZ1	
	KCNJ1		NRK	LUZP1	
	KCNQ3		SLC6A11	IGFBP7	
	KCTD6		SDHC	TAGAP	
	KLHL14		SRC	FAM222B	
	KNDC1		PDK3	SOX8	
	KNG1		PTRH2	ZC3H12B	
	LGI4		VWC2	TRIM26	
	LIN28B		GSTA2	VXN	
	LINGO2		ADAT1	ANGEL2	
	LPGAT1		HMGN5	ACAP2	

LRRC8C	LEKR1	CHD2
LRRC8D	MRPL43	ADAMTS17
LTBP1	ABCC6	LPP
LZTS3	TMEM97	CHST9
MAP3K2	AKR7A2	UBE4A
MBNL3	PCCA	WDR83OS
MCTP2	FAM122C	FAM120AOS
METTL4	POLR1C	RAD54B
MGST1	PLCB4	DHX33
MKX	TBCK	RAD51B
MON1B	EAPP	ENY2
MTM1	SMURF1	SYP
MTMR2	HMBOX1	FAT3
MYO10	JMJD7-PLA2G4B	BCAT1
NECTIN2	PLA2G4B	ABCA13
NFAT5	SMG5	NUFIP2
NFATC3	MN1	NAGK
NHLRC2	ARID1B	MIA2
NHLRC3	APH1B	PCBP2
NME9	MEF2D	KCNMA1
NR6A1	PDE7B	SIGMAR1
OGN	DNAJC14	NFS1
OLFM3	AZIN1	SELL
PCDH17	RAD51B	EPM2AIP1
PCGF3	IBA57	KCTD12
PCGF5	ITCH	MTCP1
PDE2A	ZSCAN18	TRIM67
PDK1	OTC	KCNJ15
PEG10	AFF2	IDI2
PEX11B	FSD2	ELOVL4
PGM3	RBBP9	DCAF12L1
PHLDA2	MALT1	TPP2
PITPNM3	TRIM5	INSM2
PLEKHG2	ZNF135	RSF1
PLPPR4	PRR23B	IL11
PPP1R15B	SKI	LANCL2
PPP4R1	MED14	IYD
PRKN	HDAC4	AMOT
PSMB5	ADD3	IL6ST
PTK2	USP13	COPA
PTPN14	FAM47E	LOC100130357
PXT1	FAM220A	KRTAP4-9
RAB30	ADAMDEC1	C16orf70
RAB3IP	ARMC8	ANGPT1
RAD18	IFT22	DLC1
RBP1	PANK3	RFC3
RIOK2	CLIP3	HTR2C
RNF32	ADGRL1	AKAP1
RNF6	ZNF544	DCP1A
ROR1	WDR20	EFR3A
RTN4RL2	MAL	SHOC1
SEC22B	ARHGEF11	C20orf194
SEMA3A	KRT31	GGNBP2
SGPP1	IFI44L	KRTAP4-11
SH3GLB1	RCSD1	ZNF451
SLC19A2	DHX9	MAP1A
SLC20A1	KLF12	VCAN
SLC25A53	MATN2	KRTAP4-8
SLC5A6	SEC14L4	EML6
SLC6A16	COPA	ARGFX
SLC6A4	ST6GALNAC5	FEM1A
SMAD9	C1orf21	TOP2A
SMAP1	TMED10	EDA
STAG1	ITPR1	A1CF
STAM2	HLF	E2F7
STAMBP	TCEAL5	RAVER2
STON2	DPYSL2	GLYATL3
STRN	MAT2A	ZNF74
SVOPL	PLEKHM1	LEPROTL1
TBCEL	ZNF548	PPP3R1
TBCK	PRPF4B	CTAGE1
TBP	CBX3	CTTN
TCF7L2	CNNM3	ATP8A2
TENM1	NDUFC2	RBSN
TFPI2	EPM2AIP1	ADRB3
TGFBR1	MYO1B	CAST
TLCD2	AGMAT	FAXC
TMEM170B	ALPK3	CFL2
TMEM35B	ARHGAP26	SH3KBP1
TMF1	RBM4	SAP130
TMLHE	GNS	BMPR2
TNRC6B	FYB2	C1QL3
TOMM20	C3orf33	MOB1B
TRIM45	SELENBP1	RAB10
TSC22D2	PMPCB	REG1A
TTBK2	NAA15	NOL4
TTC31	ADAMTS5	MFSD9
TXNL1	CEP63	COL4A1
UBE2E3	TSPAN33	WWP2

UBL3
UBN2
UNC13B
UNC13C
UTP25
VGLL3
WNT2B
WNT9B
YARS
YBX1
ZBTB26
ZFAND5
ZMAT2
ZNF211
ZNF652

ORMDL2
MEGF11
KCNK17
DNAH2
NUDT7
ENTPD1
SLC12A8
IRGQ
HIST2H2BF
ZNF275
MASP2
FAM13C
CDKL4
ARHGEF12
C1orf116
PDE6B
LOXHD1
XK
WASHC2A
NOTCH4
BSDC1
LURAP1L
SLC25A17
ZNF587B
DDR2
RGS7BP
FSD1L
TBC1D16
ABAT
TRDMT1
HOXB9
ZNF264
CCT6A
PSME3
GNAS
BDH2
ILDR2
FAM104A
KLHL18
POF1B
ROPN1
RNASE13
TSR1
CHMP3
UNC45B
TET2
ENKUR
SKIDA1
SGCD
C1orf74
IQSEC3
MRPL42
RNF103-CHMP3
LRP6
FUS
AP5B1
RAPH1
FLVCR1
PLBD2
KAZN
TMEM151A
MYB
CLCN5
RNF157
AQP1
ZNF678
STK38L
TSC22D1
HDAC6
C18orf54
CGNL1
ERBB4
ANOS1
LSM12
SH3TC2
SLC9A7
MTDH
SOGA1
INO80D
KRT74
DENND1B
F13A1
SLC22A23
UBE2QL1
RNF165
VWA3B
OAS2
GBA2
SSR1

DNAJC18
SLC25A13
ARL2BP
JOSD1
FOXL2NB
TAB3
HEG1
PXDN
FGF2
TP73
UBE2QL1
CUTC
TUBB1
NUAK2
MMD
ERBB3
PPP2R2A
DESI2
TAF5

TNP2
EPHB2
MAVS
SELENOT
ST7L
SMCP
PLD1
NEUROD2
GABBR2
BTNL3
LYPD6
PTCH1
FAS
PPP3R1
ABCG4
BAIAP3
CLCN6
ZBTB20
FBXL2
PRKCA
KRTAP9-3
FAXDC2
SCN8A
NR2C1
MAPRE3
PTPRB
ARV1
PTBP3
NRXN3
KRTAP9-4
TMEM178B
KRTAP9-2
WDR38
KDM4C
DZIP1
TJP1
ZKSCAN4
NOVA2
EIF2S1
ATP8A2
KRTAP9-8
OPCML
ZNF615
ASIC2
MRGPRX3
ZNF142
CCR2
CREB5
IPO9
SYT16
FAM171A1
PLCL1
ZNF460
LCOR
TENM4
CBLB
ZNF587
WAS
OBSL1
EDNRB
LSMEM2
FNDC5
ANTXR1
ATF7IP
SLC11A2
ATP5MC3
ERICH2
CASR
CDRT1
MYO3B
FAM126A
MACROD2
PLIN3
NAT2
FGF12
RAB6B
USF3
HSPB6
KLK10
PMM2
DACT2
TMEM185A
ST8SIA5
PARP11
RO60
SELENOH
ALDH4A1
FLT1
RPS24

TMEM130
TAOK2
NYNRIN
NTRK2
SLC24A2
FN1
DISC1
PPEF2
