# Supplementary material for: A Plasma Extracellular Vesicle-Derived microRNA Signature as a Potential Biomarker for Subclinical Coronary Atherosclerosis
Source: Int J Mol Sci. 2025 Sep 7;26(17):8727. doi: 10.3390/ijms26178727 (PMC12428963; doi:10.3390/ijms26178727)
Supplement: Supplementary file 1 [file ijms-26-08727-s001.zip › Table_S4.pdf]

Supplementary Table S4. Differentially expressed Genes.

Illumina HumanHT-12 versión 3 Expression BeadChip

SYMBOL	ACCESSION	FC	REGULATION	P	FDR
SPP1	NM_001040058.1	121.88	Up	1.82E-13	5.12E-10
SPP1	NM_000582.2	109.05	Up	1.82E-13	5.12E-10
MMP12	NM_002426.2	88.93	Up	1.82E-13	5.12E-10
MMP9	NM_004994.2	76.12	Up	1.82E-13	5.12E-10
CIDEC	NM_022094.2	61.20	Down	2.91E-11	8.97E-10
KIAA1881	XM_001130790.1	60.11	Down	9.97E-12	3.96E-10
LOC642113	XM_936253.1	53.25	Up	5.44E-12	2.62E-10
MMP7	NM_002423.3	52.52	Up	2.21E-13	1.70E-10
LOC647450	XM_936518.1	52.37	Up	5.12E-12	2.54E-10
LOC652493	XM_941953.1	52.08	Up	3.55E-12	2.05E-10
LOC652694	XM_942302.1	47.20	Up	3.14E-12	1.90E-10
MMP12	NM_002426.2	47.05	Up	7.00E-13	9.64E-11
APOD	NM_001647.2	39.70	Down	1.24E-10	2.83E-09
APOC1	NM_001645.3	36.21	Up	2.21E-13	1.70E-10
PLA2G7	NM_005084.2	29.88	Up	2.21E-13	1.70E-10
MMP7	NM_002423.3	28.34	Up	2.21E-13	1.70E-10
KIAA1199	NM_018689.1	25.49	Up	1.82E-13	5.12E-10
CIDEA	NM_001279.2	24.53	Down	6.22E-11	1.65E-09
ADAMDEC1	NM_014479.2	24.16	Up	1.82E-13	5.12E-10
SCARA5	NM_173833.4	22.81	Down	6.16E-13	9.46E-11
ACP5	NM_001611.2	22.57	Up	4.78E-13	9.50E-11
IGLL1	NM_020070.2	19.96	Up	7.83E-12	3.36E-10
LOC652102	XM_941434.1	19.02	Up	5.23E-11	1.43E-09
APOE	NM_000041.2	17.63	Up	7.45E-13	9.75E-11
PLIN	NM_002666.3	17.34	Down	5.87E-11	1.57E-09
ACTA1	NM_001100.3	16.34	Down	7.40E-11	1.89E-09
RGS1	NM_002922.3	15.79	Up	6.57E-13	9.51E-11
OLR1	NM_002543.3	15.77	Up	3.70E-13	9.98E-11
MYOC	NM_000261.1	15.24	Down	3.05E-13	1.12E-10
CXCL14	NM_004887.3	15.09	Down	1.95E-07	1.80E-06
IFI30	NM_006332.3	15.05	Up	5.78E-13	9.52E-11
LOC285016	NM_001002919.1	14.89	Down	1.69E-12	1.28E-10
CNTFR	NM_001842.3	14.32	Down	9.01E-13	9.71E-11
THRSP	NM_003251.2	14.22	Down	2.91E-11	8.97E-10
LOC399888	XM_941808.2	13.99	Down	3.05E-13	1.12E-10
TM4SF19	NM_138461.2	13.93	Up	1.31E-12	1.12E-10
C15orf48	NM_032413.2	13.77	Up	1.82E-13	5.12E-10
IL8	NM_000584.2	13.41	Up	9.22E-10	1.56E-08
RBPMS2	NM_194272.1	13.40	Down	3.05E-13	1.12E-10
HMOX1	NM_002133.1	13.14	Up	4.20E-13	9.91E-11
PI16	NM_153370.2	12.79	Down	1.28E-09	2.06E-08
FBLN1	NM_001996.2	12.74	Down	1.40E-12	1.15E-10
PTCHD1	NM_173495.2	12.28	Down	4.20E-13	9.91E-11
SLC16A10	NM_018593.3	11.68	Up	7.45E-13	9.75E-11
SLC2A5	NM_003039.1	11.67	Up	9.01E-13	9.71E-11
IL8	NM_000584.2	11.40	Up	3.00E-09	4.34E-08
SLC16A3	NM_004207.2	11.36	Up	1.82E-13	5.12E-10
ADORA3	NM_001081976.1	10.94	Up	5.43E-13	9.56E-11
IGJ	NM_144646.2	10.79	Up	2.90E-10	5.74E-09
LOC651751	XM_940969.1	10.51	Up	1.27E-11	4.71E-10
AQP7P2	NR_002823.1	10.32	Down	2.44E-11	7.79E-10

SLAMF8	NM_020125.2	10.23	Up	2.07E-13	2.02E-10
C6	NM_000065.1	10.21	Down	3.47E-11	1.03E-09
CTSZ	NM_001336.2	9.90	Up	2.36E-13	1.49E-10
BTC	NM_001729.1	9.77	Down	1.94E-13	2.47E-10
ITGAX	NM_000887.3	9.51	Up	2.36E-13	1.49E-10
ABCA8	NM_007168.2	9.41	Down	1.80E-12	1.33E-10
THBS1	NM_003246.2	9.34	Up	2.51E-13	1.34E-10
PNKD	NM_022572.2	9.24	Up	4.48E-13	9.68E-11
FBLN1	NM_006487.2	9.20	Down	1.65E-10	3.57E-09
MUSTN1	NM_205853.2	9.17	Down	6.53E-12	2.97E-10
RGS7BP	NM_001029875.1	9.14	Down	1.82E-13	5.12E-10
MYOC	NM_000261.1	9.11	Down	3.25E-13	1.06E-10
CILP	NM_003613.2	9.05	Down	1.58E-09	2.48E-08
PLAUR	NM_001005376.1	9.04	Up	1.09E-12	1.03E-10
CXCR4	NM_003467.2	8.86	Up	4.65E-11	1.31E-09
CILP	NM_003613.2	8.85	Down	4.52E-10	8.40E-09
TSHZ2	NM_173485.4	8.84	Down	1.19E-11	4.51E-10
NPL	NM_030769.1	8.66	Up	3.05E-13	1.12E-10
NPL	NM_030769.1	8.64	Up	3.25E-13	1.06E-10
MSR1	NM_138715.1	8.49	Up	1.23E-12	1.10E-10
IL1RN	NM_173843.1	8.40	Up	2.03E-12	1.43E-10
HLA-DQA1	XM_936128.2	8.35	Up	8.46E-13	9.64E-11
MAPK13	NM_002754.3	8.29	Up	2.07E-13	2.02E-10
SEMA3E	NM_012431.1	8.22	Down	6.57E-13	9.51E-11
AQP7P2	XR_001073.1	8.17	Down	1.35E-11	4.91E-10
ATP1A2	NM_000702.2	8.16	Down	2.36E-13	1.49E-10
PLA2G2A	NM_000300.2	8.09	Down	7.33E-07	5.97E-06
THBS4	NM_003248.3	8.03	Down	3.47E-08	3.78E-07
LILRB4	NM_001081438.1	7.95	Up	4.20E-13	9.91E-11
TNMD	NM_022144.1	7.92	Down	1.31E-12	1.12E-10
HS3ST2	NM_006043.1	7.90	Up	2.30E-12	1.55E-10
LAPTM5	NM_006762.1	7.88	Up	2.36E-13	1.49E-10
CXCR4	NM_001008540.1	7.88	Up	9.01E-13	9.71E-11
ADAMDEC1	NM_014479.2	7.86	Up	2.21E-13	1.70E-10
PPP1R1A	NM_006741.2	7.82	Down	5.78E-13	9.52E-11
KYNU	NM_003937.2	7.79	Up	2.07E-13	2.02E-10
ANKRD33	NM_182608.2	7.76	Up	1.82E-13	5.12E-10
CALD1	NM_033138.2	7.76	Down	1.94E-13	2.47E-10
MATK	NM_139354.2	7.75	Up	4.20E-13	9.91E-11
CA2	NM_000067.1	7.70	Up	3.77E-12	2.12E-10
SCG2	NM_003469.3	7.64	Up	1.82E-13	5.12E-10
SDS	NM_006843.2	7.52	Up	2.07E-13	2.02E-10
SGK	NM_005627.2	7.47	Up	7.45E-13	9.75E-11
LOC652775	XM_942419.1	7.45	Up	4.81E-09	6.60E-08
SLC11A1	NM_000578.3	7.44	Up	9.01E-13	9.71E-11
DES	NM_001927.3	7.34	Down	4.78E-10	8.81E-09
MRAP	NM_178817.3	7.28	Down	1.47E-10	3.24E-09
HOXA5	NM_019102.2	7.20	Down	2.07E-13	2.02E-10
LOC649362	XM_943669.1	7.18	Up	1.31E-12	1.12E-10
FPR3	NM_002030.3	7.16	Up	2.21E-13	1.70E-10
CCL4L2	NM_207007.2	7.14	Up	4.93E-11	1.37E-09
AOX1	NM_001159.3	7.13	Down	1.23E-12	1.10E-10
ECGF1	NM_001953.2	7.03	Up	2.07E-13	2.02E-10
TMEM200A	NM_052913.2	7.01	Up	1.82E-13	5.12E-10
HAVCR2	NM_032782.3	6.96	Up	3.47E-13	1.02E-10

SRL	NM_001098814.1	6.79	Down	4.01E-12	2.20E-10
CXCL16	NM_022059.1	6.79	Up	2.21E-13	1.70E-10
TM4SF19	XM_001134247.1	6.73	Up	1.58E-12	1.23E-10
GYG2	NM_003918.2	6.71	Down	5.33E-10	9.72E-09
CD52	NM_001803.2	6.70	Up	2.45E-12	1.61E-10
CD86	NM_006889.3	6.56	Up	2.07E-13	2.02E-10
FCGR1B	NM_001017986.1	6.50	Up	4.26E-12	2.29E-10
PLCB4	NM_000933.2	6.43	Down	1.82E-13	5.12E-10
LOC646723	XR_017241.1	6.41	Up	1.91E-12	1.37E-10
SFRP1	NM_003012.3	6.40	Down	1.05E-10	2.48E-09
HLA-DRB4	NM_021983.4	6.39	Up	3.91E-11	1.13E-09
LOC731486	XM_001133149.1	6.37	Up	4.48E-13	9.68E-11
EMR2	NM_152916.1	6.37	Up	7.45E-13	9.75E-11
CALB2	NM_007088.2	6.37	Down	7.57E-08	7.63E-07
HAMP	NM_021175.2	6.36	Up	9.60E-13	9.86E-11
CD48	NM_001778.2	6.35	Up	4.82E-12	2.46E-10
ANGPTL5	NM_178127.2	6.35	Down	1.16E-12	1.07E-10
POSTN	NM_006475.1	6.35	Up	1.82E-13	5.12E-10
ABCA6	NM_080284.2	6.16	Down	2.36E-13	1.49E-10
LYZ	NM_000239.1	6.16	Up	1.65E-10	3.57E-09
TDO2	NM_005651.1	6.13	Up	2.21E-13	1.70E-10
FBP1	NM_000507.2	6.12	Up	1.02E-12	1.00E-10
FYB	NM_001465.3	6.09	Up	5.09E-13	9.46E-11
VCAM1	NM_001078.2	6.08	Up	1.82E-13	5.12E-10
SNX10	NM_013322.2	6.07	Up	6.57E-13	9.51E-11
LOC387934	XM_937508.2	6.05	Up	3.25E-13	1.06E-10
GPR81	NM_032554.2	6.02	Down	1.82E-13	5.12E-10
CD68	NM_001251.1	6.01	Up	2.07E-13	2.02E-10
IL7R	XM_937367.1	6.00	Up	7.37E-12	3.23E-10
CAPZA1	NM_006135.1	5.93	Up	1.58E-12	1.23E-10
LAMP3	NM_014398.2	5.92	Up	2.16E-12	1.48E-10
CD68	NM_001251.2	5.91	Up	3.28E-11	9.89E-10
HLA-DRB3	NM_022555.3	5.91	Up	6.16E-13	9.46E-11
MMP11	NM_005940.3	5.90	Up	2.51E-13	1.34E-10
PLAUR	NM_002659.2	5.89	Up	1.49E-12	1.19E-10
TTYH3	NM_025250.2	5.88	Up	4.78E-13	9.50E-11
FERMT3	NM_031471.4	5.87	Up	3.94E-13	1.00E-10
RTN1	NM_206852.1	5.86	Down	1.56E-08	1.86E-07
CYP4B1	NM_000779.2	5.86	Down	3.05E-13	1.12E-10
REEP1	NM_022912.1	5.81	Down	1.02E-12	1.00E-10
CDH2	NM_001792.2	5.76	Up	4.78E-10	8.81E-09
IGLL3	NM_001013618.1	5.73	Up	9.39E-12	3.80E-10
CD74	NM_001025159.1	5.71	Up	4.82E-12	2.46E-10
FZD3	NM_017412.2	5.70	Down	1.09E-12	1.03E-10
POSTN	NM_006475.1	5.69	Up	7.45E-13	9.75E-11
CXCL10	NM_001565.2	5.68	Up	7.83E-12	3.36E-10
SORBS1	NM_001034954.1	5.67	Down	4.20E-13	9.91E-11
C1QC	NM_172369.2	5.66	Up	1.02E-12	1.00E-10
PRICKLE1	NM_153026.1	5.65	Down	8.84E-12	3.64E-10
CYP2S1	NM_030622.6	5.64	Up	3.25E-13	1.06E-10
SELE	NM_000450.1	5.62	Up	1.28E-07	1.23E-06
HIF1A	NM_181054.1	5.60	Up	4.20E-13	9.91E-11
RYR3	NM_001036.2	5.59	Down	1.23E-12	1.10E-10
HLA-DRB6	NR_001298.1	5.59	Up	1.56E-08	1.86E-07
ADAM8	NM_001109.3	5.58	Up	3.33E-12	1.98E-10

PRDM1	NM_001198.2	5.58	Up	3.70E-13	9.98E-11
ITGB2	NM_000211.1	5.57	Up	4.48E-13	9.68E-11
LY86	NM_004271.3	5.56	Up	2.86E-13	1.18E-10
FNDC1	NM_032532.1	5.55	Up	1.09E-12	1.03E-10
C5orf46	NM_206966.2	5.52	Up	1.93E-11	6.49E-10
CD300A	NM_007261.2	5.51	Up	1.80E-12	1.33E-10
TYROBP	NM_003332.2	5.50	Up	3.70E-13	9.98E-11
SLC14A1	NM_015865.2	5.49	Up	1.82E-08	2.14E-07
SLC7A10	NM_019849.1	5.48	Down	8.73E-10	1.49E-08
C5AR1	NM_001736.3	5.48	Up	5.43E-13	9.56E-11
CCL3	NM_002983.1	5.47	Up	1.17E-10	2.71E-09
SERPINA1	NM_001002236.1	5.46	Up	1.40E-12	1.15E-10
CTSD	NM_001909.3	5.46	Up	2.16E-12	1.48E-10
OSM	NM_020530.3	5.45	Up	2.31E-10	4.74E-09
IRF8	NM_002163.2	5.45	Up	8.46E-13	9.64E-11
IL1B	NM_000576.2	5.45	Up	1.85E-10	3.92E-09
CCL5	NM_002985.2	5.44	Up	2.45E-12	1.61E-10
CECR1	NM_177405.1	5.43	Up	6.16E-13	9.46E-11
HLA-DMB	NM_002118.3	5.43	Up	3.47E-13	1.02E-10
ADH1C	NM_000669.3	5.42	Down	1.93E-11	6.49E-10
	AA302842	5.40	Up	6.15E-12	2.85E-10
CCL3L1	NM_021006.4	5.37	Up	5.05E-10	9.26E-09
CKB	NM_001823.3	5.37	Down	4.93E-11	1.37E-09
	BX648964	5.35	Down	3.33E-09	4.77E-08
CCL4L1	NM_001001435.2	5.33	Up	3.70E-09	5.23E-08
IGSF21	NM_032880.2	5.31	Up	5.78E-13	9.52E-11
PLD5	NM_152666.1	5.31	Down	1.05E-10	2.48E-09
LOC401845	XM_377426.1	5.29	Up	3.15E-08	3.47E-07
OSCAR	NM_130771.3	5.29	Up	4.48E-13	9.68E-11
CD163	NM_004244.4	5.26	Up	1.40E-12	1.15E-10
CA12	NM_001218.3	5.25	Up	6.16E-13	9.46E-11
CCR1	NM_001295.2	5.25	Up	1.02E-12	1.00E-10
GZMB	NM_004131.3	5.25	Up	6.99E-11	1.81E-09
PHLDA1	NM_007350.3	5.23	Up	3.09E-11	9.42E-10
SLCO2B1	NM_007256.2	5.21	Up	4.78E-13	9.50E-11
GK	NM_203391.1	5.20	Up	3.25E-13	1.06E-10
LEP	NM_000230.1	5.20	Down	7.02E-10	1.23E-08
PDPN	NM_001006625.1	5.20	Up	1.02E-12	1.00E-10
FCGR1B	NM_001004340.1	5.19	Up	7.94E-13	9.69E-11
AGTR1	NM_004835.3	5.19	Down	5.44E-12	2.62E-10
PARVG	NM_022141.4	5.16	Up	5.43E-13	9.56E-11
	X00437	5.15	Up	9.39E-12	3.80E-10
MGST1	NM_020300.3	5.15	Down	7.37E-12	3.23E-10
OSCAR	NM_133169.2	5.15	Up	1.91E-12	1.37E-10
MAP1B	NM_005909.3	5.14	Down	8.73E-10	1.49E-08
	BX095522	5.14	Down	1.01E-07	9.92E-07
CD83	NM_001040280.1	5.13	Up	8.84E-12	3.64E-10
CCL2	NM_002982.3	5.13	Up	1.24E-10	2.83E-09
IL1RN	NM_173842.1	5.13	Up	5.33E-10	9.72E-09
	AK093878	5.12	Down	4.04E-10	7.63E-09
RNASE6	NM_005615.4	5.08	Up	5.43E-13	9.56E-11
CD163	NM_203416.1	5.06	Up	1.91E-12	1.37E-10
FCGR2A	NM_021642.2	5.05	Up	4.48E-13	9.68E-11
SRGN	NM_002727.2	5.05	Up	5.09E-13	9.46E-11
C9orf58	NM_001002260.1	5.05	Down	6.94E-12	3.10E-10

SERPINA1	NM_001002235.1	5.04	Up	1.80E-12	1.33E-10
ITGB2	NM_000211.2	5.04	Up	6.57E-13	9.51E-11
AGTR1	NM_000685.4	5.03	Down	6.15E-12	2.85E-10
PITX1	NM_002653.3	5.03	Down	1.86E-07	1.72E-06
PLIN2	NM_001122.2	5.02	Up	4.01E-12	2.20E-10
SLC19A3	NM_025243.3	5.02	Down	1.50E-09	2.37E-08
TIMD4	NM_138379.1	5.02	Up	5.07E-09	6.91E-08
PLAUR	NM_001005376.1	5.01	Up	4.20E-13	9.91E-11
SLAMF6	NM_052931.3	5.01	Up	1.80E-12	1.33E-10
	AL109696	5.01	Down	8.46E-13	9.64E-11
LCP1	NM_002298.2	5.01	Up	3.94E-13	1.00E-10
PLEK	NM_002664.1	5.01	Up	4.78E-13	9.50E-11
CTHRC1	NM_138455.2	4.99	Up	7.94E-13	9.69E-11
PLAU	NM_002658.2	4.98	Up	1.82E-13	5.12E-10
TLR1	NM_003263.3	4.97	Up	2.86E-13	1.18E-10
CD14	NM_000591.2	4.96	Up	6.16E-13	9.46E-11
MSR1	NM_138716.2	4.96	Up	8.84E-12	3.64E-10
VCAM1	NM_001078.2	4.96	Up	2.36E-13	1.49E-10
ATP1B1	NM_001677.3	4.96	Up	7.45E-13	9.75E-11
SLC7A5	NM_003486.5	4.95	Up	8.73E-10	1.49E-08
MYH11	NM_001040114.1	4.95	Down	7.00E-13	9.64E-11
TSPAN8	NM_004616.2	4.94	Down	8.84E-12	3.64E-10
NDUFA4L2	NM_020142.3	4.93	Up	3.14E-12	1.90E-10
MAPK4	NM_002747.3	4.92	Down	1.49E-12	1.19E-10
CD68	NM_001251.2	4.91	Up	5.43E-13	9.56E-11
SORBS1	NM_015385.2	4.89	Down	5.87E-11	1.57E-09
VIT	NM_053276.2	4.88	Down	6.16E-13	9.46E-11
GZMK	NM_002104.2	4.85	Up	8.32E-12	3.49E-10
KRT17	NM_000422.1	4.85	Up	2.74E-10	5.48E-09
MYOM1	NM_003803.3	4.84	Down	2.68E-13	1.25E-10
SLCO2B1	NM_007256.2	4.84	Up	7.94E-13	9.69E-11
ACADL	NM_001608.2	4.83	Down	6.57E-13	9.51E-11
LUM	NM_002345.3	4.83	Up	9.01E-13	9.71E-11
SLA	NM_006748.1	4.83	Up	1.23E-12	1.10E-10
LOC642678	XM_926130.1	4.82	Down	4.82E-12	2.46E-10
GGTA1	NR_003191.1	4.81	Down	1.35E-11	4.91E-10
PEG3	NM_006210.1	4.81	Down	3.25E-13	1.06E-10
SAMSN1	NM_022136.3	4.77	Up	4.78E-13	9.50E-11
FTHL3	NR_002201.1	4.76	Up	3.70E-13	9.98E-11
PPP1R12B	NM_032103.1	4.75	Down	8.27E-10	1.42E-08
ABCG1	NM_016818.2	4.75	Up	2.45E-12	1.61E-10
PODN	NM_153703.3	4.75	Down	1.69E-12	1.28E-10
TUBB3	NM_006086.2	4.74	Up	4.78E-13	9.50E-11
FTHL11	NR_002204.1	4.74	Up	4.48E-13	9.68E-11
SLC7A7	NM_003982.2	4.72	Up	6.16E-13	9.46E-11
MGST1	NM_145792.1	4.72	Down	8.80E-11	2.16E-09
UBN2	NM_173569.2	4.72	Down	4.01E-12	2.20E-10
BCAT1	NM_005504.4	4.70	Up	5.09E-13	9.46E-11
KYNU	NM_001032998.1	4.68	Up	6.16E-13	9.46E-11
KLRB1	NM_002258.2	4.67	Up	4.01E-12	2.20E-10
CD300LG	NM_145273.2	4.67	Down	6.29E-10	1.12E-08
ODZ3	NM_001080477.1	4.66	Down	1.39E-10	3.10E-09
KCNMA1	NM_001014797.1	4.66	Down	2.07E-13	2.02E-10
PDZRN4	NM_013377.3	4.64	Down	8.46E-13	9.64E-11
MYO1G	NM_033054.1	4.64	Up	1.58E-12	1.23E-10

CMTM7	NM_181472.1	4.62	Up	2.68E-13	1.25E-10
RORB	NM_006914.3	4.60	Down	1.31E-12	1.12E-10
CYBA	NM_000101.2	4.60	Up	5.12E-12	2.54E-10
SMTN	NM_006932.3	4.60	Down	5.78E-13	9.52E-11
IGSF9B	NM_014987.1	4.60	Down	3.05E-13	1.12E-10
CXCL9	NM_002416.1	4.59	Up	8.49E-09	1.08E-07
H19	NR_002196.1	4.59	Down	1.35E-09	2.16E-08
ABCA9	NM_080283.3	4.59	Down	9.97E-12	3.96E-10
CHST13	NM_152889.1	4.58	Up	1.35E-11	4.91E-10
F10	NM_000504.3	4.57	Down	1.82E-13	5.12E-10
HLA-DPA1	NM_033554.2	4.57	Up	4.20E-13	9.91E-11
TMEM158	NM_015444.2	4.56	Up	2.51E-13	1.34E-10
	BX097190	4.56	Down	3.24E-10	6.31E-09
ALOX5	XM_001127464.1	4.56	Up	4.48E-13	9.68E-11
CD37	NM_001774.1	4.55	Up	8.46E-13	9.64E-11
NPY5R	NM_006174.2	4.54	Down	3.05E-13	1.12E-10
CD163	NM_203416.2	4.54	Up	2.91E-11	8.97E-10
CD6	NM_006725.2	4.54	Up	1.06E-11	4.13E-10
PGM5	NM_021965.3	4.54	Down	2.86E-13	1.18E-10
SIGLEC10	NM_033130.2	4.54	Up	5.12E-12	2.54E-10
CCL3L3	NM_001001437.3	4.51	Up	2.04E-11	6.78E-10
COL11A1	NM_001854.3	4.51	Up	1.06E-11	4.13E-10
SEZ6L2	NM_201575.1	4.51	Up	4.78E-13	9.50E-11
MS4A4A	NM_148975.1	4.50	Up	4.78E-13	9.50E-11
CAST	NM_001042443.1	4.50	Down	6.53E-12	2.97E-10
EBI2	NM_004951.3	4.50	Up	5.44E-12	2.62E-10
FBLN1	NM_006486.2	4.49	Down	2.17E-11	7.10E-10
VCAM1	NM_080682.1	4.48	Up	3.33E-12	1.98E-10
CHRNA1	NM_001039523.1	4.48	Up	2.45E-10	4.97E-09
CTSB	NM_001908.3	4.47	Up	8.46E-13	9.64E-11
CD2	NM_001767.2	4.47	Up	3.77E-12	2.12E-10
LOC649143	XM_944822.1	4.47	Up	3.94E-06	2.74E-05
GK	NM_000167.3	4.45	Up	3.05E-13	1.12E-10
MELK	NM_014791.2	4.45	Up	1.27E-11	4.71E-10
FCGBP	NM_003890.1	4.44	Up	3.55E-12	2.05E-10
AIF1L	NM_031426.2	4.44	Down	3.91E-11	1.13E-09
LMOD1	NM_012134.2	4.42	Down	7.94E-13	9.69E-11
SLC38A6	NM_153811.1	4.41	Up	8.46E-13	9.64E-11
PHGDH	NM_006623.2	4.41	Down	3.05E-13	1.12E-10
EMILIN2	NM_032048.2	4.41	Up	1.09E-12	1.03E-10
ABCC3	NM_003786.2	4.40	Up	1.02E-12	1.00E-10
PALM	NM_002579.2	4.38	Down	7.83E-10	1.36E-08
C15orf48	NM_032413.2	4.38	Up	5.78E-13	9.52E-11
RAB9B	NM_016370.1	4.37	Down	1.82E-13	5.12E-10
LDOC1	NM_012317.2	4.37	Down	7.00E-13	9.64E-11
CNN1	NM_001299.4	4.36	Down	1.02E-12	1.00E-10
SORBS1	NM_001034954.1	4.36	Down	1.94E-13	2.47E-10
LYZ	NM_000239.1	4.35	Up	2.36E-13	1.49E-10
SYNPO2	XM_942780.2	4.35	Down	1.40E-12	1.15E-10
LIPA	NM_000235.2	4.34	Up	8.46E-13	9.64E-11
DMD	NM_004006.1	4.34	Down	2.45E-12	1.61E-10
	BX355045	4.34	Down	6.24E-08	6.41E-07
DDX17	NM_006386.3	4.32	Down	1.27E-11	4.71E-10
IL6	NM_000600.1	4.32	Up	2.00E-06	1.49E-05
TCEAL2	NM_080390.3	4.32	Down	1.09E-09	1.80E-08

RBPMS	NM_006867.2	4.32	Down	5.09E-13	9.46E-11
RPESP	NM_153225.2	4.31	Down	1.02E-12	1.00E-10
KCNA5	NM_002234.2	4.31	Down	7.37E-12	3.23E-10
TMEM176A	NM_018487.2	4.30	Up	3.77E-12	2.12E-10
CCL5	NM_002985.2	4.29	Up	3.68E-11	1.08E-09
ALDH1L1	NM_012190.2	4.28	Down	2.95E-12	1.82E-10
FCGR1A	NM_000566.2	4.28	Up	1.40E-12	1.15E-10
	BX093329	4.27	Up	6.15E-12	2.85E-10
PAPSS1	NM_005443.4	4.26	Up	9.39E-12	3.80E-10
DNASE2B	NM_021233.2	4.23	Up	7.40E-11	1.89E-09
CD69	NM_001781.1	4.23	Up	6.65E-10	1.18E-08
CD4	NM_000616.3	4.23	Up	9.60E-13	9.86E-11
AQP9	NM_020980.2	4.22	Up	2.59E-11	8.18E-10
XK	NM_021083.2	4.22	Down	1.16E-12	1.07E-10
DUSP26	NM_024025.1	4.22	Down	7.45E-13	9.75E-11
	CB135276	4.21	Down	1.82E-13	5.12E-10
GLT25D2	NM_015101.2	4.21	Down	4.48E-13	9.68E-11
C3AR1	NM_004054.2	4.21	Up	2.95E-12	1.82E-10
CTSS	NM_004079.3	4.19	Up	3.47E-13	1.02E-10
C3	NM_000064.1	4.19	Down	2.22E-05	0.000130498
LHFPL2	NM_005779.1	4.18	Up	4.48E-13	9.68E-11
ALPK2	NM_052947.3	4.17	Up	3.91E-11	1.13E-09
GPC3	NM_004484.2	4.17	Down	4.56E-09	6.31E-08
STS-1	NM_032873.3	4.16	Up	3.94E-13	1.00E-10
C1QB	NM_000491.3	4.14	Up	1.31E-12	1.12E-10
NAPSB	NR_002798.1	4.13	Up	1.96E-09	2.99E-08
CD53	NM_000560.3	4.13	Up	9.01E-13	9.71E-11
FHL5	NM_020482.3	4.13	Down	5.23E-11	1.43E-09
MS4A7	NM_206938.1	4.13	Up	2.68E-13	1.25E-10
EVI2A	NM_014210.2	4.12	Up	1.49E-12	1.19E-10
ZMYND15	NM_032265.1	4.11	Up	4.48E-13	9.68E-11
KRT14	NM_000526.3	4.11	Up	1.27E-11	4.71E-10
LOC653879	XM_936226.1	4.10	Down	5.96E-06	3.98E-05
NETO2	NM_018092.3	4.10	Up	2.36E-13	1.49E-10
ADAP2	NM_018404.2	4.09	Up	1.69E-12	1.28E-10
LRRN4CL	NM_203422.1	4.07	Down	2.30E-12	1.55E-10
GNA15	NM_002068.1	4.07	Up	3.70E-13	9.98E-11
MS4A6A	NM_152851.1	4.07	Up	4.26E-12	2.29E-10
CCDC149	NM_173463.2	4.07	Down	6.16E-13	9.46E-11
HLA-DRA	NM_019111.3	4.07	Up	2.51E-13	1.34E-10
RASL12	NM_016563.2	4.07	Down	9.01E-13	9.71E-11
LGR6	NM_001017404.1	4.07	Down	1.16E-12	1.07E-10
SPI1	NM_001080547.1	4.07	Up	2.95E-12	1.82E-10
CTSB	NM_147780.2	4.06	Up	3.77E-12	2.12E-10
C1orf162	NM_174896.2	4.06	Up	1.58E-12	1.23E-10
TLR7	NM_016562.3	4.06	Up	2.03E-12	1.43E-10
MT1G	NM_005950.1	4.06	Up	4.82E-12	2.46E-10
IL2RA	NM_000417.1	4.06	Up	8.46E-13	9.64E-11
RUNX3	NM_004350.2	4.06	Up	4.78E-13	9.50E-11
TIMP4	NM_003256.2	4.06	Down	4.26E-12	2.29E-10
LPAR5	NM_020400.4	4.05	Up	1.93E-11	6.49E-10
LY96	NM_015364.2	4.04	Up	3.47E-13	1.02E-10
MTP18	NM_016498.3	4.04	Up	4.20E-13	9.91E-11
RN7SK	NR_001445.1	4.04	Down	5.33E-10	9.72E-09
CD83	NM_004233.3	4.03	Up	1.43E-11	5.15E-10

FLJ20273	NM_019027.1	4.03	Up	7.94E-13	9.69E-11
SYK	NM_003177.3	4.03	Up	4.53E-12	2.38E-10
CSF2RA	NM_172249.1	4.03	Up	5.78E-12	2.74E-10
FAM49B	NM_016623.3	4.02	Up	3.55E-12	2.05E-10
GARNL3	NM_032293.3	4.02	Down	1.82E-13	5.12E-10
CD53	NM_000560.3	4.01	Up	1.49E-12	1.19E-10
EBF3	NM_001005463.1	4.01	Down	8.32E-12	3.49E-10
TBXAS1	NM_001061.2	4.01	Up	2.95E-12	1.82E-10
PILRA	NM_013439.2	3.99	Up	2.61E-12	1.67E-10
STXBP2	NM_006949.1	3.99	Up	2.77E-12	1.75E-10
	BC070337	3.98	Up	7.40E-11	1.89E-09
SVIL	NM_003174.3	3.98	Down	1.82E-13	5.12E-10
CTSH	NM_004390.2	3.98	Up	4.53E-12	2.38E-10
ART3	NM_001179.3	3.97	Down	2.45E-12	1.61E-10
MT1H	NM_005951.2	3.97	Up	9.60E-13	9.86E-11
CD8A	NM_171827.2	3.96	Up	6.57E-13	9.51E-11
GPAM	NM_020918.3	3.96	Down	1.12E-11	4.31E-10
SLC15A3	NM_016582.1	3.96	Up	5.78E-13	9.52E-11
NTRK3	NM_001007156.1	3.96	Down	1.91E-12	1.37E-10
MCOLN2	NM_153259.2	3.95	Up	5.78E-13	9.52E-11
SPON1	NM_006108.2	3.95	Down	1.28E-07	1.23E-06
CD300LF	NM_139018.2	3.95	Up	5.12E-12	2.54E-10
HOXA4	NM_002141.4	3.94	Down	1.82E-13	5.12E-10
FABP5	NM_001444.1	3.94	Up	6.94E-12	3.10E-10
MAP1B	NM_032010.1	3.93	Down	1.49E-12	1.19E-10
KCNK17	NM_031460.3	3.92	Down	8.27E-10	1.42E-08
HLA-DRB1	NM_002124.1	3.92	Up	0.000125789	0.000619193
VMO1	NM_182566.1	3.92	Up	9.01E-13	9.71E-11
PCP4	NM_006198.2	3.92	Down	7.83E-12	3.36E-10
LILRB3	NM_006864.2	3.91	Up	5.54E-11	1.50E-09
NFIC	NM_205843.1	3.91	Down	2.45E-12	1.61E-10
PCYOX1	NM_016297.2	3.91	Down	6.94E-12	3.10E-10
ANPEP	NM_001150.1	3.90	Up	1.71E-11	5.96E-10
ANKRD1	NM_014391.2	3.90	Up	3.28E-11	9.89E-10
TBC1D2	NM_018421.2	3.90	Up	9.01E-13	9.71E-11
LOC644330	XR_017492.1	3.90	Up	2.45E-12	1.61E-10
TMSB15A	NM_021992.2	3.89	Down	7.00E-13	9.64E-11
HOXA3	NM_153631.2	3.89	Down	9.60E-13	9.86E-11
RN7SK	NR_001445.1	3.89	Down	1.78E-07	1.65E-06
ST14	NM_021978.2	3.89	Up	2.61E-12	1.67E-10
HCK	NM_002110.2	3.89	Up	6.57E-13	9.51E-11
ANKRD20A1	NM_032250.1	3.89	Down	3.77E-12	2.12E-10
SLC16A9	NM_194298.1	3.88	Down	4.26E-12	2.29E-10
KLK4	NM_004917.3	3.88	Up	7.45E-13	9.75E-11
CD74	NM_001025159.1	3.87	Up	9.60E-13	9.86E-11
GPD1	NM_005276.2	3.86	Down	1.15E-09	1.88E-08
KIAA0101	NM_014736.4	3.86	Up	1.31E-10	2.97E-09
RASAL3	NM_022904.1	3.85	Up	8.46E-13	9.64E-11
MOSC1	NM_022746.2	3.85	Down	7.45E-13	9.75E-11
COL4A6	NM_001847.2	3.85	Down	1.43E-11	5.15E-10
MGAT4A	NM_012214.2	3.85	Up	4.78E-13	9.50E-11
CAMK2N1	NM_018584.5	3.85	Down	4.78E-13	9.50E-11
PRND	NM_012409.2	3.85	Up	3.14E-12	1.90E-10
CD79A	NM_021601.3	3.84	Up	1.09E-09	1.80E-08
CD8A	NM_171827.2	3.83	Up	6.16E-13	9.46E-11

CD3D	NM_001040651.1	3.83	Up	5.78E-12	2.74E-10
TNFRSF11B	NM_002546.3	3.83	Up	3.77E-12	2.12E-10
HLA-DRB6	NR_001298.1	3.83	Up	5.43E-13	9.56E-11
CKS2	NM_001827.1	3.83	Up	3.33E-12	1.98E-10
SERPINE1	NM_000602.1	3.82	Up	6.53E-12	2.97E-10
KIAA1772	NM_024935.2	3.82	Down	5.78E-13	9.52E-11
	U79293	3.81	Down	5.09E-13	9.46E-11
GPR137B	NM_003272.1	3.80	Up	4.20E-13	9.91E-11
RCOR2	NM_173587.2	3.80	Down	1.69E-12	1.28E-10
	CR596519	3.79	Up	3.28E-11	9.89E-10
GPR65	NM_003608.2	3.79	Up	6.16E-13	9.46E-11
CCL18	NM_002988.2	3.79	Up	4.26E-12	2.29E-10
SLC29A4	NM_153247.1	3.79	Down	3.20E-06	2.27E-05
MYO5A	NM_000259.2	3.79	Up	7.00E-13	9.64E-11
CSF2RA	NM_172245.1	3.78	Up	1.61E-11	5.70E-10
HLA-DQB1	NM_002123.2	3.78	Up	1.09E-06	8.55E-06
CLEC5A	NM_013252.2	3.78	Up	1.02E-12	1.00E-10
	CR743148	3.77	Up	1.82E-13	5.12E-10
PTPN22	NM_015967.3	3.76	Up	6.16E-13	9.46E-11
FAM46B	NM_052943.2	3.76	Down	3.14E-12	1.90E-10
C6orf105	NM_032744.1	3.76	Up	5.44E-12	2.62E-10
HOXC8	NM_022658.3	3.75	Down	4.65E-11	1.31E-09
TFRC	NM_003234.1	3.75	Up	1.31E-12	1.12E-10
CD3D	NM_000732.4	3.75	Up	1.71E-11	5.96E-10
CPVL	NM_019029.2	3.75	Up	1.35E-11	4.91E-10
ANXA2P1	NR_001562.1	3.75	Up	6.15E-12	2.85E-10
MMP28	NM_001032278.1	3.74	Down	3.94E-13	1.00E-10
ABCA6	NM_080284.2	3.74	Down	1.31E-12	1.12E-10
CD28	NM_006139.1	3.74	Up	7.45E-13	9.75E-11
FAIM3	NM_005449.3	3.74	Up	7.40E-11	1.89E-09
IL4I1	NM_172374.1	3.73	Up	1.82E-13	5.12E-10
SLC16A6	NM_004694.3	3.73	Up	1.31E-12	1.12E-10
ARHGAP30	NM_181720.2	3.73	Up	8.84E-12	3.64E-10
DPY19L2	NM_173812.4	3.72	Down	1.67E-09	2.59E-08
HLA-DPB1	NM_002121.4	3.72	Up	2.95E-12	1.82E-10
NFE2L3	NM_004289.5	3.71	Up	9.39E-12	3.80E-10
LOC730995	XM_001130291.1	3.71	Down	9.39E-12	3.80E-10
CDCP1	NM_178181.1	3.71	Up	3.14E-12	1.90E-10
FAM129A	NM_052966.2	3.71	Down	2.86E-13	1.18E-10
DOCK2	NM_004946.1	3.71	Up	1.16E-12	1.07E-10
GSTM5	NM_000851.2	3.71	Down	4.26E-12	2.29E-10
	AK123847	3.71	Down	1.82E-13	5.12E-10
CA2	NM_000067.1	3.70	Up	6.15E-12	2.85E-10
IL10RA	NM_001558.2	3.69	Up	5.44E-12	2.62E-10
PILRA	NM_178272.1	3.69	Up	6.53E-12	2.97E-10
	BC039516	3.69	Down	1.91E-12	1.37E-10
BCHE	NM_000055.1	3.69	Down	2.45E-10	4.97E-09
DSC2	NM_004949.2	3.69	Up	3.94E-13	1.00E-10
UCP2	NM_003355.2	3.69	Up	7.83E-12	3.36E-10
WTIP	NM_001080436.1	3.68	Down	1.31E-10	2.97E-09
CNTN4	NM_175612.1	3.68	Down	2.30E-09	3.43E-08
BTK	NM_000061.1	3.68	Up	4.78E-13	9.50E-11
RHBDF2	NM_001005498.2	3.67	Up	1.09E-12	1.03E-10
OR51E2	NM_030774.2	3.67	Down	5.62E-09	7.57E-08
LDB3	NM_007078.1	3.67	Down	2.17E-11	7.10E-10

MS4A6A	NM_152851.1	3.67	Up	2.45E-12	1.61E-10
PILRA	NM_178273.1	3.66	Up	1.02E-12	1.00E-10
JSRP1	NM_144616.2	3.66	Up	5.67E-08	5.87E-07
LEPR	NM_001003679.1	3.65	Down	1.06E-11	4.13E-10
TACC2	NM_206861.1	3.65	Down	3.25E-13	1.06E-10
SRGN	NM_002727.2	3.65	Up	9.60E-13	9.86E-11
ITGAM	NM_000632.3	3.65	Up	9.01E-13	9.71E-11
LPXN	NM_004811.1	3.65	Up	1.94E-13	2.47E-10
VIPR1	NM_004624.2	3.65	Down	1.82E-13	5.12E-10
PDGFRL	NM_006207.1	3.64	Down	2.44E-11	7.79E-10
ITGA8	NM_003638.1	3.64	Down	4.78E-10	8.81E-09
RPLP1	NM_001003.2	3.63	Up	3.33E-09	4.77E-08
GAS1	NM_002048.1	3.63	Down	3.51E-09	4.99E-08
MS4A6A	NM_022349.2	3.63	Up	7.83E-12	3.36E-10
	BU945037	3.62	Down	7.02E-10	1.23E-08
AQP7	NM_001170.1	3.62	Down	1.50E-09	2.37E-08
ACTN2	NM_001103.1	3.62	Down	7.45E-13	9.75E-11
CPVL	NM_031311.3	3.61	Up	1.05E-10	2.48E-09
HCLS1	NM_005335.3	3.61	Up	1.02E-12	1.00E-10
SLC2A3	NM_006931.1	3.61	Up	2.43E-09	3.60E-08
LOC652726	XM_942351.2	3.60	Down	3.28E-11	9.89E-10
EGR2	NM_000399.2	3.60	Up	1.21E-09	1.96E-08
GAD1	NM_000817.2	3.60	Down	9.41E-09	1.18E-07
C4orf31	NM_024574.3	3.60	Down	4.28E-10	8.00E-09
GZMA	NM_006144.2	3.60	Up	5.23E-11	1.43E-09
LIPE	NM_005357.2	3.60	Down	7.83E-10	1.36E-08
PCDH17	NM_014459.2	3.59	Down	6.94E-12	3.10E-10
SLC39A8	NM_022154.5	3.59	Up	2.45E-12	1.61E-10
PCYOX1	NM_016297.2	3.59	Down	5.09E-13	9.46E-11
KCNAB1	NM_003471.2	3.58	Down	6.15E-12	2.85E-10
	CR616845	3.57	Down	2.95E-12	1.82E-10
ZNF385A	NM_015481.1	3.57	Up	4.01E-12	2.20E-10
PIM1	NM_002648.2	3.57	Up	8.80E-11	2.16E-09
SLA	NM_001045557.1	3.57	Up	3.25E-13	1.06E-10
LACTB	NM_032857.2	3.57	Up	4.78E-13	9.50E-11
SAT1	NM_002970.1	3.57	Up	5.78E-13	9.52E-11
	AL133627	3.57	Down	9.60E-13	9.86E-11
NTNG1	NM_014917.2	3.56	Down	8.32E-12	3.49E-10
CSK	NM_004383.1	3.56	Up	7.00E-13	9.64E-11
	BX537823	3.56	Down	5.78E-13	9.52E-11
AMPH	NM_001635.2	3.55	Down	2.95E-12	1.82E-10
FREQ	NM_014286.2	3.55	Down	4.20E-13	9.91E-11
ITGB7	NM_000889.1	3.55	Up	9.39E-12	3.80E-10
LEFTY2	NM_003240.2	3.55	Down	1.67E-09	2.59E-08
TPR	NM_003292.2	3.55	Down	4.39E-05	0.000242165
SVEP1	NM_153366.2	3.55	Down	6.88E-08	7.00E-07
HRCT1	NM_001039792.1	3.54	Down	1.06E-11	4.13E-10
BATF	NM_006399.2	3.54	Up	1.58E-12	1.23E-10
TNFRSF21	NM_014452.3	3.54	Up	3.55E-12	2.05E-10
IL17D	NM_138284.1	3.53	Down	8.46E-13	9.64E-11
HIF1A	NM_001530.2	3.53	Up	6.15E-12	2.85E-10
ECT2	NM_018098.4	3.52	Up	1.31E-12	1.12E-10
WAS	NM_000377.1	3.51	Up	3.33E-12	1.98E-10
CD74	NM_004355.2	3.51	Up	4.82E-12	2.46E-10
M160	NM_174941.3	3.50	Up	1.58E-12	1.23E-10

ARPC3	NM_005719.2	3.50	Up	5.78E-13	9.52E-11
SHISA3	NM_001080505.1	3.50	Up	7.83E-12	3.36E-10
GSN	NM_000177.3	3.50	Down	5.95E-08	6.13E-07
FLJ13305	NM_032180.1	3.49	Down	2.51E-13	1.34E-10
	AK094929	3.49	Down	3.33E-12	1.98E-10
C9orf61	NM_004816.2	3.48	Down	6.16E-13	9.46E-11
ADARB1	NM_015833.2	3.48	Down	5.44E-12	2.62E-10
OSBPL3	NM_145322.1	3.48	Up	3.47E-13	1.02E-10
RNASE2	NM_002934.2	3.47	Up	9.41E-09	1.18E-07
LOC652377	XR_019346.1	3.47	Down	5.12E-12	2.54E-10
RGS16	NM_002928.2	3.47	Up	5.62E-09	7.57E-08
NLRP3	NM_001079821.1	3.46	Up	6.94E-12	3.10E-10
RAD51AP1	NM_006479.3	3.46	Up	2.30E-12	1.55E-10
APCDD1	NM_153000.3	3.46	Down	1.02E-12	1.00E-10
VIT	NM_053276.2	3.46	Down	2.61E-12	1.67E-10
CLLU1OS	NM_001025232.1	3.45	Up	2.05E-07	1.87E-06
GPR84	NM_020370.1	3.44	Up	4.20E-13	9.91E-11
LGI1	NM_005097.1	3.44	Down	7.83E-12	3.36E-10
SASH3	NM_018990.2	3.44	Up	4.82E-12	2.46E-10
EVI2B	NM_006495.3	3.44	Up	6.15E-12	2.85E-10
LOC644322	XM_933391.1	3.44	Down	7.02E-10	1.23E-08
GPR20	NM_005293.2	3.43	Down	1.82E-13	5.12E-10
CD37	NM_001774.2	3.43	Up	5.12E-12	2.54E-10
HMGA1	NM_145899.1	3.43	Up	3.33E-12	1.98E-10
PSCD4	NM_013385.2	3.42	Up	1.23E-12	1.10E-10
CD72	NM_001782.1	3.41	Up	7.94E-13	9.69E-11
TMEM26	NM_178505.5	3.41	Up	2.51E-13	1.34E-10
FAM49B	NM_016623.3	3.41	Up	2.61E-12	1.67E-10
G3BP1	NM_005754.2	3.41	Up	1.24E-10	2.83E-09
RGS4	NM_005613.3	3.41	Up	1.39E-10	3.10E-09
FABP5	NM_001444.1	3.40	Up	3.68E-11	1.08E-09
ANKS1B	NM_181670.2	3.40	Down	1.23E-12	1.10E-10
GPR65	NM_003608.2	3.39	Up	4.78E-13	9.50E-11
CTSL1	NM_001912.3	3.39	Up	7.45E-13	9.75E-11
CD84	NM_003874.1	3.39	Up	7.40E-11	1.89E-09
PLXNB1	NM_002673.3	3.38	Down	2.36E-13	1.49E-10
LILRB2	NM_001080978.1	3.38	Up	1.93E-11	6.49E-10
LGALS9	NM_009587.2	3.38	Up	1.56E-10	3.40E-09
MARCO	NM_006770.3	3.38	Up	7.02E-10	1.23E-08
CRTAC1	NM_018058.4	3.38	Up	7.94E-08	7.97E-07
ATOH8	NM_032827.4	3.38	Down	1.35E-11	4.91E-10
DHRS9	NM_005771.3	3.37	Up	1.15E-09	1.88E-08
RNF165	NM_152470.2	3.37	Down	1.06E-11	4.13E-10
SRPK3	NM_014370.2	3.37	Down	1.80E-12	1.33E-10
SLIT2	NM_004787.1	3.37	Down	9.01E-13	9.71E-11
PGM5	NM_021965.3	3.37	Down	2.68E-13	1.25E-10
MAOA	NM_000240.2	3.37	Down	7.00E-13	9.64E-11
TMOD1	NM_003275.2	3.37	Down	4.48E-13	9.68E-11
DENND2A	NM_015689.2	3.36	Down	2.75E-11	8.58E-10
RHBDF2	NM_024599.3	3.36	Up	2.16E-12	1.48E-10
GRN	NM_002087.2	3.36	Up	3.05E-13	1.12E-10
PGM5	NM_021965.3	3.35	Down	2.86E-13	1.18E-10
QPCT	NM_012413.3	3.34	Up	4.53E-12	2.38E-10
DKFZp451A211	NM_001003399.1	3.34	Down	5.05E-10	9.26E-09
FAM149A	NM_015398.2	3.34	Down	2.36E-13	1.49E-10

CD14	NM_001040021.1	3.34	Up	3.70E-13	9.98E-11
FERMT3	NM_031471.4	3.34	Up	9.01E-13	9.71E-11
HIF1A	NM_001530.2	3.34	Up	5.12E-12	2.54E-10
CTSC	NM_001814.2	3.33	Up	6.94E-12	3.10E-10
JPH2	NM_020433.4	3.33	Down	3.91E-11	1.13E-09
RORA	NM_002943.2	3.32	Down	8.27E-10	1.42E-08
GPX1	NM_201397.1	3.32	Up	5.09E-13	9.46E-11
DBC1	NM_014618.2	3.31	Up	1.34E-07	1.28E-06
FTHL12	NR_002205.1	3.31	Up	1.91E-12	1.37E-10
MPDZ	NM_003829.3	3.31	Down	9.60E-13	9.86E-11
PYCARD	NM_013258.3	3.31	Up	2.16E-12	1.48E-10
OAS2	NM_016817.2	3.31	Up	3.47E-11	1.03E-09
EBF2	NM_022659.2	3.30	Down	1.27E-11	4.71E-10
CYBB	NM_000397.2	3.29	Up	1.65E-10	3.57E-09
GBP5	NM_052942.2	3.29	Up	4.65E-11	1.31E-09
FABP4	NM_001442.1	3.29	Down	1.86E-07	1.72E-06
NLGN4X	NM_020742.2	3.29	Up	8.80E-11	2.16E-09
SPOCK2	NM_014767.1	3.29	Up	2.61E-12	1.67E-10
LOC653888	XM_936251.2	3.28	Up	5.43E-13	9.56E-11
CAMK2G	NM_001222.2	3.28	Down	2.68E-13	1.25E-10
CACNB2	NM_201572.1	3.28	Down	2.36E-13	1.49E-10
PDZD2	NM_178140.2	3.28	Down	7.45E-13	9.75E-11
P2RX1	NM_002558.2	3.28	Down	1.15E-09	1.88E-08
ITPRIPL2	NM_001034841.2	3.28	Down	8.27E-10	1.42E-08
DENND2D	NM_024901.3	3.28	Up	2.59E-11	8.18E-10
BHMT2	NM_017614.3	3.28	Down	3.77E-12	2.12E-10
AIF1	NM_032955.1	3.28	Up	4.01E-12	2.20E-10
TNC	NM_002160.2	3.27	Up	3.31E-08	3.62E-07
LGMN	NM_001008530.1	3.27	Up	3.77E-12	2.12E-10
CCL13	NM_005408.2	3.27	Up	1.96E-09	2.99E-08
NFAT5	NM_173215.1	3.27	Down	1.35E-11	4.91E-10
NUSAP1	NM_018454.5	3.27	Up	2.45E-10	4.97E-09
AAK1	NM_014911.3	3.27	Down	3.83E-10	7.27E-09
BOC	NM_033254.2	3.27	Down	6.99E-11	1.81E-09
MXRA5	NM_015419.2	3.27	Up	1.49E-12	1.19E-10
PAK2	XM_001126110.1	3.27	Down	8.07E-09	1.04E-07
TM6SF1	NM_023003.2	3.27	Up	2.61E-12	1.67E-10
CFD	NM_001928.2	3.26	Down	5.12E-12	2.54E-10
RASGRP3	NM_170672.1	3.26	Up	3.68E-11	1.08E-09
HOXA2	NM_006735.3	3.26	Down	2.31E-10	4.74E-09
SPOCD1	NM_144569.4	3.26	Up	6.15E-12	2.85E-10
SORBS2	NM_003603.4	3.26	Down	9.39E-12	3.80E-10
KANK2	NM_015493.4	3.26	Down	7.84E-11	1.98E-09
LPHN3	NM_015236.3	3.26	Down	3.42E-10	6.61E-09
GM2A	NM_000405.3	3.26	Up	1.02E-12	1.00E-10
ANXA2P1	NR_001562.1	3.26	Up	3.77E-12	2.12E-10
ZKSCAN1	NM_003439.1	3.25	Down	8.84E-12	3.64E-10
FTHL12	NR_002205.1	3.25	Up	4.48E-13	9.68E-11
PPFIBP1	NM_177444.1	3.25	Down	1.74E-10	3.74E-09
SMPDL3A	NM_006714.2	3.25	Up	6.53E-12	2.97E-10
SOD2	NM_001024465.1	3.25	Up	9.39E-12	3.80E-10
C3orf70	NM_001025266.1	3.25	Down	5.09E-13	9.46E-11
CHRDL1	NM_145234.2	3.25	Down	1.61E-11	5.70E-10
BVES	NM_147147.2	3.25	Down	9.01E-13	9.71E-11
JPH2	NM_175913.3	3.24	Down	1.40E-12	1.15E-10

ACACB	NM_001093.3	3.24	Down	7.45E-13	9.75E-11
LOC283050	XM_944265.1	3.24	Up	1.27E-11	4.71E-10
MARK1	NM_018650.3	3.24	Down	8.46E-13	9.64E-11
LOC650580	XM_942530.1	3.24	Down	6.57E-13	9.51E-11
DCN	NM_001920.3	3.24	Down	7.22E-08	7.31E-07
PALM	NM_002579.2	3.24	Down	3.06E-10	6.01E-09
GRN	NM_002087.2	3.24	Up	5.78E-13	9.52E-11
ZBTB16	NM_006006.4	3.23	Down	8.80E-11	2.16E-09
MEIS2	NM_172315.1	3.23	Down	2.36E-13	1.49E-10
IL18BP	NM_173042.2	3.23	Up	7.94E-13	9.69E-11
PCDH19	NM_020766.1	3.23	Down	6.15E-12	2.85E-10
CTHRC1	NM_138455.2	3.22	Up	5.78E-13	9.52E-11
SEMA6D	NM_153619.1	3.22	Down	1.23E-12	1.10E-10
HAPLN1	NM_001884.2	3.22	Up	1.34E-08	1.63E-07
SDSL	NM_138432.2	3.22	Up	8.46E-13	9.64E-11
MYO1F	NM_012335.2	3.22	Up	3.68E-11	1.08E-09
CD209	NM_021155.2	3.22	Up	2.30E-09	3.43E-08
PTPN6	NM_080548.3	3.21	Up	7.83E-12	3.36E-10
DARC	NM_002036.2	3.21	Down	3.94E-06	2.74E-05
C5orf23	NM_024563.3	3.21	Down	8.80E-11	2.16E-09
SPI1	NM_003120.2	3.21	Up	8.32E-12	3.49E-10
PIK3AP1	NM_152309.2	3.21	Up	1.81E-11	6.22E-10
	DA371742	3.21	Down	1.39E-10	3.10E-09
PIP5K1B	NM_003558.1	3.20	Down	1.82E-13	5.12E-10
FUCA1	NM_000147.3	3.20	Up	1.23E-12	1.10E-10
RNASET2	NM_003730.3	3.19	Up	7.94E-13	9.69E-11
ATF3	NM_001040619.1	3.19	Up	1.69E-06	1.27E-05
SF1	NM_201997.1	3.19	Down	5.44E-12	2.62E-10
PMP2	NM_002677.3	3.19	Down	2.46E-07	2.22E-06
CAMK1G	NM_020439.2	3.19	Up	2.85E-08	3.18E-07
ANGPTL1	NM_004673.3	3.18	Down	2.03E-12	1.43E-10
SPHKAP	NM_030623.2	3.18	Down	7.42E-10	1.29E-08
GPER	NM_001039966.1	3.18	Down	1.02E-12	1.00E-10
MAPK4	NM_002747.2	3.18	Down	9.60E-13	9.86E-11
AADACL1	NM_020792.3	3.18	Up	4.78E-13	9.50E-11
HLA-DMA	NM_006120.2	3.18	Up	1.23E-12	1.10E-10
C13orf15	NM_014059.2	3.18	Up	1.71E-11	5.96E-10
	BU587445	3.17	Down	7.40E-11	1.89E-09
LOC650695	XM_939781.1	3.17	Down	1.91E-08	2.23E-07
MT1F	NM_005949.2	3.17	Up	1.16E-12	1.07E-10
SLC29A3	NM_018344.3	3.16	Up	4.53E-12	2.38E-10
LOC441155	XM_926112.2	3.16	Down	5.23E-11	1.43E-09
LOC387841	XM_932678.1	3.16	Up	9.60E-13	9.86E-11
SYNPO2	XM_936336.2	3.16	Down	1.11E-10	2.59E-09
NP	NM_000270.1	3.15	Up	6.29E-10	1.12E-08
CLECL1	NM_172004.2	3.15	Up	6.65E-10	1.18E-08
ITIH4	NM_002218.3	3.15	Down	1.39E-10	3.10E-09
ROR1	NM_005012.2	3.15	Down	1.81E-11	6.22E-10
PVRL3	NM_015480.1	3.15	Down	2.51E-13	1.34E-10
MS4A14	NM_032597.3	3.15	Up	2.59E-10	5.22E-09
NRIP3	NM_020645.1	3.15	Up	2.74E-10	5.48E-09
ST8SIA4	NM_005668.3	3.15	Up	2.86E-13	1.18E-10
WTIP	XM_059037.7	3.14	Down	5.78E-13	9.52E-11
BEX5	NM_001012978.1	3.14	Down	9.39E-12	3.80E-10
THY1	NM_006288.2	3.14	Up	7.37E-12	3.23E-10

RUNX1	NM_001754.3	3.13	Up	4.20E-13	9.91E-11
SPIN1	NM_006717.2	3.13	Down	4.93E-11	1.37E-09
CD33	NM_001772.3	3.12	Up	2.04E-11	6.78E-10
GPER	NM_001039966.1	3.12	Down	2.51E-13	1.34E-10
KRT86	NM_002284.3	3.12	Up	4.90E-08	5.15E-07
RAPH1	NM_213589.1	3.12	Up	3.68E-11	1.08E-09
GPR68	NM_003485.3	3.12	Up	7.94E-13	9.69E-11
CCL3L1	NM_021006.4	3.12	Up	1.67E-09	2.59E-08
	BG221407	3.11	Down	2.11E-08	2.44E-07
PLTP	NM_006227.2	3.11	Up	7.83E-10	1.36E-08
CCL3L1	NM_021006.4	3.11	Up	1.14E-06	8.91E-06
C19orf59	NM_174918.2	3.11	Up	1.58E-09	2.48E-08
MN1	NM_002430.2	3.11	Down	2.17E-11	7.10E-10
FGR	NM_001042729.1	3.10	Up	1.35E-11	4.91E-10
CD38	NM_001775.2	3.10	Up	4.33E-09	6.02E-08
PMP22	NM_000304.2	3.10	Down	2.07E-10	4.32E-09
WFDC1	NM_021197.2	3.10	Down	8.49E-09	1.08E-07
C19orf10	NM_019107.3	3.10	Up	7.40E-11	1.89E-09
LOC652545	XM_942035.1	3.10	Down	4.20E-13	9.91E-11
MAFB	NM_005461.3	3.09	Up	1.23E-12	1.10E-10
OSR1	NM_145260.2	3.09	Down	2.45E-10	4.97E-09
FTHL2	NR_002200.1	3.09	Up	1.27E-11	4.71E-10
ZNF565	NM_152477.2	3.09	Down	3.33E-12	1.98E-10
MYOT	NM_006790.1	3.09	Down	3.91E-11	1.13E-09
CSTB	NM_000100.2	3.08	Up	3.25E-13	1.06E-10
TMEM163	NM_030923.3	3.08	Up	7.00E-13	9.64E-11
AMY1A	NM_004038.3	3.08	Up	2.90E-10	5.74E-09
ST8SIA4	NM_005668.3	3.08	Up	7.45E-13	9.75E-11
ZBTB16	NM_001018011.1	3.08	Down	4.65E-11	1.31E-09
CTSL1	NM_145918.2	3.08	Up	4.48E-13	9.68E-11
C14orf132	NM_020215.2	3.08	Down	1.19E-11	4.51E-10
CHRNA1	NM_000079.2	3.07	Up	1.42E-09	2.26E-08
MYOZ1	NM_021245.2	3.07	Down	2.61E-12	1.67E-10
HPSE	NM_006665.3	3.07	Up	2.45E-12	1.61E-10
NCF4	NM_000631.3	3.07	Up	2.95E-12	1.82E-10
HSPB7	NM_014424.3	3.07	Down	1.21E-08	1.49E-07
LST1	NM_007161.2	3.07	Up	8.80E-11	2.16E-09
NT5DC3	NM_016575.1	3.06	Down	8.84E-12	3.64E-10
FREM1	NM_144966.4	3.06	Down	5.09E-13	9.46E-11
PPFIBP1	NM_003622.2	3.06	Down	6.94E-12	3.10E-10
LIMS1	NM_004987.3	3.06	Down	6.99E-11	1.81E-09
UBE2C	NM_181803.1	3.06	Up	2.30E-11	7.44E-10
S100A9	NM_002965.2	3.06	Up	5.07E-09	6.91E-08
CLEC4A	NM_194447.2	3.05	Up	3.77E-12	2.12E-10
ALOX5AP	NM_001629.2	3.05	Up	3.05E-13	1.12E-10
IGF2	NM_000612.2	3.05	Down	1.27E-11	4.71E-10
RRM2B	NM_015713.3	3.05	Up	3.47E-11	1.03E-09
GDF15	NM_004864.1	3.05	Up	6.57E-13	9.51E-11
C1orf165	NM_024603.1	3.05	Down	9.97E-12	3.96E-10
DCLK1	NM_004734.2	3.04	Down	1.27E-11	4.71E-10
UPP1	NM_003364.2	3.04	Up	2.16E-12	1.48E-10
TSPAN13	NM_014399.3	3.04	Up	2.07E-10	4.32E-09
OTC	NM_000531.3	3.04	Down	3.42E-10	6.61E-09
MYO3A	NM_017433.3	3.04	Down	7.00E-13	9.64E-11
HLA-DRA	NM_019111.3	3.04	Up	8.46E-13	9.64E-11

C1QA	NM_015991.1	3.04	Up	8.84E-12	3.64E-10
EBI2	NM_004951.3	3.03	Up	9.87E-11	2.37E-09
GPR125	XM_944791.1	3.03	Down	4.48E-13	9.68E-11
ARHGAP9	NM_001080157.1	3.03	Up	7.37E-12	3.23E-10
ESM1	NM_007036.2	3.03	Up	1.56E-10	3.40E-09
TOP2A	NM_001067.2	3.03	Up	5.64E-10	1.02E-08
ERP27	NM_152321.1	3.03	Up	1.69E-12	1.28E-10
FAM162B	NM_001085480.1	3.03	Down	1.22E-07	1.18E-06
LAT2	NM_032464.2	3.03	Up	8.80E-11	2.16E-09
TNNT3	NM_001042780.1	3.03	Down	1.23E-12	1.10E-10
C17orf60	NM_001085423.1	3.02	Up	7.42E-10	1.29E-08
FNDC5	NM_153756.1	3.02	Down	7.37E-12	3.23E-10
TMEM149	NM_024660.2	3.02	Up	4.26E-12	2.29E-10
CDH11	NM_001797.2	3.02	Up	1.94E-13	2.47E-10
TRPV1	NM_080705.3	3.02	Down	5.78E-13	9.52E-11
AADAT	NM_182662.1	3.01	Down	2.16E-12	1.48E-10
ATP6V0B	NM_004047.3	3.01	Up	2.36E-13	1.49E-10
RCAN1	NM_203418.1	3.01	Up	7.94E-08	7.97E-07
SMAD9	NM_005905.3	3.01	Down	3.70E-13	9.98E-11
ACTR3	NM_005721.3	3.00	Up	4.93E-11	1.37E-09
MCTS1	NM_014060.1	3.00	Up	6.22E-11	1.65E-09
