# Supplementary material for: A Plasma Extracellular Vesicle-Derived microRNA Signature as a Potential Biomarker for Subclinical Coronary Atherosclerosis
Source: Int J Mol Sci. 2025 Sep 7;26(17):8727. doi: 10.3390/ijms26178727 (PMC12428963; doi:10.3390/ijms26178727)
Supplement: Supplementary file 1 [file ijms-26-08727-s001.zip › Table_S5.pdf]

Supplementary Table S5. Mature sequences of selected plasma EV-derived miRNAs.

miRNA	Mature sequence (miRBase)
hsa-miR-146b-5p	UGAGAACUGAAUUCCAUAGGCUG
hsa-miR-379-5p	UGGUAGACUAUGGAACGUAGG
hsa-miR-487b-3p	AAUCGUACAGGGUCAUCCACUU
hsa-miR-6849-5p	GAGUGGAUAGGGGAGUGUGUGGA
hsa-miR-4701-3p	AUGGGUGAUGGGUGUGGUGU
hsa-miR-1180-3p	UUUCCGGCUCGCGUGGGUGUGU
